# Catalytic defluorinative ketyl–olefin coupling by halogen-atom transfer†

**DOI:** 10.1039/d2sc02732a

**Published:** 2022-06-10

**Authors:** Peter Bellotti, Huan-Ming Huang, Teresa Faber, Ranjini Laskar, Frank Glorius

**Affiliations:** Westfälische Wilhelms-Universität Münster, Organisch-Chemisches Institut Corrensstraße 36 48149 Münster Germany glorius@uni-muenster.de; School of Physical Science and Technology, ShanghaiTech University Shanghai 201210 China huanghm@shanghaitech.edu.cn

## Abstract

Ketyl–olefin coupling reactions stand as one of the fundamental chemical transformations in synthetic chemistry and have been widely employed in the generation of complex molecular architectures and natural product synthesis. However, catalytic ketyl–olefin coupling, until the recent development of photoredox chemistry and electrosynthesis through single-electron transfer mechanisms, has remained largely undeveloped. Herein, we describe a new approach to achieve catalytic ketyl–olefin coupling reactions by a halogen-atom transfer mechanism, which provides innovative and efficient access to various *gem*-difluorohomoallylic alcohols under mild conditions with broad substrate scope. Preliminary mechanistic experimental and computational studies demonstrate that this radical-to-polar crossover transformation could be achieved by sequentially orchestrated Lewis acid activation, halogen-atom transfer, radical addition, single-electron reduction and β-fluoro elimination.

## Abstract 2

A catalytic ketyl–olefin coupling reaction including sequentially orchestrated Lewis acid activation, halogen-atom transfer, radical addition, single-electron reduction and β-fluoro elimination has been developed.

## Graphical abstract

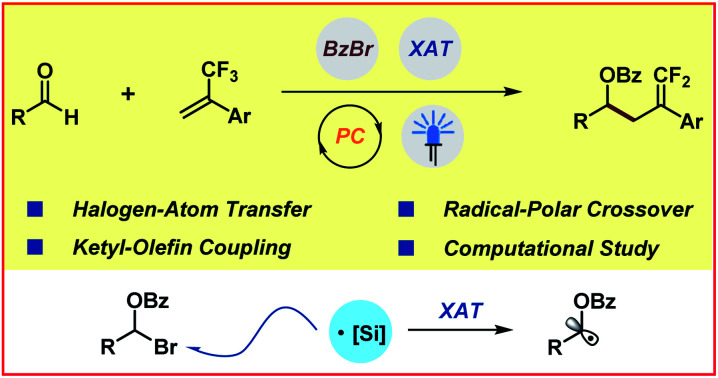

## Introduction

**Scheme 1:**
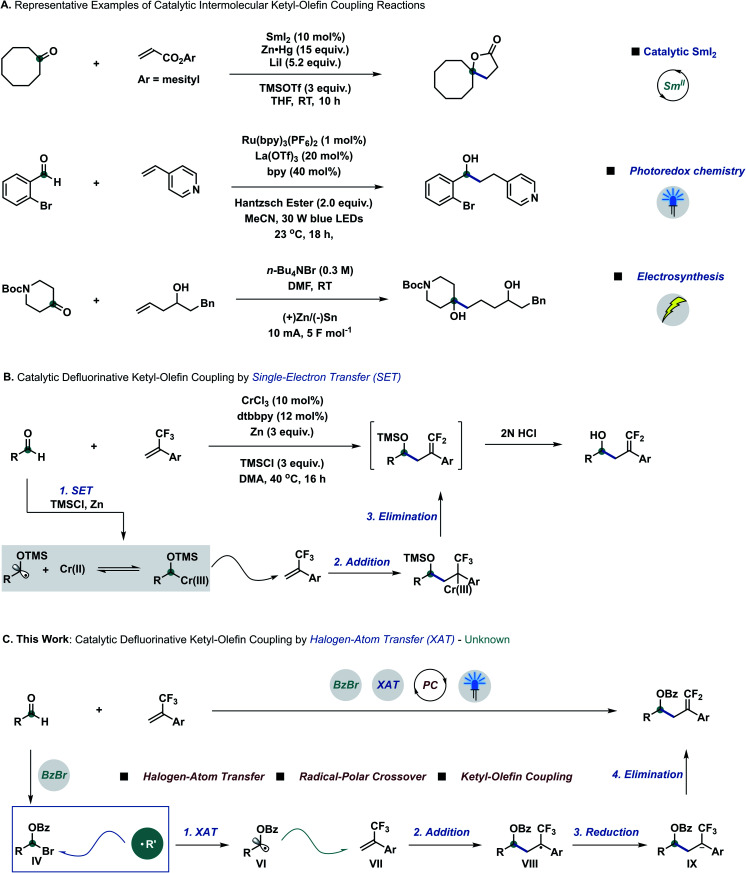
State-of-the-art of ketyl–olefin coupling reactions and our design approach enabled by halogen-atom transfer. (A) Selected examples of intermolecular ketyl–olefin coupling by means of catalytic SmI_2_, photoredox catalysis and electrosynthesis. (B) Report of defluorinative ketyl–olefin coupling *via* single-electron transfer. (C) Our strategy to achieve the mild defluorinative ketyl–olefin coupling *via* halogen-atom transfer. bpy = 2,2′-bipyridine; DMA = *N*,*N*-dimethylacetamide; DMF = *N*,*N*-dimethylformamide; dtbbpy = 4,4′-di-*tert*-butyl-2,2′-dipyridyl; PC = photocatalysis; THF = tetrahydrofuran; TMS = trimethylsilyl; RT = room temperature; XAT = halogen-atom abstraction.

Developing catalytic chemical transformations that bypass stoichiometric—and often harsh—reagents stands as a pillar principle of green chemistry.^1^ Across the plethora of fundamental functional groups, carbonyl groups arguably rank amongst the most useful synthetic building blocks to efficiently construct alcohol derivatives.^2^ Extending the repertoire of the well-established Grignard addition^3^ and Nozaki–Hiyama–Kishi (NHK) reaction,^4^ the carbonyl group has also been employed to generate ketyl radicals through single-electron transfer (SET).^5^ Since their early discovery by Corey and Pyne,^6^ ketyl–olefin coupling reactions have surged to become a popular means of constructing alcohol derivatives through carbon–carbon bond formation, despite mandating stoichiometric amounts of reductant and harsh conditions.^7^ Spurred by Corey,^8*a*^ catalytic ketyl–olefin coupling reactions have been developed by different research groups all over the world through visible light photocatalysis,^8*b*–*g*^ electrosynthesis^8*h*^ or radical relay strategies^8*i*–*l*^ (Scheme 1A).

Radical-to-polar crossover reactions—which intertwine single- and two-electron chemistry—have become an emerging synthetic tool to overcome the intrinsic limitations of traditional radical and polar chemistry,^9^ especially due to the rapid development of photoredox chemistry.^10^ Organofluorine motifs, owing to their unique reactivity, stability, and biological properties, have gained preeminent importance as building blocks in medicinal and agricultural chemistry.^11*a*^ α-Trifluoromethylstyrene derivatives are versatile synthetic intermediates for the construction of *gem*-difluoroalkene compounds, attainable through radical-to-polar crossover manifolds under mild conditions.^11*b*–*i*^ Due to the high reduction potential of aliphatic aldehydes,^12^ defluorinative ketyl–olefin coupling was only achieved very recently through single-electron transfer by using catalytic amounts of chromium (Wang),^13*a*^ iron (Wang)^13*b*^ and nickel (Montgomery)^13*c*^ with stoichiometric reducing metals and additives (Scheme 1B).

Halogen-atom transfer (XAT) can be leveraged in synthetic chemistry to efficiently generate carbon centred radical species from organohalides.^14^ MacMillan^15*a*,*b*^ and other research groups^15*c*–*e*^ have recently merged the concept of XAT into metallaphotoredox chemistry.^15*f*^ More recently, Leonori^16*a*,*b*^ and Doyle^16*c*^ discovered that aminoalkyl radicals could be used as a new type of halogen-atom abstracting reagent to form carbon based radical species from alkyl and aryl halides, to be further employed in related arylation,^16*d*^ amination,^16*e*^ hydroxymethylation^16*f*^ and elimination^16*g*^ processes. We questioned whether aldehydes could be directly employed as radical precursors to achieve carbon based radical intermediates through a XAT mechanism, prospectively extending the library of radical precursors besides overcoming the limitation of high reduction potentials. According to this hypothesis, we proposed that α-bromo alkyl intermediate IV could be formed *in situ* by reacting aldehydes and benzoyl bromide with catalytic amounts of Lewis acid.^17^ Pioneering work by Nagib and co-workers demonstrated that α-oxy halides can productively generate α-oxy radicals upon XAT using manganese metallaradicals in an overall atom-transfer catalytic cycle.^17*d*,*e*^ We posited that α-oxy bromide IV could react with suitable XAT reagents to generate α-oxy radical species VI,^14^ to be trapped by α-trifluoromethylstyrene derivatives VII to form radical intermediates VIII.^11*b*–*i*^ After sequential single-electron reduction by a suitable photocatalyst and β-fluoro elimination, the final ketyl–olefin coupling products could be obtained (Scheme 1C).

## Results and discussion

### Reaction design and optimization

Based on our concept and previous studies, hexanal 1a and α-trifluoromethylstyrene 2a were investigated to explore the catalytic defluorinative ketyl–olefin coupling reaction (Table 1). Pleasingly, coupling product 3 could be obtained in 74% isolated yield with 5 mol% 4CzIPN, 1.5 equiv. of (TMS)_3_SiOH and 4 equiv. of KOAc under visible light conditions (entry 1). Several bases were screened, indicating KOAc to be the most suitable base (entries 2–7). Highly oxidizing iridium based photocatalysts PC2 and PC3 were also investigated, but delivered lower yields (entries 8 and 9). When commercially available (TMS)_3_SiH was used in our system, only 53% yield of 3 was obtained (entry 10). Several control experiments also indicated that a photocatalyst, silane reagent, base and visible light are necessary to achieve the ketyl–olefin coupling (entry 11). Inspired by the work of Leonori^16*a*,*b*,*d*–*g*^ and Doyle,^16*c*^ we tested their standard conditions (entry 12) and other amines (entry 13).

**Table 1:** Optimization table and sensitivity screening

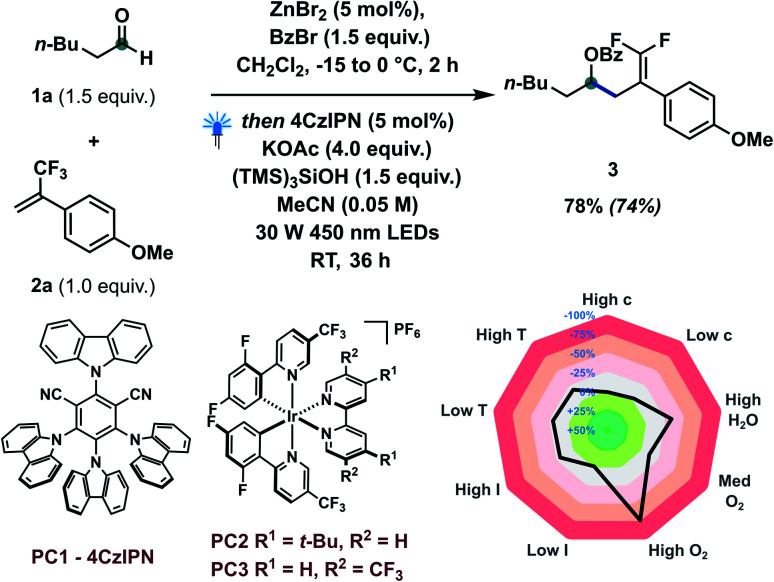		
Entry	Deviation from standard conditions	Yield^a^ (%)
1	None	78 (74)^b^
2	Na_2_CO_3_ instead of KOAc	65
3	K_2_CO_3_ instead of KOAc	70
4	K_3_PO_4_ instead of KOAc	71
5	Na_2_HPO_4_ instead of KOAc	57
6	NaOAc instead of KOAc	62
7	NaHCO_3_ instead of KOAc	53
8	1 mol% PC2 instead of 5 mol% PC1	64
9	1 mol% PC3 instead of 5 mol% PC1	28
10	(TMS)_3_SiH instead of (TMS)_3_SiOH	53
11	Without 4CzIPN or (TMS)_3_SiOH or KOAc or light	0
12	Bn_3_N instead of (TMS)_3_SiOH, MeCN : H_2_O (9 : 1)	20
13	Et_3_N or DIPEA instead of (TMS)_3_SiOH	0
14	With 2 equiv. of TEMPO	0

a Yields were determined by ^1^H NMR using dibromomethane as an internal standard.

b Isolated yield in parentheses. *c* = concentration; DIPEA = *N*,*N*-diisopropylethylamine; *I* = light intensity; *T* = temperature; TEMPO = (2,2,6,6-tetramethylpiperidin-1-yl)oxyl.

Unfortunately, only 20% yield was detected using tribenzylamine as a XAT reagent. We observed that a relatively low concentration (0.05 M) offered improved reaction yield, likely due to maximization of the MeCN : CH_2_Cl_2_ ratio. When two equivalents of TEMPO were added to the standard conditions, no desired product was generated, which indicated that the transformation may proceed through a radical mechanism (entry 14). Condition-based sensitivity screening^18^ was also applied in our current catalytic approach and showed that the reaction is sensitive to water, oxygen and lower reaction temperature.

### Synthetic scope

**Scheme 2:**
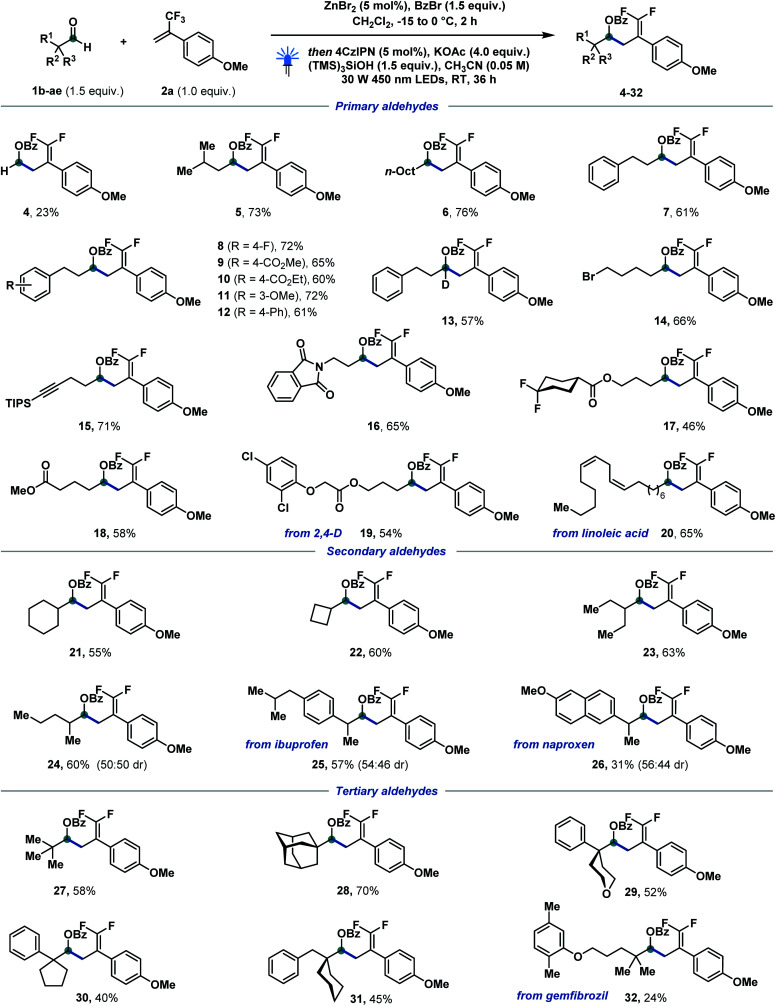
Reaction scope of α-primary, -secondary and -tertiary aldehydes. Reaction conditions: 1b–1ae (0.3 mmol, 1.5 equiv.), benzoyl bromide (0.3 mmol, 1.5 equiv.), ZnBr_2_ (0.01 mmol, 5 mol%), CH_2_Cl_2_ (1 M), −15 to 0 °C, 2 h, then 2a (0.2 mmol, 1.0 equiv.), 4CzIPN (0.01 mmol, 5 mol%), KOAc (0.8 mmol, 4.0 equiv.), (TMS)_3_SiOH (0.3 mmol, 1.5 equiv.), MeCN (0.05 M), 30 W 450 nm LEDs, RT, 36 h. For experimental details, see the ESI.†

With the optimized conditions in hand, we started to investigate the reaction scope of the catalytic defluorinative ketyl–olefin coupling reaction (Scheme 2). Firstly, different α-primary aldehydes were screened, and the corresponding coupling products 4–20 could be obtained in moderate to excellent yields. For example, paraformaldehyde (4), isopentyl aldehyde (5), and *n*-nonyl (6) and phenyl-substituted aliphatic aldehydes (7–13) were all well tolerated. Substituted phenylpropanal, functionalised with fluoro (8), ester (9, 10), methoxy (11) and phenyl (12) groups reacted equally well in 60–72% yield. Esters bearing *gem*-difluoro (17) and aliphatic chains (18) reacted in 46–58% yield. Aliphatic aldehydes bearing bromide (14), alkyne (15), imide (16), chloride (19) and alkene (20) substituents were all tolerated in the newly developed system. In particular, compounds derived from herbicide 2,4-D (19) and linoleic acid (20) testify to the appeal of the strategy towards the selective modification of complex scaffolds. Interestingly, a deuterated compound (13)—otherwise step-intensive to achieve—was also efficiently formed in 57% isolated yield. Furthermore, a variety of α-secondary aldehydes, both cyclic and acyclic, were also investigated (21–26). Cyclohexylcarbaldehyde (21) and small ring cyclobutylcarbaldehyde (22) reacted in moderate yields. An acyclic 2-methyl (24) substituted aldehyde delivered the corresponding difluoroalkene in 60% yield. Notably, aldehydes derived from non-steroidal anti-inflammatory drugs ibuprofen and naproxen were also tolerated, generating 25 and 26 in 57 and 31% isolated yield, respectively. Finally, sterically hindered tertiary aldehydes were also tested in this catalytic ketyl–olefin coupling. Remarkably, the corresponding coupling products (27–32) were formed in moderate to good yields. Pivalic (27) and adamantyl aldehyde (28) reacted to give the corresponding difluoroalkenes in 58 and 70% yield, respectively. α-Cyclic aldehydes featuring tetrahydrofuran (29), cyclopentyl (30) and cyclohexyl (31) substituents proved equally reactive and allowed for the generation of highly congested protected homoallylic alcohols. Pleasingly, gemfibrozil derivative 32 could also be obtained without interference of the electron-rich aryl ether. Benzaldehydes failed at delivering—under the optimized activation conditions—benzylic α-oxy bromides,^17*c*^ therefore hampering their successful defluorinative ketyl–olefin coupling.

**Scheme 3:**
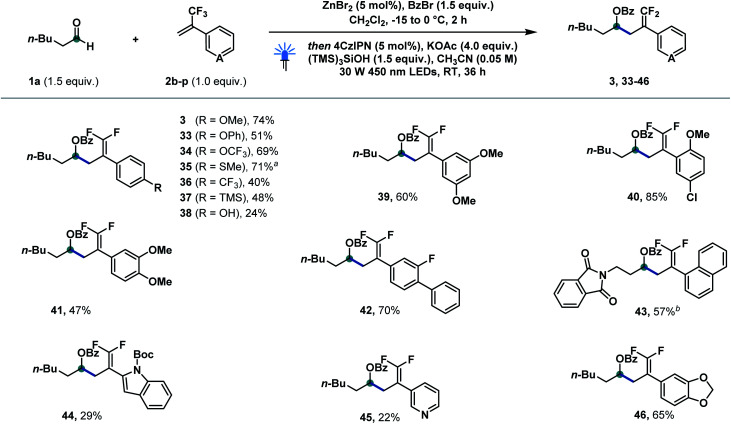
Reaction scope of α-trifluoromethylstyrene derivatives. Reaction conditions: 1a (0.3 mmol, 1.5 equiv.), benzoyl bromide (0.3 mmol, 1.5 equiv.), ZnBr_2_ (0.01 mmol, 5 mol%), CH_2_Cl_2_ (1 M), −15 to 0 °C, 2 h, then 2b–2p (0.2 mmol, 1.0 equiv.), 4CzIPN (0.01 mmol, 5 mol%), KOAc (0.8 mmol, 4.0 equiv.), (TMS)_3_SiOH (0.3 mmol, 1.5 equiv.), MeCN (0.05 M), 30 W 450 nm LEDs, RT, 36 h. ^*a*^ The reaction was performed on a 0.1 mmol scale. ^*b*^ Aldehyde 1n was used instead of 1a. Boc = *tert*-butoxycarbonyl. For experimental details, see the ESI.†

We then explored a variety of olefin derivatives to further investigate this catalytic protocol (Scheme 3). As shown below, a wide range of olefin derivatives could couple with hexanal 1a to generate the corresponding products (3, 33–46) in good to excellent yields. *para*-Substituted trifluoromethyl styrenes bearing methoxyl (3), phenoxyl (33), trifluoromethoxyl (34), thiomethyl (35), chloride (40), trifluoromethyl (36), trimethylsilyl (37), and hydroxyl (38) successfully delivered the corresponding products in moderate to good yields. Polysubstituted systems bearing 3,5-dimethoxy (39), 2-methoxy-4-chloro (40), 3,4-dimethoxy (41) and 3-fluoro-4-phenyl (42) substituents were well tolerated. 2-Naphthyl (43), indole (44), pyridine (45) and 1,3-benzodioxole (46) were successfully incorporated into the final product. α-Trifluoromethyl styrenes featuring electron-withdrawing groups afforded—as reported by Wang^13*a*^ and Molander^11*d*^—substantial amounts of trifluoromethylated side products from competitive protonation of IX (for details see the ESI†).

### Mechanistic studies

Based on previous studies,^17*a–f*^ α-bromo alkyl intermediate IV could be formed *in situ* upon reacting aldehydes and benzoyl bromide with a catalytic amount of zinc bromide. When cyclopropyl-containing alkene 2q was employed under the optimized condition, ring-opening product 47 was formed, suggesting formation of a benzyl radical as a reaction intermediate.^19^ Density functional theory (DFT) calculations at the CAM-B3LYP-D3/def-SVP, CPCM (MeCN)//CAM-B3LYP-D3/def2-TZVPP, and CPCM (MeCN) levels of theory were used to shed light on the following questions: (I) What are the mechanistic intricacies of the α-oxy radical VI formation phase? (II) What is the energetic profile of the reaction?

**Scheme 4:**
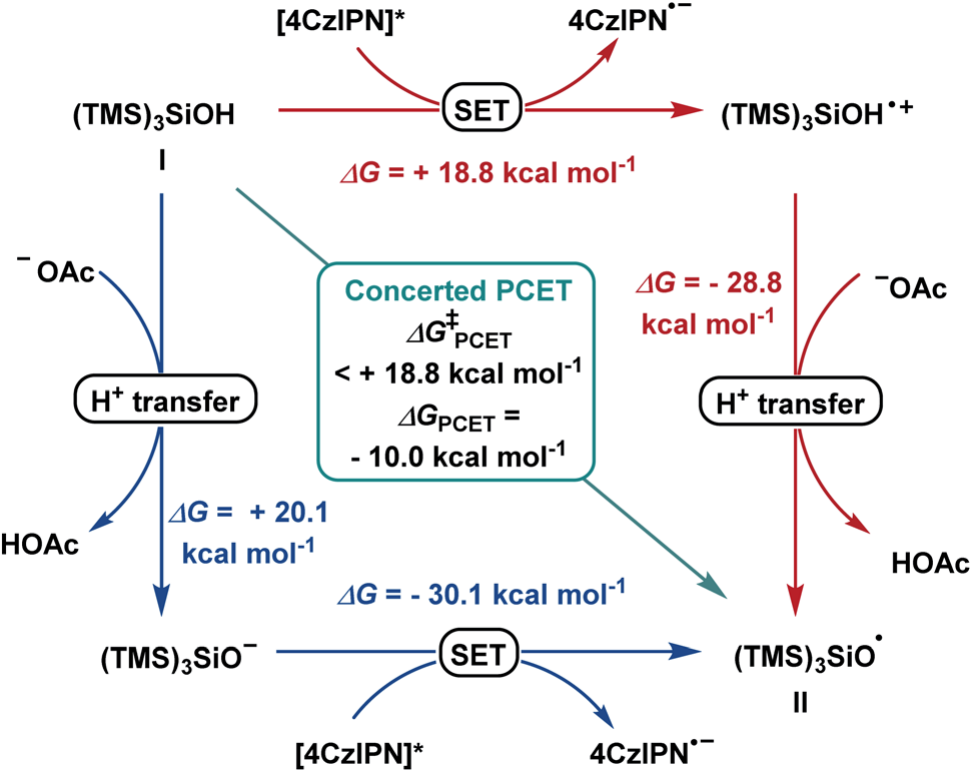
Reaction pathways for stepwise deprotonation/SET (blue), SET/deprotonation (red) and concerted PCET (green).

According to literature reports, we postulated the generation of radical (TMS)_3_SiO˙ (II) from (TMS)_3_SiOH (I) to occur in order to initiate the subsequent reaction steps (Scheme 4). The transfer of one electron and one proton was calculated to be thermodynamically downhill with Δ*G* = −10.0 kcal mol^−1^. Considering a consecutive SET/deprotonation, we found direct oxidation of supersilanol I to be highly endergonic (Δ*G* = +18.8 kcal mol^−1^). The subsequent deprotonation is exergonic (Δ*G* = −28.8 kcal mol^−1^). Considering the opposite stepwise deprotonation/SET process, we found that acetate-mediated deprotonation is endergonic with Δ*G* = +20.1 kcal mol^−1^ and the following oxidation of the deprotonated supersilyl anion *via* SET is exergonic (Δ*G* = −30.1 kcal mol^−1^). Given that, in both stepwise scenarios, the first reaction step is significantly endergonic, a concerted oxidative PCET is deemed likely.

**Scheme 5:**
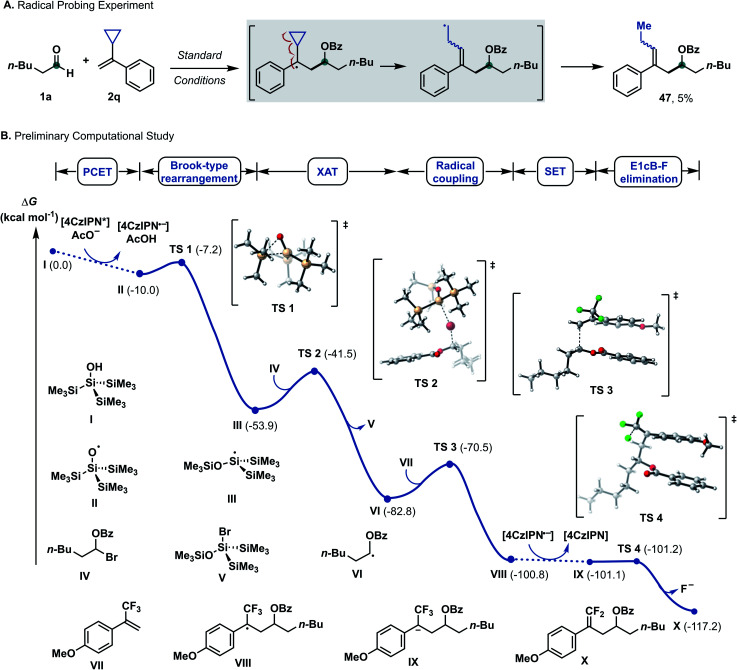
Selected mechanistic experiments and DFT calculations. Density functional theory calculations were performed at the CAM-B3LYP-D3/def-SVP, CPCM (MeCN)//CAM-B3LYP-D3/def2-TZVPP, and CPCM (MeCN) levels of theory. Δ*G* values are in kcal mol^−1^. For experimental details, see the ESI.†

Applying upper limit approximation, we propose the kinetic energy barrier of concerted PCET to be lower than 18.8 kcal mol^−1^ (Δ*G*^‡^ < +18.8 kcal mol^−1^ < +20.1 kcal mol^−1^). Next, the formation of a ketyl-type radical was further investigated (Scheme 5). Brook-type rearrangement to form silyl radical III has a small energy barrier (TS-1, Δ*G*^‡^ = +2.8 kcal mol^−1^) and it is exergonic by 43.9 kcal mol^−1^. Being both kinetically and thermodynamically favoured, Brook-type rearrangement is expected to happen before any bimolecular side reaction. The ensuing halogen atom transfer (XAT) from α-benzoate bromide IV to radical III was found to be thermodynamically favoured (Δ*G* = −28.8 kcal mol^−1^) and has to overcome a kinetic energy barrier of 12.4 kcal mol^−1^. This value agrees with reports by MacMillan and Houk for C–Br abstraction in 2-bromopropane by silyl radical III.^20^ For the addition of the ketyl-type radical to styrene VI, a kinetic energy barrier of +12.3 kcal mol^−1^ was found. The process was computed to be exergonic (Δ*G* = −18.1 kcal mol^−1^ for VI + VII → VIII). From trifluoroalkane radical VIII, single electron transfer from reduced 4CzIPN (*E*_ox_(4CzIPN/4CzIPN˙^−^) = −1.21 V *vs.* SCE)^21^ to give trifluoroalkane anion IX was found to be slightly exergonic (Δ*G* = −0.3 kcal mol^−1^ for VIII → IX). The subsequent E1cB-type fluoride elimination from methoxy substituted anion IX was significantly exergonic (Δ*G* = −16.2 kcal mol^−1^) and barrierless. Hence, this intramolecular process occurs immediately once intermediate IX is formed. Assuming that protonation occurs from supersilanol I, the process was found to be thermodynamically favoured compared to defluorination (Δ*G* = −35.5 kcal mol^−1^). However, given the barrierless E1cB-type process, we assume that the concentration of IX is so low that the bimolecular protonation—which has a first order dependence on the concentration of IX—is substantially suppressed. Considering the full energy profile, the ketyl–olefin coupling product proceeds *via* a sequence of halogen-atom abstraction, ketyl-type radical addition, SET and fluoride elimination. The rate limiting step features either halogen-atom abstraction (Δ*G*^‡^ = +12.4 kcal mol^−1^), radical addition to trifluoroalkene (Δ*G*^‡^ = +12.3 kcal mol^−1^) or PCET (Δ*G*^‡^ < +18.8 kcal mol^−1^).

## Conclusions

In summary, we successfully demonstrated the first example of catalytic defluorinative ketyl–olefin coupling reaction enabled by halogen-atom transfer. This radical-to-polar crossover methodology provides an alternative approach to achieve catalytic ketyl–olefin coupling reaction under visible light conditions, bypassing the need for (super)stoichiometric metal reductants. This newly developed method demonstrates that aliphatic aldehydes could be applied in the halogen-atom transfer induced radical chemistry and that *in situ* formed α-oxy bromides can serve as ketyl-radical surrogates to harness umpolung reactivity. Based on this concept, we expect that this approach will not only be applied in the synthesis of organofluoride compounds but also inspire related coupling reactions and radical carbonyl chemistry.

## Data availability

Experimental and computational data are provided in the ESI.† Raw NMR data (JCAMP and .*mnova* files) for literature unknown compounds and *xyz* coordinates from DFT calculations have been deposited at Zenodo, under the Creative Commons Attribution 4.0 International license: https://doi.org/10.5281/zenodo.6533181.

## Author contributions

H.-M. H., P. B. and F. G. conceptualized the research, H.-M. H., P. B., T. F. and R. L. performed the investigation, and T. F. performed the density functional theory investigation. H.-M. H., P. B. and F. G. wrote the manuscript with contributions from all authors, and F. G. supervised the project.

## Conflicts of interest

There are no conflicts to declare.

## Supplementary Material

SC-013-D2SC02732A-s001
