# Supplementary material for: Catalytic defluorinative ketyl–olefin coupling by halogen-atom transfer
Source: Chem Sci. 2022 Jun 10;13(26):7855–62. doi: 10.1039/d2sc02732a (PMC9258324; doi:10.1039/d2sc02732a)
Supplement: SC-013-D2SC02732A-s001 [file SC-013-D2SC02732A-s001.pdf]

Electronic Supplementary Material (ESI) for Chemical Science.
This journal is © The Royal Society of Chemistry 2022

Catalytic Defluorinative Ketyl-Olefin Coupling by Halogen-Atom Transfer

Peter Bellotti,[a§] Huan-Ming Huang,[a,b§] Teresa Faber,[a] Ranjini Laskar[a] and Frank Glorius[a*]

[a]Organisch-Chemisches Institut, Westfälische Wilhelms-Universität Münster, Corrensstraße 36, 48149 Münster, Germany; [b]School of Physical Science and Technology, ShanghaiTech University, Shanghai 201210, China.

[§] Denotes equal contribution * Corresponding author. Email: glorius@uni-muenster.de

SUPPORTING INFORMATION

Contents

1. General Experimental **2**

1.1 Analytical Techniques 2

1.2 Purification Techniques 3

1.3 Solvents 4

1.4 Light Sources 4

2. Synthesis of Starting Material **6**

2.1. Synthesis of photocatalysts 7

2.2. Synthesis of Aldehydes 7

2.3. Synthesis of Olefins 25

3. Substrate Scope **36**

3.1. Aldehydes Scope 37

3.2. Olefins Scope 58

3.3. Olefins bearing electron-withdrawing groups 69

4. Mechanistic investigation **72**

4.1. Radical probe experiment 72

4.2. Computional study 74

4.2.1 Cartesian coordinates of computed structures 76

5. Condition-based sensitivity screening **93**

6. ^{1}H, ^{13}C, ^{19}F NMR and IR Spectra of Compounds **95**

7. References **203**

1. General Experimental

All reactions were performed in oven-dried glassware under argon, unless otherwise stated. Reaction temperatures are referred to the ones of the heating/cooling media (heating block, cryogenic bath), unless otherwise stated. Reagents were purchased from Sigma-Aldrich, Merck, ACROS Organics, Alfa Aesar, Fluorochem, Combiblocks, TCI and used without further purification. Reactions were stirred using PTFE-coated magnetic stirring bars at ~ 1000 rpm, unless otherwise stated. Low boiling solvents (<110 °C) were removed by rotary evaporation under reduced pressure, heating the solution with a water bath at 40 °C. High boiling solvents (>110 °C) were removed in vacuo (<1 mbar) at room temperature or under mild heating (<50 °C), unless otherwise stated. The identity of literature-known compounds was assessed by comparison of ^{1}H NMR spectra and therefore reported. New compounds were characterized by means of ^{1}H NMR, ^{13}C NMR, ^{19}F NMR (when applicable), HRMS, retention factor on thin layer chromatography, unless otherwise noted. Literature known compounds were characterized by ^{1}H NMR (and ^{13}C NMR, ^{19}F NMR in some cases) and compared with the available analytical data to confirm their identity.

1.1 Analytical Techniques

Thin layer chromatography (TLC) was performed using Merck silica gel 60 F254 aluminum plates and visualization was accomplished with UV light (254 nm) and/or staining with basic $KMnO_4$ solution (4 g of $KMnO_4$, 10 g K_2CO_3, 1 g NaOH in 200 ml of distilled water). GC samples were filtered over a short plug of silica (length: 2-3 cm, diameter: 4 mm) eluting with EtOAc before analysis, if not stated otherwise. GC-MS spectra were recorded on an Agilent Technologies 7890A GC-system (Agilent 5975C VL MSD or an Agilent 5975 MSD) or Agilent Intuvo 9000 (Agilent 5977B MSD), and a HP-5MS column (0.25 mm · 30 m, film: 0.25 μm) and analyzed using MSD ChemStation E.02.02.1431. ^{1}H, ^{13}C and ^{19}F NMR spectra were recorded at room temperature on a Bruker Avance 400 (^{1}H: 400.13 MHz; ^{13}C:

100.62 MHz), Avance Neo 400 (^{1}H: 400.23 MHz; ^{13}C: 100.65 MHz), Varian 500 MHz INOVA (^{1}H: 499.83 MHz; ^{13}C: 125.70 MHz) or Varian Unity plus 600 (^{1}H: 599.31 MHz; ^{13}C: 150.71 MHz) in deuterated solvents (> 99.5% Deuteration) purchased from Sigma-Aldrich ($CDCl_3$, DMSO-*d6*; D_2O, CD_3OD, THF-*d8*, C_6D_6). Chemical shifts (δ) for ^{1}H and ^{13}C NMR spectra are given in parts per million (ppm) relative to tetramethylsilane (TMS) using the residual solvent signals as references for ^{1}H and ^{13}C NMR spectra ($CDCl_3$: δ_H= 7.26 ppm, δ_C= 77.16 ppm, DMSO-*d6*: δ_H = 2.05 ppm, δ_C = 29.84 or 206.26 ppm, CD_3OD: δ_H = 3.31 ppm, δ_C = 49.00 ppm, D_2O: δ_H = 4.79 ppm, δ_C = absolute referencing, acetone-*d6*: δ_H = 2.05 ppm, δ_C = 206.26, THF-*d8*: δ_H = 3.58 or 1.73 ppm; C_6D_6: δ_H= 7.16 ppm, δ_C = 128.06 ppm). ^{19}F NMR spectra were calibrated using absolute referencing system, as suggested by IUPAC.[1] NMR-signals multiplicities that can be analyzed as first order multiplets are reported using the following abbreviations (or combination thereof): s = singlet, d = doublet, t = triplet, q = quartet, p = quintet, h =sextet; hept = heptet; m = multiplet, br = broad signal. All spectra were processed using MestReNova 14 using standard phase and baseline correction automations. ESI accurate mass spectra (HRMS) were recorded on a MicroTof (Bruker Daltronics, Bremen) with loop injection. Mass calibration was performed using sodium formate cluster ions immediately followed by the sample in a "quasi-internal" calibration or alternatively on an LTQ Orbitap LTQ XL (Thermo-Fisher Scientific, Bremen) with nano spray. ATR-IR was measured on a Shimadzu FTIR-8400S. The wave numbers ($\tilde{\upsilon}$) of recorded IR-signals are quoted in cm^{-1}.

1.2 Purification Techniques

Unless otherwise noted, compouns were purified by flash column chromatography, according to Still and co-workers,[2] using ACROS Organics silica (0.035-0.070 mm, 60 Å) and the specified solvent system under 0.3-0.5 bar overpressure. Pentane, dichloromethane and ethyl acetate used for chromatography were purchased of technical grade and preliminarly purified by atmospheric pressure distillation.

1.3 Solvents

Unless otherwise stated, dry (H_2O content < 50 ppm) reaction solvents were used to perform reactions. The following solvents (ACROS ExtraDry solvents with ACROSeal® cap) were purchased from ACROS Organics, stored under 3 or 4Å activated molecular sieves and collected under positive argon pressure: dimethylsulfoxide (DMSO), ethyl acetate (EtOAc), 1,4-dioxane. The following solvents were purchased from ACROS Organics, Sigma-Aldrich and Fischer Scientific (HPLC grade) and purified using a custom SPS with activated aluminacolumns (built by the "Feinmechanische Werkstatt des Organisch-Chemischen Instituts, WWU Münster") and collected under positive argon pressure according to Grubbs procedure:[3] acetonitrile (MeCN), tetrahydrofuran (THF), diethyl ether (Et_2O), *N,N*-dimethylformamide (*N,N*-DMF), hexane, toluene, 1,2-dichloroethane (1,2-DCE) and dichloromethane (DCM).

1.4 Light Sources

Photochemical reactions were performed in a Hepatochem EvoluChem™ PhotoRedOx Box Duo device and irradiated with two EvoluChem™ P303-30-1 LEDs (30 W, λ_{max}= 450 nm). The reaction temperature was measured to be between 25 °C and 30 °C using this setup.

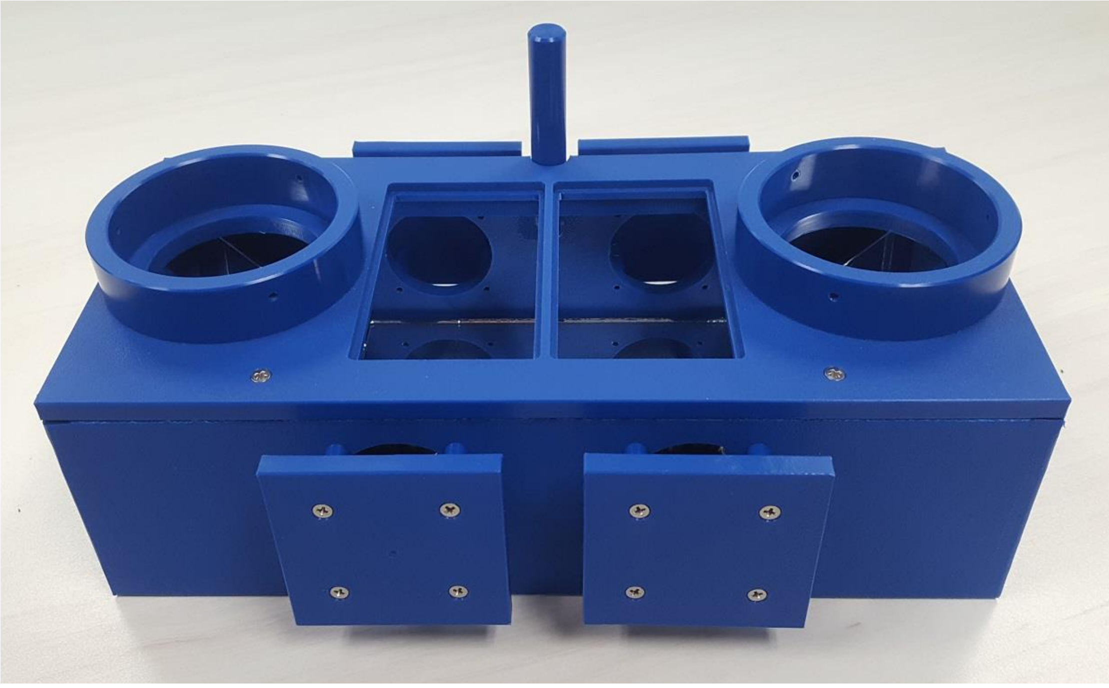

Supplementary Figure 1. Hepatochem EvoluChem™ PhotoRedOx Box Duo (without light sources and vial holders). Courtesy of Hepatochem.

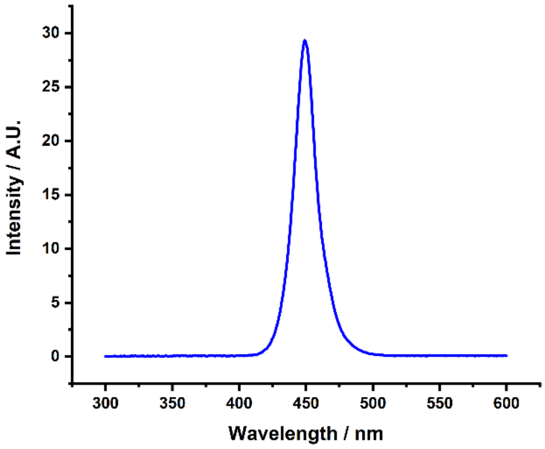

Supplementary Figure 2. Emission spectrum of the light source left - EvoluChem™ P303-30-1 LEDs.

2. Synthesis of Starting Material

Aldehydes **1a-e**, **1s-v**, **1y** were purchased from commercial sources and used as received. Other aldehydes were synthesized according to the procedures detailed below. Trifluoromethyl alkenes were prepared according to the procedures detailed below.

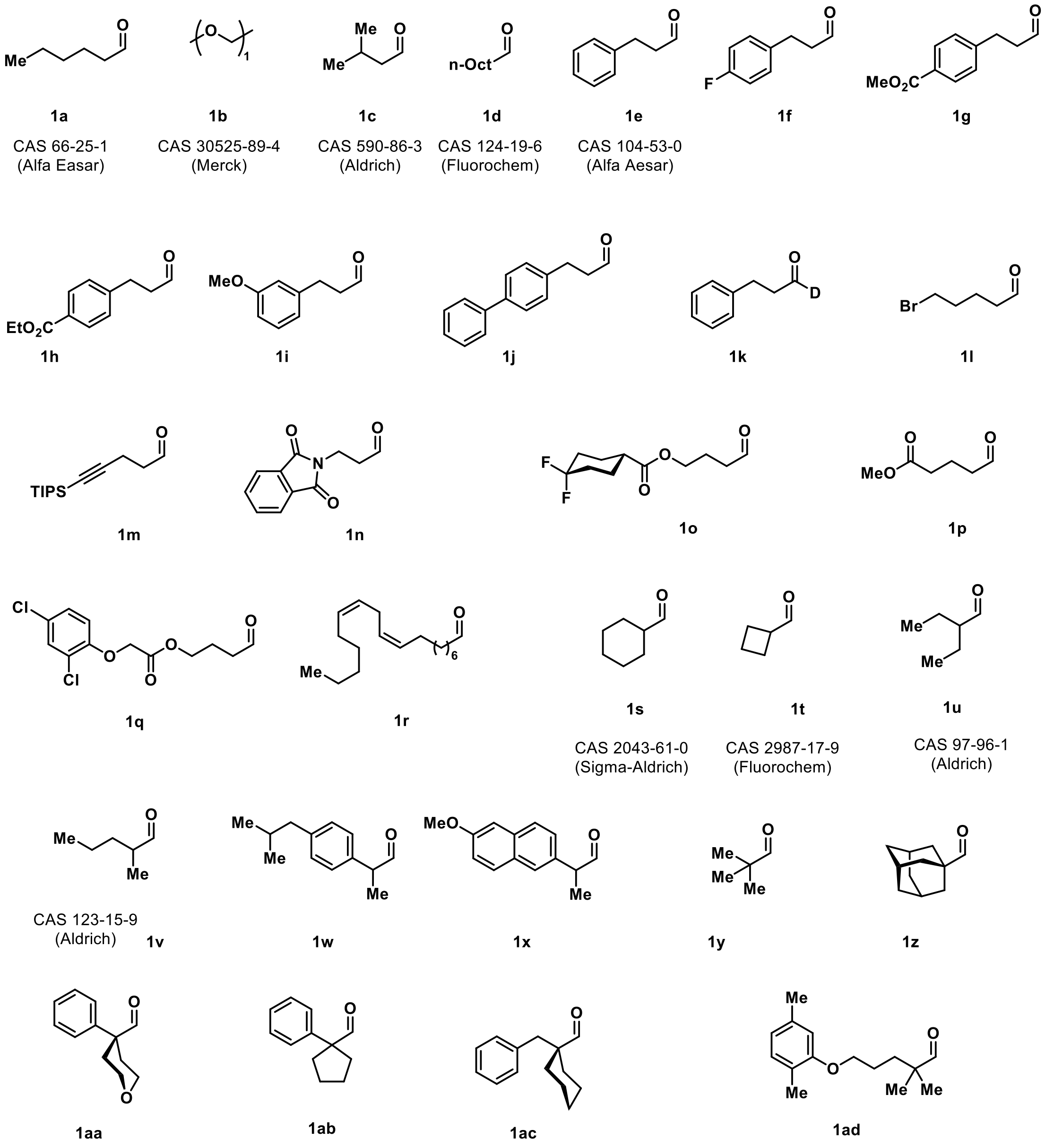

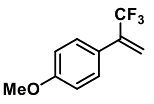
2a
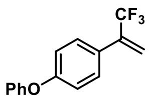
2b
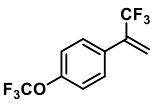
2c
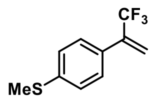
2d
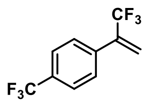
2e

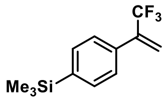
2f
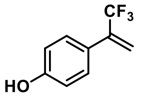
2g
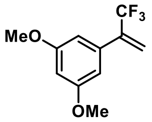
2h
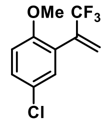
2i
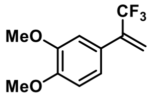
2j

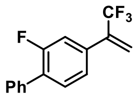
2k
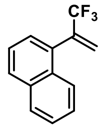
2l
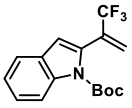
2m
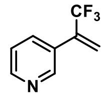
2n
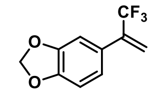
2o

2.1. Synthesis of photocatalysts

PC-1,[4] **PC-2**[4] and **PC-3**[5] were synthesized according to the referenced literature procedures.

2.2. Synthesis of Aldehydes

General procedure 1 for the synthesis of aldehydes (GP1)

The procedure was adapted from Shiner *et al.*[6] An oven-dried Schlenk flask was equipped with a PTFE-coated stirring bar and pyridinium chlorochromate (1.50 equiv.) was charged. The vessel was evacuated and backfilled with argon, then dry CH_2Cl_2 (0.2 M) was added. Upon vigorous stirring, the appropriate alcohol (1.0 equiv.) was added dropwise/portionwise and the reaction mixture was stirred for three hours until all starting material was consumed (monitored by GC-MS or TLC). The reaction mixture was filtered over a silica/Celite® pad and the flask was washed three times with additional CH_2Cl_2 (10 mL each time). The crude reaction mixture was concentrated *in vacuo* and purified by column chromatography on silica gel (pentane/Et_2O mixtures, as indicated in the individual entries) to afford the corresponding aldehyde.

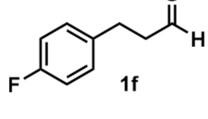
1f

3-(4-Fluorophenyl)propanal (1f). In an oven-dried Schlenk tube equipped with a PTFE-coated stirring bar, $NaHCO_3$ (5.25 g, 62.5 mmol, 2.50 equiv.), TBAB (4.03 g, 12.5 mmol, 0.5 equiv.) and $Pd(OAc)_2$ (112 mg, 0.1 mmol, 2 mol%) were charged, then the vessel was evacuated-back filled with argon three times. Dry DMF (21 ml), followed by 4-iodofluorobenzene (2.88 ml, 25.0 mmol, 1.00 equiv.) and allyl alcohol (2.55 ml, 37.5 mmol, 1.50 equiv.) were added, then the reaction was stirred at 80°C for 3 hours. The reaction was filtered through a short pad of Celite®, rinsing with EtOAc. The organic layer was dried in vacuoremoving most of the DMF, then the residue was taken-up with EtOAc and washed twice with water, then the organic layer was dried over $MgSO_4$ and the solvent removed *in vacuo.* The residue was purified by flash column chromatography (pentane/EtOAc = 15/1) affording **1f** (76 g, 18.2 mmol, 73%) as a yellow liquid. **^{1}H NMR** (300 MHz, $CDCl_3$): δ ppm 9.82 (t, *J* = 1.3 Hz, 1H), 7.19 – 7.10 (m, 2H), 7.03 – 6.92 (m, 2H), 2.93 (t, *J* = 7.4 Hz, 2H), 2.77 (td, *J* = 7.2, 1.2 Hz, 2H). The experimental data are in agreement with the literature report.[7]

1 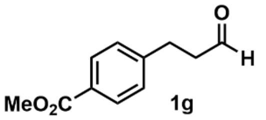

1g

Methyl 4-(3-oxopropyl)benzoate (1g). In an oven-dried Schlenk tube equipped with a PTFE-coated stirring bar, $NaHCO_3$ (1.68 g, 20.0 mmol, 2.00 equiv.), TBAB (2.40 g, 7.5 mmol, 0.5 equiv.) and $Pd(OAc)_2$ (67.2 mg, 0.3 mmol, 2 mol%) were charged, then the vessel was evacuated-back filled with argon three times. Dry DMF (12.5 ml), followed by methyl 4-iodobenzoate (3.93 g, 15.0 mmol, 1.00 equiv.) and allyl alcohol (1.53 ml, 22.5 mmol, 1.50 equiv.) were added, then the reaction was stirred at 80°C for 3 hours. The reaction was filtered through a short pad of Celite®, rinsing with EtOAc. The organic layer was dried *in vacuo* to remove most of the DMF, then the residue was taken-up with EtOAc and washed twice with water, then the organic layer was dried over $MgSO_4$ and the solvent removed *in vacuo*. The residue was purified by flash column chromatography (pentane/EtOAc = 4/1 → 3/1) affording **1g** (1.35 g, 7.03 mmol, 47%) as a yellow oil. **^{1}H NMR** (400

MHz, $CDCl_3$) δ 9.82 (t, *J* = 1.1 Hz, 1H), 7.96 (d, *J* = 8.4 Hz, 2H), 7.28 – 7.24 (m, 2H), 3.90 (s, 3H), 3.01 (t, *J* = 7.5 Hz, 3H), 2.82 (t, *J* = 7.6 Hz, 2H). The experimental data are in agreement with the literature report.[8]

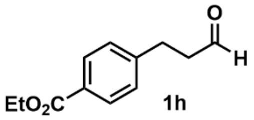

Ethyl 4-(3-oxopropyl)benzoate (1h). In an oven-dried Schlenk tube equipped with a PTFE-coated stirring bar, $NaHCO_3$ (1.51 g, 18.0 mmol, 1.80 equiv.), TBAB (3.22 g, 10.00 mmol, 1.00 equiv.) and $Pd(OAc)_2$ (45 mg, 0.1 mmol, 1 mol%) were charged, then the vessel was evacuated-back filled with argon three times. Dry DMF (5 ml), followed by methyl 4-iodobenzoate (1.63 ml, 10.0 mmol, 1.00 equiv.) and allyl alcohol (1.02 ml, 15.0 mmol, 1.50 equiv.) were added, then the reaction was stirred at 50°C overnight. The reaction was filtered through a short pad of Celite®, rinsing with EtOAc. The organic layer was dried *in vacuo* to remove most of the DMF, then the residue was taken-up with EtOAc and washed twice with water, then the organic layer was dried over $MgSO_4$ and the solvent removed *in vacuo*. The residue was purified by flash column chromatography (pentane/EtOAc = 7/1) affording **1h** (1.67 g, 8.08 mmol, 81%) as a light brown oil. **^{1}H NMR** (400 MHz, $CDCl_3$): δ ppm 9.83 (t, *J* = 1.2 Hz, 1H), 7.97 (d, *J* = 8.3 Hz, 2H), 7.30 – 7.23 (m, 2H), 4.36 (q, *J* = 7.1 Hz, 2H), 3.01 (t, *J* = 7.5 Hz, 2H), 2.86 – 2.77 (m, 2H), 1.39 (t, *J* = 7.1 Hz, 3H). The experimental data are in agreement with the literature report.[7]

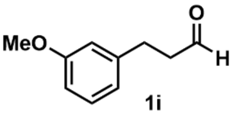

3-(3-Methoxyphenyl)propanal (1i). In an oven-dried Schlenk tube equipped with a PTFE-coated stirring bar, $NaHCO_3$ (1.51 g, 18.0 mmol, 1.80 equiv.), TBAB (3.22 g, 10.00 mmol, 1.00 equiv.) and $Pd(OAc)_2$ (45 mg, 0.1 mmol, 1 mol%) were charged, then the vessel was evacuated-back filled with argon three times. Dry DMF (5 ml), followed by methyl 4-iodobenzoate (1.19 ml, 10.0 mmol, 1.00 equiv.) and allyl alcohol (1.02 ml, 15.0 mmol, 1.50 equiv.) were added, then the reaction was stirred at 50°C overnight. The reaction was filtered through a short pad of Celite®, rinsing with EtOAc. The organic layer was dried *in vacuo* to remove most

of the DMF, then the residue was taken-up with EtOAc and washed twice with water, then the organic layer was dried over $MgSO_4$ and the solvent removed *in vacuo*. The residue was purified by flash column chromatography (pentane/EtOAc = 13/1) affording **1i** (968 mg, 5.90 mmol, 59%) as a yellow oil. **^{1}H NMR** (400 MHz, CDCl3): δ ppm 9.83 (t, *J* = 1.4 Hz, 1H), 7.25 – 7.15 (m, 1H), 6.82 – 6.71 (m, 3H), 3.80 (s, 3H), 2.94 (t, *J* = 7.5 Hz, 2H), 2.84 – 2.73 (m, 2H). The experimental data are in agreement with the literature report.[7]

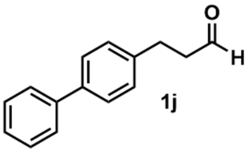

1j

3-([1,1'-Biphenyl]-4-yl)propanal (1j). In an oven-dried Schlenk tube equipped with a PTFE-coated stirring bar, $NaHCO_3$ (756 mg, 9.0 mmol, 1.80 equiv.), TBAB (1.61 g, 5.00 mmol, 1.00 equiv.) and $Pd(OAc)_2$ (13 mg, 0.05 mmol, 1 mol%) were charged, then the vessel was evacuated-back filled with argon three times. Dry DMF (5 ml), followed by 4-iodobipheyl (1.40 g, 5.00 mmol, 1.00 equiv.) and allyl alcohol (510 µl, 7.50 mmol, 1.50 equiv.) were added, then the reaction was stirred at 50°C overnight. The reaction was filtered through a short pad of Celite®, rinsing with EtOAc. The organic layer was dried in vacuoremoving most of the DMF, then the residue was taken-up with EtOAc and washed twice with water, then the organic layer was dried over $MgSO_4$ and the solvent removed *in vacuo*. The residue was purified by flash column chromatography (pentane/EtOAc = 16/1 → 14/1) affording **1j** (814 mg, 3.87 mmol, 77%) as a greyish waxy solid. **^{1}H NMR** (300 MHz, $CDCl_3$): δ ppm 9.86 (t, J = 1.4 Hz, 1H), 7.62– 7.56 (m, 2H), 7.56 – 7.50 (m, 2H), 7.48 – 7.40 (m, 2H), 7.38 – 7.31 (m, 1H), 7.31 – 7.24 (m, 2H), 3.01 (t, J = 7.5 Hz, 2H), 2.88 – 2.79 (m, 2H). The experimental data are in agreement with the literature report.[7]

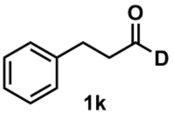

1k

3-Phenylpropanal-1-*d* (1k). The compound was synthesized according to the following two-step procedure.

Step 1. In an oven-dried Schlenk tube equipped with a PTFE-coated stirring bar, ethyl 3-phenylpropanoate (1.06 ml, 6.00 mmol, 1.0 equiv.) was charged under argon and dissolved in dry Et_2O (25 ml) at 0 °C. $LiAlD_4$ (252 mg, 6.00 mmol, 1.00 equiv.) was added in one portion and the reaction was stirred at 0 °C for 15 minutes, the warmed at room temperature and stirred for 30 minutes. The reaction was cooled to 0 °C, diluted with Et_2O (30 ml) and consecutively quenched with water (0.25 ml), NaOH (2M, 0.25 ml) and water (0.75 ml), then was warmed at room temperature and $MgSO_4$ was added. The slurry was filtered over a short pad of Celite®, rinsing thoroughly with Et_2O. The volatiles were removed *in vacuo*, affording the intermediate **SI-1k** (830 mg, 6.00 mmol, > 99% yield, d_1 > 95%) as a colourless oil. **^{1}H NMR** (400 MHz, $CDCl_3$) δ 7.32 – 7.26 (m, 2H), 7.22 – 7.16 (m, 3H), 2.76 – 2.67 (m, 2H), 1.89 (t, *J* = 7.7 Hz, 2H). **GC-MS** (EI, 1.9-normal) @ 3.02 min, *m/z* = 138 (M^+).[9]

Step 2. In a round-bottom flask equipped with a PTFE-coated stirring bar, the intermediate **SI-1k** (830 mg, 6.00 mmol, 1.00 equiv.) dissolved in CH_2Cl_2 (40 ml), then Dess-Martin periodinane (3.18 g, 7.50 mmol, 1.25 equiv.) was added and the reaction was stirred at room temperature overnight, then diluted with pentane (15 ml) and filtered over a short pad of Celite®, rinsing with little CH_2Cl_2. The organic layer was washed with saturated $NaHCO_3$, then dried over $MgSO_4$ and the solvent was removed *in vacuo*. The crude residue was purified by flash column chromatography on silica (pentane/EtOAc = 20/1), affording **1k** (548 mg, 4.05 mmol, 68 %, d_1 > 99%) as a colourless liquid. **^{1}H NMR** (400 MHz, $CDCl_3$) δ 7.33 – 7.27 (m, 2H), 7.24 – 7.17 (m, 3H), 2.97 (t, *J* = 7.5 Hz, 2H), 2.83 – 2.74 (m, 2H). **^{13}C{^{1}H} NMR** (101 MHz, $CDCl_3$) δ 140.5, 128.8, 128.4, 126.5, 45.2 (t, *J* = 3.8 Hz), 28.2. The experimental data are in agreement with the literature report.[10]

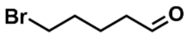

1l

5-bromopentanal (1l). The compound was prepared according to the following two step procedure.

Step 1. The reaction was adapted from Chong *et al.*[11] In a two-necked round-bottom flask equipped with a PTFE-coated stirring bar and an air condenser, 1,5-Pentandiol (13.95 mL, 135 mmol, 1.0 equiv.), toluene (400 mL) and aqueous HBr (48%, 17.82 mL) were added, then the reaction mixture was heated at reflux under vigorous stirring for 36 hours. Upon completion (determined by GC-MS and TLC), the reaction mixture was cooled to room temperature, the phases were separated and the organic layer was diluted with Et_2O (100 mL). The combined organic phases were consecutively washed with 1 M NaOH, brine, 3 M phosphate buffer with pH = 7 (50 mL each), dried over $MgSO_4$, filtered and concentrated *in vacuo*. Column chromatography of the crude material on silica gel (pentane/EtOAc = 1/0 → 1/1) afforded the intermediate alcohol compound (8.75 g, 52.4 mmol, 39%) as a colorless oil. $\mathbf{R_f}$ (pentane/EtOAc = 4/1) 0.50. **^{1}H NMR** (400 MHz, $CDCl_3$) δ 3.66 (t, *J* = 6.3 Hz, 2H), 3.42 (t, *J* = 6.8 Hz, 2H), 1.89 (m, 2H), 1.66 – 1.49 (m, 4H), 1.46 (s, 1H). **^{13}C{^{1}H} NMR** (101 MHz, $CDCl_3$) *δ* 62.8, 33.8, 32.6, 31.9, 24.6. The experimental data are in agreement with the literature report.[11]

Step 2. The compound was synthesized according to **GP1** with 5-bromopentan-1-ol (1.67 g, 10.0 mmol, 1.0 equiv.), PCC (3.24 g, 15.0 mmol, 1.5 equiv.) in CH_2Cl_2 (50 mL). Column chromatography on silica gel (pentane/Et_2O = 93/7 → 85/15) afforded **1l** (1.16 g, 7.03 mmol, 70%) as a colorless oil. $\mathbf{R_f}$ (pentane/Et_2O = 9/1) 0.30. **^{1}H NMR** (400 MHz, $CDCl_3$) *δ* 9.77 (t, *J* = 1.5 Hz, 1H), 3.41 (t, *J* = 6.5 Hz, 2H), 2.49 (td, *J* = 7.1, 1.5 Hz, 2H), 1.94 – 1.85 (m, 2H), 1.84 – 1.74 (m, 2H). **^{13}C{^{1}H} NMR** (101 MHz, $CDCl_3$) *δ* 201.9, 43.0, 33.2, 32.0, 20.8. The experimental data are in agreement with the literature report.[12]

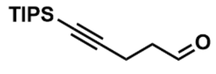

1m

5-(triisopropylsilyl)pent-4-ynal (1m). The compound was synthesized according to the following two step procedure.

Step 1. The reaction was adapted from the procedure from Layton *et al.*[13] A Schlenk flask equipped with a PTFE-coated stiring bar was charged with pent-4-yn-1-ol (1.86 mL, 20 mmol, 1.0 equiv.), then THF (40 mL) was added, followed by

EtMgBr (3 M in Et_2O, 14 mL, 42 mmol, 2.1 equiv.) at room temperature, then the reaction mixture was stirred under reflux for 12 hours. Upon cooling to room temperature, triisopropylsilyl chloride (4.28 mL, 20 mmol, 1.0 equiv.) in THF (20 mL) was added over 15 minutes and the reaction mixture was stirred under reflux for additional 6 hours. After cooling to room temperature, the reaction mixture was poured into aqueous HCl (10%, 50 mL) and the mixture was extracted three times with Et_2O (50 mL each time). The combined organic layers were dried over $MgSO_4$, filtered and concentrated *in vacuo* to afford the corresponding alcohol **SI-1m**, which was used without further purification.

Step 2. In a round-bottom flask equipped with a PTFE-coated stirring bar, Dess-Martin periodane (8.89 g, 21.0 mmol, 1.05 equiv.) was dissolved in CH_2Cl_2 (70 mL), then the crude alcohol **SI-1m** in CH_2Cl_2 (2 mL) and deionized H_2O (0.37 mL, 21.0 mmol, 1.05 equiv.) were consecutively added. The reaction mixture was stirred at room temperature for 30 minutes and then poured into a 1:1 mixture of saturated aqueous $Na_2S_2O_3$ and $NaHCO_3$ (15 mL each), then extracted three times with Et_2O (20 mL each time). The combined organic layers were washed with saturated aqueous $NaHCO_3$ and brine (10 mL each time), dried over $MgSO_4$, filtered and concentrated *in vacuo*. The crude product was purified by column chromatography on silica gel (pentane/Et_2O = 24/1 → 19/1) to afford **1m** (0.95 g, 3.99 mmol, 20% over 2 steps) as a colorless oil. **R_f** (pentane/Et_2O = 19/1) 0.40. **^{1}H NMR** (400 MHz, $CDCl_3$) δ 9.81 (t, J = 1.4 Hz, 1H), 2.67 (m, 2H), 2.62 – 2.54 (m, 2H), 1.08 – 1.00 (m, 21H). **^{13}C{^{1}H} NMR** (101 MHz, $CDCl_3$) δ 200.8, 106.5, 82.0, 43.0, 18.7, 13.5, 11.3. The experimental data are in agreement with the literature report.[13]

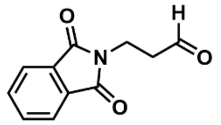

1n

3-(1,3-dioxoisoindolin-2-yl)propanal (1n). The title compound was prepared according to the following two-step procedure.

Step 1. In a round-bottom flask equipped with a PTFE-coated stirring bar, phthalic anhydride (9.85 g, 66.5 mmol, 1.0 equiv.) was dissolved in toluene (50 ml), then 3-aminopropanol (5.1 ml, 66.5 mmol, 1.0 equiv.) was added and the reaction was heated at 125 °C overnight. The reaction was cooled to room temperature, then the solvent was removed under rotary evaporation, affording substantially clean **SI-1n** (12 g) as a white solid. ^{1}**H NMR** (400 MHz, $CDCl_3$) δ 7.86 (dd, *J* = 5.5, 3.1 Hz, 2H), 7.73 (dd, *J* = 5.5, 3.0 Hz, 2H), 3.86 (t, *J* = 6.4 Hz, 2H), 3.62 (t, *J* = 5.8 Hz, 2H), 1.88 (p, *J* = 6.0 Hz, 2H). The experimental data are in agreement with the literature report.[14]

Step 2. In an oven-dried Schlenk tube equipped with a PTFE-coated stirring bar, oxalyl chloride (2.07 ml, 24.4 mmol, 1.25 equiv.) was dissolved in anhydrous CH_2Cl_2 (100 ml) under argon, then cooled to -78 °C. Anhydrous DMSO (3.7 ml, 48.8 mmol, 2.5 equiv.) was added dropwise, then the reaction was stirred at the same temperature for 30 minutes. At the same temperature, alcohol **SI-1n** (4.0 g, 19.5 mmol, 1.0 equiv.) in anhydrous CH_2Cl_2 (4 ml) was added dropwise and the reaction was stirred for 30 minutes, then anhydrous Et_3N (10 ml, 78 mmol, 4.0 equiv.) was added and the reaction was warmed to 0 °C. After 30 minutes, the reaction was carefully quenched with deionized water (40 ml), the layers were separated and the aqueous layer was extracted twice with CH_2Cl_2 (30 ml each time). The combined organic layers were washed once with brine (30 ml), then were dried over $MgSO_4$ and the solvent was removed *in vacuo*. The residue was purified by flash column chromatography on silica (pentane/EtOAc = 1/1), affording **1n** (3.58 g, 16.2 mmol, 88%) as a white solid. ^{1}**H NMR** (400 MHz, $CDCl_3$) δ 9.82 (t, *J* = 1.1 Hz, 1H), 7.85 (dd, *J* = 5.5, 3.1 Hz, 2H), 7.73 (dd, *J* = 5.5, 3.1 Hz, 2H), 4.04 (t, *J* = 7.0 Hz, 2H), 2.88 (td, *J* = 7.0, 1.4 Hz, 2H). The experimental data are in agreement with the literature report.[15]

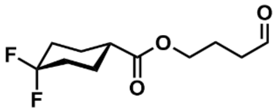

1o

4-Oxobutyl 4,4-difluorocyclohexane-1-carboxylate (1o). The title compound was synthesized using the following two-step procedure.

<u>Step 1.</u> In an oven-dried round-bottom flask equipped with a PTFE-coated stirring bar, 4,4-difluorocyclohexylacetic acid (821 mg, 5.00 mmol, 1.00 equiv.) was dissolved in CH_2Cl_2 (30 ml), then one drop of DMF followed by oxalyl chloride (515 µl, 6.00 mmol, 1.20 equiv.) were added. The reaction was stirred until gas evolution ceased (90 minutes), then the volatiles were removed *in vacuo*. The residue was dissolved in CH_2Cl_2 (15 ml) and added dropwise to a well-stirred solution of 1,4-butandiol (1.33 ml, 15.0 mmol, 3.00 equiv.) and Et_3N (2.70 ml, 20.0 mmol, 4.00 equiv.) in CH_2Cl_2 (15 ml). The reaction was stirred at room temperature for 90 minutes, then quenched with HCl 1M (40 ml). The layers were separated, then the aqueous layer was extracted twice with CH_2Cl_2 (30 ml each time) and the combined organic layers were washed twice with water (30 ml each time) and once with brine (30 ml). The organic extracts were dried over $MgSO_4$, then the solvent was removed *in vacuo* and the residue purified by flash column chromatography on silica (pentane/acetone = 5/1 → 4/1), affording the intermediate **SI-1o** (809 mg, 3.42 mmol, 68%) as a colourless thick oil. **R*f*** (pentane/acetone = 4/1): 0.65. **^{1}H NMR** (400 MHz, $CDCl_3$) δ 4.13 (t, *J* = 6.4 Hz, 2H), 3.68 (t, *J* = 6.3 Hz, 2H), 2.45 – 2.35 (m, 1H), 2.14 – 2.03 (m, 2H), 2.03 – 1.93 (m, 2H), 1.90 – 1.79 (m, 3H), 1.78 – 1.68 (m, 3H), 1.68 – 1.56 (m, 2H). **^{13}C{^{1}H} NMR** (101 MHz, $CDCl_3$) δ 174.5 – 174.4 (m), 122.8 (t, *J* = 240.9 Hz), 64.6, 62.5, 40.8 (t, *J* = 1.0 Hz), 32.7 (t, *J* = 24.6 Hz), 29.2, 25.3 – 25.1 (m). **^{19}F{^{1}H} NMR** (377 MHz, $CDCl_3$) δ -94.27 (br d, *J* = 237.0 Hz), -99.74 (br d, *J* = 237.8 Hz). **HRMS** (ESI$^+$): calcd. for $C_{11}H_{18}O_3F_2Na$ (M+Na$^+$): 259.1116. Found: 259.1114.

<u>Step 2.</u> In an oven-dried round-bottom flask equipped with a PTFE-coated stirring bar, the intermediate **SI-1o** (793 mg, 3.35 mmol, 1.00 equiv.) was dissolved in CH_2Cl_2 (5 ml), then TEMPO (53 mg, 0.34 mmol, 10 mol%) and $PhI(OAc)_2$ (1.19 g, 3.69 mmol, 1.10 equiv.) were added. The reaction was stirred at room temperature for 16 hours, then quenched with saturated $Na_2S_2O_3$ (30 ml). The layers were separated and the aqueous layer was extracted twice with CH_2Cl_2 (20

ml each time). The combined organic layers were washed once with saturated $NaHCO_3$ (30 ml), then dried over $MgSO_4$ and the solvent was removed *in vacuo.* The residue was purified by flash column chromatography on silica (pentane/EtOAc = 3/1), affording **1o** (800 mg, 2.86 mmol, 85%) as a colourless thick oil. **R*f*** (pentane/EtOAc = 9/2): 0.50. **^{1}H NMR** (599 MHz, $CDCl_3$) δ 9.79 (s, 1H), 4.12 (t, *J* = 6.5 Hz, 2H), 2.53 (t, *J* = 7.2 Hz, 2H), 2.44 – 2.34 (m, 1H), 2.13 – 2.03 (m, 2H), 2.01 – 1.94 (m, 4H), 1.87 – 1.70 (m, 8H). **^{13}C{^{1}H} NMR** (151 MHz, $CDCl_3$) δ 201.1, 174.2, 122.7 (t, *J* = 241.0 Hz), 63.8, 40.7, 40.6, 32.7 (t, *J* = 24.5 Hz), 25.3 – 25.1 (m), 21.4. **^{19}F NMR** (564 MHz, $CDCl_3$) δ -94.26 (br d, *J* = 236.3 Hz), -99.86 (br d, *J* = 235.3 Hz). **HRMS** (ESI$^+$): calcd. for $C_{11}H_{16}O_3F_2Na$ (M+Na$^+$): 257.0960. Found: 257.0958.

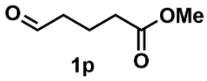

1p

Methyl 4-formylbutanoate (1p). In a round-bottom flask equipped with a PTFE-coated stirring bar, δ-valerolactone (1.0 g, 10.0 mmol, 1.00 equiv.) was dissolved in methanol (10 ml). Triethylamine (0.46 ml, 3.30 mmol, 0.33 equiv.) was added added dropwise, then the solution was stirred overnight at room temperature, then the volatiles were removed *in vacuo* to afford the crude alcohol. In the same flask, dry CH_2Cl_2 (0.2 M) was added, followed by pyridinium chlorochromate (3.24 g, 15.0 mmol, 1.5 equiv.). The reaction mixture was stirred for three hours until all starting material was consumed (monitored by GC-MS or TLC). The reaction mixture was filtered over a Celite® pad and the flask was washed three times with additional CH_2Cl_2 (10 mL each time). The crude reaction mixture was concentrated *in vacuo* and purified by column chromatography on silica gel (pentane/Et_2O = 9/1) afforded **1p** (729 mg, 5.60 mmol, 56% over two steps) as a colorless oil. **^{1}H NMR** (400 MHz, $CDCl_3$) δ: 9.75 (t, *J* = 1.3 Hz, 1H), 3.65 (s, 3 H), 2.50 (td, *J* = 7.1, 1.3 Hz, 2H), 2.35 (t, *J* = 7.1 Hz, 2H), 1.93 (pent, 3H). The experimental data are in agreement with the literature report.[16]

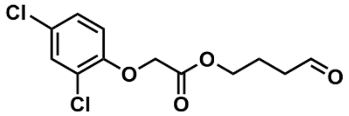

1q

4-Oxobutyl 2-(2,4-dichlorophenoxy)acetate (1q). The title compound was synthesized using the following two-step procedure.

Step 1. In an oven-dried round-bottom flask equipped with a PTFE-coated stirring bar, 2,4-D (1.11 g, 5.00 mmol, 1.00 equiv.) was dissolved in CH_2Cl_2 (30 ml), then one drop of DMF followed by oxalyl chloride (515 µl, 6.00 mmol, 1.20 equiv.) were added. The reaction was stirred until gas evolution ceased (90 minutes), then the volatiles were removed *in vacuo*. The residue was dissolved in CH_2Cl_2 (15 ml) and added dropwise to a well-stirred solution of 1,4-butandiol (1.33 ml, 15.0 mmol, 3.00 equiv.) and Et_3N (2.70 ml, 20.0 mmol, 4.00 equiv.) in CH_2Cl_2 (15 ml). The reaction was stirred at room temperature for 90 minutes, then quenched with HCl 1M (40 ml). The layers were separated, then the aqueous layer was extracted twice with CH_2Cl_2 (30 ml each time) and the combined organic layers were washed twice with water (30 ml each time) and once with brine (30 ml). The organic extracts were dried over $MgSO_4$, then the solvent was removed *in vacuo* and the residue purified by flash column chromatography on silica (pentane/acetone = 4/1 → 3/1), affording the intermediate alcohol **SI-1q** (1.01 g, 3.44 mmol, 69%) as a colourless gum. **R*f*** (pentane/acetone = 4/1): 0.40. **^{1}H NMR** (400 MHz, $CDCl_3$) δ 7.39 (d, *J* = 2.6 Hz, 1H), 7.16 (dd, *J* = 8.8, 2.5 Hz, 1H), 6.78 (d, *J* = 8.8 Hz, 1H), 4.69 (s, 2H), 4.24 (t, *J* = 6.5 Hz, 2H), 3.66 (t, *J* = 6.3 Hz, 2H), 1.80 – 1.70 (m, 2H), 1.63 – 1.51 (m, 3H). **^{13}C{^{1}H} NMR** (101 MHz, $CDCl_3$) δ 168.3, 152.5, 130.5, 127.7, 127.3, 124.4, 114.9, 66.6, 65.5, 62.3, 29.0, 25.1. **HRMS** (ESI$^+$): calcd. for $C_{12}H_{14}O_4{}^{35}Cl_2Na$ (M+Na$^+$): 315.0161. Found: 315.0161.

Step 2. In an oven-dried round-bottom flask equipped with a PTFE-coated stirring bar, the intermediate alcohol **SI-1q** (992 mg, 3.38 mmol, 1.00 equiv.) was dissolved in CH_2Cl_2 (5 ml), then TEMPO (53 mg, 0.34 mmol, 10 mol%) and $PhI(OAc)_2$ (1.20 g, 3.72 mmol, 1.10 equiv.) were added. The reaction was stirred at room temperature for 16 hours, then quenched with saturated $Na_2S_2O_3$ (30 ml).

The layers were separated and the aqueous layer was extracted twice with CH_2Cl_2 (20 ml each time). The combined organic layers were washed once with saturated $NaHCO_3$ (30 ml), then dried over $MgSO_4$ and the solvent was removed *in vacuo.* The residue was purified by flash column chromatography on silica (pentane/EtOAc = 9/2 → 3/1), affording **1q** (852 mg, 2.92 mmol, 87%) as a colourless thick oil. **R*f*** (pentane/EtOAc = 9/2): 0.40. **^{1}H NMR** (599 MHz, $CDCl_3$) δ 9.77 (s, 1H), 7.40 (s, 1H), 7.17 (d, *J* = 8.8 Hz, 1H), 6.79 (d, *J* = 8.8 Hz, 1H), 4.68 (s, 2H), 4.24 (t, *J* = 6.5 Hz, 2H), 2.52 (t, *J* = 7.1 Hz, 2H), 2.00 (p, *J* = 6.3 Hz, 2H). **^{13}C{^{1}H} NMR** (151 MHz, $CDCl_3$) δ 200.8, 168.1, 152.5, 130.5, 127.7, 127.4, 124.5, 114.9, 66.5, 64.6, 40.4, 21.3. **HRMS** (ESI$^+$): calcd. for $C_{12}H_{12}O_4{}^{35}Cl_2Na$ (M+Na$^+$): 313.0005. Found: 313.0002.

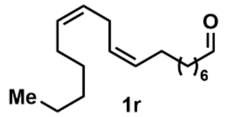

1r

(9*Z*,12*Z*)-Octadeca-9,12-dienal (1r). In an oven-dried Schlenk tube equipped with a PTFE-coated stirring bar, linoleic acid (1.55 ml, 5.00 mmol, 1.00 equiv.) was charged under argon and dissolved in dry THF (20 ml) at 0 °C. $LiAlH_4$ (285 mg, 7.50 mmol, 1.50 equiv.) was added in one portion and the reaction was stirred at 0 °C for 15 minutes, the warmed at room temperature and stirred overnight. The reaction was cooled to 0 °C, diluted with Et_2O (30 ml) and consecutively quenched with water (0.25 ml), NaOH (2M, 0.25 ml) and water (0.75 ml), then was warmed at room temperature and $MgSO_4$ was added. The slurry was filtered over a short pad of Celite®, rinsing thoroughly with Et_2O. The volatiles were removed *in vacuo*, affording the intermediate alcohol **SI-1r** as a faint yellow gum, which was used for the next step without additional purification. **^{1}H NMR** (400 MHz, $CDCl_3$) δ 5.44 – 5.26 (m, 4H), 3.64 (td, *J* = 6.5, 4.6 Hz, 2H), 2.77 (t, *J* = 6.5 Hz, 2H), 2.05 (q, *J* = 6.9 Hz, 4H), 1.65 – 1.52 (m, 2H), 1.41 – 1.18 (m, 16H), 0.93 – 0.80 (m, 3H). The experimental data are in agreement with the literature report.[17] In a round-bottom flask equipped with a PTFE-coated stirring bar, the intermediate alcohol **SI-1r** (5.00 mmol, 1.00 equiv.) dissolved in CH_2Cl_2 (40 ml), then pyridinium chlorochromate (2.15 g, 10.0 mmol, 2.00 equiv.) was added and the reaction was

stirred at room temperature for 30 minutes, then filtered over a short pad of Celite®, rinsing with little CH_2Cl_2. The organic layer was dried *in vacuo* and the crude residue was purified by flash column chromatography on silica (pentane/EtOAc = 60/1), affording **1r** (583 mg, 2.20 mmol, 44 % over two steps) as a colourless liquid. **^{1}H NMR** (400 MHz, $CDCl_3$) δ 9.76 (t, *J* = 1.9 Hz, 1H), 5.45 – 5.26 (m, 4H), 2.77 (t, *J* = 6.3 Hz, 2H), 2.42 (td, *J* = 7.4, 1.9 Hz, 2H), 2.05 (q, *J* = 6.8 Hz, 4H), 1.63 (p, *J* = 7.0 Hz, 2H), 1.42 – 1.23 (m, 14H), 0.89 (t, *J* = 6.7 Hz, 3H). The experimental data are in agreement with the literature report.[18]

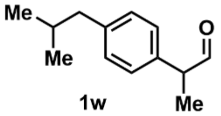

1w

2-(4-Isobutylphenyl)propanal (1w). In an oven-dried Schlenk tube equipped with a PTFE-coated stirring bar, *rac*-ibuprofene (1.03 mg, 5.00 mmol, 1.00 equiv.) was dissolved in dry THF (25 ml), then the solution was cooled to 0 °C and $LiAlH_4$ (380 mg, 10.00 mmol, 2.00 equiv.) was added portionwise, then the reaction was warmed at room temperature and stirred overnight. The reaction was diluted with Et_2O (30 ml), cooled to 0 °C and carefully quenched with water (400 µl), NaOH (2M, 400 µl), and water (1.2 ml). After warming to room temperature, MgSO4 was added and the suspension was filtered over a short pad Celite®, rinsing thoroughly with Et_2O and the solvent was removed *in vacuo*. The intermediate alcohol was dissolved in CH_2Cl_2 (30 ml) and pyridinium chlorochromate (2.15 g, 10.00 mmol, 2.00 equiv.) was added portionwise. The reaction was stirred for 90 minutes, then diluted with Et_2O (40 ml) and filtered over a pad of Celite®, thoroughly rinsing with Et_2O. The volatiles were removed *in vacuo*, then the residue was purified by flash column chromatography on silica (pentane/EtOAc = 60/1), affording **1w** (327 mg, 1.72 mmol, 34% over two steps) as a faint yellow oil. **^{1}H NMR** (400 MHz, $CDCl_3$) δ 9.67 (d, *J* = 1.5 Hz, 1H), 7.17 – 7.09 (m, 4H), 3.60 (q, *J* = 7.1 Hz, 1H), 2.47 (d, *J* = 7.2 Hz, 2H), 1.86 (hept, *J* = 6.7 Hz, 1H), 1.43 (d, *J* = 7.1 Hz, 3H), 0.90 (d, *J* = 6.6 Hz, 6H). The experimental data are in agreement with the literature report.[19]

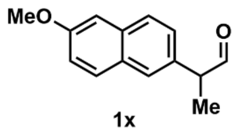

1x

(*S*)-2-(6-Methoxynaphthalen-2-yl)propanal (1x). In an oven-dried Schlenk tube equipped with a PTFE-coated stirring bar, naproxen (691 mg, 3.00 mmol, 1.00 equiv.) was dissolved in dry THF (15 ml), then the solution was cooled to 0 °C and $LiAlH_4$ (342 mg, 9.00 mmol, 3.00 equiv.) was added portionwise, then the reaction was warmed at room temperature and stirred overnight. The reaction was diluted with Et_2O (20 ml), cooled to 0 °C and carefully quenched with water (400 µl), NaOH (2M, 400 µl), and water (1.2 ml). After warming to room temperature, MgSO4 was added and the suspension was filtered over a short pad Celite®, rinsing thoroughly with Et_2O and the solvent was removed *in vacuo*. The intermediate alcohol was dissolved in CH_2Cl_2 (20 ml) and pyridinium chlorochromate (1.29 g, 6.00 mmol, 2.00 equiv.) was added portionwise. The reaction was stirred for 90 minutes, the diluted with Et_2O (40 ml) and filtered over a pad of Celite®, thoroughly rinsing with Et_2O. The volatiles were removed in vacuo, then the residue was purified by flash column chromatography on silica (pentane/EtOAc = 25/1 → 20/1), affording **1x** (478 mg, 2.23 mmol, 74% over two steps) as a white solid. **^{1}H NMR** (400 MHz, $CDCl_3$) δ 9.75 (d, *J* = 1.4 Hz, 1H), 7.75 (d, *J* = 8.5 Hz, 1H), 7.72 (d, *J* = 8.9 Hz, 1H), 7.60 (br s, 1H), 7.28 (dd, *J* = 8.4, 1.9 Hz, 1H), 7.17 (dd, *J* = 8.9, 2.5 Hz, 1H), 7.13 (d, *J* = 2.5 Hz, 1H), 3.93 (s, 3H), 1.52 (d, *J* = 7.0 Hz, 3H). The experimental data are in agreement with the literature report.[19]

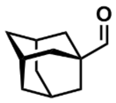

1z

Adamantane-1-carbaldehyde (1z). The title compound was synthesized according to a modified procedure from Olah and co-workers.[20] In an oven-dried round-bottom flask equipped with a PTFE-coated stirring bar, adamantylmethanol (832 mg, 5.00 mmol, 1.00 equiv.) was dissolved in dry CH_2Cl_2

(20 ml), then pyridinium chlorochromate (2.15 g, 10.0 mmol, 2.00 equiv.) was added in small portions. The reaction was stirred at room temperature for 2 hours, then diluted with Et_2O and filtered over a pad of Celite®, rinsing with additional Et_2O. The organic filtrates were washed once with NaOH 1M (40 ml), then the water layer was extracted once with Et_2O (30 ml). The combined organics were washed twice with water (20 ml each time), then dried over $MgSO_4$ and the solvent was removed *in vacuo*. The crude product was purified by flash column chromatography on silica (pentane/CH_2Cl_2 = 2/1), affording **1z** as a white foam (602 mg, 3.67 mmol, 73%). **^{1}H NMR** (400 MHz, $CDCl_3$) δ 9.31 (s, 1H), 2.10 – 2.03 (m, 3H), 1.82 – 1.64 (m, 12H). The experimental data are in agreement with the literature report.[21]

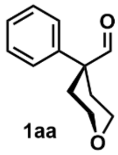

1aa

4-Phenyltetrahydro-2*H*-pyran-4-carbaldehyde (1aa). The compound was synthesized according to the following two-step procedure.

Step 1. In an oven-dried Schlenk tube equipped with a PTFE-coated stirring bar, diethyleneglycol (2.39 ml, 250 mmol, 1.25 equiv.) was charged under argon and cooled to 0 °C, then PBr_3 (1.90 ml, 20.0 mmol, 1.00 equiv.) was added dropwise over 30 minutes. The reaction was gently warmed at room temperature and stirred overnight. The reaction was diluted with CH_2Cl_2 (50 ml) and carefully quenched with water (40 ml), then the layers were separated and the organic layer was washed once with saturated $NaHCO_3$ (30 ml) and water (30 ml), dried over $MgSO_4$ and the solvent was removed *in vacuo*. The crude product **SI-1aa-1**, a colourless liquid, was used for the next step without additional purification. **GC-MS** (EI, 1.9-normal) @ 2.86 min, *m/z* = 232 (M^+). A mixture of the crude bromide **SI-1aa-1**, benzylcyanide (2.31 ml, 20.0 mmol, 1.00 equiv.) in dry Et_2O (14 ml) was added dropwise to an ice-cold suspension of NaH 60% in mineral oil (1.76 g, 44.0 mmol, 2.20 equiv.) in DMSO (14 ml), then the reaction was stirred for 20 minutes at 0 °C and gently warmed at room temperature. After 3 hours, the reaction was

carefully quenched with water (40 ml), extracted twice with EtOAc (30 ml each time), then the combined organic layers were washed twice with water (30 ml each time), dried over $MgSO_4$ and the solvent was removed *in vacuo*. The crude product was purified by flash column chromatography on silica (pentane/EtOAc = 8/1), affording the intermediate nitrile **SI-1aa-2** (2.77 g, 14.8 mmol, 74% over two steps) as a light yellow thick oil. **^{1}H NMR** (400 MHz, $CDCl_3$) δ 7.52 – 7.47 (m, 2H), 7.47 – 7.39 (m, 2H), 7.35 (tt, *J* = 7.2, 1.3 Hz, 1H), 4.09 (d, *J* = 12.3 Hz, 2H), 3.91 (td, *J* = 12.1, 2.2 Hz, 2H), 2.15 (ddd, *J* = 13.7, 11.9, 4.5 Hz, 2H), 2.06 (dq, *J* = 13.7, 2.1 Hz, 2H). **GC-MS** (EI, 1.9-normal) @ 3.52 min, *m/z* = 187 (M^+). The experimental data are in agreement with the literature report.[22]

Step 2. In an oven-dried Schlenk tube equipped with a PTFE-coated stirring bar, the intermediate nitrile **SI-1aa-2** (936 mg, 5.00 mmol, 1.00 equiv.) dissolved in dry CH_2Cl_2 (25 ml) and cooled to 0 °C, then a solution of DIBAL-H (1 M in hexanes, 5.0 ml, 5.00 mmol, 1.00 equiv.) was added dropwise. The reaction was stirred at 0 °C for 20 minutes, then quenched with HCl 1M (10 ml) and the layers were separated. The aqueous layer was extracted once with CH_2Cl_2 (30 ml), then the combined organic layers were filtered over a short pad of Celite® to remove insoluble byproducts, concentrated *in vacuo* and filtered over a short pad of silica, rinsing with CH_2Cl_2 After concentration *in vacuo*, the product was purified by flash column chromatography (pentane/EtOAc 10/1 → 7/1), affording **1aa** (782 mg, 4.11 mmol, 82%) as a colourless low-melting solid. *Note. Contains inseparable traces of residual nitrile, which does not interfere with the following step.* **R_f** (pentane/EtOAc = 7/1): 0.55. **^{1}H NMR** (400 MHz, $CDCl_3$) δ 9.41 (s, 1H), 7.45 – 7.28 (m, 5H), 3.91 (dt, *J* = 12.1, 4.0 Hz, 2H), 3.60 (ddd, *J* = 11.9, 10.9, 2.5 Hz, 2H), 2.40 (dq, *J* = 13.9, 2.0 Hz, 2H), 2.15 – 2.01 (m, 2H). **^{13}C{^{1}H} NMR** (101 MHz, $CDCl_3$) δ 201.0, 129.3, 127.9, 126.9, 125.6, 65.0, 52.3, 31.3. **HRMS** (ESI$^+$): calcd. for $C_{12}H_{14}O_2Na$ (M+Na$^+$): 213.0886. Found: 213.0883.

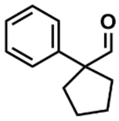

1ab

1-Phenylcyclopentane-1-carbaldehyde (1ab). The compound was synthesized according to the following two-step procedure.

<u>Step 1</u>. In an oven-dried Schlenk tube equipped with a PTFE-coated stirring bar, NaH 60% in mineral oil (1.32 g, 33.0 mmol, 2.20 equiv.) was suspended in dry DMSO (10 ml), then a solution of benzyl nitrile (1.73 ml, 15.0 mmol, 1.00 equiv.) and 1,4-dibromobutane (1.79 ml, 15.00 mmol, 1.00 equiv.) in dry Et_2O (10 ml) was added dropwise (*Warning. The reaction is exothermic and produced large amounts of gas, therefore it can be controlled using a water-ice bath*) and the reaction was stirred overnight. The reaction was carefully quenched with water (40 ml), extracted twice with EtOAc (30 ml each time), then the combined organic layers were washed twice with water (30 ml each time), dried over $MgSO_4$ and the solvent was removed *in vacuo*. The crude product was purified by flash column chromatography on silica (pentane/EtOAc = 20/1), affording the intermediate nitrile **SI-1ab** (1.35 g, 7.88 mmol, 52%) as a colourless liquid. **^{1}H NMR** (400 MHz, $CDCl_3$) δ 7.49 – 7.43 (m, 2H), 7.41 – 7.35 (m, 2H), 7.31 (tt, *J* = 7.3, 1.1 Hz, 1H), 2.53 – 2.43 (m, 2H), 2.14 – 2.01 (m, 4H), 2.01 – 1.88 (m, 2H). **GC-MS** (EI, 1.9-normal) @ 3.41 min, *m/z* = 171 (M^+). The experimental data are in agreement with the literature report.[22]

<u>Step 2.</u> In an oven-dried Schlenk tube equipped with a PTFE-coated stirring bar, the intermediate nitrile **SI-1ab** (856 mg, 5.00 mmol, 1.00 equiv.) dissolved in dry CH_2Cl_2 (25 ml) and cooled to 0 °C, then a solution of DIBAL-H (1 M in hexanes, 5.0 ml, 5.00 mmol, 1.00 equiv.) was added dropwise. The reaction was stirred at 0 °C for 10 minutes, then quenched with HCl 1M (10 ml) and the layers were separated. The aqueous layer was extracted once with CH_2Cl_2 (30 ml), then the combined organic layers were filtered over a short pad of Celite® to remove insoluble byproducts, concentrated *in vacuo* and filtered over a short pad of silica, rinsing with CH_2Cl_2. After concentration *in vacuo*, the product was purified by flash column chromatography (pentane/EtOAc = 100/1), affording **1ab** (629 mg, 3.61 mmol, 72%) as a colourless liquid. **^{1}H NMR** (400 MHz, $CDCl_3$) δ 9.39 (s, 1H), 7.38 – 7.32 (m, 2H), 7.29 – 7.22 (m, 3H), 2.57 – 2.46 (m, 2H), 1.92 – 1.83 (m, 2H), 1.80 –

1.70 (m, 2H), 1.69 – 1.57 (m, 2H). **GC-MS** (EI, 1.9-normal) @ 3.32 min, *m/z* = 174 (M^+). The experimental data are in agreement with the literature report.[22]

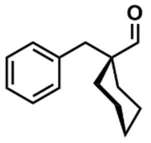

1ac

1-Benzylcyclohexane-1-carbaldehyde (1ac). The compound was synthesized according to a slightly modified procedure by Huang *et al*.[23]

In an oven-dried Schlenk tube equipped with a PTFE-coated stirring bar, freshly ground KOH (2.30 g, 41.0 mmol, 6.00 equiv.) was suspended in dry DME (21 ml), then benzyl bromide (2.50 ml, 21.0 mmol, 3.00 equiv.) and cyclohexylcarbaldehyde (863 µl, 7.00 mmol, 1.00 equiv.) were consecutively added. The vessel was sealed, and the reaction was heated at 50 °C for 2 hours, then cooled to room temperature and filtered to remove the residual KOH, rinsing with CH_2Cl_2. The filtrate was partitioned between CH_2Cl_2 and HCl 10%, then the aqueous layer was extracted twice with CH_2Cl_2 (40 ml each time), the combined organic layers dried over $MgSO_4$ and the solvent was removed *in vacuo*. The crude product was purified by flash column chromatography on silica (pentane/EtOAc = 150/1 → 100/1), affording **1ac** (770 mg, 3.81 mmol, 54%) as a colourless liquid. **^{1}H NMR** (400 MHz, $CDCl_3$) δ 9.52 (s, 1H), 7.28 – 7.17 (m, 3H), 7.10 – 7.02 (m, 2H), 2.72 (s, 2H), 1.96 – 1.84 (m, 2H), 1.66 – 1.49 (m, 4H), 1.36 – 1.15 (m, 4H). **GC-MS** (EI, 1.9-normal) @ 3.60 min, *m/z* = 202 (M^+). The experimental data are in agreement with the literature report.[23]

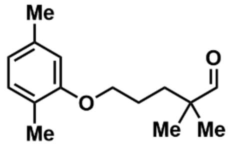

1ad

5-(2,5-dimethylphenoxy)-2,2-dimethylpentanal (1ad). In an oven-dried round-bottom flask equipped with a PTFE-coated stirring bar, gemfibrozil (751 mg, 3.00 mmol, 1.0 equiv.) was charged under argon counterflow, then anhydrous THF (15 ml) was added and the solution was cooled to 0 °C. BH_3·THF (1 M in THF,

9 ml, 9.00 mmol, 3.0 equiv.) was added dropwise, then the reaction was gently warmed at room temperature and the reaction was stirred for 16 hours. The reaction was carefully quenched with deionized H_2O (20 ml), was extracted three times with EtOAc (20 ml each time) and the combined organic layers were washed once with aqueous saturated $NaHCO_3$ (20 ml), were dried over $MgSO_4$ and the solvent was removed *in vacuo*. The residue was dissolved in anhydrous CH_2Cl_2 (20 ml), then cooled to 0 °C under argon atmosphere. Pyridinium chlorocromate (1.29 g, 6.00 mmol, 2.0 equiv.) was added in one portion, then the reaction was warmed to room temperature and stirred for additional 30 minutes. The reaction was diluted with Et_2O (50 ml), filtered over Celite®, then the organic layer was washed twice with aqueous NaOH (2M, 20 ml each time) and once with deionized water (20 ml), then was dried over $MgSO_4$ and the solvent was removed *in vacuo*. The residue was purified by flash column chromatography on silica (pentane/EtOAc = 40/1), affording **1ad** (361 mg, 1.54 mmol, 51% over two steps) as a faint yellow oil. **^{1}H NMR** (400 MHz, $CDCl_3$) δ 9.49 (s, 1H), 7.01 (d, *J* = 7.5 Hz, 1H), 6.66 (d, *J* = 7.6 Hz, 1H), 6.60 (s, 1H), 3.92 (t, *J* = 5.8 Hz, 2H), 2.31 (s, 3H), 2.17 (s, 3H), 1.76 – 1.63 (m, 4H), 1.10 (s, 6H). The experimental data are in agreement with the literature report.[15]

2.3. Synthesis of Olefins

General procedure 2 for the synthesis of olefins (GP2)

The procedure was adapted from Zhu *et al.*[24] In an oven-dried Schlenk flask equipped with a PTFE-coated stirring bar, the appropriate boronic acid (1.00 equiv.), K_2CO_3 (4.0 equiv.) and $Pd(PPh_3)_2Cl_2$ (3 mol%) were charged under air. The vessel was evacuated and back-filled with argon three times, then degassed THF:H_2O (3:2, 0.2 M) was added, followed by 1-bromo-1-trifluoromethylethane (1.5 equiv.). The reaction was heated at 60 °C overnight, the the reaction was cooled to room temperature and partitioned between Et_2O/H_2O. The layers were separated and the aqueous layer was extracted twice with Et_2O (approx. 5 ml $mmol^{-1}$). The combined organic layers were dried over $MgSO_4$, concentrated *in*

vacuo and purified by column chromatography on silica gel (pentane/Et_2O or pentane/EtOAc mixtures, as indicated in the individual entries) to afford the corresponding alkene.

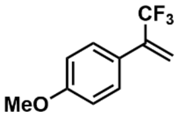

2a

1-Methoxy-4-(3,3,3-trifluoroprop-1-en-2-yl)benzene (2a). The compound was synthesized according to **GP2**, starting from 4-methoxyphenyl boronic acid (1.50 g, 10.0 mmol, 1.00 equiv.). Upon purification by flash column chromatography on silica (pentane 100%), **2a** (1.45 g, 7.20 mmol, 72%) was obtained as a colourless oil. **^{1}H NMR** (400 MHz, $CDCl_3$) δ 7.40 (d, *J* = 8.7 Hz, 2H), 6.93 – 6.89 (m, 2H), 5.87 (q, *J* = 1.4 Hz, 1H), 5.70 (q, *J* = 1.7 Hz, 1H), 3.83 (s, 3H). **^{19}F NMR** (377 MHz, $CDCl_3$) δ -64.9. The experimental data are in agreement with the literature report.[24]

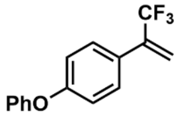

2b

1-phenoxy-4-(3,3,3-trifluoroprop-1-en-2-yl)benzene (2b). The title compound was synthesized according to the following two-step procedure.

Step 1. In a flame-dried Schlenk tube equipped with a PTFE-coated stirring bar, magnesium turnings (360 mg, 15 mmol, 1.5 equiv.) were charged, then a small crystal of iodine was added. Anhydrous THF (20 ml) was added and the pale brown solution was stirred for 10 minutes. 4-Phenoxybromobenzene (1.79 ml, 10.0 mmol, 1.0 equiv.) was added dropwise, then the reaction was left reacting at room temperature for 30 minutes. In another oven-dried Schlenk tube, ethyl trifluoromethylacetate (1.42 ml, 12.0 mmol, 1.2 equiv.) was dissolved in anhydrous THF (20 ml), then cooled to -78 °C. The solution of Grignard reagent was added dropwise, then the reaction was stirred at -78 °C for 5 hours. The reaction was carefully quenched with saturated NH_4Cl (10 ml), then was extracted with CH_2Cl_2 (30 ml each time). The combined organic layers were dried over

$MgSO_4$ and the solvent was removed *in vacuo*. **^{1}H NMR** (400 MHz, $CDCl_3$) δ 8.05 (dt, *J* = 8.0, 1.1 Hz, 2H), 7.49 – 7.38 (m, 2H), 7.31 – 7.22 (m, 1H), 7.15 – 7.08 (m, 2H), 7.07 – 7.00 (m, 2H). **^{19}F NMR** (376 MHz, $CDCl_3$) δ -71.1. The experimental data are in agreement with the literature report.[25]

Step 2. In a two-neck oven-dried round-bottom flask equipped with a PTFE-coated stirring bar, *t*-BuOK (1.23 g, 11.6 mmol, 1.5 equiv.) was charged under argon, then anhydrous THF (50 ml) was added and the suspension was cooled to 0 °C. Methyltriphenylphosphonium bromide (5.28 g, 14.8 mmol, 2.0 equiv.) was added in small portion, the the reaction was stirred at 0 °C for additional 30 minutes. 2,2,2-Trifluoro-1-(4-phenoxyphenyl)ethan-1-one (1.98 g, 7.44 mmol, 1.0 equiv.) was added dropwise, then the reaction was allowed to reach room temperature overnight. The reaction was carefully quenched with saturated aqueous NH_4Cl (1 ml), then the solvent was removed *in vacuo*. The residue was taken-up with the minimal amount of CH_2Cl_2, then pentane was added to precipitate triphenylphosphine oxide. The solid was removed by filtration over Celite®, rinsing throroughly with pentane. The organic layer was concentrated *in vacuo* and the residue was purified by flash column chromatography on silica (pentane 100%), affording **2b** (1.57 g, 5.95 mmol, 80%) as a colourless oil. **^{1}H NMR** (400 MHz, $CDCl_3$) δ 7.42 (d, *J* = 8.4 Hz, 2H), 7.37 (dddd, *J* = 9.0, 7.5, 2.6, 1.6 Hz, 2H), 7.15 (tt, *J* = 7.4, 1.1 Hz, 1H), 7.09 – 7.04 (m, 2H), 7.03 – 6.96 (m, 2H), 5.92 (q, *J* = 1.4 Hz, 1H), 5.74 (q, *J* = 1.7 Hz, 1H). **^{19}F NMR** (376 MHz, $CDCl_3$) δ -64.9. The experimental data are in agreement with the literature report.[24]

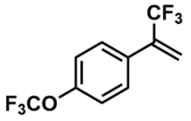

2c

1-(trifluoromethoxy)-4-(3,3,3-trifluoroprop-1-en-2-yl)benzene (2c). The compound was synthesized according to **GP2**, starting from 4-trifluoromethoxyphenyl boronic acid (618 mg, 3.00 mmol, 1.00 equiv.). Upon purification by flash column chromatography on silica (pentane 100%), **2c** (460

mg, 1.80 mmol, 60%) was obtained as a colourless oil. **^{1}H NMR** (400 MHz, $CDCl_3$) δ 7.48 (d, *J* = 8.6 Hz, 2H), 7.24 (d, *J* = 9.0 Hz, 2H), 6.00 (q, *J* = 1.4 Hz, 1H), 5.78 (q, *J* = 1.7 Hz, 1H). **^{19}F NMR** (376 MHz, $CDCl_3$) δ -57.9, -65.1. The experimental data are in accordance with the literature report.[26]

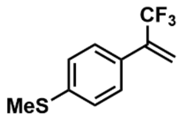

2d

Methyl(4-(3,3,3-trifluoroprop-1-en-2-yl)phenyl)sulfane (2d). The compound was synthesized according to **GP2**, starting from (4-(methylthio)phenyl)boronic acid (504 mg, 3.00 mmol, 1.00 equiv.). Upon purification by flash column chromatography on silica (pentane 100%), **2d** (625 mg, 2.71 mmol, 91%) was obtained as a colourless oil. **^{1}H NMR** (400 MHz, $CDCl_3$) δ 7.42 – 7.34 (m, 2H), 7.29 – 7.21 (m, 2H), 5.92 (q, *J* =1.4 Hz, 1H), 5.76 (q, *J* =1.7 Hz, 1H), 2.50 (s, 3H). **^{19}F NMR** (377 MHz, $CDCl_3$) δ -64.8. The experimental data are in agreement with the literature report.[27]

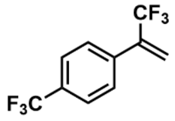

2e

1-(Trifluoromethyl)-4-(3,3,3-trifluoroprop-1-en-2-yl)benzene (2e). The compound was synthesized according to **GP2**, starting from (4-trifluoromethylphenyl)boronic acid (950 mg, 5.00 mmol, 1.00 equiv.). Upon purification by flash column chromatography on silica (pentane 100%), **2e** (891 mg, 3.71 mmol, 74%) was obtained as a colourless oil. **^{1}H NMR** (400 MHz, $CDCl_3$) δ 7.66 (d, *J* = 8.2 Hz, 2H), 7.58 (d, *J* = 8.1 Hz, 2H), 6.07 (q, *J* = 1.4 Hz, 1H), 5.85 (q, *J* = 1.6 Hz, 1H). **^{19}F NMR** (377 MHz, $CDCl_3$) δ -62.9, -64.9. The experimental data are in agreement with the literature report.[28]

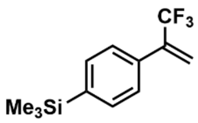

2f

Trimethyl(4-(3,3,3-trifluoroprop-1-en-2-yl)phenyl)silane (2f). The compound was synthesized according to **GP2**, starting from

(4-(trimethylsilyl)phenyl)boronic acid (582 mg, 3.00 mmol, 1.00 equiv.). Upon purification by flash column chromatography on silica (pentane 100%), **2f** (672 mg, 2.75 mmol, 92%) was obtained as a colourless oil. **^{1}H NMR** (400 MHz, $CDCl_3$) δ 7.59 – 7.50 (m, 2H), 7.48 – 7.41 (m, 2H), 5.96 (q, *J* = 1.4 Hz, 1H), 5.79 (q, *J* = 1.7 Hz, 1H), 0.28 (s, 9H). **^{19}F NMR** (376 MHz, $CDCl_3$) δ -64.7. The experimental data are in agreement with the literature report.[29]

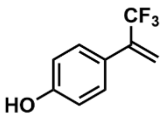

2g

4-(3,3,3-trifluoroprop-1-en-2-yl)phenol (2g). The compound was synthesized according to **GP2**, starting from 4-Hydroxyboronic acid (411 mg, 3.0 mmol, 1.00 equiv.). Upon purification by flash column chromatography on silica (pentane/EtOAc = 95/5 → 90/10), **2g** (211 mg, 1.12 mmol, 37%) was obtained as a pale-yellow oil. **^{1}H NMR** (400 MHz, $CDCl_3$) δ 7.39 – 7.31 (m, 1H), 6.88 – 6.80 (m, 1H), 5.86 (q, *J* = 1.4 Hz, 1H), 5.69 (q, *J* = 1.7 Hz, 1H), 5.08 (s, 1H). **^{19}F NMR** (377 MHz, $CDCl_3$) δ -64.9. The experimental data are in agreement with the literature report.[30]

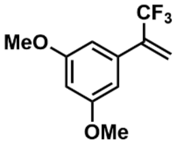

2h

1,3-Dimethoxy-5-(3,3,3-trifluoroprop-1-en-2-yl)benzene (2h). The compound was synthesized according to **GP2**, starting from 3,5-dimethoxyphenylboronic acid (546 mg, 3.00 mmol, 1.00 equiv.). The crude product was purified by flash column chromatographyv on silica (pentane/EtOAc = 80/1), affording **2h** (531 mg, 2.29 mmol, 76%) as a colourless oil. **^{1}H NMR** (400 MHz, $CDCl_3$) δ 6.60 – 6.56 (m, 2H), 6.49 (t, *J* = 2.3 Hz, 1H), 5.95 (q, *J* = 1.4 Hz, 1H), 5.77 (q, *J* = 1.7 Hz, 1H), 3.81 (s, 6H). **^{19}F NMR** (376 MHz, $CDCl_3$) δ -64.8. The experimental data are in accordance with the literature report.[31]

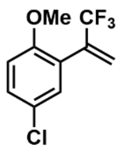

2i

4-Chloro-1-methoxy-2-(3,3,3-trifluoroprop-1-en-2-yl)benzene (2i). The title compound was synthesized according to the following four-step procedure.

Step 1**.** A 100 mL round bottom flask was charged with chloroform (30 mL) and 4-chlorophenol (1.02 mL, 10 mmol, 1.0 equiv.). A solution of Br_2 (0.56 mL, 11 mmol, 1.1 equiv.) in chloroform (4 mL) was added dropwise. After stirring for one hour, *p*-toluenesulfonyl acid monohydrate (0.190 g, 1.0 mmol, 10 mol%) was added. After 20 minutes, the reaction was quenched with saturated potassium thiosulphate solution. 20 ml of water was added, and the aqueous layer was extracted three times with DCM (20 mL each time). The combined organic layers were dried over $MgSO_4$, filtered and purified by flash column chromatography (pentane/EtOAc = 95:5 → 90:10) to obtain **SI-2i-1** (1.70 g, 8.18 mmol, 82%) as a colourless oil. **^{1}H NMR** (400 MHz, $CDCl_3$) δ 7.46 (d, *J* = 2.4 Hz, 1H), 7.19 (dd, *J* = 8.7, 2.4 Hz, 1H), 6.95 (d, *J* = 8.7 Hz, 1H), 5.51 (s, 1H). The experimental data are in agreement with the literature report.[32]

Step 2**.** A 50 mL round-bottom flask was charged with **SI-2i-1** (1.70 g, 8.2 mmol, 1.0 equiv.), K_2CO_3 (2.26 g, 16.4 mmol, 2.0 equiv.) and acetone (16 mL). While stirring, MeI (0.76 mL, 12.27 mmol, 1.5 equiv.) was added in one portion. After stirring overnight at room temperature, the reaction was filtered and concentrated by rotary evaporation. The remaining residue was dissolved in CH_2Cl_2 (20 mL) and washed once with water (20 mL). The organic layer was dried over $NaSO_4$, filtered and the solvent was removed by rotary evaporation to obtain **SI-2i-2** (1.73 g, 7.69 mmol, 94%) as a colourless oil, which was used for the next step without further purification. **^{1}H NMR** (400 MHz, $CDCl_3$) δ 7.53 (d, *J* = 2.6 Hz, 1H), 7.24 (dd, *J* = 8.8, 2.5 Hz, 1H), 6.82 (d, *J* = 8.8 Hz, 1H), 3.88 (s, 3H). The experimental data are in agreement with the literature report.[32]

***Step 3*.** **SI-2i-2** (1.73 g, 7.68 mmol, 1.0 equiv.) was transferred to an oven-dried Schlenk tube. Under argon, anhydrous THF (20 mL) was added and the solution was cooled to -78 °C. *n*BuLi (1.6 M in hexanes, 6.24 mL, 9.98 mmol, 1.3 equiv.) was added dropwise. After stirring of the solution at -78 °C for 90 minutes, tri*iso*propyl borate (3.52 mL, 15.36 mmol, 2.0 equiv.) was added dropwise. After the solution was stirred at room temperature overnight, ethyl acetate (20 mL) was added. The reaction was acidified to pH 1 by addition of HCl (2M). The organic phase was separated and the aqueous phase was extracted three times with ethyl acetate (20 mL each time). The combined organic phases were dried with Na_2SO_4, filtered and concentrated *in vacuo*. The residue was purified by flash column chromatography (pentane/EtOAc = 4/1) to obtain **SI-2i-3** (535 mg, 2.86 mmol, 37%) as a white solid. **R_f** (pentane/EtOAc = 4/1): 0.33 and 0.20. *The NMR was inconclusive due to peak broadening from the presence of a mixture of monomeric boronic acid and dehydrated boroxine.* **HRMS (ESI):** *m/z* calculated for $[M+Na]^+$ ($C_7H_8BClNaO_3^+$) 209.0153, found: 209.0149.

Step 4. Compound **2i** was synthesized following **GP2** with **SI-2i-3** (485 mg, 2.60 mmol, 1.0 equiv.). The product was purified by flash column chromatography (pentane/EtOAc = 97.5/2.5), to obtain **2i** (280 mg, 1.18 mmol, 46%) as a pale-yellow oil. **R_f** (pentane/EtOAc = 9/1): 0.84. **^{1}H NMR** (400 MHz, $CDCl_3$) δ 7.30 (dd, *J* = 8.8, 2.7 Hz, 1H), 7.20 (d, *J* = 2.6 Hz, 1H), 6.86 (d, *J* = 8.9 Hz, 1H), 6.10 (q, *J* = 1.5 Hz, 1H), 5.65 (q, *J* = 1.2 Hz, 1H), 3.80 (s, 3H). **^{13}C{^{1}H} NMR** (101 MHz, $CDCl_3$) δ 156.2, 135.1 (q, *J* = 32.1 Hz), 130.6, 130.0, 125.4, 124.2 (q, *J* = 5.2 Hz), 121.5 (q, *J* = 274.0 Hz), 112.5, 56.1. **^{19}F NMR** (377 MHz, $CDCl_3$) δ -65.7. **HRMS** (ESI^+): calcd. for $C_{10}H_8ClF_3NaO_3$ (M^+): 236.0216. Found: 236.0212. **IR** (ATR) ῦ = 2949 (w), 2943 (w), 2839 (w), 1489 (s), 1462 (w), 1392 (w), 1330 (w), 1283 (w), 1248 (m), 1166 (s), 1122 (s), 1070 (m), 1026 (m), 953 (m), 885 (m), 810 (m), 699 (w), 641 (m), 631 (m).

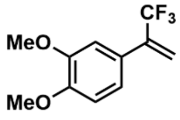

2j

1-(3,4-Dimethoxyphenyl)-2,2,2-trifluoroethan-1-one (2j). The title compound was synthessized according to the following two-step procedure.

***Step 1*.** An oven dried Schlenk flask was charged with magnesium turnings (316 mg, 13 mmol, 1.3 equiv.), and placed under argon atmosphere. Anhydrous THF (24 mL) and catalytic amounts of iodine (tiny crystal) were added, and the suspension was stirred for two and a half hours until the colour disappeared. Then 4-bromo-1,2-dimethoxybenzene (1.73 mL, 12 mmol, 1.2 equiv.) was added in one portion and the suspension was stirred for another hour. A separate Schlenk tube was placed under argon and charged with anhydrous THF (20 mL) and 2,2,2-trifluoroacetyl bromide (1.2 mL, 10 mmol, 1.0 equiv.). The reaction solution was cooled down to -78 °C and the Grignard reagent solution was added dropwise over the course of 20 minutes. The solution was stirred until full consumption was observed based on GCMS (90 minutes). At -78 °C, few drops of saturated NH_4Cl solution was added, the reaction was allowed to warm to room temperature, and the solvent was removed by rotary evaporation. The remaining crude was dissolved in diethyl ether (40 mL) and washed three times with water (30 mL each time). The organic layer was dried over $MgSO_4$ and filtered. Product **SI-2j** was purified by flash column chromatography (pentane/EtOAc = 95/5) and obtained as a white solid (1.02 g, 4.53 mmol, 45%). **^{1}H NMR** (400 MHz, $CDCl_3$) δ 7.73 (dp, *J* = 8.6, 1.7 Hz, 1H), 7.57 (d, *J* = 2.1 Hz, 1H), 6.95 (d, *J* = 8.6 Hz, 1H), 3.99 (s, 3H), 3.95 (s, 3H). The experimental data are in agreement with the literature report.[33]

***Step 2*.** An oven dried Schlenk flask was charged with methyltriphenylphosphonium bromide (1.82 g, 5.01 mmol, 1.25 equiv.), and placed under argon atmosphere. Anhydrous THF (10 mL) was added and the suspension was placed on an ice bath. While stirring, *n*BuLi (1.6 M in hexanes, 3.07 mL, 4.92 mmol, 1.2 equiv.) was added dropwise. After 30 minutes of stirring, the

reaction was cooled down to -78 °C and **SI-2j** (0.992 g, 4.079 mmol, 1.0 equiv.) was added dropwise. After stirring of the solution for one hour at at 0°C, the reaction mixture was removed from the ice bath and stirred at room temperature overnight. To the reaction few drops of NH_4Cl solution (sat.) was added. The solvent was removed by rotary evaporation. The residue was dissolved in CH_2Cl_2 (5 mL) and pentane (25 mL) was added under vigorous stirring. The mixture was filtered to remove the insoluble triphenylphosphine oxide and the remaining solids were washed with pentane (25 mL), and filtered again. The obtained filtrate was combined and subjected to flash column chromatography (pentane/EtOAc = 95/5) to obtain **2j** (747 mg, 3.20 mmol, 79%) as a colourless oil. **^{1}H NMR** (400 MHz, $CDCl_3$) δ 7.04 (ddd, *J* = 8.4, 2.1, 0.9 Hz, 1H), 6.96 (d, *J* = 2.1 Hz, 1H), 6.87 (d, *J* = 8.4 Hz, 1H), 5.89 (q, *J* = 1.4 Hz, 1H), 5.71 (q, *J* = 1.7 Hz, 1H), 3.90 (s, 6H). The experimental data are in agreement with the literature report.[34]

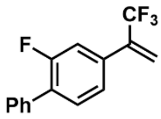

2k

2-fluoro-4-(3,3,3-trifluoroprop-1-en-2-yl)-1,1'-biphenyl (2k). The compound was synthesized according to **GP2**, starting from 2-fluoro-4-biphenylylboronic acid (648 mg, 3.00 mmol, 1.00 equiv.). The crude product was purified by flash column chromatography on silica (pentane 100%), affording **2k** (706 mg, 2.65 mmol, 88%) as a colourless oil. **R*f*** (pentane 100%): 0.70. **^{1}H NMR** (400 MHz, $CDCl_3$) δ 7.60 – 7.55 (m, 2H), 7.51 – 7.44 (m, 3H), 7.40 (tt, *J* = 7.2, 1.3 Hz, 1H), 7.36 – 7.32 (m, 1H), 7.32 – 7.27 (m, 1H), 6.03 (q, *J* = 1.4 Hz, 1H), 5.87 (q, *J* = 1.7 Hz, 1H). **^{13}C{^{1}H} NMR** (101 MHz, $CDCl_3$) δ 159.7 (d, *J* = 248.5 Hz), 137.8 (q, *J* = 30.5 Hz), 135.1 (d, *J* = 1.5 Hz), 134.4 (d, *J* = 8.5 Hz), 131.0 (d, *J* = 4.1 Hz), 129.8 (d, *J* = 13.6 Hz), 129.1 (d, *J* = 3.0 Hz), 128.7, 128.2, 123.4 (apt dd, *J* = 3.6, 1.2 Hz), 123.2 (q, *J* = 274.0 Hz), 121.2 (q, *J* = 5.7 Hz), 115.3 (dd, *J* = 25.0, 1.0 Hz). **^{19}F NMR** (377 MHz, $CDCl_3$) δ -64.8, -117.2. **HRMS** (ESI$^+$): calcd. for $C_{15}H_{10}F_4$ (M $^+$): 266.0713. Found: 266.0712. **IR** (ATR) ῦ = 3050 (w), 3027 (w), 1581 (w), 1553 (w), 1486 (m), 1397

(m), 1362 (m), 1347 (m), 1222 (m), 1161 (s), 1122 (s), 1077 (s), 938 (m), 876 (m), 835 (m), 767 (m), 695 (s).

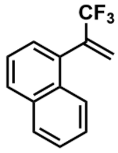

2l

1-(3,3,3-trifluoroprop-1-en-2-yl)naphthalene (2l). The compound was synthesized according to **GP2**, starting from 2-nathpthylboronic acid (516 mg, 3.00 mmol, 1.00 equiv.). The crude product was purified by flash column chromatography on silica (pentane 100%), affording **2l** (587 mg, 2.64 mmol, 88%) as a colourless oil. **^{1}H NMR** (400 MHz, $CDCl_3$) δ 7.97 – 7.92 (m, 1H), 7.92 – 7.86 (m, 2H), 7.56 – 7.49 (m, 2H), 7.48 (d, *J* = 8.0 Hz, 1H), 7.44 (d, *J* = 7.0 Hz, 1H), 6.34 (q, *J* = 1.5 Hz, 1H), 5.68 (q, *J* = 1.3 Hz, 1H). The experimental data are in accordance with the literature report.[28]

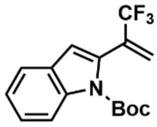

2m

***tert*-Butyl 2-(3,3,3-trifluoroprop-1-en-2-yl)-1*H*-indole-1-carboxylate (2m).** The compound was synthesized according to **GP2**, starting from 1-Boc-1*H*-indole-2-boronic acid pinacol ester (783 mg, 3.00 mmol, 1.00 equiv.). The crude product was purified by flash column chromatography on silica (pentane/CH_2Cl_2 = 95/5 → 90/10), affording **2m** (442 mg, 1.41 mmol, 47%) as a colourless oil. **R_f** (pentane/CH_2Cl_2 = 90/10): 0.23. **^{1}H NMR** (400 MHz, $CDCl_3$) δ 8.19 (dq, *J* = 8.5, 0.9 Hz, 1H), 7.56 (dt, *J* = 7.8, 1.0 Hz, 1H), 7.41 – 7.22 (m, 3H), 6.64 (d, *J* = 0.8 Hz, 1H), 6.07 (q, *J* = 1.4 Hz, 1H), 5.76 (q, *J* = 1.1 Hz, 1H), 1.63 (s, 9H). **^{19}F NMR** (377 MHz, $CDCl_3$) δ -65.5. **^{13}C{^{1}H} NMR** (101 MHz, $CDCl_3$) δ 149.7, 137.1, 133.8 (q, *J* = 32.8 Hz), 131.4, 128.5* (m), 125.2, 123.1 (q, *J* = 4.5 Hz), 123.2*, 122.6 (q, *J* = 274.0 Hz), 120.9, 115.6, 112.4, 84.8, 28.0. **overlapping signals.* **HRMS** (ESI^{+}): calcd. for $C_{16}H_{16}F_3NNaO_2$ (M^{+}): 334.1031. Found: 334.1025.

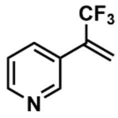

2n

3-(3,3,3-Trifluoroprop-1-en-2-yl)pyridine (2n). The compound was synthesized according to **GP2**, starting from 4 pyridin-3-ylboronic acid (369 mg, 3.00 mmol, 1.00 equiv.). The crude product was purified by flash column chromatography on silica (pentane/EtOAc = 8/2 → 6/4), affording **2n** (141 mg, 0.81 mmol, 27%) as a colourless oil. ^{1}**H NMR** (400 MHz, $CDCl_3$) δ 8.71 (d, *J* = 2.4 Hz, 1H), 8.64 (dd, *J* = 4.9, 1.6 Hz, 1H), 7.93 – 7.57 (m, 1H), 7.47 – 7.24 (m, 1H), 6.08 (d, *J* = 1.5 Hz, 1H), 5.86 (d, *J* = 1.7 Hz, 1H). ^{19}**F NMR** (377 MHz, $CDCl_3$) δ -65.2. Spectroscopic data are in agreement with those in the literature.[35]

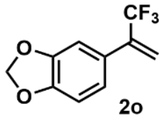

2o

5-(3,3,3-Trifluoroprop-1-en-2-yl)benzo[*d*][1,3]dioxole (2o). The compound was synthesized according to **GP2**, starting from 3,4-methylenedioxyphenylboronic acid (495 mg, 3.00 mmol, 1.00 equiv.). The crude product was purified by flash column chromatography on silica (pentane/EtOAc = 99/1), affording **2n** (202 mg, 0.93 mmol, 31%) as a colourless oil. ^{1}**H NMR** (400 MHz, $CDCl_3$) δ 6.97 – 6.92 (m, 2H), 6.81 (dd, *J* = 7.8, 0.8 Hz, 1H), 5.99 (s, 2H), 5.88 (q, *J* = 1.3 Hz, 1H), 5.68 (q, *J* = 1.8 Hz, 1H). Spectroscopic data are in agreement with those in the literature.[29]

3. Substrate Scope

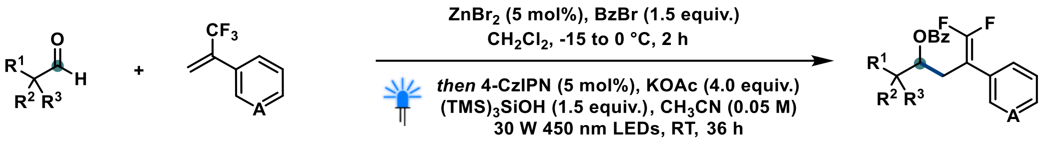

General procedure GP3. In an oven-dried Schlenk tube equipped with a PTFE-coated stirring bar, $ZnBr_2$ (0.015 mmol, 5 mol%) and the appropriate aldehyde **1** (*if solid or gum*, 0.3 mmol, 1.50 equiv.) were charged under air, then the vessel was evacuated and back-filled three times with argon. Dry CH_2Cl_2 (0.2 ml) was added, then the solution was cooled to -15 °C and benzoyl bromide (0.3 mmol, 1.50 equiv.) was added dropwise, followed by the appropriate aldehyde **1** (*if liquid*, 0.3 mmol, 1.50 equiv.). The reaction was stirred at 0 °C for two hours (*usually, a change in color from colourless to yellow-orange could be observed*). In another oven-dried Schlenk tube equipped with a PTFE-coated stirring bar, 4-CzIPN (10 µmol, 5 mol%), KOAc (0.8 mmol, 4.0 equiv.) and the appropriate alkene **2** (0.2 mmol, 1.00 equiv.) were charged under air, then the vessel was evacuated and back-filled three times with argon. Dry MeCN (3 ml, 0.075 M) was added, then the bromide solution was transferred via syringe, rinsing with MeCN (1 ml, 0.025 M), followed by $(TMS)_3SiOH$ (0.3 mmol, 1.50 equiv.). The reaction was further degassed by freeze-pump-thaw (three times), then sealed and irradiated with 30W 450 nm LEDs using the standard set-up. After 36 hours, the irradiation was interrupted, then the reaction was filtered through a short pad of silica, rinsing with EtOAc. The volatiles were removed *in vacuo*, then the crude residue was purified by flash column chromatography on silica (pentane/EtOAc *or* pentane/Et_2O mixtures, *as detailed in the individual entries*), affording the corresponding coupling product.

Note. Polar unprotected groups (e.g. alcohols, amines) are not tolerated as functional groups on the aldehyde coupling partner, due to side-reactivity with electrophilic benzoyl bromide.

3.1. Aldehydes Scope

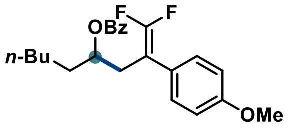

1,1-Difluoro-2-(4-methoxyphenyl)non-1-en-4-yl benzoate (3). According to the **GP3**, using hexanal (0.3 mmol, 1.5 equiv.), benzoyl bromide (0.3 mmol, 1.5 equiv.), alkene **2a** (0.2 mmol, 1.0 equiv.), 4CzIPN (10 μmol, 5 mol%), $(TMS)_3SiOH$ (0.3 mmol, 1.5 equiv.), KOAc (0.8 mmol, 4.0 equiv.) in MeCN (4 ml) and stirring at room temperature for 36 h under 30W blue LEDs irradiation. The reaction mixture was filtered through a pad of silica gel and concentrated *in vacuo*, then purified by chromatography (pentane/Et_2O = 9/1) to give **3** (57.3 mg, 0.148 mmol, 74%) as a colorless oil. $\mathbf{R_f}$ (pentane/ Et_2O = 9/1) 0.31. **^{1}H NMR** (599 MHz, $CDCl_3$) δ ppm 7.89 – 7.84 (m, 2H), 7.55 – 7.49 (m, 1H), 7.41 – 7.36 (m, 2H), 7.25 – 7.20 (m, 2H), 6.83 (d, *J* = 8.8 Hz, 2H), 5.12 (ddd, *J* = 7.5, 5.1, 2.3 Hz, 1H), 3.76 (s, 3H), 2.83 (dddd, *J* = 14.6, 7.4, 2.4, 1.7 Hz, 1H), 2.77 – 2.66 (m, 1H), 1.75 – 1.59 (m, 2H), 1.36 (ddt, *J* = 11.5, 6.4, 2.1 Hz, 2H), 1.30 – 1.22 (m, 4H), 0.86 (t, *J* = 7.0 Hz, 3H). **^{13}C{^{1}H} NMR** (101 MHz, $CDCl_3$) δ ppm 166.2, 158.9, 154.3 (dd, *J* = 289.5, 287.8 Hz), 132.8, 130.5, 129.5, 129.4 (t, *J* = 3.1 Hz), 128.3, 125.4 (t, *J* = 2.3 Hz), 114.1, 88.8 (dd, *J* = 20.3, 16.6 Hz), 73.1 (t, *J* = 3.0 Hz), 55.3, 33.8, 32.7, 31.7, 25.0, 22.6, 14.1. **^{19}F{^{1}H} NMR** (377 MHz, $CDCl_3$) δ ppm -91.0 (d, *J* = 42.0 Hz), -91.1 (d, *J* = 42.0 Hz). **HRMS** (ESI$^+$): calcd. for $C_{23}H_{26}F_2O_3Na$ (M + Na$^+$): 411.17422. Found: 411.17372.

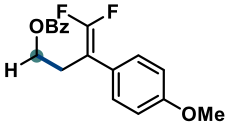

4,4-Difluoro-3-(4-methoxyphenyl)but-3-en-1-yl benzoate (4). According to the **GP3**, using polyformaldehyde (0.3 mmol, 1.5 equiv.), benzoyl bromide (0.3 mmol, 1.5 equiv.), alkene **2a** (0.2 mmol, 1.0 equiv.), 4CzIPN (10 μmol, 5 mol%), $(TMS)_3SiOH$ (0.3 mmol, 1.5 equiv.), KOAc (0.8 mmol, 4.0 equiv.) in MeCN (4 ml) and stirring at room temperature for 36 h under 30W blue LEDs irradiation. The reaction mixture was filtered through a pad of silica gel and concentrated *in vacuo*, then purified by chromatography (pentane/Et_2O = 95/5) to give **4** (14.5 mg, 0.046

mmol, 23%) as a colorless oil. **R$_f$** (pentane/ Et_2O = 95/5) 0.18. **^{1}H NMR** (599 MHz, $CDCl_3$) δ 7.96 – 7.90 (m, 2H), 7.54 (d, *J* = 7.4 Hz, 1H), 7.41 (dd, *J* = 8.2, 7.5 Hz, 2H), 7.29 – 7.24 (m, 2H), 6.89 (d, *J* = 8.8 Hz, 2H), 4.33 (t, *J* = 6.6 Hz, 2H), 3.80 (s, 2H), 2.84 (t, *J* = 6.6 Hz, 2H). **^{13}C{^{1}H} NMR** (151 MHz, $CDCl_3$) δ 166.4, 158.9, 154.2 (dd, *J* = 290.1, 287.7 Hz), 132.9, 130.0, 129.5, 129.4 (t, *J* = 3.2 Hz), 128.3, 125.4 (t, *J* = 2.3 Hz), 114.1, 88.8 (dd, *J* = 21.0, 15.8 Hz), 62.6 (d, *J* = 3.7 Hz), 55.2, 27.7 (d, *J* = 2.0 Hz). **^{19}F NMR** (564 MHz, $CDCl_3$) δ -90.5 (dt, *J* = 42.0, 1.1 Hz), -91.2 (dt, *J* = 42.1, 2.7 Hz). **HRMS** (ESI$^+$): calcd. for $C_{18}H_{16}F_2O_3Na$ (M + Na$^+$): 341.09597. Found: 341.09598.

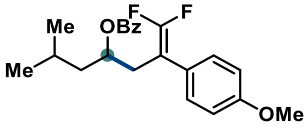

1,1-Difluoro-2-(4-methoxyphenyl)-6-methylhept-1-en-4-yl benzoate (5). According to the **GP3**, using *iso*pentanal (0.3 mmol, 1.5 equiv.), benzoyl bromide (0.3 mmol, 1.5 equiv.), alkene **2a** (0.2 mmol, 1.0 equiv.), 4CzIPN (10 μmol, 5 mol%), $(TMS)_3SiOH$ (0.3 mmol, 1.5 equiv.), KOAc (0.8 mmol, 4.0 equiv.) in MeCN (4 ml) and stirring at room temperature for 36 h under 30W blue LEDs irradiation. The reaction mixture was filtered through a pad of silica gel and concentrated *in vacuo*, then purified by chromatography (pentane/Et_2O = 95/5) to give **5** (54.6 mg, 0.146 mmol, 73%) as a colorless oil. **R$_f$** (pentane/ Et_2O = 95/5) 0.21. **^{1}H NMR** (599 MHz, $CDCl_3$) δ 7.86 (dd, *J* = 7.8, 2.0 Hz, 2H), 7.52 (t, *J* = 7.3 Hz, 1H), 7.38 (t, *J* = 7.2 Hz, 2H), 7.23 (d, *J* = 8.2 Hz, 2H), 6.85 – 6.78 (m, 2H), 5.21 (dd, *J* = 8.6, 5.0 Hz, 1H), 3.75 (dd, *J* = 2.6, 1.3 Hz, 3H), 2.90 – 2.79 (m, 1H), 2.70 (ddd, *J* = 14.6, 5.4, 2.7 Hz, 1H), 1.67 (ddt, *J* = 19.2, 13.2, 7.5 Hz, 2H), 1.43 (td, *J* = 9.7, 9.2, 4.5 Hz, 1H), 0.93 – 0.89 (m, 3H), 0.88 – 0.83 (m, 3H). **^{13}C{^{1}H} NMR** (101 MHz, $CDCl_3$) δ 166.0, 158.7, 154.3 (dd, *J* = 289.8, 287.5 Hz), 132.7, 130.3, 129.5, 129.4 (t, *J* = 3.1 Hz), 128.1, 125.4 (t, *J* = 2.3 Hz), 113.9, 88.8 (dd, *J* = 21.0, 15.8 Hz), 71.6, 55.1, 42.8, 33.1 (d, *J* = 1.8 Hz), 24.7, 23.1, 22.1. **^{19}F{^{1}H} NMR** (377 MHz, $CDCl_3$) δ -91.0 (d, *J* = 42.1 Hz), -91.2 (d, *J* = 42.0 Hz). **HRMS** (ESI$^+$): calcd. for $C_{22}H_{24}F_2O_3Na$ (M + Na$^+$): 397.15857. Found: 397.15842.

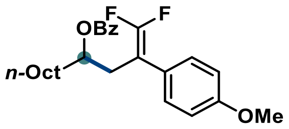

1,1-Difluoro-2-(4-methoxyphenyl)dodec-1-en-4-yl benzoate (6). According to the **GP3**, using nonanal (0.3 mmol, 1.5 equiv.), benzoyl bromide (0.3 mmol, 1.5 equiv.), alkene **2a** (0.2 mmol, 1.0 equiv.), 4CzIPN (10 µmol, 5 mol%), $(TMS)_3SiOH$ (0.3 mmol, 1.5 equiv.), KOAc (0.8 mmol, 4.0 equiv.) in MeCN (4 ml) and stirring at room temperature for 36 h under 30W blue LEDs irradiation. The reaction mixture was filtered through a pad of silica gel and concentrated *in vacuo*, then purified by chromatography (pentane/Et_2O = 95/5) to give **6** (67.1 mg, 0.151 mmol, 76%) as a colorless oil. **R*f*** (pentane/ Et_2O = 95/5) 0.41. **^{1}H NMR** (500 MHz, $CDCl_3$) δ ppm 7.91 – 7.83 (m, 2H), 7.55 – 7.50 (m, 1H), 7.41 – 7.36 (m, 2H), 7.26 – 7.21 (m, 2H), 6.87 – 6.81 (m, 2H), 5.12 (ddd, *J* = 7.4, 5.1, 2.3 Hz, 1H), 3.76 (s, 3H), 2.83 (ddt, *J* = 14.6, 7.4, 2.1 Hz, 1H), 2.71 (ddt, *J* = 14.6, 5.1, 2.4 Hz, 1H), 1.74 – 1.62 (m, 2H), 1.42 – 1.17 (m, 14H), 0.88 (t, *J* = 7.0 Hz, 3H). **^{13}C{^{1}H} NMR** (126 MHz, $CDCl_3$) δ ppm 166.0, 158.8, 154.3 (dd, *J* = 289.4, 287.9 Hz), 132.7, 130.4, 129.5, 129.4 (t, *J* = 3.1 Hz), 128.2, 125.4 (t, *J* = 2.3 Hz), 114.0, 88.9 (dd, *J* = 20.1, 16.7 Hz), 73.1, 55.1, 33.7, 32.6, 31.9, 29.5, 29.4, 29.4, 29.3, 25.2, 22.7, 14.1. **^{19}F{^{1}H} NMR** (470 MHz, $CDCl_3$) δ ppm -91.0 (d, *J* = 42.0 Hz), -91.1 (d, *J* = 42.0 Hz). **HRMS** (ESI^+): calcd. for $C_{27}H_{34}F_2O_3Na$ (M + Na^+): 467.23682. Found: 467.23654.

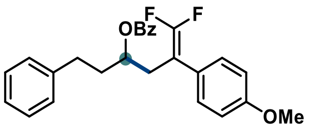

6,6-Difluoro-5-(4-methoxyphenyl)-1-phenylhex-5-en-3-yl benzoate (7). According to the **GP3**, using hydrocinnamaldehyde (0.3 mmol, 1.5 equiv.), benzoyl bromide (0.3 mmol, 1.5 equiv.), alkene **2a** (0.2 mmol, 1.0 equiv.), 4CzIPN (10 µmol, 5 mol%), $(TMS)_3SiOH$ (0.3 mmol, 1.5 equiv.), KOAc (0.8 mmol, 4.0 equiv.) in MeCN (4 ml) and stirring at room temperature for 36 h under 30W blue LEDs irradiation. The reaction mixture was filtered through a pad of silica gel and concentrated *in vacuo*, then purified by chromatography (pentane/Et_2O = 95/5) to give **7** (51.9 mg, 0.123 mmol, 61%) as a colorless oil. **R*f*** (pentane/ Et_2O = 95/5) 0.16. **^{1}H NMR** (599 MHz, $CDCl_3$) δ 7.91 – 7.87 (m, 2H), 7.58 – 7.53 (m, 1H), 7.43 – 7.39 (m, 2H), 7.29 –

7.22 (m, 4H), 7.19 (d, *J* = 7.4 Hz, 1H), 7.14 (ddt, *J* = 7.7, 1.3, 0.6 Hz, 2H), 6.85 – 6.82 (m, 2H), 5.19 (ddd, *J* = 7.5, 4.0, 1.7 Hz, 1H), 3.77 (s, 3H), 2.93 – 2.86 (m, 1H), 2.81 – 2.74 (m, 1H), 2.72 (dt, *J* = 10.2, 4.7 Hz, 1H), 2.66 (dt, *J* = 10.1, 7.0 Hz, 1H), 2.11 – 2.04 (m, 1H), 2.04 – 1.96 (m, 1H). **^{13}C{^{1}H} NMR** (151 MHz, $CDCl_3$) δ 166.0, 158.8, 154.3 (dd, *J* = 289.8, 287.8 Hz), 141.3, 132.8, 130.2, 129.5, 129.4 (t, *J* = 3.1 Hz), 128.4, 128.3, 128.2, 126.0, 125.3 (dd, *J* = 3.3, 2.2 Hz), 114.0, 88.7 (dd, *J* = 20.7, 16.4 Hz), 72.6 (t, *J* = 3.0 Hz), 55.2, 35.3, 32.5 (d, *J* = 1.6 Hz), 31.6. **^{19}F NMR** (564 MHz, $CDCl_3$) δ -90.8 (dt, *J* = 41.9, 2.6 Hz), -90.9 (dt, *J* = 41.8, 1.4 Hz). **HRMS** (ESI^+): calcd. for $C_{26}H_{24}F_2O_3Na$ (M + Na^+): 445.15857. Found: 445.15826.

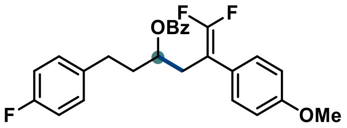

6,6-Difluoro-1-(4-fluorophenyl)-5-(4-methoxyphenyl)hex-5-en-3-yl benzoate (8). According to the **GP3**, using aldehyde **1f** (0.3 mmol, 1.5 equiv.), benzoyl bromide (0.3 mmol, 1.5 equiv.), alkene **2a** (0.2 mmol, 1.0 equiv.), 4CzIPN (10 μmol, 5 mol%), $(TMS)_3SiOH$ (0.3 mmol, 1.5 equiv.), KOAc (0.8 mmol, 4.0 equiv.) in MeCN (4 ml) and stirring at room temperature for 36 h under 30W blue LEDs irradiation. The reaction mixture was filtered through a pad of silica gel and concentrated *in vacuo*, then purified by chromatography (pentane/Et_2O = 95/5) to give **8** (63.4 mg, 0.144 mmol, 72%) as a colorless oil. **R_f** (pentane/ Et_2O = 95/5) 0.18. **^{1}H NMR** (599 MHz, $CDCl_3$) δ 7.87 (dt, *J* = 8.4, 1.6 Hz, 2H), 7.57 – 7.52 (m, 1H), 7.43 – 7.38 (m, 2H), 7.23 – 7.19 (m, 2H), 7.08 – 7.03 (m, 2H), 6.92 (td, *J* = 8.8, 1.8 Hz, 2H), 6.84 – 6.81 (m, 2H), 5.14 (t, *J* = 6.4 Hz, 1H), 3.76 (s, 3H), 2.90 – 2.84 (m, 1H), 2.74 (ddd, *J* = 14.6, 5.3, 2.4 Hz, 1H), 2.71 – 2.65 (m, 1H), 2.64 – 2.58 (m, 1H), 2.02 (dtd, *J* = 15.8, 8.2, 7.1, 4.3 Hz, 1H), 1.95 (ddd, *J* = 10.6, 7.0, 3.5 Hz, 1H). **^{13}C{^{1}H} NMR** (151 MHz, $CDCl_3$) δ 166.0, 160.5 (t, *J* = 243.1 Hz), 154.3 (dd, *J* = 289.8, 287.8 Hz), 136.8 (d, *J* = 3.3 Hz), 132.9, 130.1, 129.7, 129.6, 129.5, 129.4 (t, *J* = 3.1 Hz), 128.2, 125.1 (d, *J* = 2.2 Hz), 115.1 (d, *J* = 21.2 Hz), 114.0, 88.6 (d, *J* = 2.8 Hz), 72.4 (t, *J* = 3.0 Hz), 55.2, 35.3, 32.5, 30.8. **^{19}F NMR** (564 MHz, $CDCl_3$) δ -90.7– -90.9 (m), -117.5 – -117.6 (m). **HRMS** (ESI^+): calcd. for $C_{26}H_{23}F_3O_3Na$ (M + Na^+): 463.14915. Found: 463.14890.

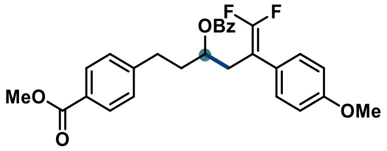

Methyl 4-(3-(benzoyloxy)-6,6-difluoro-5-(4-methoxyphenyl)hex-5-en-1-yl)benzoate (9). According to the **GP3**, using aldehyde **1g** (0.3 mmol, 1.5 equiv.), benzoyl bromide (0.3 mmol, 1.5 equiv.), alkene **2a** (0.2 mmol, 1.0 equiv.), 4CzIPN (10 μmol, 5 mol%), $(TMS)_3SiOH$ (0.3 mmol, 1.5 equiv.), KOAc (0.8 mmol, 4.0 equiv.) in MeCN (4 ml) and stirring at room temperature for 36 h under 30W blue LEDs irradiation. The reaction mixture was filtered through a pad of silica gel and concentrated *in vacuo*, then purified by chromatography (pentane/EtOAc = 10/1) to give **9** (62.5 mg, 0.130 mmol, 65%) as a yellowish solid. $\mathbf{R}_f$ (pentane/EtOAc = 10/1) 0.45. ^{1}**H NMR** (400 MHz, $CDCl_3$) δ 7.91 (d, *J* = 8.2 Hz, 1H), 7.88 – 7.82 (m, 1H), 7.54 (tt, *J* = 7.4, 1.1 Hz, 1H), 7.38 (t, *J* = 7.9 Hz, 2H), 7.21 – 7.14 (m, 4H), 6.84 – 6.80 (m, 2H), 5.14 (td, *J* = 7.1, 3.3 Hz, 1H), 3.89 (s, 3H), 3.76 (s, 3H), 2.88 (ddt, *J* = 14.7, 6.9, 2.3 Hz, 1H), 2.78 – 2.68 (m, 3H), 2.09 – 1.94 (m, 2H). ^{13}**C{^{1}H} NMR** (101 MHz, $CDCl_3$) δ 166.8, 165.8, 158.6, 154.1 (t, *J* = 289.2 Hz), 146.5, 132.7, 129.8, 129.6, 129.3, 129.2 (t, *J* = 3.1 Hz), 128.1, 128.0, 127.8, 124.9, 113.8, 88.4 (dd, *J* = 19.7, 17.4 Hz), 72.2, 55.0, 51.8, 34.6, 32.3, 31.5. ^{19}**F NMR** (377 MHz, $CDCl_3$) δ -90.6 – -90.9 (m). **HRMS** (ESI^+): calcd. for $C_{28}H_{26}O_5F_2Na$ ($M + Na^+$): 503.1641. Found: 503.1646.

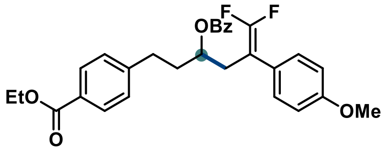

Ethyl 4-(3-(benzoyloxy)-6,6-difluoro-5-(4-methoxyphenyl)hex-5-en-1-yl)benzoate (10). According to the **GP3**, using aldehyde **1h** (0.3 mmol, 1.5 equiv.), benzoyl bromide (0.3 mmol, 1.5 equiv.), alkene **2a** (0.2 mmol, 1.0 equiv.), 4CzIPN (10 μmol, 5 mol%), $(TMS)_3SiOH$ (0.3 mmol, 1.5 equiv.), KOAc (0.8 mmol, 4.0 equiv.) in MeCN (4 ml) and stirring at room temperature for 36 h under 30W blue LEDs irradiation. The reaction mixture was filtered through a pad of silica gel and

concentrated *in vacuo*, then purified by chromatography (pentane/EtOAc = 12/1) to give **10** (59.3 mg, 0.120 mmol, 60%) as a colorless oil. **R*f*** (pentane/EtOAc = 95/5) 0.15. **^{1}H NMR** (400 MHz, $CDCl_3$) δ 7.93 – 7.90 (m, 2H), 7.87 – 7.82 (m, 2H), 7.54 (ddt, *J* = 8.7, 7.0, 1.3 Hz, 1H), 7.40 (t, *J* = 8.0 Hz, 2H), 7.23 – 7.14 (m, 4H), 6.85 – 6.79 (m, 2H), 5.14 (td, *J* = 7.3, 3.3 Hz, 1H), 4.35 (q, *J* = 7.1 Hz, 2H), 3.76 (s, 3H), 2.87 (ddt, *J* = 14.6, 6.9, 2.2 Hz, 1H), 2.79 – 2.67 (m, 3H), 2.09 – 1.94 (m, 2H), 1.38 (t, *J* = 7.1 Hz, 3H). **^{13}C{^{1}H} NMR** (101 MHz, $CDCl_3$) δ 167.0, 166.4, 159.3, 154.7 (t, *J* = 289.6 Hz), 147.0, 133.3, 130.5, 130.2, 130.0, 129.8 (t, *J* = 3.1 Hz), 128.8, 128.7, 128.7, 125.5, 114.5, 89.0 (dd, *J* = 19.7, 17.3 Hz), 72.9 (t, *J* = 3.1 Hz), 61.2, 55.6, 35.3, 33.0, 32.1, 14.8. **^{19}F{^{1}H} NMR** (377 MHz, $CDCl_3$) δ -90.7 (d, *J* = 41.7 Hz), -90.8 (d, *J* = 41.7 Hz). **HRMS** (ESI$^+$): calcd. for $C_{29}H_{28}O_5F_2Na$ (M + Na$^+$): 517.1797. Found: 517.1806.

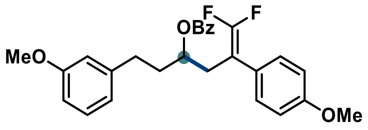

6,6-Difluoro-1-(3-methoxyphenyl)-5-(4-methoxyphenyl)hex-5-en-3-yl benzoate (11). According to the **GP3**, using aldehyde **1i** (0.3 mmol, 1.5 equiv.), benzoyl bromide (0.3 mmol, 1.5 equiv.), alkene **2a** (0.2 mmol, 1.0 equiv.), 4CzIPN (10 μmol, 5 mol%), $(TMS)_3SiOH$ (0.3 mmol, 1.5 equiv.), KOAc (0.8 mmol, 4.0 equiv.) in MeCN (4 ml) and stirring at room temperature for 36 h under 30W blue LEDs irradiation. The reaction mixture was filtered through a pad of silica gel and concentrated *in vacuo*, then purified by chromatography (pentane/Et_2O = 95/5) to give **11** (47.1 mg, 0.104 mmol, 72%) as a colorless oil. **R*f*** (pentane/ Et_2O = 95/5) 0.07. **^{1}H NMR** (500 MHz, $CDCl_3$) δ 7.93 – 7.82 (m, 2H), 7.59 – 7.52 (m, 1H), 7.45 – 7.37 (m, 2H), 7.26 – 7.21 (m, 2H), 7.18 (t, *J* = 7.9 Hz, 1H), 6.90 – 6.80 (m, 2H), 6.73 (ddd, *J* = 8.2, 3.4, 2.0 Hz, 2H), 6.68 (t, *J* = 2.1 Hz, 1H), 5.25 – 5.10 (m, 1H), 3.78 (s, 6H), 2.89 (ddt, *J* = 14.6, 7.0, 2.2 Hz, 1H), 2.81 – 2.59 (m, 3H), 2.11 – 1.95 (m, 2H). **^{13}C{^{1}H} NMR** (126 MHz, $CDCl_3$) δ 166.0, 159.7, 158.8, 154.3 (dd, *J* = 289.7, 287.8 Hz), 142.9, 132.8, 130.2, 129.5, 129.4 (t, *J* = 3.1 Hz), 128.2, 125.2 (t, *J* = 2.7 Hz),

120.7, 114.0, 114.0, 111.4, 88.7 (dd, *J* = 20.6, 16.3 Hz), 72.6 (t, *J* = 3.0 Hz), 55.1 (d, *J* = 9.0 Hz), 35.2, 32.5 (d, *J* = 1.7 Hz), 31.7. **^{19}F NMR** (470 MHz, $CDCl_3$) δ -90.8 (dt, *J* = 41.8, 2.6 Hz), -90.9 (d, *J* = 41.7 Hz). **HRMS** (ESI$^+$): calcd. for $C_{27}H_{26}F_2O_4Na$ (M + Na$^+$): 475.16914. Found: 475.16890.

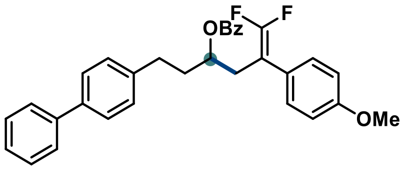

1-([1,1'-Biphenyl]-4-yl)-6,6-difluoro-5-(4-methoxyphenyl)hex-5-en-3-yl benzoate (12). According to the **GP3**, using aldehyde **1j** (0.3 mmol, 1.5 equiv.), benzoyl bromide (0.3 mmol, 1.5 equiv.), alkene **2a** (0.2 mmol, 1.0 equiv.), 4CzIPN (10 μmol, 5 mol%), $(TMS)_3SiOH$ (0.3 mmol, 1.5 equiv.), KOAc (0.8 mmol, 4.0 equiv.) in MeCN (4 ml) and stirring at room temperature for 36 h under 30W blue LEDs irradiation. The reaction mixture was filtered through a pad of silica gel and concentrated *in vacuo*, then purified by chromatography (pentane/EtOAc = 15/1) to give **12** (60.8 mg, 0.122 mmol, 61%) as a white solid. **R*f*** (pentane/EtOAc = 15/1) 0.48. **^{1}H NMR** (400 MHz, $CDCl_3$) δ 7.78 – 7.71 (m, 2H), 7.44 – 7.38 (m, 3H), 7.36 – 7.33 (m, 2H), 7.31 – 7.24 (m, 9H), 7.20 (tt, *J* = 7.3, 1.1 Hz, 1H), 7.12 – 7.09 (m, 1H), 7.09 – 7.04 (m, 3H), 6.71 – 6.67 (m, 2H), 3.60 (s, 3H), 2.76 (ddt, *J* = 14.6, 6.9, 2.2 Hz, 1H), 2.66 – 2.53 (m, 3H), 2.00 – 1.84 (m, 2H). **^{13}C{^{1}H} NMR** (101 MHz, $CDCl_3$) δ 165.8, 158.6, 154.1 (t, *J* = 288.6 Hz), 140.8, 140.2, 138.7, 132.7, 129.9, 129.8, 129.3, 129.2 (t, *J* = 3.1 Hz), 128.5, 128.5, 128.0, 127.0, 126.9, 126.8, 125.0, 113.8, 88.5 (dd, *J* = 20.2, 16.8 Hz), 72.4 (t, *J* = 3.1 Hz), 55.0, 35.0, 32.3, 31.1. **^{19}F{^{1}H} NMR** (377 MHz, $CDCl_3$) δ -90.7 (d, *J* = 42.1 Hz), -90.8 (d, *J* = 42.1 Hz). **HRMS** (ESI$^+$): calcd. for $C_{32}H_{28}O_3F_2Na$ (M + Na$^+$): 521.1899. Found: 521.1902.

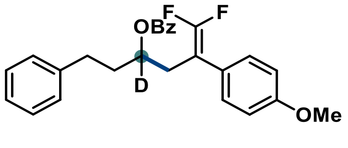

6,6-Difluoro-5-(4-methoxyphenyl)-1-phenylhex-5-en-3-yl-3-*d* benzoate (13). According to the **GP3**, using aldehyde **1k** (0.3 mmol, 1.5 equiv.), benzoyl bromide (0.3 mmol, 1.5 equiv.), alkene **2a** (0.2 mmol, 1.0 equiv.), 4CzIPN (10 μmol, 5 mol%), $(TMS)_3SiOH$ (0.3 mmol, 1.5 equiv.), KOAc (0.8 mmol, 4.0 equiv.) in MeCN (4 ml) and stirring at room temperature for 36 h under 30W blue LEDs irradiation. The reaction mixture was filtered through a pad of silica gel and concentrated *in vacuo*, then purified by chromatography (pentane/Et_2O = 98/2 → 92/8) to give **13** (48.5 mg, 0.114 mmol, 57%, d_1> 95%) as a colorless oil. $\mathbf{R_f}$ (pentane/EtOAc = 98/2): 0.35. **^{1}H NMR** (500 MHz, $CDCl_3$) δ 7.90 – 7.86 (m, 2H), 7.55 (tt, *J* = 7.5, 1.2 Hz, 1H), 7.43 – 7.37 (m, 2H), 7.28 – 7.20 (m, 4H), 7.17 (tt, *J* = 7.4, 1.4 Hz, 1H), 7.15 – 7.11 (m, 2H), 6.85 – 6.81 (m, 2H), 3.77 (s, 3H), 2.88 (d, *J* = 14.6 Hz, 1H), 2.75 (d, *J* = 14.6 Hz, 1H), 2.73 – 2.61 (m, 2H), 2.10 – 1.94 (m, 2H). **^{13}C{^{1}H, ^{19}F} NMR** (126 MHz, $CDCl_3$) δ 166.0, 158.8, 154.3, 141.3, 132.8, 130.2, 129.6, 129.4, 128.5, 128.3, 128.2, 126.0, 125.3, 114.0, 88.7, 72.3, 55.2, 35.2, 32.4, 31.6. **^{19}F{^{1}H} NMR** (470 MHz, $CDCl_3$) δ -90.8 (d, *J* =41.8 Hz), -90.9 (d, *J* =41.8 Hz). **HRMS** (ESI$^+$): calcd. for $C_{26}H_{23}DF_2NaO_3Na$ (M + Na^+): 446.1654. Found: 446.1647.

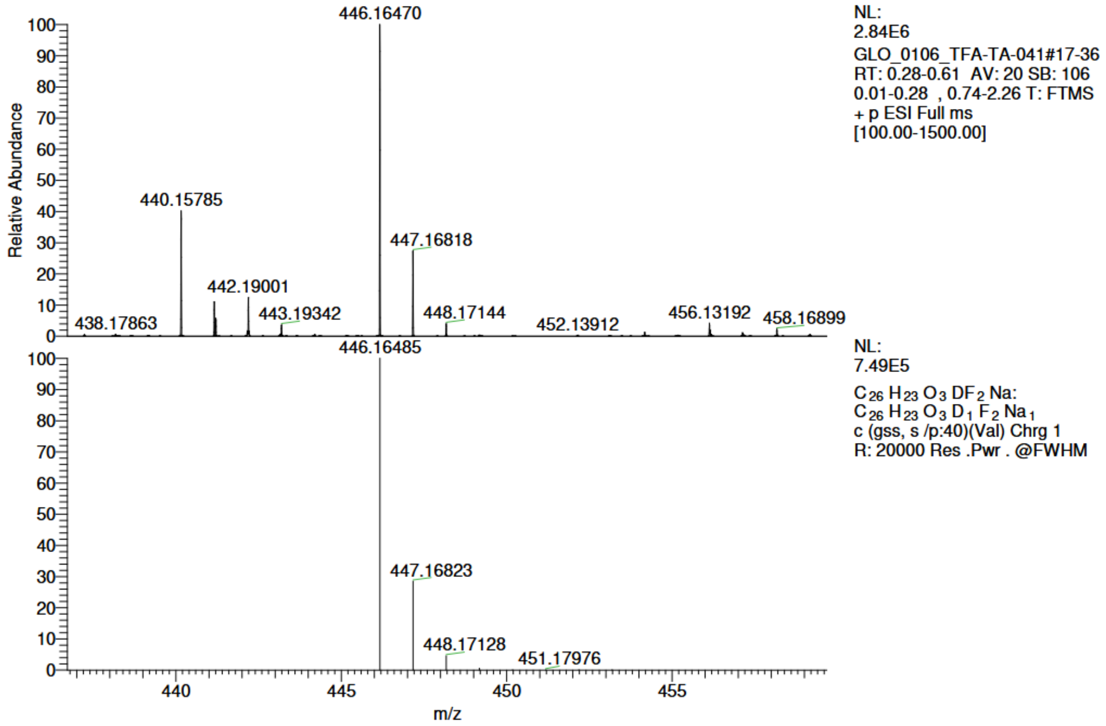

Supplementary Figure 3. High-resolution mass spectrum of product **13**.

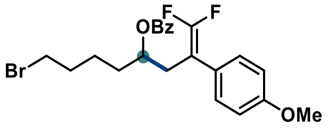

8-Bromo-1,1-difluoro-2-(4-methoxyphenyl)oct-1-en-4-yl benzoate (14). According to the **GP3**, using aldehyde **1l** (0.3 mmol, 1.5 equiv.), benzoyl bromide (0.3 mmol, 1.5 equiv.), alkene **2a** (0.2 mmol, 1.0 equiv.), 4CzIPN (10 μmol, 5 mol%), $(TMS)_3SiOH$ (0.3 mmol, 1.5 equiv.), KOAc (0.8 mmol, 4.0 equiv.) in MeCN (4 ml) and stirring at room temperature for 36 h under 30W blue LEDs irradiation. The reaction mixture was filtered through a pad of silica gel and concentrated *in vacuo*, then purified by chromatography (pentane/Et_2O = 95/5) to give **14** (59.2 mg, 0.131 mmol, 66%) as a colorless oil. **R_f** (pentane/ Et_2O = 95/5) 0.09. **^{1}H NMR** (500 MHz, $CDCl_3$) δ 7.91 – 7.85 (m, 2H), 7.58 – 7.52 (m, 1H), 7.44 – 7.37 (m, 2H), 7.27 – 7.22 (m, 2H), 6.85 (d, *J* = 8.9 Hz, 2H), 5.19 – 5.08 (m, 1H), 3.77 (s, 3H), 3.36 (t, *J* = 6.7 Hz, 2H), 2.90 – 2.80 (m, 1H), 2.75 – 2.67 (m, 1H), 1.90 – 1.79 (m, 2H), 1.77 – 1.65 (m, 2H), 1.59 – 1.47 (m, 2H). **^{13}C{^{1}H} NMR** (126 MHz, $CDCl_3$) δ 166.9, 158.8, 154.3 (dd, *J* = 290.5, 287.7 Hz), 132.9, 130.1, 129.5, 129.4 (t, *J* = 3.1 Hz), 128.2, 125.2 (d, *J* = 2.3 Hz), 114.0, 88.7 (dd, *J* = 20.8, 16.1 Hz), 72.6 (t, *J* = 2.9 Hz), 55.2, 33.3, 32.7, 32.6, 32.4, 23.7. **^{19}F NMR** (470 MHz, $CDCl_3$) δ -90.7- -91.0 (m). **HRMS** (ESI^+): calcd. for $C_{22}H_{23}BrF_2O_3Na$ (M + Na^+): 475.06908. Found: 475.06893.

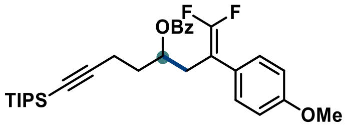

1,1-Difluoro-2-(4-methoxyphenyl)-8-(tri*iso*propylsilyl)oct-1-en-7-yn-4-yl benzoate (15). According to the **GP3**, using aldehyde **1m** (0.3 mmol, 1.5 equiv.), benzoyl bromide (0.3 mmol, 1.5 equiv.), alkene **2a** (0.2 mmol, 1.0 equiv.), 4Cz-IPN (10 μmol, 5 mol%), $(TMS)_3SiOH$ (0.3 mmol, 1.5 equiv.), KOAc (0.8 mmol, 4.0 equiv.) in MeCN (4 ml) and stirring at room temperature for 36 h under 30W blue LEDs irradiation. The reaction mixture was filtered through a pad of silica gel and concentrated *in vacuo*, then purified by chromatography (pentane/Et_2O = 95/5) to give **15** (74.2 mg, 0.141 mmol, 71%) as a colorless oil. **R_f** (pentane/ Et_2O = 95/5) 0.15. **^{1}H NMR** (500 MHz, $CDCl_3$) δ 7.92 – 7.85 (m, 2H), 7.59 – 7.52 (m, 1H), 7.40 (dd, *J* = 8.2, 7.4 Hz, 2H), 7.30 – 7.22 (m, 2H), 6.87 – 6.79 (m, 2H), 5.25 (tt, *J* = 7.8,

5.2 Hz, 1H), 3.77 (s, 3H), 2.91 (ddt, *J* = 14.6, 7.1, 2.1 Hz, 1H), 2.76 (ddt, *J* = 14.6, 5.3, 2.4 Hz, 1H), 2.41 – 2.31 (m, 2H), 2.07 – 1.89 (m, 2H), 1.04 (s, 6H), 1.03 (s, 9H). **^{13}C{^{1}H} NMR** (126 MHz, $CDCl_3$) δ 165.8, 158.8, 154.3 (dd, *J* = 290.5, 287.7 Hz), 132.8, 130.1, 129.5, 129.3 (t, *J* = 3.2 Hz), 128.2, 125.2 (d, *J* = 2.5 Hz), 114.0, 107.3, 88.6 (dd, *J* = 20.4, 16.6 Hz), 81.0, 72.0 (t, *J* = 3.0 Hz), 55.1, 33.2, 32.5, 18.6, 16.2, 11.2. **^{19}F NMR** (470 MHz, $CDCl_3$) δ -90.7 (dt, *J* = 41.6, 2.6 Hz), -90.8 (d, *J* = 41.5 Hz). **HRMS** (ESI$^+$): calcd. for $C_{31}H_{40}SiF_2O_3Na$ (M + Na$^+$): 549.26070. Found: 549.26039.

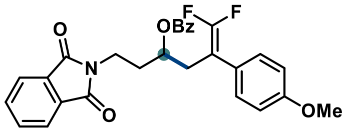

1-(1,3-Dioxoisoindolin-2-yl)-6,6-difluoro-5-(4-methoxyphenyl)hex-5-en-3-yl benzoate (16). According to the **GP3**, using aldehyde **1n** (0.3 mmol, 1.5 equiv.), benzoyl bromide (0.3 mmol, 1.5 equiv.), alkene **2a** (0.2 mmol, 1.0 equiv.), 4CzIPN (10 μmol, 5 mol%), $(TMS)_3SiOH$ (0.3 mmol, 1.5 equiv.), KOAc (0.8 mmol, 4.0 equiv.) in MeCN (4 ml) and stirring at room temperature for 36 h under 30W blue LEDs irradiation. The reaction mixture was filtered through a pad of silica gel and concentrated *in vacuo*, then purified by chromatography (pentane/EtOAc = 4/1) to give **16** (65.7 mg, 0.130 mmol, 65%) as a colorless oil. **R*f*** (pentane/EtOAc = 4/1) 0.11. **^{1}H NMR** (500 MHz, $CDCl_3$) δ 7.83 – 7.79 (m, 2H), 7.74 (dd, *J* = 5.4, 3.0 Hz, 2H), 7.64 (dd, *J* = 5.5, 3.0 Hz, 2H), 7.51 – 7.46 (m, 1H), 7.34 – 7.28 (m, 2H), 7.24 – 7.19 (m, 2H), 6.79 – 6.74 (m, 2H), 5.19 – 5.05 (m, 1H), 3.76 (q, *J* = 7.1 Hz, 2H), 3.70 (s, 3H), 2.92 (dd, *J* = 14.6, 6.4 Hz, 1H), 2.76 (dd, *J* = 14.6, 6.4 Hz, 1H), 2.10 (tdd, *J* = 7.2, 5.8, 1.6 Hz, 2H). **^{13}C{^{1}H} NMR** (126 MHz, $CDCl_3$) δ 168.1, 165.9, 158.8, 154.3 (dd, *J* = 290.5, 287.7 Hz), 133.8, 132.8, 132.0, 129.9, 129.5, 129.4 (t, *J* = 3.2 Hz), 128.1, 124.8 (t, *J* = 3.3 Hz), 123.1, 114.0, 88.4 (dd, *J* = 20.8, 16.1 Hz), 71.0, 55.1, 34.5, 32.1, 31.8. **^{19}F NMR** (470 MHz, $CDCl_3$) δ -90.4 (dt, *J* = 41.3, 2.7 Hz), -90.6 (d, *J* = 41.3 Hz). **HRMS** (ESI$^+$): calcd. for $C_{28}H_{23}NF_2O_5Na$ (M + Na$^+$): 514.14365. Found: 514.14334.

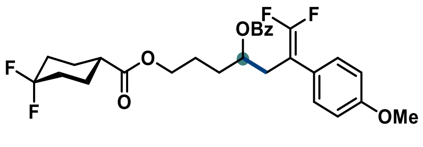

7-((4,4-Difluorocyclohexane-1-carbonyl)oxy)-1,1-difluoro-2-(4-methoxyphenyl)hept-1-en-4-yl benzoate (17). According to the **GP3**, using aldehyde **1o** (0.3 mmol, 1.5 equiv.), benzoyl bromide (0.3 mmol, 1.5 equiv.), alkene **2a** (0.2 mmol, 1.0 equiv.), 4CzIPN (10 µmol, 5 mol%), $(TMS)_3SiOH$ (0.3 mmol, 1.5 equiv.), KOAc (0.8 mmol, 4.0 equiv.) in MeCN (4 ml) and stirring at room temperature for 36 h under 30W blue LEDs irradiation. The reaction mixture was filtered through a pad of silica gel and concentrated *in vacuo*, then purified by chromatography (pentane/Et_2O = 90/10 → 82/8) to give **17** (48.5 mg, 0.093 mmol, 46%) as a colorless oil. **R_f** (pentane/EtOAc = 98/2): 0.35. **^{1}H NMR** (400 MHz, $CDCl_3$) δ 7.88 – 7.83 (m, 2H), 7.58 – 7.51 (m, 1H), 7.42 – 7.37 (m, 3H), 7.24 – 7.19 (m, 2H), 7.14 (dd, *J* = 8.8, 2.5 Hz, 1H), 6.86 – 6.81 (m, 2H), 6.75 (d, *J* = 8.8 Hz, 1H), 5.17 – 5.11 (m, 1H), 4.64 (s, 2H), 4.17 (q, *J* = 4.0 Hz, 2H), 3.75 (s, 3H), 2.83 (ddt, *J* = 14.6, 7.3, 2.1 Hz, 1H), 2.68 (ddt, *J* = 14.7, 5.2, 2.4 Hz, 1H), 1.79 – 1.64 (m, 4H). **^{13}C{^{1}H} NMR** (151 MHz, $CDCl_3$) δ 168.2, 166.1, 154.4 (t, *J* = 287.9 Hz), 152.5, 133.1 (t, *J* = 288.1 Hz), 130.5, 130.1, 129.7, 129.5, 128.4, 127.7, 127.2, 125.2 (d, *J* = 1.2 Hz), 124.4, 114.9, 114.2, 89.0 – 88.2 (m), 72.4, 66.5, 65.2, 55.3, 32.8, 30.3, 24.5. **^{19}F NMR** (377 MHz, $CDCl_3$) δ -90.7 (br s).* **HRMS** (ESI$^+$): calcd. for $C_{28}H_{30}F_4NaO_5Na$ (M + Na$^+$): 545.1927. Found: 545.1922. **The overlap of the expected two doublets results in a broad singlet.*

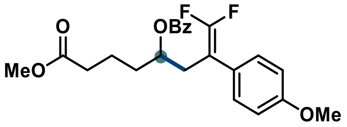

1,1-Difluoro-8-methoxy-2-(4-methoxyphenyl)-8-oxooct-1-en-4-yl benzoate (18). According to the **GP3**, using aldehyde **1p** (0.3 mmol, 1.5 equiv.), benzoyl bromide (0.3 mmol, 1.5 equiv.), alkene **2a** (0.2 mmol, 1.0 equiv.), 4CzIPN (10 µmol, 5 mol%), $(TMS)_3SiOH$ (0.3 mmol, 1.5 equiv.), KOAc (0.8 mmol, 4.0 equiv.) in MeCN (4 ml) and stirring at room temperature for 36 h under 30W blue LEDs irradiation. The reaction mixture was filtered through a pad of silica gel and concentrated *in vacuo*, then purified by chromatography (pentane/EtOAc = 9/1) to give **18** (48.4 mg, 0.116 mmol, 58%) as a colorless oil. **R_f** (pentane/EtOAc = 9/1) 0.12. **^{1}H NMR** (500 MHz, $CDCl_3$) δ 7.93 – 7.79 (m, 2H), 7.59 – 7.51 (m, 1H), 7.45 – 7.36 (m, 2H), 7.27 – 7.17 (m, 2H), 6.91 – 6.69 (m, 2H), 5.13 (ddt, *J* = 10.1, 7.2, 3.8 Hz, 1H), 3.77

(s, 3H), 3.65 (s, 3H), 2.86 (ddt, *J* = 14.6, 7.3, 2.1 Hz, 1H), 2.71 (ddt, *J* = 14.6, 5.1, 2.4 Hz, 1H), 2.31 (ddd, *J* = 6.6, 5.3, 3.0 Hz, 2H), 1.79 – 1.61 (m, 4H). **^{13}C{^{1}H} NMR** (126 MHz, $CDCl_3$) δ 173.6, 166.0, 158.8, 154.3 (dd, *J* = 290.5, 287.7 Hz), 132.8, 130.1, 129.5, 129.4 (t, *J* = 3.2 Hz), 128.2, 125.2 (d, *J* = 1.9 Hz), 114.0, 88.7 (dd, *J* = 20.8, 16.1 Hz), 72.4 (t, *J* = 2.9 Hz), 55.2, 51.5, 33.6, 33.0, 32.6, 20.6. **^{19}F NMR** (470 MHz, $CDCl_3$) δ -90.8 - -90.9 (m). **HRMS** (ESI$^+$): calcd. for $C_{23}H_{24}F_2O_5Na$ (M + Na$^+$): 441.14840. Found: 441.14797.

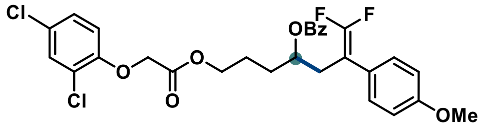

7-(2-(2,4-Dichlorophenoxy)acetoxy)-1,1-difluoro-2-(4-methoxyphenyl)hept-1-en-4-yl benzoate (19). According to the **GP3**, using aldehyde **1q** (0.3 mmol, 1.5 equiv.), benzoyl bromide (0.3 mmol, 1.5 equiv.), alkene **2a** (0.2 mmol, 1.0 equiv.), 4CzIPN (10 μmol, 5 mol%), $(TMS)_3SiOH$ (0.3 mmol, 1.5 equiv.), KOAc (0.8 mmol, 4.0 equiv.) in MeCN (4 ml) and stirring at room temperature for 36 h under 30W blue LEDs irradiation. The reaction mixture was filtered through a pad of silica gel and concentrated *in vacuo*, then purified by chromatography (pentane/Et_2O = 9/1 → 75/25) to give **19** (62.6 mg, 0.108 mmol, 54%) as a colorless oil. **R*f*** (pentane/ Et_2O = 7/3) 0.30. **^{1}H NMR** (599 MHz, $CDCl_3$) δ 7.88 – 7.83 (m, 2H), 7.54 (tt, *J* = 7.5, 1.2 Hz, 1H), 7.42 – 7.37 (m, 3H), 7.24 – 7.19 (m, 2H), 7.14 (dd, *J* = 8.8, 2.5 Hz, 1H), 6.86 – 6.81 (m, 2H), 6.75 (d, *J* = 8.8 Hz, 1H), 5.17 – 5.11 (m, 1H), 4.64 (s, 2H), 4.20 – 4.13 (m, 2H), 3.75 (s, 3H), 2.83 (ddt, *J* = 14.6, 7.3, 2.1 Hz, 1H), 2.68 (ddt, *J* = 14.7, 5.2, 2.4 Hz, 1H), 1.79 – 1.64 (m, 4H). **^{13}C{^{1}H} NMR** (151 MHz, $CDCl_3$) δ 168.2, 166.1, 154.4 (t, *J* = 287.9 Hz), 152.5, 133.1, 130.5, 130.1, 129.6, 129.5 (t, *J* = 3.0 Hz), 128.5, 128.4, 127.7, 127.2, 125.2 (d, *J* = 1.2 Hz), 124.4, 114.9, 114.2, 88.7 (t, *J* = 18.9 Hz), 72.3 (t, *J* = 3.0 Hz), 66.4, 65.2, 55.3, 32.8, 30.3, 24.5. **^{19}F NMR** (564 MHz, $CDCl_3$) δ -90.6 – -90.7 (m). **HRMS** (ESI$^+$): calcd. for $C_{21}H_{24}NO_2F_2$ (M + H$^+$): 360.1770. Found: 360.1770.

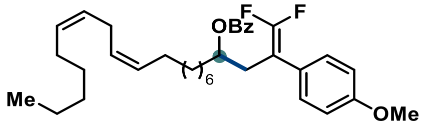

(12*Z*,15*Z*)-1,1-Difluoro-2-(4-methoxyphenyl)henicosa-1,12,15-trien-4-yl benzoate (20). According to the **GP3**, using aldehyde **1r** (0.3 mmol, 1.5 equiv.), benzoyl bromide (0.3 mmol, 1.5 equiv.), alkene **2a** (0.2 mmol, 1.0 equiv.), 4CzIPN (10 μmol, 5 mol%), $(TMS)_3SiOH$ (0.3 mmol, 1.5 equiv.), KOAc (0.8 mmol, 4.0 equiv.) in MeCN (4 ml) and stirring at room temperature for 36 h under 30W blue LEDs irradiation. The reaction mixture was filtered through a pad of silica gel and concentrated *in vacuo*, then purified by chromatography (pentane/Et_2O = 90/10 → 82/8) to give **20** (71.6 mg, 0.130 mmol, 65%) as a colorless oil. **R*f*** (pentane/EtOAc = 98/2): 0.35. **^{1}H NMR** (599 MHz, $CDCl_3$) δ 7.90 – 7.72 (m, 2H), 7.55 – 7.49 (m, 1H), 7.41 – 7.35 (m, 2H), 7.25 – 7.19 (m, 2H), 6.85 – 6.80 (m, 2H), 5.42 – 5.28 (m, 4H), 5.10 (tt, *J* = 7.4, 5.1 Hz, 1H), 3.75 (s, 3H), 2.82 (dd, *J* = 14.6, 7.4 Hz, 1H), 2.76 (t, *J* = 6.8 Hz, 2H), 2.69 (dd, *J* = 14.6, 5.3 Hz, 1H), 2.08 – 1.92 (m, 4H), 1.74 – 1.59 (m, 2H), 1.42 – 1.19 (m, 16H), 0.88 (t, *J* = 7.0 Hz, 3H). **^{13}C{^{1}H, ^{19}F} NMR** (151 MHz, $CDCl_3$) δ 166.2, 158.9, 154.4, 132.9, 130.5, 130.4, 130.2, 129.7, 129. 6, 128.3, 128.1, 128.1, 125.6, 114.1, 89.0, 73.2, 55.3, 33.8, 32.8, 31.7, 29.8, 29.6, 29.5, 29.5, 29.3, 27.4, 25.8, 25.3, 22.7, 14.2. **^{19}F{^{1}H} NMR** (564 MHz, $CDCl_3$) δ -91.0 (d, *J* =42.4 Hz), -91.1 (d, *J* = 42.1 Hz). **HRMS** (ESI$^+$): calcd. for $C_{35}H_{46}F_2NaO_3Na$ (M + Na^+): 575.3313. Found: 575.3308. **One peak in the aromatic region was not detected, presumably due to an overlap of peaks*

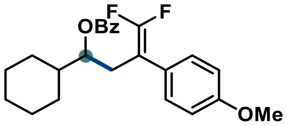

1-Cyclohexyl-4,4-difluoro-3-(4-methoxyphenyl)but-3-en-1-yl benzoate (21). According to the **GP3**, using aldehyde **1s** (0.3 mmol, 1.5 equiv.), benzoyl bromide (0.3 mmol, 1.5 equiv.), alkene **2a** (0.2 mmol, 1.0 equiv.), 4CzIPN (10 μmol, 5 mol%), $(TMS)_3SiOH$ (0.3 mmol, 1.5 equiv.), KOAc (0.8 mmol, 4.0 equiv.) in MeCN (4 ml) and stirring at room temperature for 36 h under 30W blue LEDs irradiation. The reaction mixture was filtered through a pad of silica gel and concentrated *in vacuo*, then purified by chromatography (pentane/Et_2O = 95/5) to give **21** (44.1 mg, 0.110 mmol, 55%) as a colorless oil. **R$_f$** (pentane/ Et_2O = 95/5) 0.20. **^{1}H NMR** (500 MHz, $CDCl_3$) δ 7.95 – 7.86 (m, 2H), 7.53 (d, *J* = 7.4 Hz, 1H), 7.45 – 7.37 (m, 2H),

7.24 – 7.16 (m, 2H), 6.86 – 6.78 (m, 2H), 5.02 (ddd, *J* = 9.2, 5.5, 4.4 Hz, 1H), 3.76 (s, 3H), 2.83 (dddd, *J* = 14.7, 8.9, 2.5, 1.1 Hz, 1H), 2.71 (ddt, *J* = 14.7, 4.2, 2.7 Hz, 1H), 1.82 – 1.70 (m, 4H), 1.70 – 1.61 (m, 2H), 1.27 – 1.08 (m, 5H). **^{13}C{^{1}H} NMR** (126 MHz, $CDCl_3$) δ 166.0, 158.7, 154.2 (dd, *J* = 289.9, 287.4 Hz), 132.7, 130.4, 129.5, 129.5, 128.2, 125.2 (d, *J* = 2.5 Hz), 113.9, 88.6 (dd, *J* = 21.1, 15.7 Hz), 76.4 (t, *J* = 5.7 Hz), 55.1, 41.4, 30.2, 29.1, 27.8, 26.3, 26.1, 26.0. **^{19}F NMR** (470 MHz, $CDCl_3$) δ -91.1 (dq, *J* = 42.6, 1.2 Hz), -91.4 (dt, *J* = 42.7, 2.6 Hz). **HRMS** (ESI$^+$): calcd. for $C_{24}H_{26}F_2O_3Na$ (M + Na$^+$): 423.17422. Found: 423.17371.

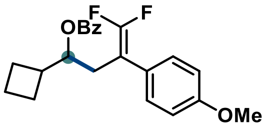

1-Cyclobutyl-4,4-difluoro-3-(4-methoxyphenyl)but-3-en-1-yl benzoate (22). According to the **GP3**, using aldehyde **1t** (0.3 mmol, 1.5 equiv.), benzoyl bromide (0.3 mmol, 1.5 equiv.), alkene **2a** (0.2 mmol, 1.0 equiv.), 4CzIPN (10 μmol, 5 mol%), $(TMS)_3SiOH$ (0.3 mmol, 1.5 equiv.), KOAc (0.8 mmol, 4.0 equiv.) in MeCN (4 ml) and stirring at room temperature for 36 h under 30W blue LEDs irradiation. The reaction mixture was filtered through a pad of silica gel and concentrated *in vacuo*, then purified by chromatography (pentane/Et_2O = 95/5) to give **22** (46.6 mg, 0.120 mmol, 60%) as a colorless oil. **R_f** (pentane/ Et_2O = 95/5) 0.22. **^{1}H NMR** (599 MHz, $CDCl_3$) δ 7.95 – 7.83 (m, 2H), 7.59 – 7.49 (m, 1H), 7.43 – 7.32 (m, 2H), 7.23 (dd, *J* = 8.9, 1.1 Hz, 2H), 6.86 – 6.76 (m, 2H), 5.12 (td, *J* = 7.2, 5.1 Hz, 1H), 3.75 (s, 3H), 2.79 – 2.68 (m, 1H), 2.68 – 2.55 (m, 2H), 1.97 (dddd, *J* = 12.2, 9.7, 6.0, 1.4 Hz, 3H), 1.90 – 1.83 (m, 1H), 1.83 – 1.67 (m, 2H). **^{13}C{^{1}H} NMR** (151 MHz, $CDCl_3$) δ 166.2, 158.7, 154.2 (dd, *J* = 289.9, 287.4 Hz), 132.7, 130.4, 129.5, 129.4, 128.2, 125.2 (d, *J* = 2.5 Hz), 113.9, 88.6 (dd, *J* = 21.1, 15.7 Hz), 75.5 (t, *J* = 2.9 Hz), 55.1, 38.6, 30.5, 24.4, 24.0, 18.0. **^{19}F NMR** (564 MHz, $CDCl_3$) δ -91.1 (d, *J* = 42.1 Hz), -91.2 (dt, *J* = 42.1, 2.6 Hz). **HRMS** (ESI$^+$): calcd. for $C_{22}H_{22}F_2O_3Na$ (M + Na$^+$): 395.14292. Found: 395.14294.

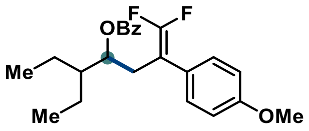

5-Ethyl-1,1-difluoro-2-(4-methoxyphenyl)hept-1-en-4-yl benzoate (23). According to the **GP3**, using aldehyde **1u** (0.3 mmol, 1.5 equiv.), benzoyl bromide (0.3 mmol, 1.5 equiv.), alkene **2a** (0.2 mmol, 1.0 equiv.), 4CzIPN (10 µmol, 5 mol%), $(TMS)_3SiOH$ (0.3 mmol, 1.5 equiv.), KOAc (0.8 mmol, 4.0 equiv.) in MeCN (4 ml) and stirring at room temperature for 36 h under 30W blue LEDs irradiation. The reaction mixture was filtered through a pad of silica gel and concentrated *in vacuo*, then purified by chromatography (pentane/Et_2O = 95/5) to give **23** (49.2 mg, 0.127 mmol, 63%) as a colorless oil. **R_f** (pentane/ Et_2O = 95/5) 0.25. **^{1}H NMR** (599 MHz, $CDCl_3$) δ 7.96 – 7.85 (m, 2H), 7.57 – 7.49 (m, 1H), 7.44 – 7.36 (m, 2H), 7.24 – 7.13 (m, 2H), 6.86 – 6.75 (m, 2H), 5.22 (dt, *J* = 8.6, 4.2 Hz, 1H), 3.76 (s, 3H), 2.84 (dddd, *J* = 14.7, 8.9, 2.6, 1.6 Hz, 1H), 2.67 (ddt, *J* = 14.6, 4.7, 2.4 Hz, 1H), 1.55 – 1.47 (m, 2H), 1.46 – 1.34 (m, 3H), 0.92 (d, *J* = 7.3 Hz, 3H), 0.85 (t, *J* = 7.4 Hz, 3H). **^{13}C{^{1}H} NMR** (151 MHz, $CDCl_3$) δ 165.9, 158.8, 154.1 (dd, *J* = 289.8, 287.2 Hz), 132.7, 130.4, 129.51, 129.5 (t, *J* = 3.1 Hz), 128.2, 125.2 (d, *J* = 2.5 Hz), 113.9, 89.2 (dd, *J* = 21.2, 15.6 Hz), 74.2 (t, *J* = 2.7 Hz), 55.1, 44.3, 29.7, 22.3, 22.0, 11.8, 11.7. **^{19}F NMR** (564 MHz, $CDCl_3$) δ -91.3 (d, *J* = 42.9 Hz), -91.6 (dt, *J* = 42.8, 2.7 Hz) **HRMS** (ESI^+): calcd. for $C_{23}H_{26}F_2O_3Na$ (M + Na^+): 411.17422. Found: 411.17396.

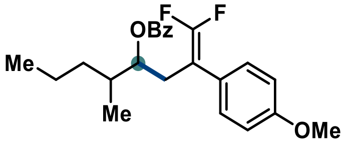

1,1-Difluoro-2-(4-methoxyphenyl)-5-methyloct-1-en-4-yl benzoate (24). According to the **GP3**, using aldehyde **1v** (0.3 mmol, 1.5 equiv.), benzoyl bromide (0.3 mmol, 1.5 equiv.), alkene **2a** (0.2 mmol, 1.0 equiv.), 4CzIPN (10 µmol, 5 mol%), $(TMS)_3SiOH$ (0.3 mmol, 1.5 equiv.), KOAc (0.8 mmol, 4.0 equiv.) in MeCN (4 ml) and stirring at room temperature for 36 h under 30W blue LEDs irradiation. The reaction mixture was filtered through a pad of silica gel and concentrated *in vacuo*, then purified by chromatography (pentane/Et_2O = 95/5) to give **24** (46.6

mg, 0.120 mmol, 60%, 50:50 d.r.) as a colorless oil. **R$_f$** (pentane/ Et_2O = 95/5) 0.15. **^{1}H NMR** (599 MHz, $CDCl_3$) δ 7.93 (d, *J* = 7.6 Hz, 2H), 7.90 (d, *J* = 7.7 Hz, 2H), 7.56 – 7.50 (m, 2H), 7.40 (q, *J* = 7.3 Hz, 4H), 7.25 – 7.22 (m, 2H), 7.19 (d, *J* = 8.2 Hz, 2H), 6.82 (ddd, *J* = 14.9, 9.2, 2.2 Hz, 4H), 5.07 (ddd, *J* = 19.4, 9.2, 4.9 Hz, 2H), 3.76 (d, *J* = 1.0 Hz, 3H), 3.75 (s, 3H), 2.88 – 2.78 (m, 2H), 2.73 – 2.61 (m, 2H), 1.91 – 1.83 (m, 1H), 1.78 (s, 1H), 1.46 – 1.40 (m, 2H), 1.33 (ddd, *J* = 27.6, 13.2, 6.4 Hz, 2H), 1.27 – 1.13 (m, 4H), 1.01 (d, *J* = 6.8 Hz, 3H), 0.95 (d, *J* = 6.8 Hz, 3H), 0.89 (s, 4H), 0.82 (s, 3H). **^{13}C{^{1}H} NMR** (126 MHz, $CDCl_3$) δ 166.0, 166.0, 158.8, 158.8, 154.1(dd, *J* = 289.8, 287.2 Hz), 154.1(dd, *J* = 289.8, 287.2 Hz), 132.7, 132.7, 130.4, 130.4, 129.5, 129.5, 129.5, 129.5, 128.2, 128.2, 125.2 (d, *J* = 2.5 Hz), 125.2 (d, *J* = 2.5 Hz), 113.9, 113.9, 89.2 (dd, *J* = 21.2, 15.6 Hz), 89.2 (dd, *J* = 21.2, 15.6 Hz), 76.4 (t, *J* = 2.7 Hz), 75.6 (t, *J* = 2.7 Hz), 55.2, 55.2, 35.9, 35.7, 35.3, 34.2, 30.1, 29.2, 20.2, 20.2, 15.1, 14.2, 14.2, 14.1. **^{19}F NMR** (470 MHz, $CDCl_3$) δ -91.1 – -91.2 (m, two diastereoisomers), -91.4 (dt, *J* = 42.7, 2.7 Hz, single diastereoisomer), -91.53 (dt, *J* = 42.8, 2.6 Hz, single diastereoisomer). **HRMS** (ESI$^+$): calcd. for $C_{23}H_{26}F_2O_3Na$ (M + Na$^+$): 411.17422. Found: 411.17395.

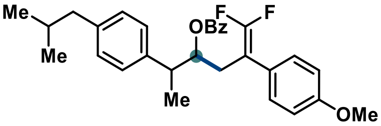

6,6-Difluoro-2-(4-*iso*butylphenyl)-5-(4-methoxyphenyl)hex-5-en-3-yl benzoate (25). According to the **GP3**, using aldehyde **1w** (0.3 mmol, 1.5 equiv.), benzoyl bromide (0.3 mmol, 1.5 equiv.), alkene **2a** (0.2 mmol, 1.0 equiv.), 4CzIPN (10 μmol, 5 mol%), $(TMS)_3SiOH$ (0.3 mmol, 1.5 equiv.), KOAc (0.8 mmol, 4.0 equiv.) in MeCN (4 ml) and stirring at room temperature for 36 h under 30W blue LEDs irradiation. The reaction mixture was filtered through a pad of silica gel and concentrated *in vacuo*, then purified by chromatography (pentane/EtOAc = 100/0 → 96/4), followed by preparative TLC (pentane/Et_2O = 92/8) to give **25** (54.3 mg, 0.113 mmol, 57%, 54:46 d.r.) as a colorless oil. **R$_f$** (pentane/EtOAc = 96/4): 0.44. *Note. Due to overlap and limited resolution of 2D NMR methods, not all the signals could be individually assigned. **M** = major isomer, **m** = minor isomer.* **^{1}H NMR** (500 MHz, $CDCl_3$) δ 7.97 – 7.91 (m, ***M*** (2H)), 7.86 – 7.82 (m, ***m*** (2H)), 7.58 – 7.48 (m, ***M*** (1H) and ***m*** (1H)), 7.45 – 7.35 (m, ***M*** (2H) and ***m*** (2H)), 7.18 (d, *J* = 8.0 Hz, ***m*** (2H)),

7.10 – 7.00 (m, ***M*** (6H) and ***m*** (4H)), 6.80 – 6.71 (m, ***M*** (2H) and ***m*** (2H)), 5.33 – 5.25 (m, ***M*** (1H) and ***m*** (1H)), 3.75 (s, ***M*** (3H)), 3.73 (s, ***m*** (3H)), 3.20 – 3.10 (m, ***m*** (1H)), 3.06 – 2.97 (m, ***M*** (1H)), 2.75 – 2.62 (m, ***M*** (1H) and ***m*** (1H)), 2.59 – 2.50 (m, ***M*** (1H) and ***m*** (1H)), 2.49 – 2.42 (m, ***M*** (2H) and **m** (2H)), 1.96 – 1.76 (m, ***M*** (1H) and **m** (1H)), 1.33 (d, *J* = 7.1 Hz, ***m*** (3H)), 1.28 (d, *J* = 7.0 Hz, ***M*** (3H)), 0.95 – 0.85 (m, ***M*** (6H) and ***m*** (6H)). **^{13}C{^{1}H, ^{19}F} NMR** (151 MHz, $CDCl_3$) δ 166.1 (***M***), 166.0 (***m***), 158.8 (***M and m***), 154.2 (***M***), 154.1 (***m***), 140.34 (***M***), 140.32 (***M***), 140.2 (***m***), 139.2 (***m***), 132.9 (***M***), 132.8 (***m***), 130.40 (***M***), 130.38 (***m***), 129.7 (***M***), 129.6 (***m***), 129.54 (***M***), 129.50 (***m***), 129.4 (***M***), 129.2 (***m***), 128.4 (***M***), 128.3 (***m***), 128.1 (***m***), 127.6 (***M***), 125.33 (***m***), 125.28 (***M***), 114.0 (***m***), 113.9 (***M***), 89.1 (***M***), 89.0 (***m***), 76.8 (***M***), 76.5 (***m***), 55.29 (***M***), 55.28 (***m***), 45.17 (***m***), 45.16 (***M***), 43.9 (***M***), 42.7 (***m***), 31.3 (***M***), 30.4 (***M and m***), 30.1 (***m***), 22.52 (***M*** and/*or* ***m***), 22.47 (***M*** and/*or* ***m***), 17.9 (***M***), 16.4 (***m***). **^{19}F NMR** (470 MHz, $CDCl_3$) δ -90.9 (dd, *J* = 42.0, 2.7 Hz, ***m***), -91.07 (dt, *J* = 41.9, 2.5 Hz, ***m***), -91.13 (dd, *J* =42.3, 1.8 Hz, ***M***), -91.3 (dt, *J* = 42.1, 2.6 Hz, ***M***). **HRMS** (ESI$^+$): calcd. for $C_{30}H_{32}F_2NaO_3Na$ (M + Na$^+$): 501.2217. Found: 501.2211.

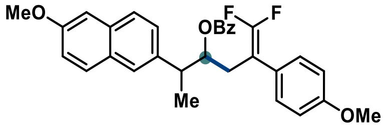

6,6-Difluoro-2-(7-methoxynaphthalen-2-yl)-5-(4-methoxyphenyl)hex-5-en-3-yl benzoate (26). According to the **GP3**, using aldehyde **1x** (0.3 mmol, 1.5 equiv.), benzoyl bromide (0.3 mmol, 1.5 equiv.), alkene **2a** (0.2 mmol, 1.0 equiv.), 4CzIPN (10 μmol, 5 mol%), $(TMS)_3SiOH$ (0.3 mmol, 1.5 equiv.), KOAc (0.8 mmol, 4.0 equiv.) in MeCN (4 ml) and stirring at room temperature for 36 h under 30W blue LEDs irradiation. The reaction mixture was filtered through a pad of silica gel and concentrated *in vacuo*, then purified by chromatography (pentane/EtOAc = 100/0 → 96/4), followed by preparative TLC (pentane/EtOAc = 93/7) to give **26** (31.3 mg, 0.062 mmol, 31%, 57:43 d.r.) as a colorless oil. **R*f*** (pentane/EtOAc = 9:1): 0.30. *Note. Due to overlap and limited resolution of 2D NMR methods, not all the signals could be individually assigned.* ***M*** *= major isomer,* ***m*** *= minor isomer.* **^{1}H NMR** (599 MHz, $CDCl_3$) δ 8.00 – 7.94 (m, ***M*** (1H), 7.92 – 7.87 (m, ***m*** (1H)), 7.75 – 7.67 (m, 3H), 7.67 – 7.61 (m, 2H), 7.60 – 7.50 (m, 3H), 7.47 – 7.38 (m, 5H), 7.28 (dd, J = 8.4, 1.8 Hz, 1H), 7.18 – 7.12 (m, 4H), 7.06 – 7.00 (m, 4H), 6.78 – 6.69 (m, 2H), 5.42 (ddt,

J = 11.8, 8.5, 3.8 Hz, 1H), 3.93 (s, 3H), 3.75 (s, ***M*** (3H)) and 3.73 (s, ***m*** (3H)), 3.37 – 3.27 (m, ***m*** (1H)), 3.19 (p, *J* = 7.2 Hz, ***M*** (1H)), 2.77 – 2.65 (m, 2H), 2.64 – 2.55 (m, 2H), 1.42 (d, *J* = 7.1 Hz, ***m** (3 H)*), 1.37 (d, *J* = 7.0 Hz, 2H, ***M*** (3H)). **^{13}C{^{19}F} NMR** (151 MHz, $CDCl_3$) δ 166.2 (***M***), 166.0 (***m***), 158.81 (***M***), 158.80 (***m***), 157.7 (***M***) 157.6 (***m***), 154.2 (***M***), 154.1 (***m***), 138.3 (***M***), 137.1 (***m***), 133.70 (***M***), 133.66 (***m***), 133.0 (***M***), 132.9 (***m***), 130.4 (***M***), 130.3 (***m***), 129.73 (***M***) 129.67 (***m***), 129.6 (***M***), 129.5 (***m***), 129.4, 129.3, 129.2, 129.0, 128.40, 128.36, 127.3, 126.9, 126.7, 126.4, 125.3, 119.1 (***M***), 118.9 (***m***), 113.97 (***M***), 113.95 (***m***), 105.73 (***M***), 105.70 (***m***), 89.03 (***M***) 89.00 (***m***), 76.6 (***M***) 76.3 (***m***), 55.45 (***M***) 55.44 (***m***), 55.28 (***M***) 55.26 (***m***), 44.2 (***M***) 42.9 (***m***), 31.4 (***M***) 30.3 (***m***), 18.2 (***M***) 16.6 (***m***). **^{19}F NMR** (564 MHz, $CDCl_3$) δ -90.8 (d, *J* = 41.7 Hz, ***m***), -91.1 (d, *J* =41.9 Hz, ***m***), -90.9 (d, *J* = 41.7 Hz, ***M***), -91.2 (d, *J* =41.9 Hz, ***M***). **HRMS** (ESI$^+$): calcd. for $C_{31}H_{28}F_2NaO_4Na$ (M + Na^+): 525.1848. Found: 525.1847.

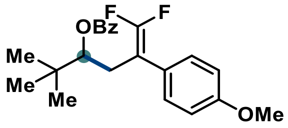

6,6-Difluoro-5-(4-methoxyphenyl)-2,2-dimethylhex-5-en-3-yl benzoate (27). According to the **GP3**, using aldehyde **1y** (0.3 mmol, 1.5 equiv.), benzoyl bromide (0.3 mmol, 1.5 equiv.), alkene **2a** (0.2 mmol, 1.0 equiv.), 4CzIPN (10 μmol, 5 mol%), $(TMS)_3SiOH$ (0.3 mmol, 1.5 equiv.), KOAc (0.8 mmol, 4.0 equiv.) in MeCN (4 ml) and stirring at room temperature for 36 h under 30W blue LEDs irradiation. The reaction mixture was filtered through a pad of silica gel and concentrated *in vacuo*, then purified by chromatography (pentane/Et_2O = 95/5) to give **27** (43.1 mg, 0.115 mmol, 58%) as a colorless oil. **R_f** (pentane/ Et_2O = 95/5) 0.23. **^{1}H NMR** (599 MHz, $CDCl_3$) δ 8.03 – 7.91 (m, 2H), 7.52 (d, *J* = 7.4 Hz, 1H), 7.40 (dd, *J* = 8.2, 7.4 Hz, 2H), 7.23 – 7.12 (m, 2H), 6.86 – 6.70 (m, 2H), 5.00 (dd, *J* = 10.4, 2.4 Hz, 1H), 3.73 (s, 3H), 2.78 – 2.64 (m, 2H), 0.99 (s, 9H). **^{13}C{^{1}H} NMR** (151 MHz, $CDCl_3$) δ 165.8, 158.7, 154.0 (dd, *J* = 289.9, 287.4 Hz), 132.6, 130.3, 129.5, 129.5, 128.1, 125.5 (d, *J* = 2.5 Hz), 113.8, 89.4 (dd, *J* = 21.3, 15.4 Hz), 79.0(d, *J* = 1.5 Hz), 55.1, 34.9, 29.1, 25.9. **^{19}F NMR** (564 MHz, $CDCl_3$) δ -91.3 (ddt, *J* = 42.8, 2.9, 1.3 Hz), -91.8 (dt, *J* = 42.7, 2.6 Hz). **HRMS** (ESI$^+$): calcd. for $C_{22}H_{24}F_2O_3Na$ (M + Na^+): 397.1586. Found: 397.1584.

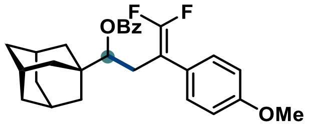

1-(Adamantan-1-yl)-4,4-difluoro-3-(4-methoxyphenyl)but-3-en-1-yl benzoate (28). According to the **GP3**, using aldehyde **1z** (0.3 mmol, 1.5 equiv.), benzoyl bromide (0.3 mmol, 1.5 equiv.), alkene **2a** (0.2 mmol, 1.0 equiv.), 4CzIPN (10 µmol, 5 mol%), $(TMS)_3SiOH$ (0.3 mmol, 1.5 equiv.), KOAc (0.8 mmol, 4.0 equiv.) in MeCN (4 ml) and stirring at room temperature for 36 h under 30W blue LEDs irradiation. The reaction mixture was filtered through a pad of silica gel and concentrated *in vacuo*, then purified by chromatography (pentane/EtOAc = 10/1) to give **28** (63.3 mg, 0.140 mmol, 70%) as a colorless oil. **R_f** (pentane/ EtOAc = 10/1) 0.45. **^{1}H NMR** (400 MHz, $CDCl_3$) δ 7.97 – 7.89 (m, 2H), 7.53 (tt, *J* = 7.5, 1.2 Hz, 1H), 7.44 – 7.37 (m, 2H), 7.19 – 7.09 (m, 2H), 6.81 – 6.74 (m, 2H), 4.84 (dd, *J* = 10.4, 2.5 Hz, 1H), 3.73 (s, 3H), 2.81 – 2.62 (m, 2H), 2.02 – 1.95 (m, 3H), 1.75 – 1.56 (m, 12H). **^{13}C{^{1}H} NMR** (101 MHz, $CDCl_3$) δ 165.7, 158.4, 153.8 (t, *J* = 289.7 Hz), 132.4, 130.1, 129.8, 129.4 (t, *J* = 3.1 Hz), 129.3, 128.0, 125.3 (t, *J* = 3.4 Hz), 113.6, 89.3 (dd, *J* = 21.3, 15.3 Hz), 79.0 (t, *J* = 2.5 Hz), 54.9, 37.9, 36.8, 36.5, 27.9, 27.3. **^{19}F{^{1}H} NMR** (377 MHz, $CDCl_3$) δ -91.1 (d, *J* = 42.0 Hz), -91.9 (d, *J* = 42.0 Hz). **HRMS** (ESI$^+$): calcd. for $C_{28}H_{30}F_2O_3Na$ (M + Na$^+$): 475.2055. Found: 475.2056.

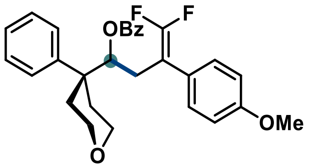

4,4-Difluoro-3-(4-methoxyphenyl)-1-(4-phenyltetrahydro-2H-pyran-4-yl)but-3-en-1-yl benzoate (29). According to the **GP3**, using aldehyde **1aa** (0.3 mmol, 1.5 equiv.), benzoyl bromide (0.3 mmol, 1.5 equiv.), alkene **2a** (0.2 mmol, 1.0 equiv.), 4CzIPN (10 µmol, 5 mol%), $(TMS)_3SiOH$ (0.3 mmol, 1.5 equiv.), KOAc (0.8 mmol, 4.0 equiv.) in MeCN (4 ml) and stirring at room temperature for 36 h under 30W blue LEDs irradiation. The reaction mixture was filtered through a pad of silica gel and concentrated *in vacuo*, then purified by chromatography

(pentane/EtOAc =100/0 → 96/4) to give **29** (50.1 mg, 0.104 mmol, 52%) as a colorless oil. **R*f*** (pentane/Et_2O = 85/15): 0.30. **^{1}H NMR** (500 MHz, $CDCl_3$) δ 7.79 (dd, *J* =8.3, 1.5 Hz, 2H), 7.42 (t, *J* =7.4 Hz, 1H), 7.34 – 7.20 (m, 8H), 6.74 – 6.61 (m, 2H), 6.59 – 6.49 (m, 2H), 5.11 (dd, *J* =8.1, 4.3 Hz, 1H), 3.72 (dt, *J* =11.7, 3.8 Hz, 1H), 3.57 (s, 2H), 3.38 (td, *J* =11.7, 2.3 Hz, 3H), 3.20 (td, *J* =11.6, 2.0 Hz, 1H), 2.25 (dt, *J* =6.7, 2.3 Hz, 2H), 2.11 (dd, *J* =13.8, 2.8 Hz, 1H), 2.05 – 1.93 (m, 2H), 1.80 (ddd, *J* =13.8, 11.5, 4.1 Hz, 1H). **^{13}C{^{1}H, ^{19}F} NMR** (126 MHz, $CDCl_3$) δ 166.0, 158.7, 154.0, 139.9, 133.1, 130.0, 129.8, 129.4, 128.9, 128.5, 128.4, 127.1, 125.0, 113.9, 89.0, 78.3, 64.2, 63.9, 55.2, 44.6, 33.6, 30.6, 28.4. **^{19}F{^{1}H} NMR** (470 MHz, $CDCl_3$) δ -90.8 (d, *J* =42.0 Hz), -91.5 (d, *J* =42.0 Hz). **HRMS** (ESI$^+$): calcd. for $C_{29}H_{28}F_2NaO_4Na$ (M + Na^+): 501.1853. Found: 501.1847. **IR** (ATR) ṽ = 2953 (w), 2929 (w), 2857 (w), 1716 (s), 1601 (w), 1514 (w), 1450 (w), 1266 (s), 1246 (s), 1176 (w), 1105 (m), 1069 (m), 1025 (m), 831 (w), 708 (s).

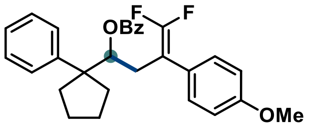

4,4-Difluoro-3-(4-methoxyphenyl)-1-(1-phenylcyclopentyl)but-3-en-1-yl benzoate (30). According to the **GP3**, using aldehyde **1ab** (0.3 mmol, 1.5 equiv.), benzoyl bromide (0.3 mmol, 1.5 equiv.), alkene **2a** (0.2 mmol, 1.0 equiv.), 4CzIPN (10 μmol, 5 mol%), $(TMS)_3SiOH$ (0.3 mmol, 1.5 equiv.), KOAc (0.8 mmol, 4.0 equiv.) in MeCN (4 ml) and stirring at room temperature for 36 h under 30W blue LEDs irradiation. The reaction mixture was filtered through a pad of silica gel and concentrated *in vacuo*, then purified by chromatography (pentane/Et_2O = 99/1 → 96/4) to give **30** (32.8 mg, 0.071 mmol, 40%) as a colorless oil. **R*f*** (pentane/Et_2O = 98:2): 0.39. **^{1}H NMR** δ 8.03 – 7.80 (m, 2H), 7.59 – 7.52 (m, 1H), 7.46 – 7.34 (m, 7H), 7.31 – 7.23 (m, 1H), 6.98 – 6.90 (m, 2H), 6.78 – 6.63 (m, 2H), 5.42 (dd, *J* = 9.8, 2.7 Hz, 1H), 3.72 (s, 3H), 2.51 – 2.33 (m, 2H), 2.18 – 1.98 (m, 3H), 1.86 (dt, *J* = 12.8, 7.6 Hz, 1H), 1.80 – 1.69 (m, 1H), 1.68 – 1.46 (m, 3H). **^{13}C{^{1}H} NMR** (101 MHz, $CDCl_3$ δ 166.1, 158. 7, 154.0 (dd, *J* = 290.2, 287.3 Hz), 143.9, 132.9, 130.3, 129.8, 129.5 (t, *J* = 3.2 Hz), 128.4, 128.3, 128.0, 126.6, 113.8, 89.3 (dd, *J* = 21.1, 15.1 Hz), 55.4 (d, *J* = 21.5 Hz), 36.2, 33.2, 30.6 (d, *J* = 2.0 Hz), 23.5, 23.2.). **^{19}F{^{1}H} NMR** (376 MHz, $CDCl_3$) 91.2 (d, *J* = 42.3 Hz), -91.7 (d, *J* = 42.2 Hz). **HRMS** (ESI$^+$): calcd. for

$C_{29}H_{28}F_2O_3Na$ (M + Na+): 485.1904. Found: 485.1898. **IR** (ATR) ῦ = 2957 (w), 1716 (s), 1610 (w), 1514 (m), 1450 (w), 1266 (s), 1246 (s), 1177 (m), 1107 (m), 1026 (m), 829 (m), 701 (s).

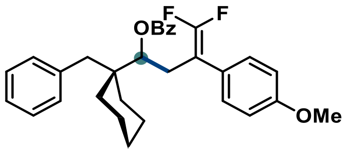

1-(1-Benzylcyclohexyl)-4,4-difluoro-3-(4-methoxyphenyl)but-3-en-1-yl benzoate (31). According to the **GP3**, using aldehyde **1ac** (0.3 mmol, 1.5 equiv.), benzoyl bromide (0.3 mmol, 1.5 equiv.), alkene **2a** (0.2 mmol, 1.0 equiv.), 4CzIPN (10 μmol, 5 mol%), $(TMS)_3SiOH$ (0.3 mmol, 1.5 equiv.), KOAc (0.8 mmol, 4.0 equiv.) in MeCN (4 ml) and stirring at room temperature for 36 h under 30W blue LEDs irradiation. The reaction mixture was filtered through a pad of silica gel and concentrated *in vacuo*, then purified by chromatography (pentane/EtOAc = 10/1 → 9/1) to give **31** (43.0 mg, 0.090 mmol, 45%) as a colorless oil. **R*f*** (pentane/EtOAc = 96:4): 0.60. **^{1}H NMR** (400 MHz, $CDCl_3$) δ 7.76 – 7.69 (m, 2H), 7.48 (tt, *J* = 7.5, 1.2 Hz, 1H), 7.34 (t, *J* = 7.8 Hz, 2H), 7.27 – 7.19 (m, 3H), 7.14 (d, *J* = 7.1 Hz, 4H), 6.78 – 6.72 (m, 2H), 5.48 (dd, *J* = 10.8, 2.2 Hz, 1H), 3.70 (s, 3H), 2.92 – 2.81 (m, 2H), 2.76 (d, *J* = 13.6 Hz, 1H), 2.70 (dq, *J* = 14.6, 2.8 Hz, 1H), 1.63 – 1.14 (m, 8H). **^{13}C{^{1}H} NMR** (101 MHz, $CDCl_3$) δ 165.6, 158.5, 153.8 (t, *J* = 287.5 Hz), 138.3, 132.4, 130.8, 129.9, 129.5 (t, *J* = 3.0 Hz), 129.4, 127.9, 127.7, 125.8, 125.3 (q, *J* = 2.9 Hz), 113.5, 89.2 (dd, *J* = 21.3, 15.8 Hz), 54.9, 40.9, 40.0, 32.4, 31.5, 28.2, 25.5, 21.1, 21.0. **^{19}F{^{1}H} NMR** (376 MHz, $CDCl_3$) δ -91.2 (d, *J* = 42.3 Hz), -91.7 (d, *J* = 42.5 Hz). **HRMS** (ESI+): calcd. for $C_{31}H_{32}F_2O_3Na$ (M + Na+): 513.2212. Found: 513.2207.

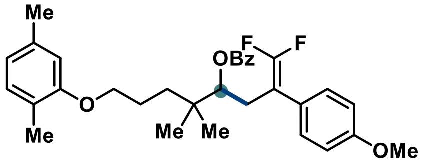

8-(2,5-Dimethylphenoxy)-1,1-difluoro-2-(4-methoxyphenyl)-5,5-dimethyloct-1-en-4-yl benzoate (32). According to the **GP3**, using aldehyde **1ad** (0.3 mmol, 1.5 equiv.), benzoyl bromide (0.3 mmol, 1.5 equiv.), alkene **2a** (0.2

mmol, 1.0 equiv.), 4CzIPN (10 μmol, 5 mol%), $(TMS)_3SiOH$ (0.3 mmol, 1.5 equiv.), KOAc (0.8 mmol, 4.0 equiv.) in MeCN (4 ml) and stirring at room temperature for 36 h under 30W blue LEDs irradiation. The reaction mixture was filtered through a pad of silica gel and concentrated *in vacuo*, then purified by chromatography (pentane/Et_2O = 99.5/0.5 → 95/5) to give **32** (24.7 mg, 0.047 mmol, 24%) as a colorless oil. **R*f*** (pentane/Et_2O = 96:4): 0.33. **^{1}H NMR** (400 MHz, $CDCl_3$) δ 7.95 – 7.88 (m, 2H), 7.59 – 7.49 (m, 1H), 7.40 (t, *J* = 7.7 Hz, 2H), 7.16 (d, *J* = 8.4 Hz, 2H), 7.00 (d, *J* = 7.4 Hz, 1H), 6.80 – 6.71 (m, 2H), 6.69 – 6.58 (m, 2H), 5.10 (dd, *J* = 10.4, 2.3 Hz, 1H), 3.96 – 3.79 (m, 2H), 3.72 (s, 3H), 2.99 – 2.48 (m, 2H), 2.30 (s, 3H), 2.13 (s, 3H), 1.82 (tt, *J* = 11.8, 6.1 Hz, 1H), 1.71 (tt, *J* = 12.4, 5.9 Hz, 1H), 1.62 – 1.40 (m, 2H), 1.01 (s, 3H), 1.00 (s, 3H). **^{13}C{^{1}H} NMR** (101 MHz, $CDCl_3$) δ 165.9, 158.8, 157.1, 154.2, 136.6, 132.8, 130.4, 130.3, 129.7 (t, *J* = 3.2 Hz), 128.5, 128.3, 125.5 (t, *J* = 4.6 Hz), 123.7, 120.8, 113.9, 112.0, 78.3, 68.3, 55.3, 37.4, 35.4, 29.0 (d, *J* = 1.5 Hz), 24.1, 23.8, 23.3, 21.6, 15.9. **^{19}F{^{1}H} NMR** (376 MHz, $CDCl_3$) δ -91.2 (d, *J* = 42.5 Hz), -91.7 (d, *J* = 42.5 Hz). **HRMS** (ESI^+): calcd. for $C_{32}H_{36}F_2O_4Na$ ($M + Na^+$): 545.2480. Found: 545.2474. **IR** (ATR) ῦ = 2953 (w), 1717 (s), 1610 (w), 1586 (w), 1515 (m), 1450 (w), 1263 (s), 1247 (s), 1178 (m), 1127 (m), 1111 (m), 1125 (m), 833(m), 800 (m), 708 (s).

3.2. Olefins Scope

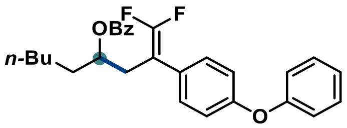

1,1-Difluoro-2-(4-phenoxyphenyl)non-1-en-4-yl benzoate (33). According to the **GP3**, using aldehyde **1a** (0.3 mmol, 1.5 equiv.), benzoyl bromide (0.3 mmol, 1.5 equiv.), alkene **2b** (0.2 mmol, 1.0 equiv.), 4CzIPN (10 μmol, 5 mol%), $(TMS)_3SiOH$ (0.3 mmol, 1.5 equiv.), KOAc (0.8 mmol, 4.0 equiv.) in MeCN (4 ml) and stirring at room temperature for 36 h under 30W blue LEDs irradiation. The reaction mixture was filtered through a pad of silica gel and concentrated *in vacuo*, then purified by chromatography (pentane/Et_2O = 9/1) to give **33** (57.3 mg, 0.102 mmol, 51%) as a colorless oil. **R*f*** (pentane/ Et_2O = 9/1) 0.41. **^{1}H NMR** (400 MHz, $CDCl_3$) δ 7.79 – 7.72 (m, 2H), 7.41 (tt, *J* = 7.5, 1.3 Hz, 1H), 7.33 – 7.24 (m, 2H), 7.23

– 7.17 (m, 2H), 7.14 – 7.11 (m, 2H), 6.98 (tt, *J* = 7.7, 1.2 Hz, 1H), 6.86 – 6.82 (m, 2H), 6.81 – 6.76 (m, 2H), 5.00 (tt, *J* = 7.4, 5.1 Hz, 1H), 2.70 (dddd, *J* = 11.6, 7.4, 2.6, 1.3 Hz, 1H), 2.64 – 2.52 (m, 1H), 1.61 – 1.44 (m, 2H), 1.29 – 1.07 (m, 6H), 0.80 – 0.60 (m, 3H). **^{13}C{^{1}H} NMR** (101 MHz, $CDCl_3$) δ 166.5, 157.4 (d, *J* = 58.1 Hz), 154.8 (t, *J* = 288.7 Hz), 133.3, 130.7, 130.2, 130.1 (t, *J* = 3.2 Hz), 130.0, 128.7, 128.4 (t, *J* = 4.0 Hz), 124.0, 119.7, 118.9, 89.3 (dd, *J* = 21.4, 15.7 Hz), 73.5 (t, *J* = 2.8 Hz), 34.2, 33.0, 32.1, 25.3, 22.9, 14.4. One aromatic signal is missing due to overlap. **^{19}F{^{1}H} NMR** (376 MHz, $CDCl_3$) δ -90.3 (d, *J* = 43.1 Hz), -90.6 (d, *J* = 38.8 Hz). **HRMS** (ESI$^+$): calcd. for $C_{28}H_{28}F_2O_3Na$ (M + Na$^+$): 473.1899. Found: 473.1893.

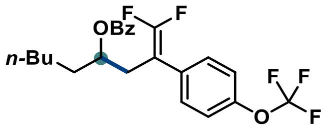

1,1-Difluoro-2-(4-(trifluoromethoxy)phenyl)non-1-en-4-yl benzoate (34). According to the **GP3**, using aldehyde **1a** (0.3 mmol, 1.5 equiv.), benzoyl bromide (0.3 mmol, 1.5 equiv.), alkene **2c** (0.2 mmol, 1.0 equiv.), 4CzIPN (10 μmol, 5 mol%), $(TMS)_3SiOH$ (0.3 mmol, 1.5 equiv.), KOAc (0.8 mmol, 4.0 equiv.) in MeCN (4 ml) and stirring at room temperature for 36 h under 30W blue LEDs irradiation. The reaction mixture was filtered through a pad of silica gel and concentrated *in vacuo*, then purified by chromatography (pentane/Et_2O = 100/1 → 80/1) to give **34** (61.0 mg, 0.138 mmol, 69%) as a colorless oil. **R*f*** (pentane/ Et_2O = 20/1) 0.85. **^{1}H NMR** (599 MHz, $CDCl_3$) δ 7.85 – 7.82 (m, 2H), 7.53 (ddt, *J* = 8.8, 7.2, 1.3 Hz, 1H), 7.41 – 7.36 (m, 2H), 7.34 – 7.31 (m, 2H), 7.16 – 7.10 (m, 2H), 5.13 (tt, *J* = 7.6, 5.0 Hz, 1H), 2.84 (dddd, *J* = 14.8, 7.5, 2.6, 1.6 Hz, 1H), 2.74 (ddt, *J* = 14.8, 4.9, 2.5 Hz, 1H), 1.74 – 1.67 (m, 1H), 1.67 – 1.60 (m, 1H), 1.40 – 1.20 (m, 6H), 0.87 – 0.83 (m, 3H). **^{13}C{^{1}H} NMR** (151 MHz, $CDCl_3$) δ 166.2, 154.6 (dd, *J* = 291.8, 288.7 Hz), 148.4 (q, *J* = 1.9 Hz), 133.0, 132.2 (dd, *J* = 4.6, 3.2 Hz), 130.2, 129.9 (t, *J* = 3.2 Hz), 129.5, 128.4, 121.0, 121.0 (q, *J* = 267.8 Hz), 88.7 (dd, *J* = 22.2, 15.0 Hz), 73.1 (t, *J* = 2.9 Hz), 33.9, 32.7 (d, *J* = 1.7 Hz), 31.7, 25.0, 22.6, 14.1. **^{13}C{^{1}H, ^{19}F} NMR** (151 MHz, $CDCl_3$) δ 166.2, 154.6, 148.4, 133.0, 132.2, 130.2, 129.9, 129.5, 128.4, 121.0, 88.7, 73.1, 33.9, 32.7, 31.7,

25.0, 22.6, 14.1. One signal cannot be observed due to overlap. **^{19}F NMR** (564 MHz, $CDCl_3$) δ -57.9 (t, *J* = 1.0 Hz), -89.0 (dt, *J* = 37.6, 2.6 Hz), -89.4 (dt, *J* = 37.5, 1.8 Hz). **^{19}F{^{1}H} NMR** (564 MHz, $CDCl_3$) δ -57.9, -89.9 (d, *J* = 37.7 Hz), -89.4 (d, *J* = 37.7 Hz). **HRMS** (ESI$^+$): calcd. for $C_{23}H_{23}F_5O_3Na$ (M + Na$^+$): 465.1460. Found: 465.1456.

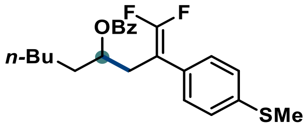

1,1-Difluoro-2-(4-(methylthio)phenyl)non-1-en-4-yl benzoate (35). According to the **GP3**, using aldehyde **1a** (0.15 mmol, 1.5 equiv.), benzoyl bromide (0.15 mmol, 1.5 equiv.), alkene **2d** (0.1 mmol, 1.0 equiv.), 4CzIPN (5 μmol, 5 mol%), $(TMS)_3SiOH$ (0.15 mmol, 1.5 equiv.), KOAc (0.4 mmol, 4.0 equiv.) in MeCN (2 ml) and stirring at room temperature for 36 h under 30W blue LEDs irradiation. The reaction mixture was filtered through a pad of silica gel and concentrated *in vacuo*, then purified by chromatography (pentane/Et_2O = 99/1 → 98/2) to give **35** (28.8 mg, 0.071 mmol, 71%) as a colorless oil. **R*f*** (pentane/ Et_2O = 96/4) 0.47. **^{1}H NMR** (599 MHz, $CDCl_3$) δ 7.82 (ddd, *J* = 8.0, 1.3, 0.8 Hz, 2H), 7.52 (ddt, *J* = 8.7, 7.3, 1.3 Hz, 1H), 7.41 – 7.34 (m, 2H), 7.24 – 7.21 (m, 2H), 7.18 – 7.14 (m, 2H), 5.11 (tt, *J* = 7.4, 5.1 Hz, 1H), 2.83 (dd, *J* = 14.7, 7.3 Hz, 1H), 2.72 (dd, *J* = 14.7, 5.2 Hz, 1H), 2.43 (s, 3H), 1.74 – 1.58 (m, 2H), 1.42 – 1.19 (m, 6H), 0.91 – 0.80 (m, 3H). **^{13}C{^{1}H, ^{19}F} NMR** (151 MHz, $CDCl_3$) δ 166.2, 154.5, 137.9, 132.9, 130.4, 130.2, 129.6, 128.8, 128.3, 126.6, 89.1, 73.3, 33.8, 32.5, 31.8, 25.0, 22.6, 15.8, 14.1. **^{19}F{^{1}H} NMR (**564 MHz, $CDCl_3$) δ -89.8 (d, *J* = 39.6 Hz), -90.0 (d, *J* = 39.5 Hz). **HRMS** (ESI$^+$): calcd. for $C_{23}H_{26}F_2NaO_2SNa$ (M + Na$^+$): 427.1519. Found: 427.1511.

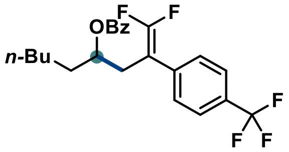

1,1-Difluoro-2-(4-(trifluoromethyl)phenyl)non-1-en-4-yl benzoate (36). According to the **GP3**, using aldehyde **1a** (0.3 mmol, 1.5 equiv.), benzoyl bromide

(0.3 mmol, 1.5 equiv.), alkene **2e** (0.2 mmol, 1.0 equiv.), 4CzIPN (10 μmol, 5 mol%), $(TMS)_3SiOH$ (0.3 mmol, 1.5 equiv.), KOAc (0.8 mmol, 4.0 equiv.) in MeCN (4 ml) and stirring at room temperature for 36 h under 30W blue LEDs irradiation. The reaction mixture was filtered through a pad of silica gel and concentrated *in vacuo*, then purified by chromatography (pentane/Et_2O = 100/0 → 99.5/1.5) followed by preperative TLC (pentane/Et_2O = 98/2) to give **36** (34.2 mg, 0.080 mmol, 40%) as a colorless oil. **R*f*** (pentane/ Et_2O = 98/2) 0.37. **^{1}H NMR** (500 MHz, $CDCl_3$) δ 7.76 – 7.73 (m, 2H), 7.53 – 7.49 (m, 3H), 7.43 – 7.40 (m, 2H), 7.38 – 7.32 (m, 2H), 5.13 (tt, *J* = 7.5, 5.0 Hz, 1H), 2.90 – 2.76 (m, 2H), 1.77 – 1.59 (m, 2H), 1.41 – 1.20 (m, 6H), 0.88 – 0.82 (m, 3H). **^{13}C{^{1}H, ^{19}F} NMR** (126 MHz, $CDCl_3$) δ 154.8, 137.4, 133.1, 130.1, 129.6, 129.5, 128.8, 128.3, 125.6, 124.1, 89.1, 73.2, 33.9, 32.5, 31.7, 25.0, 22.6, 14.1. **^{19}F{^{1}H} NMR** (470 MHz, $CDCl_3$) δ -62.7, -87.9 (d, *J* = 35.3 Hz), -88.6 (d, *J* = 35.3 Hz). **HRMS** (ESI^+): calcd. for $C_{23}H_{23}F_5O_2Na$ ($M + Na^+$): 449.1516. Found: 449.1511.

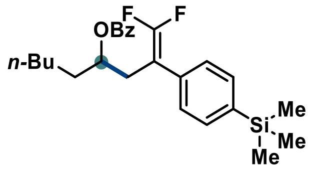

1,1-Difluoro-2-(4-(trimethylsilyl)phenyl)non-1-en-4-yl benzoate (37). According to the **GP3**, using aldehyde **1a** (0.3 mmol, 1.5 equiv.), benzoyl bromide (0.3 mmol, 1.5 equiv.), alkene **2f** (0.2 mmol, 1.0 equiv.), 4CzIPN (10 μmol, 5 mol%), $(TMS)_3SiOH$ (0.3 mmol, 1.5 equiv.), KOAc (0.8 mmol, 4.0 equiv.) in MeCN (4 ml) and stirring at room temperature for 36 h under 30W blue LEDs irradiation. The reaction mixture was filtered through a pad of silica gel and concentrated *in vacuo*, then purified by chromatography (pentane/EtOAc = 16/1) to give **37** (41.3 mg, 0.096 mmol, 48%) as a colorless oil. **R$_f$** (pentane/EtOAc = 16/1) 0.40. **^{1}H NMR** (500 MHz, $CDCl_3$) δ 7.87 – 7.80 (m, 2H), 7.51 (ddt, *J* = 8.7, 7.3, 1.3 Hz, 1H), 7.47 – 7.41 (m, 2H), 7.41 – 7.34 (m, 2H), 7.32 – 7.27 (m, 2H), 5.13 (tt, *J* = 7.4, 5.2 Hz, 1H), 2.85 (ddt, *J* = 14.7, 7.4, 2.1 Hz, 1H), 2.75 (ddt, *J* = 14.7, 5.1, 2.5 Hz, 1H), 1.73 – 1.60

(m, 2H), 1.39 – 1.20 (m, 6H), 0.88 – 0.80 (m, 3H), 0.23 (s, 9H). **^{13}C{^{1}H, ^{19}F} NMR** (126 MHz, $CDCl_3$) δ 166.2, 154.6, 139.7, 133.8, 133.6, 132.9, 130.5, 129.6, 128.3, 127.6, 89.5, 73.3, 33.8, 32.5, 31.8, 24.9, 22.6, 14.1, -1.0 (d, *J* = 9.4 Hz). **^{19}F NMR** (470 MHz, $CDCl_3$) δ -89.6 (dt, *J* = 38.9, 2.6 Hz), -89.9 (dt, *J* = 38.7, 1.7 Hz). **^{19}F{^{1}H} NMR** (470 MHz, $CDCl_3$) δ -89.6 (d, *J* = 38.9 Hz), -89. 9 (d, *J* = 38.9 Hz). **HRMS** (ESI$^+$): calcd. for $C_{25}H_{32}F_2O_2Na$ (M + Na$^+$): 453.2032. Found: 453.2024.

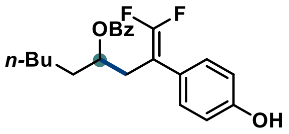

1**,1-Difluoro-2-(4-hydroxyphenyl)non-1-en-4-yl benzoate (38).** According to the **GP3**, using aldehyde **1a** (0.3 mmol, 1.5 equiv.), benzoyl bromide (0.3 mmol, 1.5 equiv.), alkene **2g** (0.2 mmol, 1.0 equiv.), 4CzIPN (10 μmol, 5 mol%), $(TMS)_3SiOH$ (0.3 mmol, 1.5 equiv.), KOAc (0.8 mmol, 4.0 equiv.) in MeCN (4 ml) and stirring at room temperature for 36 h under 30W blue LEDs irradiation. The reaction mixture was filtered through a pad of silica gel and concentrated *in vacuo*, then purified by chromatography (pentane/Et_2O = 93/7 → 92/8) to give **38** (18.1 mg, 0.048 mmol, 24%) as a colorless oil. **R*f*** (pentane/ EtOAc = 9/1) 0.51. **^{1}H NMR** (599 MHz, $CDCl_3$) δ 7.91 – 7.86 (m, 2H), 7.56 – 7.50 (m, 1H), 7.44 – 7.37 (m, 2H), 7.19 – 7.15 (m, 2H), 6.78 – 6.74 (m, 2H), 5.10 (ddd, *J* = 12.7, 7.4, 5.2 Hz, 1H), 2.82 (dd, *J* = 14.5, 7.5 Hz, 1H), 2.71 – 2.62 (m, 1H), 1.75 – 1.58 (m, 2H), 1.39 – 1.21 (m, 8H), 0.86 – 0.81 (m, 3H). **^{13}C{^{1}H, ^{19}F} NMR** (126 MHz, $CDCl_3$) δ 166.4, 155.1, 154.4, 133.0, 130.4, 129.8, 129.7, 128.4, 125.6, 115.6, 88.9, 73.3, 33.8, 32.8, 31.8, 25.0, 22.6, 14.1. **^{19}F{^{1}H} NMR** (470 MHz, $CDCl_3$) δ -91.0 (d, *J* = 41.9 Hz), -91.1 (d, *J* = 41.9 Hz). **HRMS** (ESI$^+$): calcd. for $C_{22}H_{24}F_2O_3Na$ (M + Na$^+$): 397.1591. Found: 397.1586.

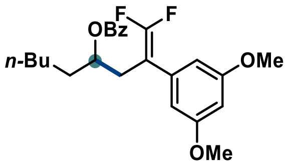

2-(3,5-dimethoxyphenyl)-1,1-difluoronon-1-en-4-yl benzoate (39). According to the **GP3**, using aldehyde **1a** (0.3 mmol, 1.5 equiv.), benzoyl bromide (0.3 mmol, 1.5 equiv.), alkene **2h** (0.2 mmol, 1.0 equiv.), 4CzIPN (10 μmol, 5 mol%), $(TMS)_3SiOH$ (0.3 mmol, 1.5 equiv.), KOAc (0.8 mmol, 4.0 equiv.) in MeCN (4 ml) and stirring at room temperature for 36 h under 30W blue LEDs irradiation. The reaction mixture was filtered through a pad of silica gel and concentrated *in vacuo*, then purified by chromatography (pentane/Et_2O = 20/1) to give **39** (49.7 mg, 0.119 mmol, 60%) as a colorless oil. **R_f** (pentane/ EtOAc = 20/1) 0.35. **^{1}H NMR** (400 MHz, $CDCl_3$) δ 7.90 – 7.83 (m, 2H), 7.53 (ddt, *J* = 8.8, 7.0, 1.4 Hz, 1H), 7.42 – 7.33 (m, 2H), 6.44 (dd, *J* = 2.3, 1.1 Hz, 2H), 6.28 (t, *J* = 2.3 Hz, 1H), 5.14 (tt, *J* = 7.4, 5.1 Hz, 1H), 3.71 (s, 6H), 2.81 (dddd, *J* = 14.6, 7.4, 2.6, 1.6 Hz, 1H), 2.72 (ddt, *J* = 14.6, 5.1, 2.5 Hz, 1H), 1.76 – 1.58 (m, 2H), 1.41 – 1.19 (m, 6H), 0.89 – 0.80 (m, 3H). **^{13}C{^{1}H} NMR** (101 MHz, $CDCl_3$) δ 166.1, 160.8, 154.5 (dd, *J* = 291.6, 287.7 Hz), 135.4 (t, *J* = 4.6 Hz), 132.8, 130.4, 129.6, 128.3, 106.7, 99.5, 89.6 (dd, *J* = 21.7, 14.9 Hz), 73.2 (t, *J* = 2.7 Hz), 55.4, 33.8, 32.7 (d, *J* = 1.7 Hz), 31.7, 25.0, 22.6, 14.1. **^{19}F{^{1}H} NMR** (376 MHz, $CDCl_3$) δ -88.8 (d, *J* = 38.8 Hz), -89.7 (d, *J* = 38.7 Hz). **HRMS** (ESI$^+$): calcd. for $C_{24}H_{28}F_2O_4Na$ (M + Na^+): 441.1848. Found: 441.1843.

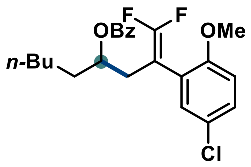

2-(5-Chloro-2-methoxyphenyl)-1,1-difluoronon-1-en-4-yl benzoate (40). According to the **GP3**, using aldehyde **1a** (0.3 mmol, 1.5 equiv.), benzoyl bromide (0.3 mmol, 1.5 equiv.), alkene **2i** (0.2 mmol, 1.0 equiv.), 4CzIPN (10 μmol, 5 mol%), $(TMS)_3SiOH$ (0.3 mmol, 1.5 equiv.), KOAc (0.8 mmol, 4.0 equiv.) in MeCN (4 ml) and stirring at room temperature for 36 h under 30W blue LEDs irradiation. The reaction mixture was filtered through a pad of silica gel and concentrated *in vacuo*, then purified by chromatography (pentane/Et_2O = 99/1 → 97.5/2.5) to give **40** (71.7 mg, 0.170 mmol, 85%) as a colorless oil. **R_f** (Pentane/EtOAc = 98/2): 0.51.

^{1}**H NMR** (599 MHz, $CDCl_3$) δ 7.84 (dd, *J* = 8.2, 1.4 Hz, 2H), 7.59 – 7.48 (m, 1H), 7.41 – 7.35 (m, 2H), 7.15 – 7.08 (m, 2H), 6.68 (d, *J* = 8.7 Hz, 1H), 5.05 (tt, *J* = 7.3, 5.0 Hz, 1H), 3.64 (s, 3H), 2.74 (qdq, *J* = 9.7, 4.8, 2.4 Hz, 2H), 1.74 – 1.60 (m, 2H), 1.39 – 1.18 (m, 8H), 0.89 – 0.82 (m, 3H). ^{13}**C{^{1}H} NMR** (151 MHz, $CDCl_3$) δ 166.0, 155.9 (d, *J* = 2.4 Hz), 154.1 (t, *J* = 288.8 Hz), 132.8, 130.8 – 130.7 (m), 130.5, 129.6, 128.9, 128.3, 125.4, 124.0 (dd, *J* = 4.9, 2.0 Hz), 112.2, 85.6 (dd, *J* = 24.0, 18.1 Hz), 73.2 (t, *J* = 2.8 Hz), 55.7, 34.0, 32.5 (d, *J* = 1.7 Hz), 31.8, 24.9, 22.6, 14.1. ^{19}**F{^{1}H} NMR** (564 MHz, $CDCl_3$) δ -87.2 (d, *J* = 36.9 Hz), -91.3 (d, *J* = 37.0 Hz). **HRMS** (ESI$^+$): calcd. for $C_{23}H_{25}ClF_2O_3Na$ (M + Na$^+$): 445.1358. Found: 445.1351.

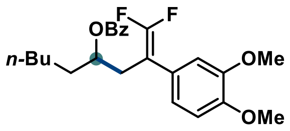

2-(3,4-Dimethoxyphenyl)-1,1-difluoronon-1-en-4-yl benzoate (41). According to the **GP3**, using aldehyde **1a** (0.3 mmol, 1.5 equiv.), benzoyl bromide (0.3 mmol, 1.5 equiv.), alkene **2j** (0.2 mmol, 1.0 equiv.), 4CzIPN (10 μmol, 5 mol%), $(TMS)_3SiOH$ (0.3 mmol, 1.5 equiv.), KOAc (0.8 mmol, 4.0 equiv.) in MeCN (4 ml) and stirring at room temperature for 36 h under 30W blue LEDs irradiation. The reaction mixture was filtered through a pad of silica gel and concentrated *in vacuo*, then purified by chromatography (pentane/Et_2O = 92/8 → 85/15) to give **41** (38.9 mg, 0.093 mmol, 47%) as a colorless oil. * *isolated sample contains 7% of hexanoate indicated by chemical shifts at 4.90 (tt, J = 7.2, 6.6 Hz) ppm in ^{1}HNMR and -90.37 (d, J = 40.9 Hz) and -90.65 (d, J = 40.9 Hz) ppm in ^{19}F NMR.* **R$_f$** (Pentane/Et_2O = 92/8): 0.18. ^{1}**H NMR** (500 MHz, $CDCl_3$) δ 7.90 – 7.84 (m, 2H), 7.58 – 7.50 (m, 1H), 7.43 – 7.36 (m, 2H), 6.91 – 6.86 (m, 1H), 6.82 – 6.77 (m, 2H), 5.14 (tt, *J* = 7.3, 5.1 Hz, 1H), 3.84 (s, 3H), 3.82 (s, 3H), 2.88 – 2.79 (m, 1H), 2.72 (ddt, *J* = 14.6, 5.1, 2.3 Hz, 1H), 1.77 – 1.60 (m, 2H), 1.42 – 1.21 (m, 6H), 0.86 (dt, *J* = 6.1, 4.0 Hz, 3H). ^{13}**C{^{1}H, ^{19}F} NMR** (126 MHz, $CDCl_3$) δ 166.2, 154.5, 148.9, 148.4, 132.9, 130.5, 129.56, 128.3, 125.9, 120.9, 111.8, 111.2, 89.3, 73.3, 56.0, 55.9, 33.8, 32.8, 31.7, 25.0, 22.6, 14.1.

^{19}F{^{1}H} NMR (470 MHz, $CDCl_3$) δ -90.4 (d, *J* = 41.8 Hz), -90.8 (d, *J* = 41.7 Hz). **HRMS** (ESI$^+$): calcd. for $C_{24}H_{28}F_2O_4Na$ (M + Na$^+$): 441.1853. Found: 441.1842.

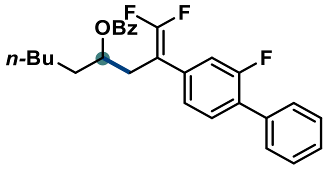

1,1-Difluoro-2-(2-fluoro-[1,1'-biphenyl]-4-yl)non-1-en-4-yl benzoate (42). According to the **GP3**, using aldehyde **1a** (0.3 mmol, 1.5 equiv.), benzoyl bromide (0.3 mmol, 1.5 equiv.), alkene **2k** (0.2 mmol, 1.0 equiv.), 4CzIPN (10 μmol, 5 mol%), $(TMS)_3SiOH$ (0.3 mmol, 1.5 equiv.), KOAc (0.8 mmol, 4.0 equiv.) in MeCN (4 ml) and stirring at room temperature for 36 h under 30W blue LEDs irradiation. The reaction mixture was filtered through a pad of silica gel and concentrated *in vacuo*, then purified by chromatography (pentane/Et_2O = 70/1 → 60/1) to give **41** (63.2 mg, 0.140 mmol, 70%) as a colorless oil. **R_f** (Pentane/Et_2O = 50/1): 0.40. **^{1}H NMR** (500 MHz, $CDCl_3$) δ 7.88 – 7.83 (m, 2H), 7.52 – 7.41 (m, 5H), 7.39 – 7.32 (m, 4H), 7.18 (dt, *J* = 8.1, 1.5 Hz, 1H), 7.14 (dt, *J* = 11.8, 1.3 Hz, 1H), 5.21 (tt, *J* = 7.4, 5.0 Hz, 1H), 2.87 (dddd, *J* = 14.8, 7.3, 2.6, 1.7 Hz, 1H), 2.81 (ddt, *J* = 14.8, 5.0, 2.4 Hz, 1H), 1.80 – 1.73 (m, 1H), 1.72 – 1.63 (m, 1H), 1.45 – 1.25 (m, 6H), 0.89 – 0.85 (m, 3H). **^{13}C{^{1}H} NMR** (126 MHz, $CDCl_3$) δ 166.1, 159.7 (d, *J* = 248.3 Hz), 154.8 (dd, *J* = 293.0, 289.0 Hz), 135.4 (d, *J* = 1.4 Hz), 134.6 (dd, *J* = 7.9, 3.7 Hz), 133.0, 130.8 (d, *J* = 4.2 Hz), 130.3, 129.6, 129.1 (d, *J* = 3.0 Hz), 128.5, 128.3, 128.1-128.0 (m), 127.9, 124.3 (q, *J* = 3.2 Hz), 116.1 (dt, *J* = 24.5, 3.4 Hz), 88.8 (ddd, *J* = 20.5, 14.3, 1.9 Hz), 73.3 (t, *J* = 2.9 Hz), 33.9, 32.4 (d, *J* = 1.4 Hz), 31.7, 25.0, 22.6, 14.1. **^{13}C{^{19}F} NMR** (126 MHz, $CDCl_3$) δ 166.1, 159.7, 154.8, 135.4, 134.6, 133.0, 130.8, 130.3, 129.6, 129.1, 128.5, 128.3, 128.1, 127.9, 124.3, 116.1, 88.8, 73.3, 33.9, 32.4, 31.7, 25.0, 22.6, 14.1. **^{19}F NMR** (470 MHz, $CDCl_3$) δ -88.1 (dt, *J* = 36.1, 2.7 Hz), -88.4 (dt, *J* = 36.1, 1.6 Hz), -117.7 (dt, *J* = 11.8, 1.4 Hz). **^{19}F{^{1}H} NMR** (470 MHz, $CDCl_3$) δ -88.1 (d, *J* = 36.0 Hz), -88.4 (d, *J* = 36.1 Hz), -117.7. **HRMS** (ESI$^+$): calcd. for $C_{28}H_{27}F_3O_2Na$ (M + Na$^+$): 475.1855. Found: 475.1853.

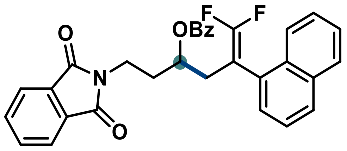

7-(1,3-Dioxoisoindolin-2-yl)-1,1-difluoro-2-(naphthalen-1-yl)hept-1-en-4-yl benzoate (43). According to the **GP3**, using aldehyde **1a** (0.3 mmol, 1.5 equiv.), benzoyl bromide (0.3 mmol, 1.5 equiv.), alkene **2l** (0.2 mmol, 1.0 equiv.), 4CzIPN (10 μmol, 5 mol%), $(TMS)_3SiOH$ (0.3 mmol, 1.5 equiv.), KOAc (0.8 mmol, 4.0 equiv.) in MeCN (4 ml) and stirring at room temperature for 36 h under 30W blue LEDs irradiation. The reaction mixture was filtered through a pad of silica gel and concentrated *in vacuo*, then purified by chromatography (pentane/EtOAc= 10/1) to give **43** (59.9 mg, 0.114 mmol, 57%) as a colorless oil. **R_f** (pentane/EtOAc= 10/1) 0.50. *Indered rotation around the napthalene moiety causes signal broadening due to rotamers presence. Some resonances cannot be detected.* **^{1}H NMR** (400 MHz, $CDCl_3$) δ 7.99 (dt, *J* = 6.8, 1.4 Hz, 1H), 7.85 – 7.78 (m, 2H), 7.73 – 7.67 (m, 1H), 7.67 – 7.61 (m, 2H), 7.56 – 7.28 (m, 8H), 5.24 – 5.06 (br, 1H), 4.39 (t, *J* = 6.1 Hz, 1H), 3.91 (t, *J* = 6.9 Hz, 1H), 3.85 – 3.65 (br, 2H), 3.22 – 2.63 (br, 1H), 2.28 – 2.10 (br, 2H). **^{13}C{^{1}H} NMR** (101 MHz, $CDCl_3$) δ 168.1, 167.8, 166.2, 133.8, 133.6, 132.7, 131.9, 131.8, 131.3 – 130.7 (br), 129.3, 129.3 – 129.1 (br), 128.5, 128.1, 128.0 – 127.6 (br), 126.3, 125.7, 123.1, 123.0, 86.6 (t, *J* = 20.7 Hz), 62.3, 35.1, 34.2, 27.5.

^{19}F{^{1}H} NMR (377 MHz, $CDCl_3$) δ -85.8 (apt dd, *J* = 223.4, 37.3 Hz), -90.5 (apt dd, *J* = 159.8, 37.3 Hz). **HRMS** (ESI$^+$): calcd. for $C_{31}H_{23}NF_2O_4Na$ (M + Na$^+$): 534.1487. Found: 534.1487.

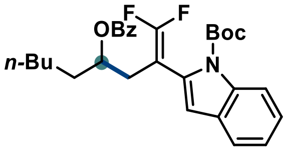

***Tert*-butyl 2-(4-(benzoyloxy)-1,1-difluoronon-1-en-2-yl)-1H-indole-1-carboxylate (44).** According to the **GP3**, using aldehyde **1a** (0.3 mmol, 1.5 equiv.),

benzoyl bromide (0.3 mmol, 1.5 equiv.), alkene **2m** (0.2 mmol, 1.0 equiv.), 4CzIPN (10 μmol, 5 mol%), $(TMS)_3SiOH$ (0.3 mmol, 1.5 equiv.), KOAc (0.8 mmol, 4.0 equiv.) in MeCN (4 ml) and stirring at room temperature for 36 h under 30W blue LEDs irradiation. The reaction mixture was filtered through a pad of silica gel and concentrated *in vacuo*, then purified by chromatography (pentane/Et_2O = 100/0 → 98.7/1.3) to give **44** (29.0 mg, 0.058 mmol, 29%) as a colorless oil. $\mathbf{R_f}$ (pentane/EtOAc = 98/2): 0.44. ^{1}**H NMR** (500 MHz, $CDCl_3$) δ 8.04 (dq, *J* = 8.4, 0.8 Hz, 1H), 7.86 – 7.81 (m, 2H), 7.51 – 7.42 (m, 2H), 7.34 – 7.26 (m, 3H), 7.19 (td, *J* = 7.4, 1.0 Hz, 1H), 6.53 (s, 1H), 5.25 – 5.16 (m, 1H), 2.80 (dd, *J* = 15.0, 8.6 Hz, 1H), 2.64 (d, *J* = 14.9 Hz, 1H), 1.79 – 1.66 (m, 2H), 1.64 (s, 9H), 1.44 – 1.17 (m, 6H), 0.86 – 0.80 (m, 3H). ^{13}**C{^{1}H, ^{19}F} NMR** (126 MHz, $CDCl_3$) δ 166.2, 154.7, 149.9, 136.9, 132.8, 131.1, 130.3, 129.6, 129.1, 128.3, 124.6, 123.0, 120.8, 115.8, 112.0, 84.5, 84.1, 73.0, 34.3, 33.5, 31.8, 28.2, 25.0, 22.6, 14.1. ^{19}**F{^{1}H} NMR** (470 MHz, $CDCl_3$) δ -86.40 (d, *J* = 34.2 Hz), -90.43 (br s). **HRMS** (ESI^+): calcd. for $C_{29}H_{33}F_2NO_4Na$ (M + Na^+): 520.2275. Found: 520.2272.

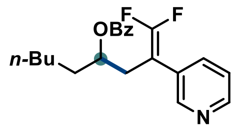

1,1-Difluoro-2-(pyridin-3-yl)non-1-en-4-yl benzoate (45). According to the **GP3**, using aldehyde **1a** (0.3 mmol, 1.5 equiv.), benzoyl bromide (0.3 mmol, 1.5 equiv.), alkene **2n** (0.2 mmol, 1.0 equiv.), 4CzIPN (10 μmol, 5 mol%), $(TMS)_3SiOH$ (0.3 mmol, 1.5 equiv.), KOAc (0.8 mmol, 4.0 equiv.) in MeCN (4 ml) and stirring at room temperature for 36 h under 30W blue LEDs irradiation. The reaction mixture was filtered through a pad of silica gel and concentrated *in vacuo*, then purified by chromatography (pentane/Et_2O = 90/10 → 70/30) to give **45** (16.1 mg, 0.048 mmol, 22%) as a colorless oil. $\mathbf{R_f}$ (pentane/EtOAc = 80/20): 0.29. ^{1}**H NMR** (500 MHz, $CDCl_3$) δ 8.62 (s, 1H), 8.45 (s, 1H), 7.86 – 7.80 (m, 2H), 7.67 (d, *J* = 7.9 Hz, 1H), 7.53 (dddd, *J* = 8.0, 6.4, 1.6, 1.0 Hz, 1H), 7.42 – 7.34 (m, 2H), 5.12 (tt, *J* = 7.7, 4.9 Hz, 1H), 2.86 (dd, *J* = 14.9, 7.6 Hz, 1H), 2.77 (dd, *J* = 14.8, 4.9 Hz, 1H), 1.78

– 1.59 (m, 2H), 1.40 – 1.14 (m, 6H), 0.90 – 0.81 (m, 3H). **^{13}C{^{1}H, ^{19}F} NMR** (151 MHz, $CDCl_3$) δ 166.1, 154.9, 148.6, 147.6, 136.59, 133.1, 130.2, 130.1, 129.6, 128.4, 123.7, 86.9, 72.9, 34.0, 31.7, 25.0, 22.6, 14.1. **^{19}F{^{1}H} NMR** (470 MHz, $CDCl_3$) δ -87.1 (d, *J* = 34.3 Hz), -88.2 (d, *J* = 34.3 Hz). **HRMS** (ESI$^+$): calcd. for $C_{21}H_{24}F_2NO_2Na$ (M + Na$^+$): 360.1775. Found: 360.1770. **IR** (ATR) ῦ = 2951 (w), 2854 (w), 1717 (s), 1609 (w), 1514 (m), 1445 (w), 1255 (s), 1237 (s), 1177 (m), 1099 (m), 1025 (s), 831 (m), 701 (s).

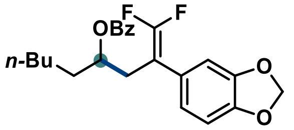

2-(Benzo[*d*][1,3]dioxol-5-yl)-1,1-difluoronon-1-en-4-yl benzoate (46). According to the **GP3**, using aldehyde **1a** (0.3 mmol, 1.5 equiv.), benzoyl bromide (0.3 mmol, 1.5 equiv.), alkene **2o** (0.2 mmol, 1.0 equiv.), 4CzIPN (10 µmol, 5 mol%), $(TMS)_3SiOH$ (0.3 mmol, 1.5 equiv.), KOAc (0.8 mmol, 4.0 equiv.) in MeCN (4 ml) and stirring at room temperature for 36 h under 30W blue LEDs irradiation. The reaction mixture was filtered through a pad of silica gel and concentrated *in vacuo*, then purified by chromatography (pentane/EtOAc = 20/1) to give **46** (50.5 mg, 0.130 mmol, 65%) as a pale yellow oil. **R_f** (pentane/EtOAc = 20/1): 0.55. **^{1}H NMR** (400 MHz, $CDCl_3$) δ 7.90 – 7.83 (m, 2H), 7.53 (tt, *J* = 7.5, 1.2 Hz, 1H), 7.40 (t, *J* = 7.8 Hz, 2H), 6.78 – 6.70 (m, 1H), 5.88 (dd, *J* = 17.2, 1.3 Hz, 2H), 5.11 (td, *J* = 7.5, 3.8 Hz, 1H), 2.77 (dt, *J* = 14.6, 1.9 Hz, 1H), 2.69 (dddd, *J* = 14.5, 9.6, 6.1, 2.5 Hz, 1H), 1.75 – 1.55 (m, 3H), 1.37 – 1.21 (m, 6H), 0.88 – 0.81 (m, 3H). **^{13}C{^{1}H} NMR** (101 MHz, $CDCl_3$) δ 166.4, 154.8 (dd, *J* = 290.2, 287.7 Hz), 148.2, 147.2, 133.2, 130.7, 129.9, 128.6, 127.4 (t, *J* = 4.0 Hz), 122.3 (t, *J* = 3.1 Hz), 109.3 (t, *J* = 3.3 Hz), 108.8, 101.5, 89.6 (dd, *J* = 21.7, 15.6 Hz), 73.5 (t, *J* = 2.8 Hz), 34.2, 33.2 (d, *J* = 1.5 Hz), 32.1, 25.3, 22.9, 14.4. **^{19}F{^{1}H} NMR** (377 MHz, $CDCl_3$) δ 159.4 (d, *J* = 41.0 Hz), 159.1 (d, *J* = 41.1 Hz). **HRMS** (ESI$^+$): calcd. for $C_{22}H_{22}F_2O_4Na$ (M + Na$^+$): 411.4006. Found: 411.4003.

3.3. Olefins bearing electron-withdrawing groups

α-Trifluoromethylstyrenes with carbonyl-containing substituents in *para* position were subjected to our optimized conditions. We observed formation of substantial amounts of trifluoromethyl alkanes.

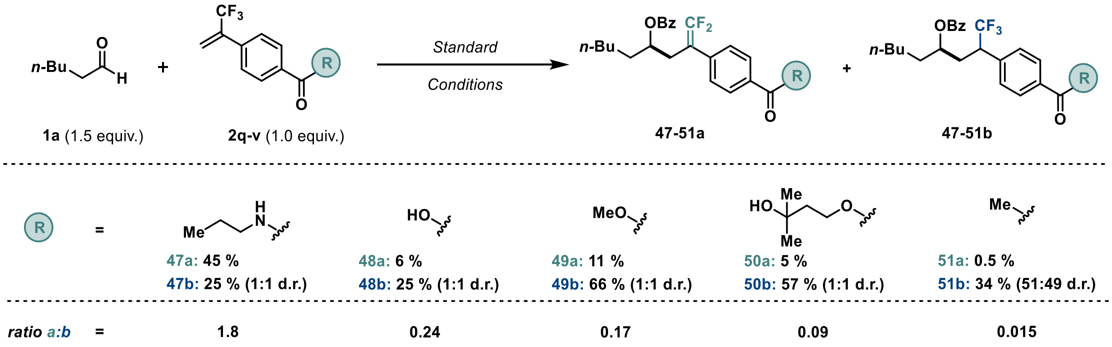

Supplementary Figure 4. Reaction outcomes when employing EWG-substituted α-trifluoromethylstyrenes. Yields and ratios were obtained by ^{19}F NMR, using 1-fluoronaphthalene as internal standard.

Analysis of the NMR yields (**Supplementary Figure 4**) revealed an increase of the ratio between alkene and alkane going from amide to carboxylic acid, to esters. Ketone (**51a-b**) corroborated the trend that the ratio increases with increasing mesomeric effect of the substituent. We envisioned two possible pathways that can lead to the formation of the trifluoromethyl alkanes. Firstly, radical **VIII** may undergo HAT to form alkane products **47-51b**. Notably, HAT catalyst tetrabutylammonium decatungstate (TBADT) was employed previously to exactly follow this pathway.[36] However, in the reported procedure, substrates bearing electron withdrawing groups (e.g. esters) or electron donating groups (e.g. methoxy) afforded similar yields. Another proposed pathway features the protonation of anionic species **IX**. We performed DFT calculations to account for the observed reactivity pitfalls.

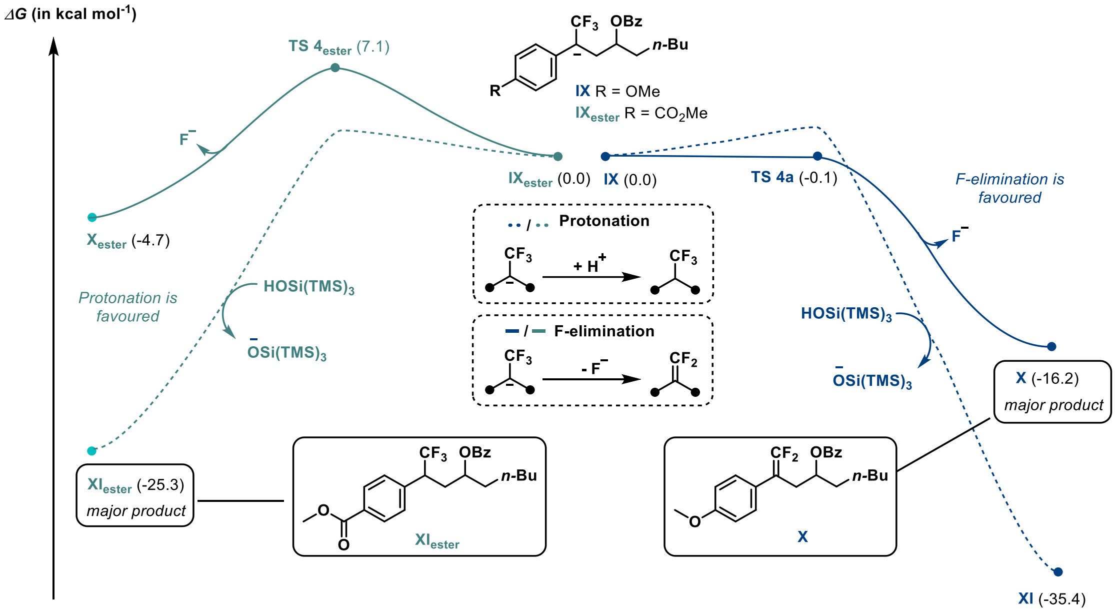

Supplementary Figure 5. DFT-computed profile between EWG- and EWG-substituted α-trifluoromethylstyrenes. Details regarding DFT calculations are reported in Section **4.2**.

From trifluoroalkane radical **VIII**, single electron transfer with singly reduced 4CzIPN (E_{ox}(4CzIPN/4CzIPN$^{\bullet-}$) = - 1.21 V vs. SCE)[37] to afford trifluoroalkane anion **IX** is expected to take place. Under consideration of the redox potential of the photocatalyst, Gibbs free energies of – 0.3 kcal mol^{-1} (for **VIII** → **IX**) and – 14.1 kcal mol^{-1} (for **VIII$_{ester}$** → **IX$_{ester}$**) were calculated. This remarkable difference in energy ($\Delta\Delta G$ = – 13.8 kcal mol^{-1}) can be explained by the electron withdrawing effect of the ester substituent which stabilizes the negative charge of formed anion **XI$_{ester}$**. Next, we sought to compare the energy profiles of protonation and fluoride elimination. For methoxy substituted anion **IXa** (**Supplementary Figure 5**, blue), we found that E1cb-type fluoride elimination for **IX** → **X** is significantly exergonic (ΔG = - 16.2 kcal mol^{-1}), making this an irreversible process. Besides that, no kinetic energy barrier was detected even though a transition state was found.[1] Hence, this intramolecular chemical transformation occurs immediately once intermediate **IX** is formed. Assuming that protonation occurs from supersilanol **I**,

[1] $\Delta G^{\ddagger}$ = - 0.1 kcal mol^{-1} which may arise from slight errors in the prediction. Further calculations with different functionals and basis sets may be required to obtain more precise results.

it was found that this process is thermodynamically favoured compared to a defluorination (ΔG = - 35.4 kcal mol^{-1}). However, given that the decay of **IX** to **X** is essentially barrierless, we assume that the concentration of **IX** is diminished to such an extent that the bimolecular protonation, which has a rate constant with a first order dependence on the concentration of **IX**, cannot take place.[2]

In comparison, for ester substituted anion **IX$_{ester}$** (**Supplementary Figure 5**, in turquoise) it was found that the chemical decay to **X$_{ester}$** *via* fluoride elimination is less exergonic (ΔG = - 4.7 kcal mol^{-1}). Furthermore, a kinetic energy barrier of 7.1 kcal mol^{-1} is associated to this process. Hence, it is expected that fluoride elimination is slower than for methoxy-susbtituted anion **IX**. Consequently, intermediate **IX$_{ester}$** is longer-lived, and a higher concentration is expected. Given that protonation is highly exergonic (ΔG = -25.3 kcal mol^{-1}), intermediate **IX$_{ester}$** can be scavenged by protonation. Since the proton transfer in the thermodynamically favoured direction is diffusion controlled,[38] a low kinetic energy barrier of the protonation is expected (as indicated by the dashed line in **Supplementary Figure 5**). This makes the protonation of intermediate **IX$_{ester}$** both kinetically and thermodynamically favoured over intramolecular E1cb process, which is in line with the experimental data.

---

[2] Due to numerous possibilities on how the protonation can occur, no meaningful transition state for the protonation step can be obtained. However, usually protonation in the thermodynamically favored direction are associated to a low kinetic energy barrier.[38]

4. Mechanistic investigation

4.1. Radical probe experiment

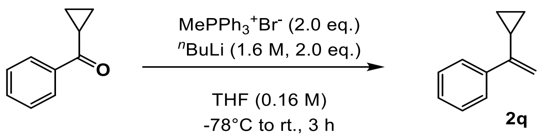

Cyclopropyl(phenyl)methanone (2q). An oven dried Schlenk flask was charged with methyltriphenylphosphonium bromide (3.57 g, 10 mmol, 2.0 equiv.), and placed under argon atmosphere. Anhydrous THF (30 mL) was added and the suspension was cooled to -78°C. While stirring, *n*BuLi (1.6 M in hexanes, 6.25 mL, 10 mmol, 2.0 equiv.) was added dropwise. After 10 minutes of stirring, cyclopropyl phenyl ketone (690 µl, 5.0 mmol, 1.0 equiv.) was added dropwise. After stirring of the solution for three hours at room temperature, the reaction mixture was quenched by the addition of NH_4Cl solution (satd.). The reaction mixture was transferred to a separatory funnel, and the phases were separated. The aqueous layer was extracted three times with pentane (10 mL each time). The combined organic layers were dried with Na_2SO_4, filtered and the solvent was removed *in vacuo*. The reaction mixture was purified by a silica plug filtration (with 200 mL of pentane) to give **2q** (767 mg, 5.32 mmol, 99%), a colourless oil. **R*f*** (pentane): 0.84. **^{1}H NMR** (400 MHz, $CDCl_3$) δ 7.67 – 7.59 (m, 2H), 7.42 – 7.34 (m, 2H), 7.32 – 7.24 (m, 1H), 5.31 (d, *J* = 1.1 Hz, 1H), 4.97 (t, *J* = 1.2 Hz, 1H), 0.90 – 0.84 (m, 2H), 0.68 – 0.58 (m, 2H). **GCMS (EI)** *m/z* calculated for $[M]^+$ ($C_{11}H_{12}^+$): 144.1, found: 144.1 (r_t = 2.984 min). Spectroscopic data are in agreement with those in the literature.[39]

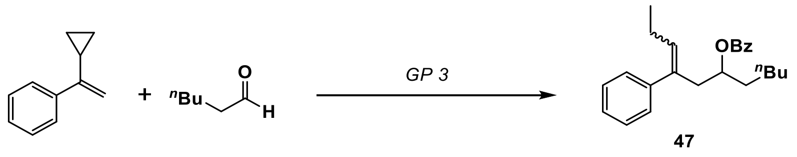

4-Phenylundec-3-en-6-yl benzoate (47). Following **GP3**, using hexanal (36.9 µl, 0.30 mmol, 1.5 equiv.) and alkene **2q** (28.8 mg, 0.2 mmol, 1.0 equiv.). The reaction

mixture was purified by column chromatography (pentane/Et_2O = 99/1 → 98.7/1.3), followed by preparative TLC (pentane/$CHCl_2$ = 70/30), to a afford a colourless oil (3.4 mg, 1:1:0.3 mixture of **47**: hexyl benzoate : hexyl hexanoate). **HRMS (ESI):** *m*/*z* calculated for $[M + Na]^+$ ($C_{24}H_{30}NaO_2^+$): 373.21435, found 373.21386.

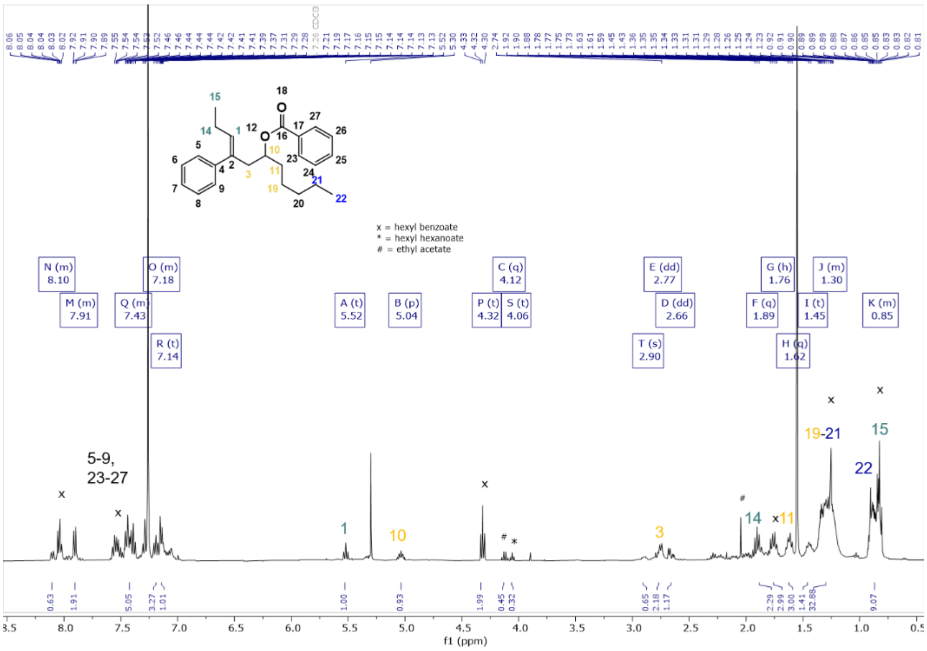

Supplementary Figure 6. ^{1}H NMR for ring-opening product **47.**

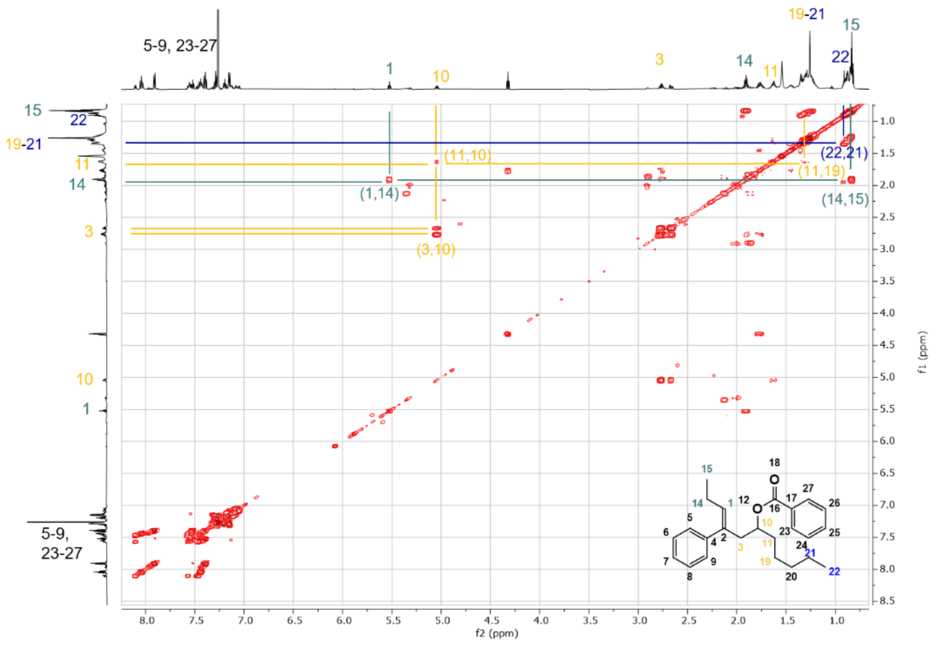

Supplementary Figure 7. ^{1}H-^{1}H COSY for ring-opening product **47.**

4.2. Computional study

All computations were carried out using the ORCA 5.0.2 software package.[40] The geometries were optimized with the CAM-B3LYP range-separated hybrid functional[41] on a recontracted Ahlrichs double-ζ-basis (def2-SVP),[42] applying the CPCM continuum solvation model for acetonitrile[43,44] and a Grimme D3 atom-pairwise dispersion correction[45] was applied. Optimized geometries were characterized to be minima or transition states on the respective potential energy surface by harmonic frequency analysis on the same level of theory. In most cases geometry was preoptimized by molecular dynamic simulations while the energy of multiple conformers and rotamers was evaluated. Relaxed surface scans were carried out to investigate kinetical barriers of bond break and formation as well as to preoptimize transition state geometries. For further energy evaluation single point calculations were performed on the optimized geometries using the CAM-B3LYP range-separated hybrid functional on a recontracted triple-ζ-basis set (def2-TZVPP) with the CPCM for acetonitrile and a Grimme D3 atom-pairwise dispersion correction. No symmetry or internal coordinate constraints were applied. Optimized structures were visualized using Cylview

(http://www.cylview.org). Redox potentials were determined adapting a protocol reported by Nicewicz and coworkers[46] using the CAM-B3LYP range-separated hybrid functional on a triple-ζ-basis set (def2-TZVPP) with the CPCM model for acetonitrile. Gibbs free energies at 298 K (G_{298}) were obtained as the sum of the electronic energies (SCF energies) and the corresponding free energy corrections (ZPVE, thermal corrections, enthalpy correction, entropic corrections), as obtained from the frequency calculation.

Redox potentials were then calculated from the Gibbs free energies of the respective oxidized and reduced species; the values were referenced to saturated calomel electrode (SCE) by subtraction of its absolute potential in acetonitrile (E_{ref} = 4.42 V).[46]

Stationary point energies of relevant structures (in E_h)

	Electronic Energy / E_h	Thermal Corr. / E_h	ZPVE / E_h	Enthalpy Corr. / E_h	Entropy Corr. / E_h
**I**	-1593.220197	0.02749625	0.34935184	0.00094421	0.08090993
**AcO^-**	-228.640597	0.00441157	0.04879879	0.00094421	0.03227201
**AcOH**	-229.111793	0.00452917	0.06197732	0.00094421	0.03228567
**TMS_3SiO^-**	-1592.718170	0.02713957	0.33691068	0.00094421	0.08025384
**$TMS_3SiOH^{•+}$**	-1592.967338	0.02735224	0.33915769	0.00094421	0.08132730
**II**	-1592.554843	0.02701956	0.33842246	0.00094421	0.08074414
**III**	-1592.624344	0.02680120	0.33915641	0.00094421	0.0817518
**IV**	-3230.330249	0.01658128	0.27772043	0.00094421	0.06185547
**V**	-4167.018997	0.02852949	0.34100112	0.00094421	0.08416535
**VI**	-655.9796848	0.01552121	0.27316233	0.00094421	0.05927661
**VII**	-761.2507913	0.01227055	0.17330163	0.00094421	0.05116935
**VIII**	-1417.290074	0.02803961	0.45209531	0.00094421	0.08459977
**IX**	-1417.406223	0.02834503	0.44972743	0.00094421	0.08477355
**X**	-1317.422104	0.02711497	0.44974862	0.00094421	0.0820696
**F^-**	-99.997216	0.00141627	0.00000000	0.00094421	0.01651905

**TS1**	-1592.551396	0.02612988	0.33859807	0.00094421	0.07904790
**TS2**	-4822.960484	0.04421532	0.61595904	0.00094421	0.11695623
**TS3**	-1417.236951	0.02843521	0.44819865	0.00094421	0.08577957
**TS4**	-1417.407391	0.02800603	0.44997725	0.00094421	0.08370825
**VIII_{ester}**	-1530.656018	0.029899	0.462662	0.000944	0.088506
**IX_{ester}**	-1530.793746	0.030054	0.461295	0.000944	0.088671
**X_{ester}**	-1430.790413	0.029008	0.460383	0.000944	0.086143
**XI**	-1416.300404	0.027912	0.465841	0.000944	0.083564
**XI_{ester}**	-1529.367986	0.030162	0.475785	0.000944	0.088968
**$TS4_{ester}$**	-1529.028706	0.029804	0.461291	0.000944	0.087585

4.2.1 Cartesian coordinates of computed structures

I

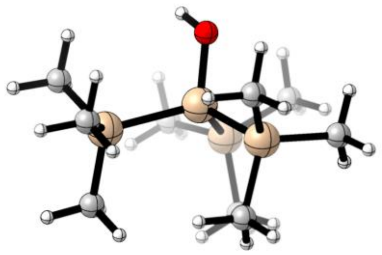

Charge: 0 , Multiplicity: 1

# of Imaginary Frequencies: 0

G_{sol}: -999574.672 kcal mol^{-1}

O	1.174234	0.104433	-0.096967
Si	2.885932	0.060546	-0.066464
Si	3.62228	0.506764	2.120445
C	2.554308	-0.498472	3.311455
C	5.434032	0.00763	2.307625
C	3.432553	2.338948	2.538245
Si	3.622602	-2.055081	-0.773542
C	2.598937	-2.567597	-2.275353
C	5.450864	-1.9767	-1.238438
C	3.380099	-3.330874	0.598875
Si	3.571003	1.743954	-1.543959
C	3.339377	1.2054	-3.33846
C	2.52813	3.282793	-1.217963
C	5.39505	2.121649	-1.238747
H	0.755625	-0.525406	0.504669
H	2.685183	-1.578846	3.146365
H	1.488253	-0.261381	3.166917
H	2.813103	-0.276502	4.359457
H	5.571338	-1.067036	2.111609
H	5.790658	0.215649	3.329385
H	6.072978	0.563852	1.60423
H	2.390095	2.666015	2.401192
H	4.069489	2.961735	1.891182
H	3.716728	2.532854	3.585322
H	2.786522	-1.896391	-3.127268
H	1.524148	-2.519615	-2.040397
H	2.837517	-3.596776	-2.588775
H	5.615926	-1.275917	-2.071569
H	5.817627	-2.968318	-1.549501
H	6.061567	-1.639887	-0.386631
H	2.328078	-3.366262	0.923181
H	3.995055	-3.08613	1.478727
H	3.664654	-4.338252	0.254066
H	2.291926	0.928699	-3.535003
H	3.969744	0.333263	-3.572037
H	3.613682	2.019598	-4.028686
H	1.458171	3.048336	-1.329725
H	2.780238	4.090242	-1.92424
H	2.686356	3.657553	-0.195463
H	6.015688	1.2242	-1.385789

H	5.559348	2.482702	-0.211778
H	5.751653	2.898717	-1.934055

AcO⁻

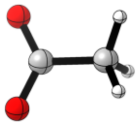

Charge: -1, Multiplicity: 1

# of Imaginary Frequencies: 0

G_{sol}: -143460.438 kcal mol^{-1}

O	1.138729	0.107084	-0.006617
C	2.387907	0.052598	0.043407
C	3.159951	1.013613	-0.883646
O	3.07786	-0.72408	0.740488
H	2.573302	1.918414	-1.098525
H	3.354472	0.50673	-1.843835
H	4.133943	1.285964	-0.451938

AcOH

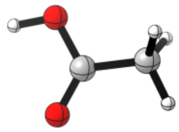

Charge: 0 , Multiplicity: 1

# of Imaginary Frequencies: 0

G_{sol}: -143747.783 kcal mol^{-1}

O	1.292357	-0.277571	0.099192
C	2.596245	-0.112615	-0.141100
C	3.056815	1.279341	0.170206
O	3.292964	-1.004251	-0.560614
H	1.056523	-1.196709	-0.119497
H	2.489102	2.000094	-0.43592
H	4.127989	1.372446	-0.037619
H	2.854757	1.507192	1.226824

TMS_3SiO^-

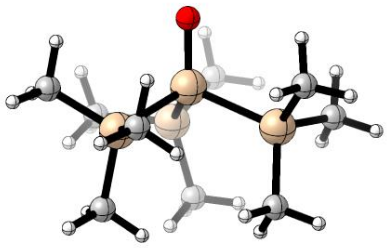

Charge: -1, Multiplicity: 1

# of Imaginary Frequencies: 0

G_{sol}: -999267.264 kcal mol^{-1}

O	1.045250	0.057738	-0.029731
Si	2.650736	0.059043	-0.051562
Si	3.592803	0.490024	2.073138
C	2.585095	-0.490396	3.349097
C	5.419123	-0.000559	2.249011
C	3.447380	2.323700	2.550831
Si	3.567138	-2.000403	-0.757857
C	2.568587	-2.600767	-2.257159
C	5.395446	-1.913337	-1.262734
C	3.406014	-3.337460	0.583297
Si	3.555659	1.696620	-1.492990
C	3.390386	1.208519	-3.321817
C	2.551049	3.290056	-1.255062
C	5.385783	2.091957	-1.176100
H	2.741129	-1.573748	3.225120
H	1.513139	-0.290925	3.190303
H	2.838384	-0.222539	4.388094
H	5.556307	-1.077218	2.061896
H	5.797870	0.219021	3.261106
H	6.046189	0.545705	1.526951
H	2.402691	2.663426	2.465650
H	4.056495	2.955021	1.884558
H	3.779978	2.502977	3.586900
H	2.715911	-1.932361	-3.120141
H	1.495157	-2.588212	-2.008756
H	2.841359	-3.623883	-2.564230
H	5.536824	-1.216078	-2.103537
H	5.774757	-2.900938	-1.573972
H	6.018868	-1.558811	-0.427050
H	2.360325	-3.421861	0.920543
H	4.018787	-3.087396	1.463955
H	3.726229	-4.327000	0.216822
H	2.344160	0.963670	-3.565721
H	4.001104	0.319728	-3.547605
H	3.714191	2.022424	-3.991679

H	1.477777	3.055799	-1.340161
H	2.798925	4.064423	-1.999451
H	2.716943	3.712145	-0.251300
H	6.010654	1.193677	-1.300535
H	5.535812	2.462218	-0.149847
H	5.757705	2.862132	-1.872235

$TMS_3SiOH^{\bullet+}$

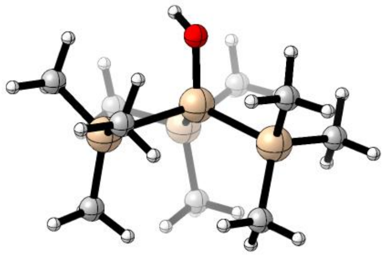

Charge: +1, Multiplicity: 2

# of Imaginary Frequencies: 0

G_{sol}: -999422.749 kcal mol^{-1}

O	1.257543	-0.063736	0.049595
Si	2.900146	0.219535	0.003125
Si	3.628546	0.434889	2.262729
C	2.464070	-0.566522	3.339613
C	5.418861	-0.100016	2.432335
C	3.451734	2.275448	2.617871
Si	3.696342	-2.118680	-0.823216
C	2.563667	-2.410816	-2.269715
C	5.497450	-1.905867	-1.229085
C	3.321880	-3.210110	0.635860
Si	3.555403	1.789786	-1.678508
C	3.235144	1.158709	-3.414685
C	2.476826	3.284565	-1.299712
C	5.372934	2.163980	-1.397541
H	0.740071	0.062170	-0.761299
H	2.573664	-1.647324	3.170027
H	1.417958	-0.288267	3.142017
H	2.679674	-0.361773	4.400843
H	5.554739	-1.172463	2.228715
H	5.754401	0.093310	3.464190
H	6.071273	0.468335	1.751877
H	2.414573	2.616282	2.479418
H	4.106973	2.877991	1.970546
H	3.738266	2.465754	3.665435
H	2.755762	-1.722639	-3.103339
H	1.509195	-2.334055	-1.968503

H	2.745889	-3.440959	-2.622782
H	5.647233	-1.225978	-2.079792
H	5.889311	-2.899547	-1.509315
H	6.073504	-1.543693	-0.365686
H	2.272677	-3.112507	0.949550
H	3.980341	-3.011072	1.492342
H	3.486593	-4.252611	0.311375
H	2.190542	0.838745	-3.546191
H	3.896053	0.317489	-3.671554
H	3.433427	1.974734	-4.128595
H	1.407693	3.037913	-1.384662
H	2.704023	4.084383	-2.023734
H	2.664494	3.674373	-0.288046
H	6.004093	1.282025	-1.582387
H	5.558658	2.516689	-0.371497
H	5.688726	2.959341	-2.092369

II

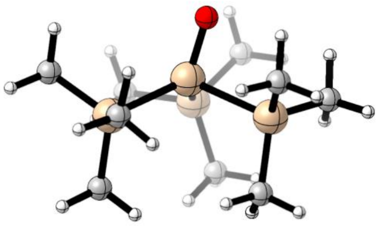

Charge: 0 , Multiplicity: 2

# of Imaginary Frequencies: 0

G_{sol}: -999164.210 kcal mol^{-1}

O	1.284805	-0.357341	-0.128928
Si	2.815754	0.143242	-0.022519
Si	3.631603	0.459392	2.175517
C	2.547449	-0.550535	3.337750
C	5.436999	-0.065931	2.313929
C	3.487641	2.293296	2.597522
Si	3.617421	-2.045002	-0.784796
C	2.581458	-2.511855	-2.271753
C	5.434934	-1.868168	-1.220588
C	3.348578	-3.232785	0.6388
Si	3.546461	1.753833	-1.586127
C	3.312569	1.150732	-3.354969
C	2.532322	3.320506	-1.314217
C	5.368036	2.109767	-1.250953
H	2.654741	-1.62849	3.146818
H	1.488528	-0.285368	3.196249
H	2.814890	-0.358457	4.389345

H	5.561254	-1.142276	2.121645
H	5.815233	0.143884	3.327572
H	6.065295	0.483843	1.596207
H	2.445618	2.638762	2.515393
H	4.103815	2.905787	1.921146
H	3.830125	2.476449	3.629097
H	2.700421	-1.785242	-3.088086
H	1.519572	-2.545173	-1.990578
H	2.883555	-3.504057	-2.644603
H	5.577237	-1.189341	-2.074161
H	5.831039	-2.858784	-1.500568
H	6.021005	-1.491092	-0.370206
H	2.297357	-3.216409	0.961078
H	3.983212	-2.984718	1.501680
H	3.598031	-4.256300	0.313904
H	2.267796	0.853152	-3.532743
H	3.957198	0.285077	-3.570618
H	3.566962	1.951424	-4.068013
H	1.462660	3.128040	-1.489160
H	2.856968	4.114364	-2.006451
H	2.647717	3.694156	-0.285265
H	5.985124	1.208604	-1.384870
H	5.518380	2.476331	-0.223720
H	5.738354	2.882813	-1.943720

III

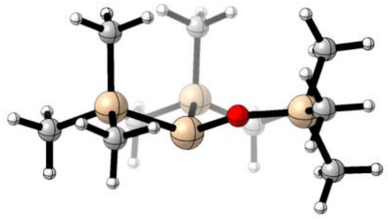

Charge: 0 , Multiplicity: 2

# of Imaginary Frequencies: 0

G_{sol}: -999208.131 kcal mol^{-1}

C	1.009274	-0.445978	-0.102229
Si	2.858319	-0.174316	0.147329
Si	3.893200	-0.436356	-1.943127
Si	6.240532	-0.238190	-1.990443
C	7.046406	-1.550746	-0.897389
C	6.921765	-0.393781	-3.742207
C	6.654854	1.473035	-1.313002
O	3.340975	-1.930436	-2.490123
Si	3.518666	-3.076299	-3.698154
C	3.701269	-2.198682	-5.34214
C	5.026906	-4.121861	-3.324915
C	1.973936	-4.127931	-3.670186
C	3.181856	1.538044	0.862941
C	3.514326	-1.491952	1.331832
H	0.573813	0.345219	-0.731792
H	0.477797	-0.450918	0.863019
H	0.829213	-1.411867	-0.599311
H	6.788070	-2.565228	-1.236155
H	6.716938	-1.447194	0.147854
H	8.144061	-1.450345	-0.923687
H	6.746984	-1.396845	-4.159031
H	8.009482	-0.215828	-3.737060
H	6.457270	0.341583	-4.416778
H	6.302936	1.590613	-0.276576
H	6.193565	2.266472	-1.920909
H	7.745942	1.627772	-1.320737
H	4.584596	-1.541892	-5.343630
H	2.815427	-1.579483	-5.553391
H	3.815562	-2.922759	-6.164297
H	5.949799	-3.523172	-3.350813
H	5.128750	-4.931626	-4.064762
H	4.945876	-4.580480	-2.326959
H	1.079251	-3.515445	-3.862246
H	1.851128	-4.615759	-2.690621
H	2.020994	-4.914670	-4.439782
H	2.823592	2.327520	0.184650
H	4.256822	1.699553	1.036192
H	2.661377	1.653868	1.827357
H	3.379319	-2.498083	0.905278
H	2.976255	-1.452108	2.293252
H	4.587308	-1.348778	1.531590

IV

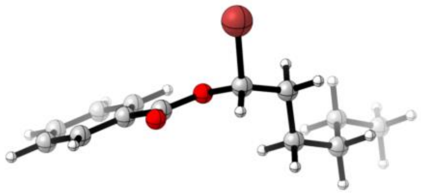

Charge: 0 , Multiplicity: 1

# of Imaginary Frequencies: 0

G_{sol}: -2026916.788 kcal mol^{-1}

Br	0.394848	0.703875	-1.319518
C	1.798542	1.508499	-0.181252

O	1.915486	0.759508	0.992134
C	1.088837	1.036946	2.024395
C	1.359526	0.181789	3.205599
C	2.337318	-0.818228	3.187440
C	2.554864	-1.591075	4.323236
C	1.801260	-1.368170	5.474980
C	0.825191	-0.371790	5.494082
C	0.603150	0.401607	4.360988
O	0.242704	1.891584	1.965749
C	3.127637	1.513365	-0.894351
C	4.199807	2.238353	-0.081837
C	5.567815	2.260420	-0.760343
C	6.243587	0.895356	-0.860800
C	7.627729	0.961592	-1.493514
H	1.416862	2.509786	0.044876
H	2.921935	-0.987954	2.283459
H	3.317034	-2.372676	4.309987
H	1.975618	-1.976660	6.365123
H	0.235618	-0.198504	6.396452
H	-0.155722	1.185354	4.356295
H	2.994117	2.007516	-1.867838
H	3.415864	0.470429	-1.092950
H	4.291022	1.761597	0.907829
H	3.864270	3.272543	0.100477
H	6.228481	2.940787	-0.197088
H	5.470587	2.697293	-1.770085
H	6.316703	0.459681	0.150916
H	5.611286	0.204936	-1.442998
H	8.297989	1.618917	-0.916875
H	7.575888	1.361190	-2.518879
H	8.096758	-0.032434	-1.545816

V

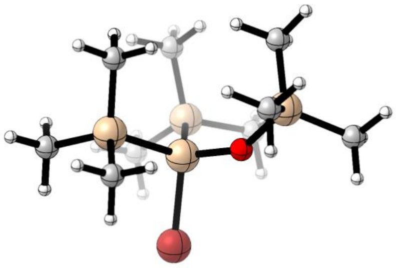

Charge: 0 , Multiplicity: 1

# of Imaginary Frequencies: 0

G_{sol}: -2614664.762 kcal mol^{-1}

Br	-2.028430	4.131284	-0.599618
Si	0.232456	4.368806	-0.991702
Si	0.519860	3.647491	-3.209623
C	-0.874833	4.345394	-4.263613
C	2.173965	4.272046	-3.859840
C	0.478005	1.765540	-3.191096
Si	0.703806	6.608422	-0.465090
C	0.187295	6.938154	1.313847
C	2.551109	6.909719	-0.686646
C	-0.272037	7.691959	-1.656073
O	0.944419	3.312457	0.068707
Si	2.366287	2.753562	0.762365
C	3.739125	2.893145	-0.501955
C	2.741472	3.800248	2.266816
C	2.058942	0.974151	1.238160
H	-0.875328	5.446226	-4.234863
H	-1.854366	3.994885	-3.906184
H	-0.756506	4.031553	-5.313196
H	2.203175	5.372791	-3.862436
H	2.330720	3.925004	-4.893833
H	3.011506	3.909107	-3.246304
H	-0.482788	1.401639	-2.795454
H	1.282056	1.364752	-2.554781
H	0.608720	1.35953	-4.206874
H	0.739769	6.287223	2.008189
H	-0.888311	6.748164	1.448437
H	0.389634	7.985632	1.589194
H	3.146714	6.29005	0.000754
H	2.791413	7.965922	-0.484320
H	2.868291	6.678579	-1.715201
H	-1.352670	7.505756	-1.561292
H	0.018673	7.492817	-2.699456
H	-0.086762	8.757983	-1.448176
H	3.872477	3.937542	-0.824605
H	3.517241	2.28488	-1.392523
H	4.694762	2.543612	-0.080276
H	2.917299	4.850454	1.987879
H	3.642222	3.4303	2.781685
H	1.903751	3.772242	2.980928
H	1.814000	0.371312	0.349851
H	1.218128	0.899449	1.945235
H	2.948049	0.533991	1.716460

VI

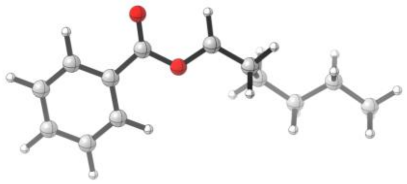

Charge: 0 , Multiplicity: 2

# of Imaginary Frequencies: 0

G_{sol}: -411489.002 kcal mol^{-1}

C	5.173358	-3.199507	-0.510309
H	4.500488	-3.614412	-1.260819
O	4.637027	-2.247809	0.315773
C	3.436663	-1.706856	0.032062
C	3.015450	-0.704277	1.042269
C	1.789051	-0.059066	0.854319
C	1.350549	0.885294	1.775032
C	2.134967	1.188002	2.887892
C	3.358001	0.546029	3.078600
C	3.801133	-0.398563	2.158748
O	2.794747	-2.018382	-0.941760
C	6.402830	-3.844697	0.001569
C	6.178174	-4.699752	1.262107
C	7.462472	-5.275445	1.853005
C	8.170030	-6.296268	0.965870
C	9.417814	-6.883429	1.612944
H	1.188542	-0.310203	-0.021187
H	0.393176	1.388731	1.626150
H	1.790735	1.929980	3.611786
H	3.971216	0.783694	3.950156
H	4.757004	-0.902655	2.301271
H	6.822371	-4.459912	-0.807629
H	7.154197	-3.068295	0.235965
H	5.478269	-5.518697	1.023010
H	5.677561	-4.074731	2.018737
H	7.226058	-5.750791	2.820283
H	8.158633	-4.449072	2.083560
H	8.444961	-5.830018	0.005458
H	7.462294	-7.106581	0.718186
H	9.906369	-7.619726	0.957054
H	9.174465	-7.388489	2.561437
H	10.154497	-6.095659	1.838351

VII

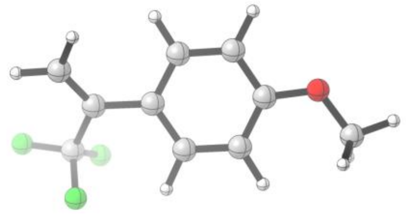

Charge: 0 , Multiplicity: 1

# of Imaginary Frequencies: 0

G_{sol}: -477607.248 kcal mol^{-1}

C	3.057435	0.873533	1.237798
C	2.486258	0.501501	0.089666
C	1.109882	-0.112467	0.136372
F	0.286309	0.442907	-0.763024
F	1.141298	-1.430832	-0.138343
F	0.527178	0.01124	1.331025
C	3.109136	0.66378	-1.247122
C	3.998512	1.72482	-1.485389
C	4.609731	1.889645	-2.715535
C	4.344005	0.997961	-3.763898
O	4.97561	1.235478	-4.930139
C	4.719902	0.390028	-6.031339
C	3.454794	-0.057909	-3.549735
C	2.850818	-0.214347	-2.3043
H	2.538088	0.768012	2.190673
H	4.065538	1.291276	1.253795
H	4.203708	2.448347	-0.694288
H	5.294662	2.719918	-2.896513
H	5.319602	0.771828	-6.866077
H	3.654461	0.407479	-6.313457
H	5.016073	-0.650074	-5.818928
H	3.224443	-0.769543	-4.341272
H	2.172097	-1.055881	-2.166362

VIII

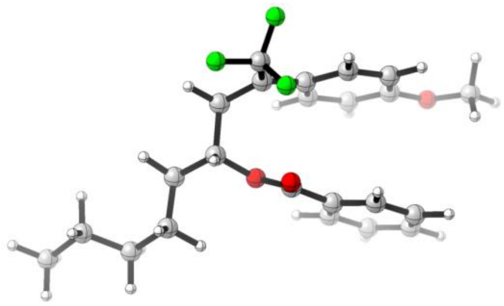

Charge: 0 , Multiplicity: 2

# of Imaginary Frequencies: 0

G_{sol}: -889114.333 kcal mol^{-1}

F	5.408528	4.116197	2.258375
C	5.502231	2.802749	1.968635
F	4.377978	2.501944	1.287044
F	6.510131	2.675579	1.093883
C	5.707564	1.9643	3.189682

C	4.746507	2.018786	4.244797
C	4.950619	1.312772	5.463946
C	3.989226	1.267506	6.447188
C	2.754813	1.914213	6.271712
C	2.538697	2.66052	5.105665
C	3.515647	2.717196	4.124962
C	6.839239	0.982831	3.166876
C	6.460274	-0.388008	2.57962
H	6.127916	-0.270253	1.539366
O	5.361319	-0.921238	3.338656
C	4.111434	-0.762569	2.897292
C	3.098961	-1.159207	3.914535
C	1.778979	-0.745239	3.722294
C	0.811332	-1.032555	4.679194
C	1.157564	-1.748206	5.82446
C	2.471248	-2.178455	6.01025
C	3.443464	-1.877857	5.062535
O	3.835574	-0.320467	1.806755
C	7.598121	-1.386901	2.663643
C	7.277997	-2.721916	1.999738
C	8.328499	-3.804643	2.23428
C	9.707247	-3.498449	1.656277
C	10.696472	-4.641356	1.847895
H	5.88044	0.771207	5.628208
H	4.154753	0.703241	7.366327
H	1.600124	3.189337	4.945843
H	3.296607	3.290908	3.226175
H	7.229099	0.821987	4.18081
H	7.670842	1.373326	2.566584
H	1.528316	-0.179482	2.823968
H	-0.216675	-0.694304	4.534017
H	0.39892	-1.971771	6.577804
H	2.74128	-2.741857	6.90572
H	4.477181	-2.188982	5.212511
H	8.482023	-0.92417	2.199053
H	7.846059	-1.542914	3.727646
H	7.145049	-2.562739	0.915337
H	6.308308	-3.080505	2.379851
H	7.964512	-4.751238	1.799663
H	8.424349	-3.986553	3.31985
H	10.112651	-2.58455	2.121327
H	9.604356	-3.272115	0.580544
H	11.683445	-4.400065	1.42509
H	10.338626	-5.562106	1.359917
H	10.836936	-4.868527	2.916893
O	1.852463	1.761891	7.256757
C	0.563408	2.31876	7.10162
H	0.604412	3.417076	7.024937
H	0.059765	1.912727	6.209579
H	-0.006644	2.041694	7.996152

IX

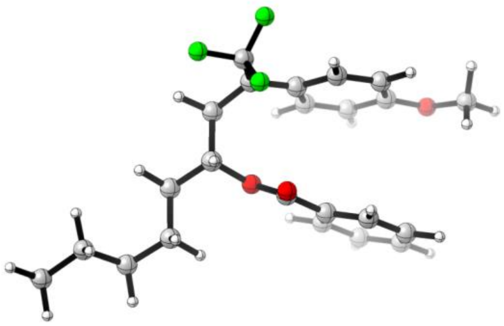

Charge: -1, Multiplicity: 1

# of Imaginary Frequencies: 0

G_{sol}: -889188.621 kcal mol^{-1}

F	5.623957	4.077543	2.293526
C	5.964117	2.758411	2.132054
F	5.112330	2.395823	1.066202
F	7.184033	2.773059	1.533160
C	5.91962	1.962413	3.329834
C	4.727960	1.990816	4.141533
C	4.665908	1.344036	5.409153
C	3.526718	1.334735	6.194465
C	2.354001	1.971569	5.777430
C	2.372545	2.635919	4.552307
C	3.523507	2.640633	3.762093
C	6.905943	0.823940	3.422640
C	6.479958	-0.484743	2.743650
H	6.148899	-0.27497	1.717756
O	5.342581	-1.015273	3.461032
C	4.129297	-0.981753	2.928028
C	3.074802	-1.415533	3.891204
C	1.737945	-1.212301	3.542103
C	0.723442	-1.551964	4.431568
C	1.042153	-2.11133	5.668408
C	2.375517	-2.333032	6.011956
C	3.390654	-1.978518	5.129871
O	3.887502	-0.639911	1.791671
C	7.554877	-1.556087	2.730432
C	7.105580	-2.86723	2.092819
C	8.207245	-3.916727	1.959918
C	9.303660	-3.561041	0.958765
C	10.339737	-4.665519	0.793360
H	5.543836	0.810944	5.777086
H	3.518914	0.807007	7.151529

H	1.482427	3.143859	4.178169
H	3.469385	3.143237	2.796597
H	7.150693	0.579976	4.470878
H	7.862438	1.109877	2.959714
H	1.508524	-0.769654	2.571819
H	-0.320236	-1.378832	4.160867
H	0.246810	-2.377174	6.368415
H	2.625274	-2.773893	6.979368
H	4.435996	-2.124408	5.400565
H	8.415697	-1.138629	2.185332
H	7.898711	-1.740010	3.763603
H	6.682821	-2.657011	1.094171
H	6.279609	-3.283471	2.690845
H	7.752356	-4.874871	1.655680
H	8.662540	-4.099864	2.949934
H	9.808813	-2.631745	1.269462
H	8.837137	-3.339447	-0.017151
H	11.115165	-4.389733	0.062760
H	9.870715	-5.601066	0.448461
H	10.84299	-4.882600	1.749179
O	1.264406	1.878522	6.606931
C	0.039896	2.372273	6.136411
H	0.071504	3.461787	5.957926
H	-0.266843	1.878293	5.196932
H	-0.711746	2.16416	6.909191

X

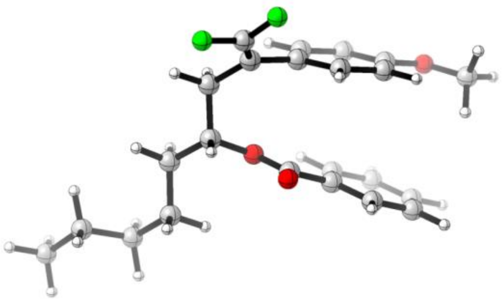

Charge: 0 , Multiplicity: 1

# of Imaginary Frequencies: 0

G_{sol}: -826446.688 kcal mol^{-1}

C	5.956049	2.157126	3.282806
C	4.872817	2.129741	4.299457
C	5.153833	1.786344	5.631297
C	4.147910	1.693208	6.576861
C	2.814607	1.931023	6.219737
C	2.516897	2.294336	4.905758
C	3.541221	2.385009	3.964869
C	7.002425	1.068905	3.263997
C	6.510629	-0.261723	2.696843
H	6.147598	-0.115586	1.671063
O	5.399927	-0.721052	3.490200
C	4.162715	-0.665138	2.998279
C	3.135401	-1.080077	3.992446
C	1.795419	-1.074229	3.595976
C	0.798724	-1.416681	4.501800
C	1.138394	-1.763951	5.809961
C	2.473924	-1.773896	6.207137
C	3.473524	-1.434237	5.300411
O	3.901140	-0.308075	1.871980
C	7.578777	-1.339435	2.732332
C	7.111151	-2.675953	2.166430
C	8.133246	-3.802021	2.301282
C	9.416041	-3.603171	1.498871
C	10.375229	-4.780886	1.614931
H	6.180724	1.576678	5.935674
H	4.368882	1.413051	7.608341
H	1.490902	2.479871	4.591174
H	3.282398	2.633781	2.934819
H	7.374526	0.895847	4.284982
H	7.864854	1.384457	2.662334
H	1.550421	-0.788720	2.572206
H	-0.247921	-1.410037	4.190728
H	0.355110	-2.027017	6.524392
H	2.738231	-2.041736	7.232090
H	4.519428	-1.429630	5.604098
H	8.445911	-0.960638	2.170104
H	7.912348	-1.471121	3.776326
H	6.840331	-2.546964	1.103776
H	6.186747	-2.971565	2.686968
H	7.662363	-4.748510	1.985542
H	8.389768	-3.932471	3.367958
H	9.926863	-2.684344	1.831093
H	9.154408	-3.439159	0.438908
H	11.291656	-4.617852	1.027936
H	9.906837	-5.711186	1.255527
H	10.673944	-4.945956	2.662559
C	6.008470	3.117387	2.364608
F	5.179748	4.138447	2.268831
F	6.918790	3.199949	1.412114
O	1.894579	1.774082	7.195812
C	0.529942	1.932509	6.876365
H	0.310853	2.956100	6.530762
H	0.214502	1.216595	6.100180
H	-0.033268	1.735583	7.796540

F⁻

Charge: -1, Multiplicity: 1

# of Imaginary Frequencies: 1

G_{sol}: -62758.098 kcal mol^{-1}

F	0.000000	0.000000	0.000000

TS1

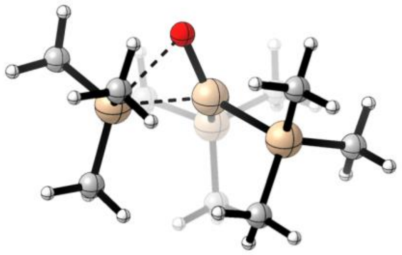

Charge: 0 , Multiplicity: 2

# of Imaginary Frequencies: 1 (-244.78 cm^{-1})

G_{sol}: -889083.935 kcal mol^{-1}

O	1.666458	-0.792956	-0.284151
Si	2.963779	0.158809	-0.029945
Si	3.66183	0.519674	2.197546
C	2.536097	-0.47441	3.33236
C	5.451586	-0.046083	2.375048
C	3.545792	2.357432	2.603892
Si	3.538195	-2.086536	-0.785481
C	2.693022	-2.587615	-2.379008
C	5.404696	-2.024115	-1.128828
C	3.225143	-3.28536	0.619805
Si	3.52197	1.83751	-1.582288
C	3.180047	1.229644	-3.330869
C	2.517559	3.392363	-1.223901
C	5.35885	2.194043	-1.351064
H	2.593413	-1.548733	3.10293
H	1.488502	-0.157709	3.21401
H	2.821826	-0.331627	4.386985
H	5.548297	-1.126477	2.188988
H	5.817848	0.160231	3.393751
H	6.105744	0.482274	1.664001
H	2.513556	2.724699	2.496922
H	4.189434	2.950191	1.935496
H	3.870742	2.541609	3.640811
H	2.812663	-1.810709	-3.148648
H	1.619319	-2.755544	-2.222071
H	3.150302	-3.514912	-2.76094
H	5.634889	-1.321766	-1.94436
H	5.747656	-3.028585	-1.431872
H	5.970982	-1.717445	-0.237352
H	2.158228	-3.320088	0.878021
H	3.793597	-3.002311	1.518436
H	3.552337	-4.294631	0.320868
H	2.133537	0.903642	-3.432285
H	3.828764	0.377496	-3.585076
H	3.364324	2.031091	-4.064135
H	1.438872	3.200773	-1.330995
H	2.794378	4.196762	-1.924706
H	2.700789	3.752302	-0.200076
H	5.96399	1.295505	-1.546979
H	5.571857	2.532197	-0.324841
H	5.687248	2.985491	-2.04396

TS2

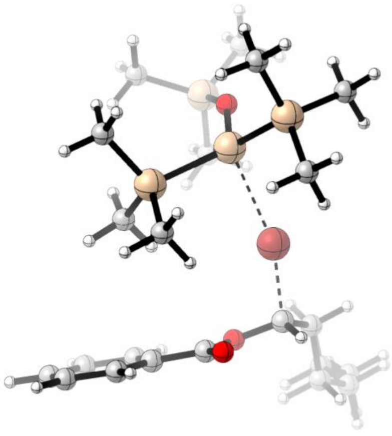

Charge: 0 , Multiplicity: 2

# of Imaginary Frequencies: 1 (-204.26 cm^{-1})

G_{sol}: -889188.741 kcal mol^{-1}

Br	0.530431	0.78273	-2.139301
C	1.809226	1.550106	-0.630035
O	1.428962	0.892707	0.536579
C	0.309742	1.29127	1.171949
C	-0.104301	0.343691	2.234526
C	0.626082	-0.815519	2.51535
C	0.175549	-1.695998	3.492824
C	-1.002126	-1.424438	4.188502
C	-1.730946	-0.267899	3.910538
C	-1.282079	0.616492	2.936695

O	-0.287347	2.296138	0.871802
C	3.239903	1.243837	-0.958714
C	4.20727	1.809421	0.084113
C	5.676599	1.526844	-0.222623
C	6.071018	0.054104	-0.146493
C	7.558455	-0.17389	-0.384836
H	1.541221	2.611159	-0.600872
H	1.539789	-1.025778	1.95981
H	0.744281	-2.602027	3.711108
H	-1.355159	-2.120207	4.952865
H	-2.653297	-0.057249	4.455376
H	-1.84136	1.523021	2.701015
H	3.462885	1.67236	-1.947646
H	3.350921	0.152294	-1.045648
H	3.953079	1.396216	1.074189
H	4.051659	2.898514	0.153384
H	6.299517	2.095434	0.488564
H	5.924032	1.919449	-1.224947
H	5.7883	-0.34057	0.844954
H	5.491673	-0.528949	-0.88122
H	8.1671	0.370891	0.354593
H	7.858997	0.180323	-1.383993
H	7.821833	-1.240119	-0.315368
C	-1.188901	1.146115	-6.435124
Si	-2.423995	1.13844	-5.012766
Si	-1.687024	-0.487538	-3.497102
Si	-2.84806	-0.903557	-1.492297
C	-4.458843	-1.82388	-1.836897
C	-1.781332	-1.916779	-0.314208
C	-3.225133	0.769522	-0.712284
O	-1.277496	-1.859531	-4.363276
Si	-0.585508	-3.382221	-4.201367
C	0.847768	-3.261852	-3.003948
C	-1.895362	-4.565295	-3.578653
C	0.000881	-3.883846	-5.902353
C	-2.455917	2.815697	-4.157048
C	-4.142841	0.711918	-5.663928
H	-0.186682	1.433344	-6.081718
H	-1.496678	1.856439	-7.219267
H	-1.115728	0.144791	-6.886813
H	-4.265201	-2.790119	-2.326659
H	-5.116893	-1.234745	-2.494296
H	-4.999249	-2.019886	-0.896227
H	-1.615127	-2.936997	-0.690573
H	-2.276099	-1.992863	0.667794
H	-0.801625	-1.440392	-0.164974
H	-3.866115	1.381412	-1.365256
H	-2.302943	1.331945	-0.505761
H	-3.754632	0.621035	0.24301
H	0.509347	-2.933106	-2.009814
H	1.593097	-2.536053	-3.364482
H	1.344967	-4.238017	-2.889907
H	-2.250987	-4.280454	-2.576956
H	-1.496867	-5.590332	-3.516996
H	-2.762365	-4.576205	-4.257762
H	0.751684	-3.173907	-6.282883
H	-0.839013	-3.909588	-6.614086
H	0.458069	-4.885632	-5.878816
H	-1.462286	3.07263	-3.759992
H	-3.169612	2.823675	-3.318996
H	-2.757341	3.601216	-4.868641
H	-4.143714	-0.275503	-6.150763
H	-4.472536	1.459629	-6.403948
H	-4.88066	0.689624	-4.847106

TS3

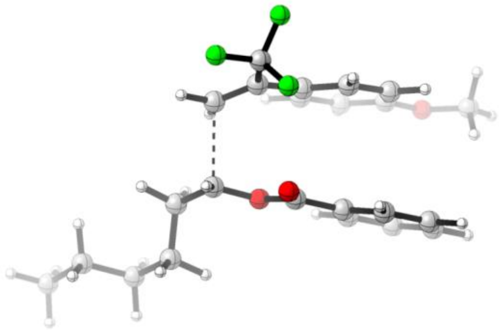

Charge: 0 , Multiplicity: 2

# of Imaginary Frequencies: 1 (-202.31 cm^{-1})

G_{sol}: -999161.430 kcal mol^{-1}

F	5.010486	4.396285	2.288807
C	5.243118	3.136796	1.876607
F	4.116555	2.736906	1.255579
F	6.184076	3.209712	0.928190
C	5.640393	2.249543	3.020922
C	4.733282	2.169838	4.181781
C	5.192835	1.664118	5.413770
C	4.346414	1.507497	6.493745
C	2.991727	1.853263	6.390757
O	2.228916	1.627927	7.480185
C	0.859270	1.964324	7.434235
C	2.520934	2.393834	5.194475
C	3.384066	2.542712	4.111984

C	6.792585	1.539914	2.909650
C	6.219632	-0.672474	2.219463
H	6.016810	-0.458929	1.168473
O	5.101786	-0.897968	2.979983
C	3.885820	-0.564245	2.500454
C	2.804200	-0.794664	3.488929
C	1.489912	-0.524792	3.095616
C	0.442735	-0.712960	3.988833
C	0.704958	-1.169465	5.281234
C	2.013853	-1.437920	5.676628
C	3.064163	-1.252597	4.783581
O	3.721793	-0.139682	1.382776
C	7.395950	-1.476393	2.637870
C	7.228471	-2.976778	2.350637
C	8.414320	-3.829752	2.795445
C	9.703440	-3.585473	2.015636
C	10.839509	-4.506887	2.442474
H	6.240551	1.385601	5.532463
H	4.712208	1.105840	7.440323
H	0.435657	1.688799	8.407428
H	0.334998	1.407478	6.641384
H	0.71518	3.044656	7.269395
H	1.476127	2.677539	5.078411
H	2.968129	2.934330	3.185197
H	7.195114	0.998897	3.763144
H	7.463140	1.696461	2.065883
H	1.305810	-0.163292	2.082977
H	-0.582911	-0.501738	3.679651
H	-0.117711	-1.315756	5.984731
H	2.219076	-1.786119	6.690778
H	4.090065	-1.453258	5.089410
H	8.278986	-1.080537	2.114256
H	7.570310	-1.336092	3.718691
H	7.054012	-3.120813	1.270565
H	6.316679	-3.324901	2.861709
H	8.139222	-4.893789	2.698092
H	8.600865	-3.662062	3.871278
H	10.02118	-2.536569	2.135251
H	9.500840	-3.720024	0.938809
H	11.756844	-4.313073	1.866427
H	10.569255	-5.564937	2.296406
H	11.080173	-4.372952	3.509229

TS4

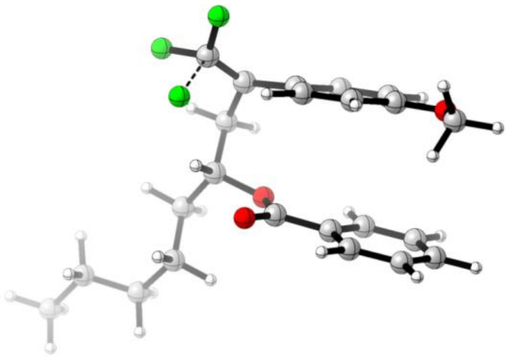

Charge: -1, Multiplicity: 1

# of Imaginary Frequencies: 1 (-252.71 cm^{-1})

G_{sol}: -3026112.537 kcal mol^{-1}

F	5.96437	4.04484	2.684266
C	6.573983	2.876056	2.84994
F	5.757705	2.130413	1.293088
F	7.773706	2.923473	2.275012
C	6.350938	2.095093	3.933774
C	5.018263	2.132971	4.575947
C	4.856695	1.958636	5.960689
C	3.600504	1.929805	6.547453
C	2.450024	2.078666	5.765253
C	2.583809	2.269975	4.38942
C	3.853496	2.282261	3.811058
C	7.204563	0.856771	4.064038
C	6.647812	-0.317341	3.255175
H	6.261868	0.076888	2.30538
O	5.522902	-0.838402	4.005734
C	4.338819	-0.985216	3.427602
C	3.261041	-1.310795	4.406216
C	1.955472	-1.445288	3.926899
C	0.909878	-1.696597	4.807757
C	1.167185	-1.82022	6.173602
C	2.469971	-1.698231	6.653254
C	3.516517	-1.440976	5.77277
O	4.137797	-0.86375	2.239276
C	7.648301	-1.434411	3.02501
C	7.075974	-2.645795	2.295782
C	8.113381	-3.711703	1.949643
C	9.104677	-3.303113	0.863056
C	10.09607	-4.406492	0.51664
H	5.740846	1.831249	6.590841
H	3.482124	1.779151	7.622613
H	1.706614	2.374364	3.75118
H	3.975792	2.361124	2.728402
H	7.307341	0.536984	5.114322
H	8.220642	1.061642	3.697212

H	1.775296	-1.336212	2.856733
H	-0.11038	-1.794072	4.430972
H	0.346076	-2.013448	6.867641
H	2.670582	-1.794123	7.722374
H	4.535508	-1.326749	6.139695
H	8.480697	-0.999946	2.449618
H	8.070774	-1.749713	3.995448
H	6.570523	-2.310282	1.37416
H	6.292624	-3.097298	2.925278
H	7.591793	-4.626941	1.621831
H	8.668608	-3.99115	2.863014
H	9.657787	-2.402721	1.177681
H	8.541736	-3.011406	-0.040736
H	10.789562	-4.095579	-0.279283
H	9.575328	-5.31418	0.171668
H	10.69937	-4.686863	1.39486
O	1.259716	2.006939	6.418956
C	0.07384	2.063277	5.663833
H	-0.031357	3.027338	5.138033
H	0.027836	1.24877	4.921757
H	-0.758159	1.949292	6.370001

XI

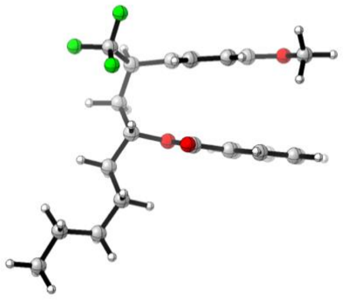

Charge: 0 , Multiplicity: 1

# of Imaginary Frequencies: 0

G_{sol}: --889510.848 kcal mol^{-1}

F	5.87544	4.29945	2.28938
C	6.50623	3.12205	2.36056
F	6.03672	2.36653	1.34901
F	7.79774	3.35533	2.08257
C	6.35400	2.47620	3.72158
C	4.91963	2.41972	4.21403
C	4.69169	2.47610	5.59586
C	3.41701	2.36102	6.12320
C	2.31665	2.18887	5.27420
O	1.11173	2.05257	5.86988
C	-0.03257	1.89021	5.06160
C	2.52067	2.16119	3.89504
C	3.81363	2.26848	3.38035
C	7.12011	1.14022	3.79734
C	6.47118	-0.08982	3.17589
H	5.98069	0.14805	2.22458
O	5.45272	-0.53738	4.09288
C	4.24177	-0.85167	3.63912
C	3.29318	-1.17495	4.74021
C	1.96474	-1.44984	4.40719
C	1.03453	-1.70944	5.40709
C	1.43085	-1.69723	6.74434
C	2.75830	-1.43367	7.07887
C	3.68992	-1.17105	6.07957
O	3.93997	-0.85850	2.46726
C	7.47925	-1.21098	2.96885
C	6.87519	-2.51681	2.46361
C	7.89888	-3.62899	2.24481
C	8.89729	-3.36948	1.11990
C	9.84774	-4.53764	0.88997
H	6.89403	3.17162	4.38199
H	5.53967	2.60389	6.27380
H	3.24612	2.39001	7.20069
H	-0.88623	1.78714	5.74228
H	0.03994	0.98557	4.43728
H	-0.19302	2.76451	4.40945
H	1.68730	2.03041	3.20634
H	3.93872	2.21411	2.29933
H	7.30665	0.91554	4.85774
H	8.10744	1.28376	3.33556
H	1.67343	-1.44332	3.35609
H	-0.00491	-1.91714	5.14514
H	0.70002	-1.89640	7.53133
H	3.06787	-1.42674	8.12595
H	4.72658	-0.95118	6.33231
H	8.23259	-0.83700	2.25879
H	8.00762	-1.39172	3.92108
H	6.32659	-2.32911	1.52517
H	6.12676	-2.86747	3.19161
H	7.36002	-4.56685	2.02740
H	8.44931	-3.80821	3.18585
H	9.48374	-2.46219	1.33966
H	8.34151	-3.15346	0.19072
H	10.55460	-4.33157	0.07216
H	9.29471	-5.45519	0.63254

H	10.43736	-4.75184	1.79574

VIII$_{ester}$

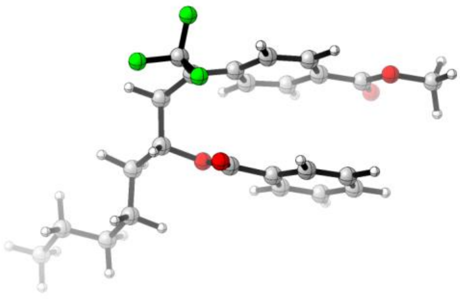

Charge: 0 , Multiplicity: 2

# of Imaginary Frequencies: 0

G_{sol}: -960247.205 kcal mol^{-1}

F	5.40715	4.17086	2.27437
C	5.49687	2.86759	1.95349
F	4.35925	2.56679	1.30188
F	6.48343	2.75611	1.05598
C	5.73539	2.00883	3.16121
C	4.80339	2.04595	4.24346
C	5.05862	1.32810	5.44265
C	4.09793	1.20953	6.42596
C	2.83743	1.79366	6.26282
C	1.79477	1.51496	7.28184
O	0.60327	2.00850	6.95103
C	2.58743	2.57315	5.12511
C	3.55074	2.71533	4.14549
C	6.86058	1.02468	3.09845
C	6.44991	-0.35372	2.54380
H	6.10213	-0.24895	1.50736
O	5.36057	-0.86620	3.32900
C	4.10331	-0.69300	2.91107
C	3.10423	-1.08502	3.94288
C	1.79080	-0.63630	3.78709
C	0.83923	-0.90999	4.76415
C	1.19386	-1.65047	5.89033
C	2.50009	-2.11654	6.03940
C	3.45735	-1.82742	5.07361
O	3.81544	-0.23371	1.83085
C	7.58189	-1.35923	2.62989
C	7.24312	-2.70440	1.99615
C	8.27482	-3.79695	2.26605
C	9.66418	-3.52509	1.69645
C	10.63584	-4.67453	1.93358
H	6.01437	0.82854	5.58588
H	4.29786	0.62440	7.32384
H	1.61466	3.04845	5.00164
H	3.30936	3.30582	3.26400
H	7.29447	0.87479	4.09561
H	7.66345	1.40520	2.45508
H	1.53382	-0.05061	2.90349
H	-0.18110	-0.53838	4.65116
H	0.44872	-1.86194	6.66040
H	2.77667	-2.69654	6.92207
H	4.48635	-2.16518	5.19632
H	8.46320	-0.91178	2.14598
H	7.84145	-1.49680	3.69362
H	7.11912	-2.57016	0.90743
H	6.26591	-3.03840	2.37918
H	7.90077	-4.74799	1.85018
H	8.35655	-3.95414	3.35661
H	10.07582	-2.60336	2.14012
H	9.57741	-3.32872	0.61347
H	11.63077	-4.45852	1.51582
H	10.27151	-5.60467	1.46879
H	10.76082	-4.87154	3.01045
O	1.98827	0.88480	8.29664
C	-0.46931	1.75182	7.85183
H	-1.35420	2.23113	7.41990
H	-0.25225	2.17452	8.84256
H	-0.63404	0.66991	7.95320

IX$_{ester}$

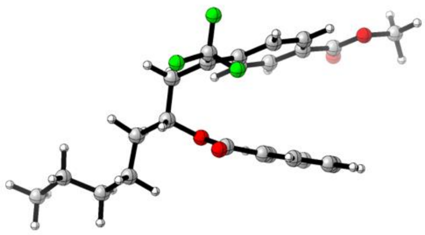

Charge: -1, Multiplicity: 1

# of Imaginary Frequencies: 0

G_{sol}: 960334.494 kcal mol^{-1}

F	5.07329	3.88206	2.13259
C	5.03277	2.51765	1.98845
F	3.76243	2.30225	1.53364

F	5.83319	2.25937	0.93082
C	5.41534	1.75454	3.18122
C	4.61633	1.81007	4.32998
C	4.92317	1.05931	5.52219
C	4.11578	1.07480	6.63101
C	2.92539	1.83150	6.68344
C	2.08618	1.77047	7.86545
O	0.97200	2.53133	7.77356
C	2.61563	2.60538	5.54202
C	3.41773	2.61117	4.42762
C	6.64868	0.89622	3.08241
C	6.43621	-0.49663	2.46498
H	6.23190	-0.39512	1.39120
O	5.27039	-1.08616	3.07089
C	4.10127	-0.97409	2.44483
C	2.94085	-1.25248	3.33750
C	1.65995	-0.97778	2.85092
C	0.54738	-1.17532	3.66088
C	0.71118	-1.65834	4.95937
C	1.98707	-1.94230	5.44439
C	3.10243	-1.73369	4.63912
O	3.98301	-0.68901	1.27372
C	7.60019	-1.44098	2.68973
C	7.45980	-2.76591	1.94764
C	8.54344	-3.79196	2.27021
C	9.95660	-3.37959	1.86785
C	10.98949	-4.46917	2.12857
H	5.80925	0.42622	5.53969
H	4.38456	0.47112	7.50117
H	1.71096	3.21667	5.54841
H	3.12167	3.23347	3.58449
H	7.09259	0.75891	4.07898
H	7.42581	1.38572	2.47481
H	1.55614	-0.59523	1.83458
H	-0.45136	-0.94932	3.28152
H	-0.16161	-1.81309	5.59781
H	2.11429	-2.31653	6.46233
H	4.10434	-1.93428	5.01558
H	8.51679	-0.91655	2.37758
H	7.69787	-1.62325	3.77434
H	7.45042	-2.56983	0.86094
H	6.47567	-3.19849	2.18936
H	8.29543	-4.74117	1.76490
H	8.52567	-4.01177	3.35287
H	10.24744	-2.46451	2.40991
H	9.96124	-3.11211	0.79656
H	12.00056	-4.15052	1.83307
H	10.74978	-5.38627	1.56682
H	11.02204	-4.73711	3.19686
O	2.31109	1.11228	8.86912
C	0.09547	2.50884	8.88564
H	-0.74501	3.16601	8.63283
H	0.59573	2.87577	9.79412
H	-0.27239	1.49058	9.08022

X_{ester}

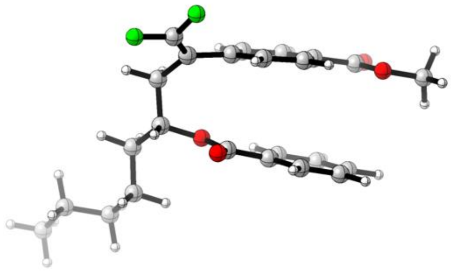

Charge: 0 , Multiplicity: 1

# of Imaginary Frequencies: 0

G_{sol}: --897581.085 kcal mol^{-1}

C	5.93008	2.15745	3.24428
C	4.87456	2.11090	4.28758
C	5.19266	1.70788	5.59240
C	4.20245	1.55985	6.55015
C	2.86579	1.80550	6.22871
C	1.83132	1.52590	7.25777
O	0.59077	1.72136	6.81424
C	2.54115	2.24072	4.94342
C	3.53544	2.39242	3.98573
C	6.98911	1.08240	3.19526
C	6.49511	-0.25656	2.64664
H	6.12284	-0.12185	1.62261
O	5.39317	-0.71293	3.45473
C	4.14757	-0.64761	2.98281
C	3.13257	-1.04953	3.99535
C	1.78198	-0.91790	3.65953
C	0.79828	-1.22024	4.59267
C	1.16058	-1.66044	5.86597
C	2.50553	-1.80463	6.20024
C	3.49265	-1.49827	5.26791
O	3.87126	-0.27925	1.86404
C	7.56906	-1.32779	2.68787
C	7.11676	-2.66551	2.11260
C	8.12717	-3.79504	2.29761

C	9.45180	-3.59750	1.56650
C	10.40035	-4.77796	1.73220
H	6.22597	1.48328	5.85828
H	4.45125	1.22148	7.55666
H	1.49992	2.42795	4.68252
H	3.25967	2.69002	2.97415
H	7.39983	0.91818	4.20237
H	7.82576	1.40804	2.56364
H	1.51981	-0.55889	2.66350
H	-0.25574	-1.10610	4.33169
H	0.38793	-1.88971	6.60338
H	2.78772	-2.14384	7.19880
H	4.54668	-1.59066	5.52706
H	8.44046	-0.94208	2.13710
H	7.89104	-1.45961	3.73544
H	6.88970	-2.54209	1.03920
H	6.17081	-2.95398	2.59731
H	7.67105	-4.73937	1.95485
H	8.32635	-3.92834	3.37614
H	9.94568	-2.68093	1.92921
H	9.24913	-3.43009	0.49421
H	11.34857	-4.61525	1.19796
H	9.95086	-5.70608	1.34423
H	10.63970	-4.94686	2.79438
O	2.07415	1.14037	8.37888
C	-0.46689	1.43031	7.72136
H	-1.39804	1.64684	7.18702
H	-0.38929	2.05643	8.62091
H	-0.43682	0.37264	8.01834
C	5.94064	3.11922	2.32466
F	5.09337	4.12315	2.25153
F	6.82618	3.21755	1.35511

XI$_{ester}$

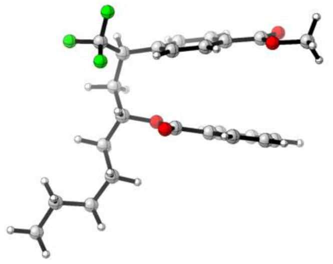

Charge: 0 , Multiplicity: 1

# of Imaginary Frequencies: 0

G_{sol}: -960606.140 kcal mol^{-1}

F	5.69216	4.31058	1.97168
C	6.29224	3.13127	2.16368
F	5.80893	2.29433	1.22607
F	7.59163	3.30101	1.88244
C	6.11163	2.62991	3.58364
C	4.67582	2.67901	4.07585
C	4.46162	2.90866	5.44044
C	3.17988	2.91214	5.97157
C	2.07673	2.69201	5.14291
C	0.72205	2.65756	5.76007
O	-0.23905	2.37111	4.88429
C	2.27816	2.48777	3.77707
C	3.56546	2.48065	3.24952
C	6.83104	1.28375	3.81161
C	6.21334	0.01851	3.22523
H	5.80635	0.19783	2.22379
O	5.11265	-0.36698	4.07741
C	3.94229	-0.70229	3.53408
C	2.90804	-1.03185	4.55655
C	1.60618	-1.28548	4.11438
C	0.59957	-1.56735	5.03122
C	0.89189	-1.60437	6.39484
C	2.19128	-1.36099	6.83897
C	3.19947	-1.07321	5.92354
O	3.73336	-0.72097	2.34229
C	7.22431	-1.11953	3.16612
C	6.65660	-2.44169	2.65737
C	7.70079	-3.54533	2.48756
C	8.71804	-3.29919	1.37505
C	9.68347	-4.46233	1.18155
H	6.67237	3.36893	4.17582
H	5.31773	3.08207	6.09700
H	3.01847	3.08094	7.03729
H	1.42236	2.31922	3.12350
H	3.69327	2.30011	2.18280
H	6.93871	1.13744	4.89662
H	7.85072	1.38180	3.41249
H	1.39650	-1.24977	3.04434
H	-0.41732	-1.76020	4.68289
H	0.10182	-1.82635	7.11594
H	2.41952	-1.39374	7.90639
H	4.21584	-0.87573	6.26448
H	8.04224	-0.78041	2.51201
H	7.66345	-1.26233	4.16888
H	6.14329	-2.27280	1.69546
H	5.88332	-2.79175	3.35992

H	7.17787	-4.49459	2.28061
H	8.23399	-3.69709	3.44329
H	9.29341	-2.38274	1.58660
H	8.17700	-3.10583	0.43219
H	10.40265	-4.26180	0.37300
H	9.14421	-5.38927	0.92840
H	10.25958	-4.65674	2.10042
O	0.50631	2.85402	6.93396
C	-1.56856	2.29121	5.38983
H	-2.20851	2.05104	4.53399
H	-1.87215	3.24913	5.83464
H	-1.64316	1.50315	6.15221

TS4$_{ester}$

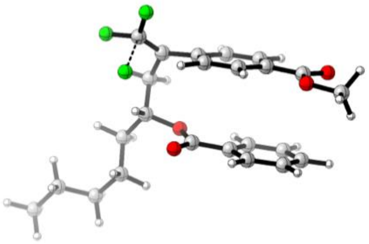

Charge: -1, Multiplicity: 1

# of Imaginary Frequencies: 1 (-118.18 cm^{-1})

G_{sol}: -960327.369kcal mol^{-1}

F	5.85809	3.98727	2.54966
C	6.54069	2.88118	2.77276
F	5.51071	1.91460	1.22482
F	7.65558	2.89559	2.06522
C	6.37916	2.10831	3.85872
C	5.08019	2.13450	4.56112
C	4.98696	2.01018	5.95506
C	3.75009	1.94161	6.57918
C	2.57295	1.99142	5.82647
C	1.27930	1.82762	6.52874
O	0.23475	1.78687	5.69749
C	2.65562	2.12847	4.43617
C	3.89173	2.19658	3.81130
C	7.26582	0.89385	3.98574
C	6.65988	-0.31294	3.26269
H	6.18182	0.05049	2.34112
O	5.61969	-0.82307	4.13398
C	4.39673	-1.05093	3.67000
C	3.41480	-1.31014	4.76293
C	2.06015	-1.37300	4.42808
C	1.10186	-1.54512	5.41993
C	1.49617	-1.67247	6.75152
C	2.84806	-1.62606	7.08766
C	3.80691	-1.43828	6.09726
O	4.09482	-1.03396	2.49774
C	7.66228	-1.41582	2.97848
C	7.05665	-2.66419	2.34480
C	8.08586	-3.71020	1.92159
C	8.93542	-3.31158	0.71745
C	9.91812	-4.39592	0.29452
H	5.89931	1.95005	6.55287
H	3.67744	1.82810	7.66181
H	1.73980	2.14152	3.84460
H	4.00527	2.22076	2.72328
H	7.44194	0.62253	5.03906
H	8.24890	1.10019	3.54061
H	1.77175	-1.26377	3.38188
H	0.04263	-1.57593	5.15654
H	0.74409	-1.80400	7.53267
H	3.15611	-1.72400	8.13068
H	4.86422	-1.37916	6.35334
H	8.42820	-0.98417	2.31525
H	8.17851	-1.68753	3.91608
H	6.45015	-2.37119	1.47141
H	6.35519	-3.11882	3.06237
H	7.56204	-4.65169	1.68406
H	8.74798	-3.93938	2.77577
H	9.48965	-2.38449	0.93913
H	8.26502	-3.06902	-0.12542
H	10.51198	-4.08798	-0.57930
H	9.39231	-5.32744	0.03032
H	10.62098	-4.63397	1.10894
O	1.15578	1.72226	7.72969
C	-1.04227	1.57925	6.28658
H	-1.76689	1.59620	5.46518
H	-1.27162	2.37246	7.01161
H	-1.07586	0.60749	6.79947

x

5. Condition-based sensitivity screening

A condition-based sensitivity screening approach was undertaken according to the report of Glorius and co-workers,[47] as detailed below.

Preparation of stock solution 1. $ZnBr_2$ (0.075 mmol for each reaction, 5 mol%) was charged under air, then the vessel was evacuated and back-filled three times with argon. Anhydrous CH_2Cl_2 (0.2 ml) was added, then the solution was cooled to -15 °C and benzoyl bromide (0.15 mmol for each reaction, 1.50 equiv.) was added, followed by the dropwise addition of aldehyde **1a** (0.15 mmol for each reaction, 1.50 equiv.). The reaction was stirred at 0 °C for two hours.

Preparation of stock solution 2. In an oven-dried Schlenk tube equipped with a PTFE-coated stirring bar, 4CzIPN (5 µmol for each reaction, 5 mol%), alkene **2** (0.1 mmol for each reaction, 1.00 equiv.) were charged under air, then the vessel was evacuated and back-filled three times with argon. Dry MeCN (1.5 ml for each reaction) was added and the solution was stirred for 5 minutes.

Reaction set-up. In the individual oven-dried Schlenk tube equipped with a PTFE-coated stirring bar, KOAc (0.4 mmol, 4.0 equiv.) and $(TES)_3SiOH$ (0.15 mmol, 1.50 equiv.) were charged under air, then the vessel was evacuated and back-filled three times with argon. The appropriate amount of dry MeCN (*indicated in table*) was added, then additives (*indicated in table*). Stock solution 1 (1.5 ml) and stock solution 2 (100 µl) were transferred via syringe, then the reaction was degassed as detailed in the individual entries, then sealed and irradiated with 30W 450 nm LEDs using the standard set-up or the variation detailed in [47]. After 36 hours, the irradiation was interrupted, then the reaction was filtered through a short pad of silica, rinsing with EtOAc. The volatiles were removed *in vacuo*, then the crude residue was analyzed by ^1H NMR using dibromomethane as internal standard.

Supplementary Table 1. Reactions of the condition-based sensitivity screening

Entry	Reaction	Added MeCN	Other
1	Standard	500 µl	-

2	High C (+10%)	300 µl	-
3	Low C (-10%)	700 µl	-
4	High H_2O	500 µl	20 µl of H_2O added
5	Medium O_2	500 µl	Argon sparging
6	High O_2	500 µl	20 ml of air injected
7	High T	500 µl	Aluminum foil wrapping
8	High I (16 x)	500 µl	d =0.8 cm
9	Low I (1/16 x)	500 µl	d = 8.8 cm
10	Standard low T	500 µl	Water jacket, no flow
11	Low T	500 µl	Flowing water jacket

Supplementary Table 2. Results the condition-based sensitivity screening

Entry	Reaction	Yield %	Variation %
1	Standard	48	-
2	High C (+10%)	48	0
3	Low C (-10%)	46	-4
4	High H_2O	31	-35
5	Medium O_2	42	-13
6	High O_2	12	-75
7	High T	39	-19
8	High I (16 x)	38	-21
9	Low I (1/16 x)	48	0
10	Standard low T	41	-
11	Low T	32	-22

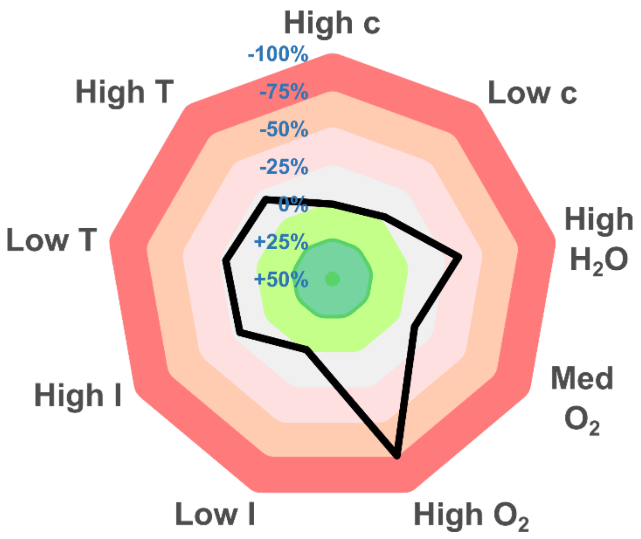

6. ^{1}H, ^{13}C, ^{19}F NMR and IR Spectra of Compounds

^{1}H NMR (400 MHz, $CDCl_3$)

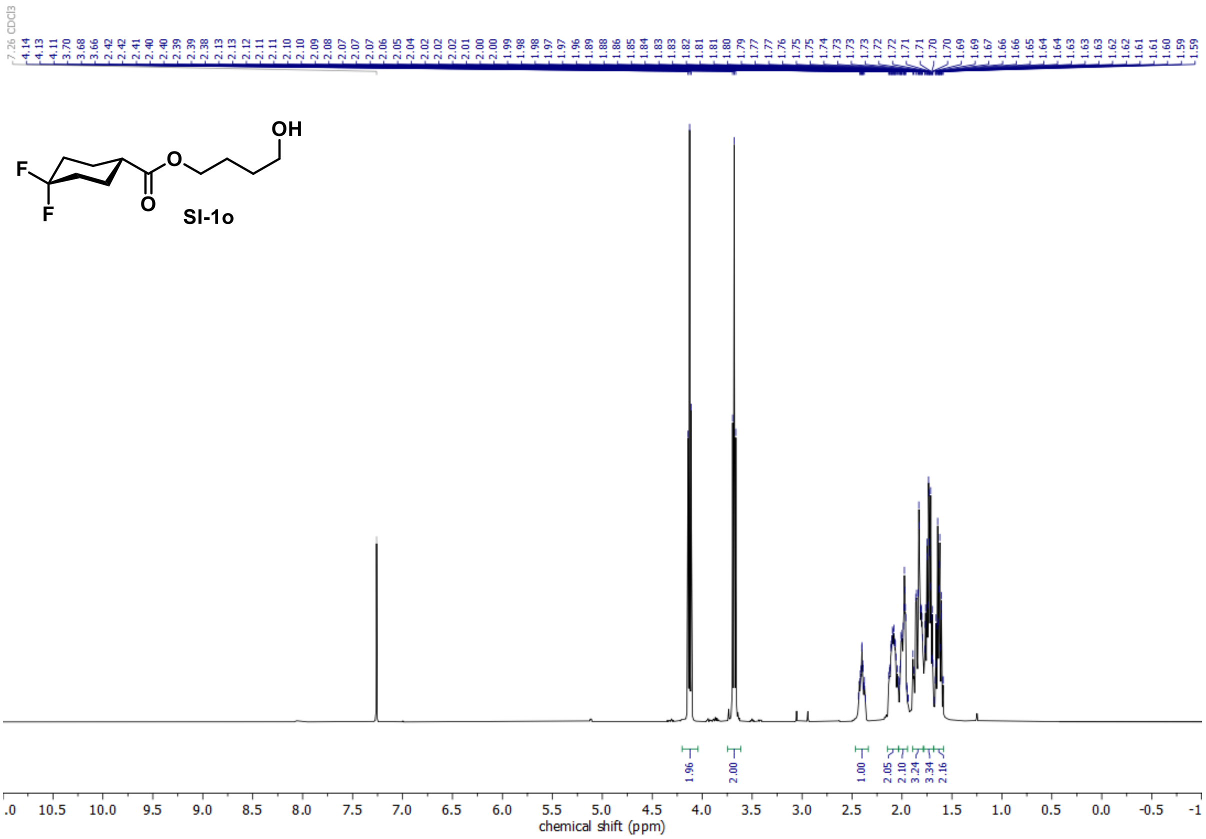

$^{13}C\{^{1}H\}$ NMR (101 MHz, $CDCl_3$)

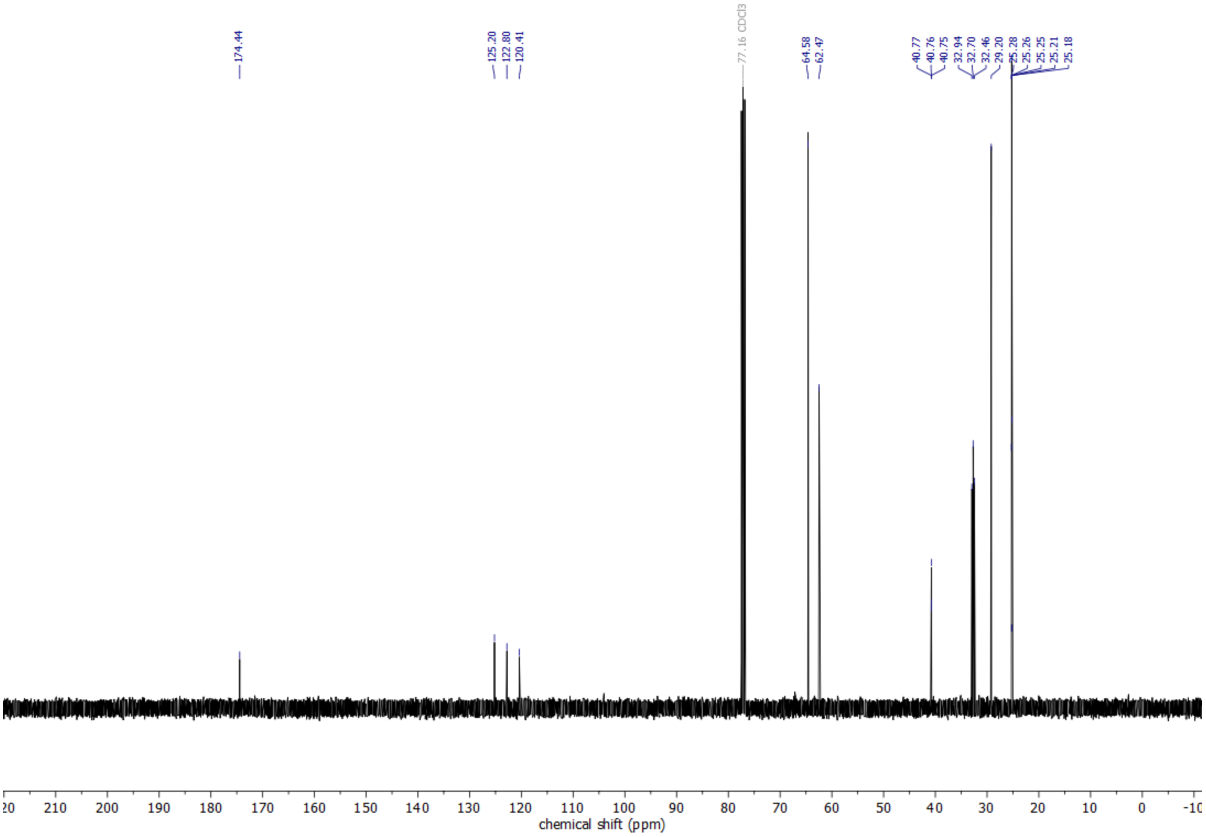

^{19}F NMR (377 MHz, $CDCl_3$)

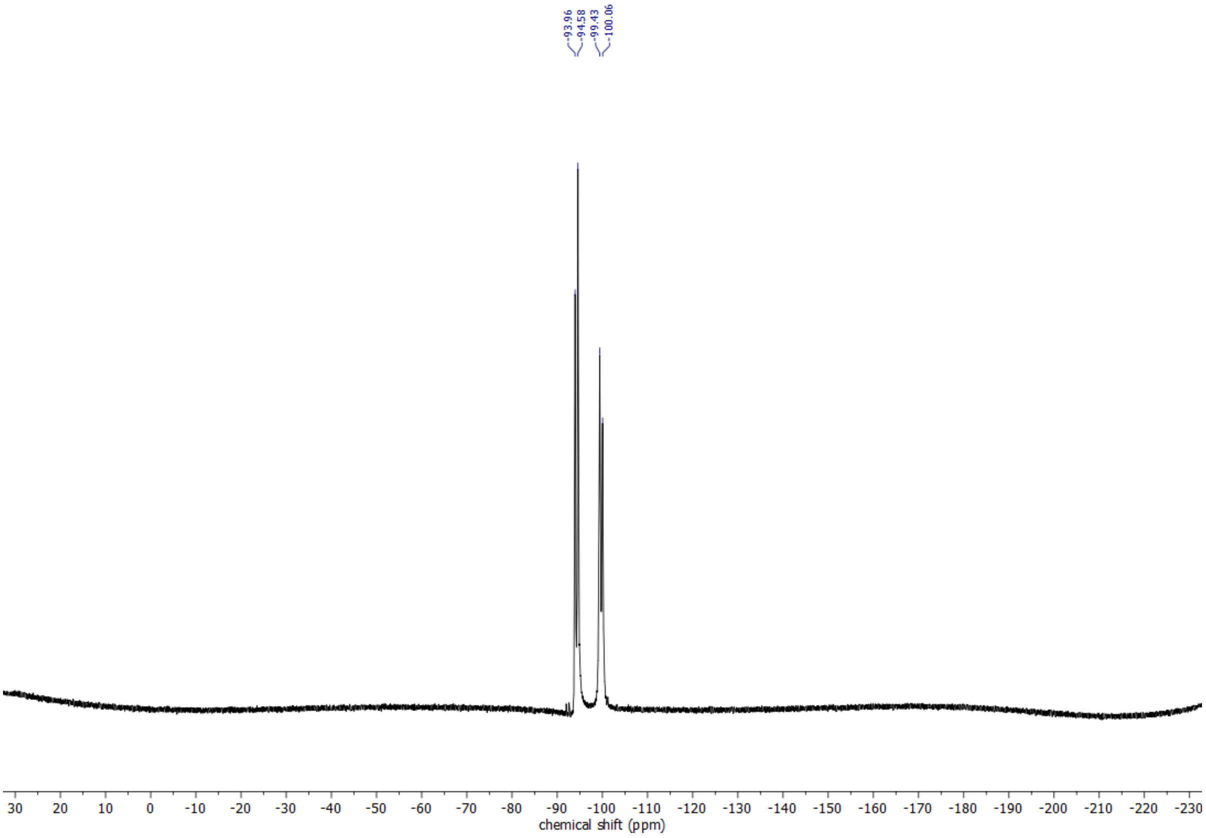

^{1}H NMR (599MHz, $CDCl_3$)

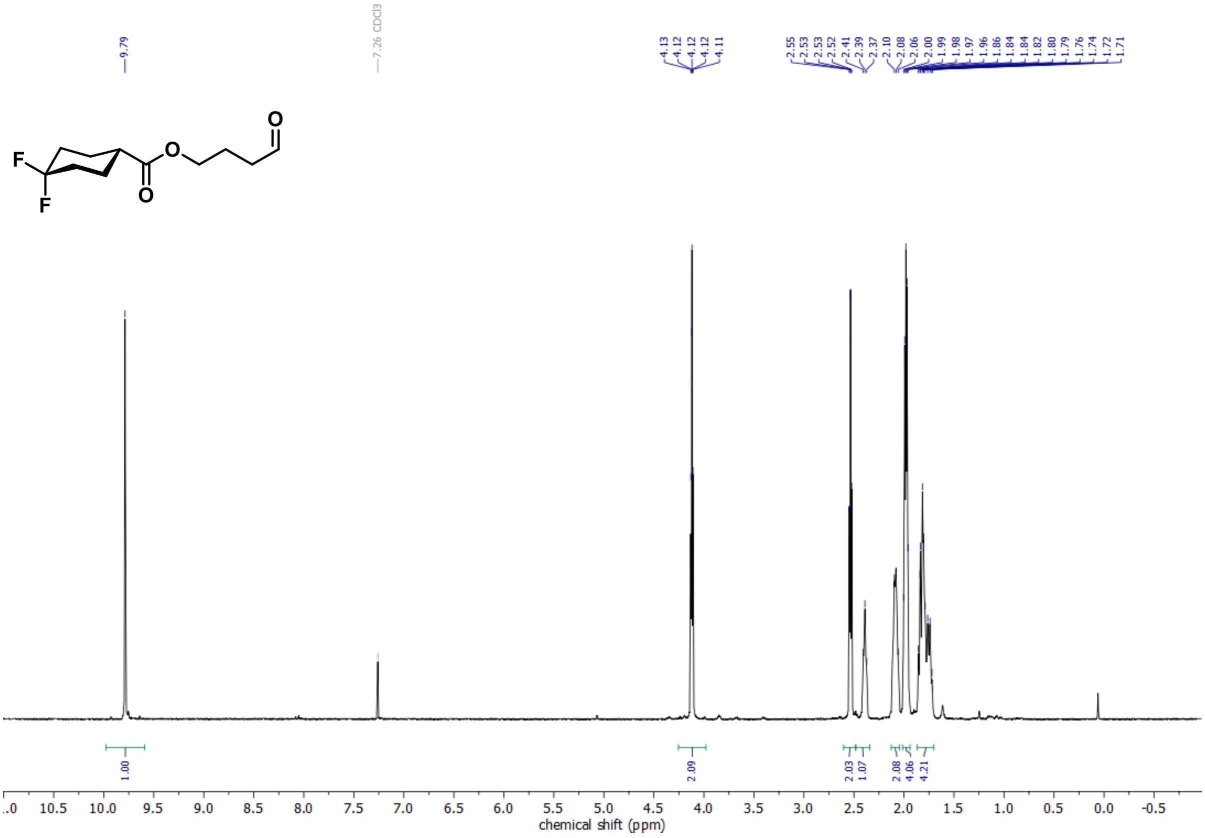

$^{13}C\{^{1}H\}$ NMR (151 MHz, $CDCl_3$)

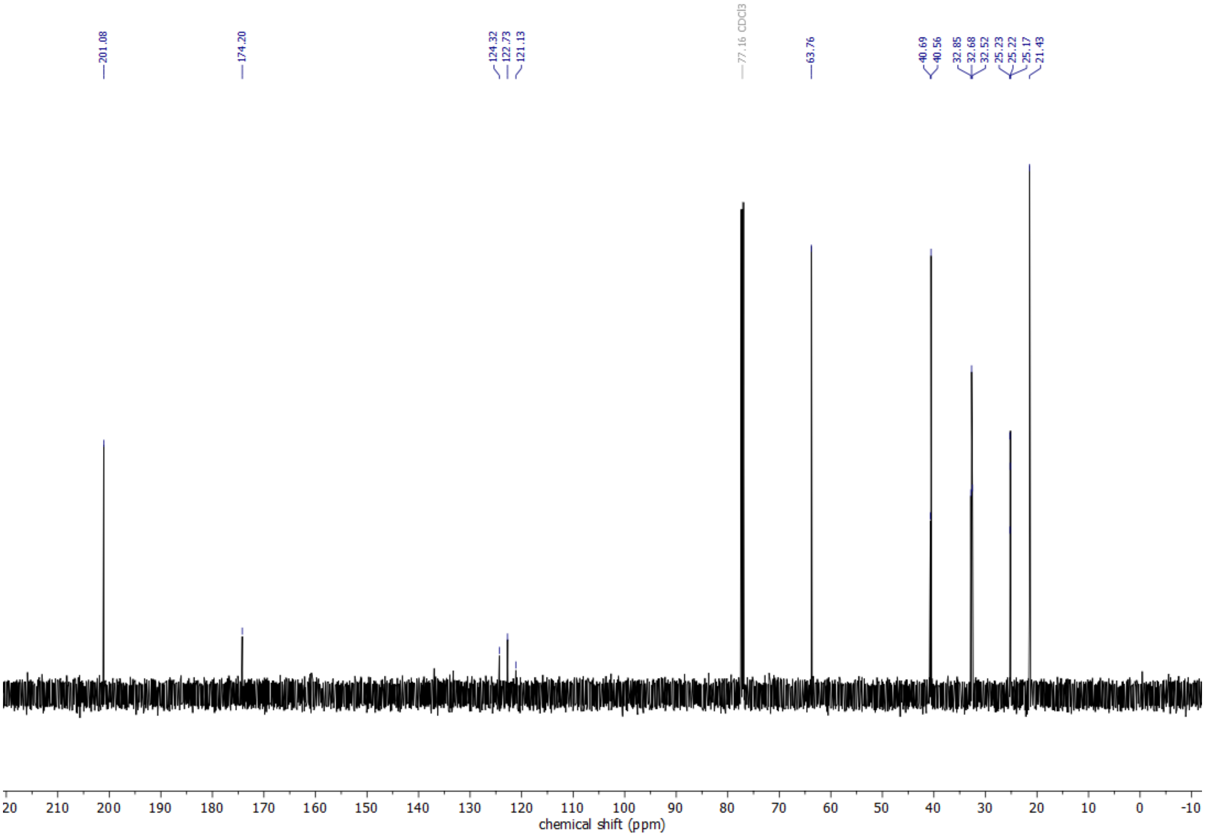

^{19}F NMR (564 MHz, $CDCl_3$)

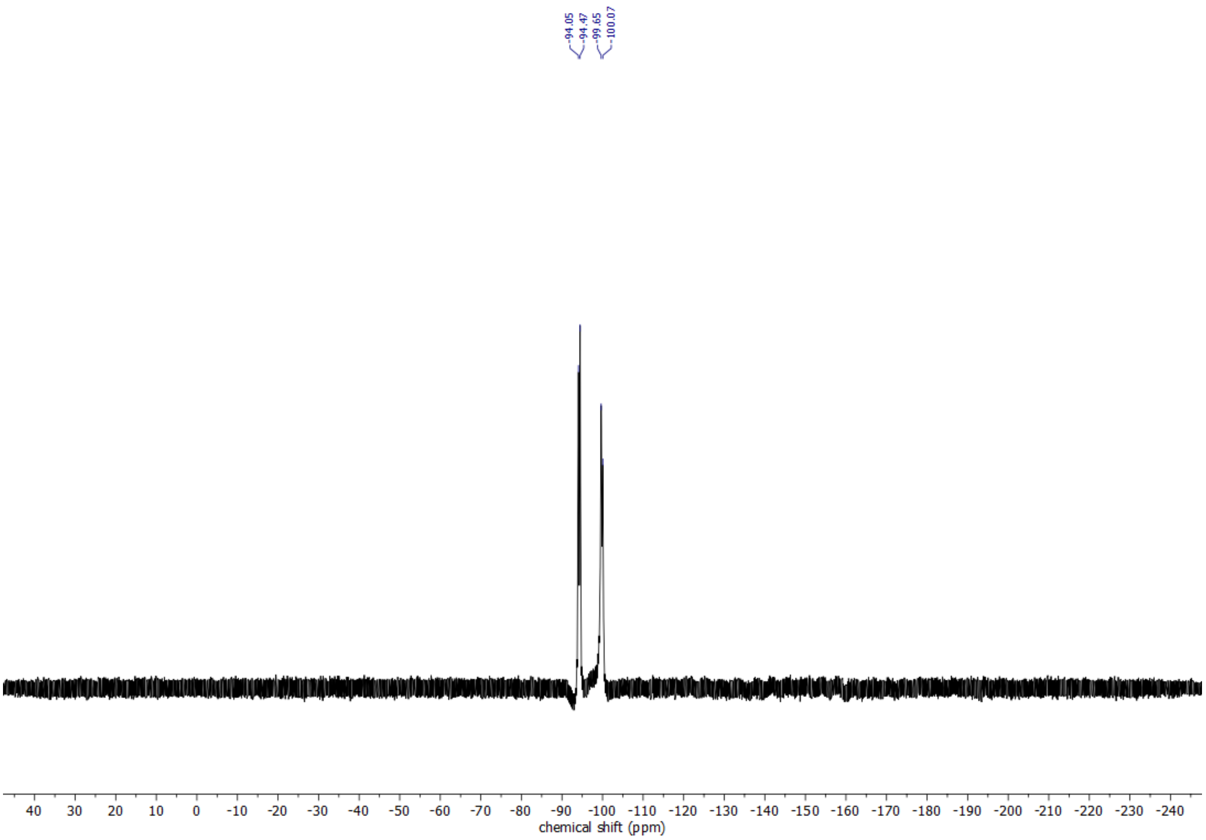

^1H NMR (400 MHz, $CDCl_3$)

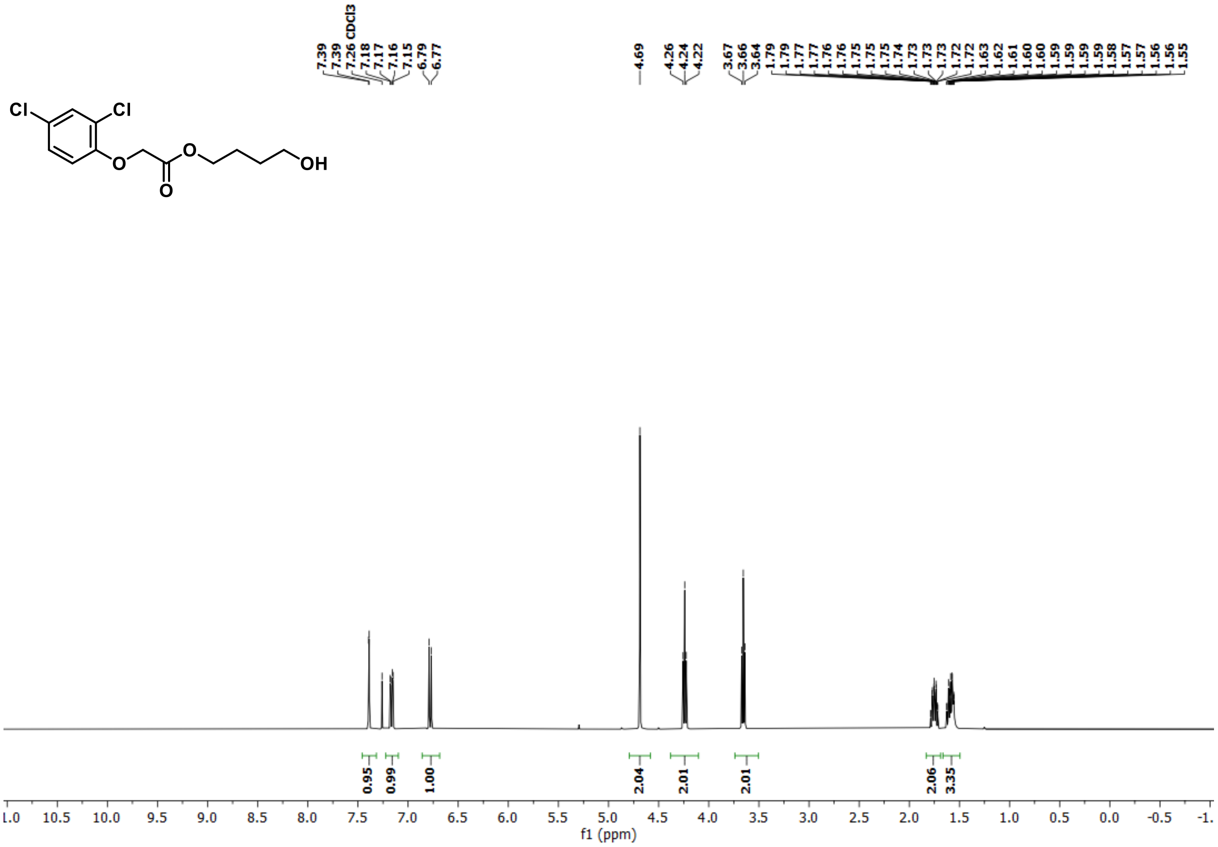

$^{13}C\{^1H\}$ NMR (101 MHz, $CDCl_3$)

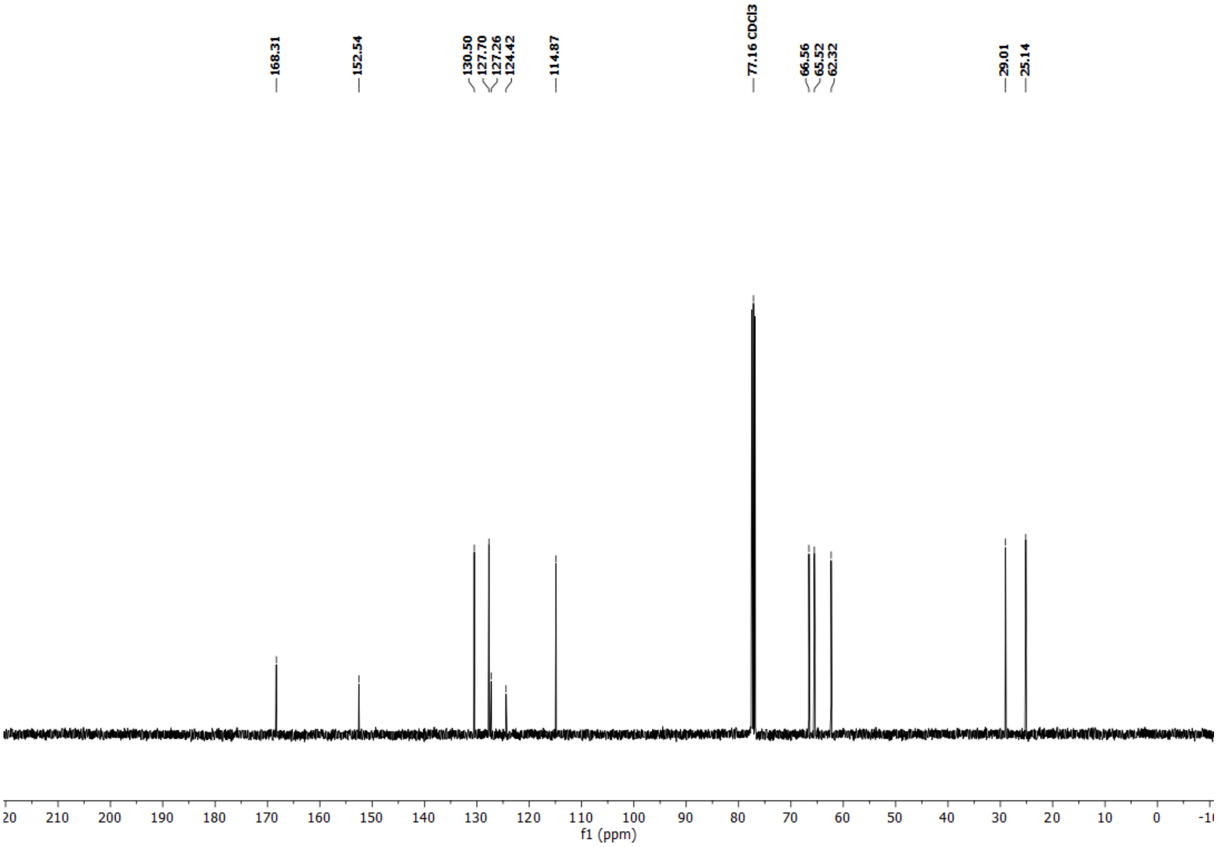

^{1}H NMR (599 MHz, $CDCl_3$)

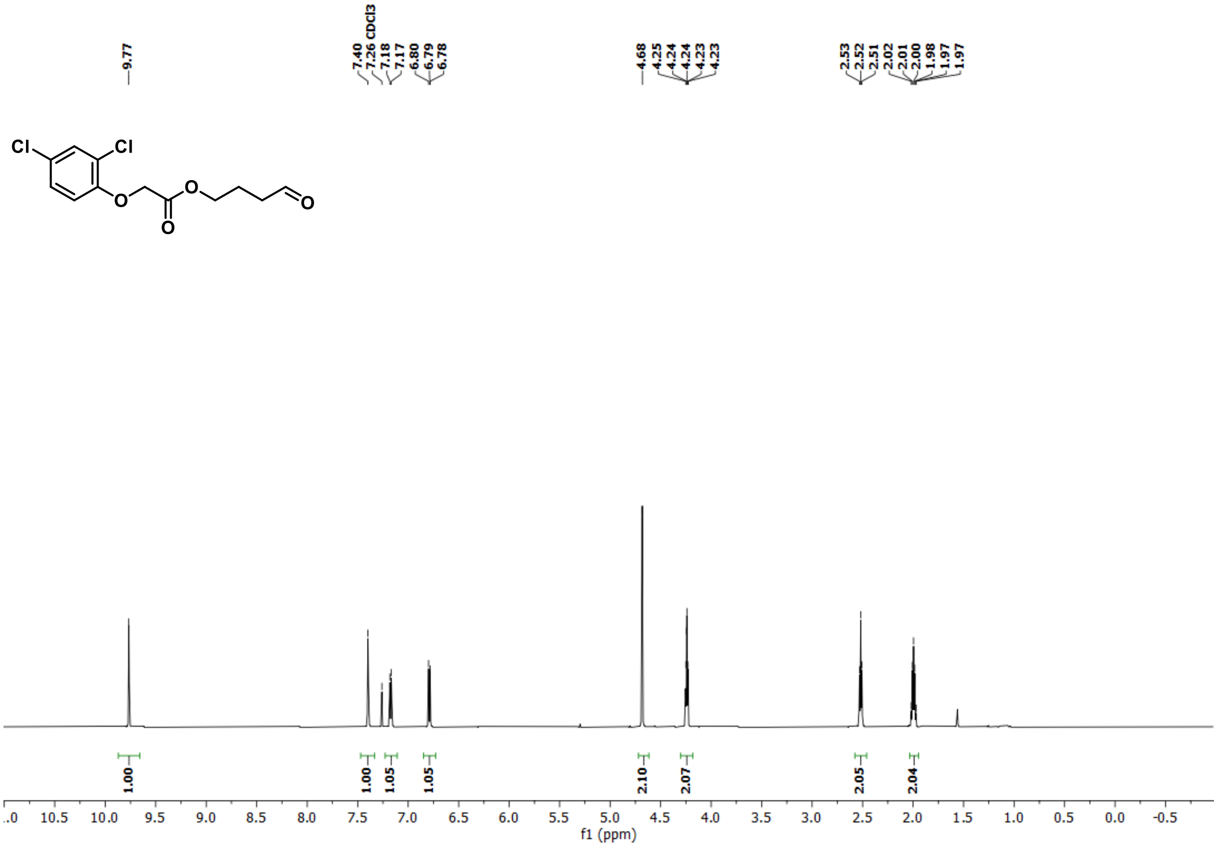

$^{13}C\{^{1}H\}$ NMR (151 MHz, $CDCl_3$)

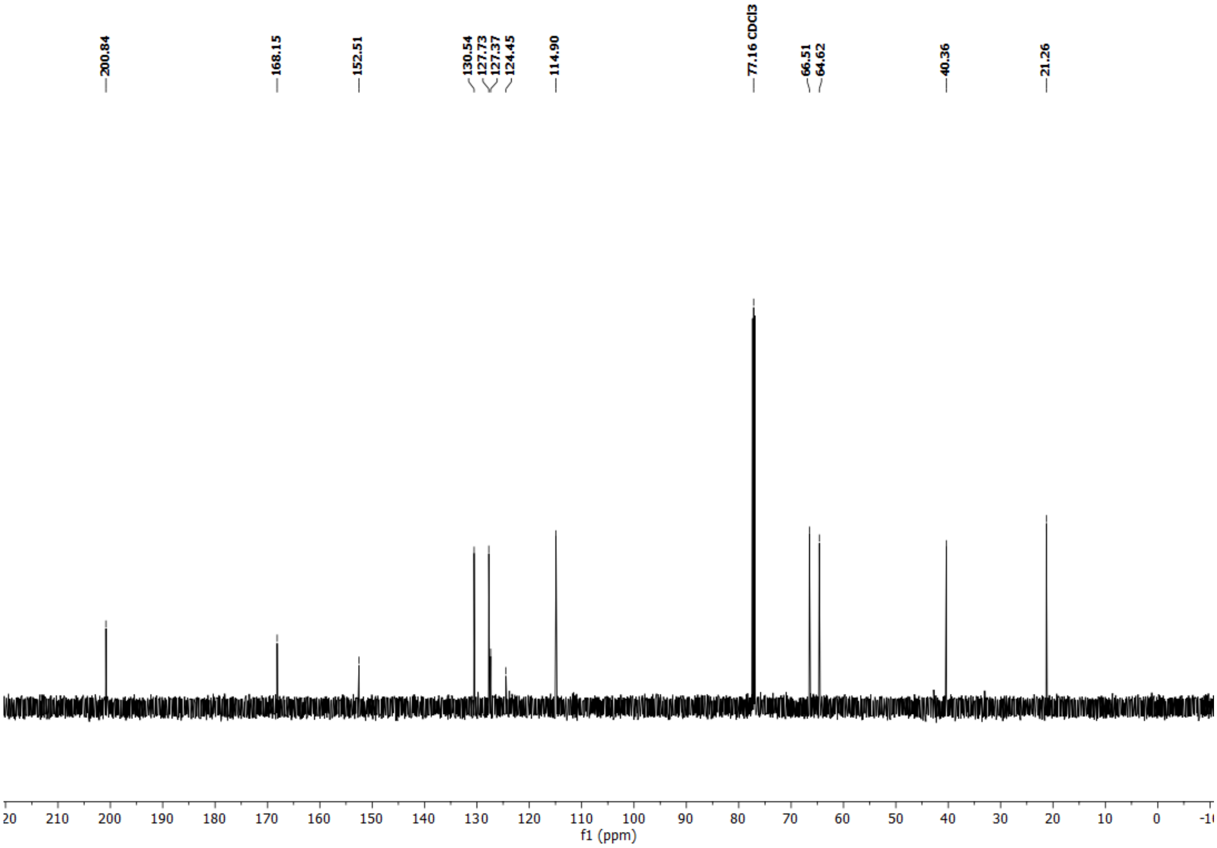

^{1}H NMR (400 MHz, $CDCl_3$)

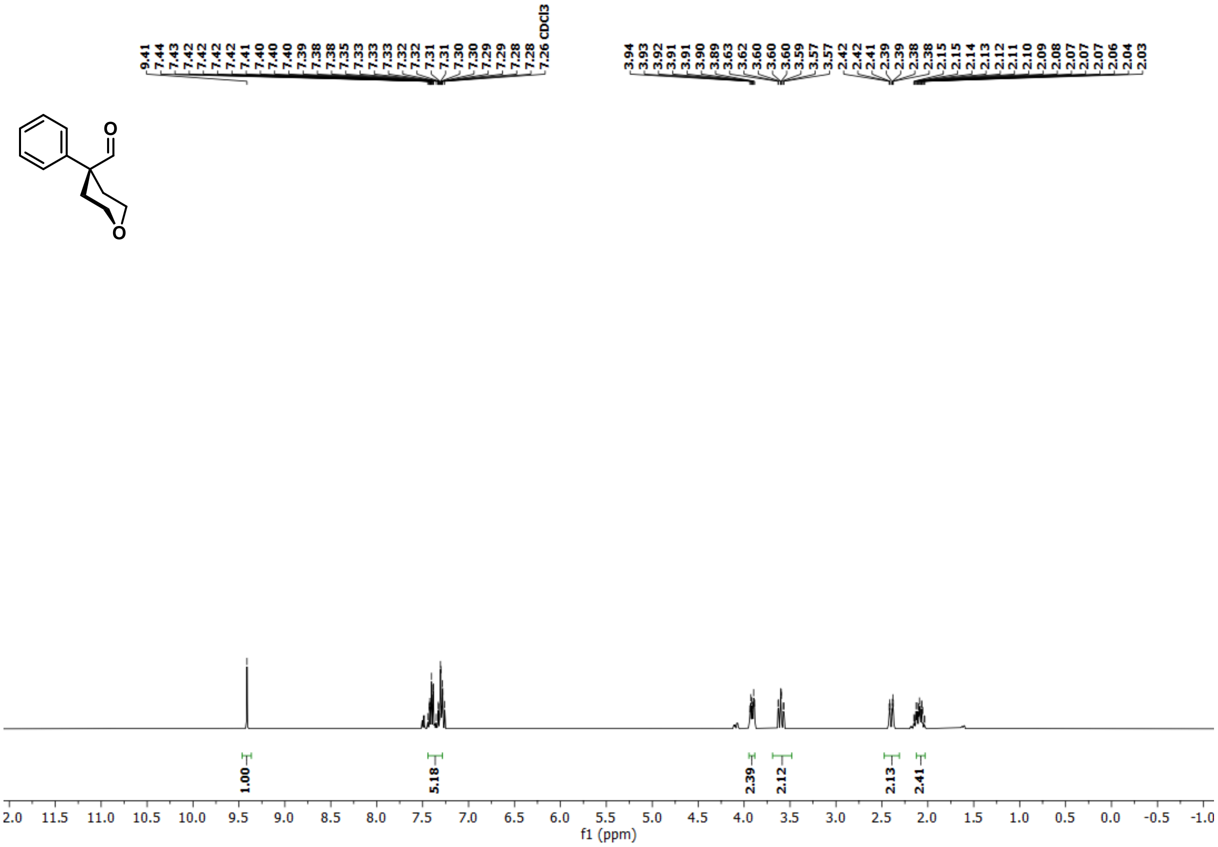

^{13}C{^{1}H} NMR (101 MHz, $CDCl_3$)

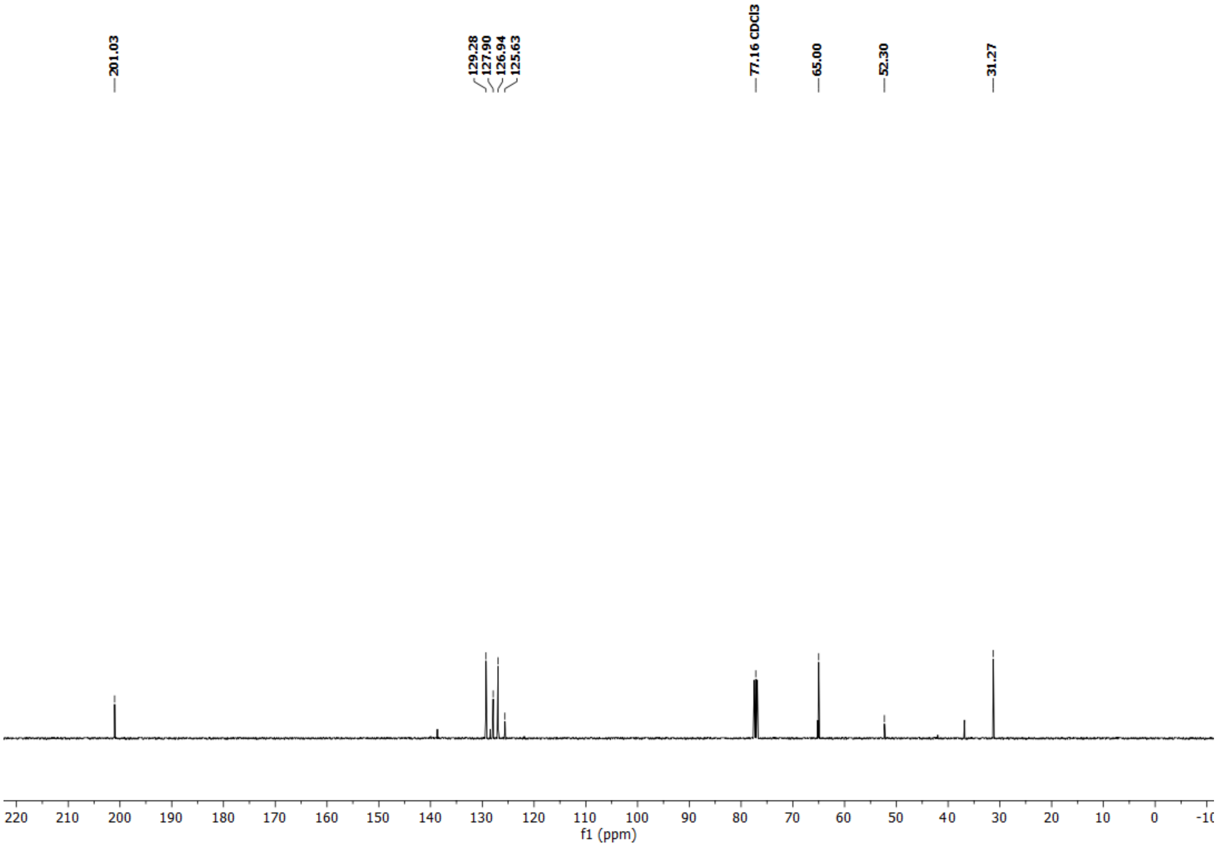

^{1}H NMR (400 MHz, $CDCl_3$)

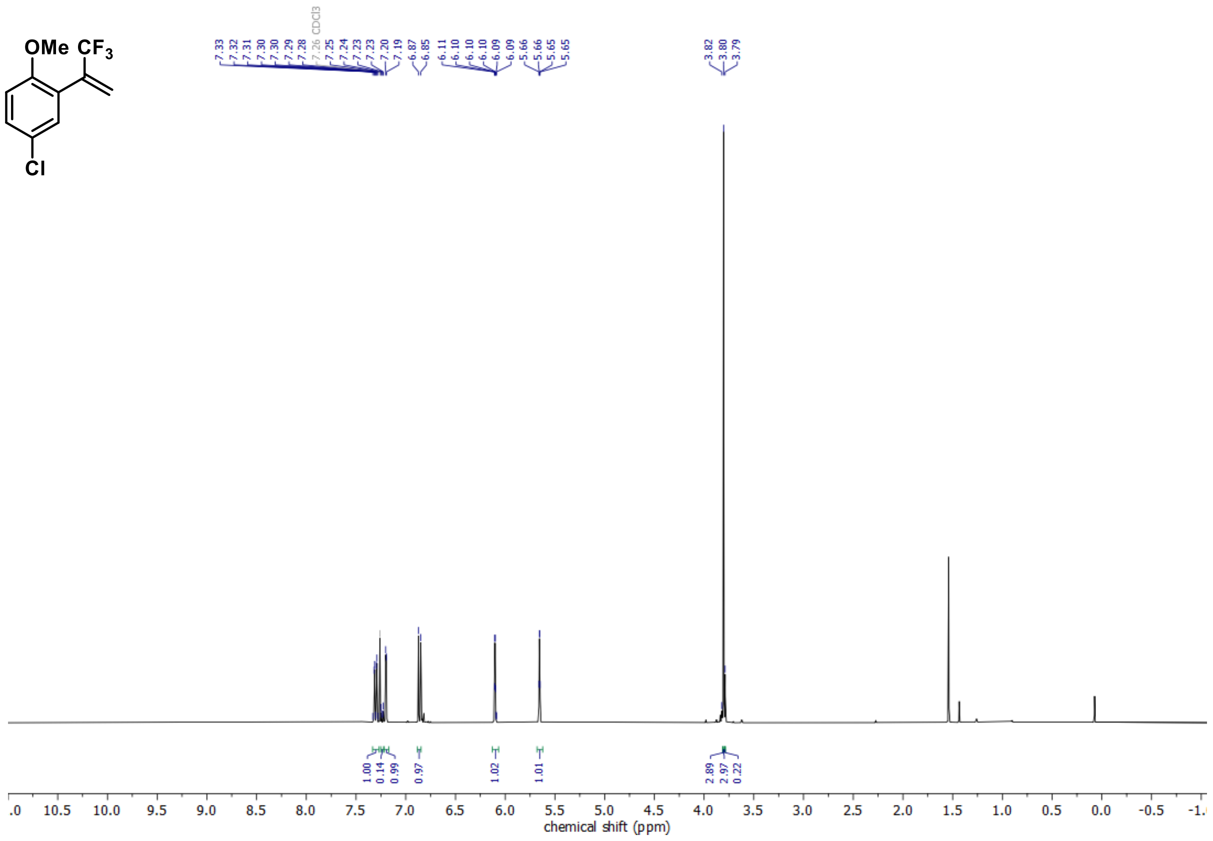

$^{13}C\{^{1}H\}$ NMR (101 MHz, $CDCl_3$)

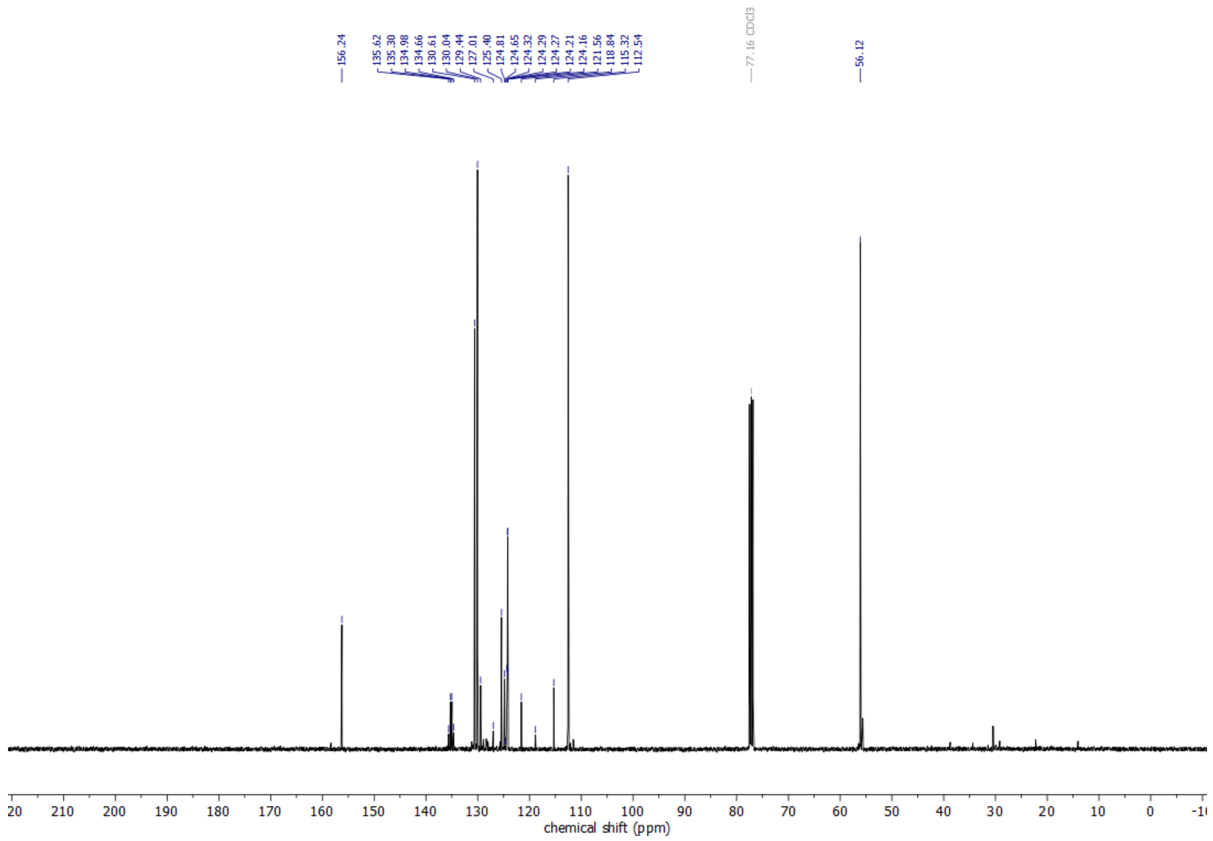

^{19}F NMR (377 MHz, $CDCl_3$)

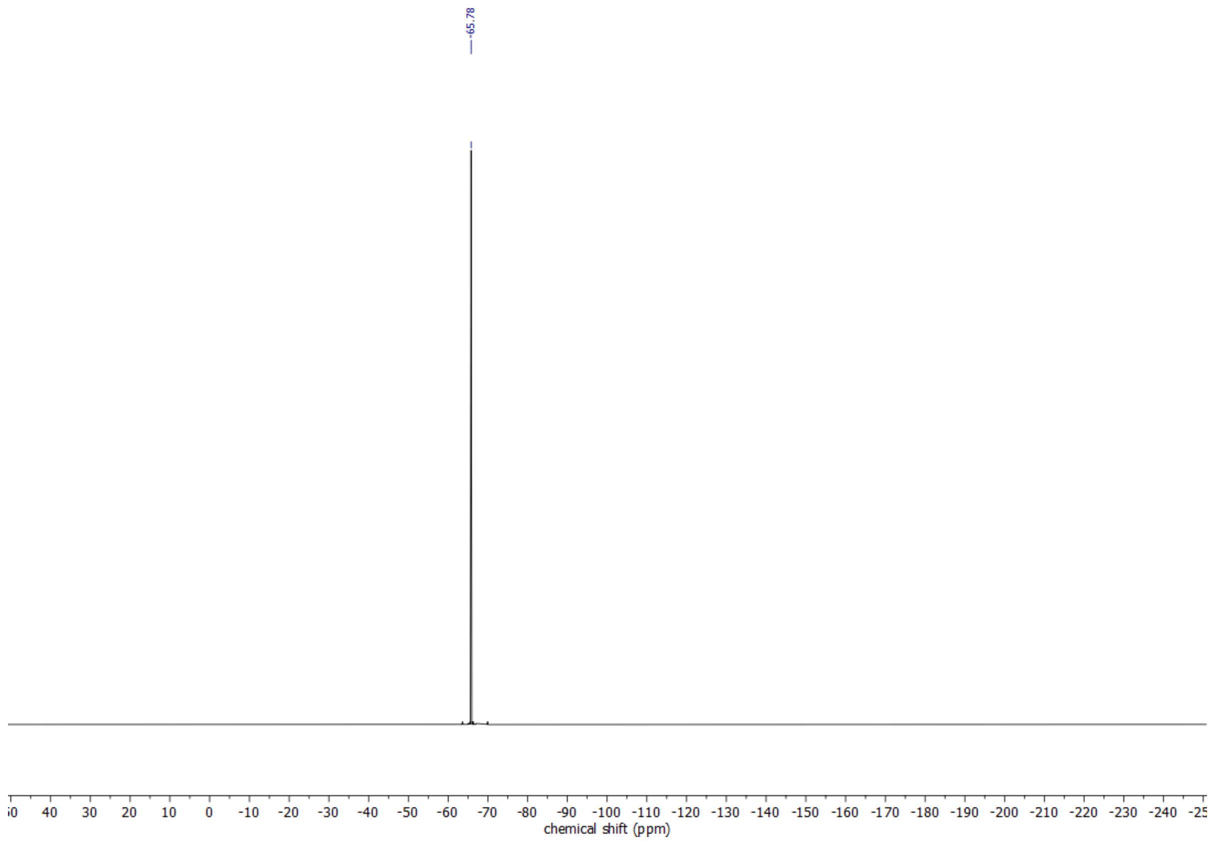

^{1}H NMR (400 MHz, $CDCl_3$)

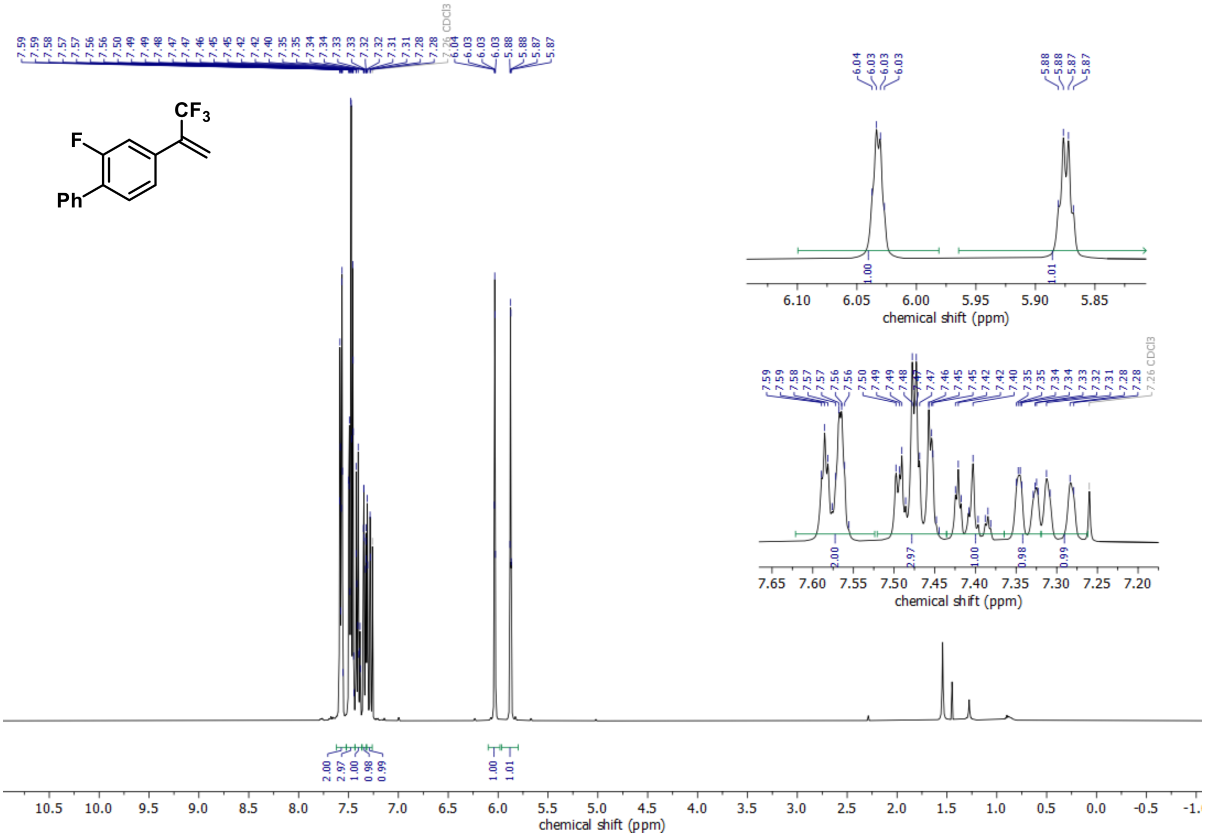

$^{13}C\{^{1}H\}$ NMR (101 MHz, $CDCl_3$)

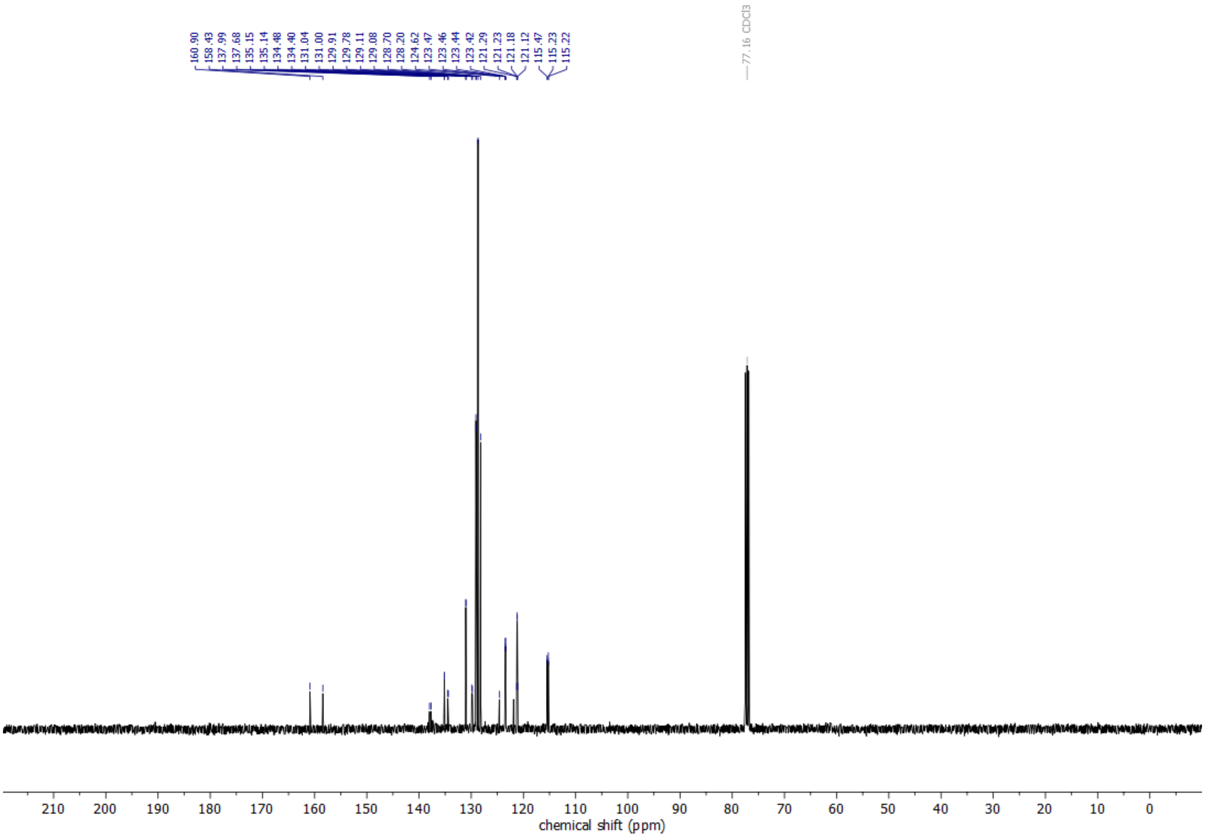

^{19}F NMR (377 MHz, $CDCl_3$)

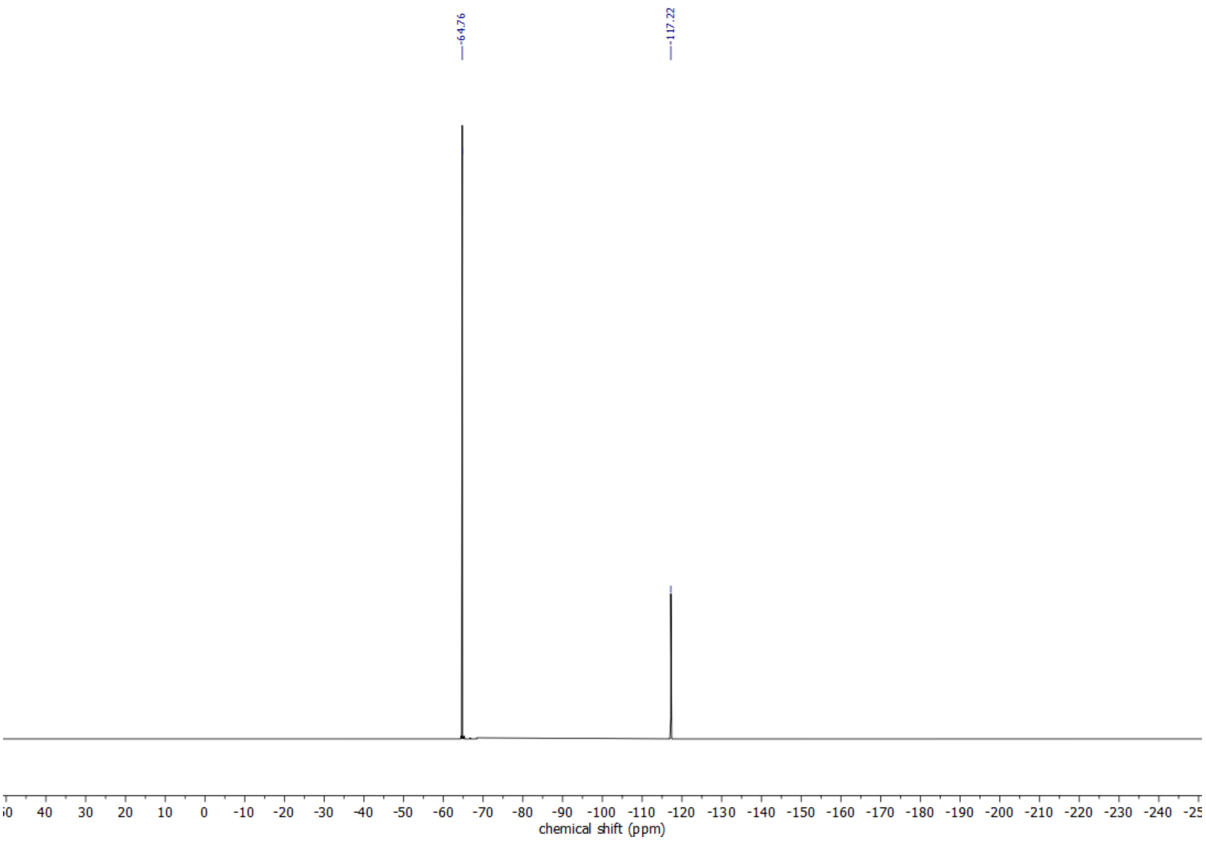

^{1}H NMR (400 MHz, $CDCl_3$)

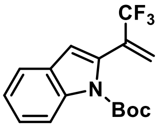

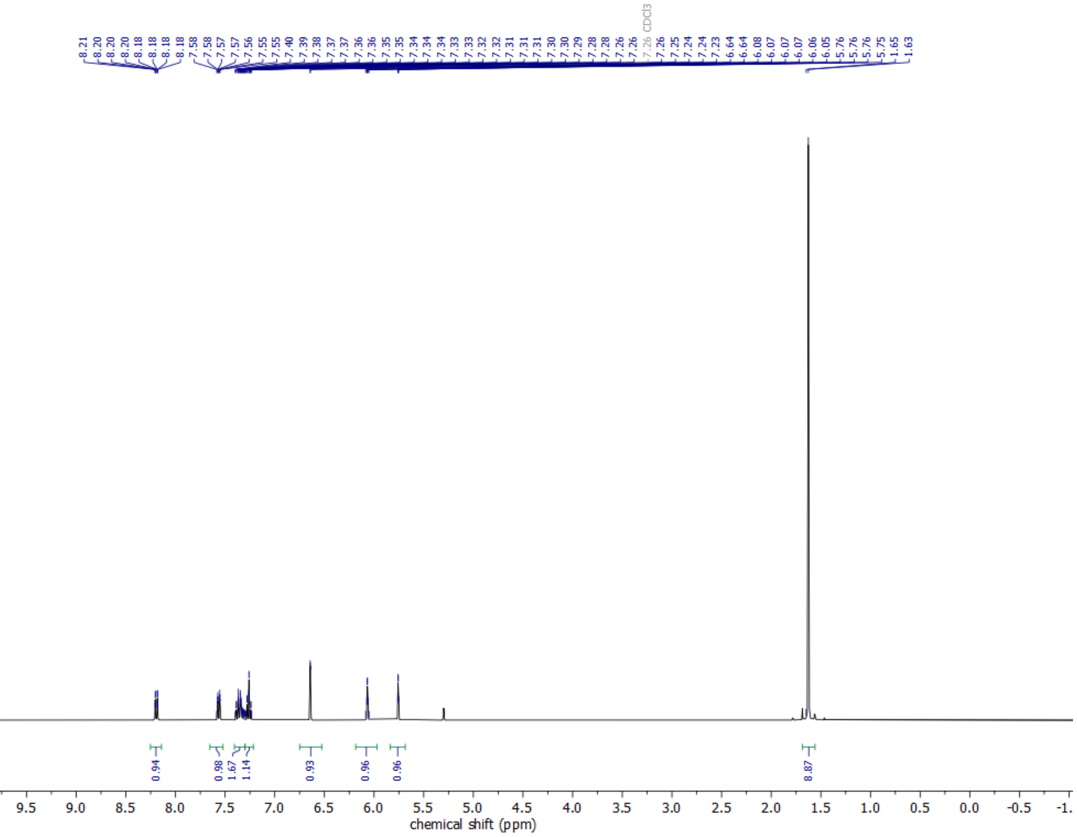

$^{13}C\{^{1}H\}$ NMR (101 MHz, $CDCl_3$)

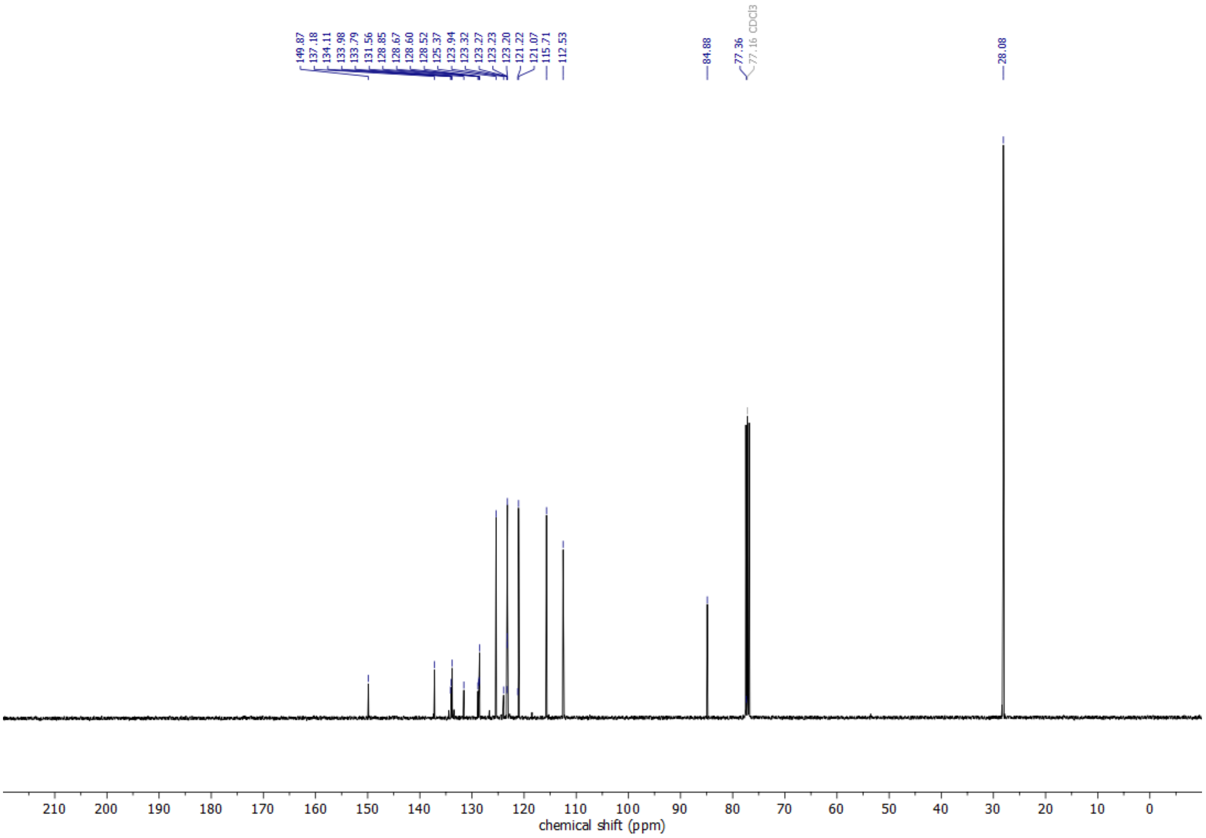

^{19}F NMR (377 MHz, $CDCl_3$)

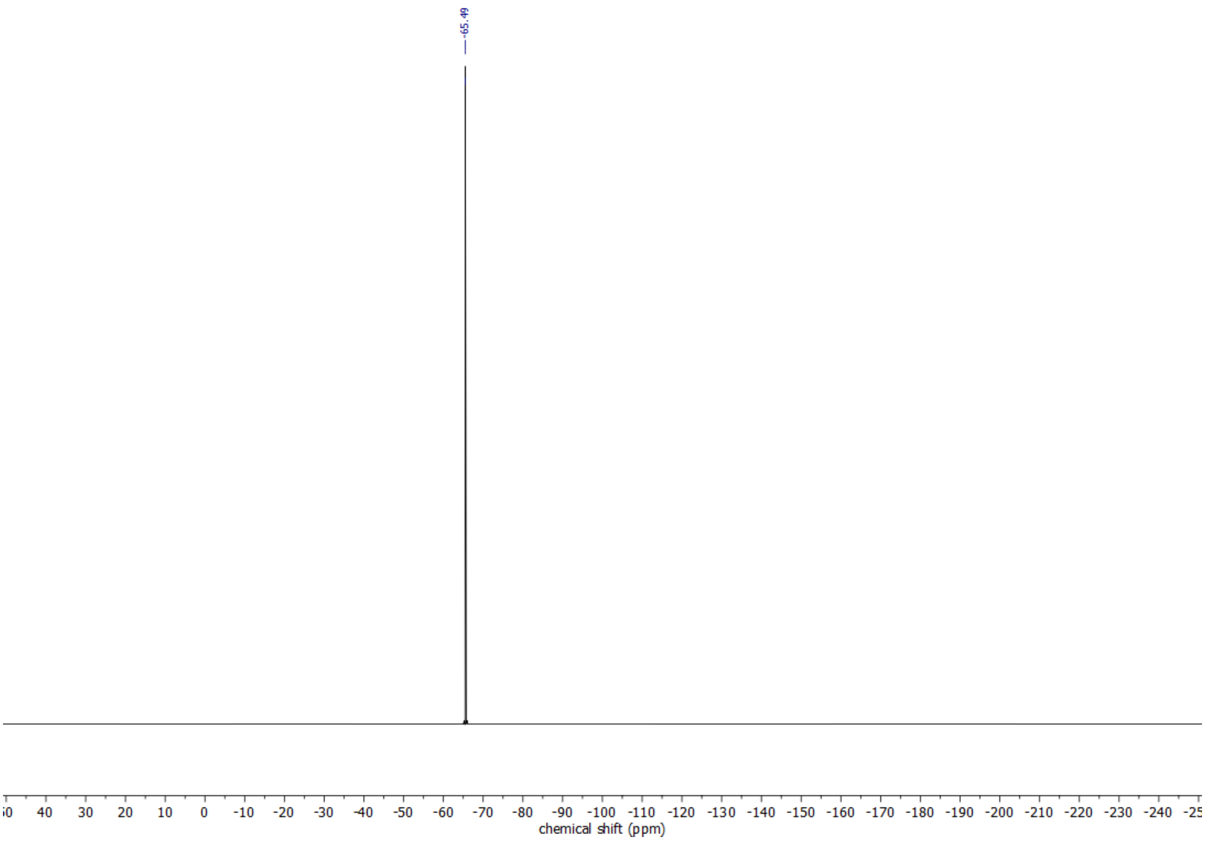

^{1}H NMR (599 MHz, $CDCl_3$)

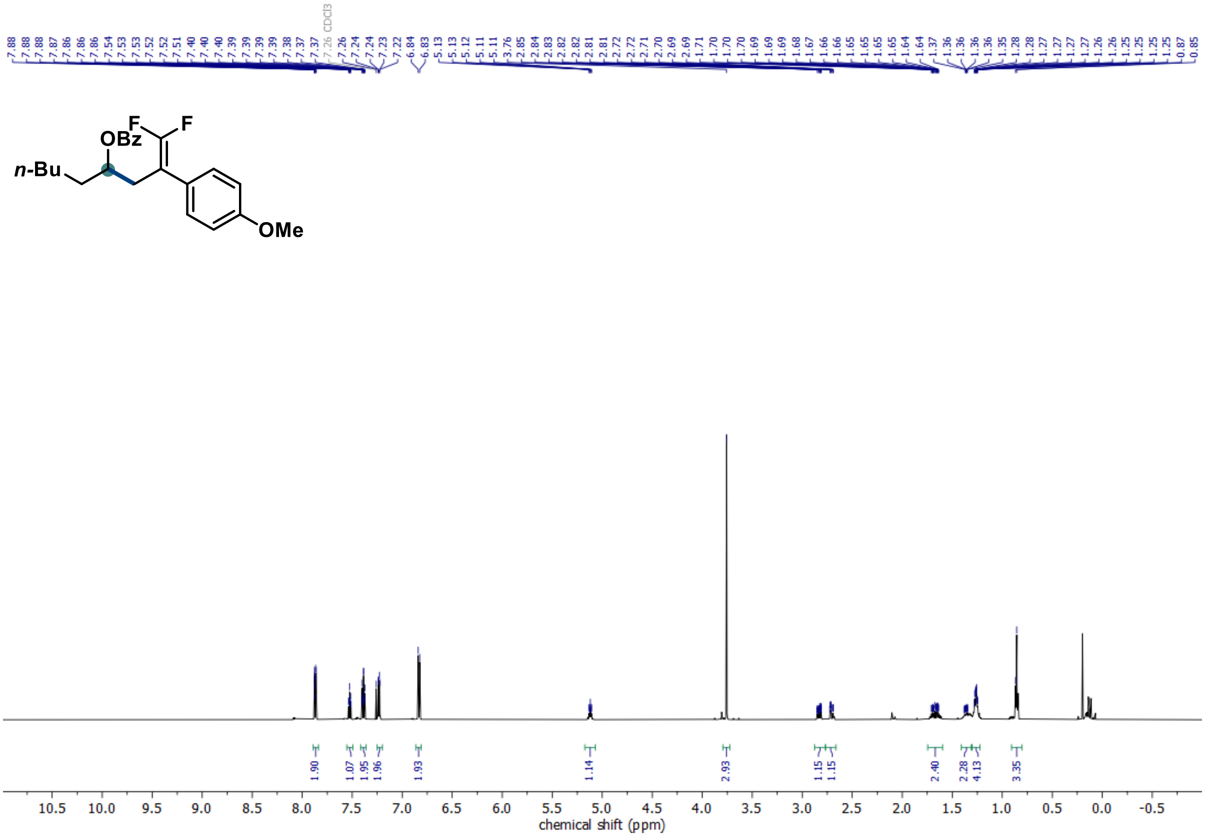

$^{13}C\{^{1}H\}$ NMR (101 MHz, $CDCl_3$)

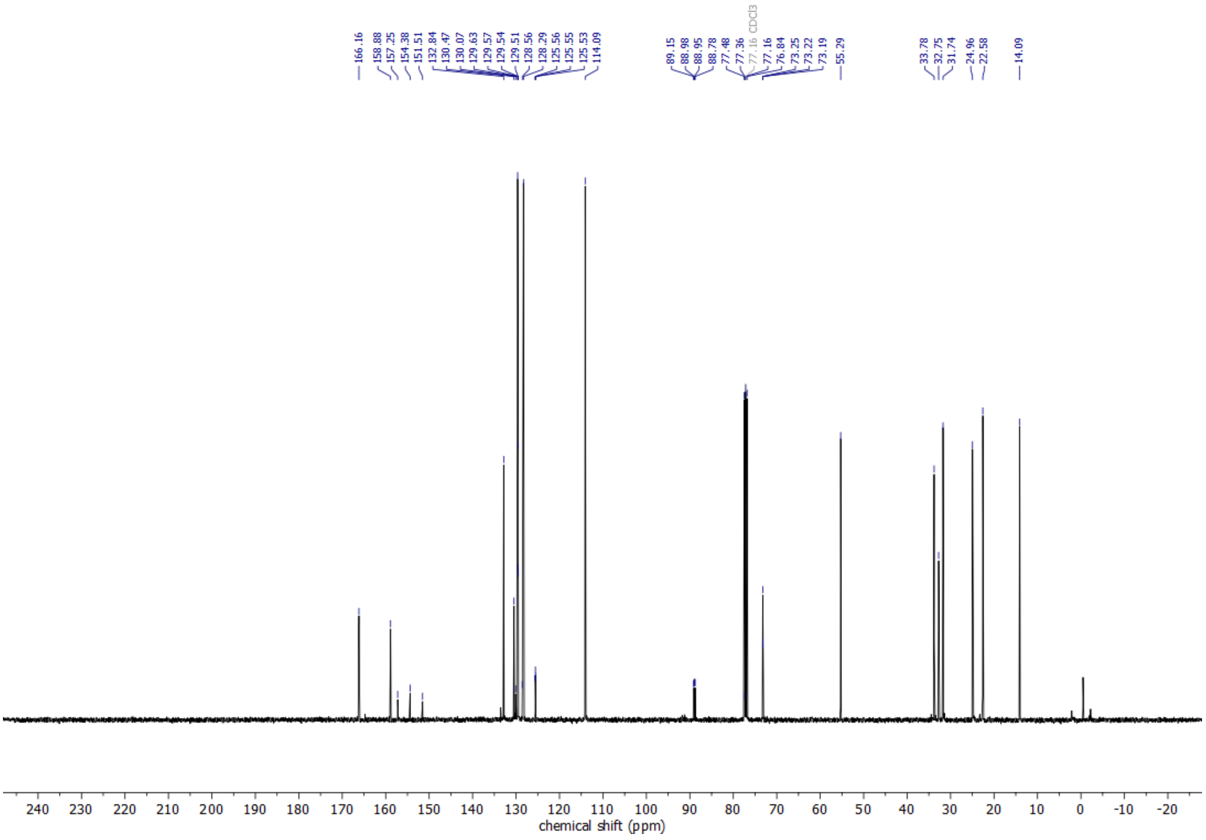

^{19}F NMR (377 MHz, $CDCl_3$)

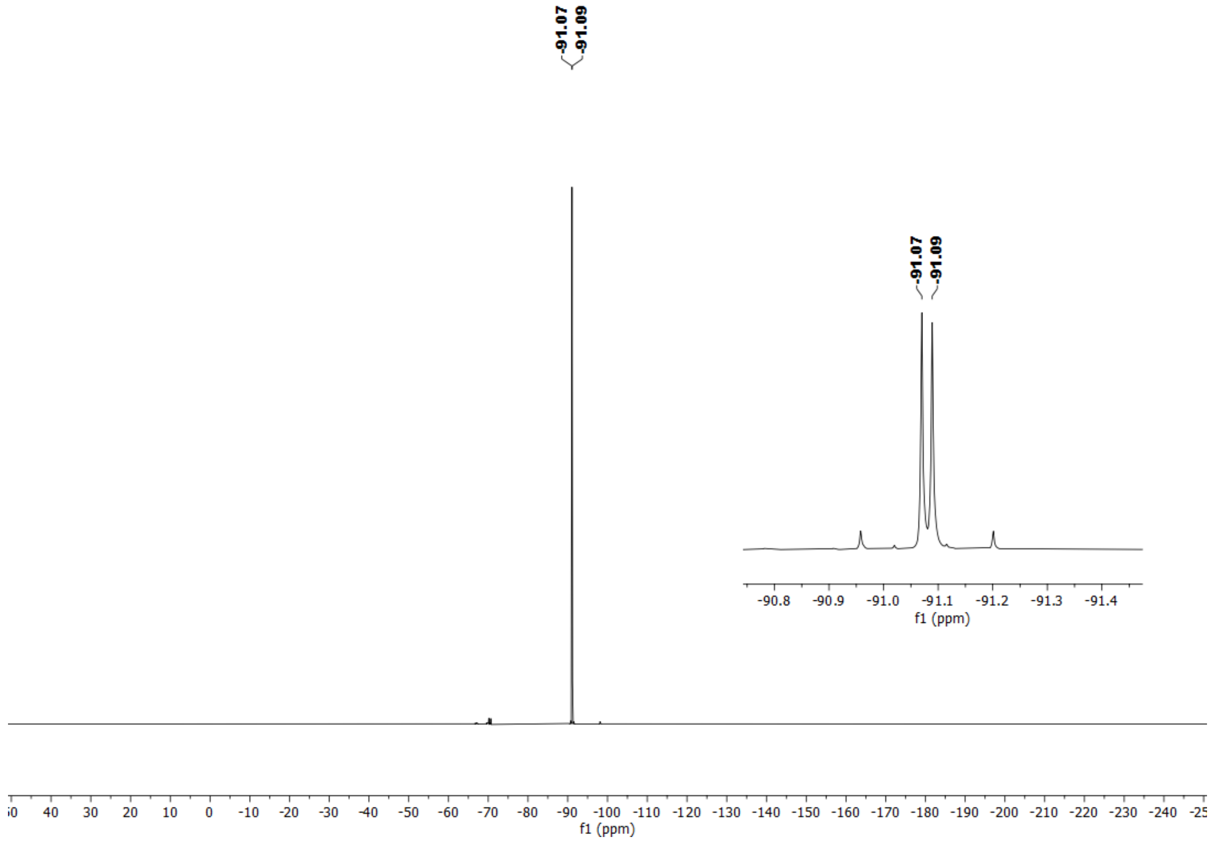

^{1}H NMR (599 MHz, CDCl$_3$)

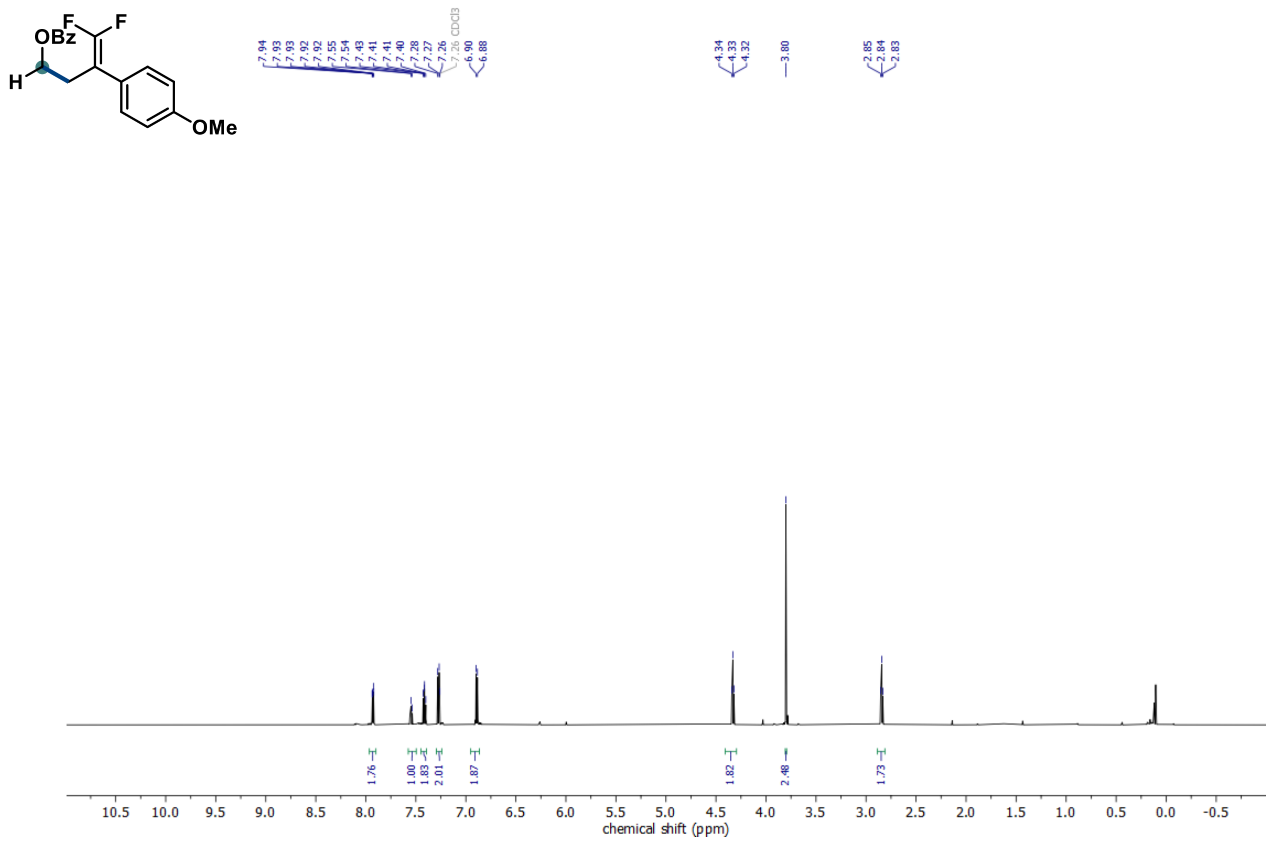

^{13}C{^{1}H} NMR (151 MHz, CDCl$_3$)

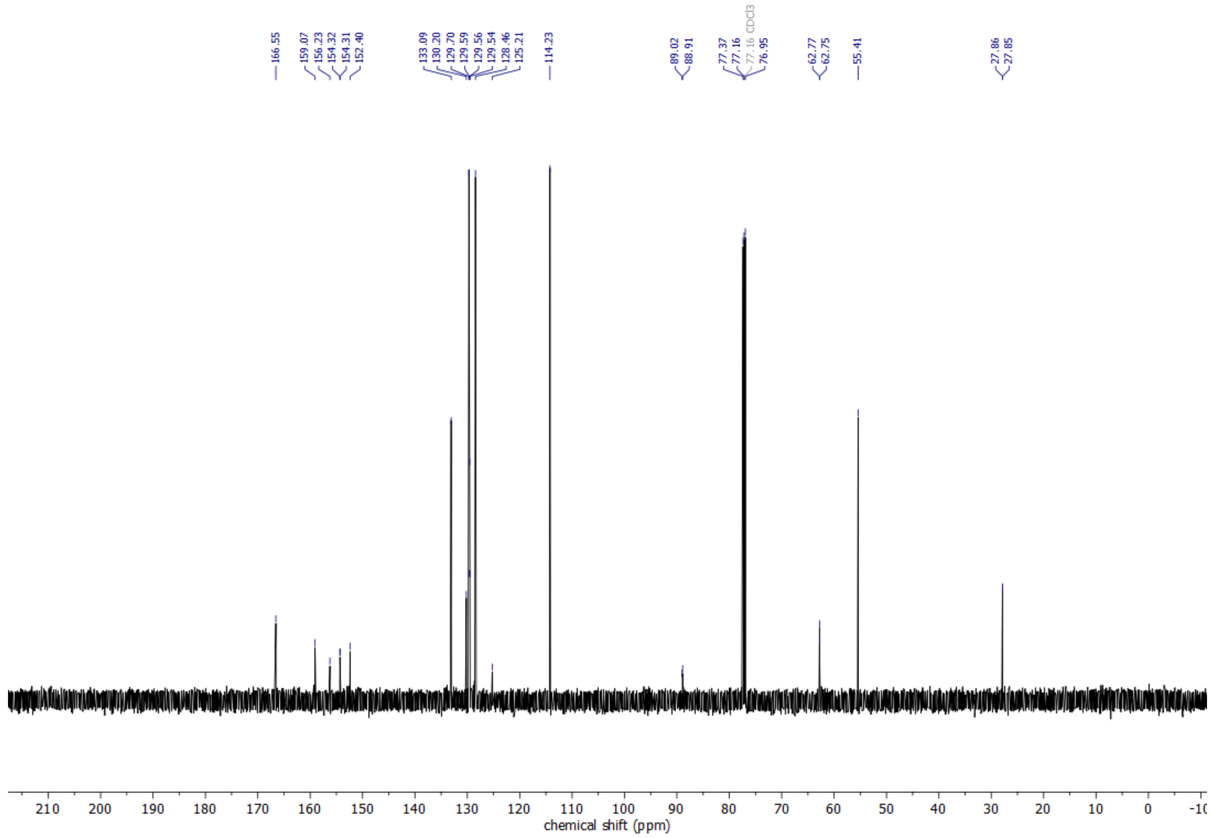

^{19}F NMR (564 MHz, CDCl$_3$)

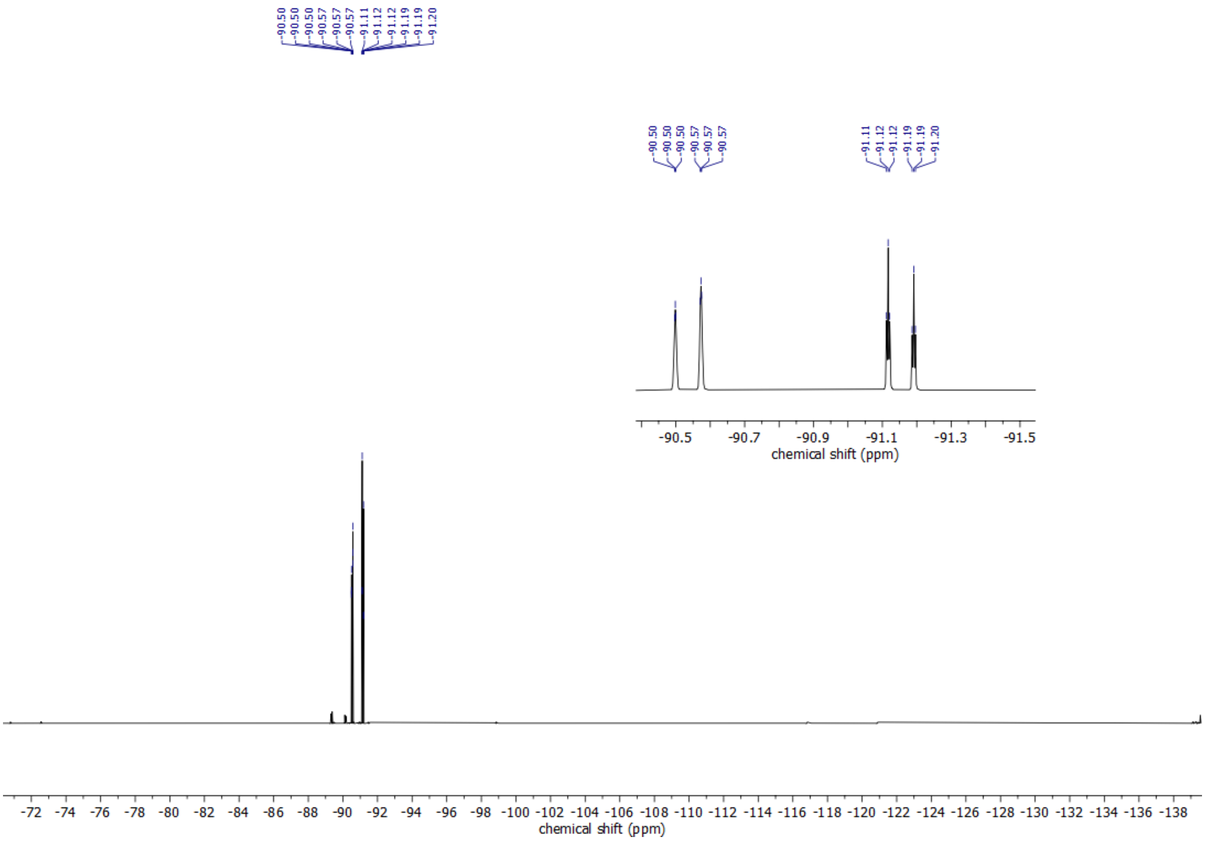

^{1}H NMR (599 MHz, CDCl$_3$)

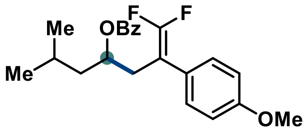

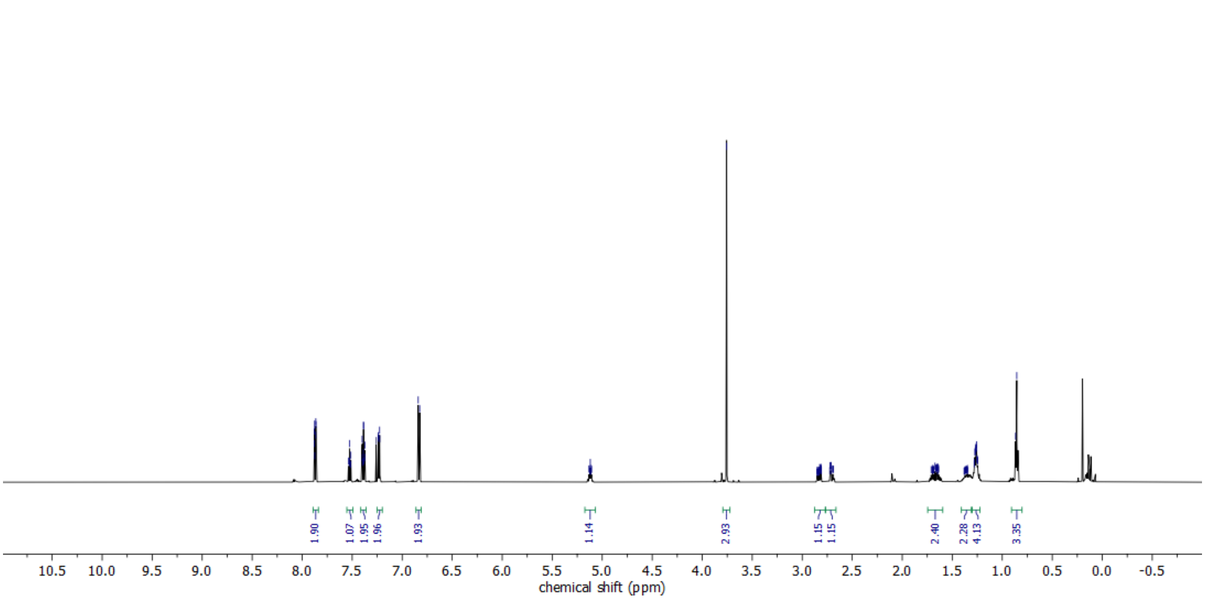

^{13}C{^{1}H} NMR (101 MHz, CDCl$_3$)

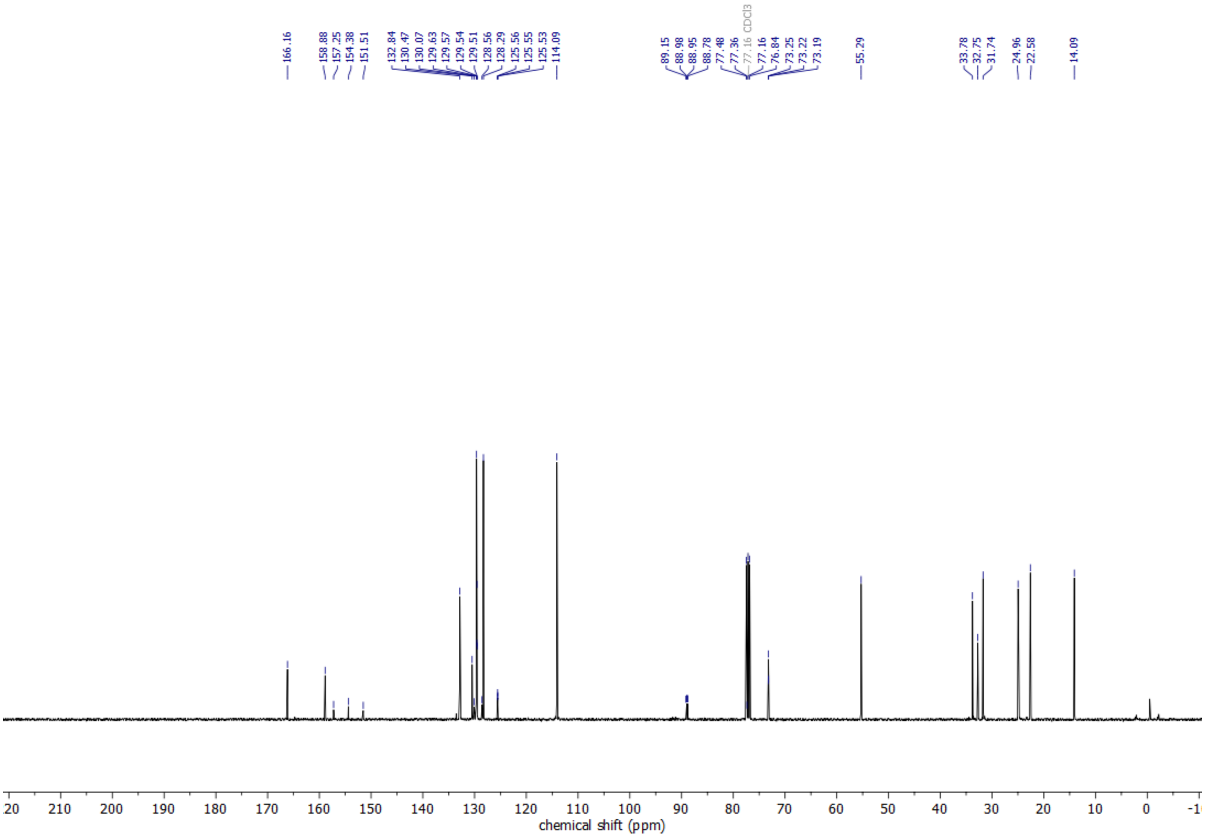

^{19}F NMR (377 MHz, $CDCl_3$)

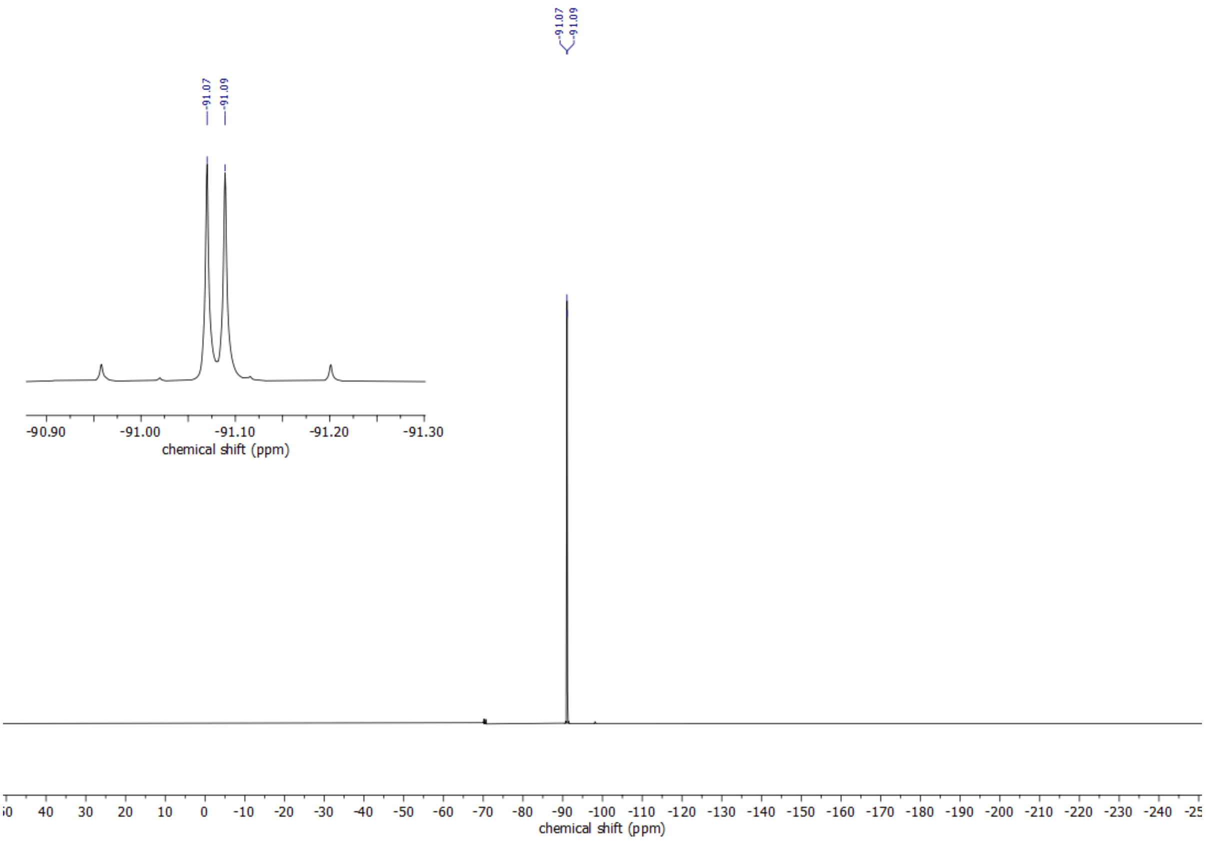

^{1}H NMR (500 MHz, $CDCl_3$)

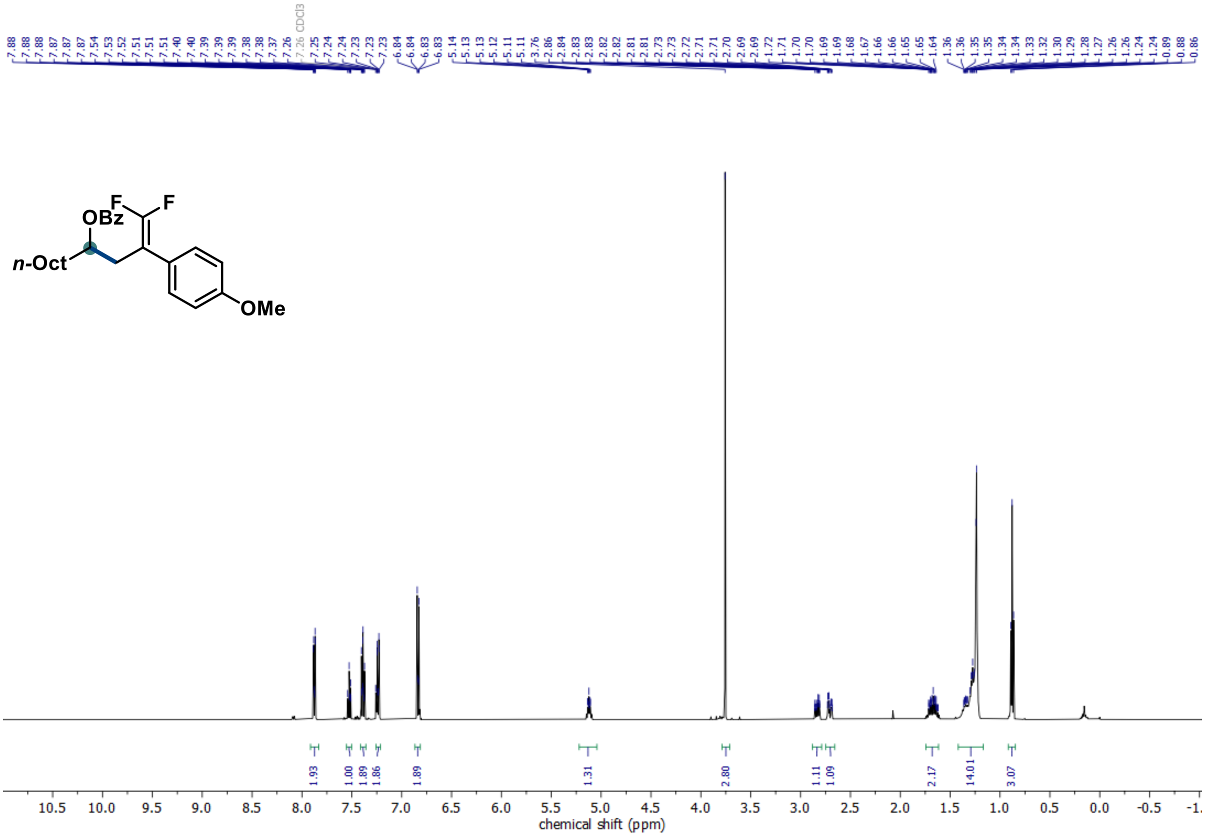

$^{13}C\{^{1}H\}$ NMR (126 MHz, $CDCl_3$)

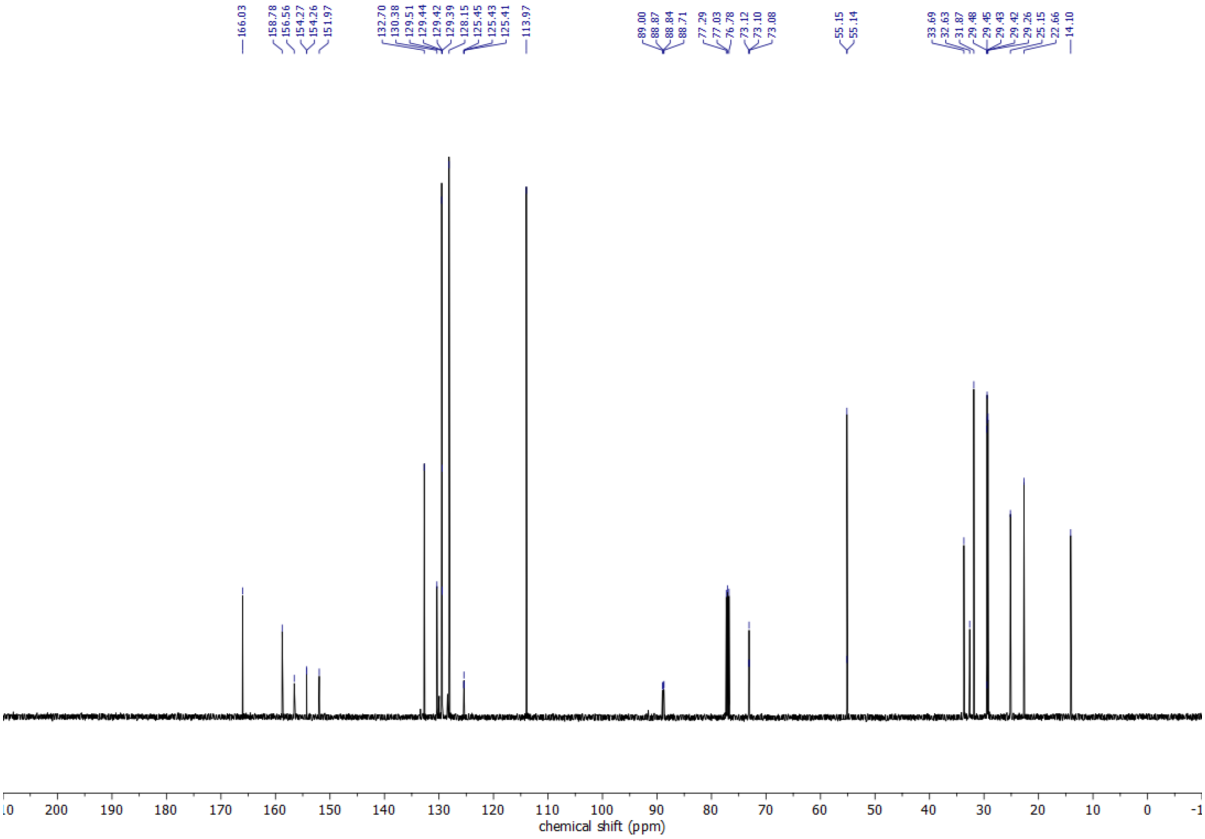

^{19}F NMR (470 MHz, $CDCl_3$)

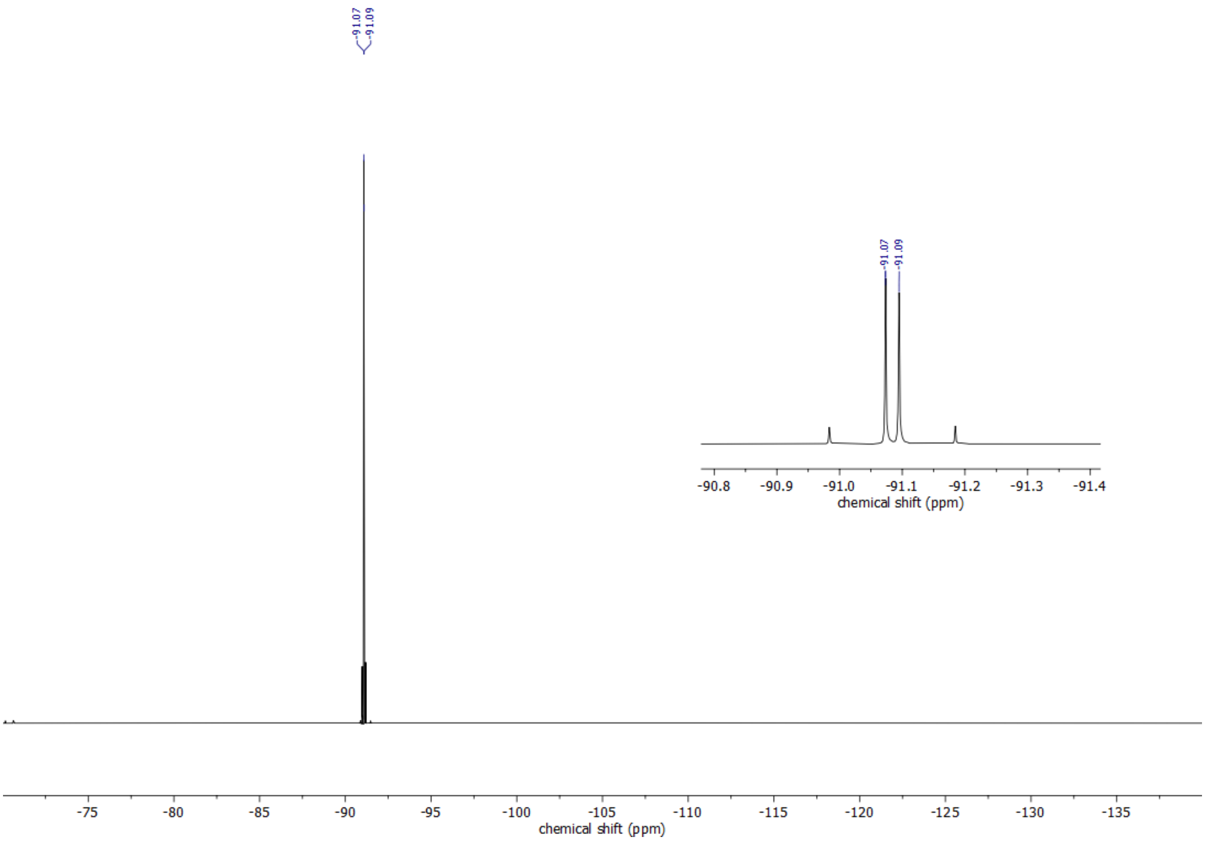

^{1}H NMR (599 MHz, $CDCl_3$)

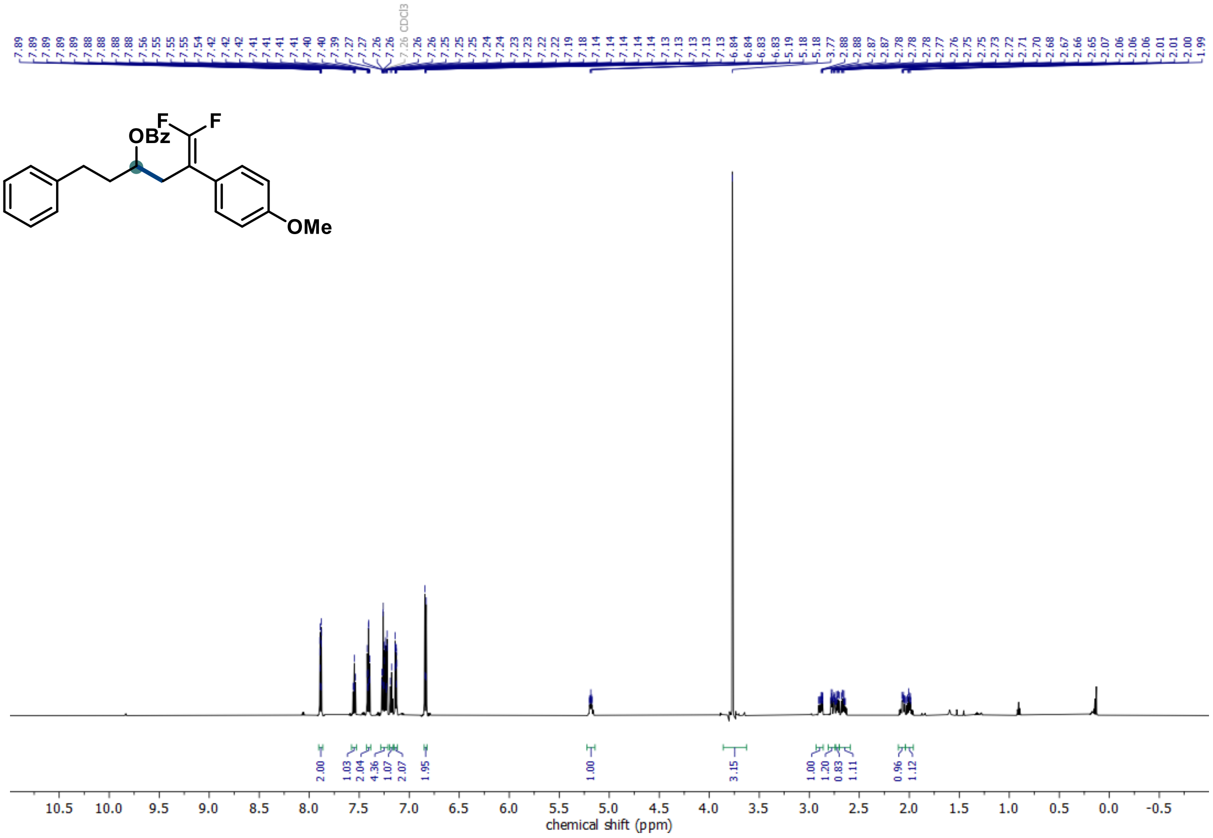

$^{13}C\{^{1}H\}$ NMR (151 MHz, $CDCl_3$)

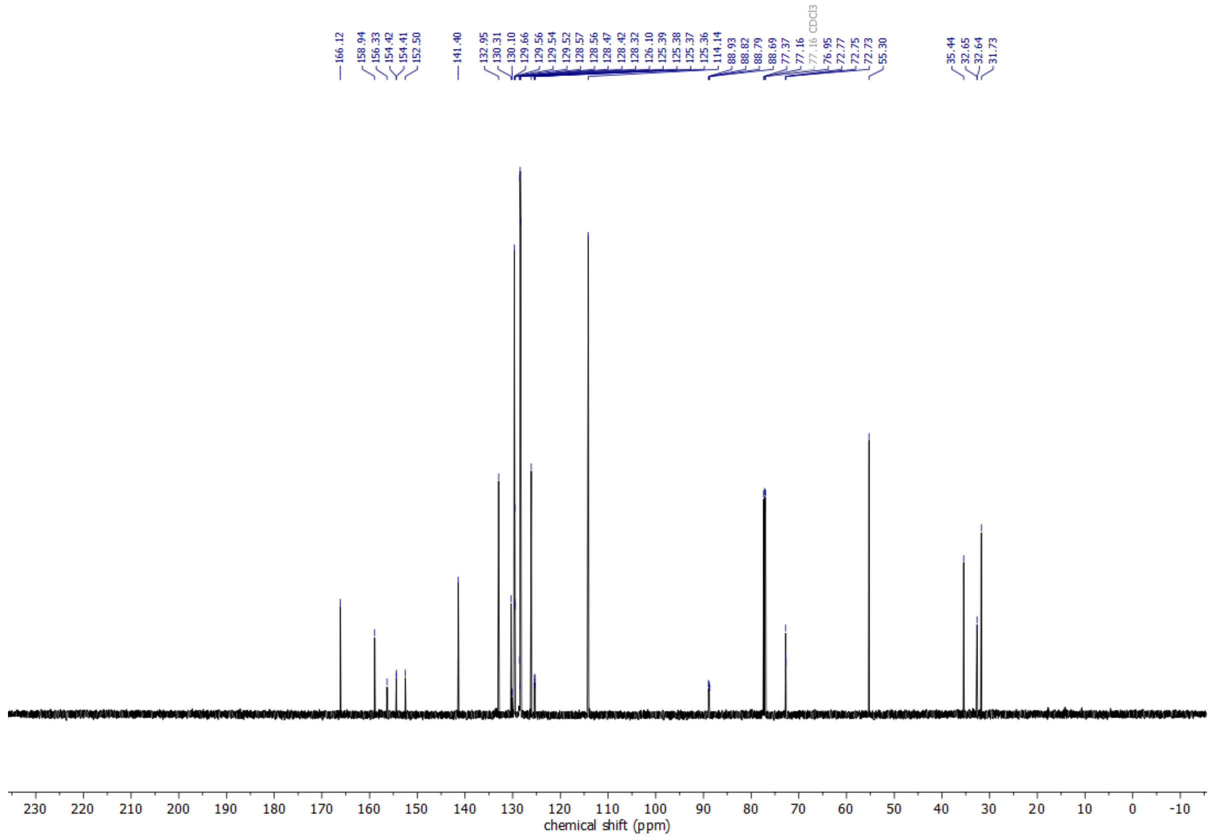

^{19}F NMR (564 MHz, $CDCl_3$)

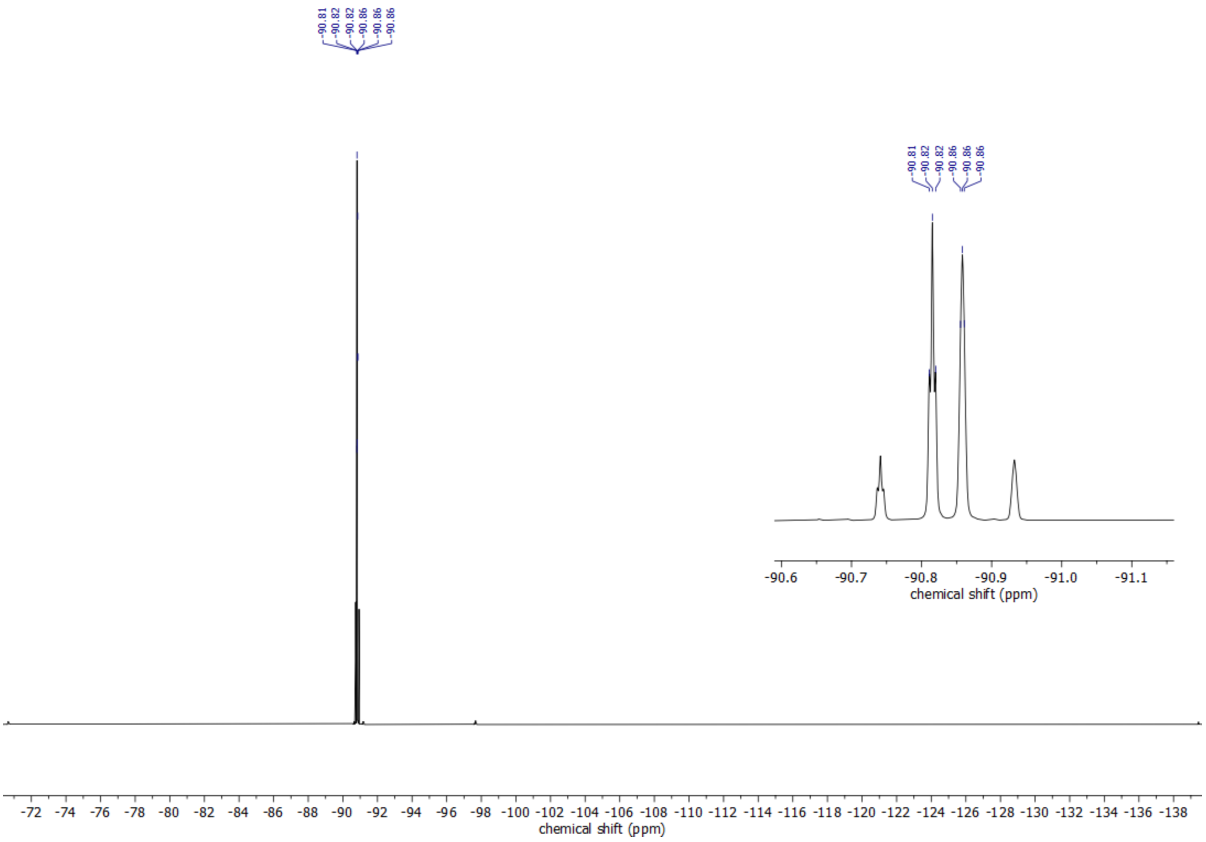

^{1}H NMR (599 MHz, $CDCl_3$)

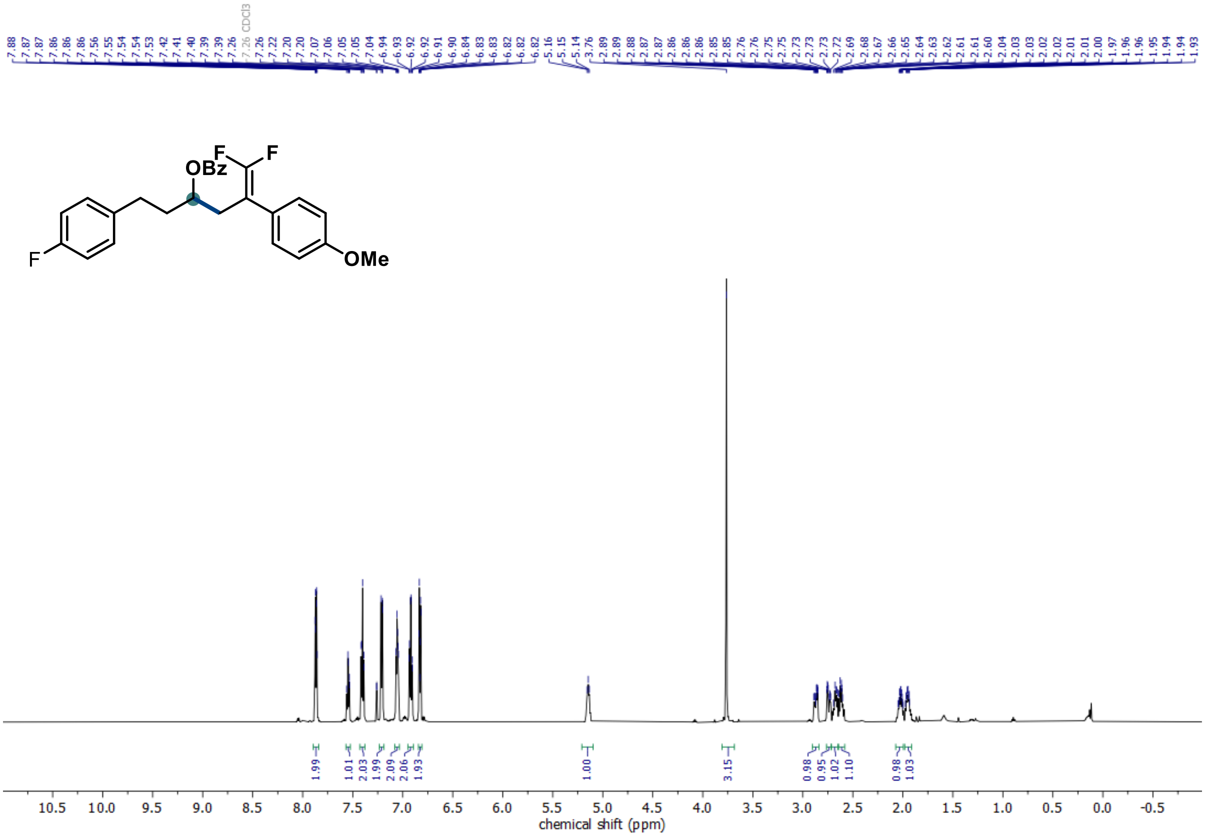

$^{13}C\{^{1}H\}$ NMR (151 MHz, $CDCl_3$)

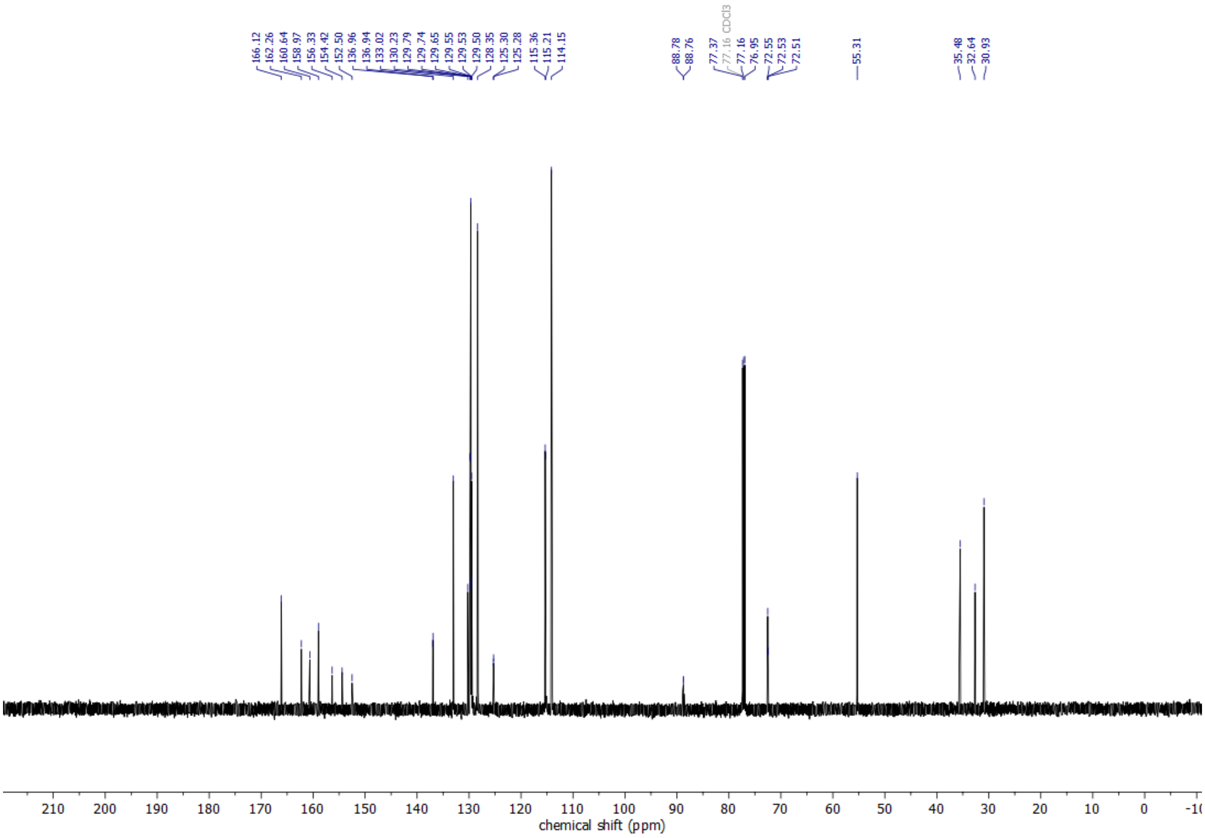

^{19}F NMR (564 MHz, CDCl$_3$)

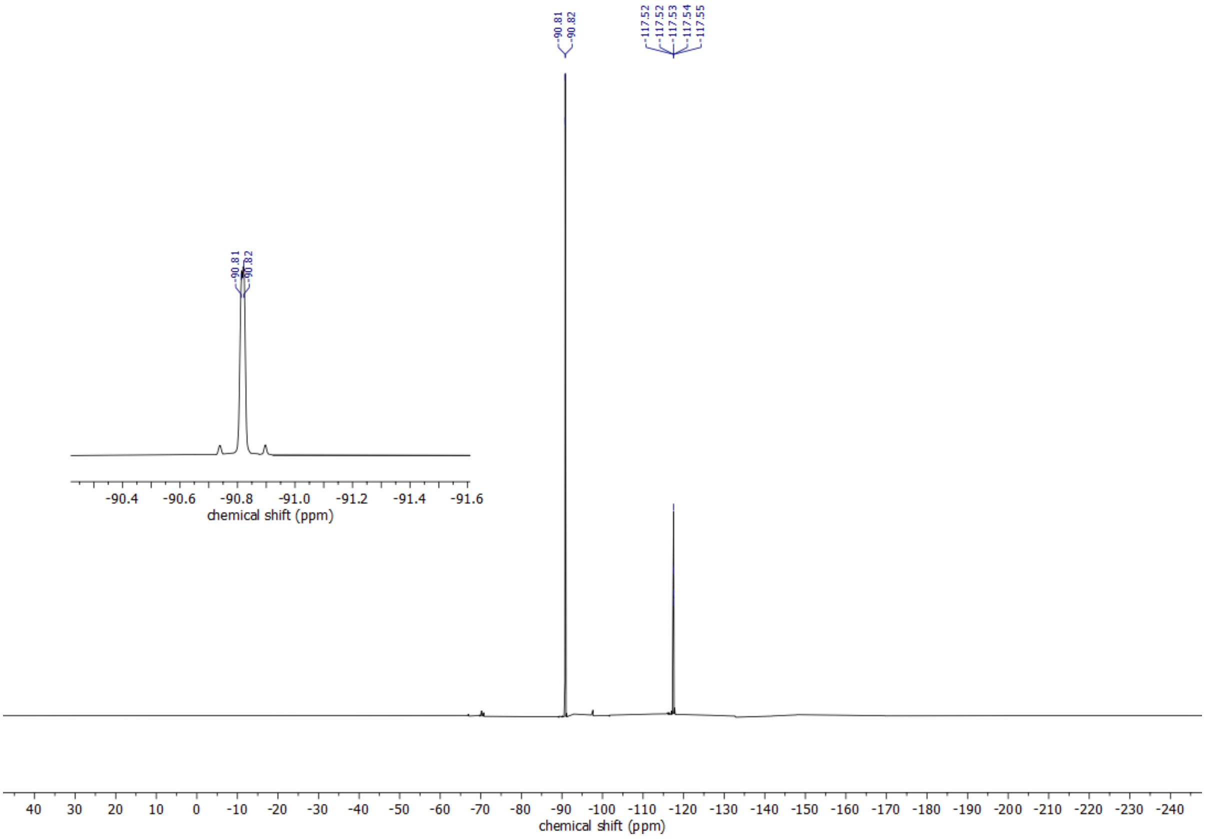

^{1}H NMR (400 MHz, $CDCl_3$)

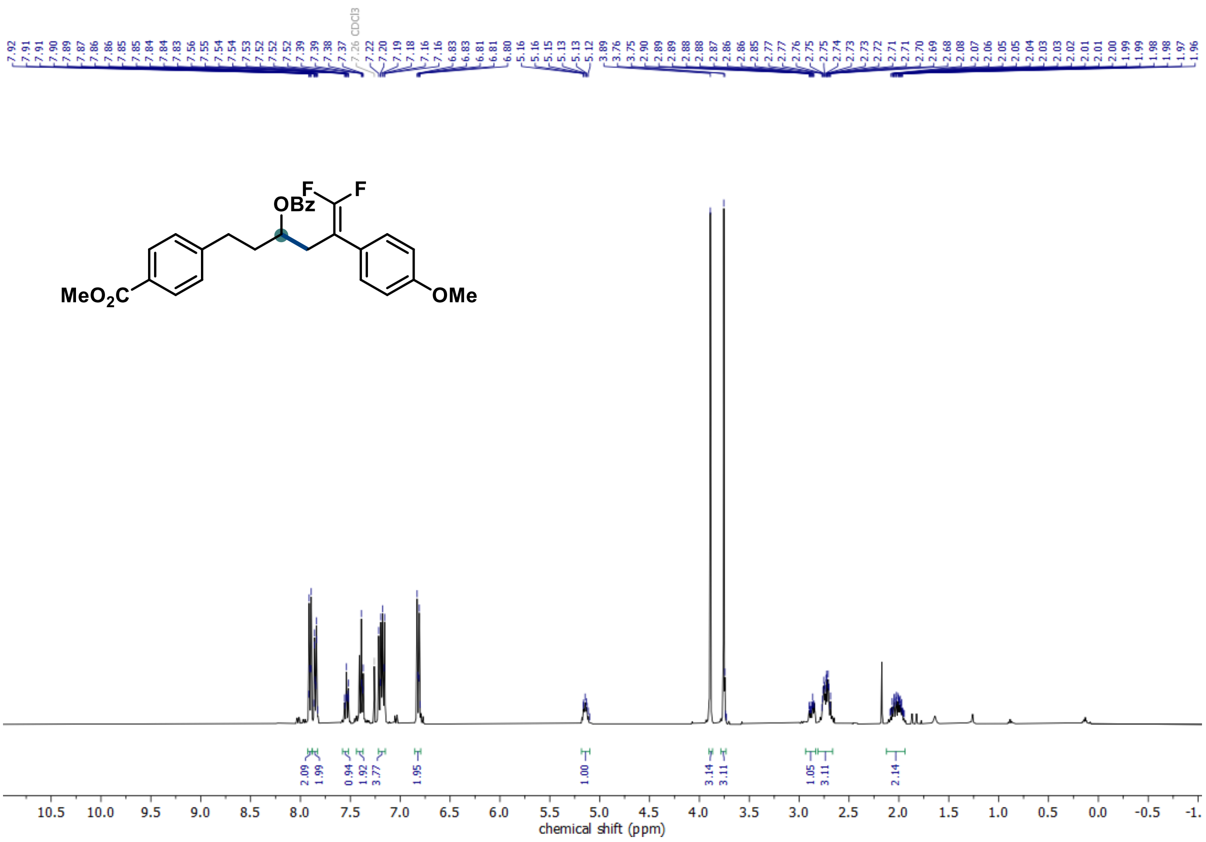

$^{13}C\{^{1}H\}$ NMR (101 MHz, $CDCl_3$)

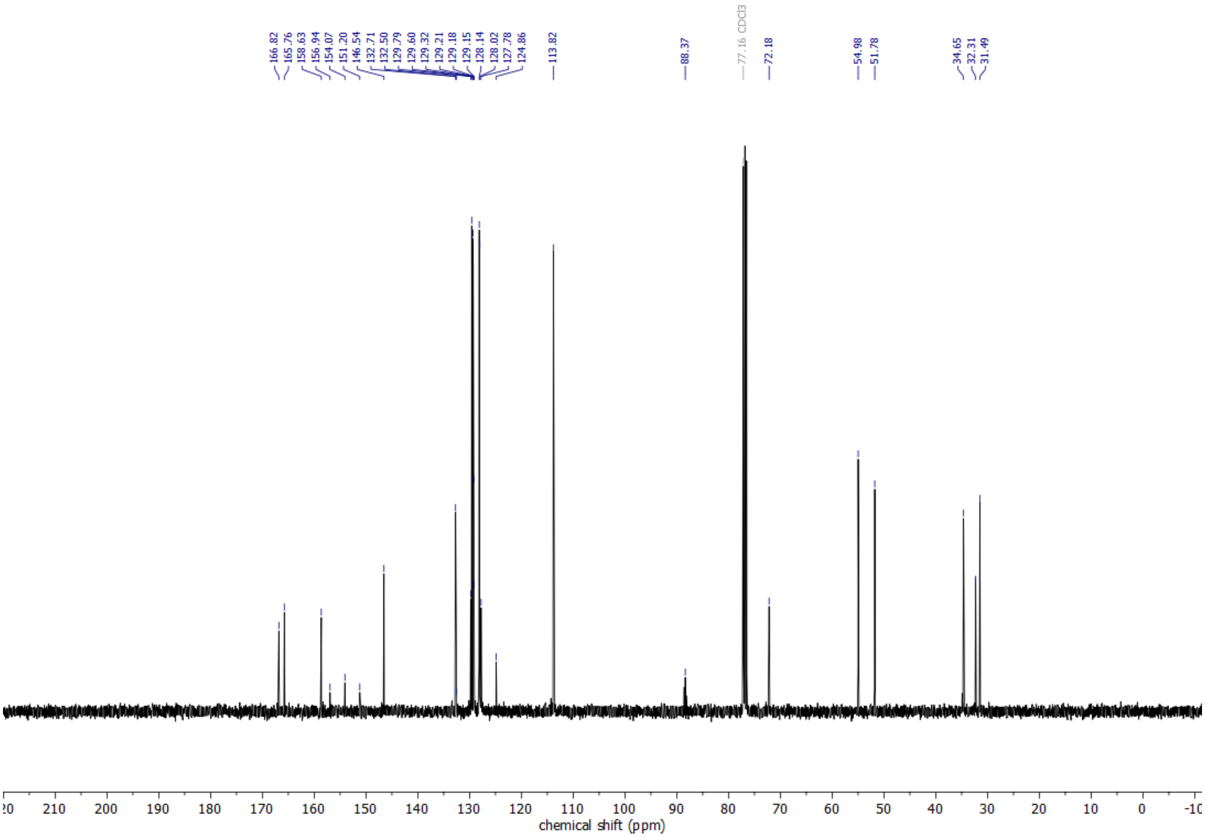

$^{19}F\{^{1}H\}$ NMR (377 MHz, $CDCl_3$)

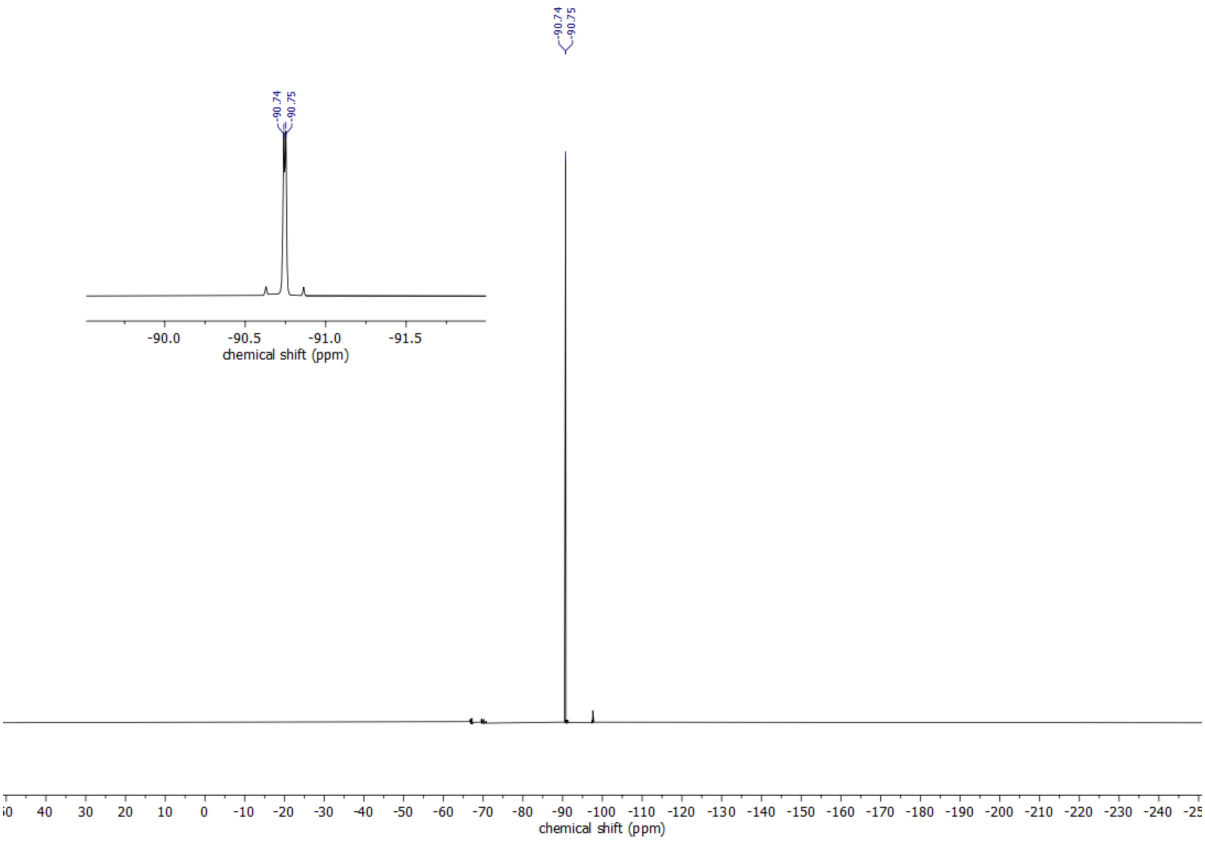

^{1}H NMR (400 MHz, $CDCl_3$)

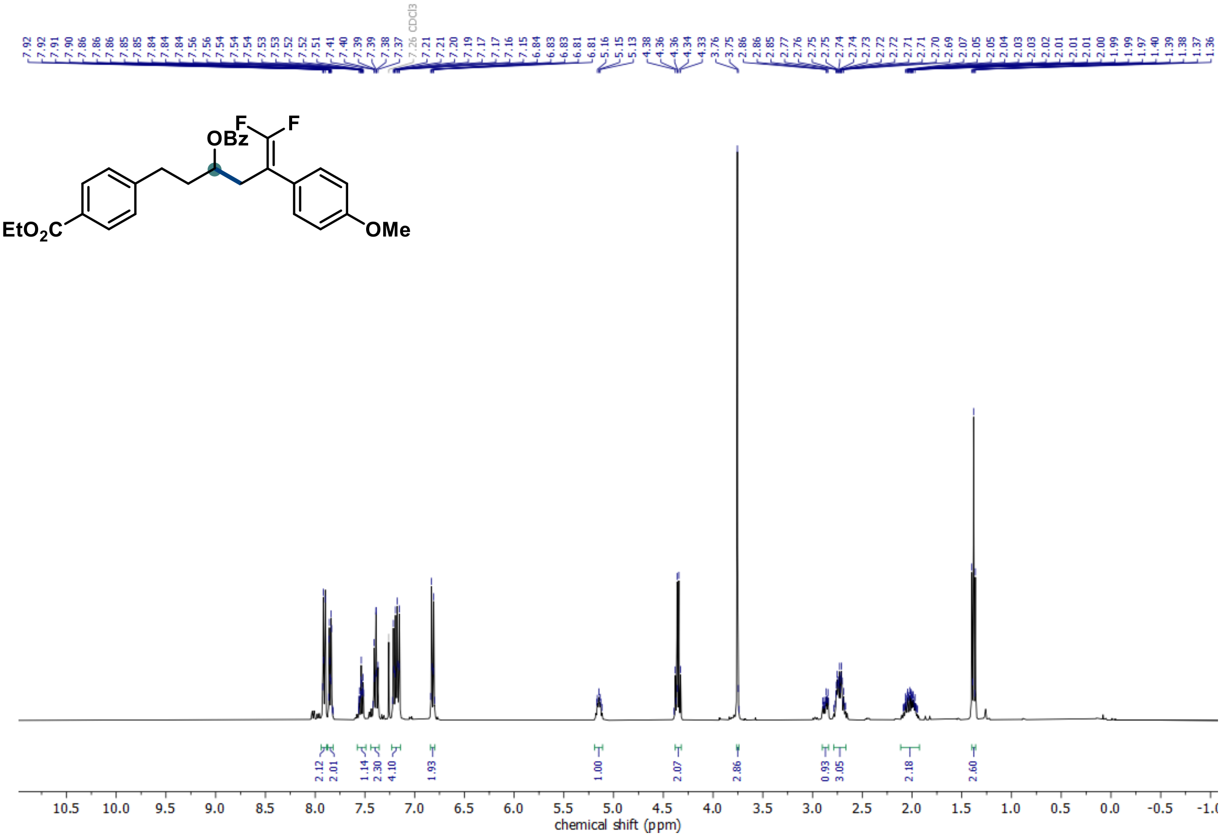

$^{13}C\{^{1}H\}$ NMR (101 MHz, $CDCl_3$)

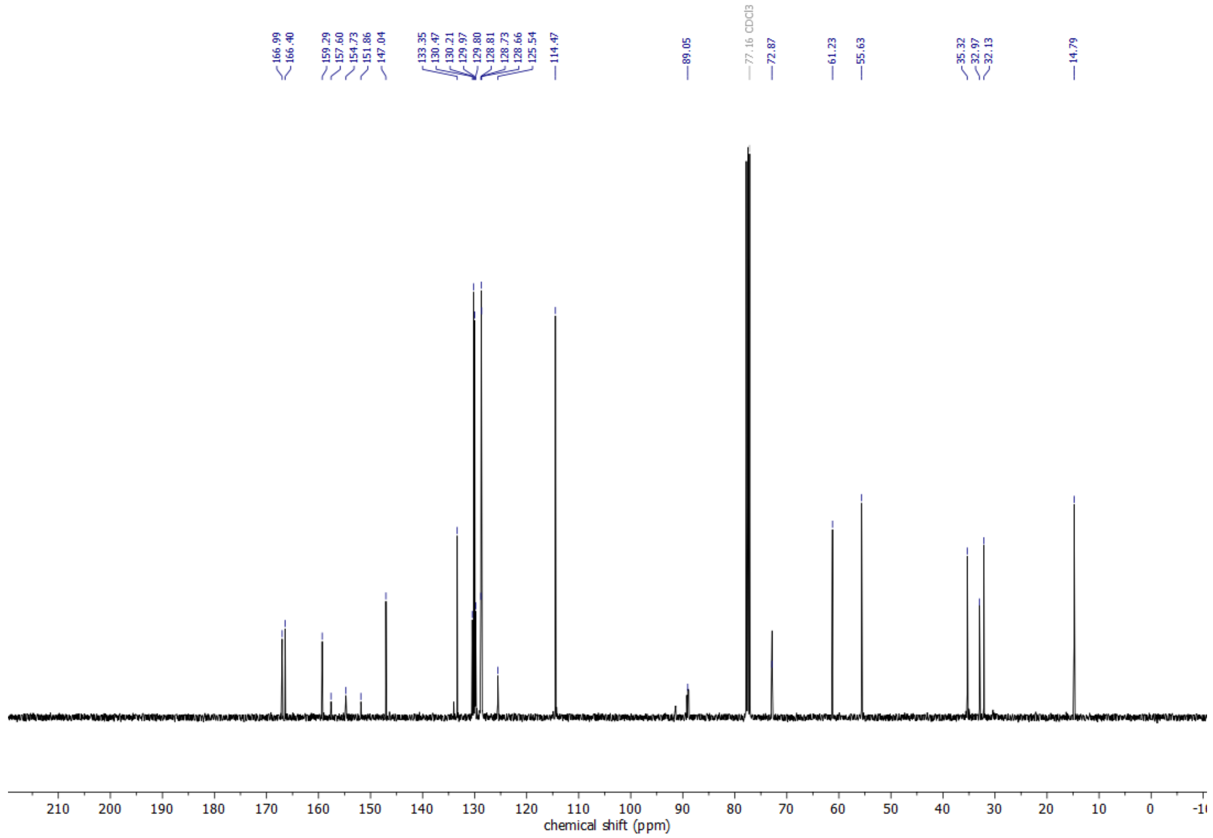

$^{19}F\{^1H\}$ NMR (377 MHz, $CDCl_3$)

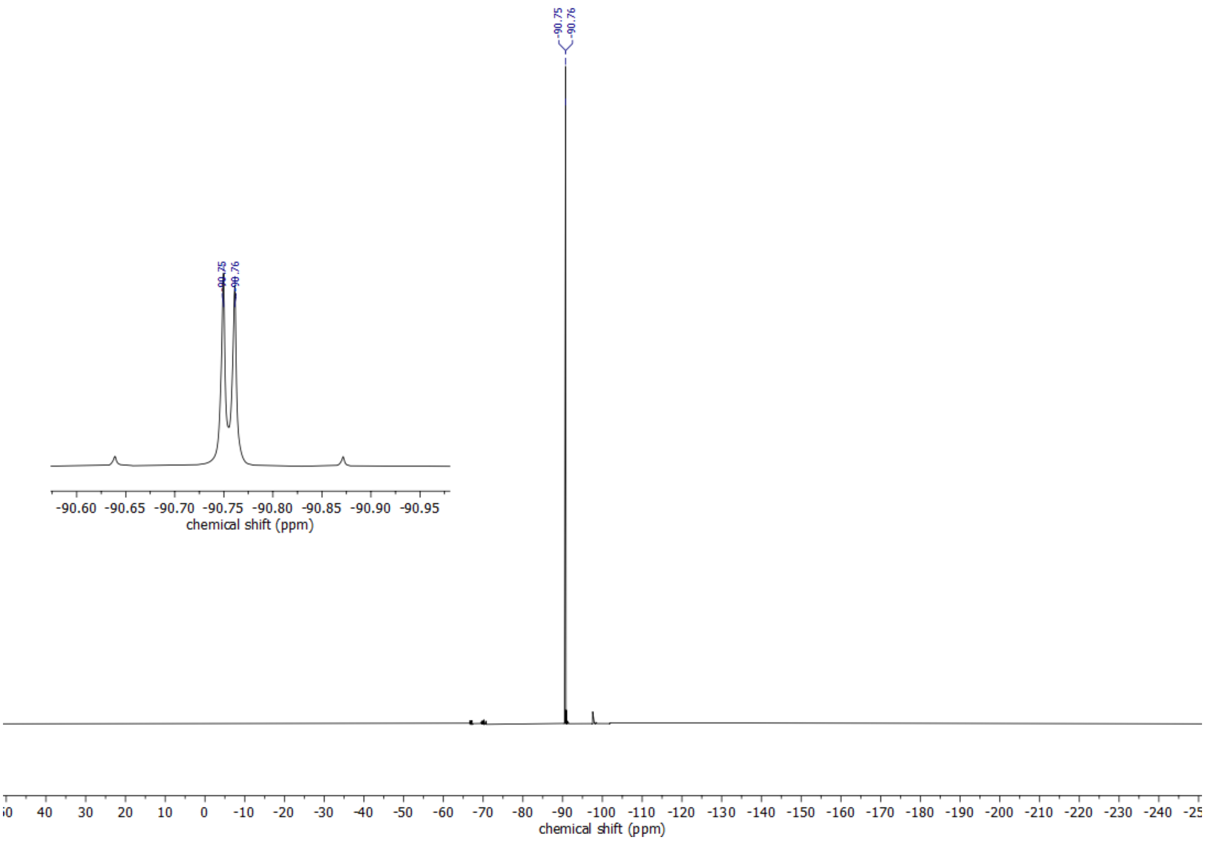

^{1}H NMR (500 MHz, $CDCl_3$)

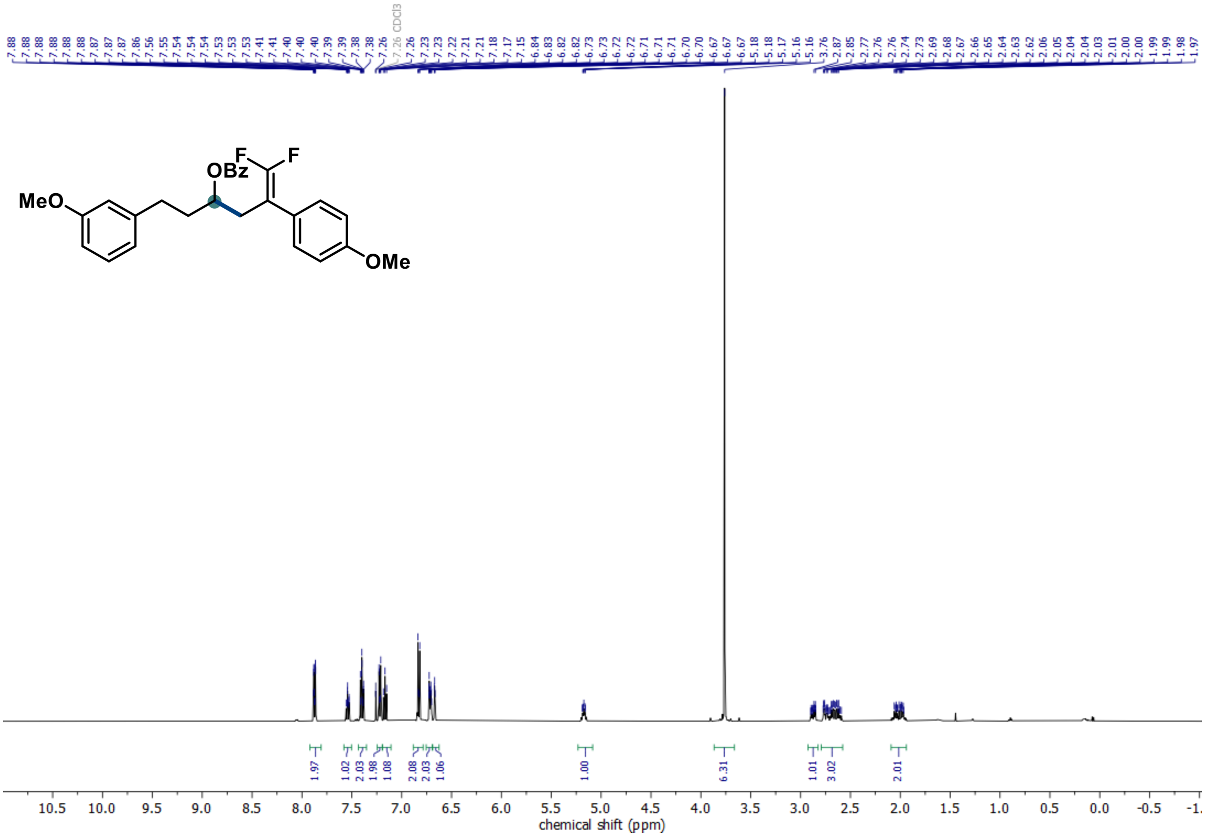

$^{13}C\{^1H\}$ NMR (126 MHz, $CDCl_3$)

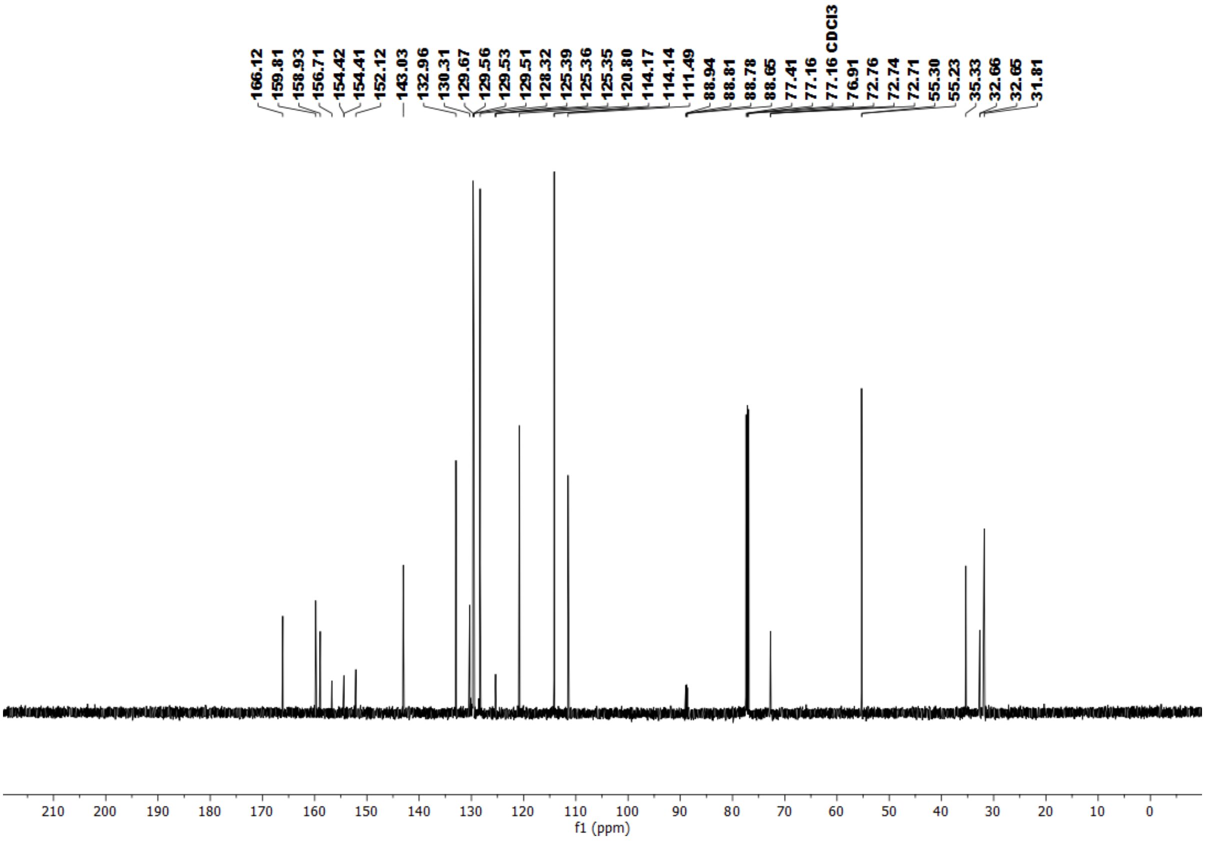

^{19}F NMR (470 MHz, $CDCl_3$)

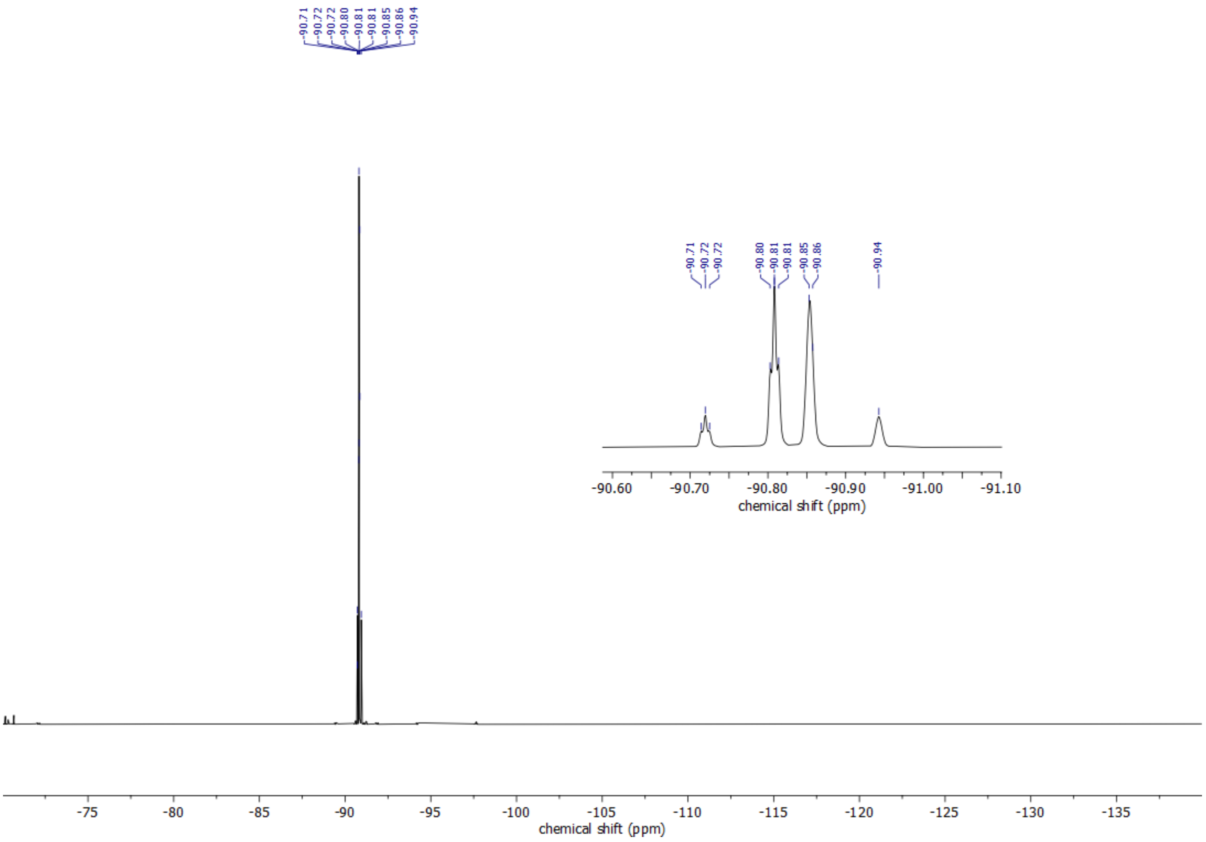

^{1}H NMR (400 MHz, $CDCl_3$)

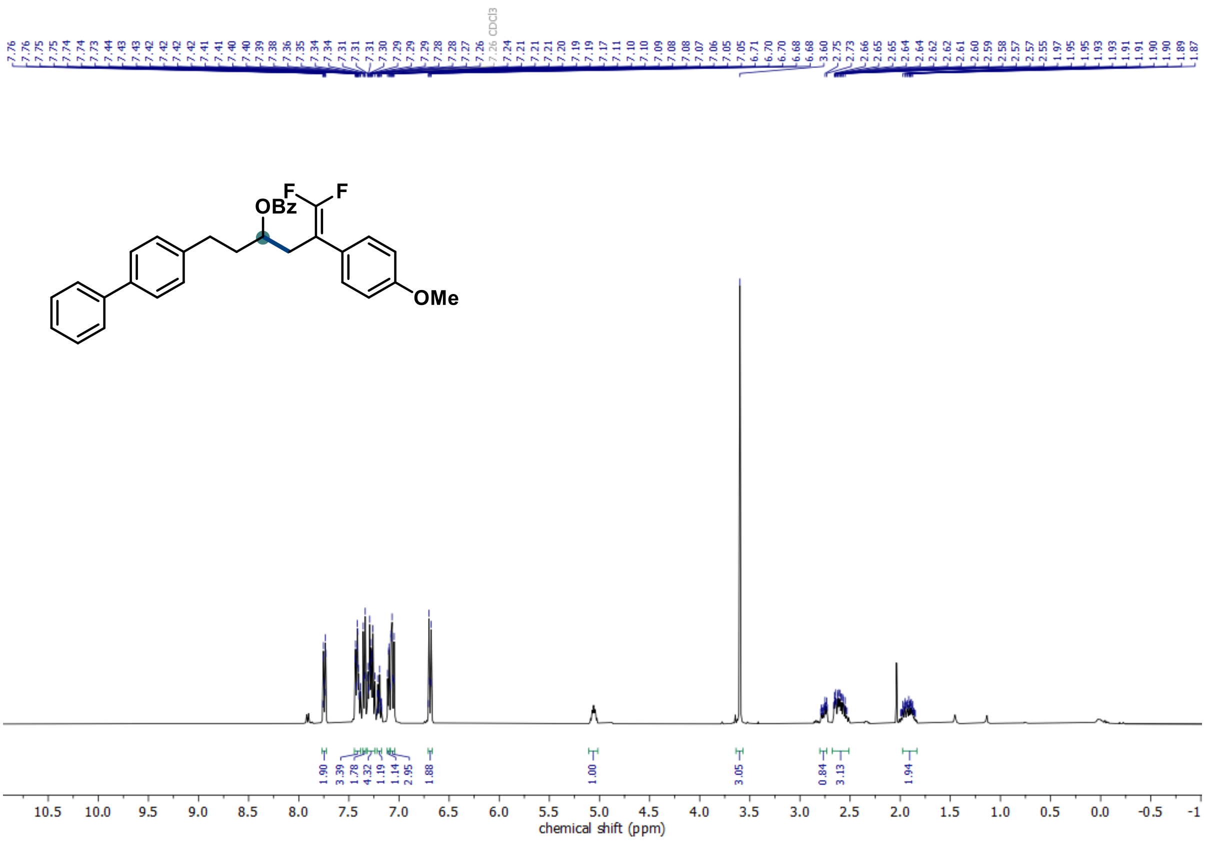

$^{13}C\{^1H\}$ NMR (101MHz, $CDCl_3$)

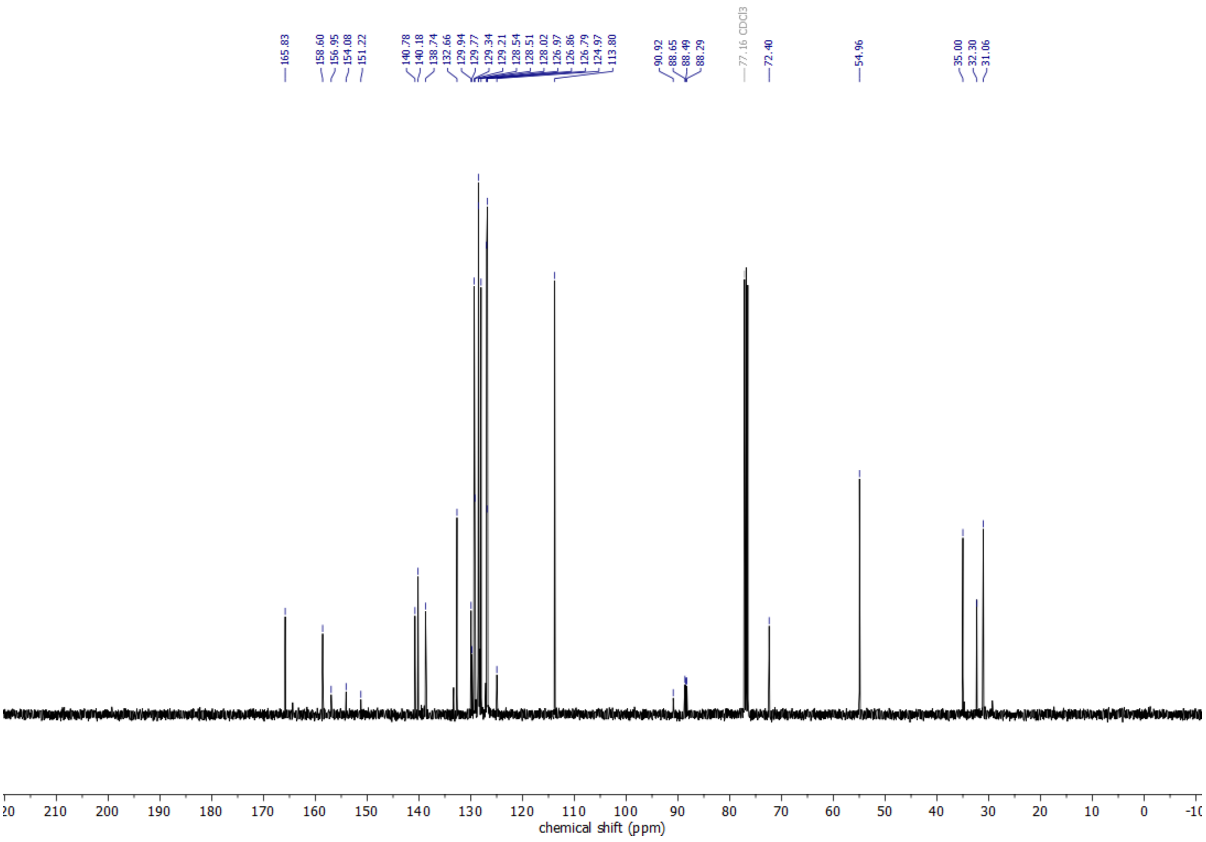

$^{19}F\{^{1}H\}$ NMR (377 MHz, $CDCl_3$)

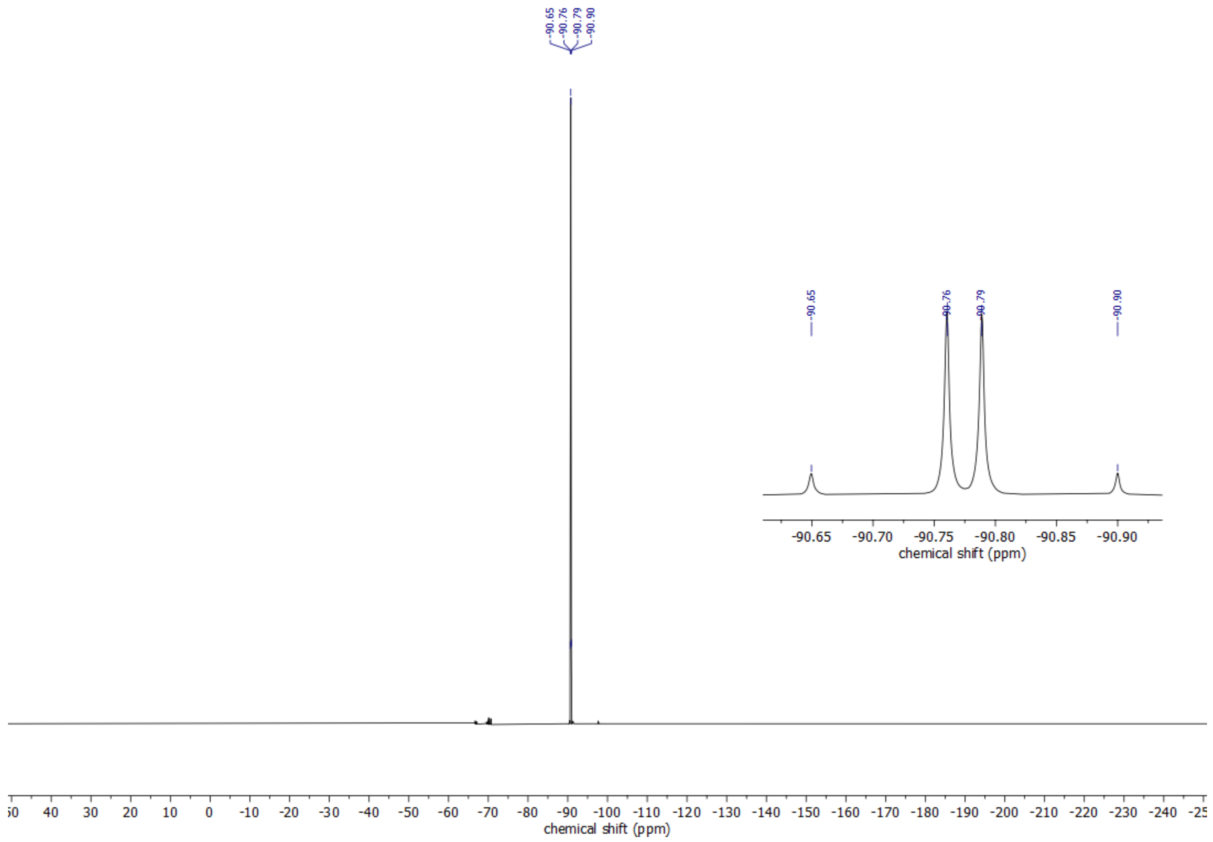

^{1}H NMR (500 MHz, $CDCl_3$)

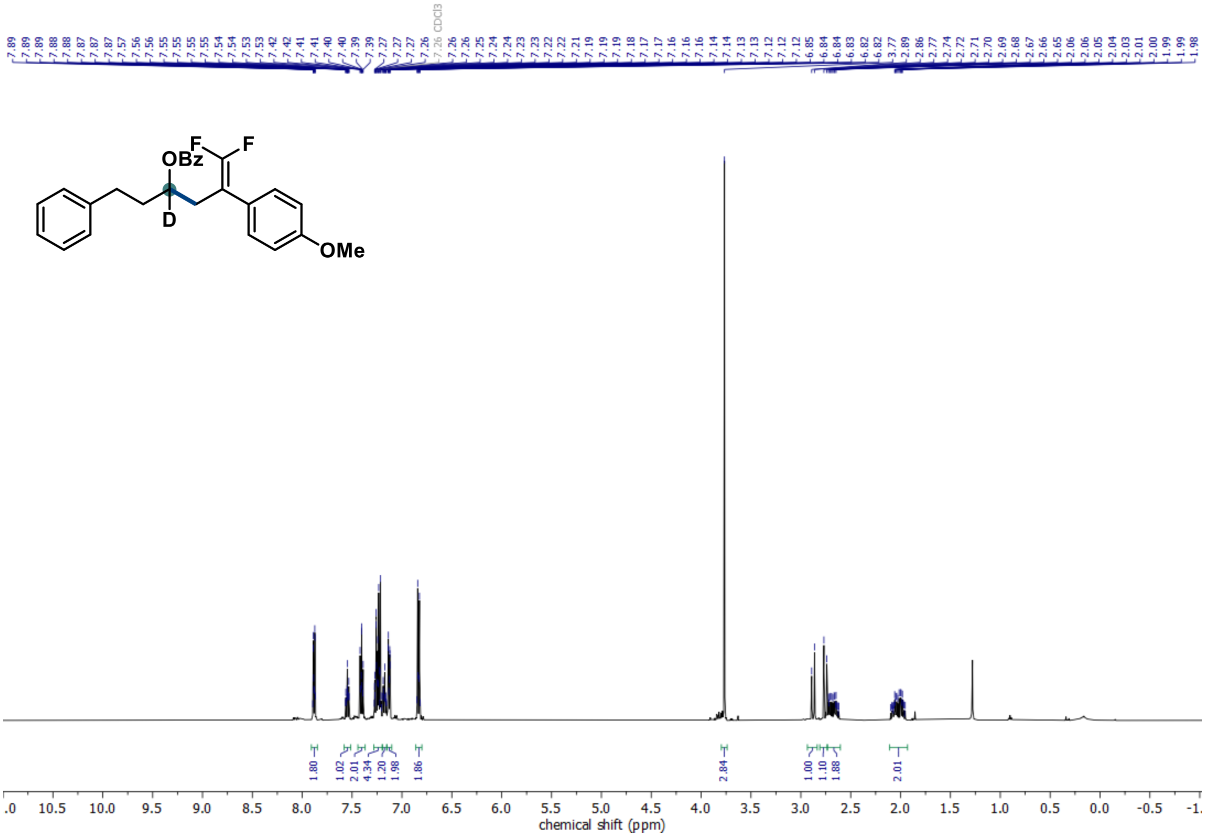

^{13}C{^{1}H, ^{19}F} NMR (126 MHz, $CDCl_3$)

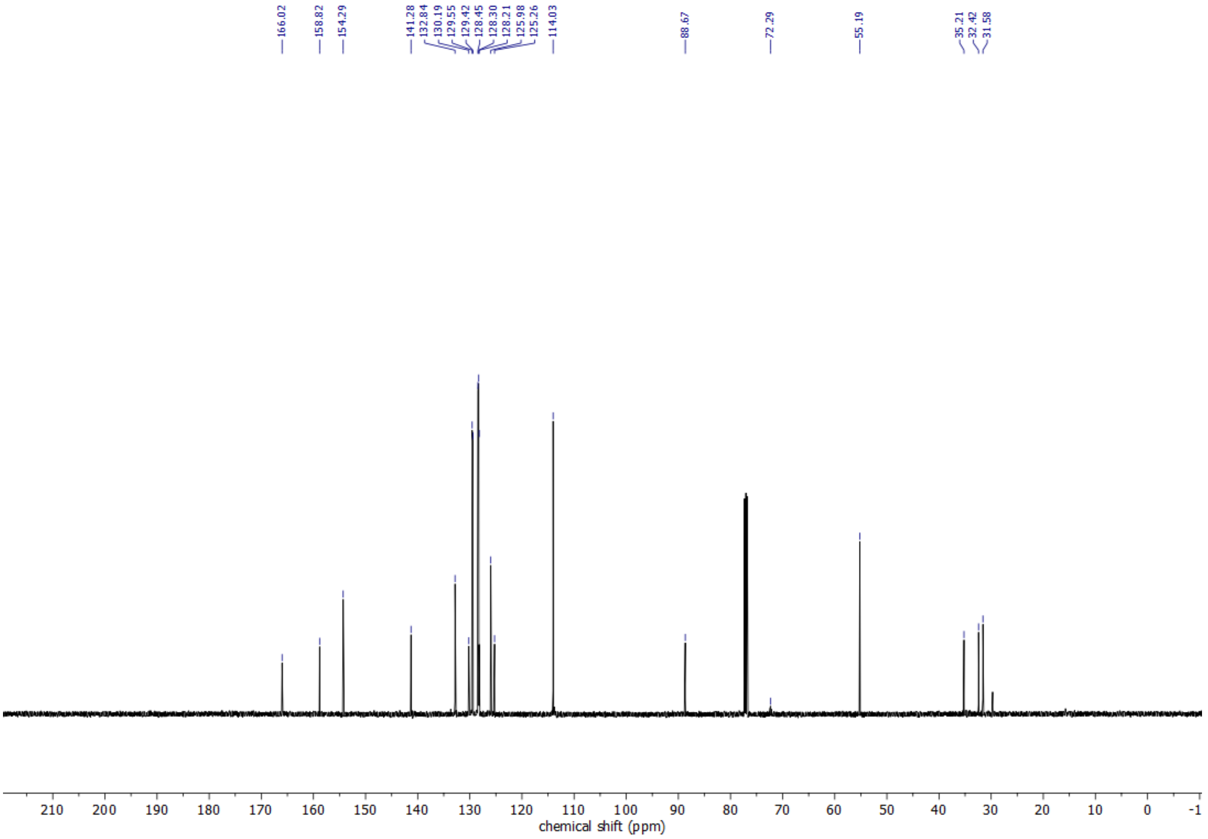

$^{19}F\{^{1}H\}$ NMR (377 MHz, $CDCl_3$)

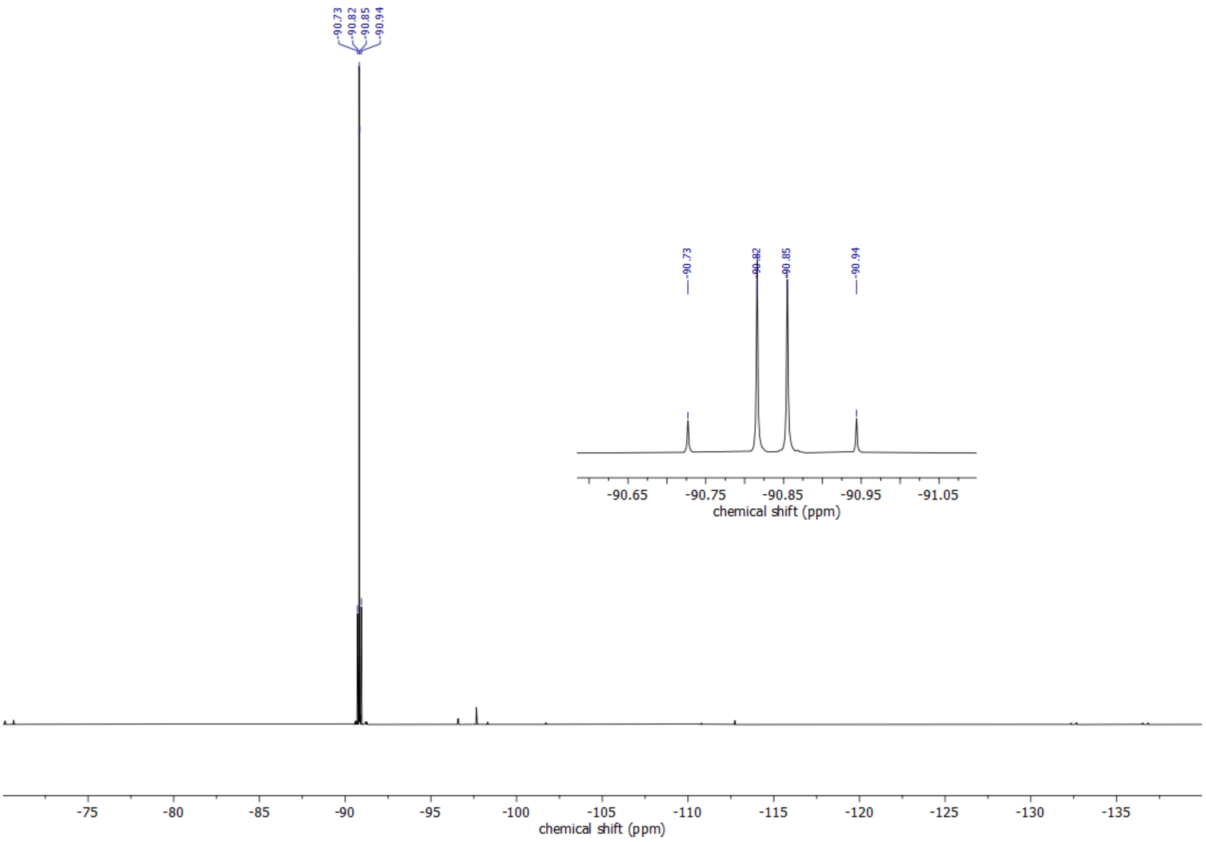

^{1}H NMR (500 MHz, $CDCl_3$)

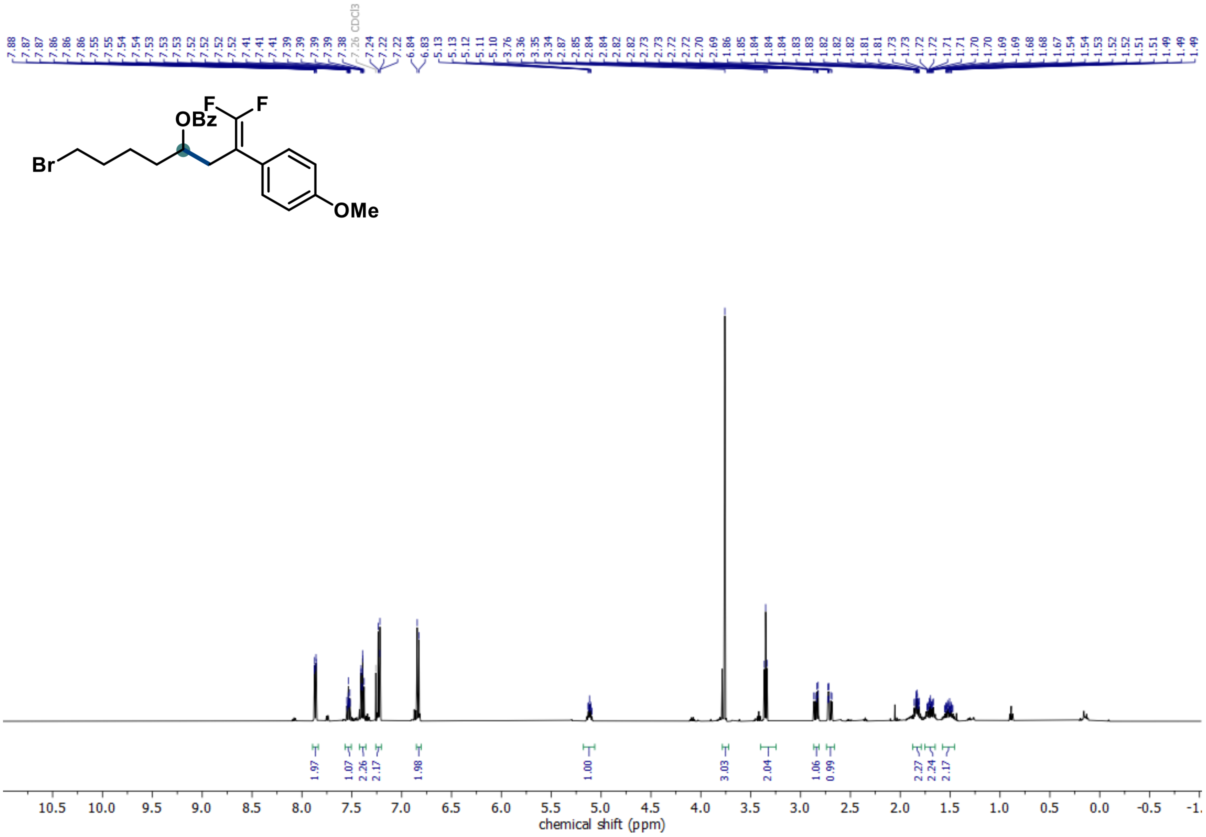

^{13}C{^{1}H} NMR (126 MHz, $CDCl_3$)

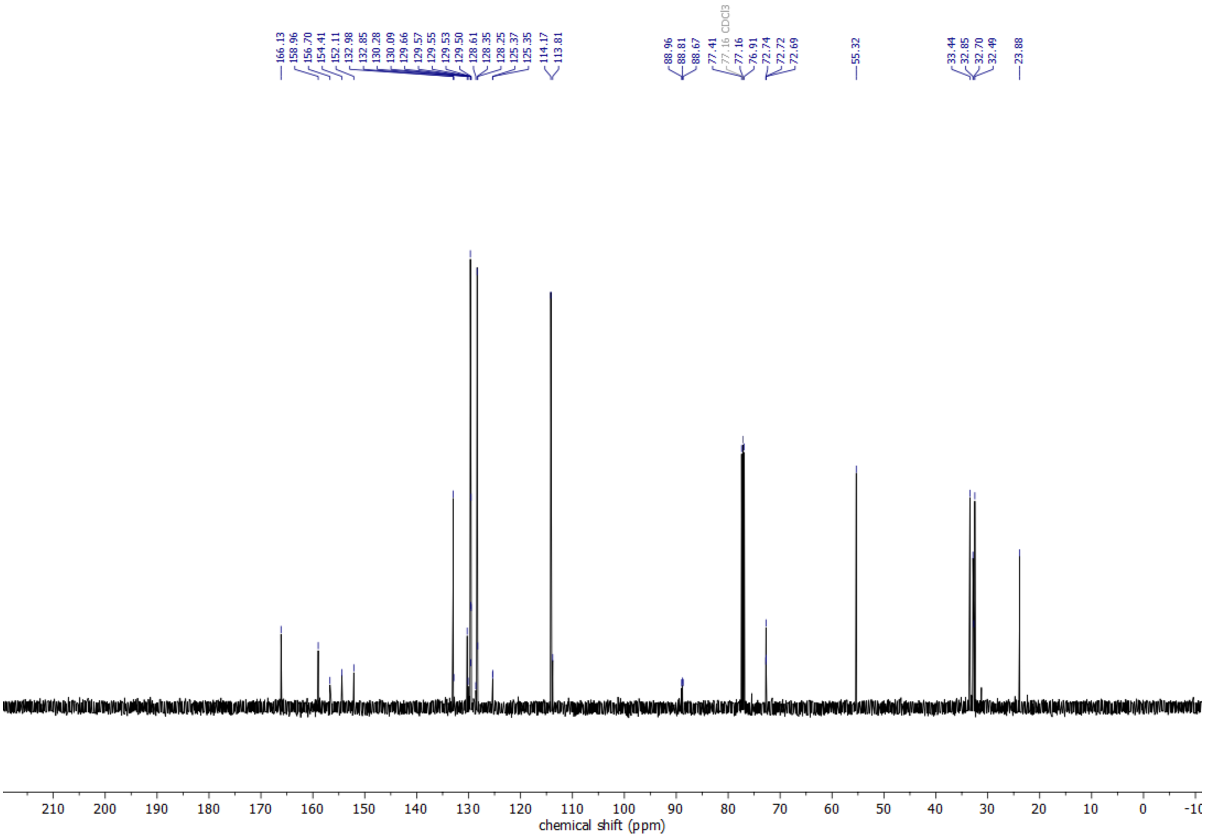

^{19}F NMR (470 MHz, $CDCl_3$)

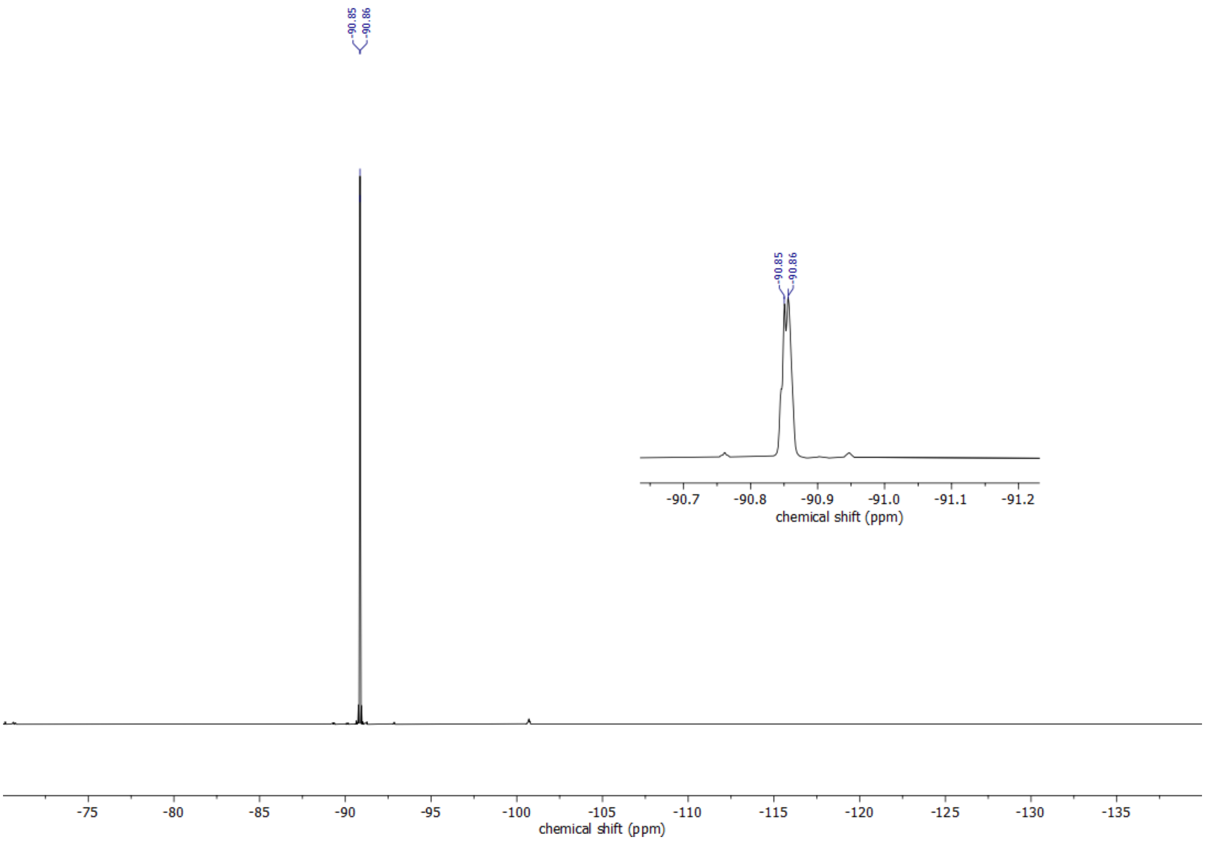

^1H NMR (500 MHz, $CDCl_3$)

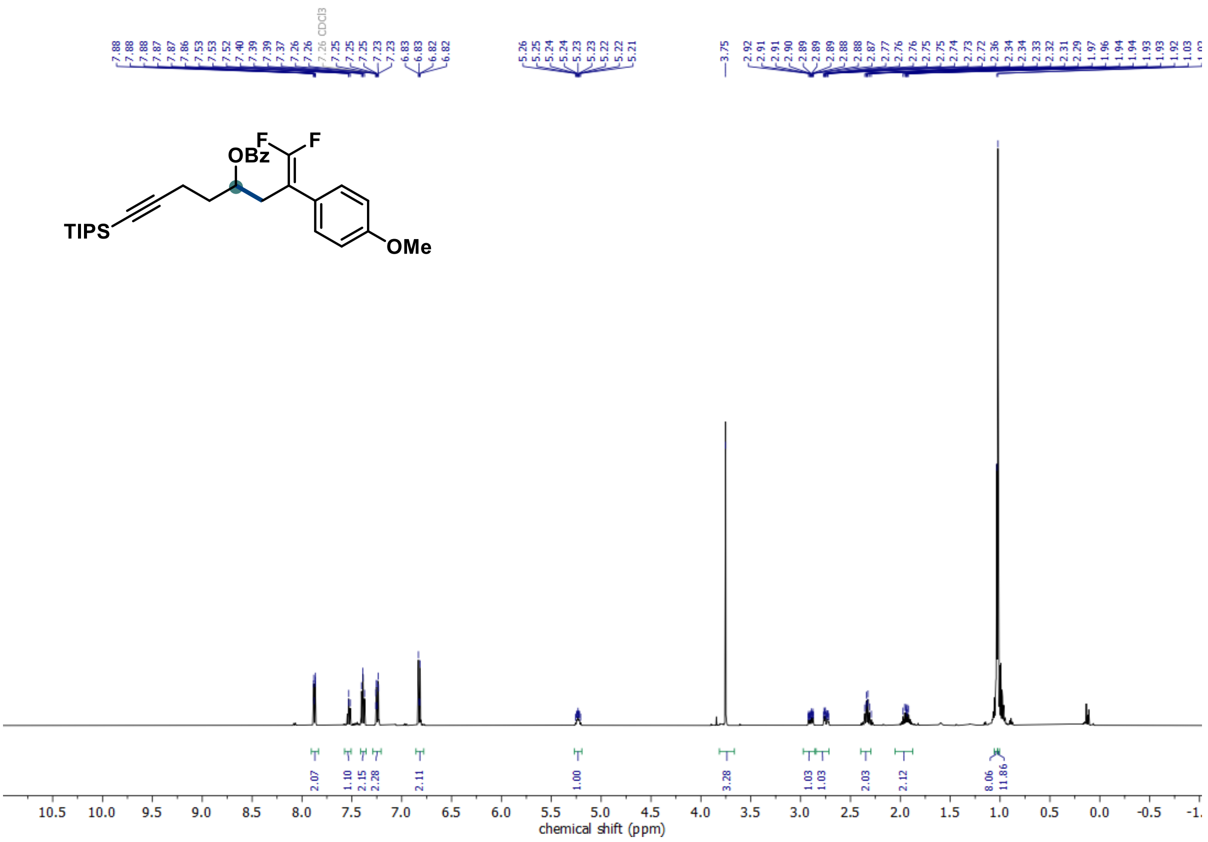

$^{13}C\{^1H\}$ NMR (126 MHz, $CDCl_3$)

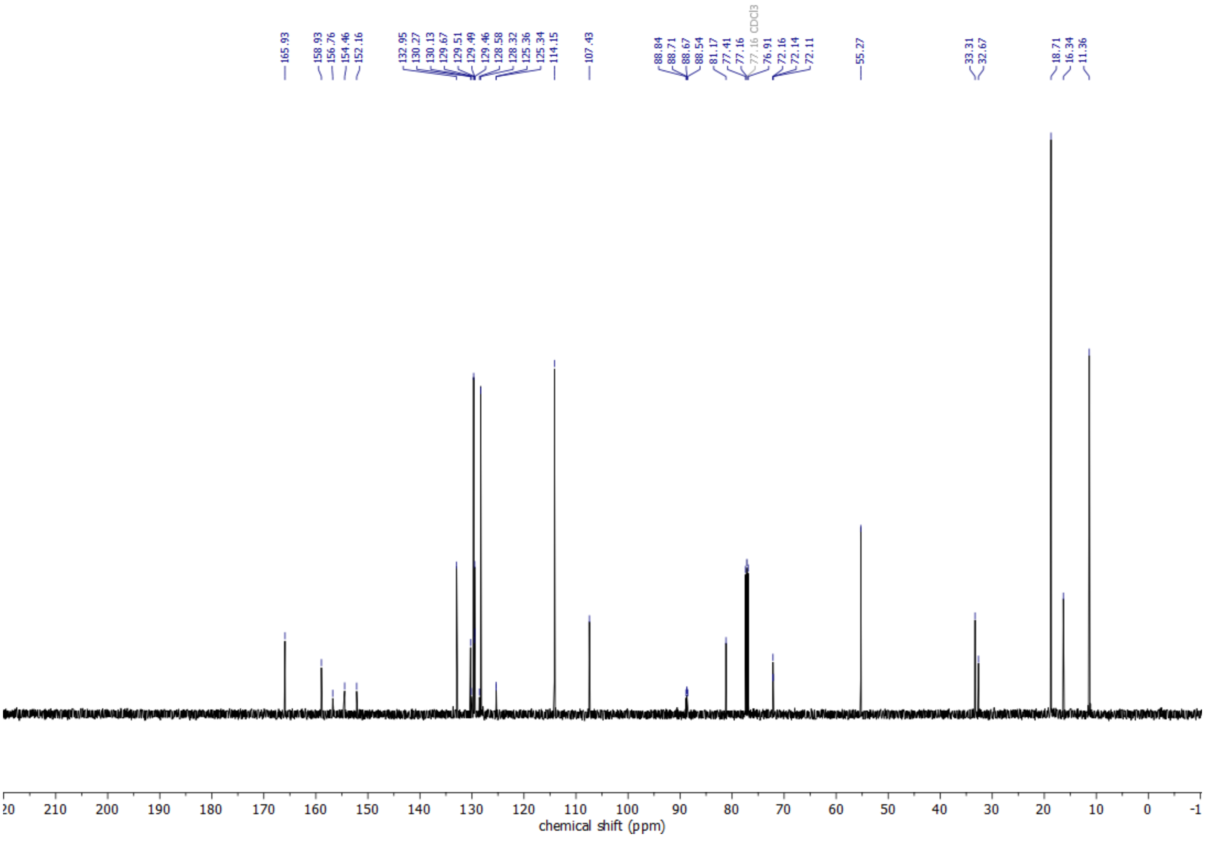

^{19}F NMR (470 MHz, $CDCl_3$)

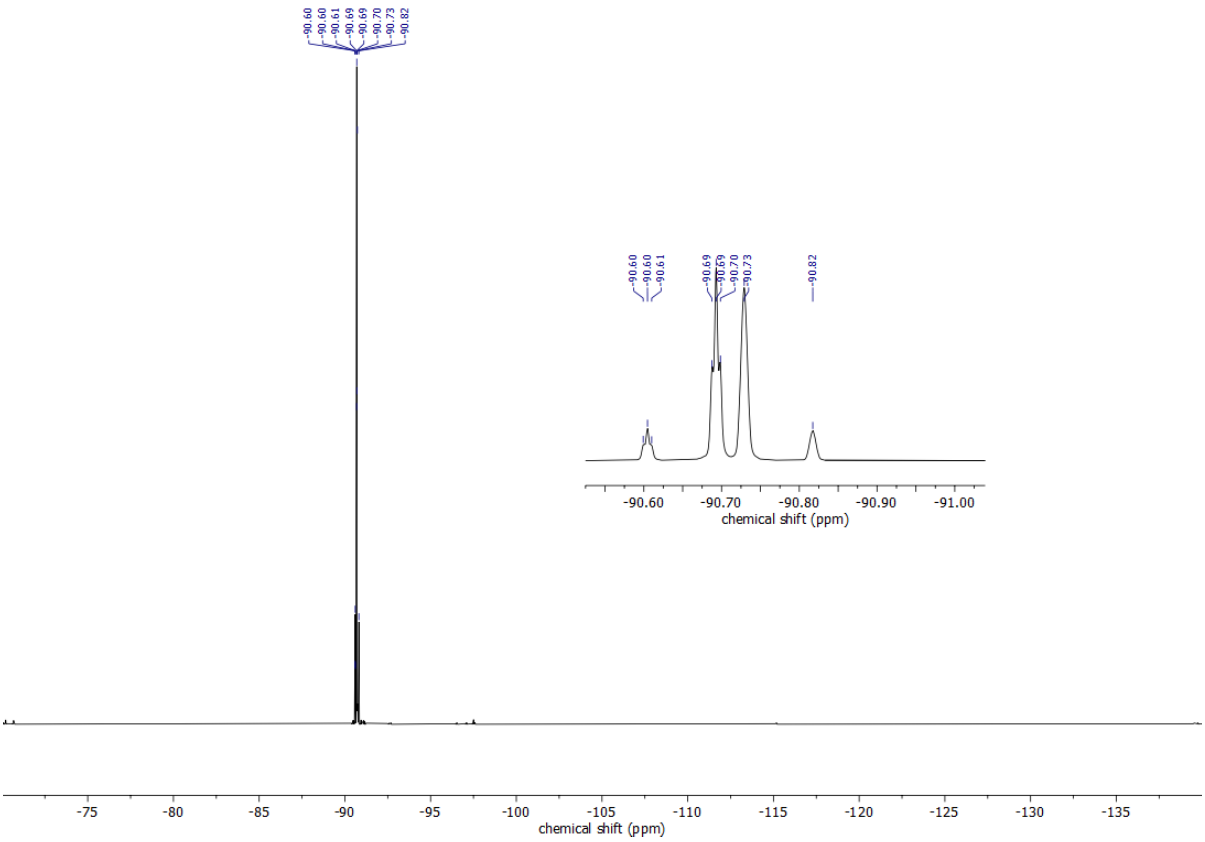

^{1}H NMR (500 MHz, $CDCl_3$)

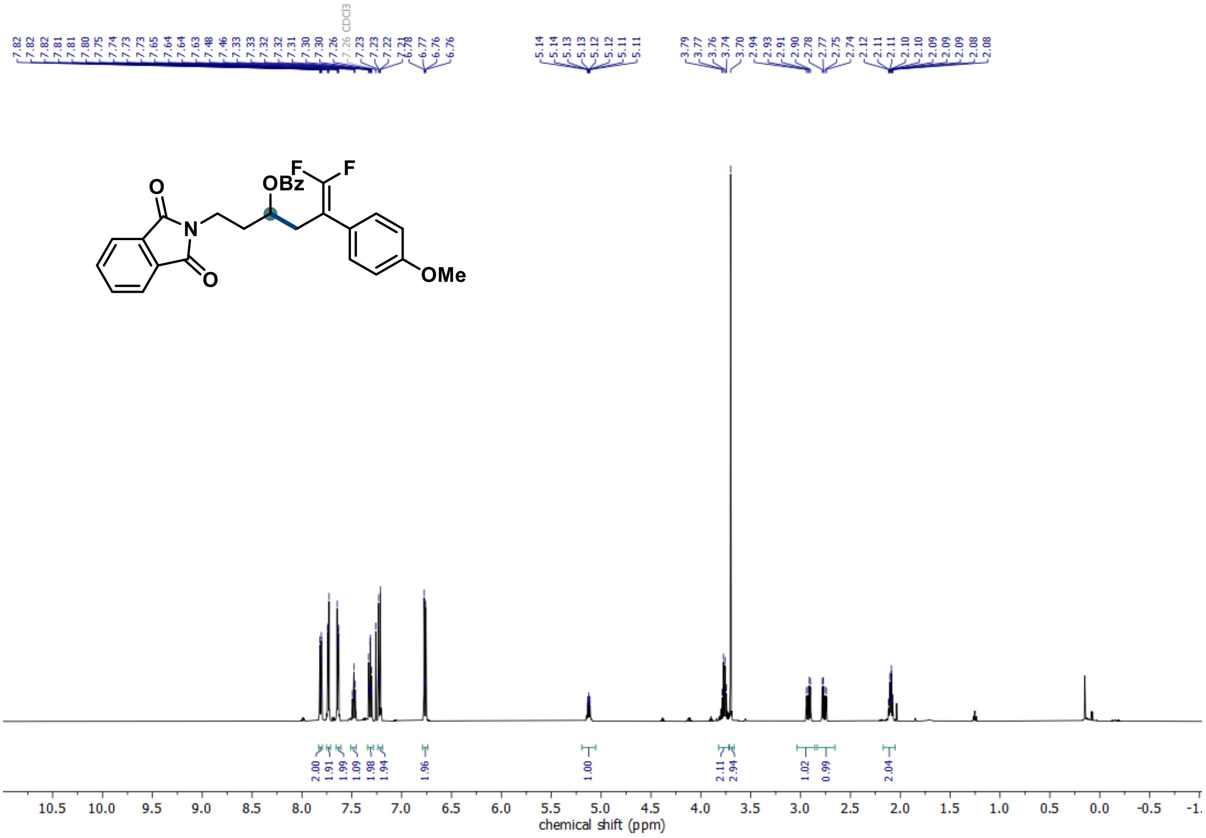

$^{13}C\{^{1}H\}$ NMR (126 MHz, $CDCl_3$)

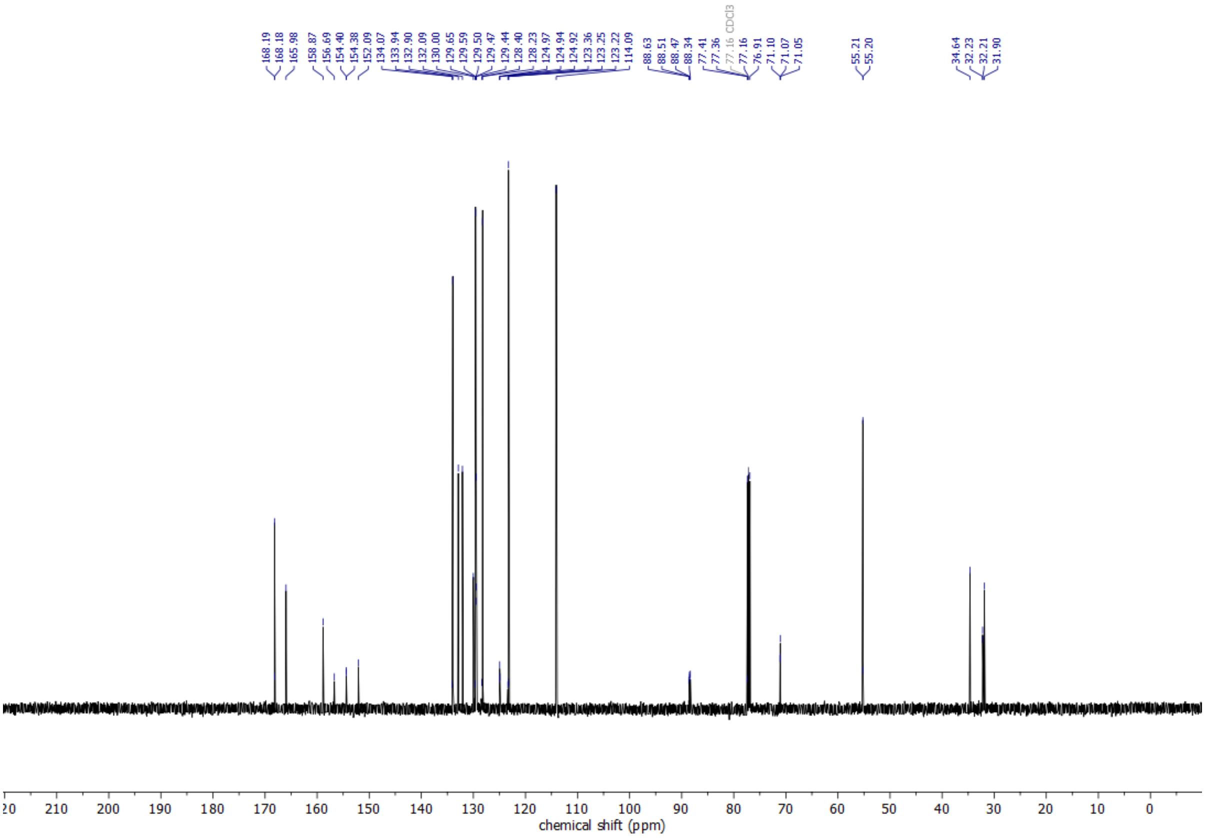

^{19}F NMR (470 MHz, CDCl$_3$)

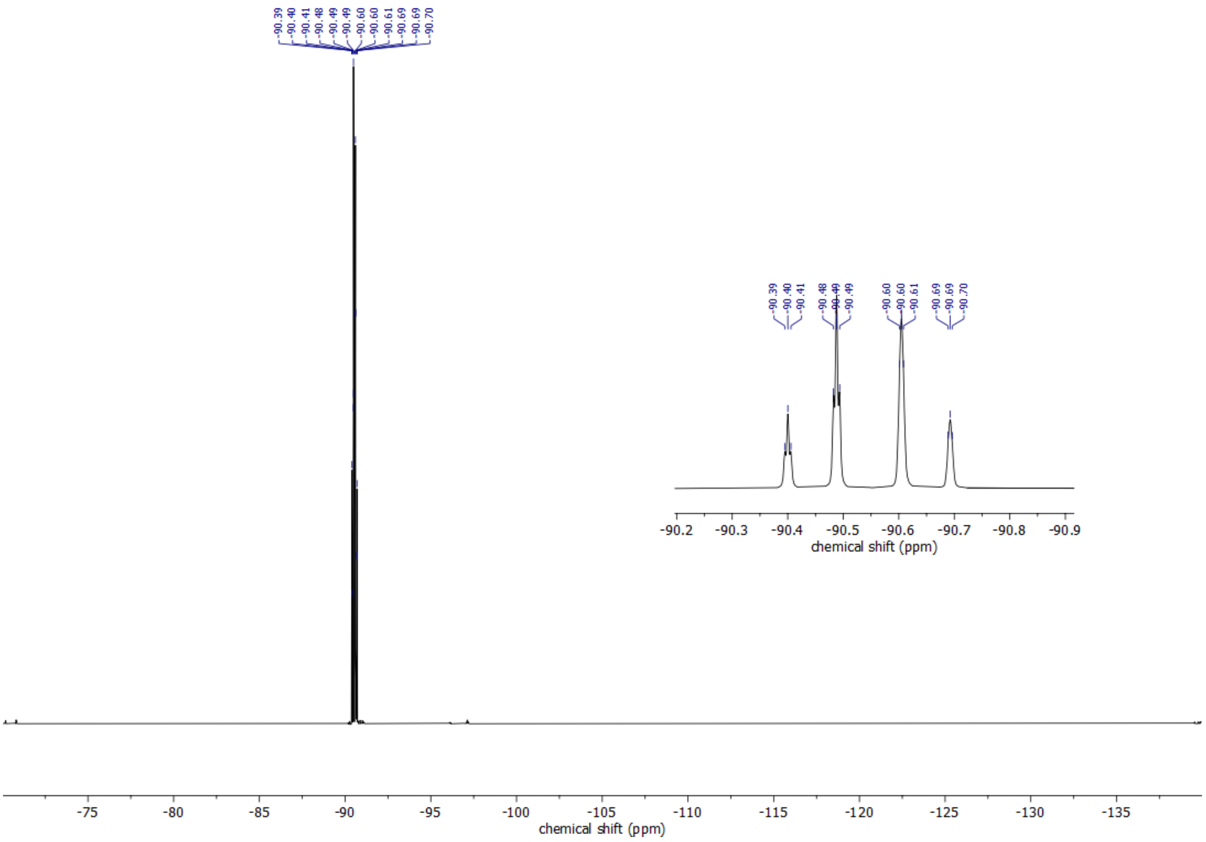

^{1}H NMR (400 MHz, $CDCl_3$)

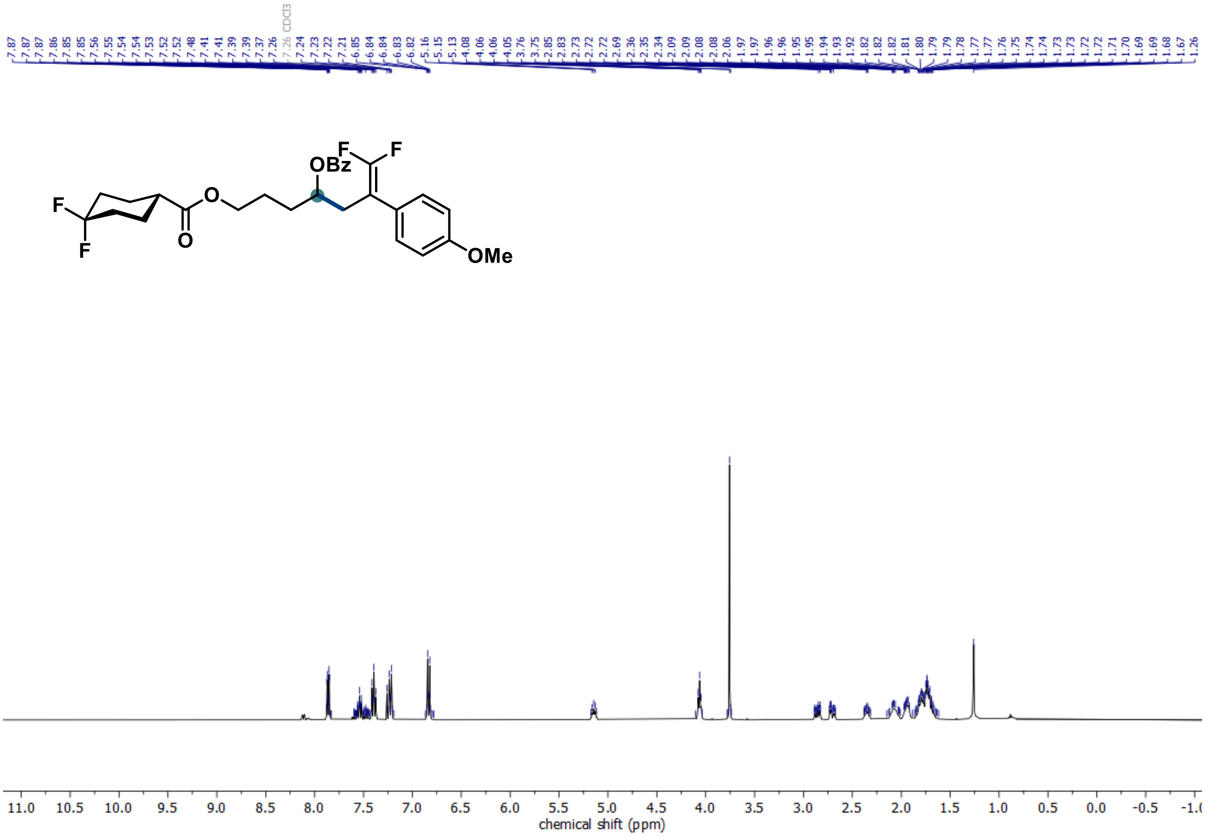

$^{13}C\{^{1}H\}$ NMR (151 MHz, $CDCl_3$)

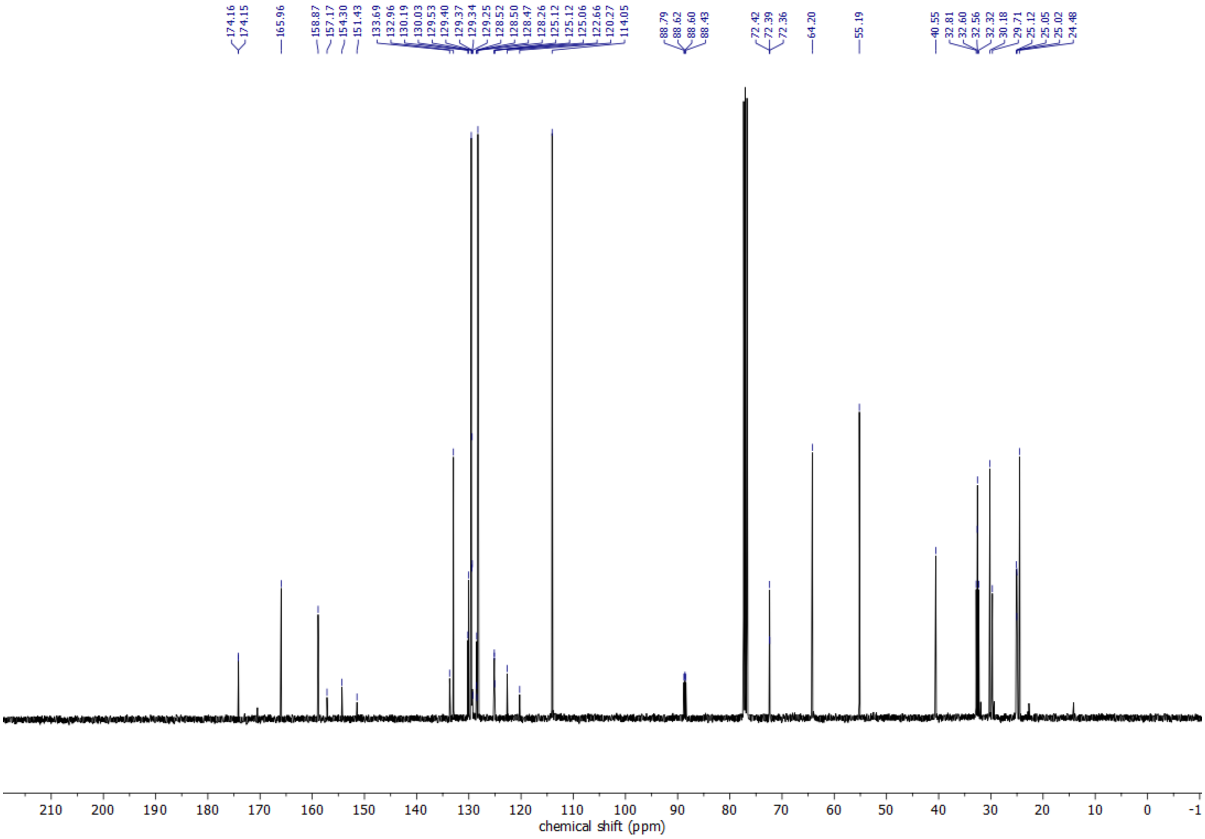

^{19}F NMR (377 MHz, CDCl$_3$)

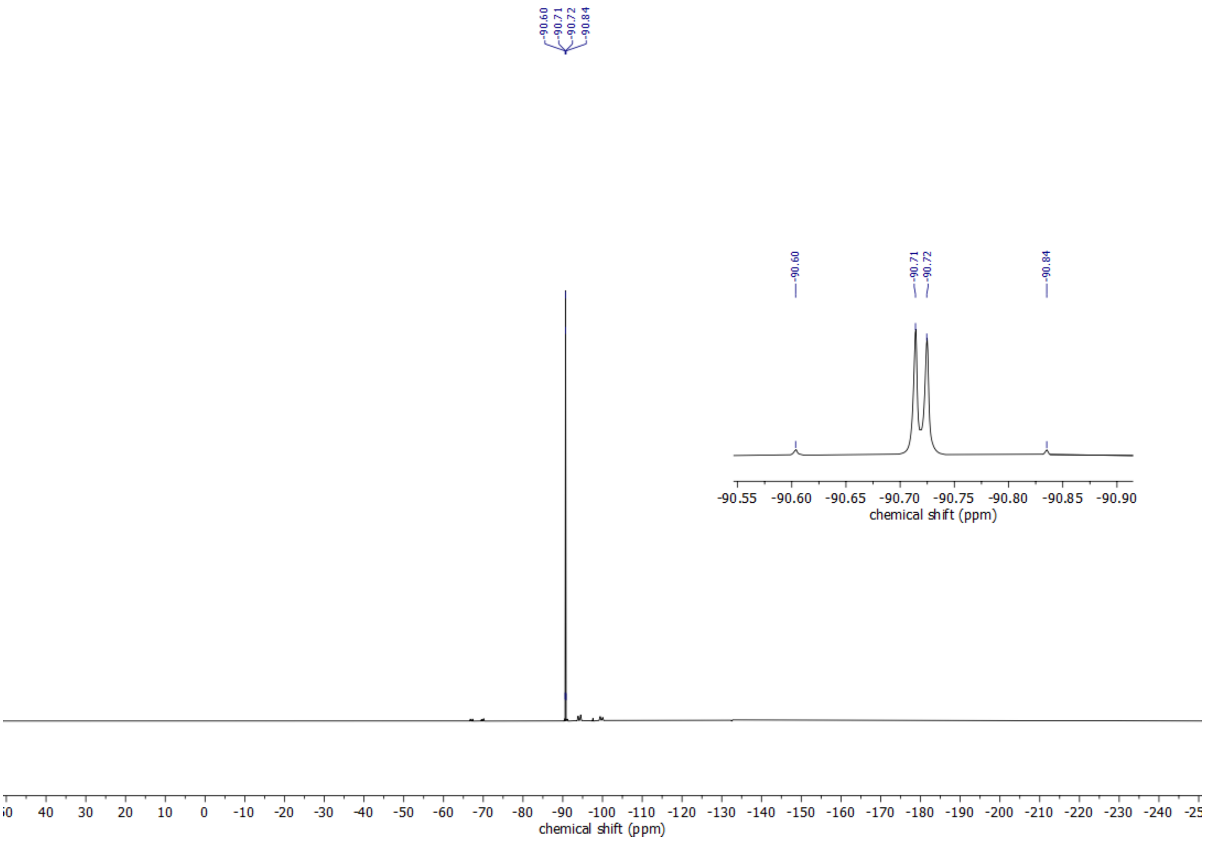

^{1}H NMR (500 MHz, $CDCl_3$)

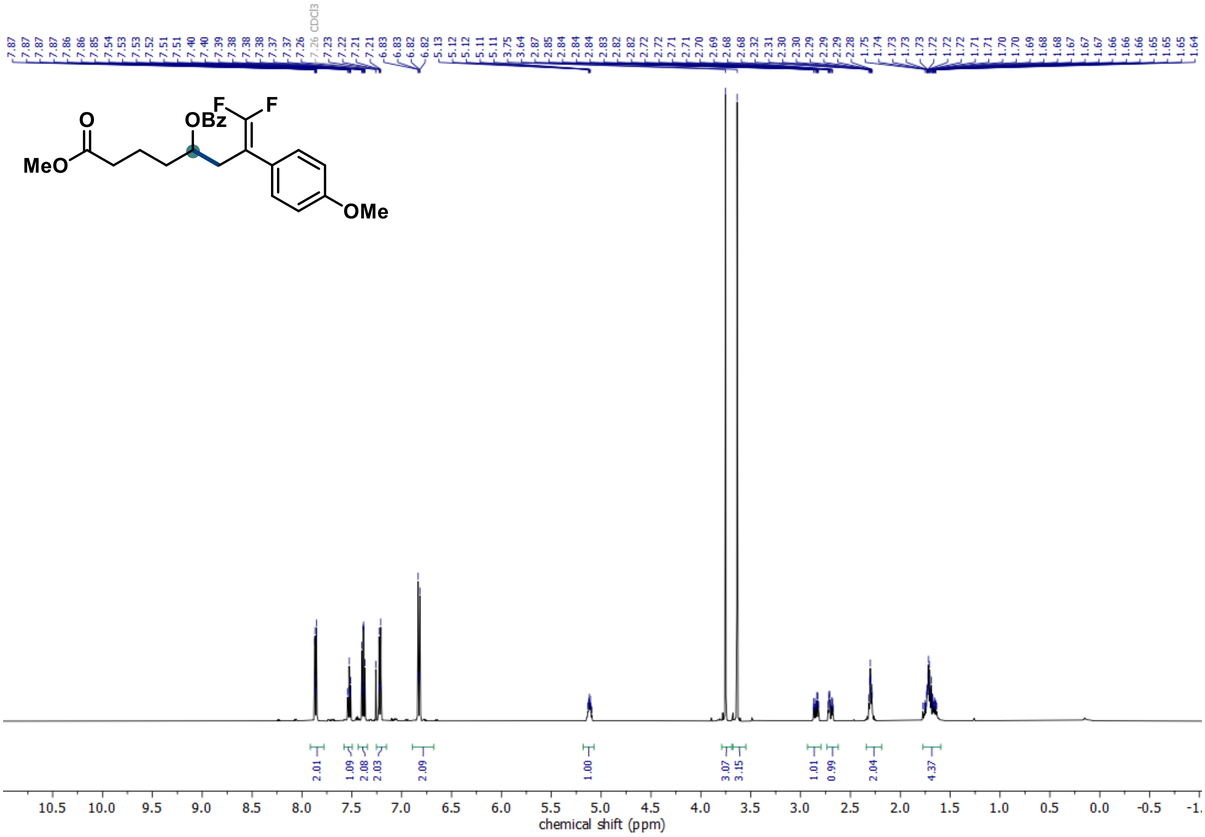

^{13}C{^{1}H} NMR (126 MHz, $CDCl_3$)

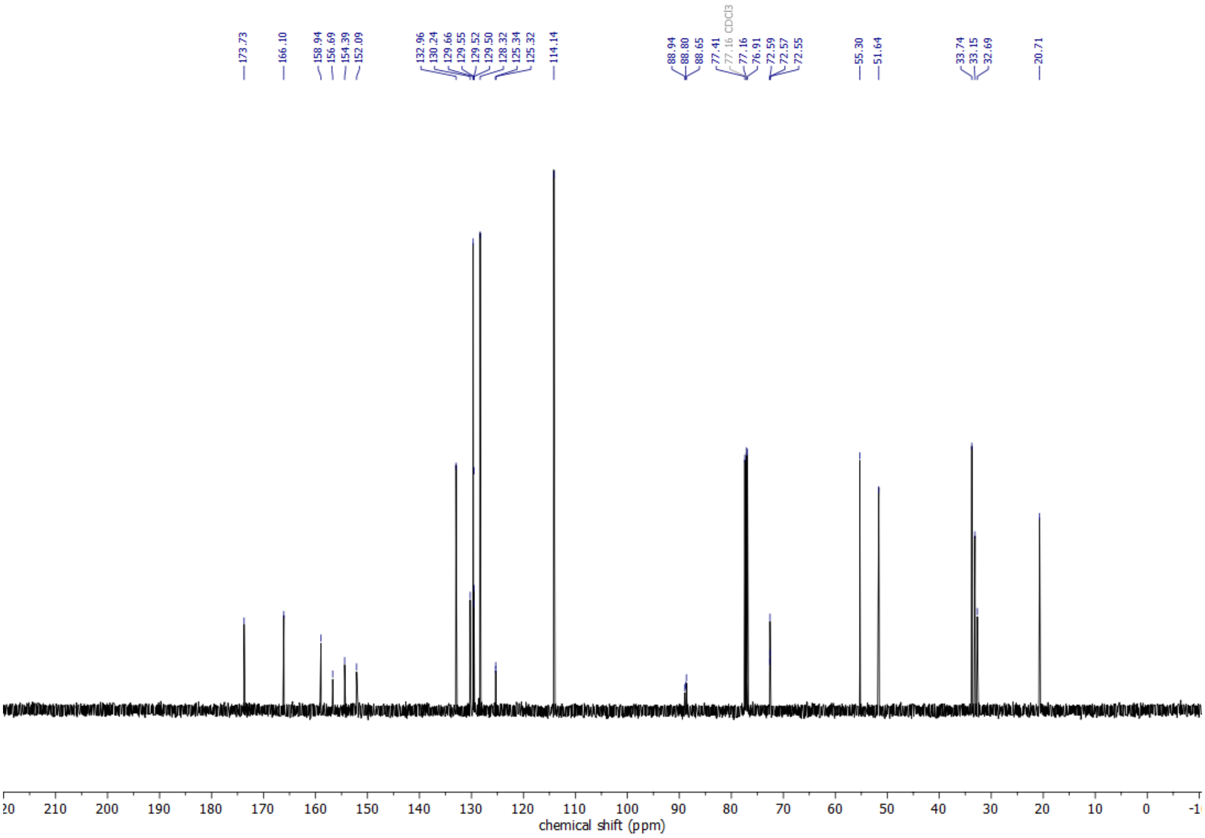

^{19}F NMR (470 MHz, $CDCl_3$)

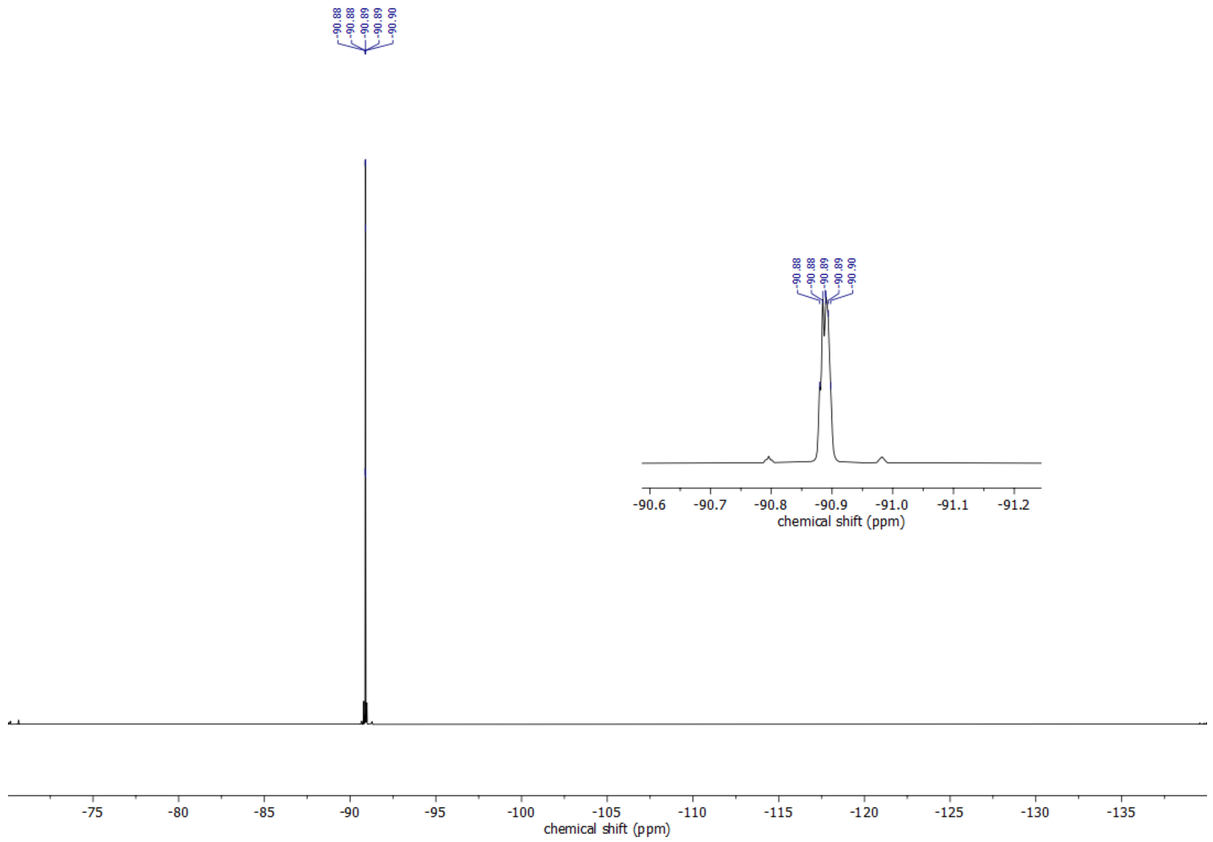

^{1}H NMR (599 MHz, $CDCl_3$)

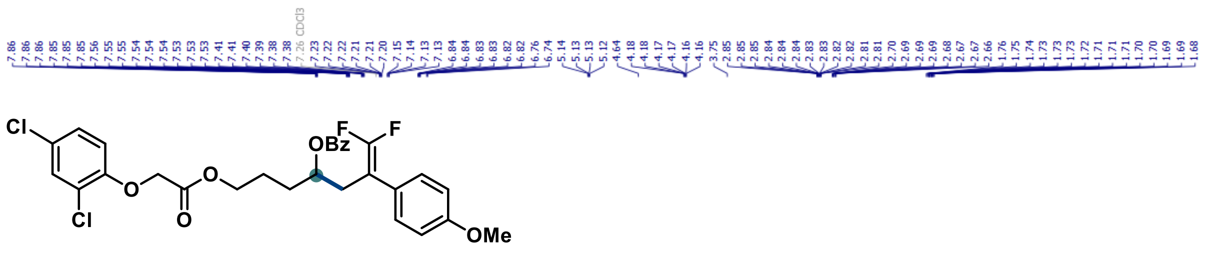

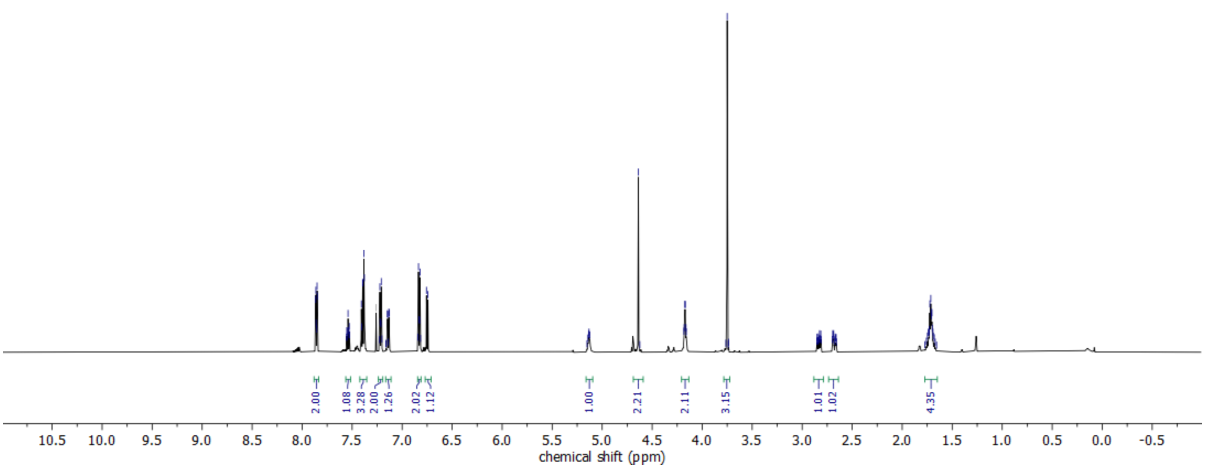

$^{13}C\{^{1}H\}$ NMR (151 MHz, $CDCl_3$)

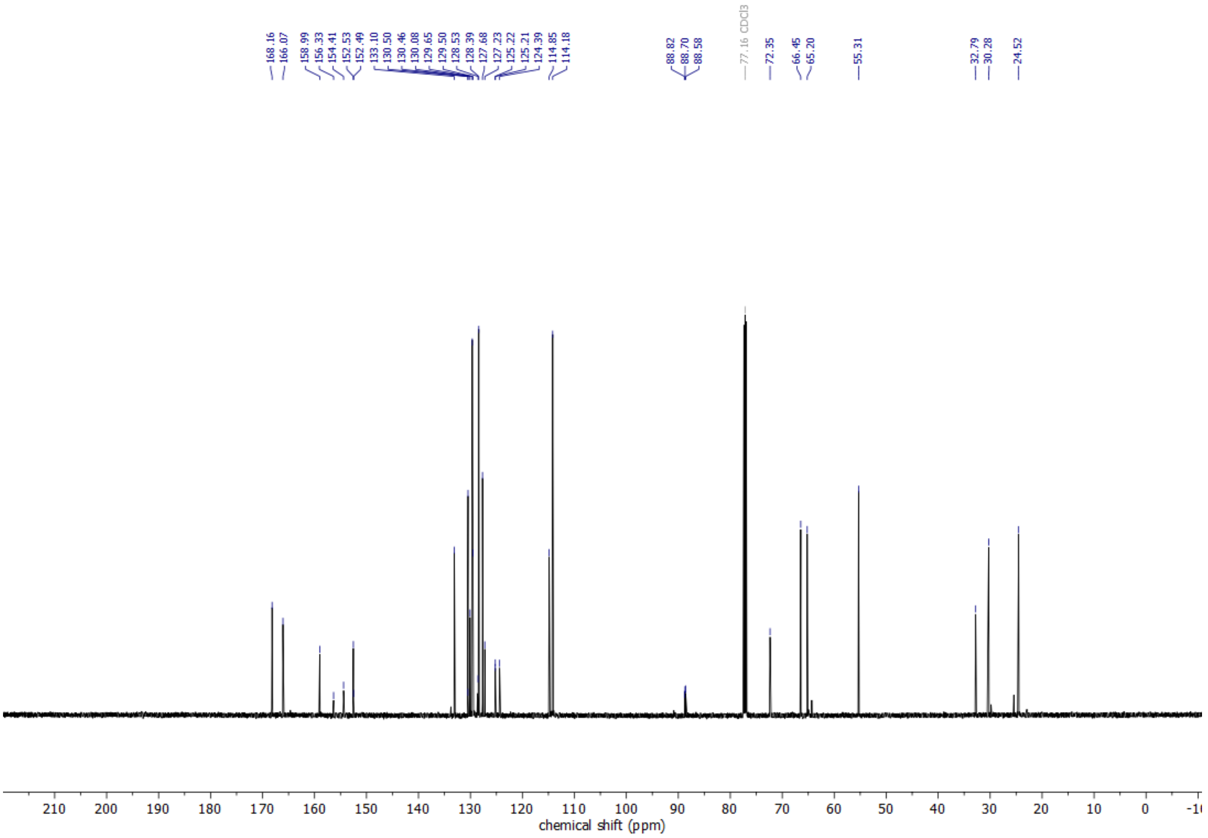

^{19}F NMR (564 MHz, $CDCl_3$)

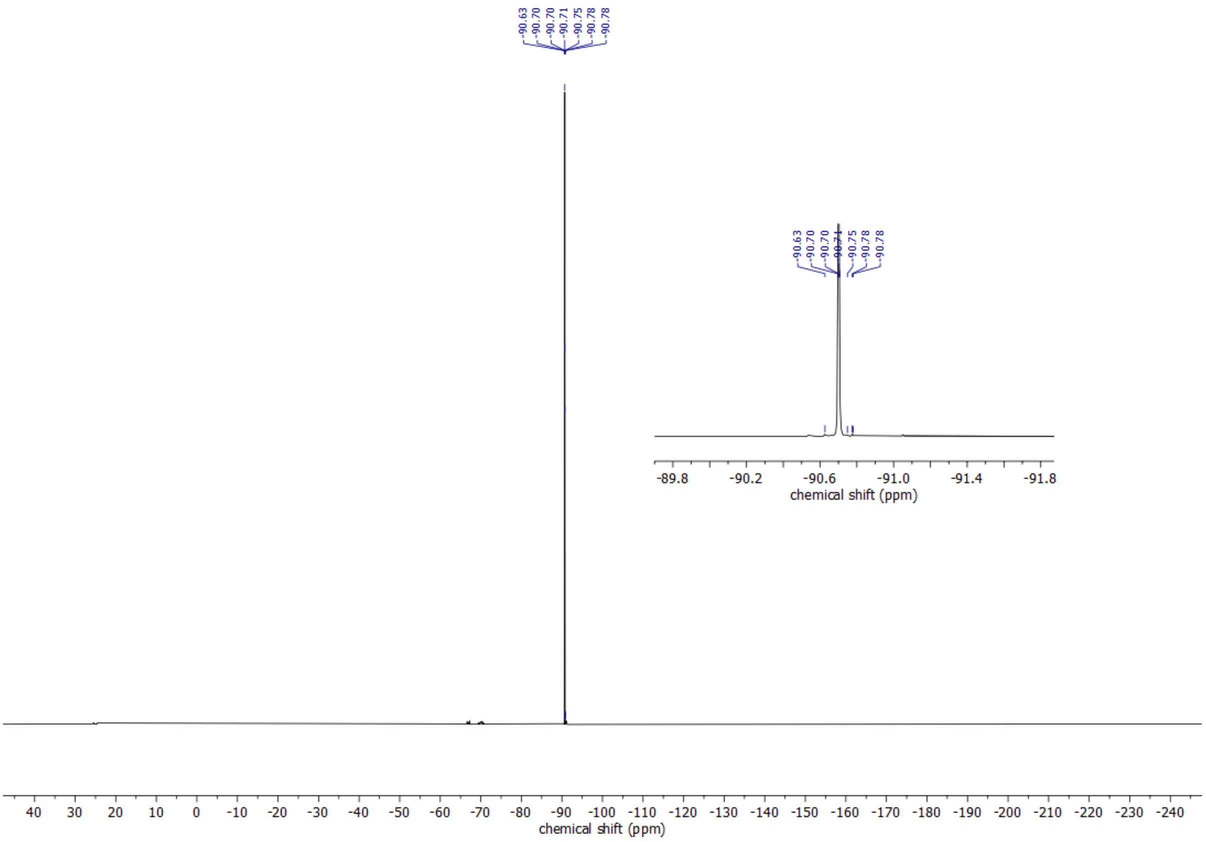

^1H-^1H COSY NMR (599 MHz, $CDCl_3$)

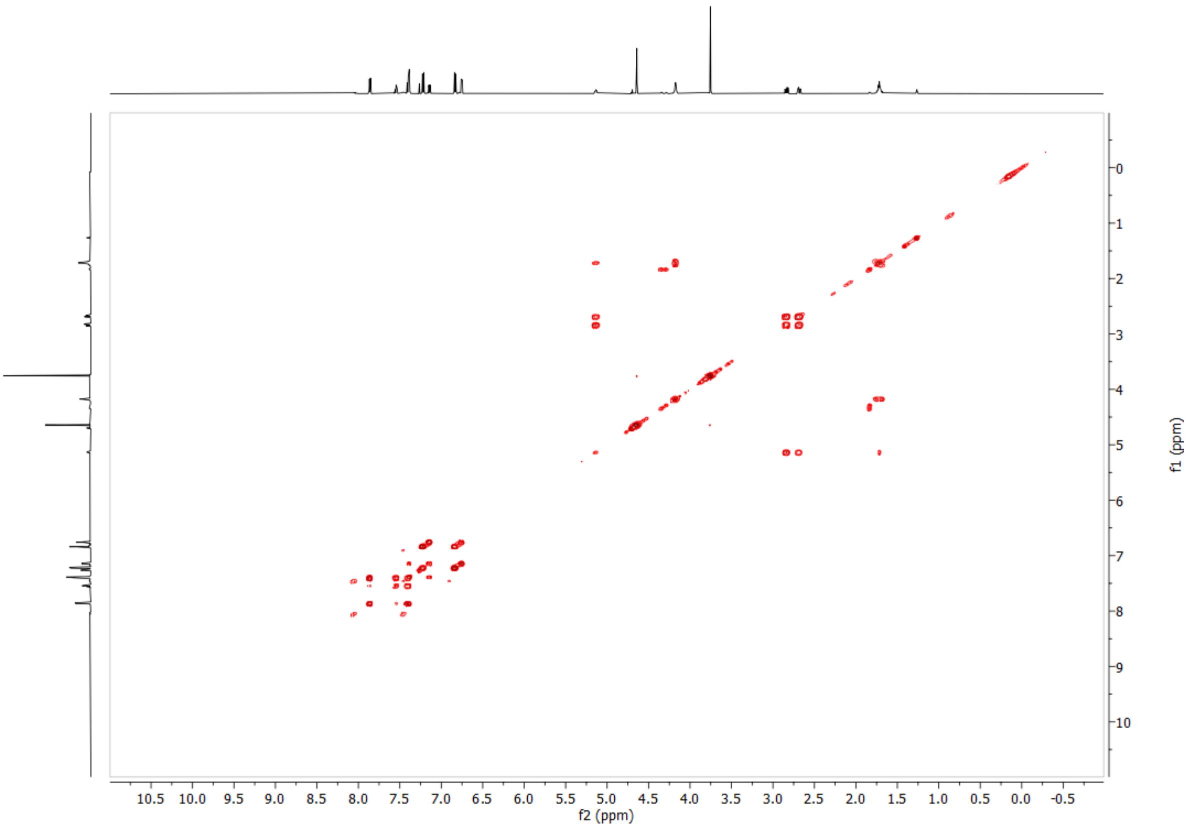

^{1}H-^{13}C HSQC NMR (599 MHz, $CDCl_3$)

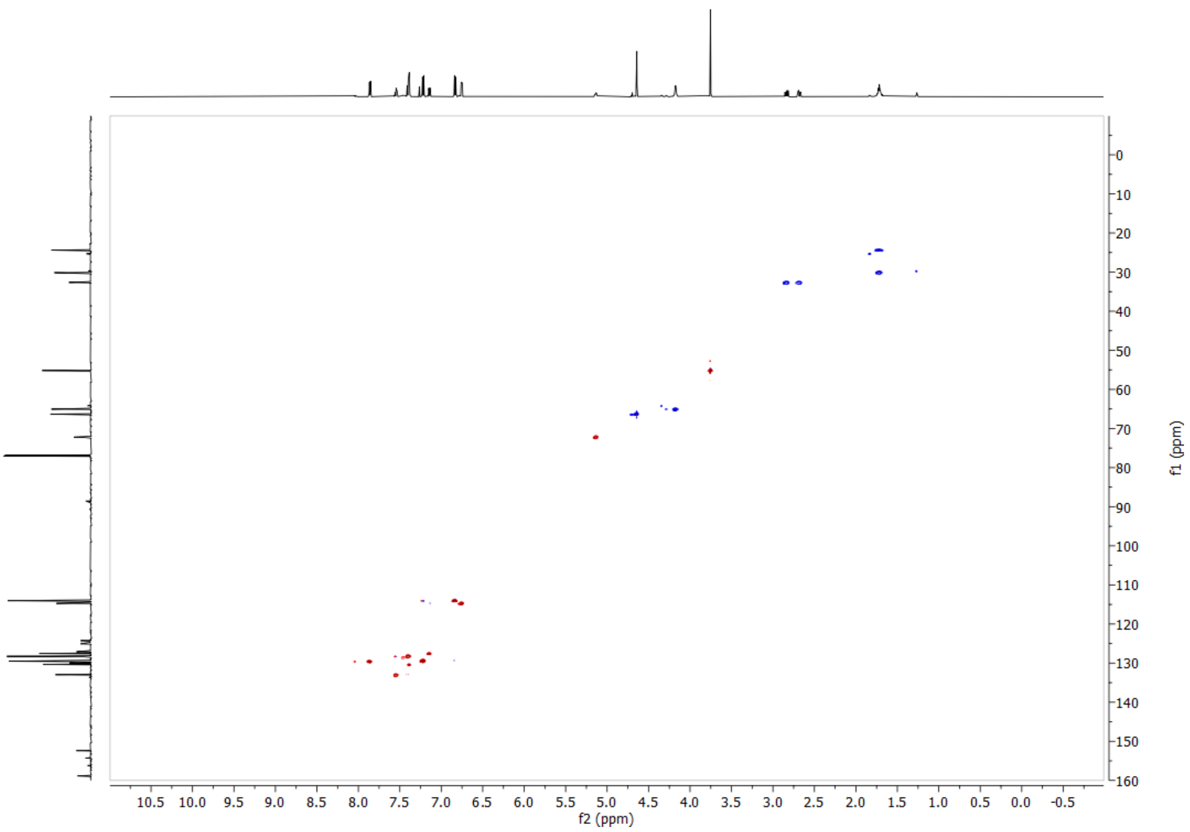

^{1}H-^{13}C HMBC NMR (599 MHz, $CDCl_3$)

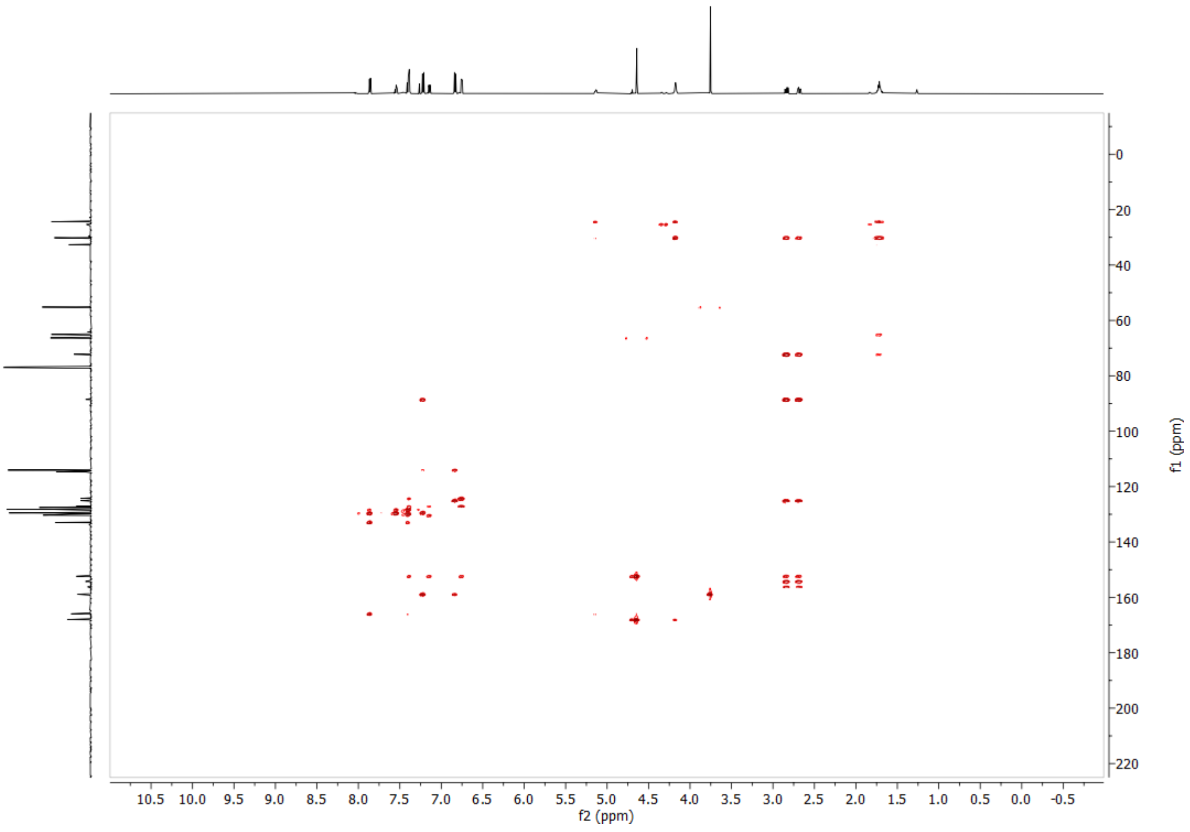

^{1}H NMR (599 MHz, $CDCl_3$)

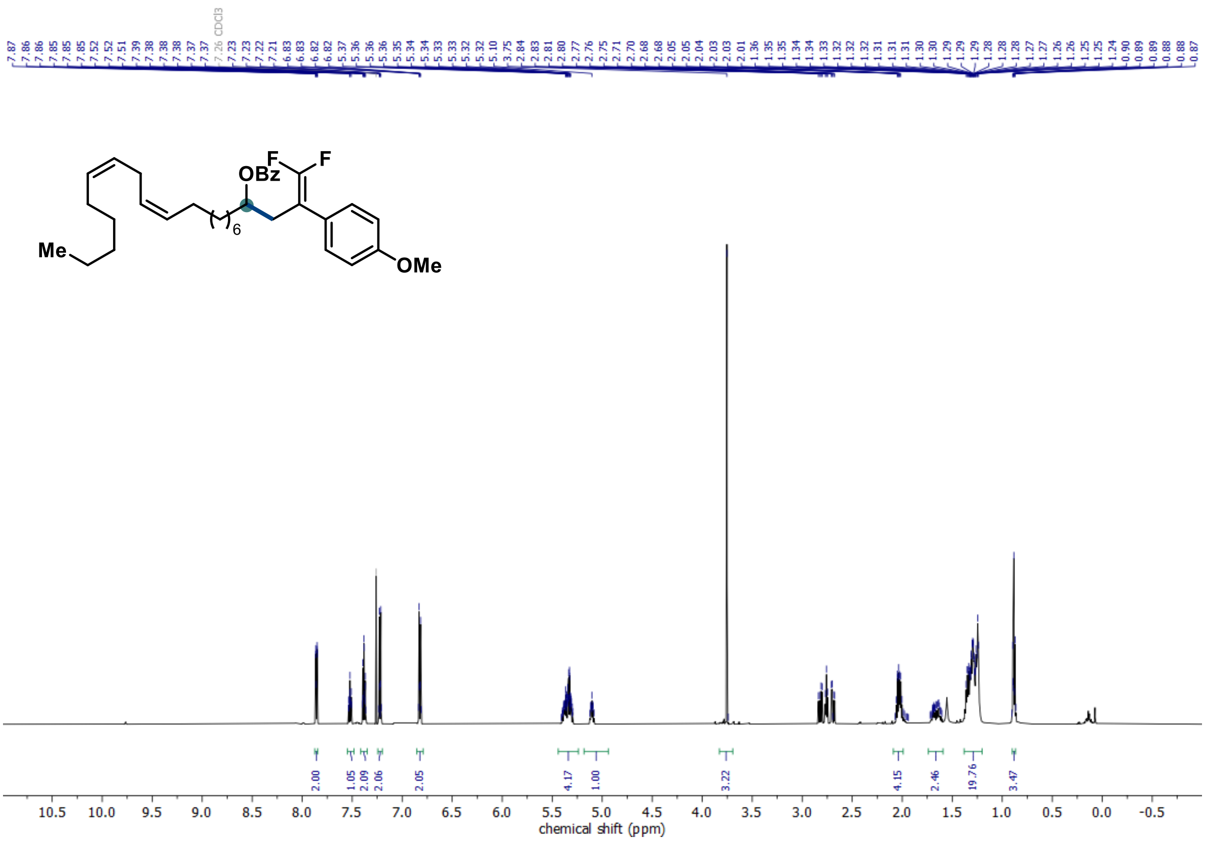

^{13}C{^{1}H, ^{19}F} NMR (151 MHz, $CDCl_3$)

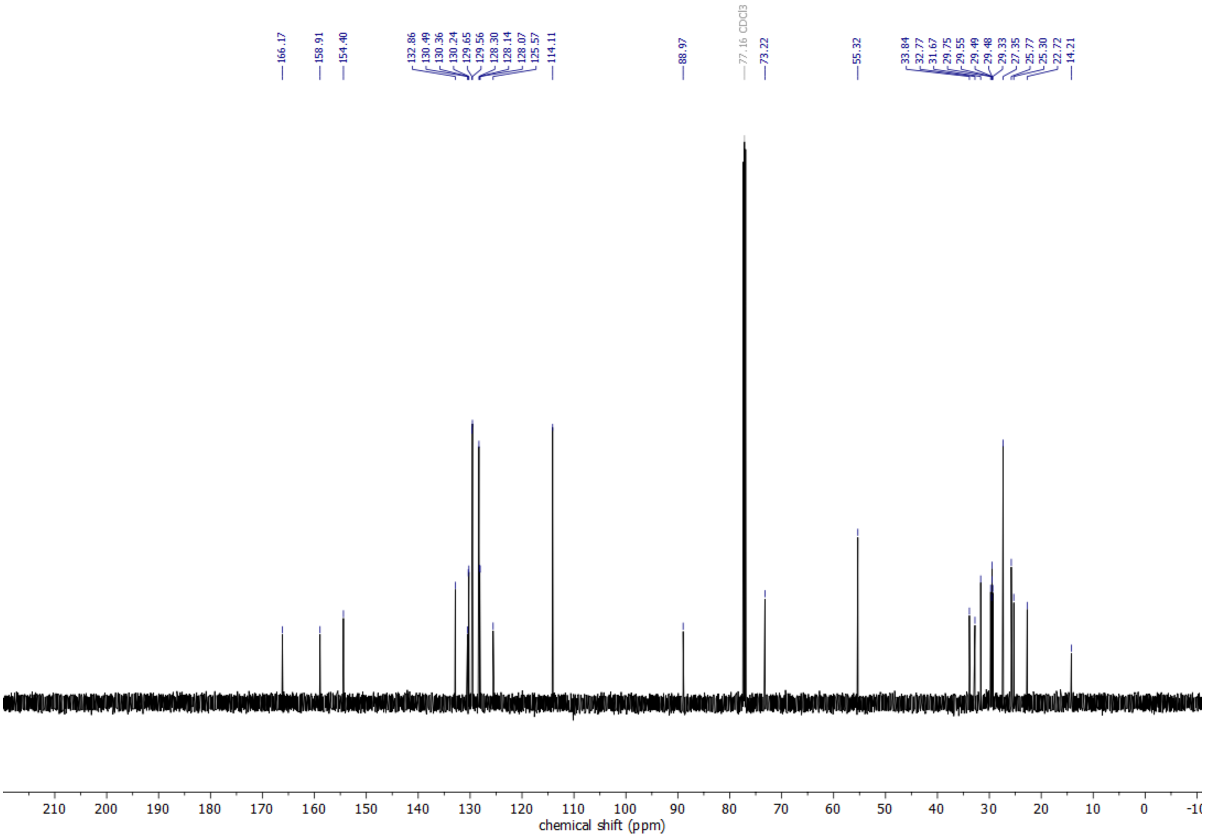

^{19}F NMR (564 MHz, CDCl$_3$)

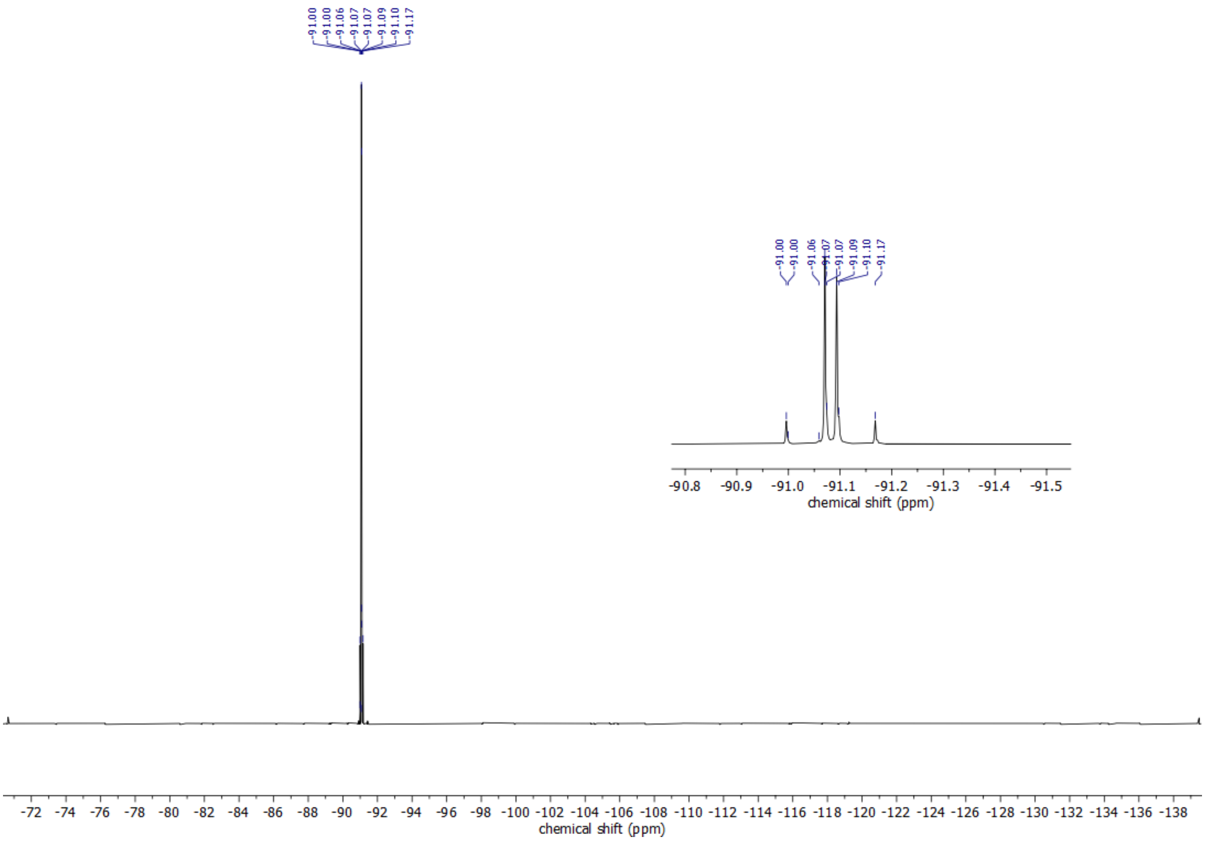

^{1}H NMR (500 MHz, $CDCl_3$)

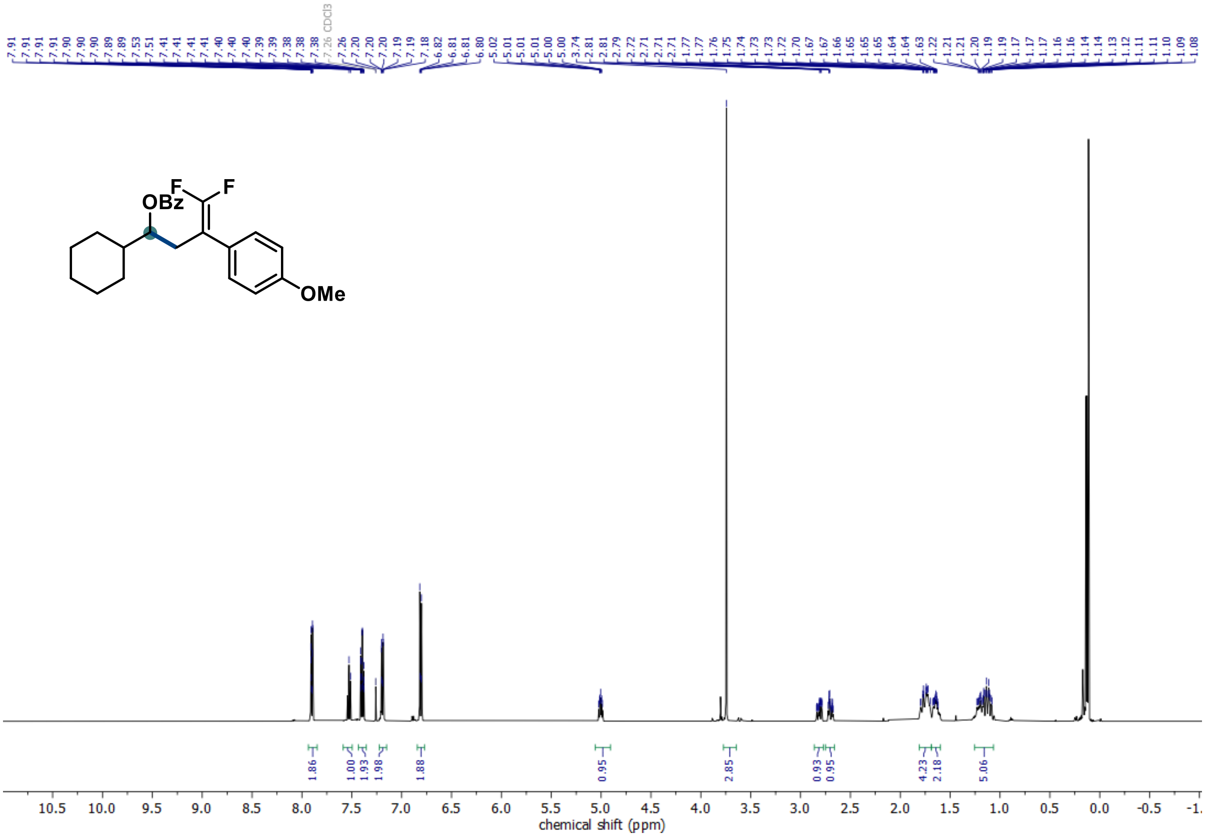

$^{13}C\{^{1}H\}$ NMR (126 MHz, $CDCl_3$)

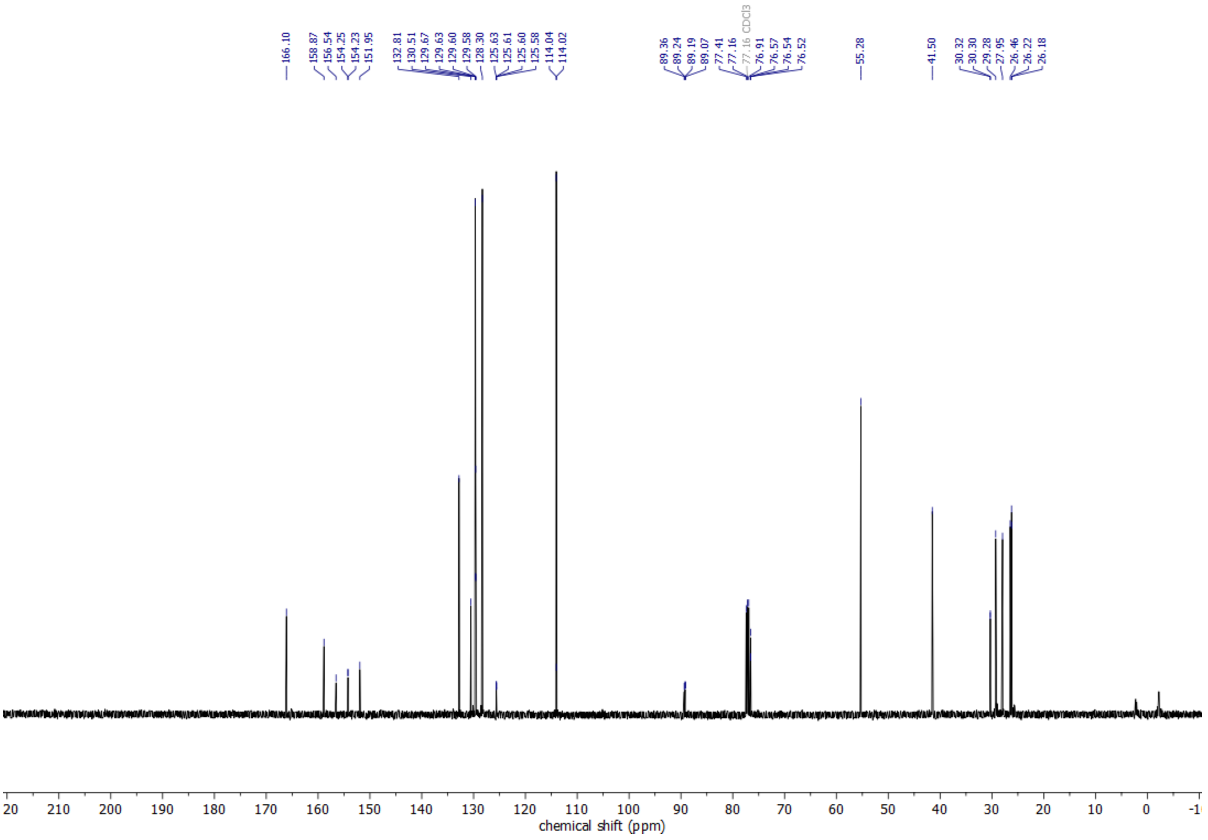

^{19}F NMR (470 MHz, CDCl$_3$)

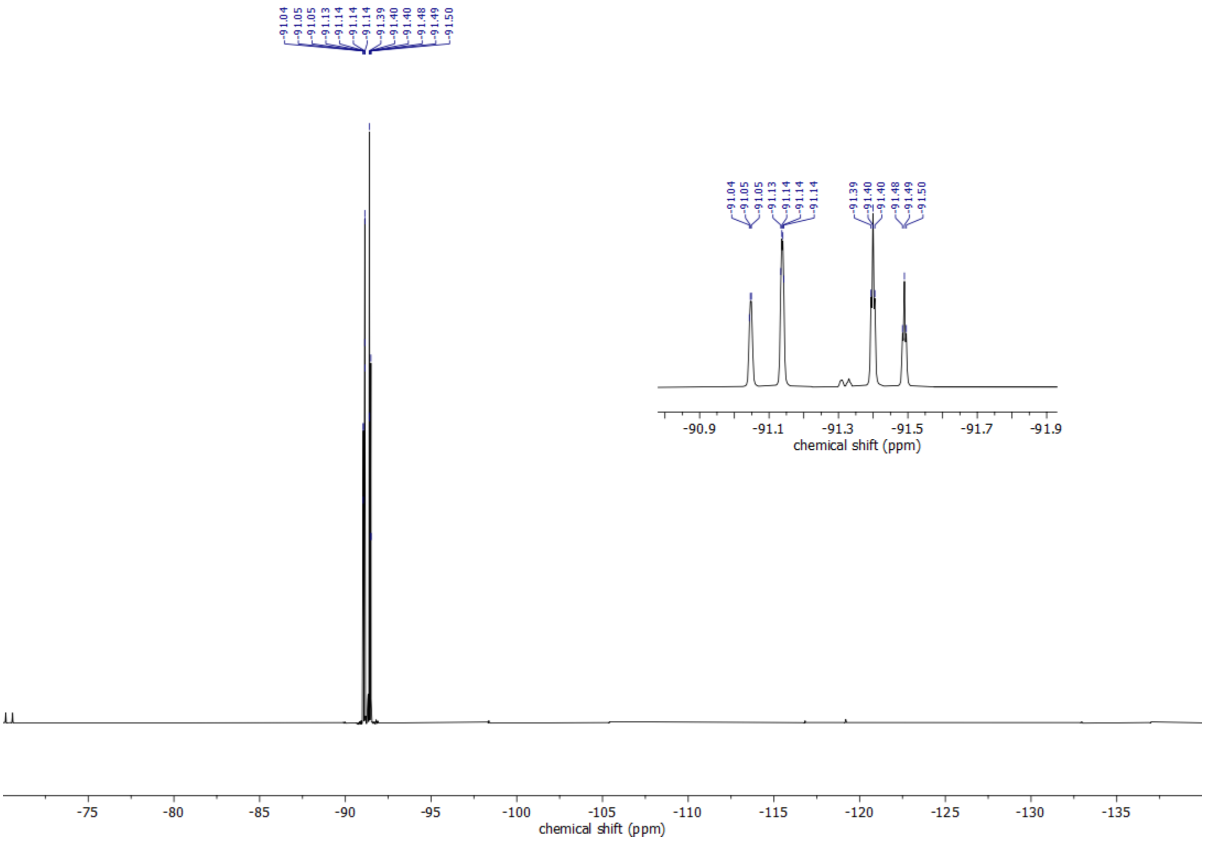

^{1}H NMR (599 MHz, $CDCl_3$)

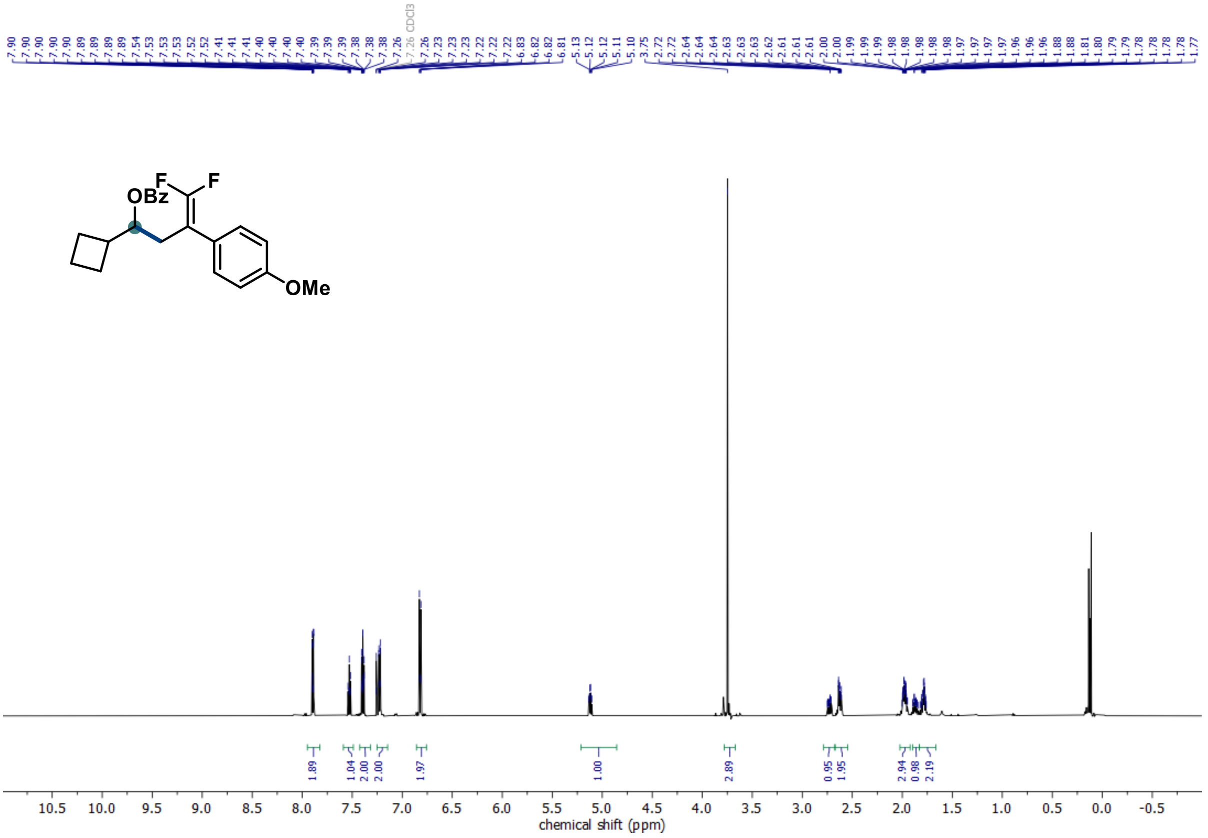

$^{13}C\{^1H\}$ NMR (151 MHz, $CDCl_3$)

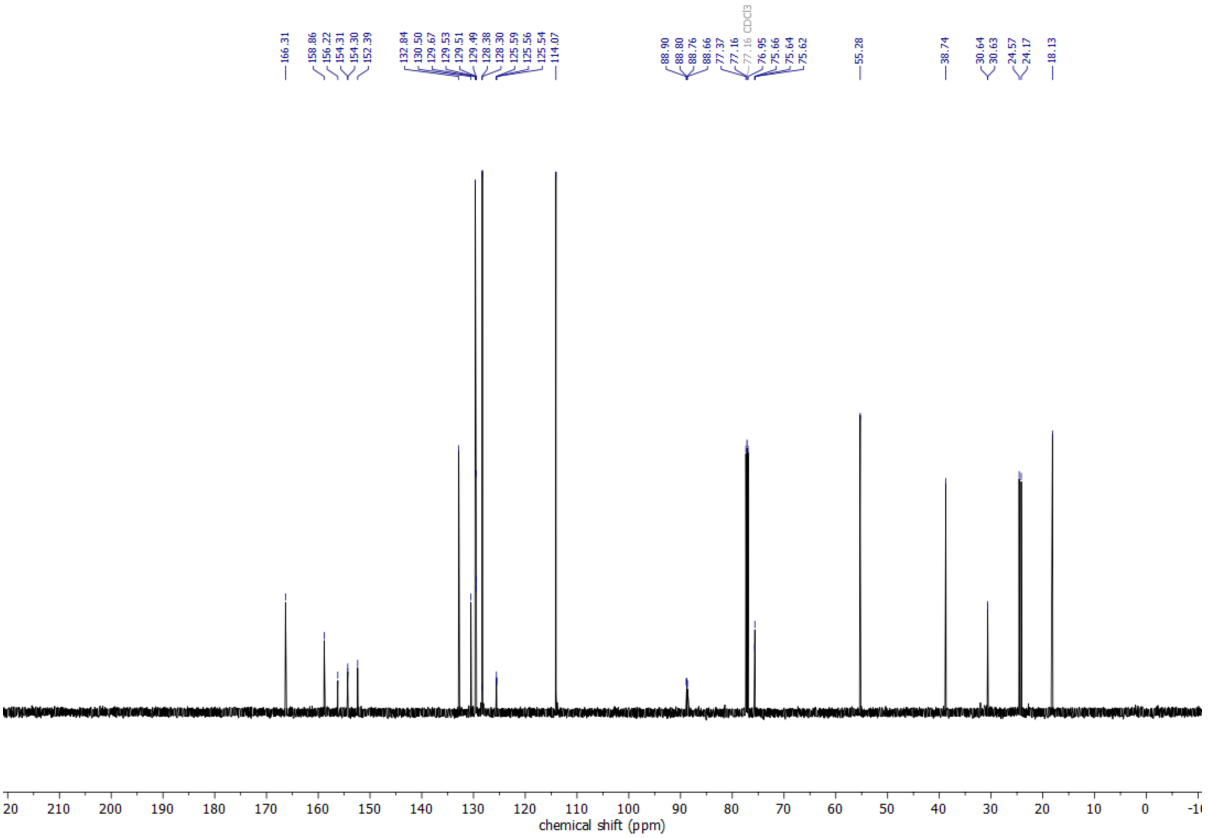

^{19}F NMR (564 MHz, $CDCl_3$)

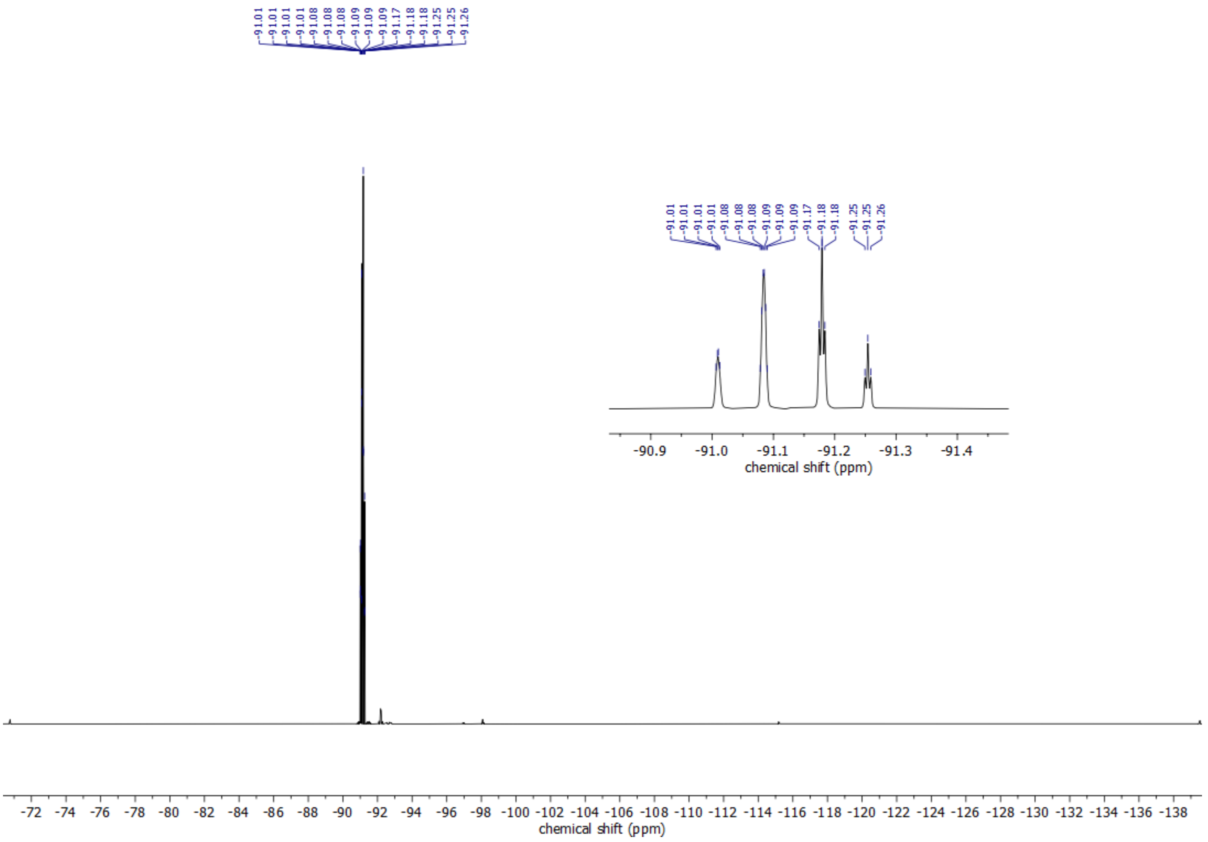

^{1}H NMR (599 MHz, $CDCl_3$)

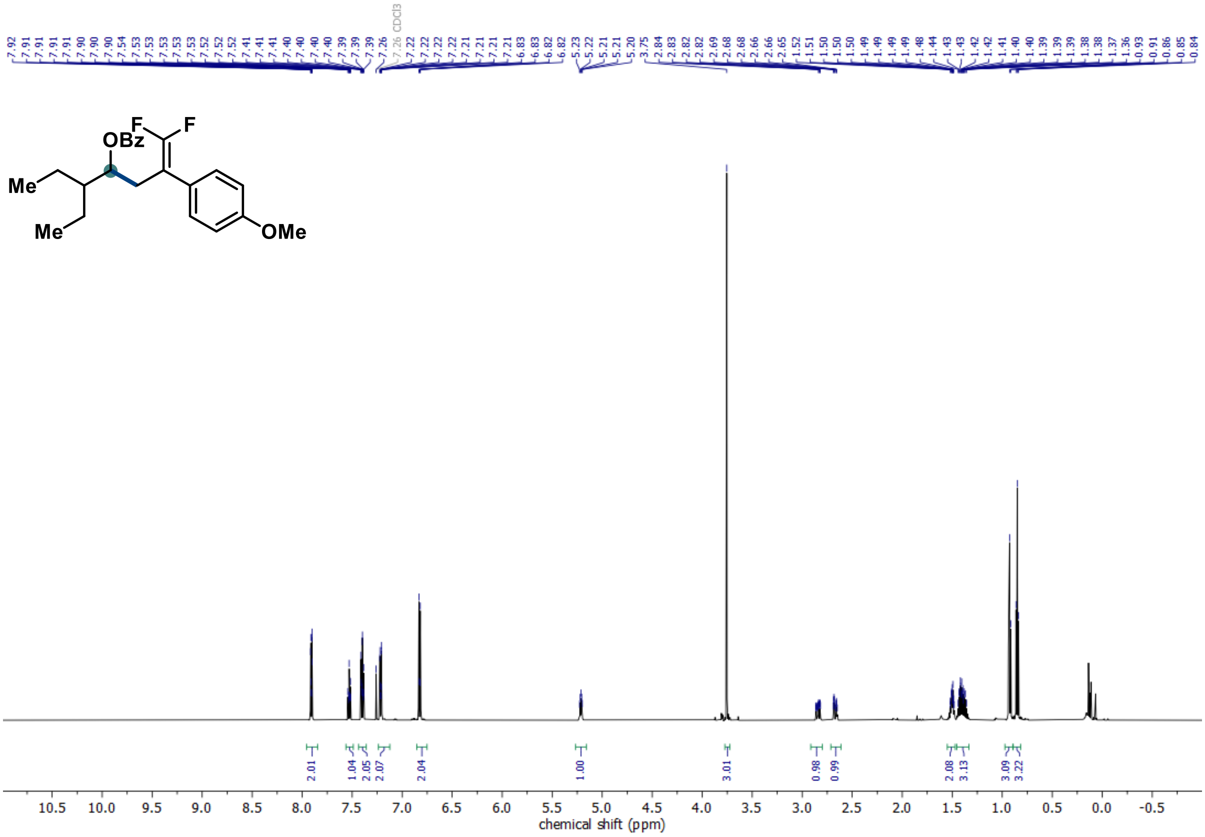

$^{13}C\{^{1}H\}$ NMR (151 MHz, $CDCl_3$)

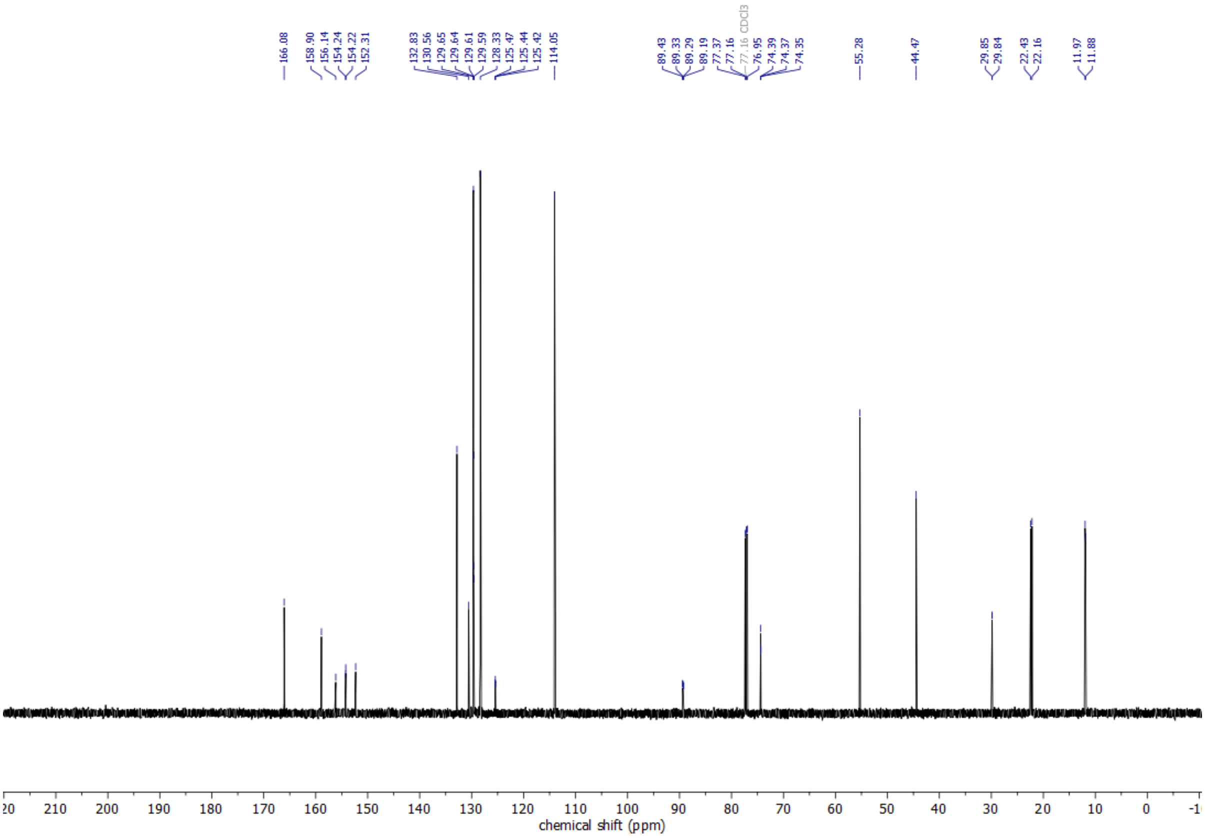

^{19}F NMR (564 MHz, $CDCl_3$)

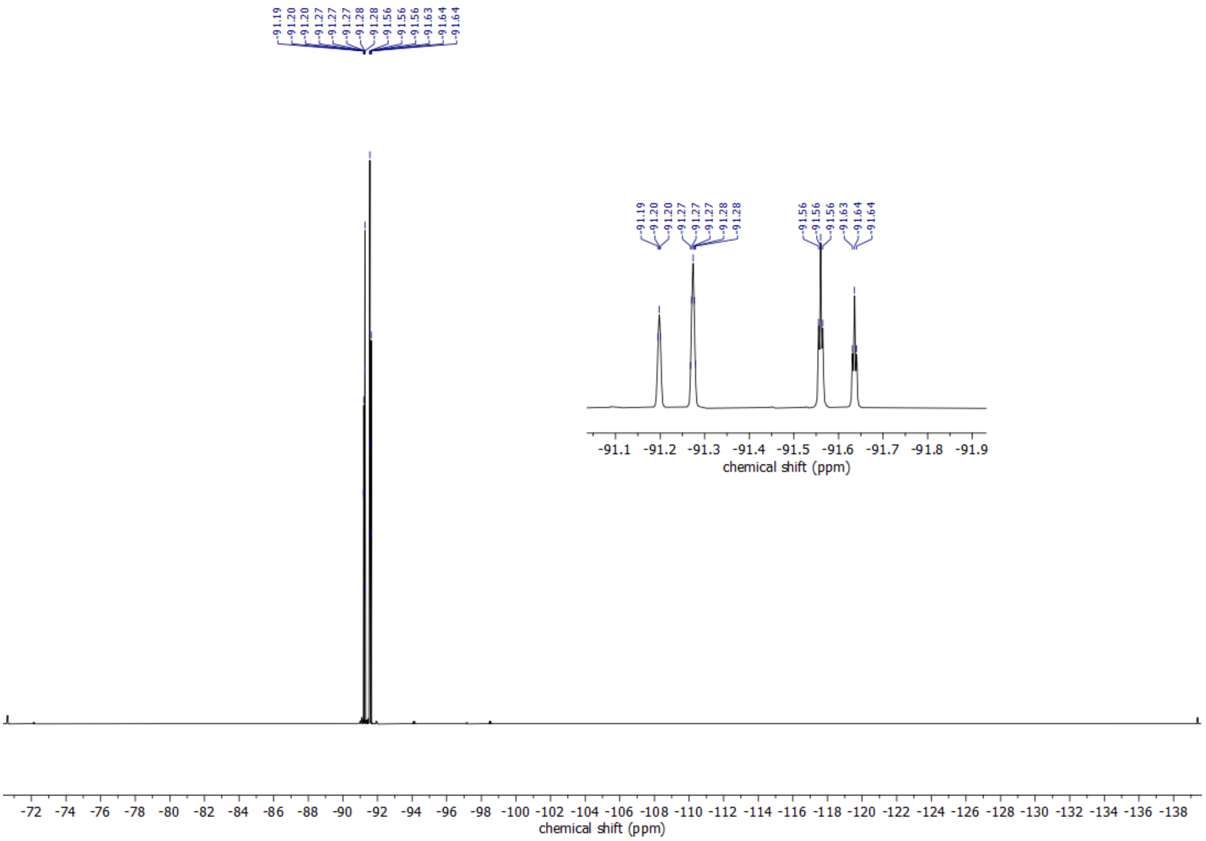

^{1}H NMR (599 MHz, CDCl$_3$)

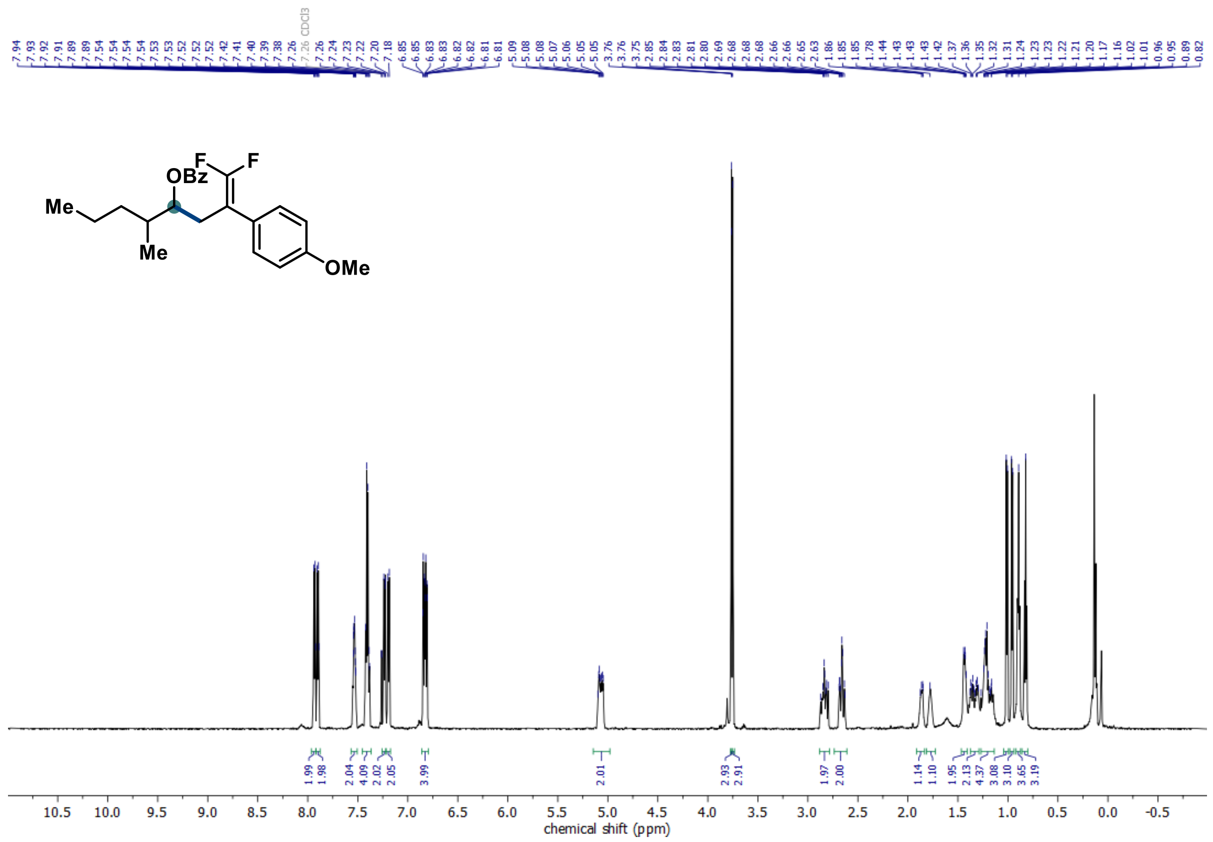

^{13}C{^{1}H} NMR (126 MHz, CDCl$_3$)

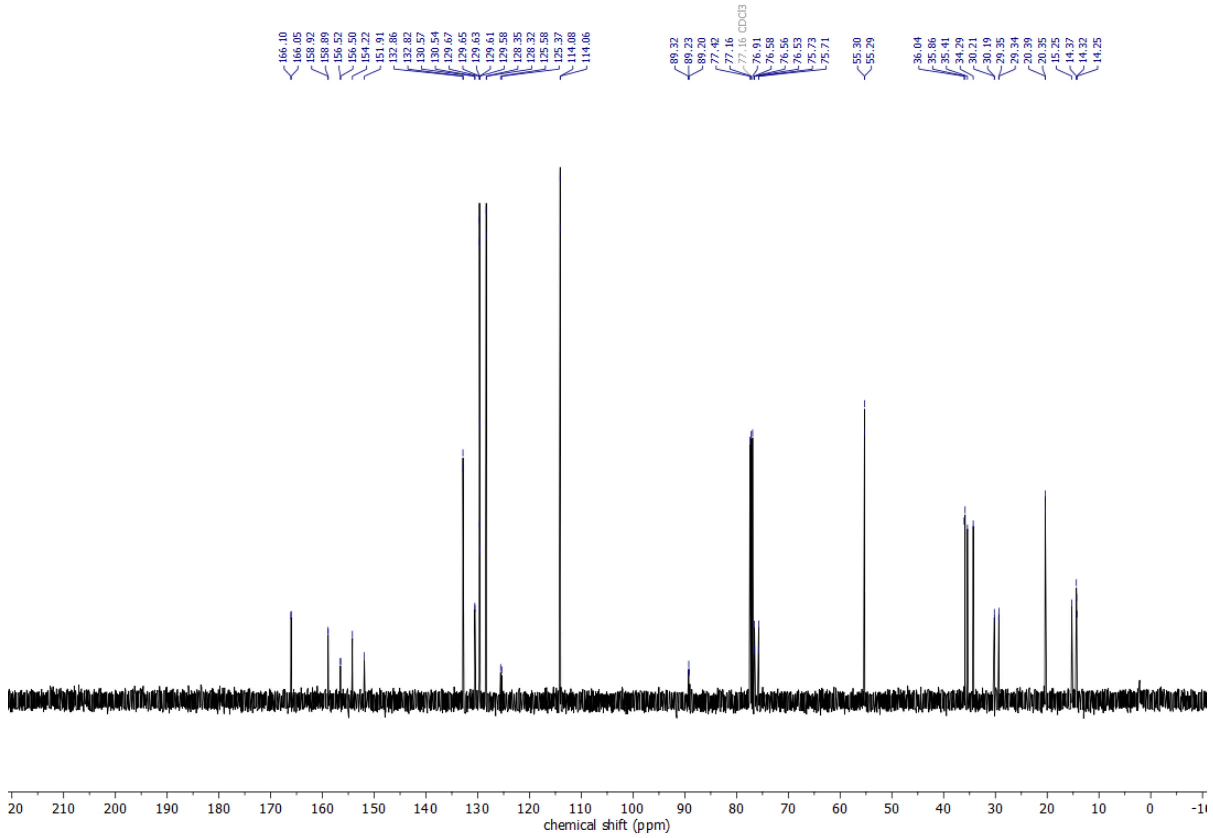

^{19}F NMR (470 MHz, CDCl$_3$)

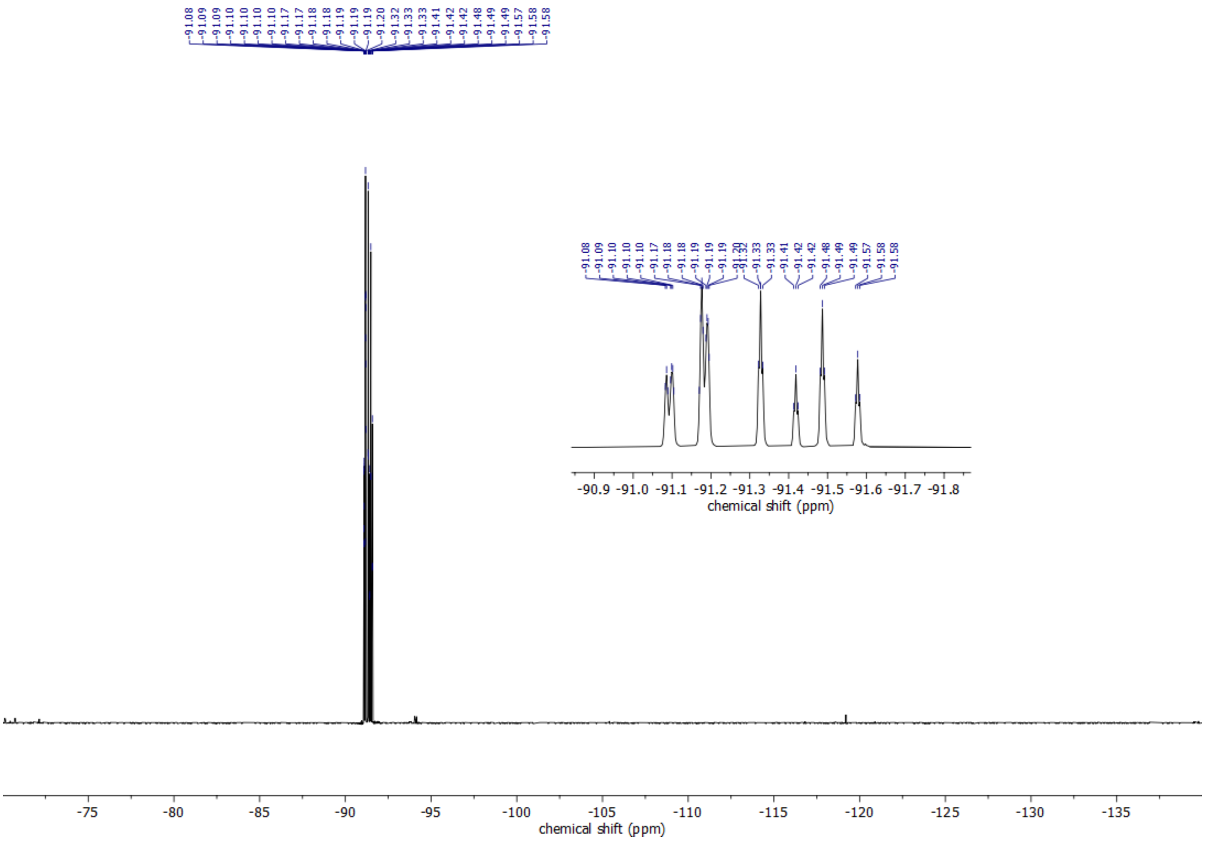

^{1}H NMR (500 MHz, $CDCl_3$)

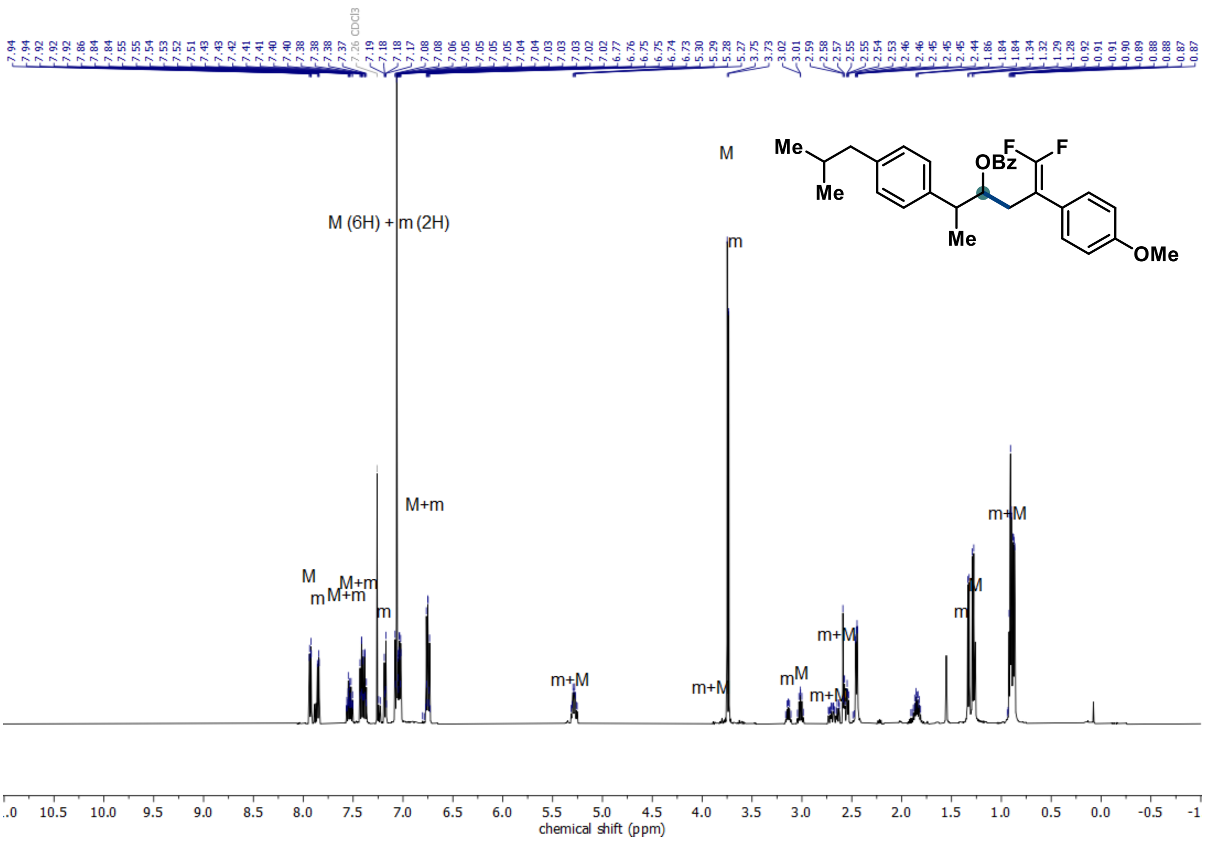

$^{13}C\{^{1}H, ^{19}F\}$ NMR (151 MHz, $CDCl_3$)

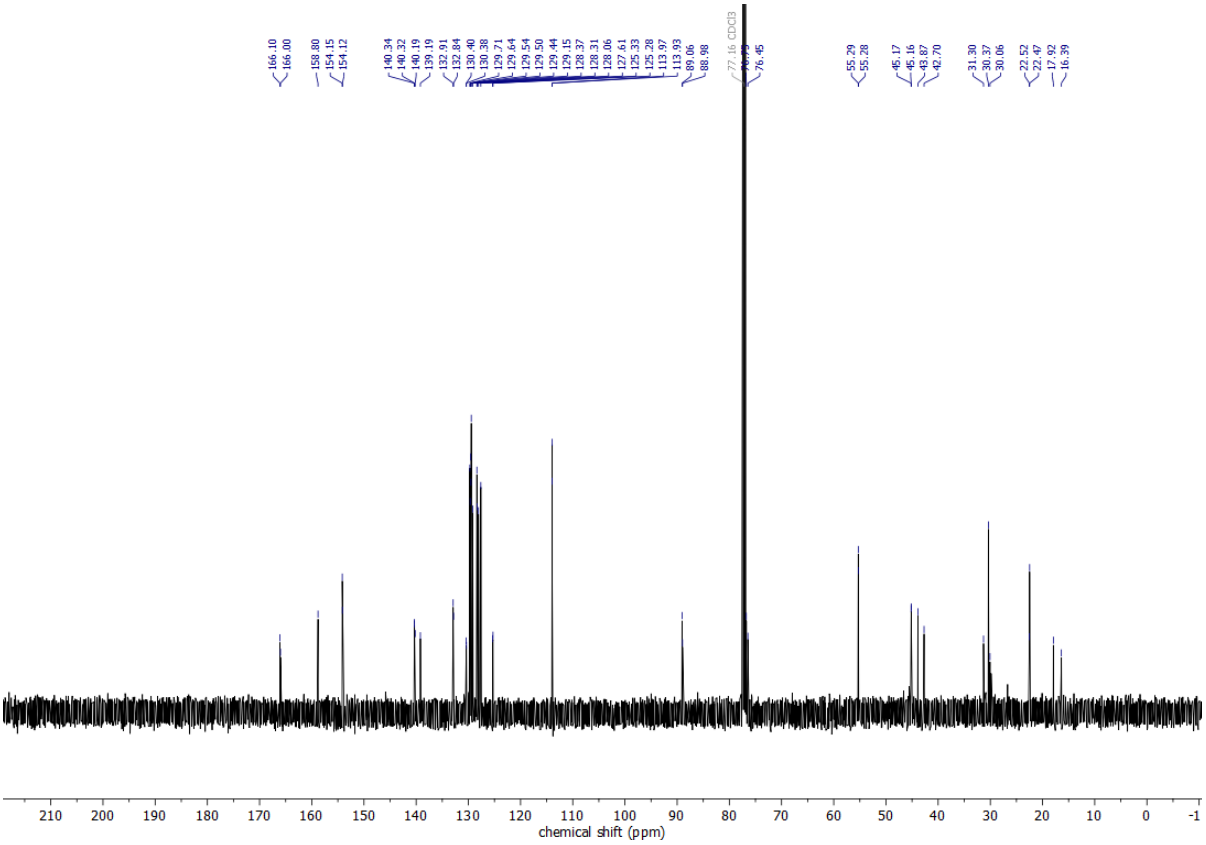

^{19}F NMR (470 MHz, $CDCl_3$)

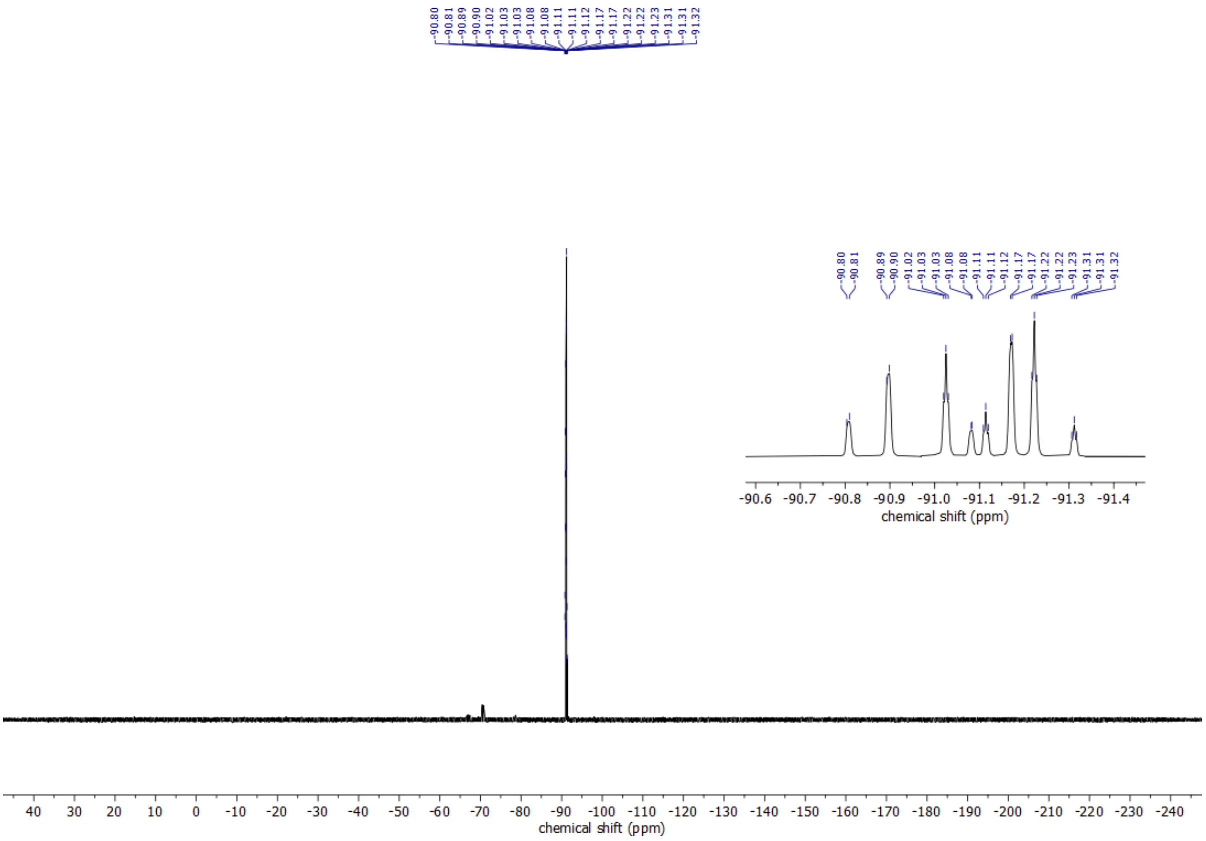

^{1}H-^{1}H COSY NMR (500 MHz, $CDCl_3$)

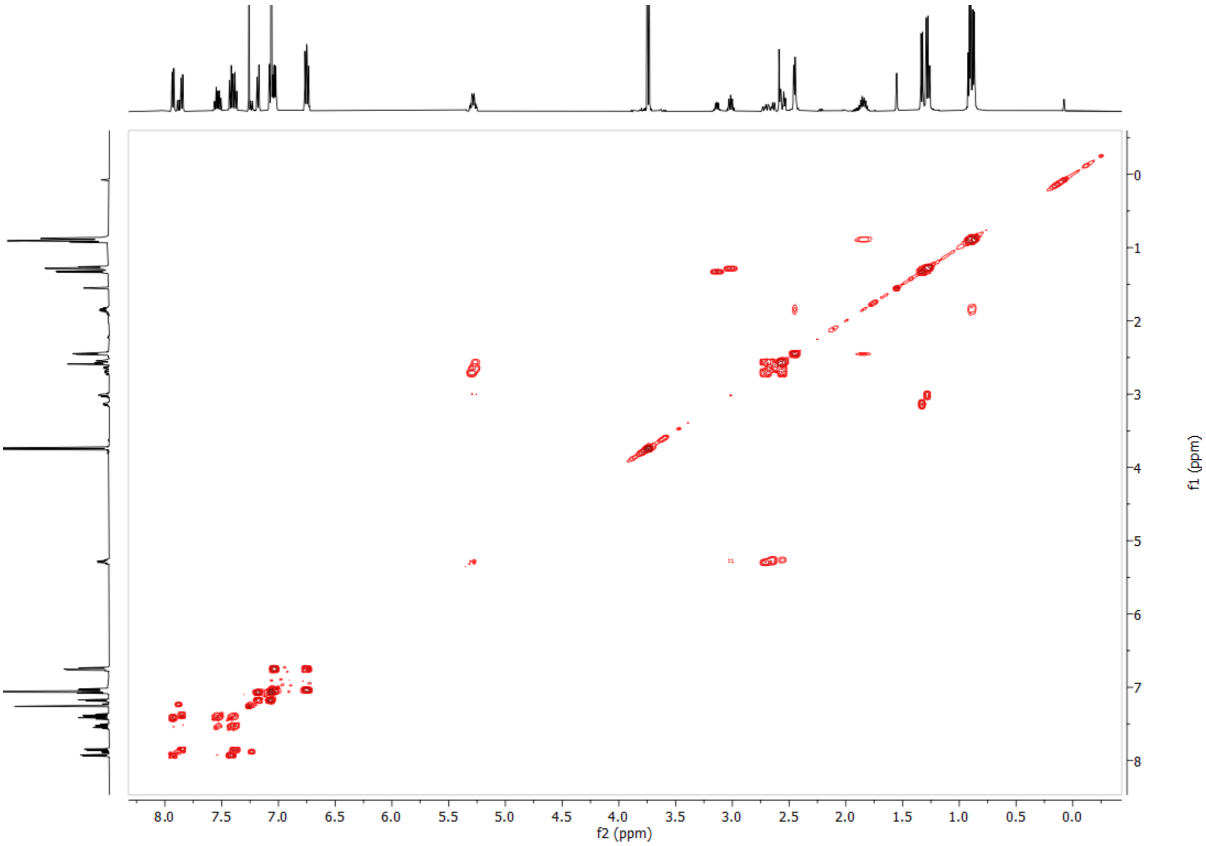

^1H-^{13}C HSQC NMR (500 MHz, $CDCl_3$)

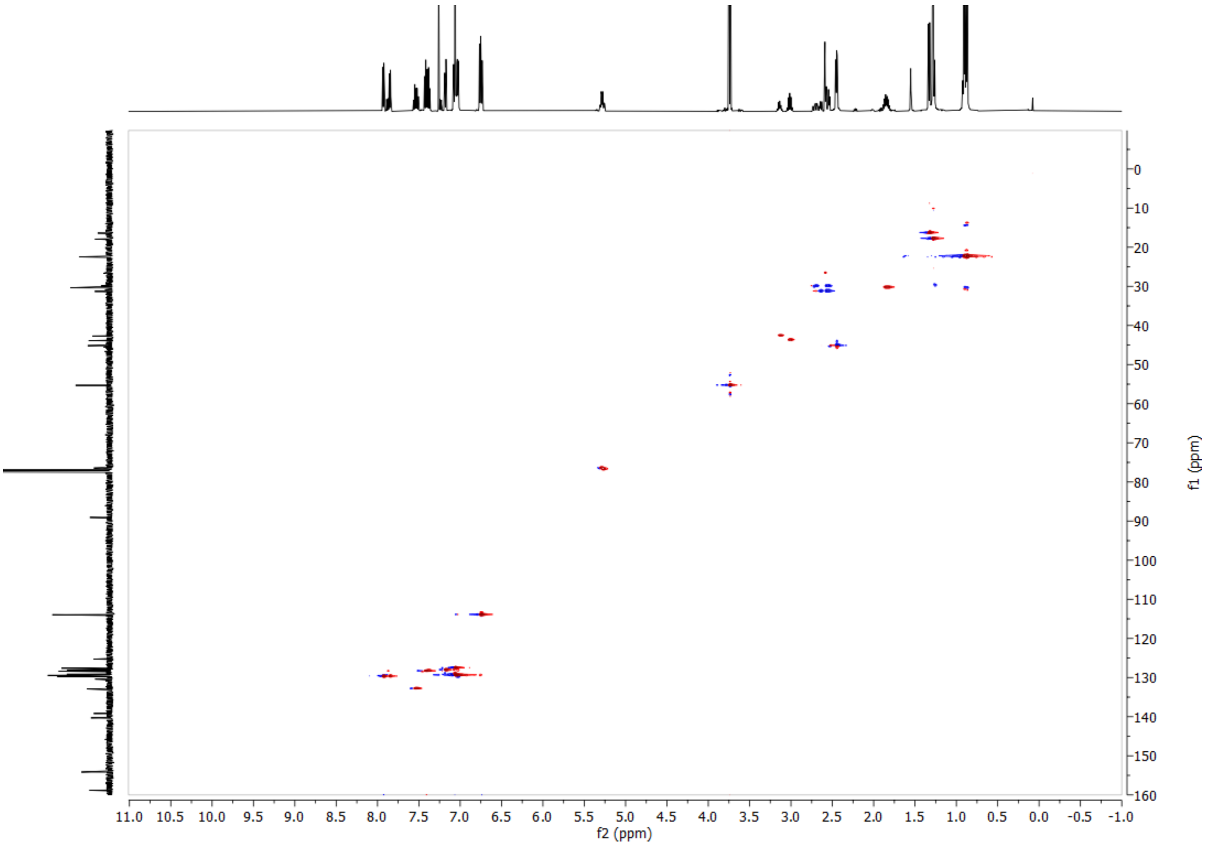

^1H-^{13}C HMBC NMR (500 MHz, $CDCl_3$)

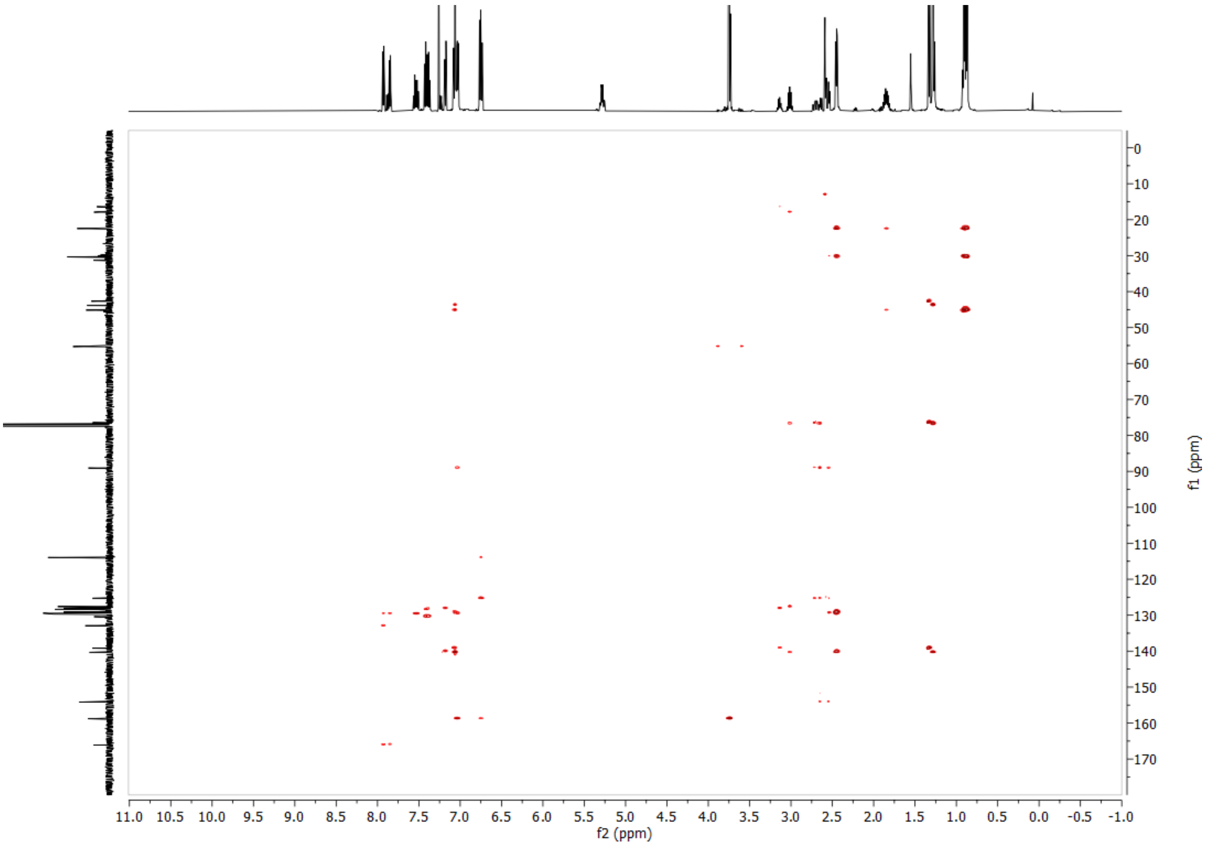

^{1}H NMR (599 MHz, $CDCl_3$)

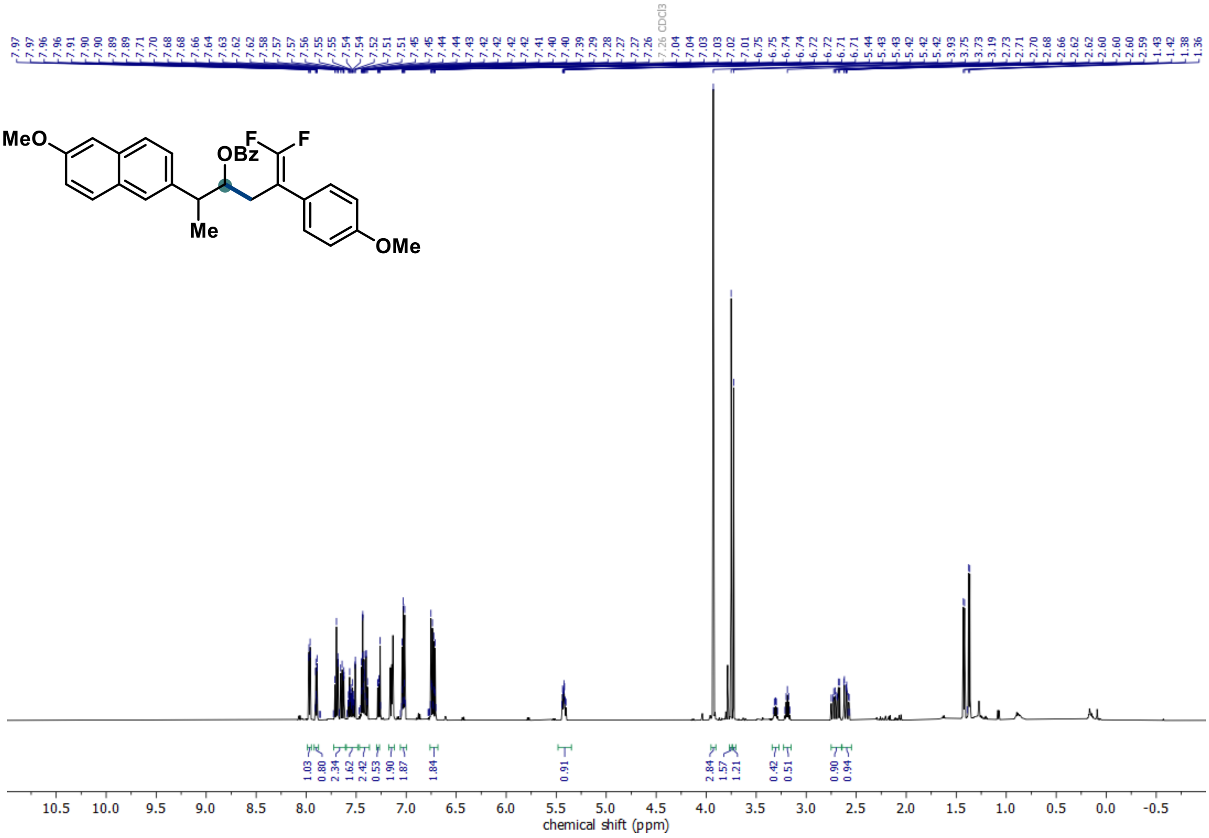

$^{13}C\{^{1}H, ^{19}F\}$ NMR (151 MHz, $CDCl_3$)

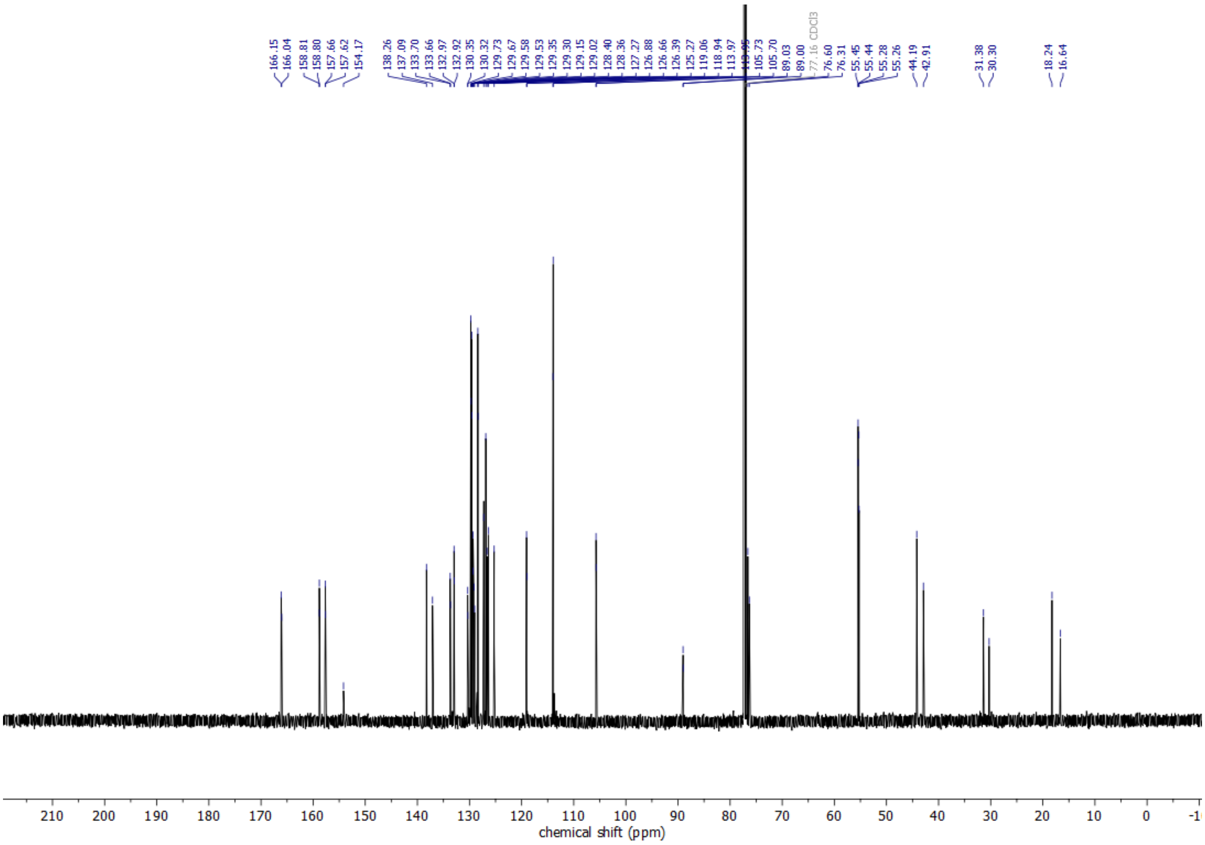

^{19}F NMR (564 MHz, $CDCl_3$)

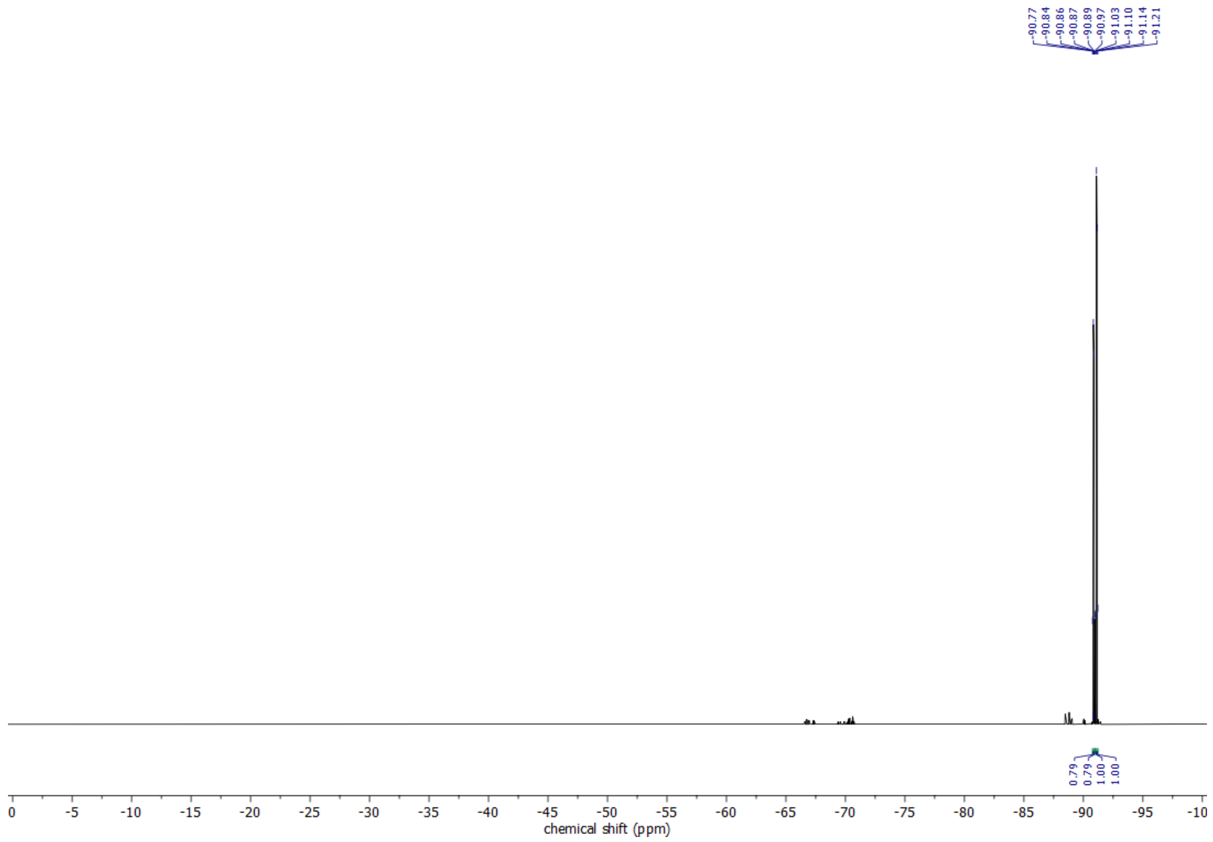

^{1}H-^{1}H COSY NMR (599 MHz, $CDCl_3$)

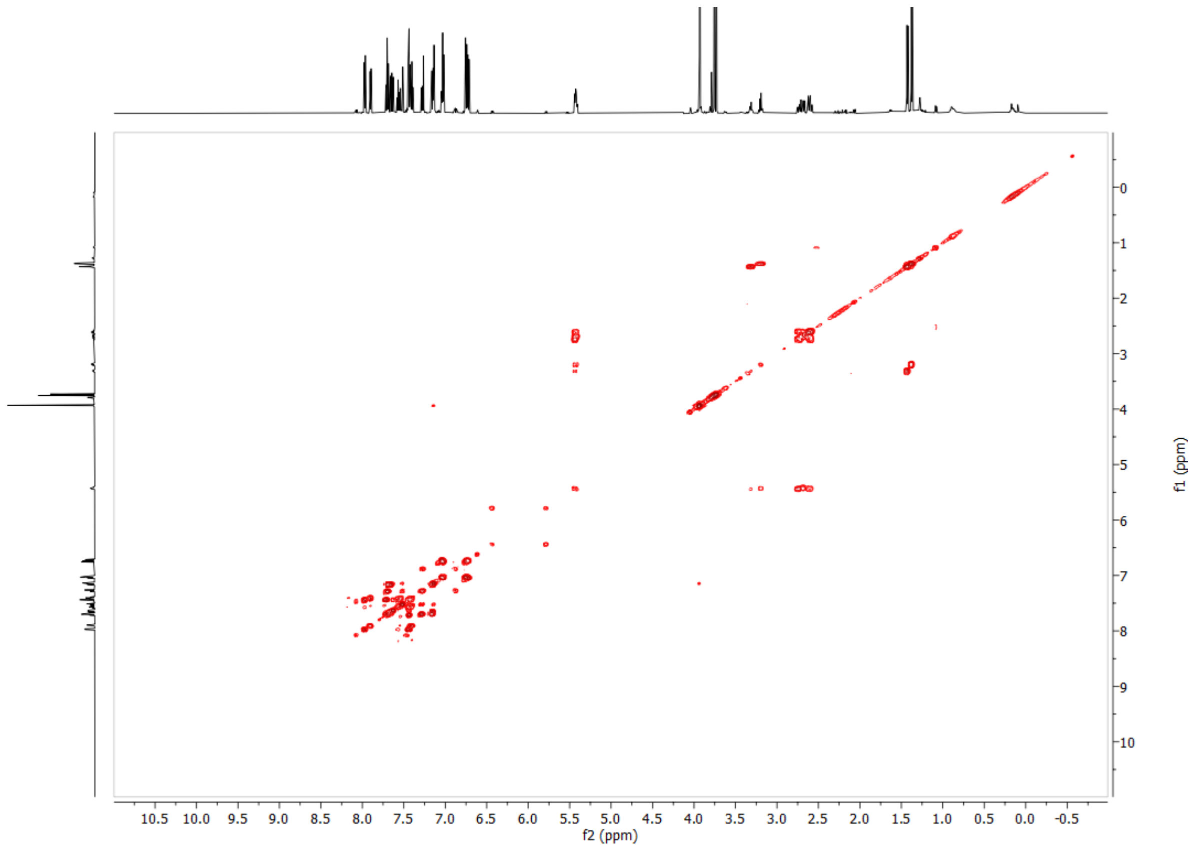

^{1}H-^{13}C HSQC NMR (599 MHz, $CDCl_3$)

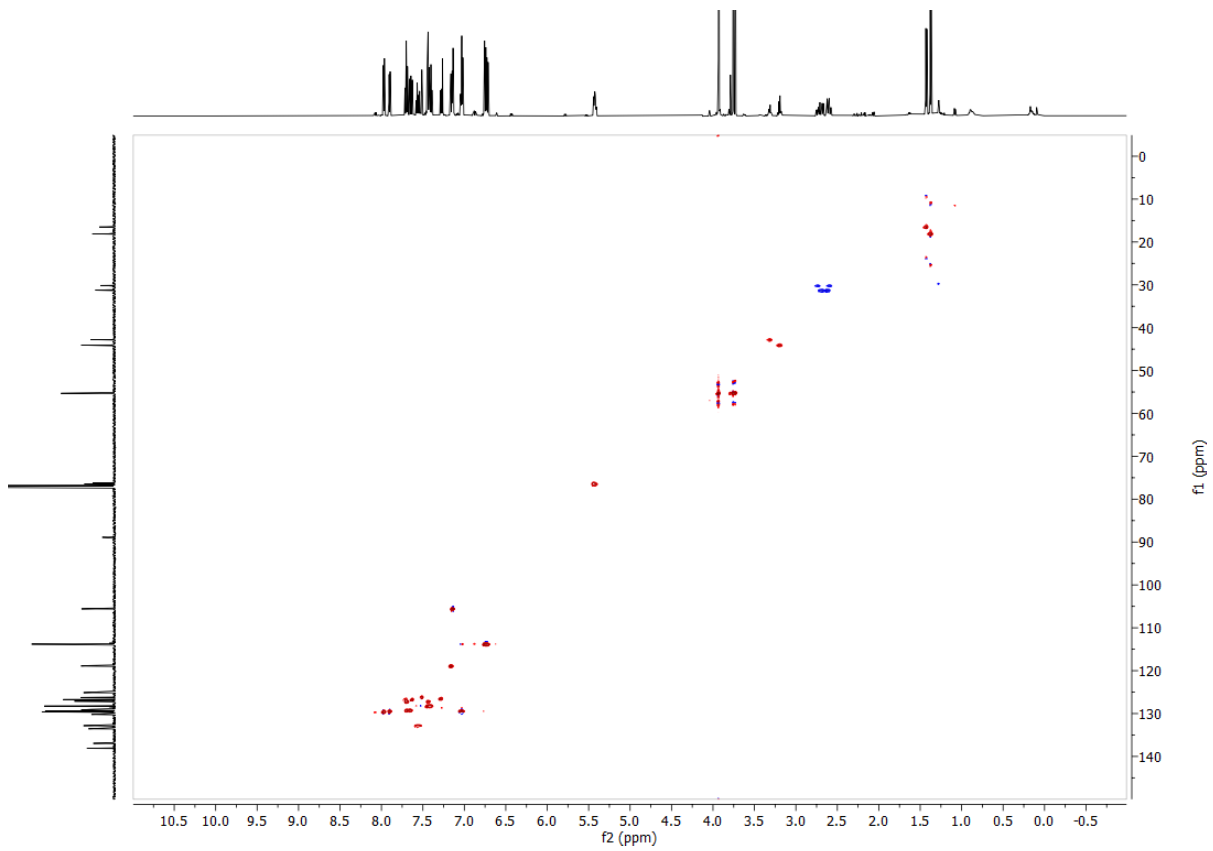

^{1}H-^{13}C HMBC NMR (599 MHz, $CDCl_3$)

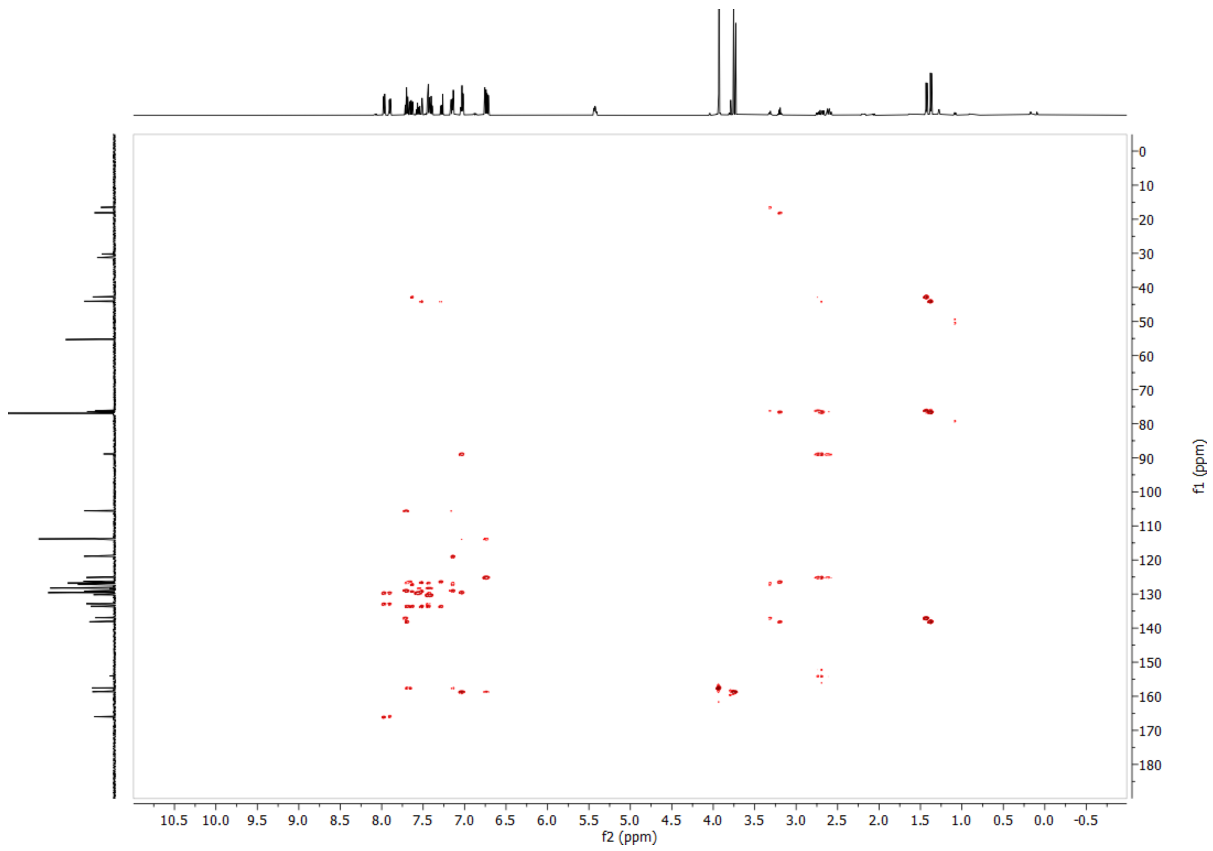

^{1}H NMR (599 MHz, $CDCl_3$)

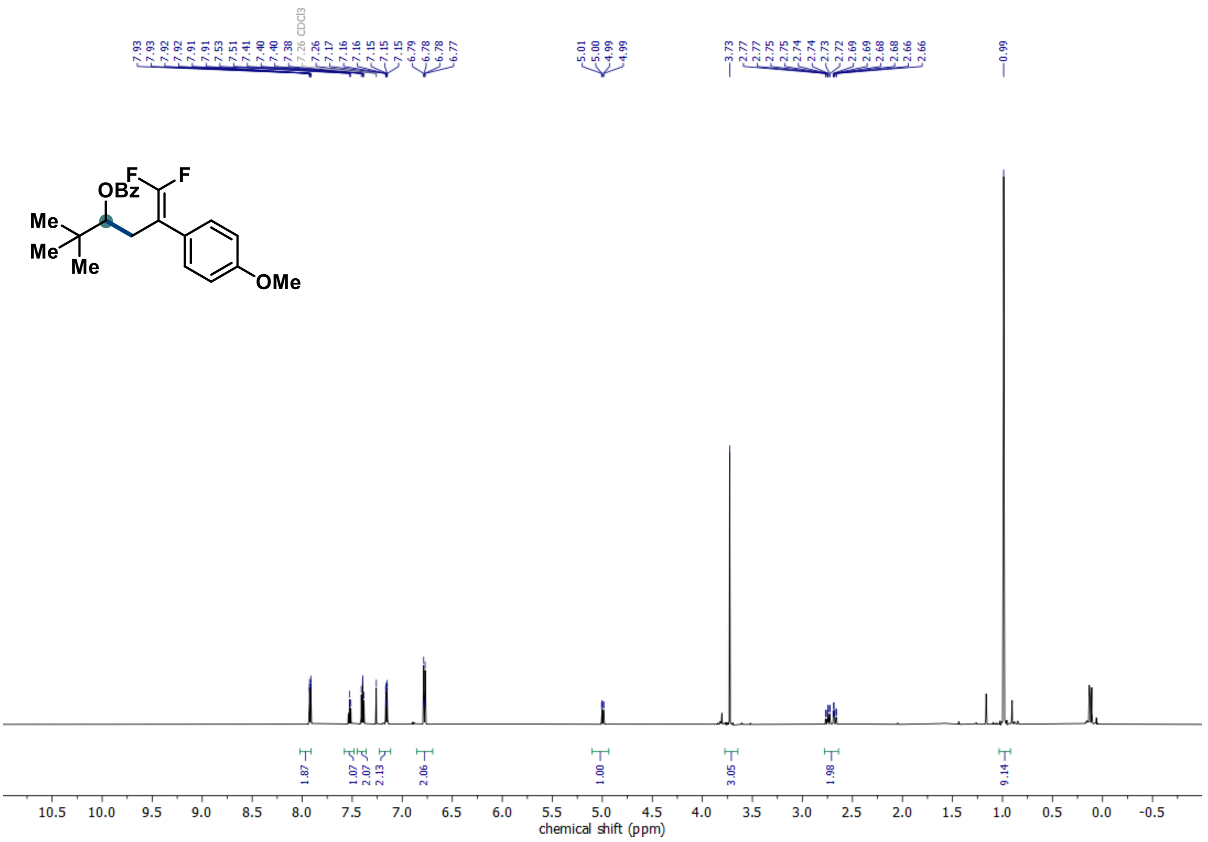

$^{13}C\{^{1}H\}$ NMR (151 MHz, $CDCl_3$)

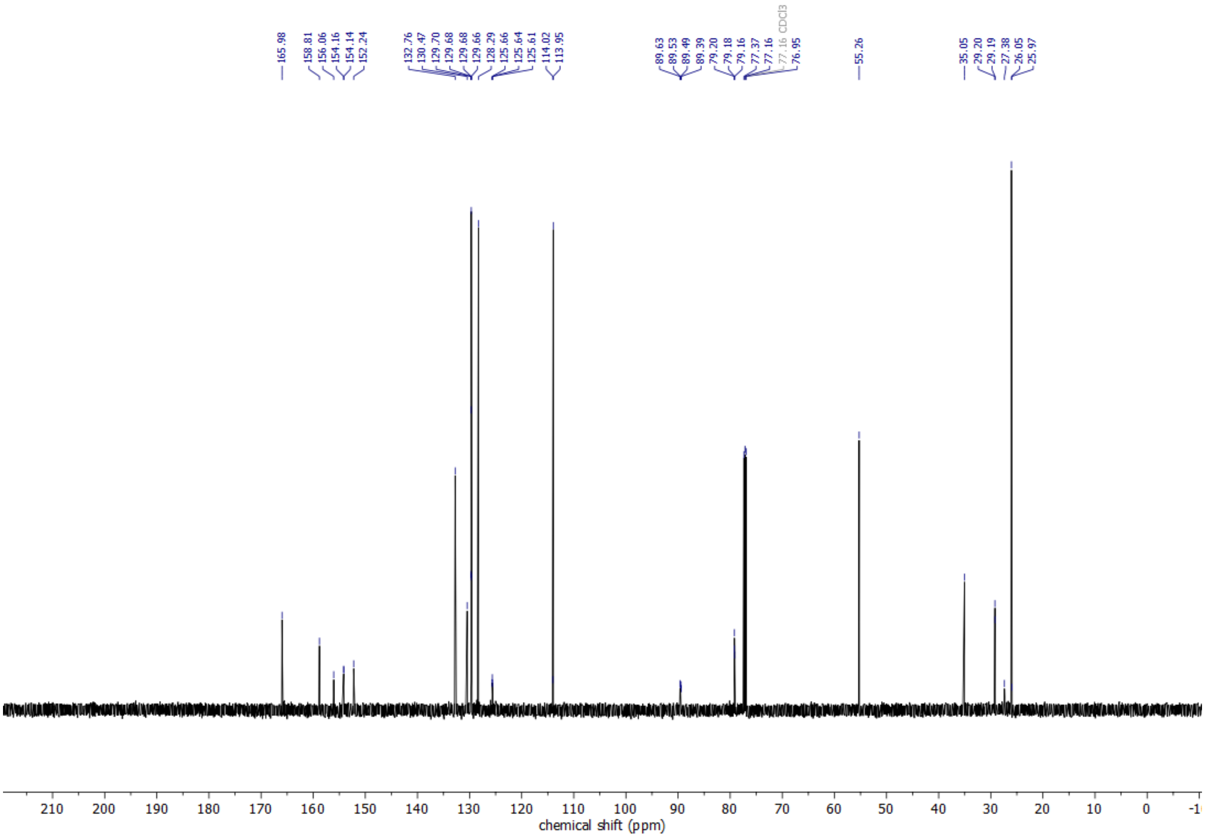

^{19}F NMR (564 MHz, $CDCl_3$)

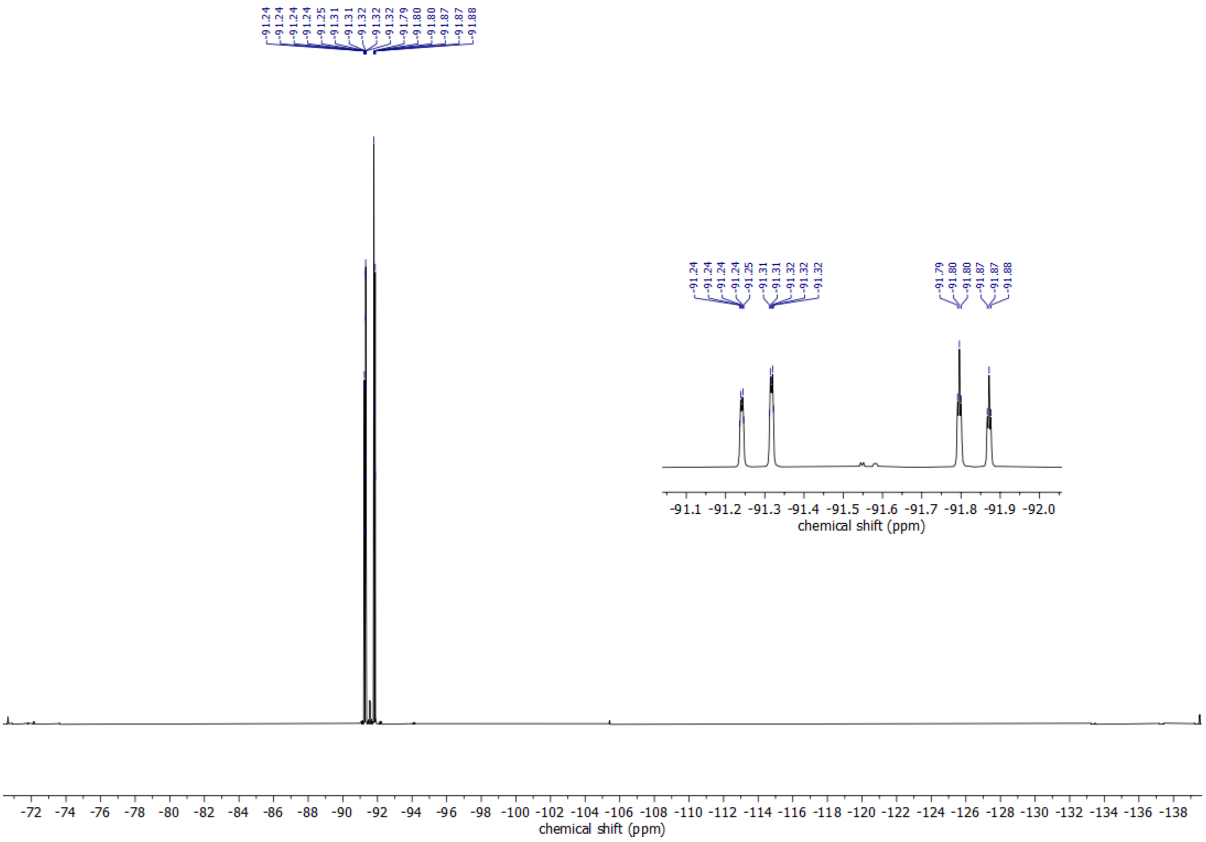

^{1}H NMR (400 MHz, $CDCl_3$)

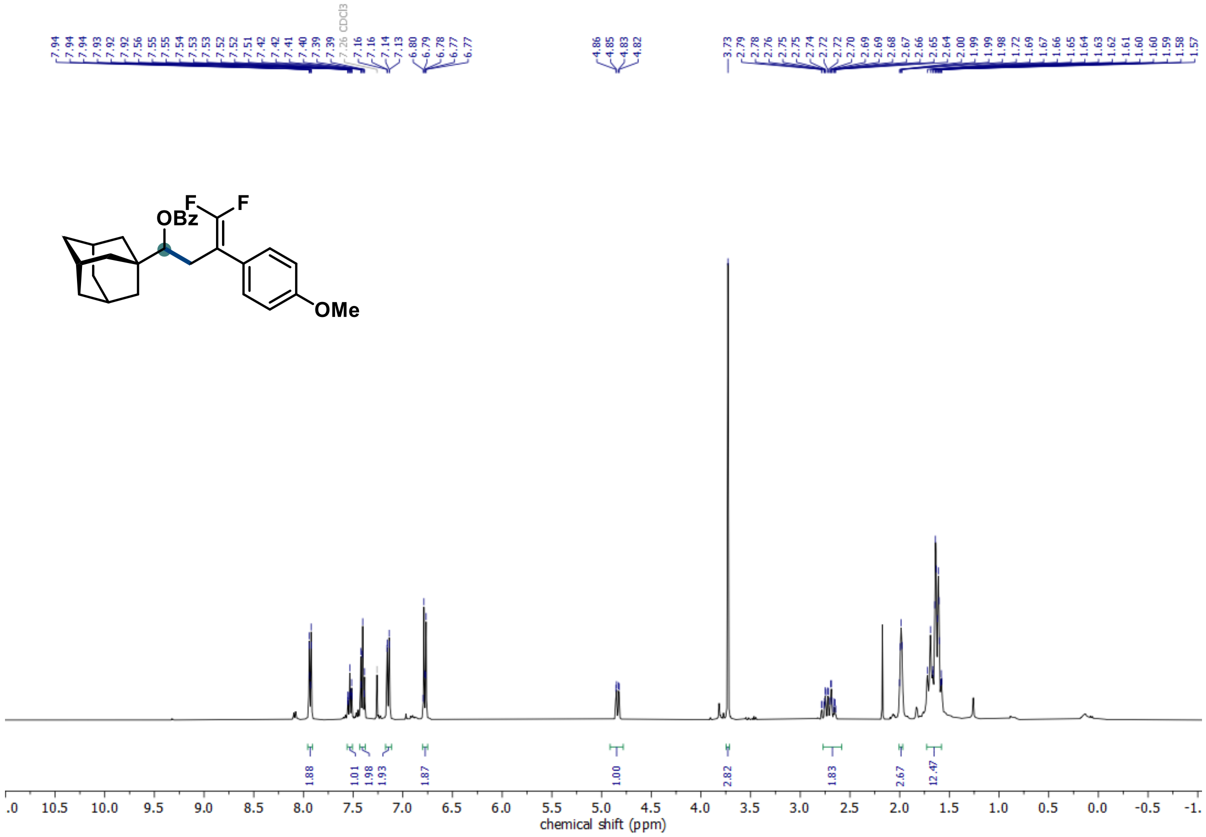

$^{13}C\{^{1}H\}$ NMR (101 MHz, $CDCl_3$)

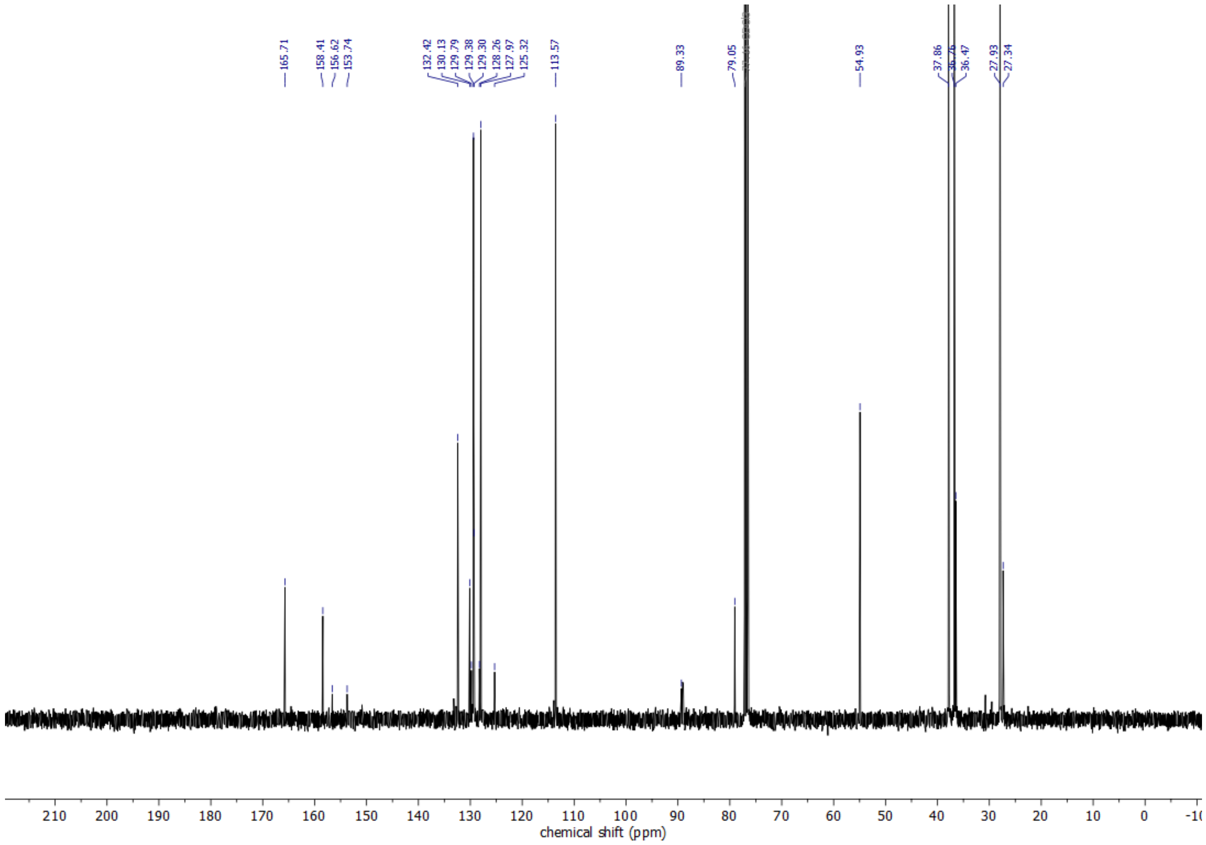

$^{19}F\{^1H\}$ NMR (377 MHz, CDCl$_3$)

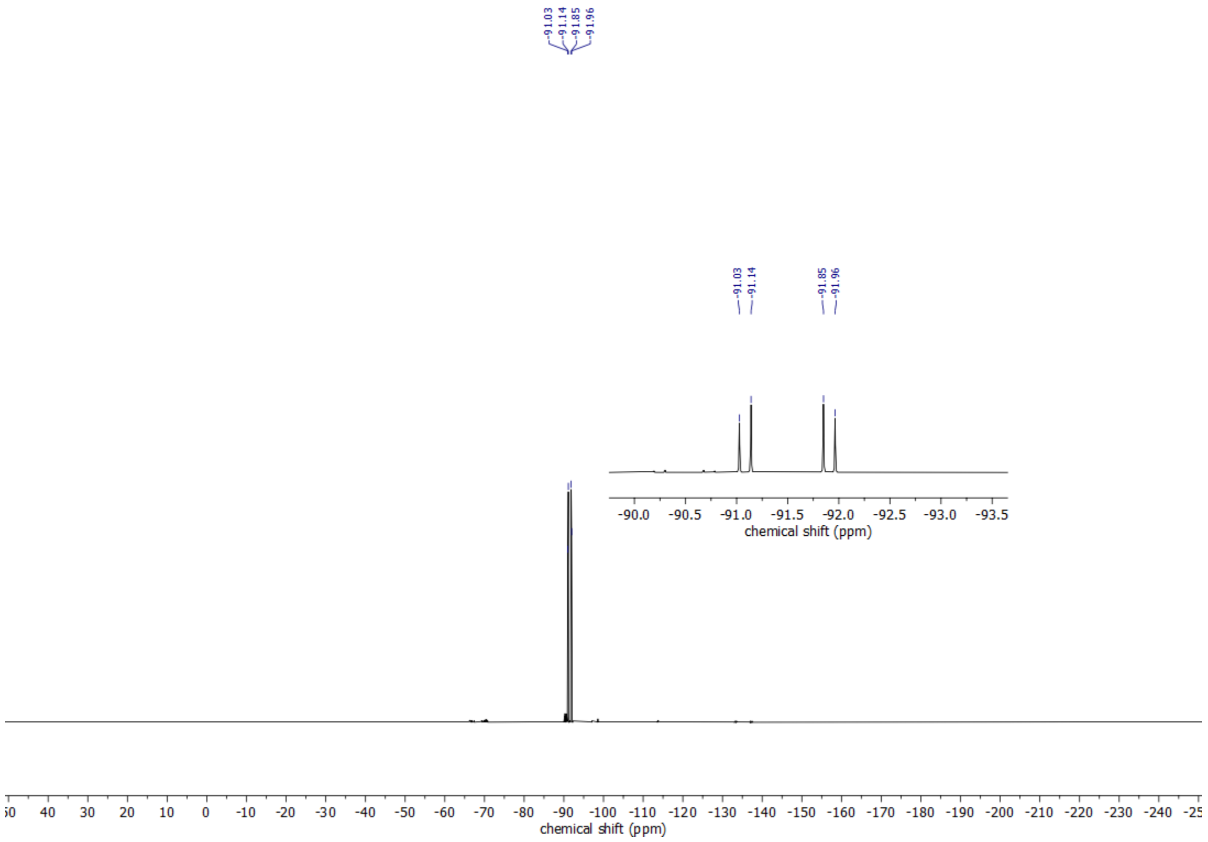

^{1}H NMR (500 MHz, CDCl$_3$)

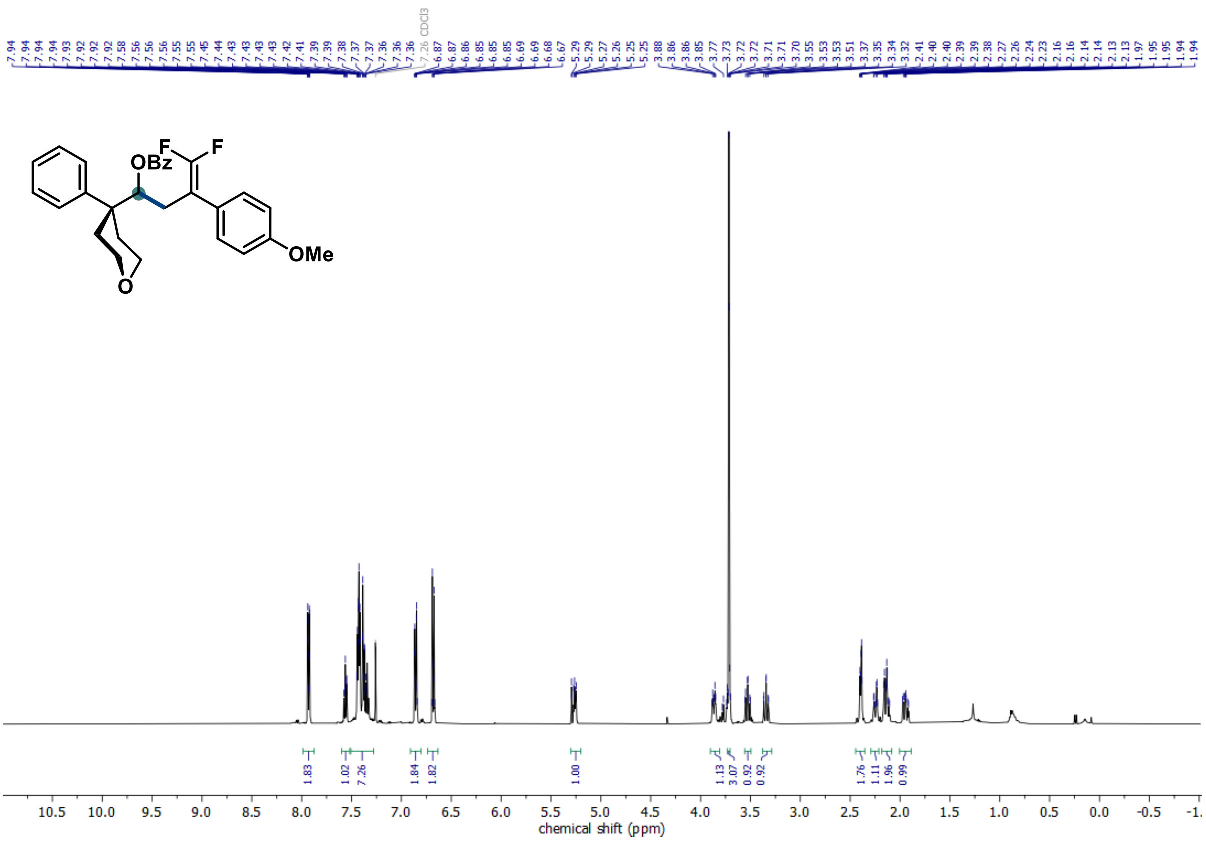

^{13}C{^{1}H, ^{19}F} NMR (126 MHz, CDCl$_3$)

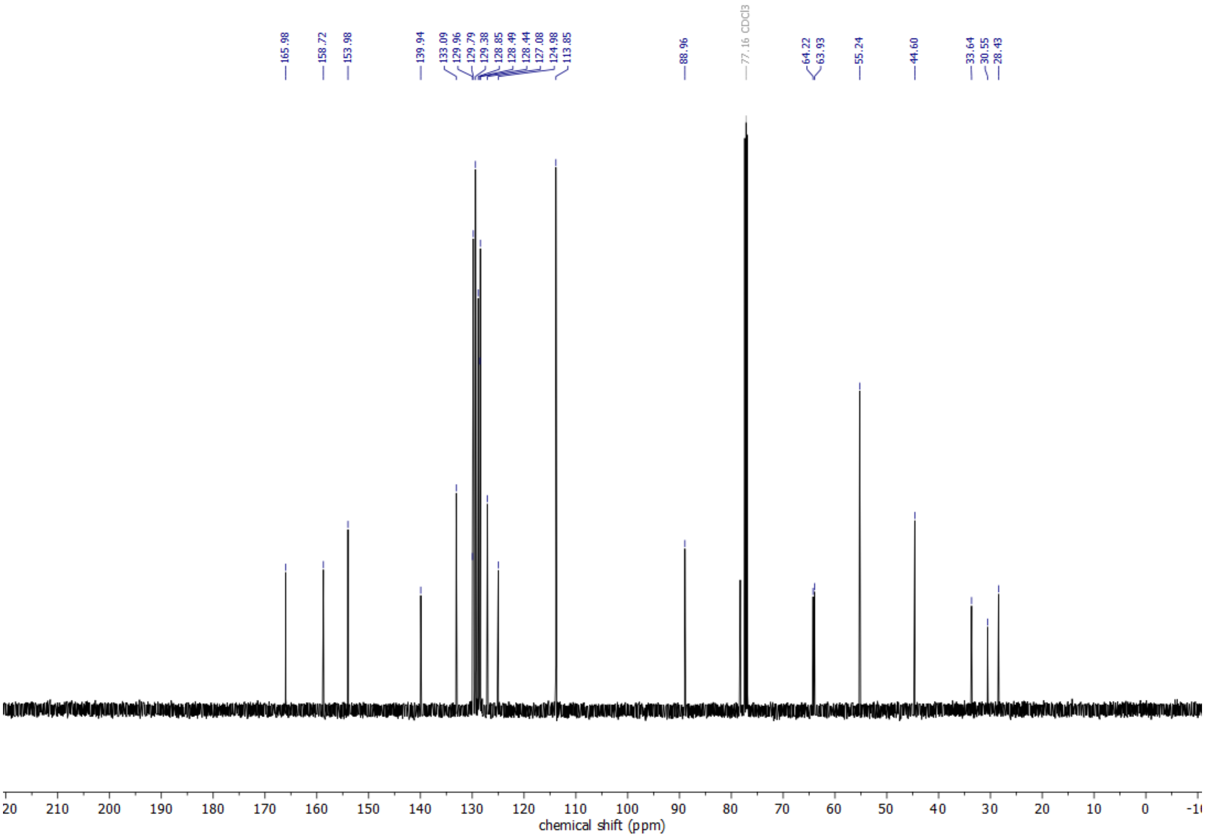

$^{19}F\{^{1}H\}$ NMR (470 MHz, $CDCl_3$)

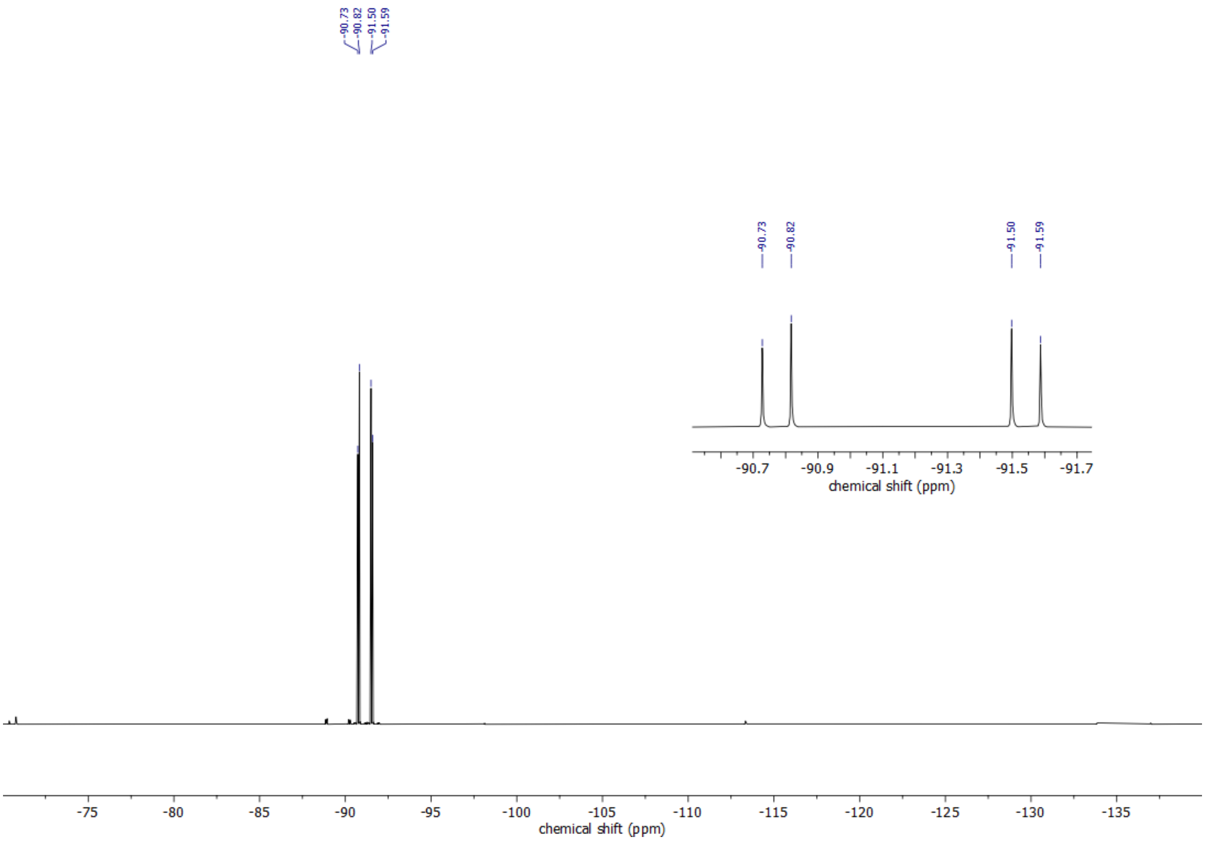

^{1}H NMR (400 MHz, $CDCl_3$)

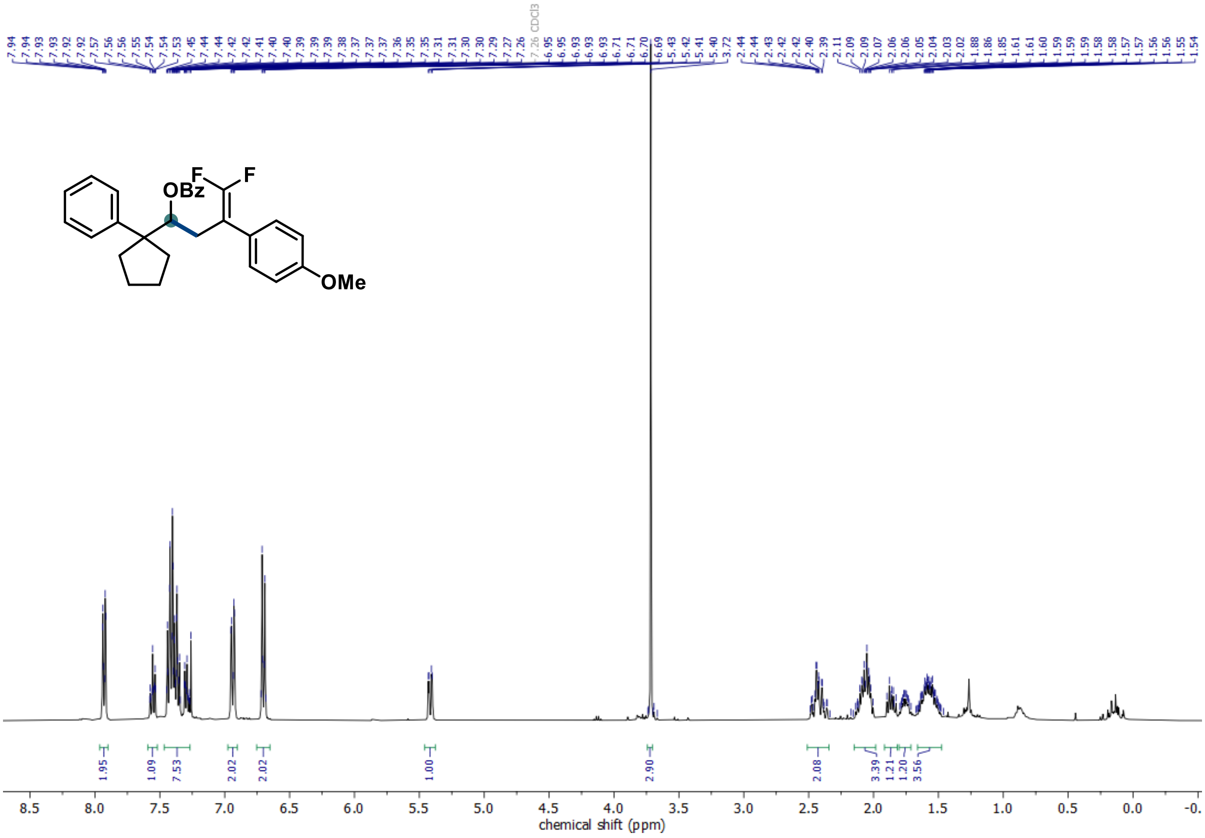

^{13}C{^{1}H} NMR (101 MHz, $CDCl_3$)

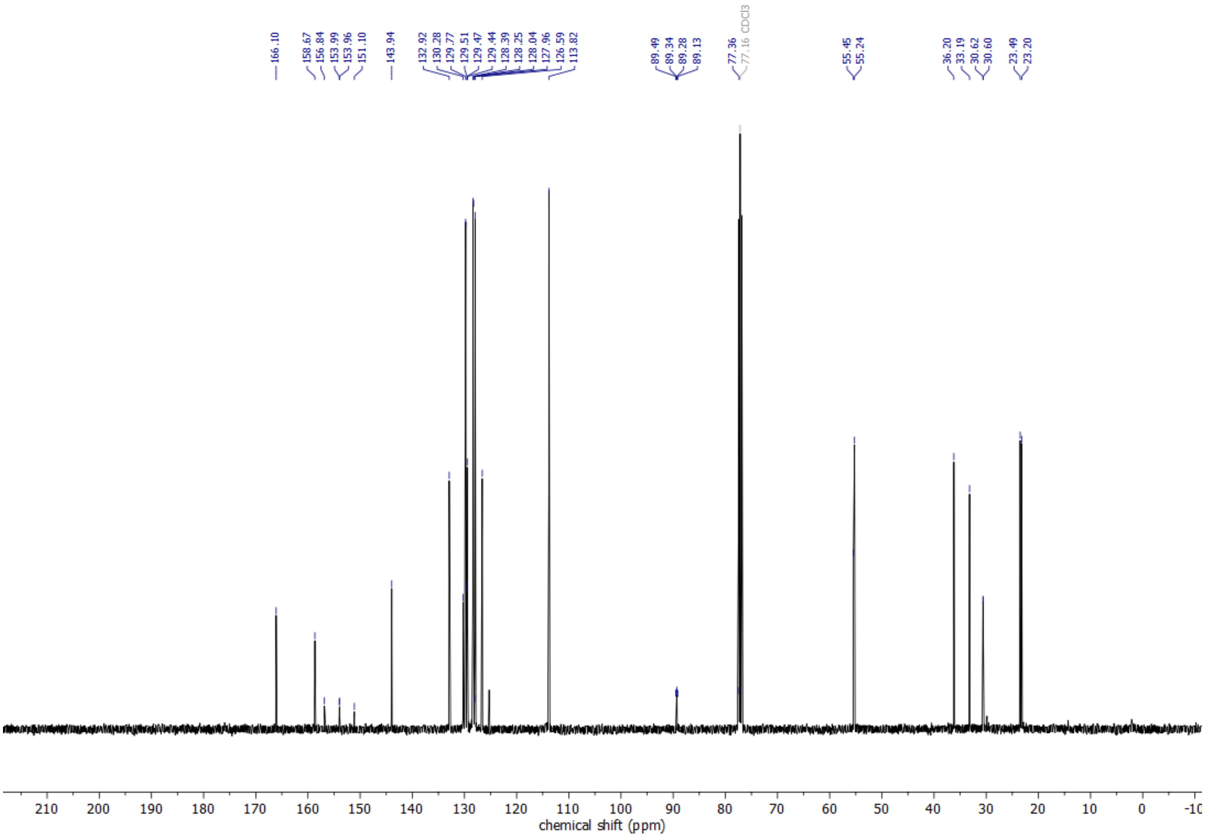

$^{19}F\{^{1}H\}$ NMR (377 MHz, $CDCl_3$)

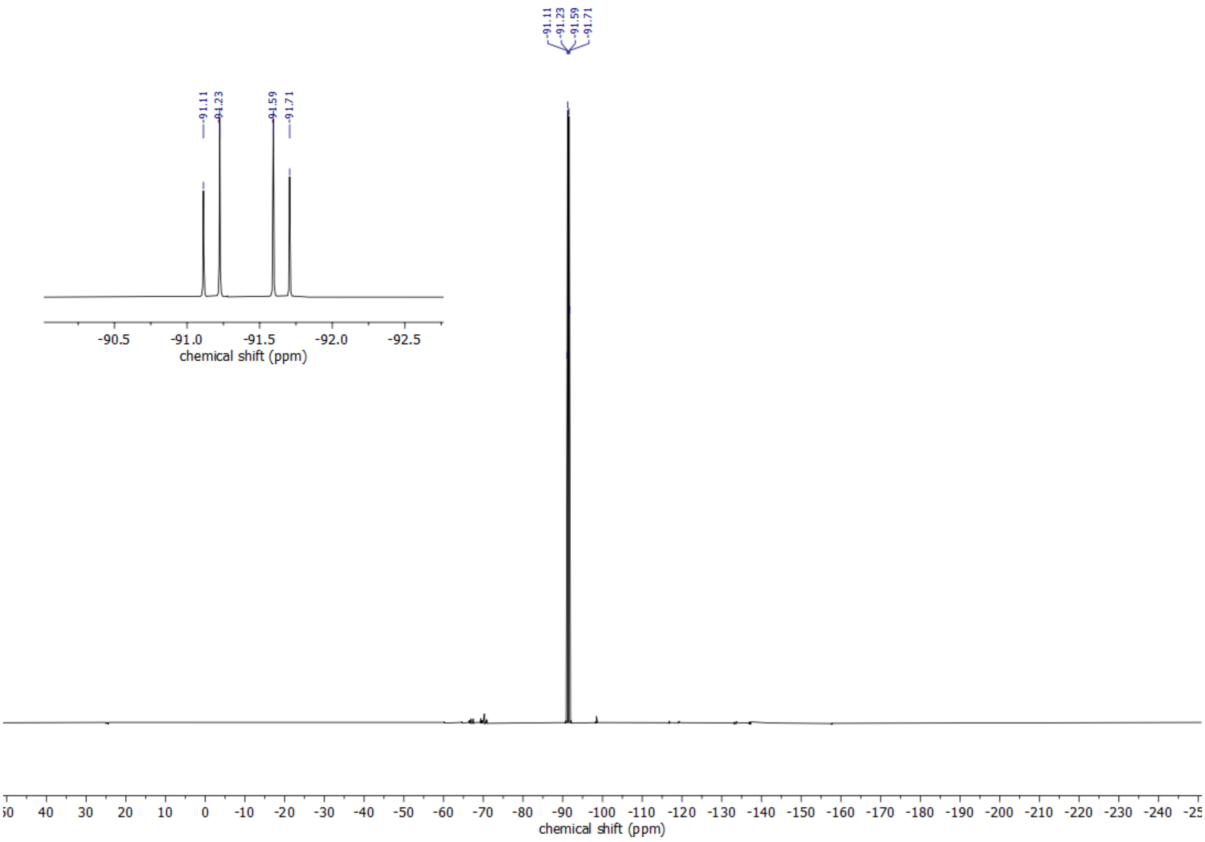

^{1}H NMR (400 MHz, CDCl$_3$)

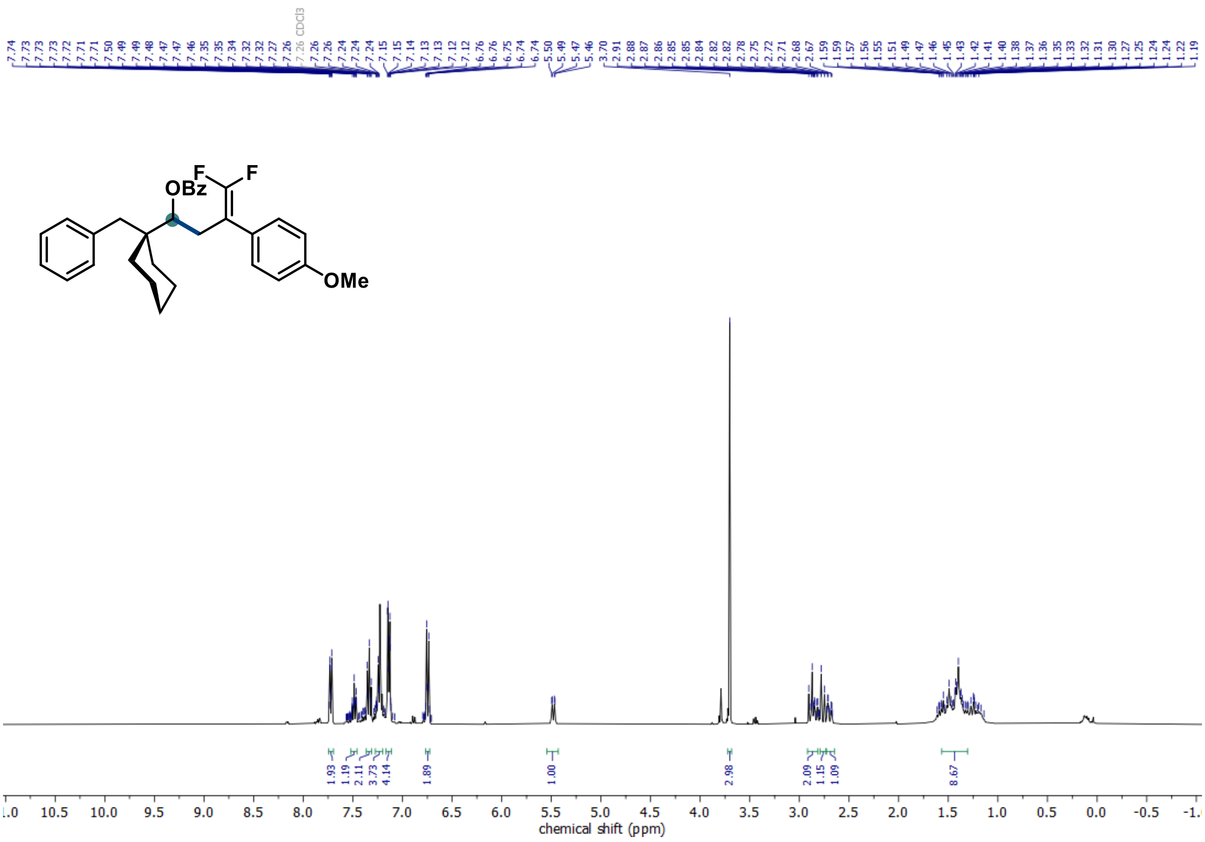

^{13}C{^{1}H} NMR (101 MHz, CDCl$_3$)

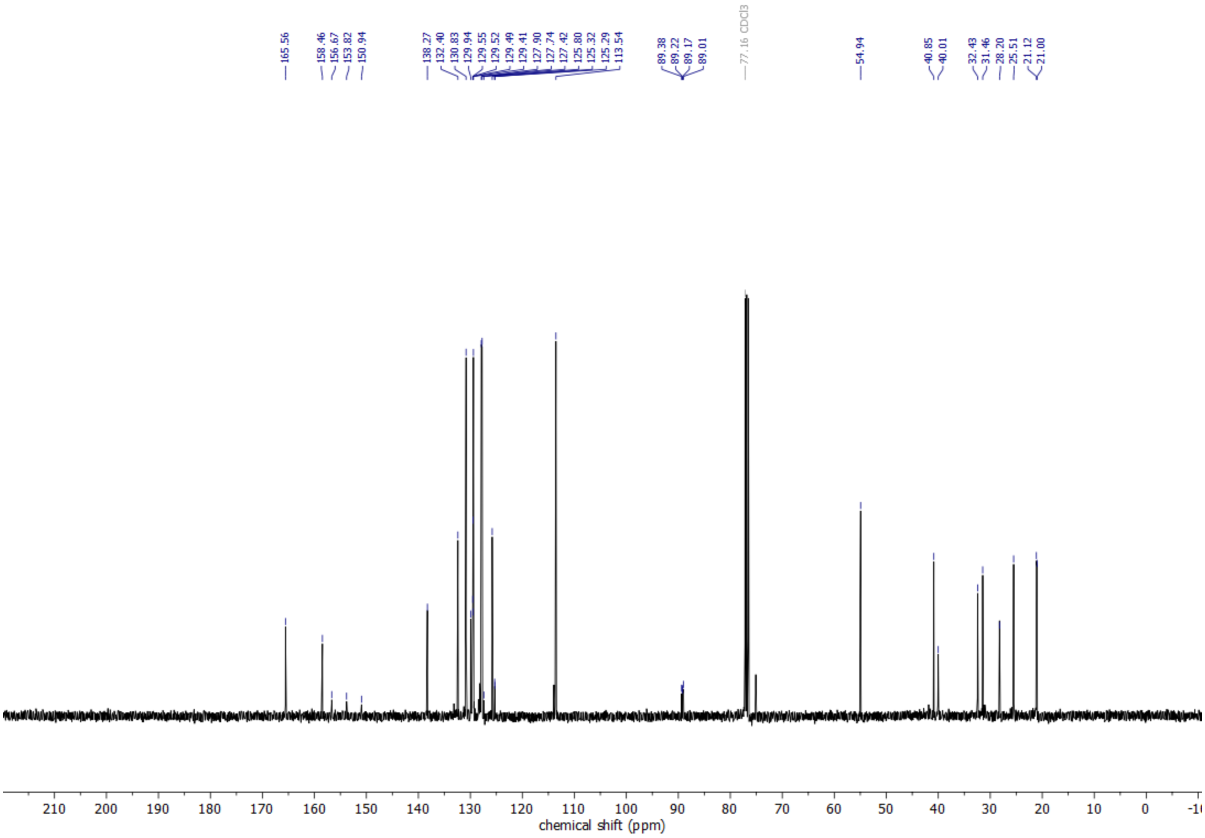

$^{19}F\{^{1}H\}$ NMR (377 MHz, $CDCl_3$)

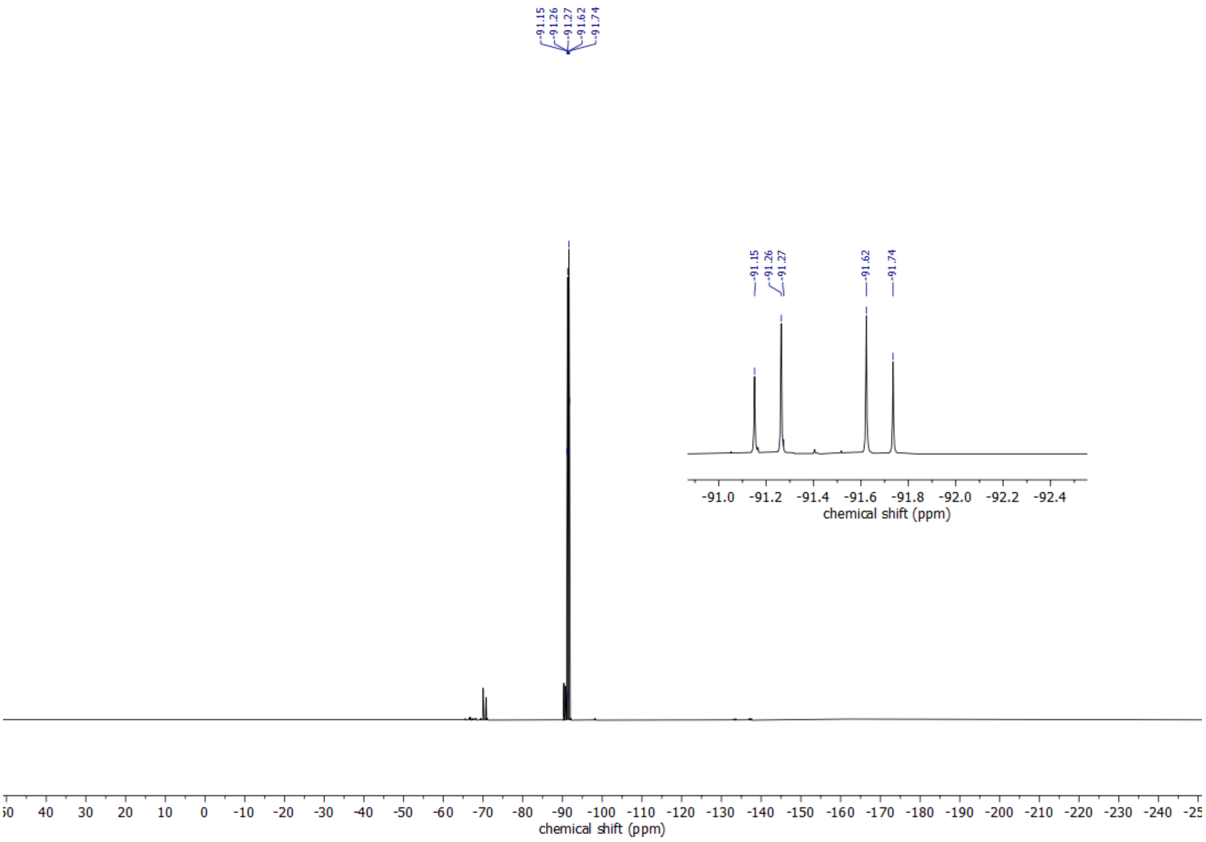

^{1}H NMR (400 MHz, $CDCl_3$)

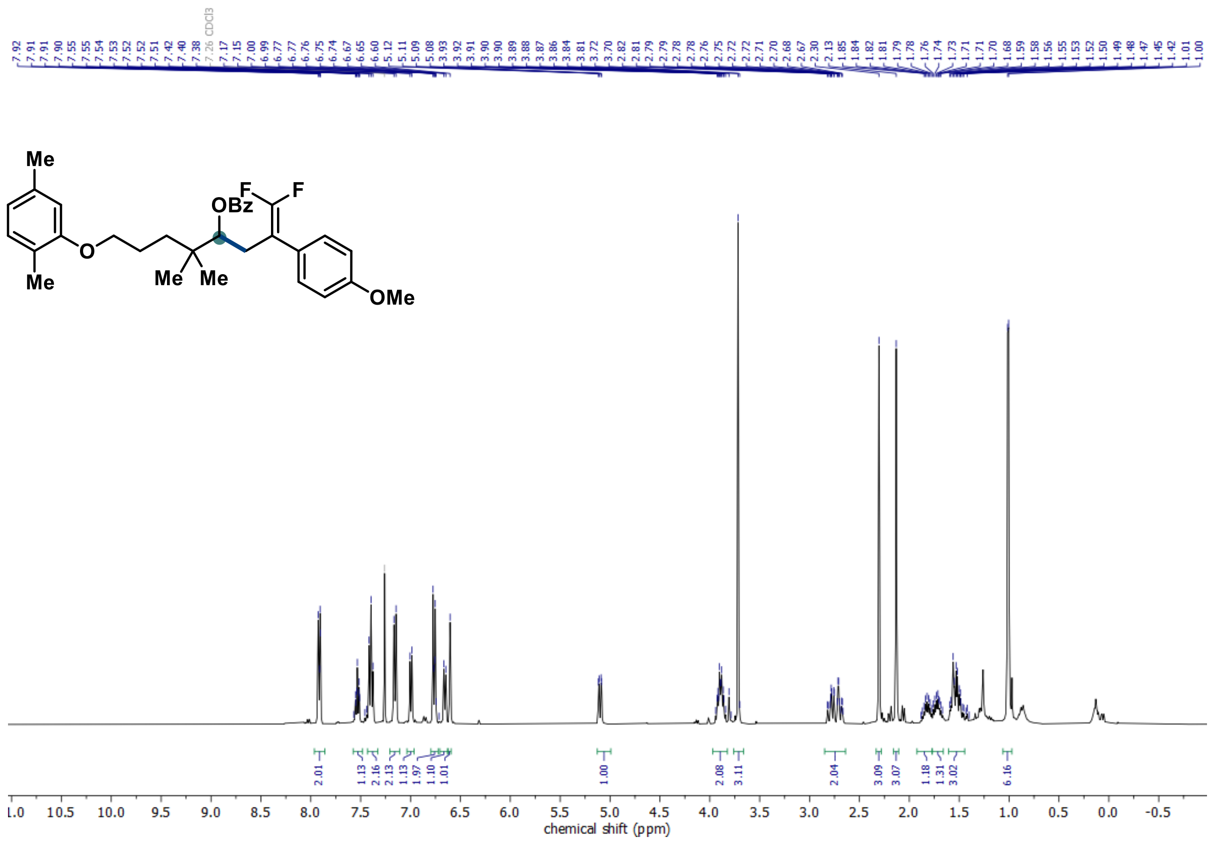

$^{13}C\{^{1}H\}$ NMR (101 MHz, $CDCl_3$)

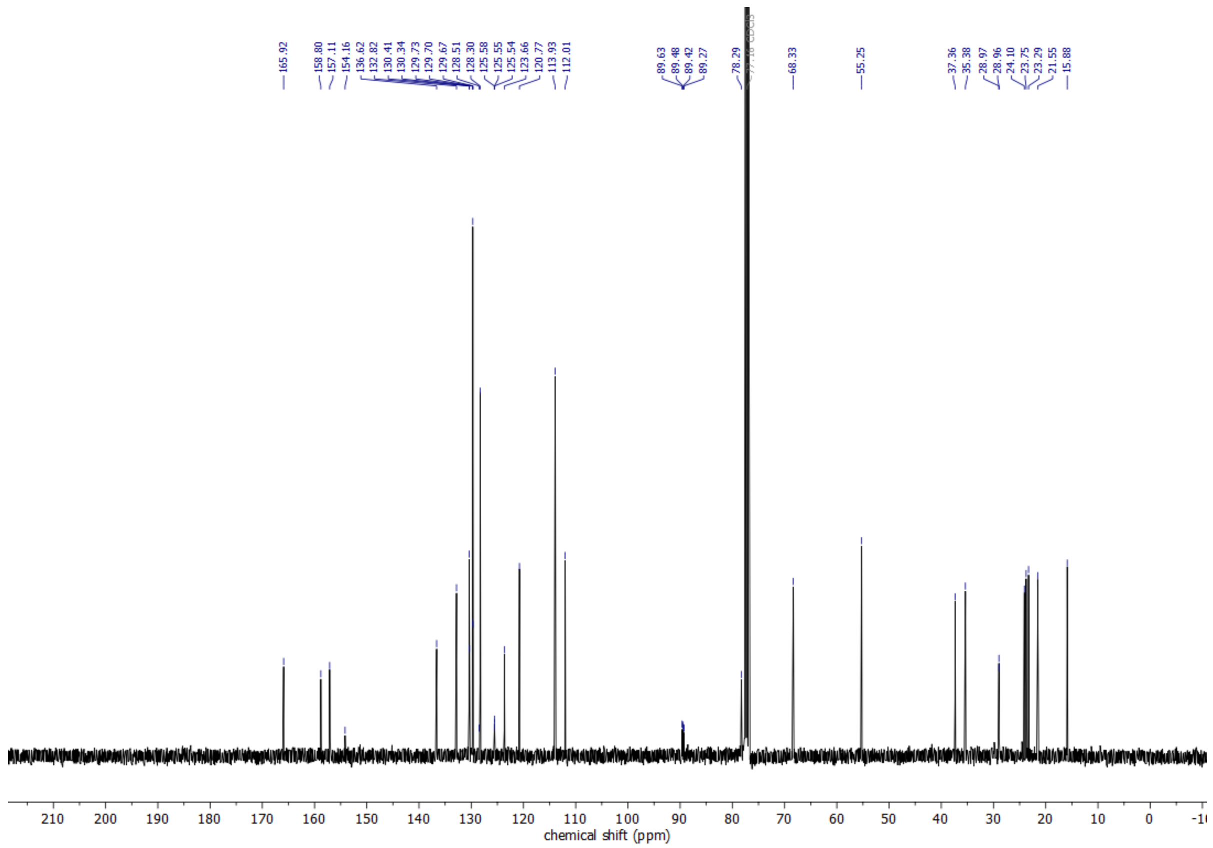

$^{19}F\{^1H\}$ NMR (377 MHz, $CDCl_3$)

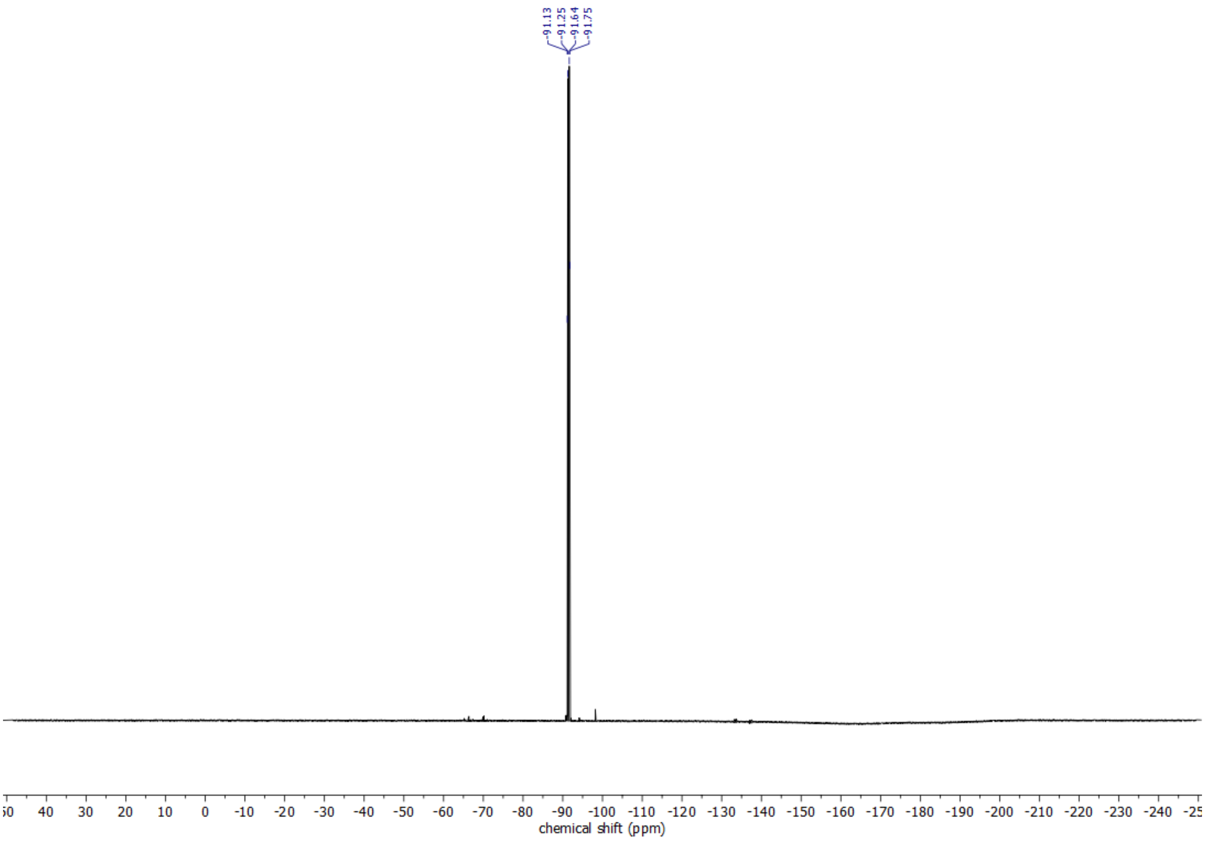

^{1}H NMR (400 MHz, $CDCl_3$)

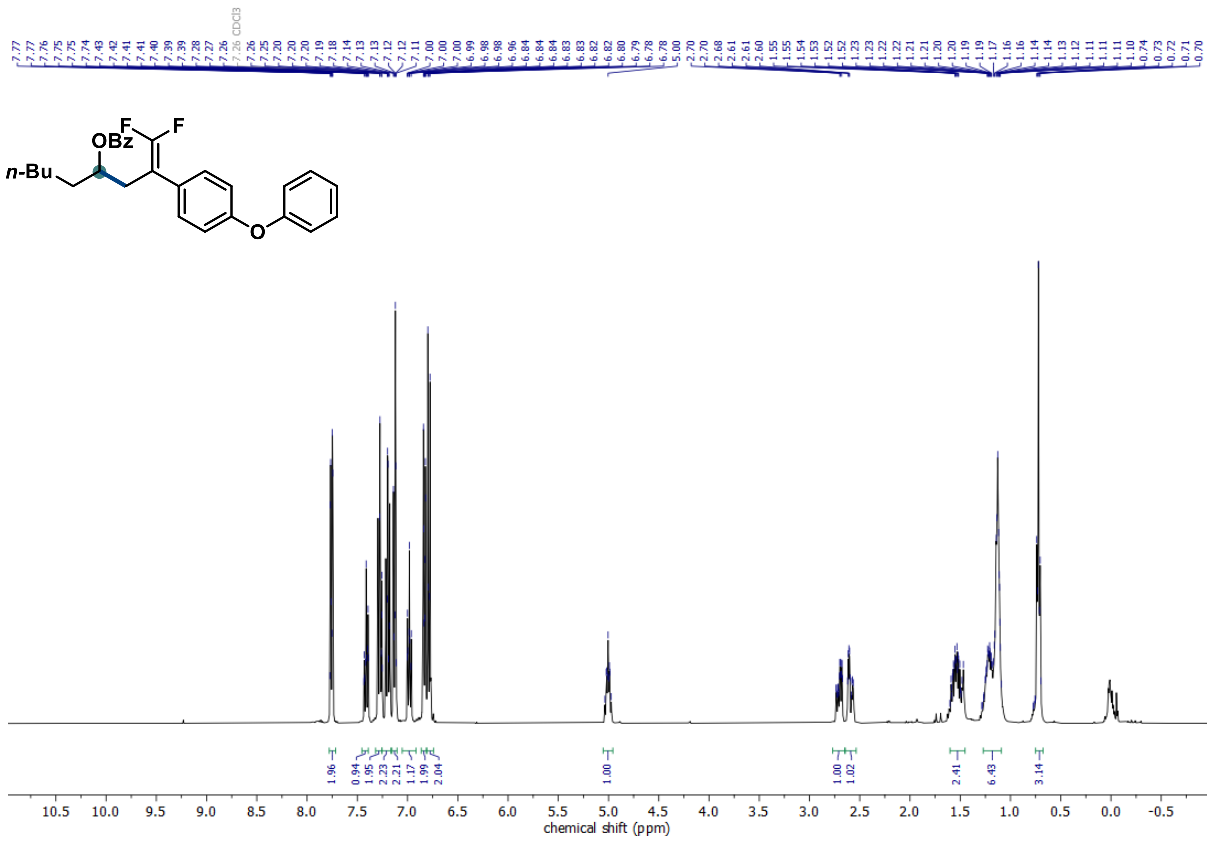

$^{13}C\{^{1}H\}$ NMR (101 MHz, $CDCl_3$)

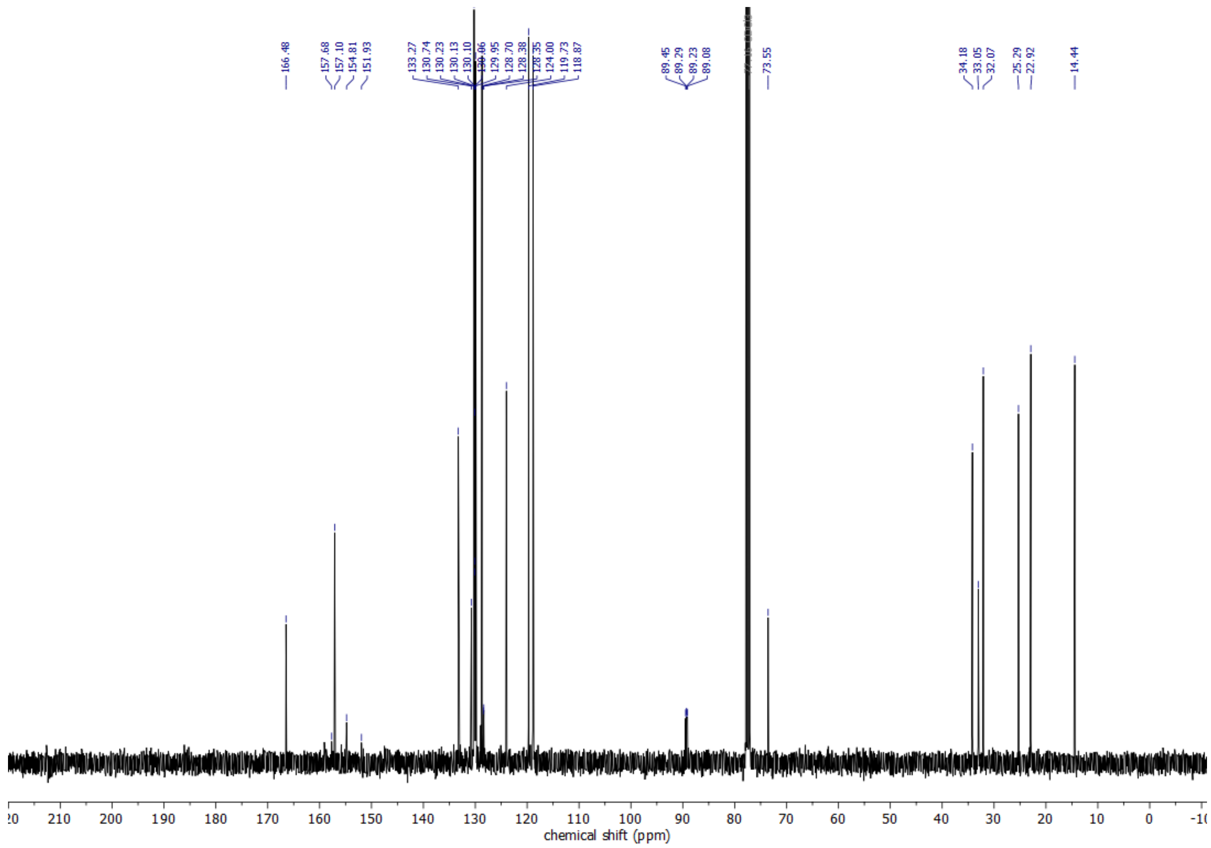

$^{19}F\{^{1}H\}$ NMR (377 MHz, $CDCl_3$)

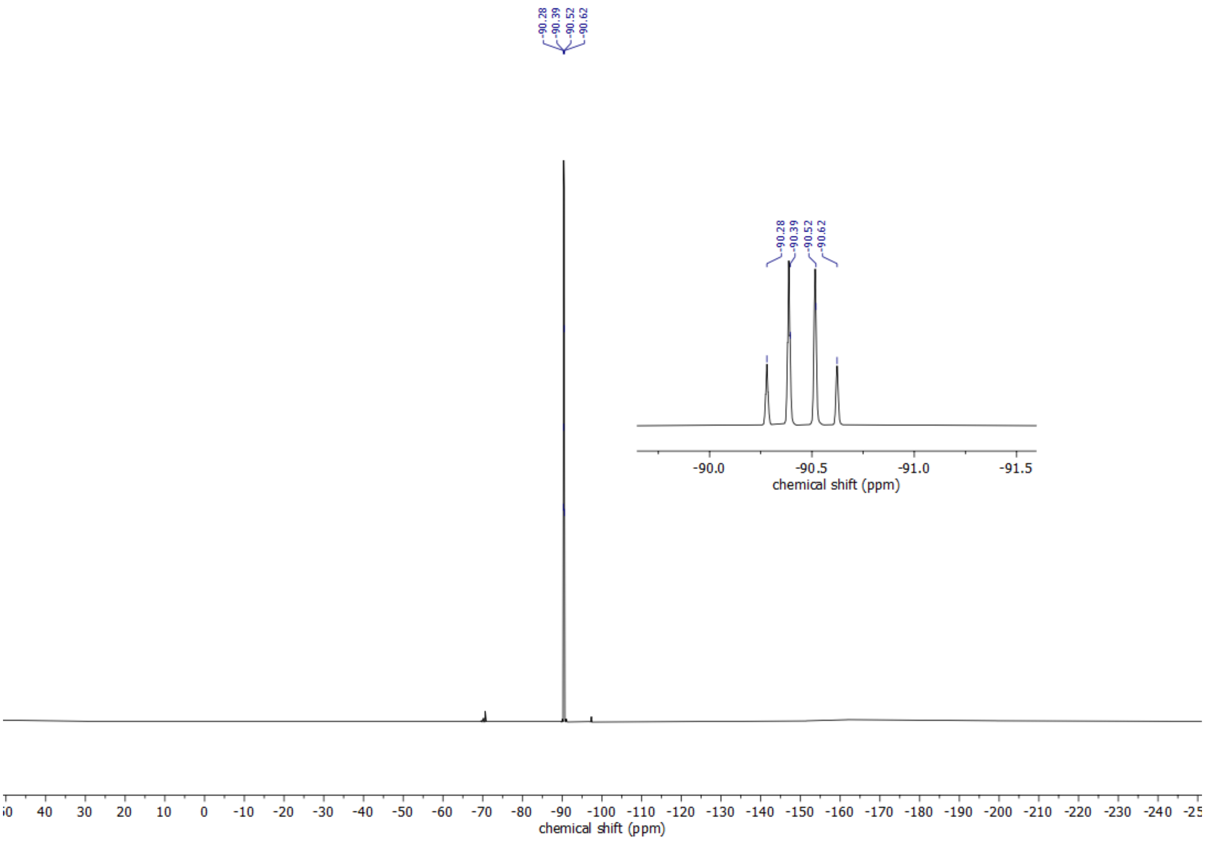

^{1}H NMR (599 MHz, $CDCl_3$)

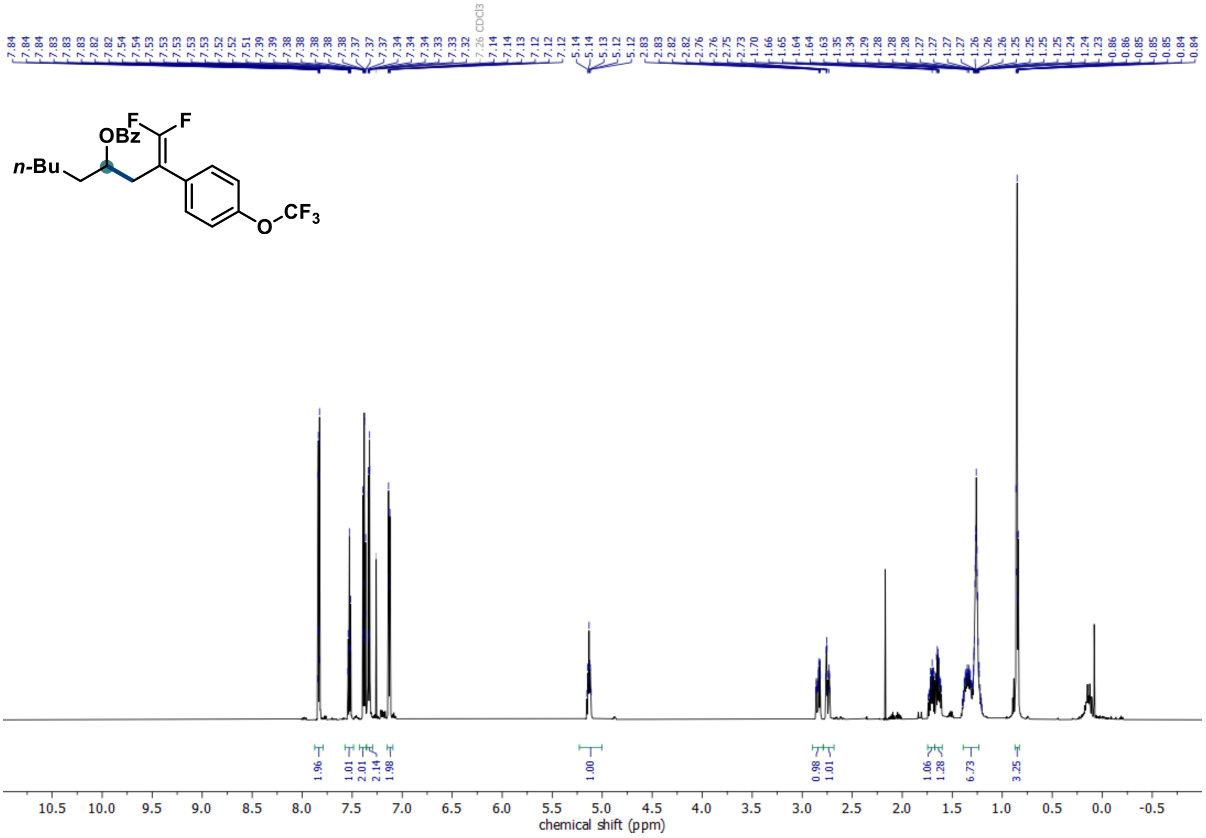

$^{13}C\{^{1}H\}$ NMR (151 MHz, $CDCl_3$)

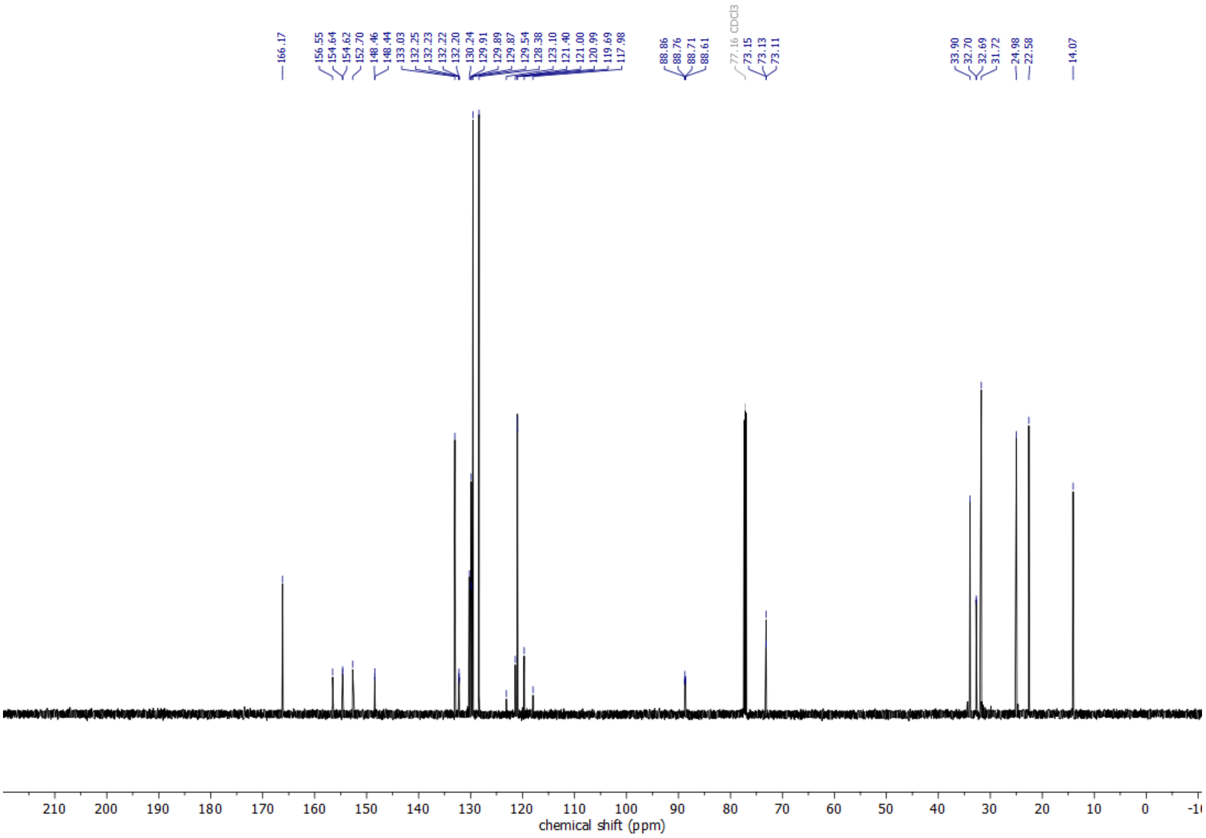

$^{13}C\{^{1}H, ^{19}F\}$ NMR (151 MHz, $CDCl_3$)

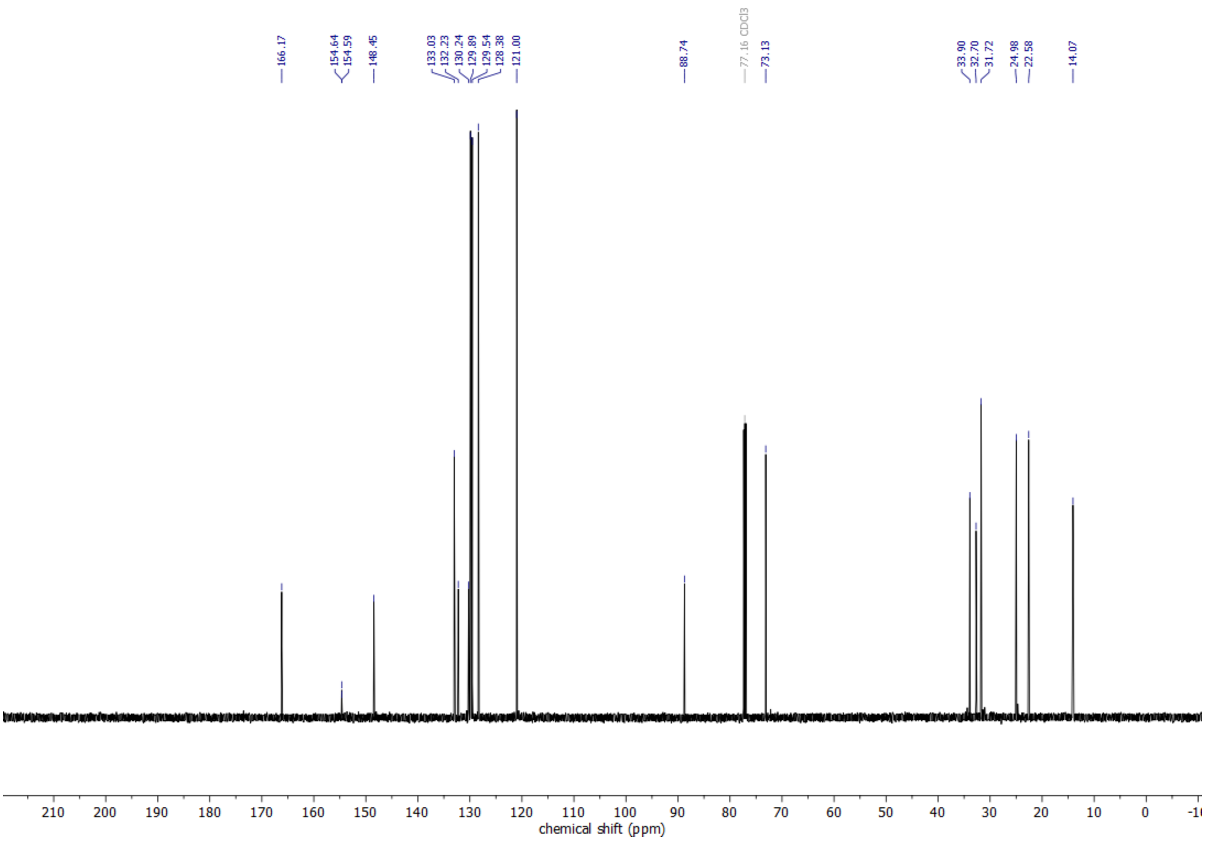

^{19}F NMR (564 MHz, $CDCl_3$)

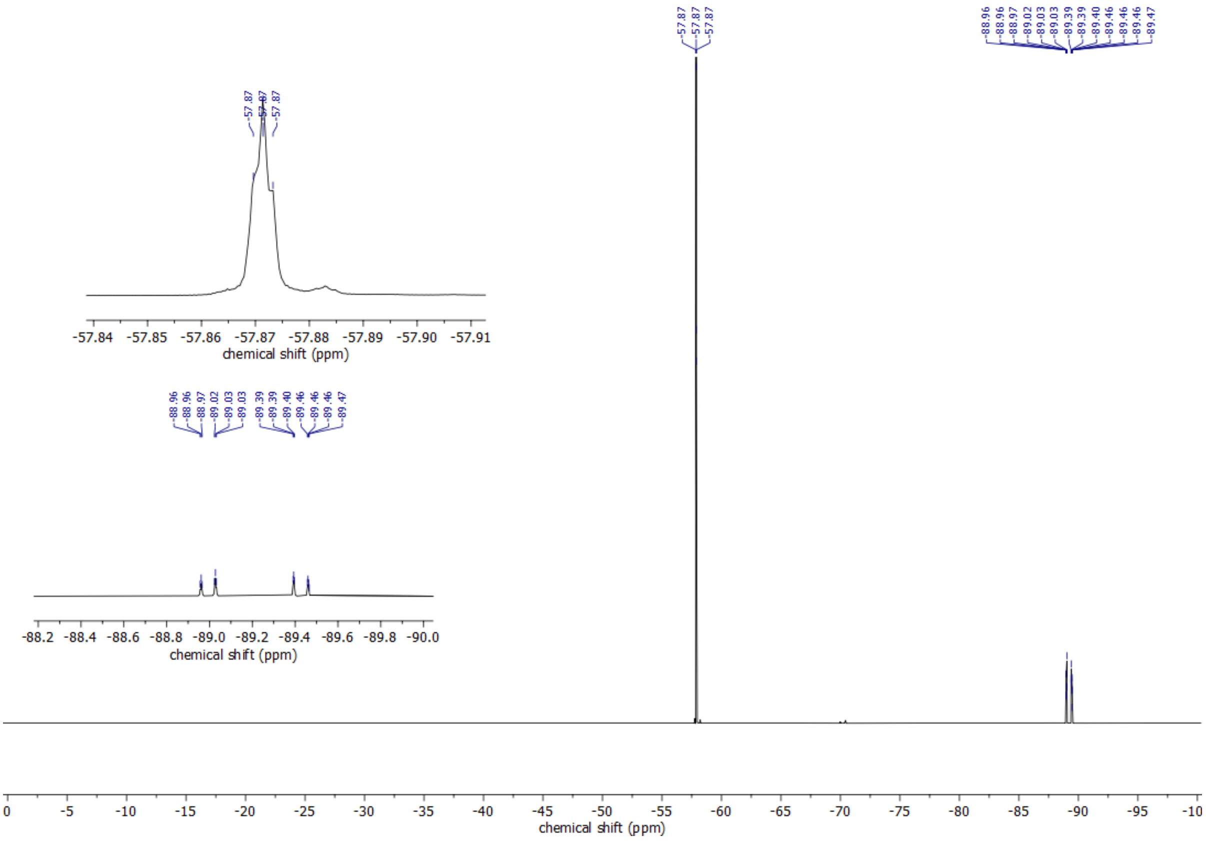

$^{19}F\{^{1}H\}$ NMR (564 MHz, $CDCl_3$)

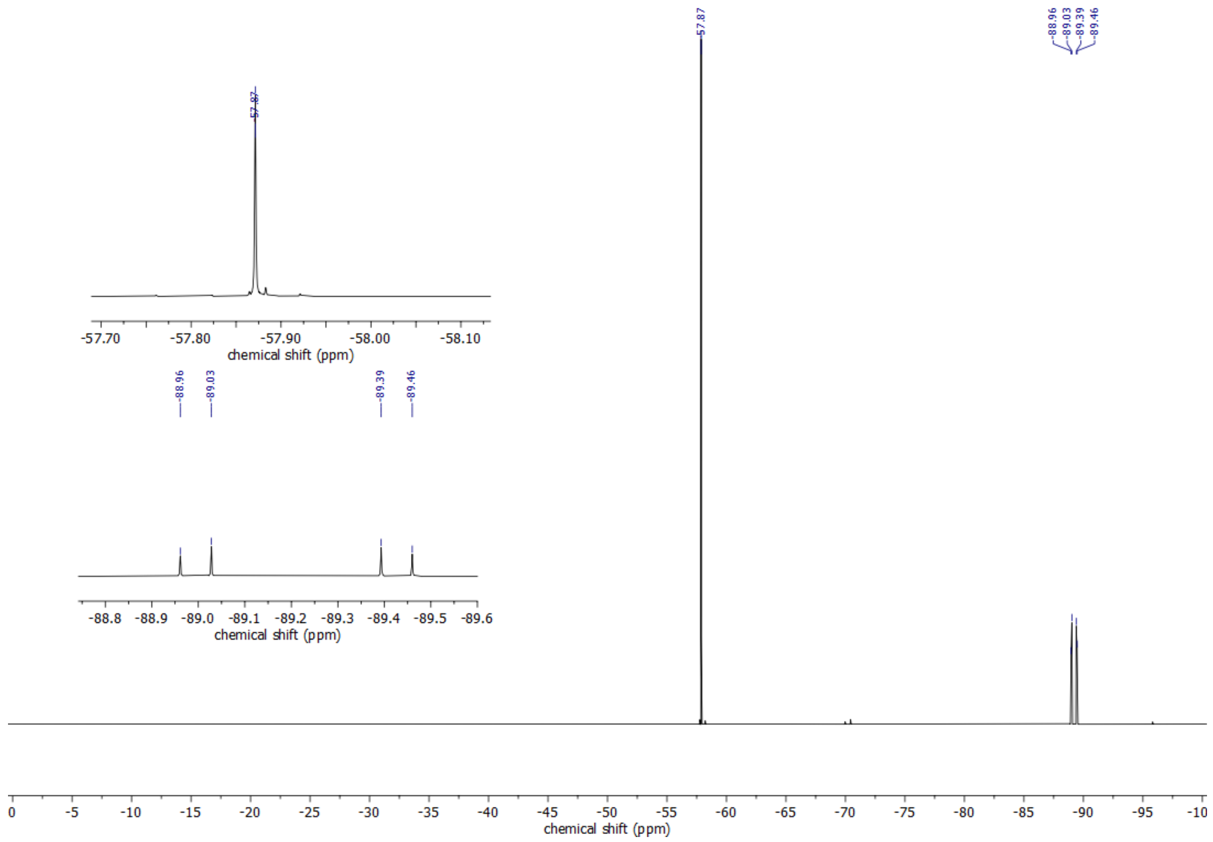

^{1}H NMR (599 MHz, $CDCl_3$)

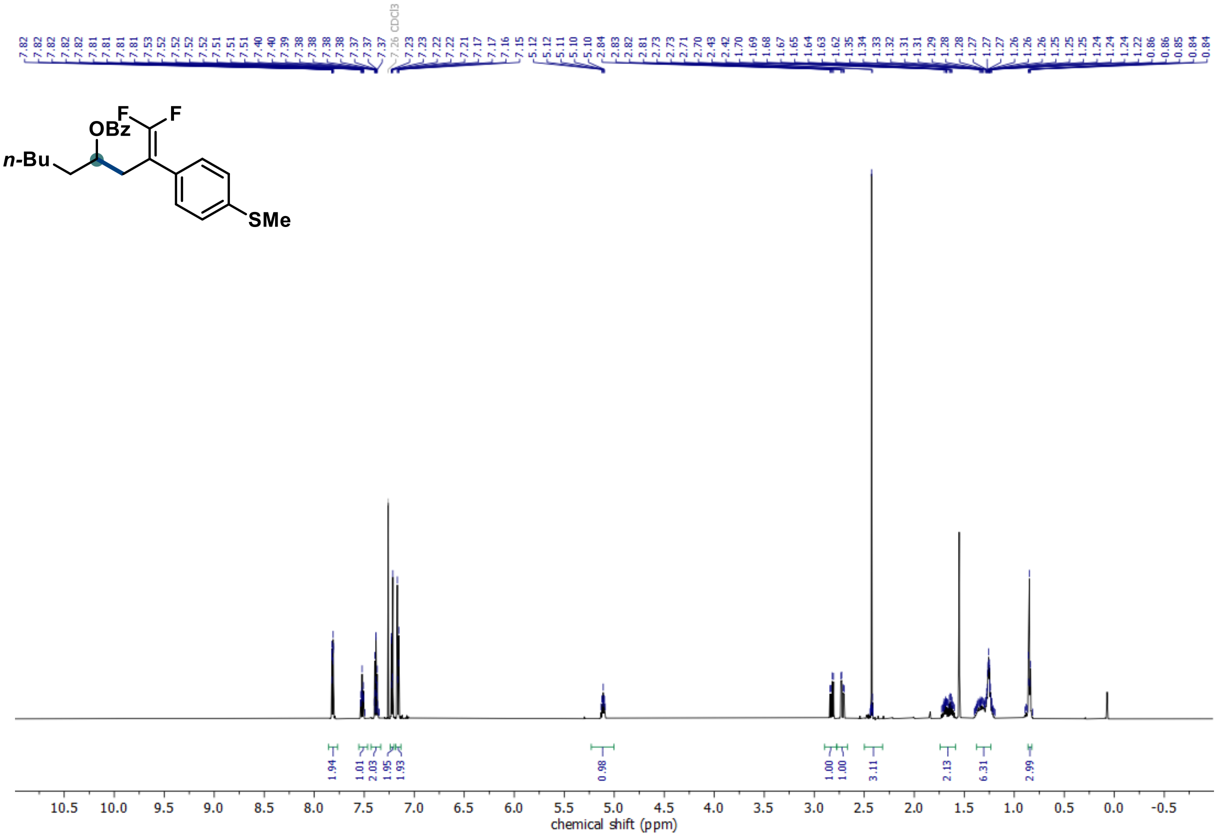

$^{13}C\{^{1}H, ^{19}F\}$ NMR (151 MHz, $CDCl_3$)

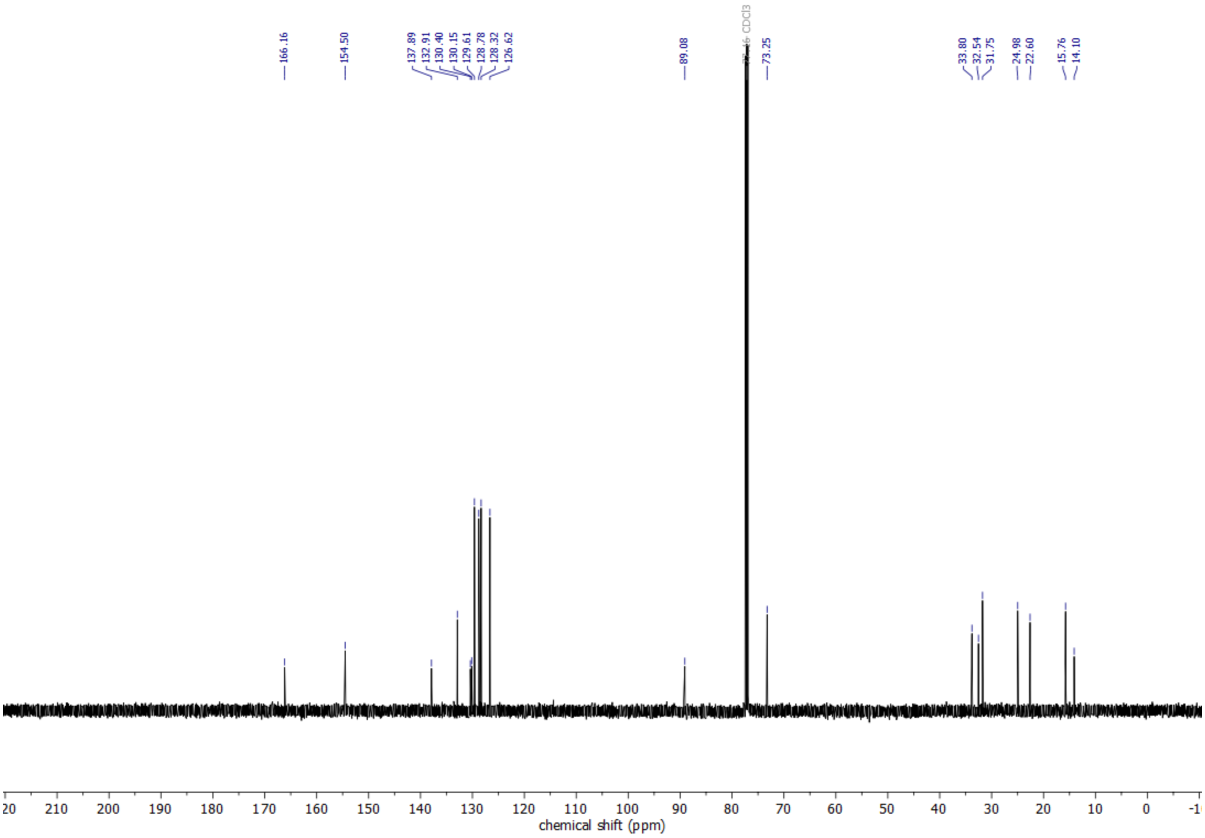

$^{19}F\{^1H\}$ NMR (564 MHz, $CDCl_3$)

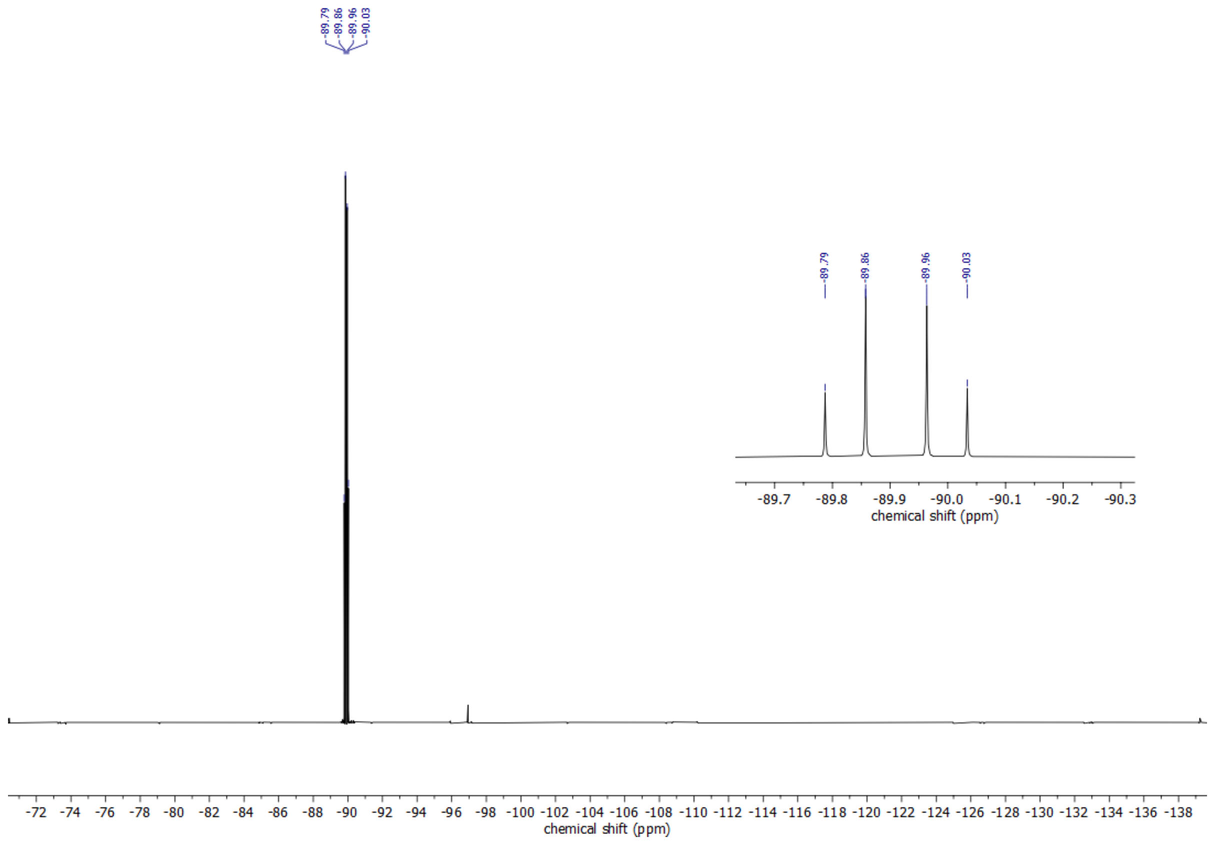

^{1}H NMR (500 MHz, $CDCl_3$)

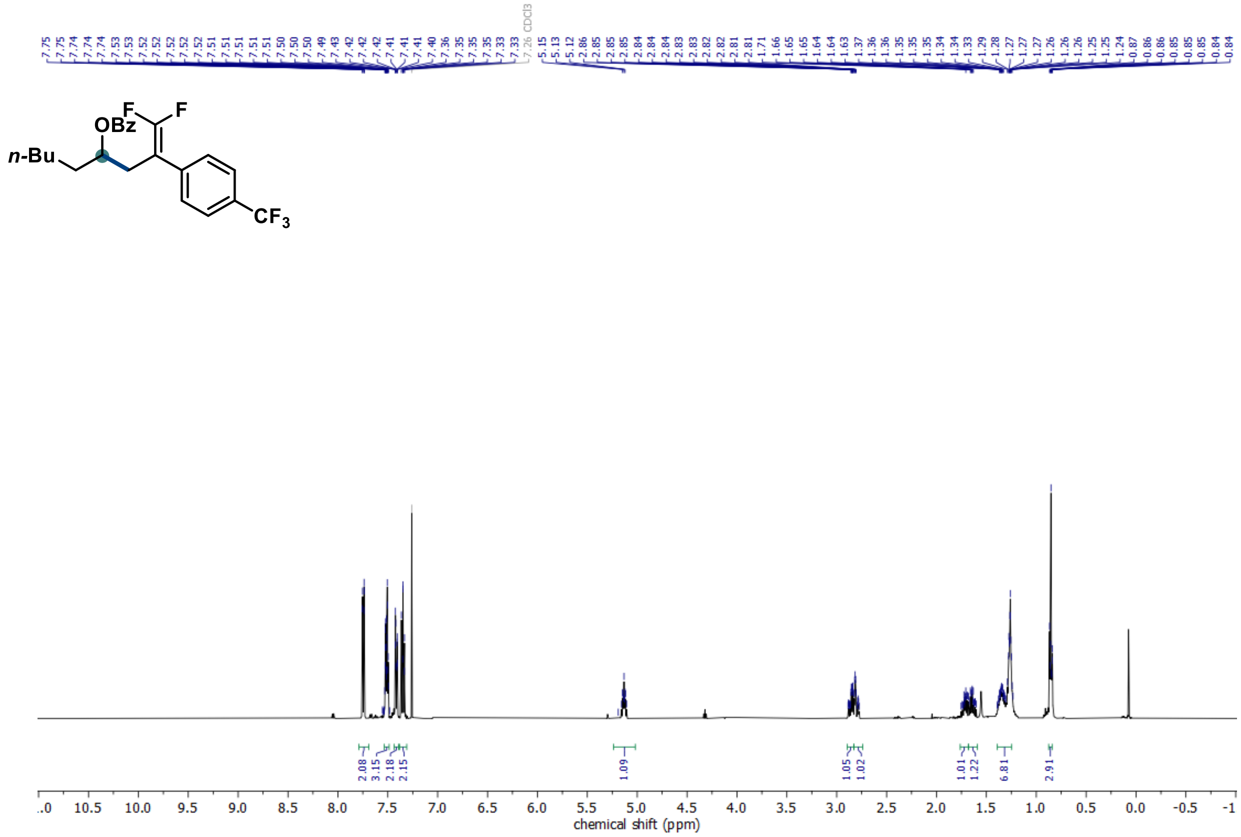

$^{13}C\{^{1}H, ^{19}F\}$ NMR (126 MHz, $CDCl_3$)

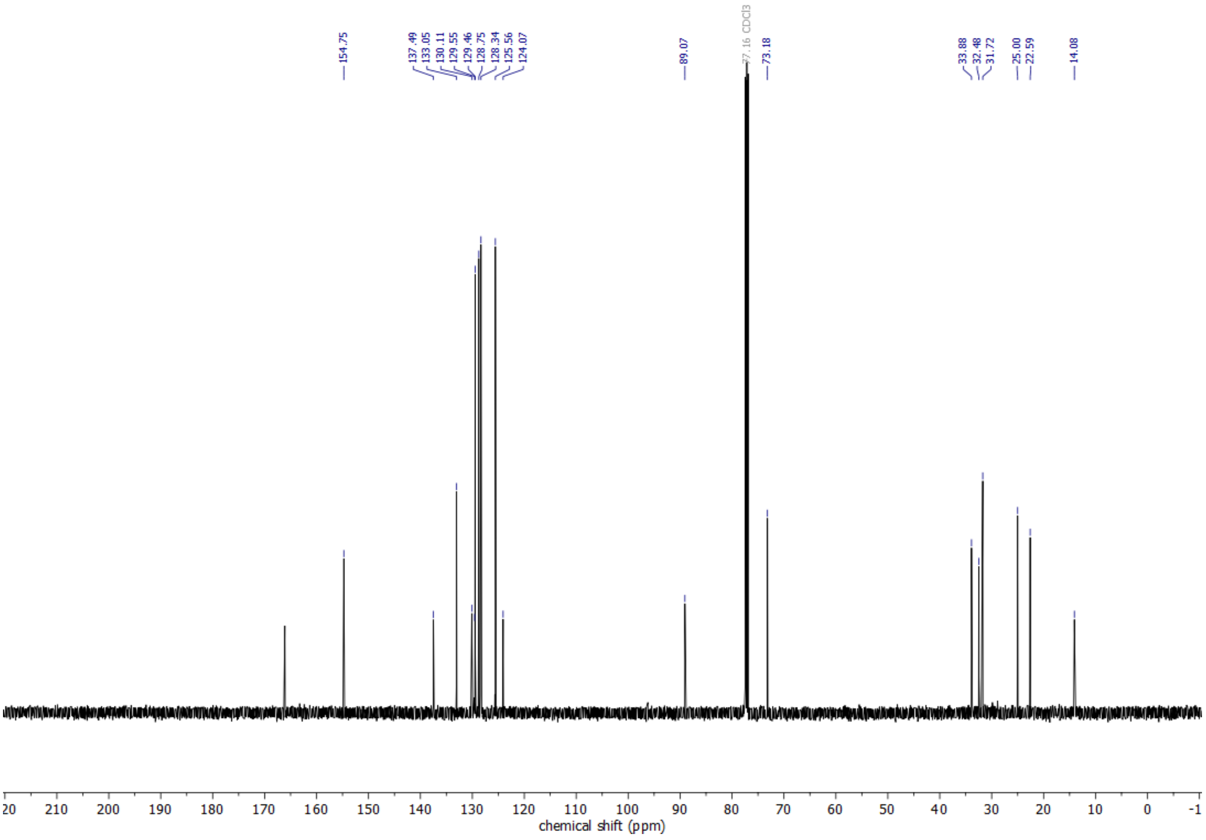

$^{19}F\{^{1}H\}$ NMR (564 MHz, $CDCl_3$)

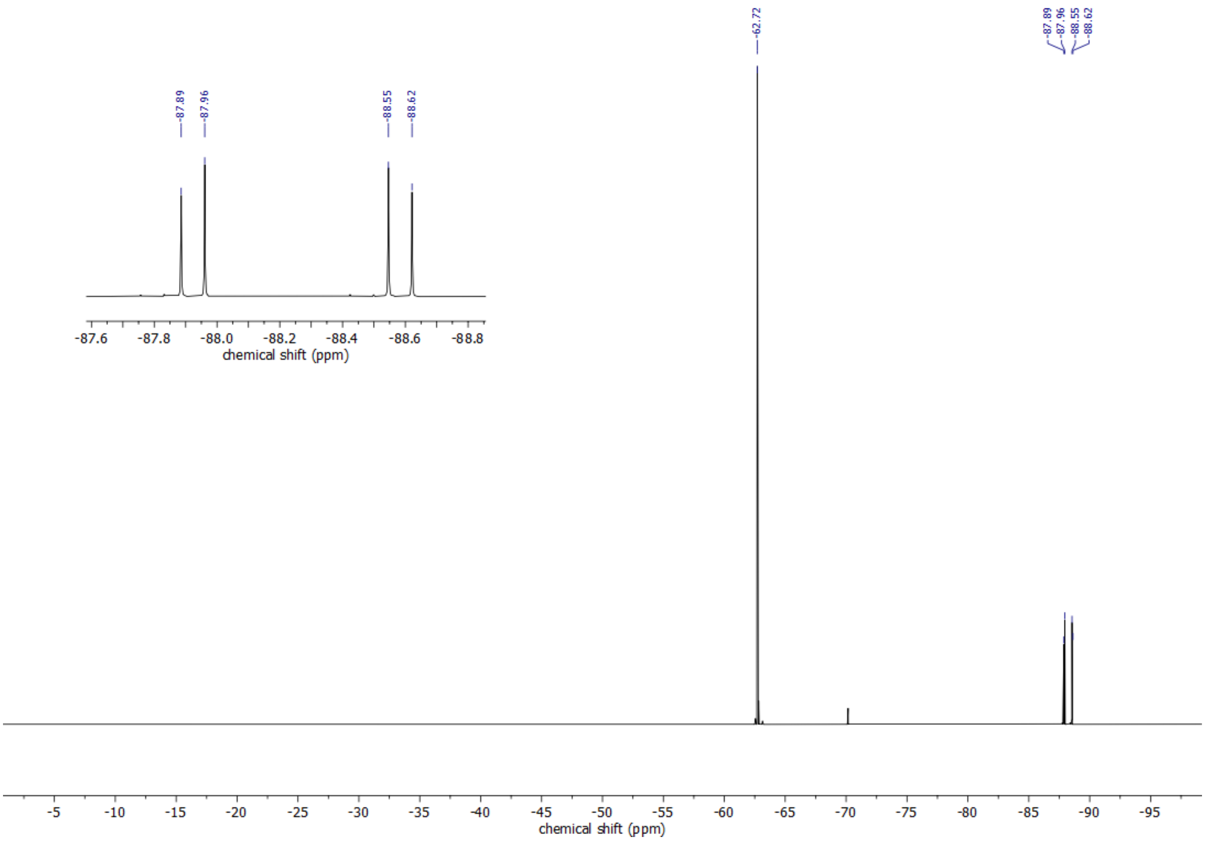

$^{1}H\{^{19}F\}$ NMR (500 MHz, $CDCl_3$)

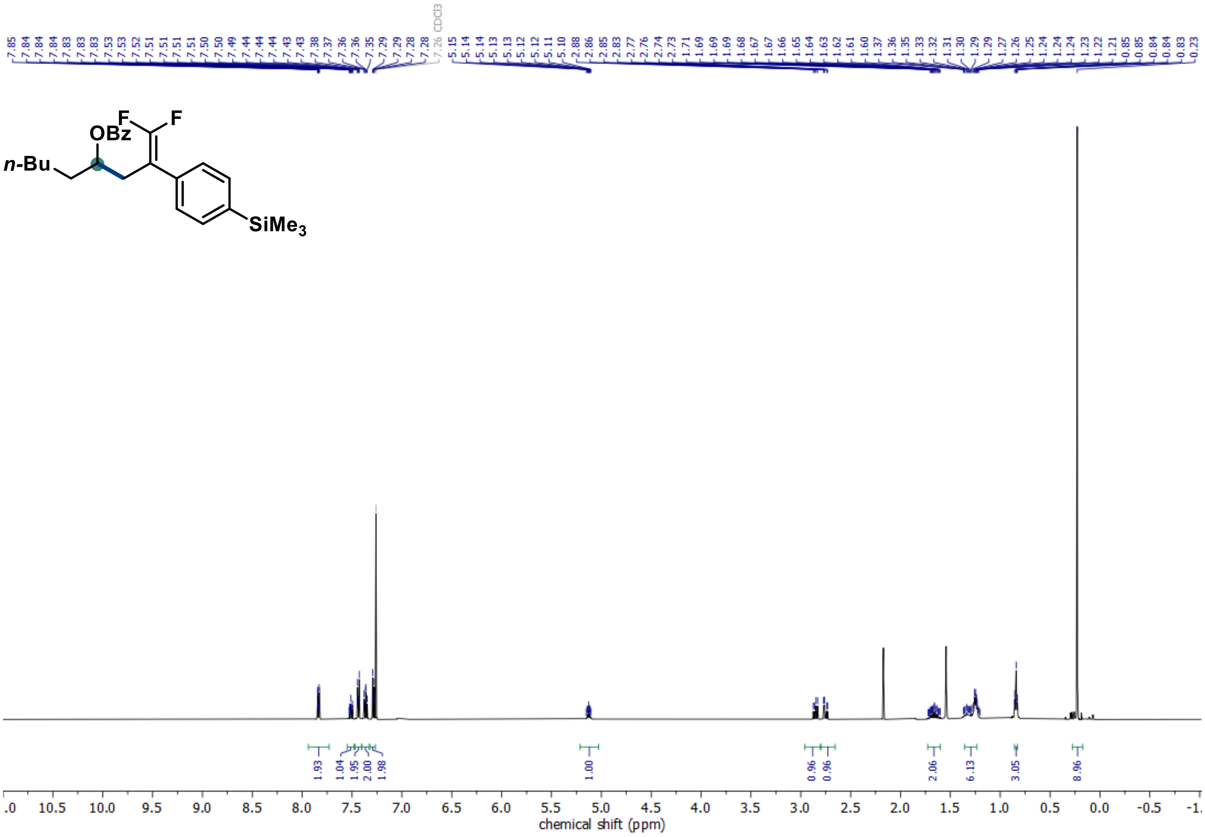

^{1}H NMR (500 MHz, $CDCl_3$)

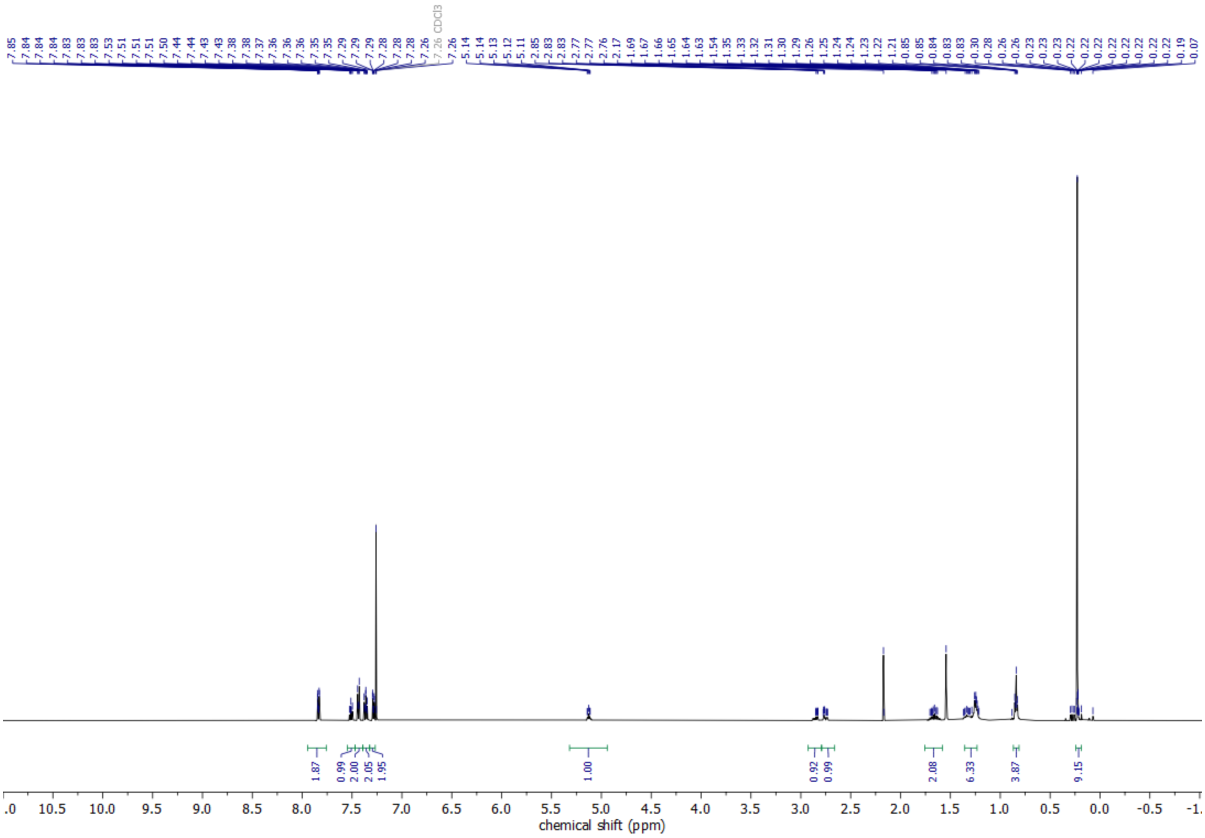

$^{13}C\{^1H, ^{19}F\}$ NMR (126 MHz, $CDCl_3$)

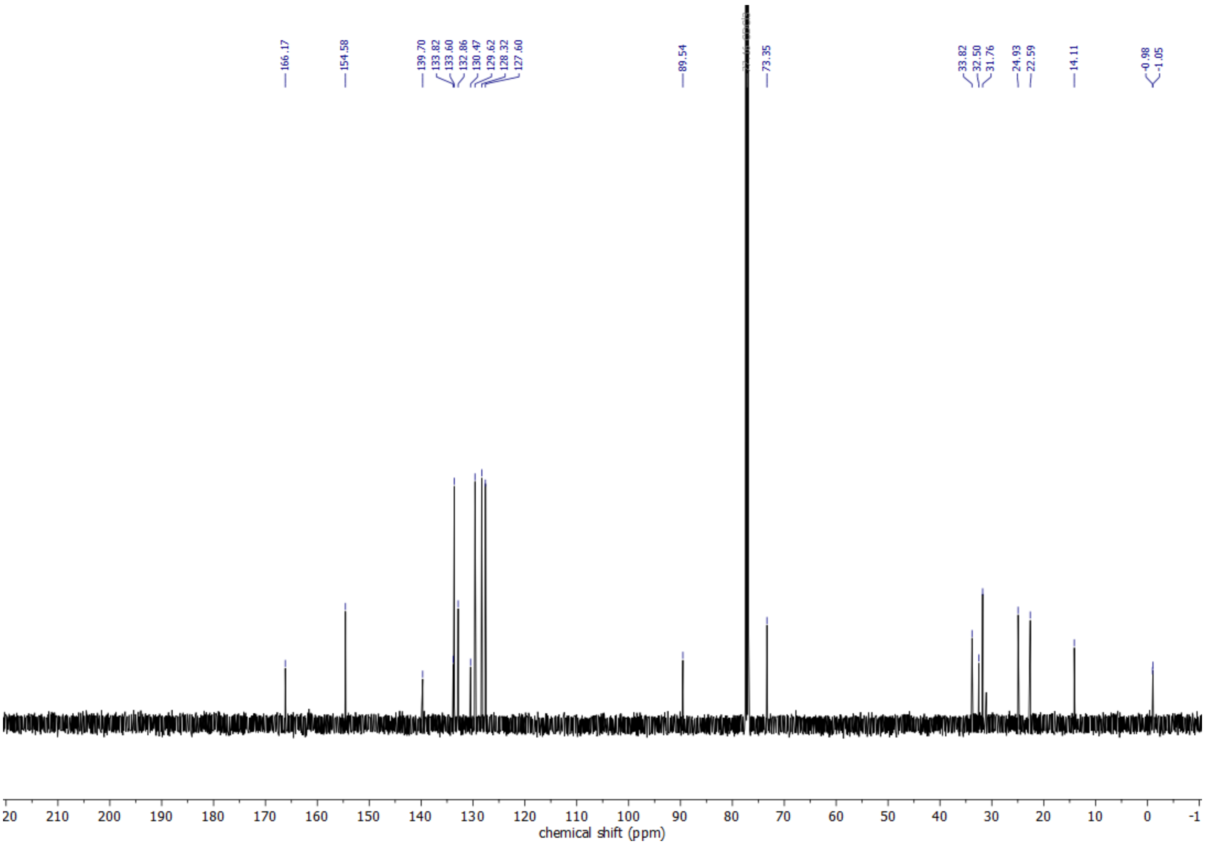

^{19}F NMR (564 MHz, $CDCl_3$)

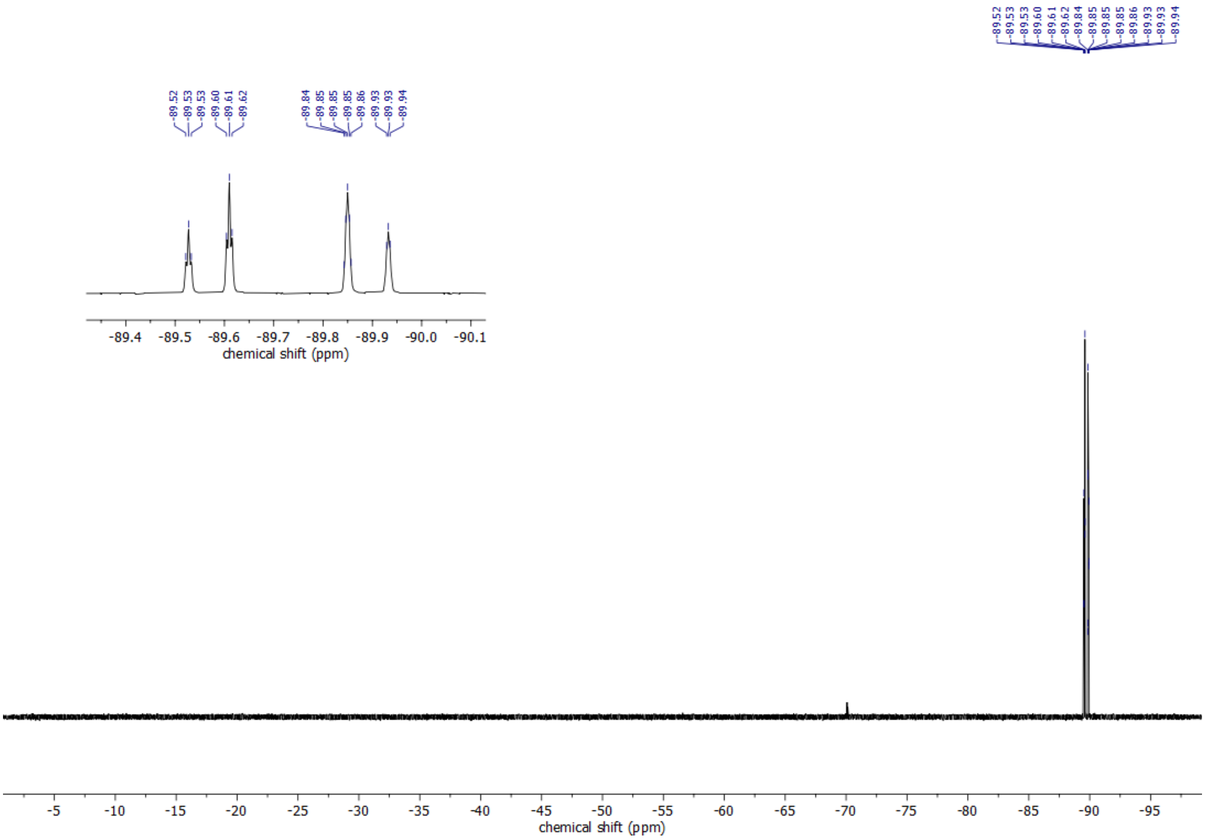

$^{19}F\{^1H\}$ NMR (564 MHz, $CDCl_3$)

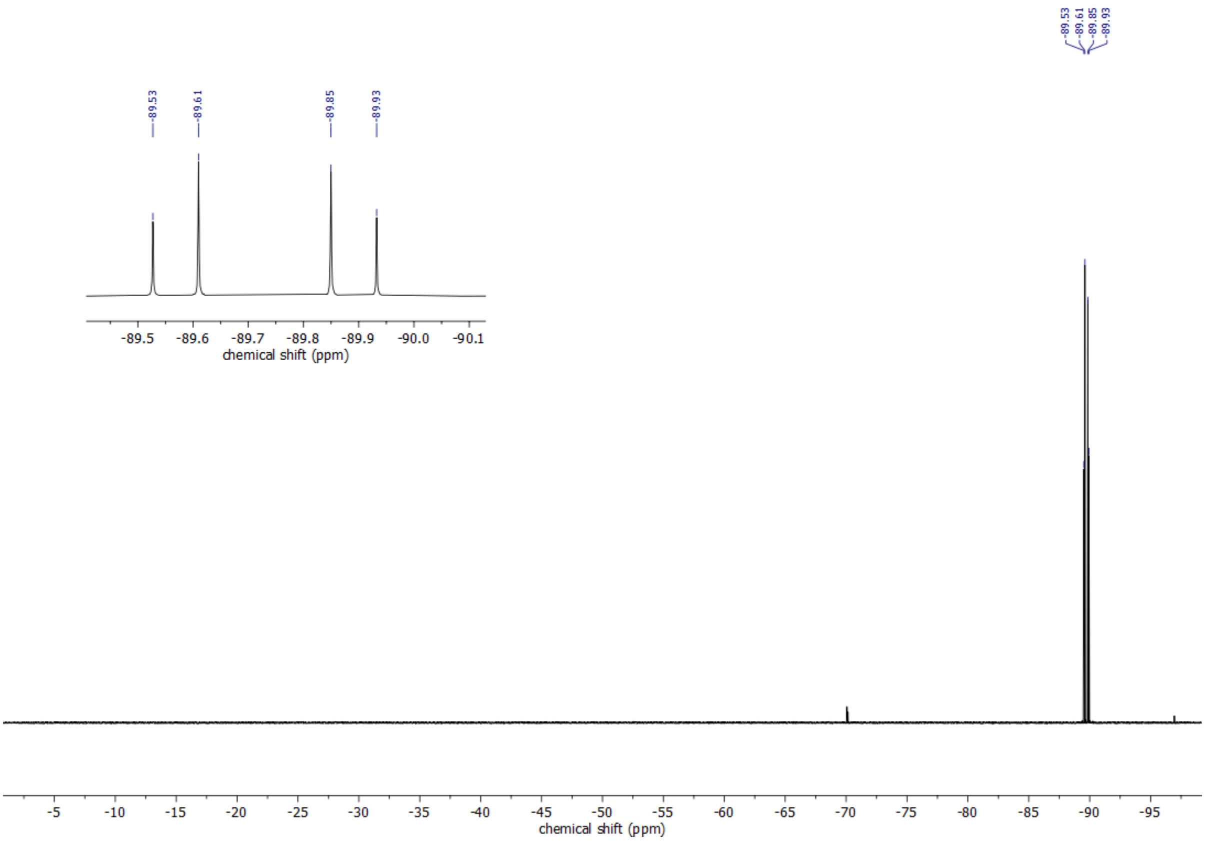

^{1}H NMR (500 MHz, $CDCl_3$)

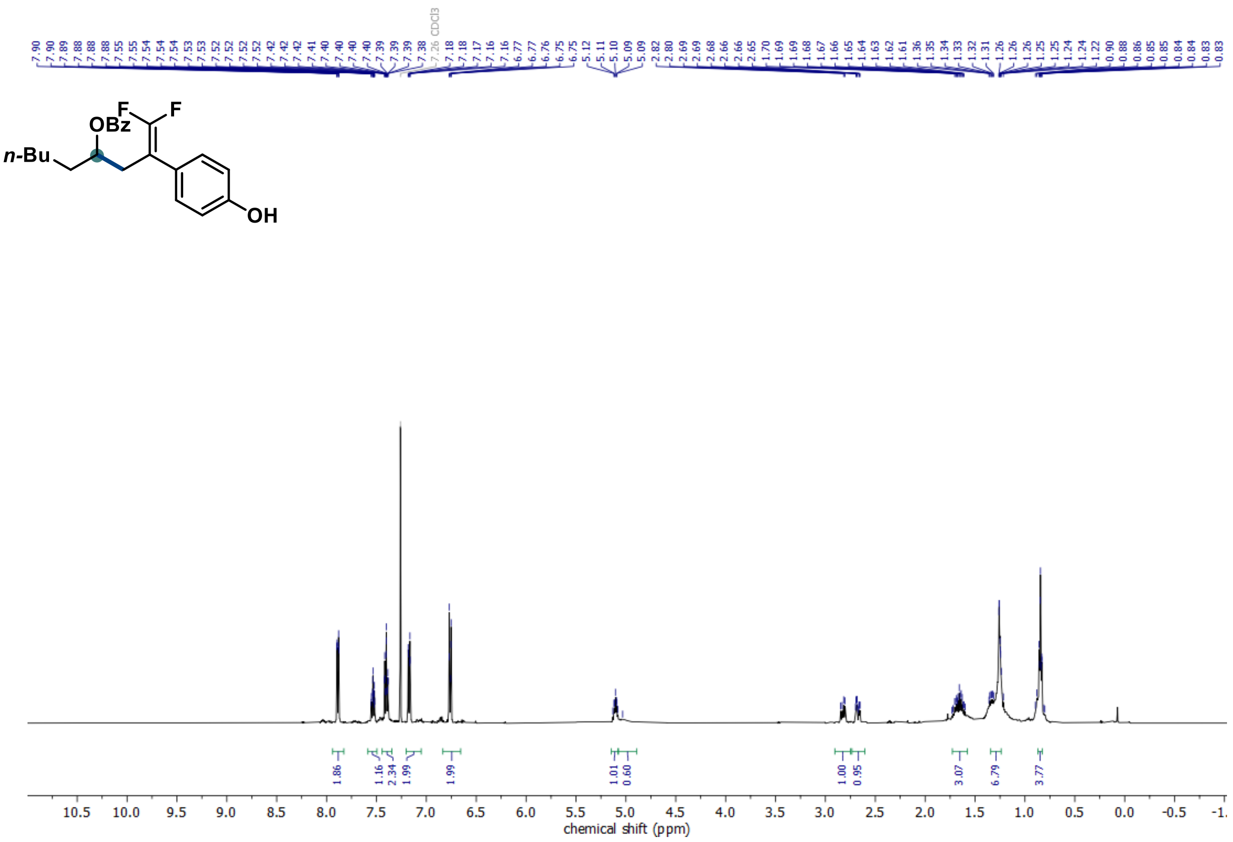

^{13}C{^{1}H, ^{19}F} NMR (126 MHz, $CDCl_3$)

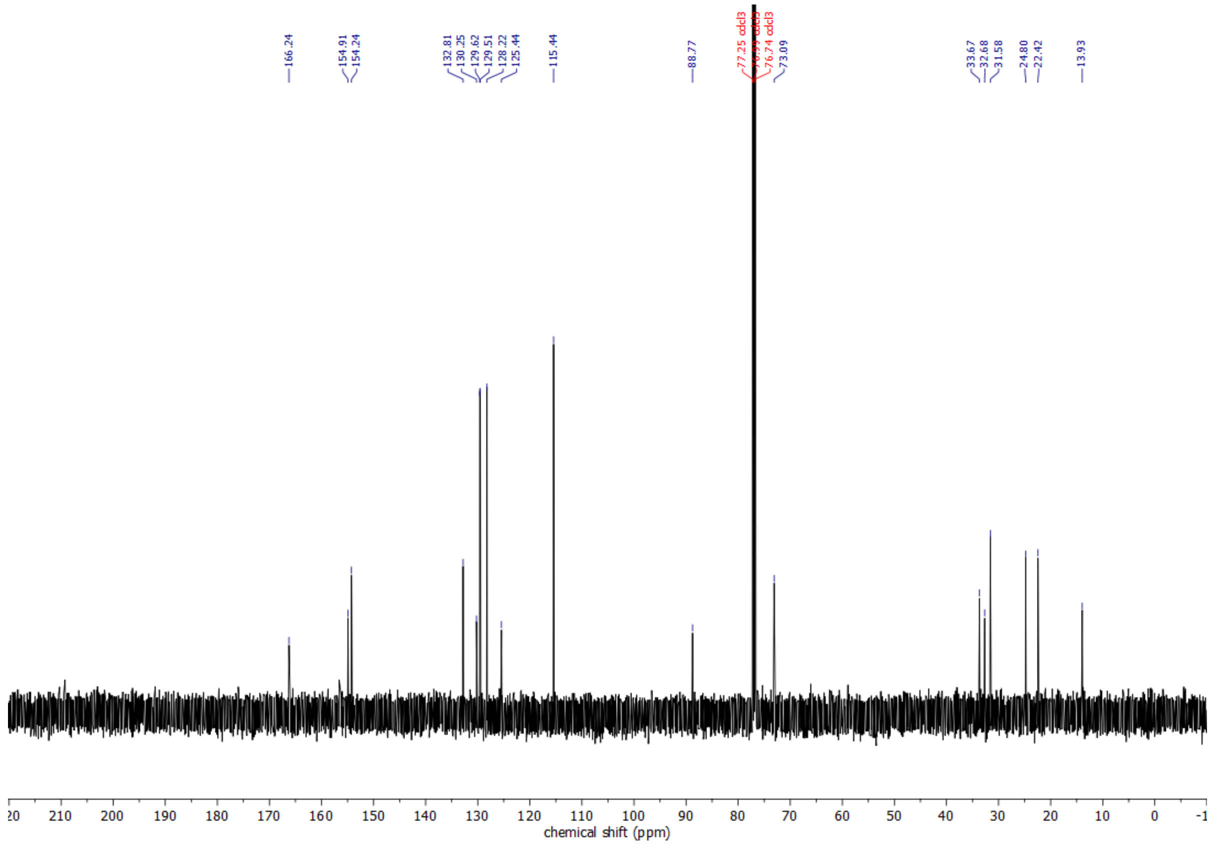

$^{19}F\{^{1}H\}$ NMR (564 MHz, $CDCl_3$)

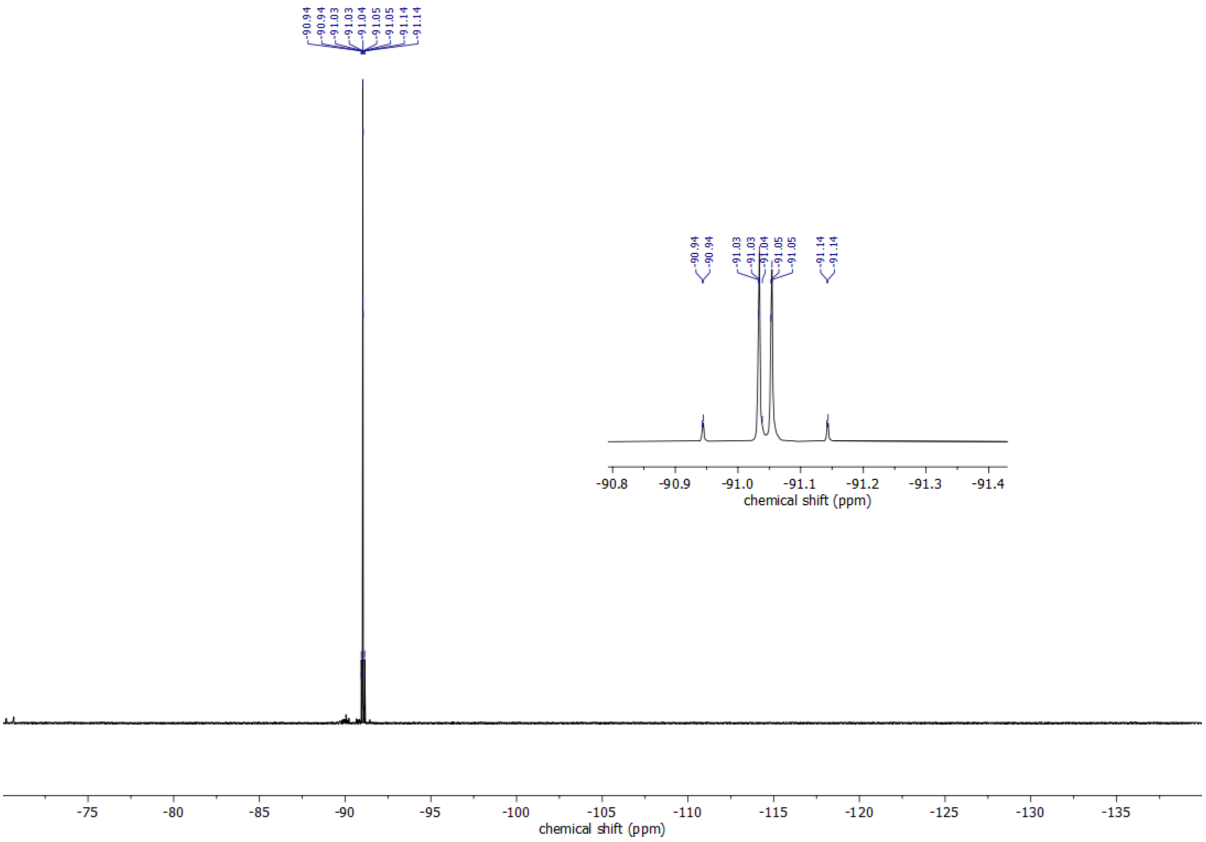

^{1}H NMR (400 MHz, CDCl$_3$)

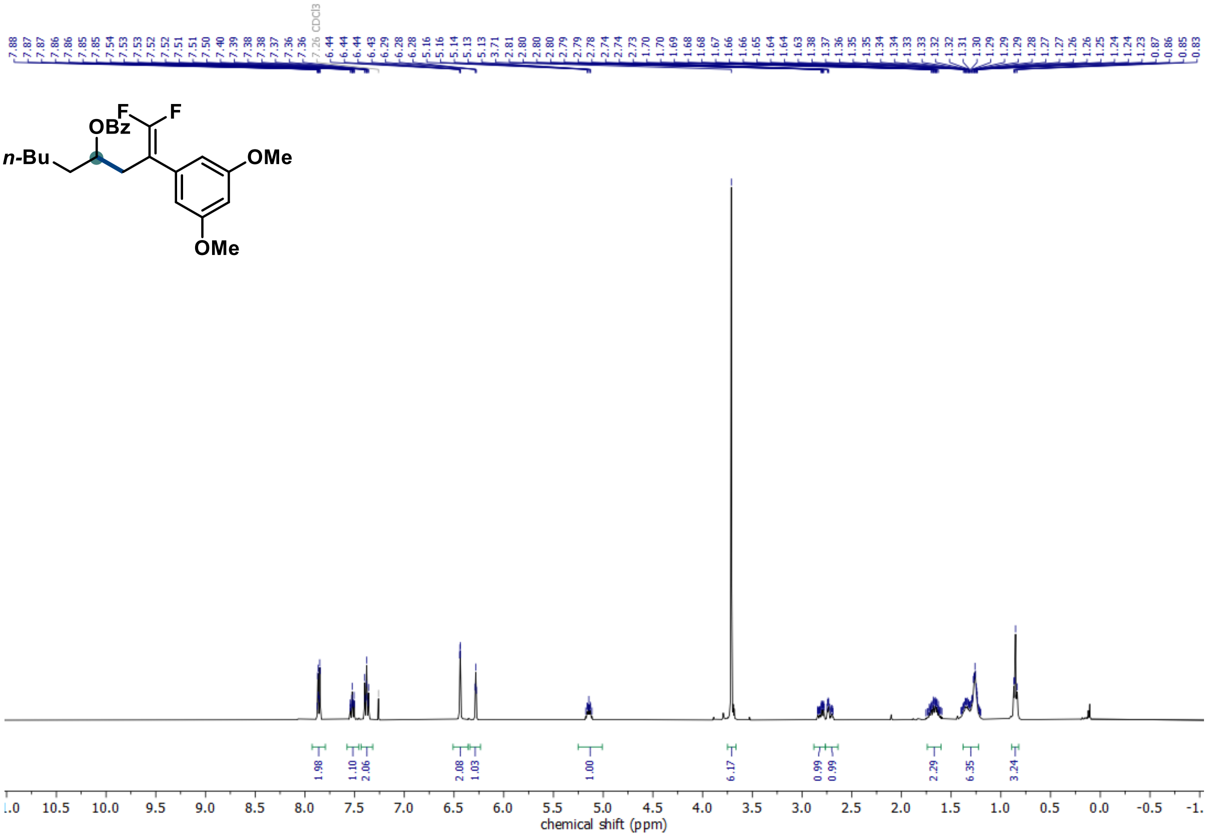

^{13}C{^{1}H} NMR (101 MHz, CDCl$_3$)

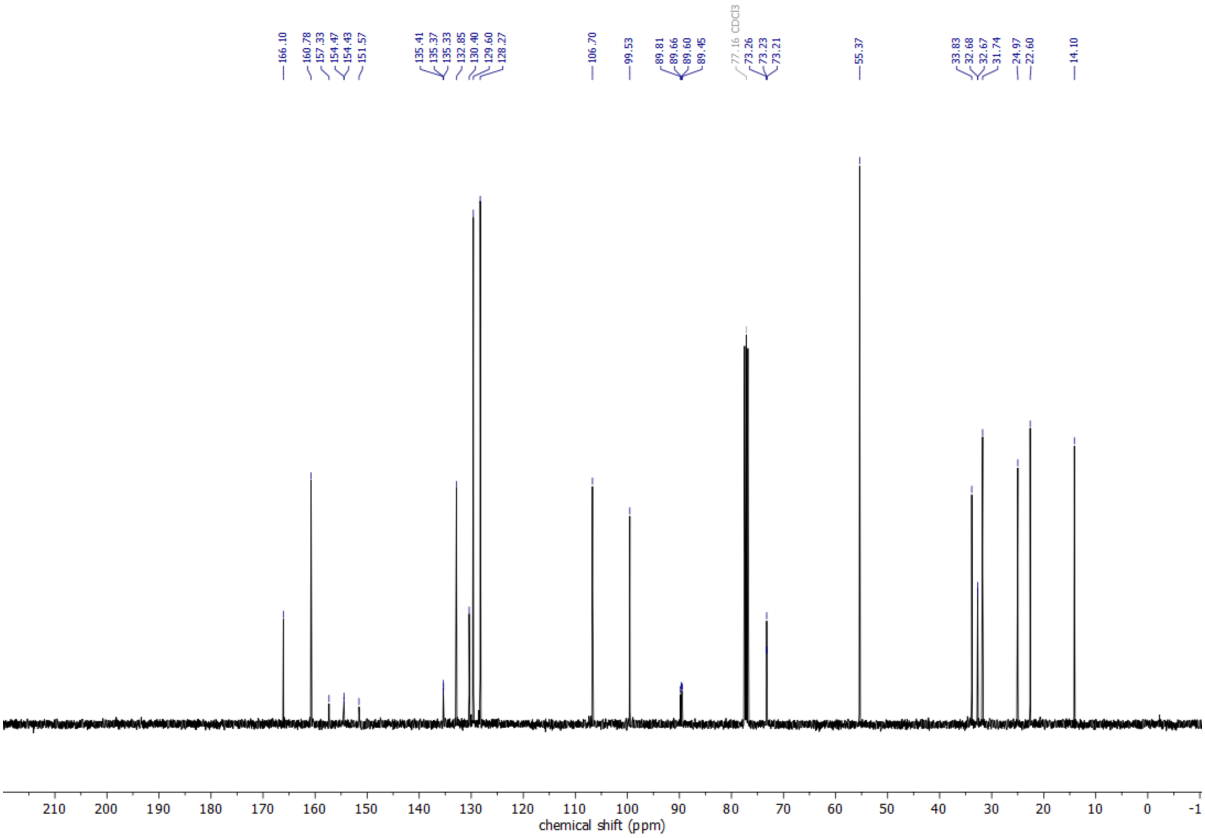

$^{19}F\{^1H\}$ NMR (377 MHz, $CDCl_3$)

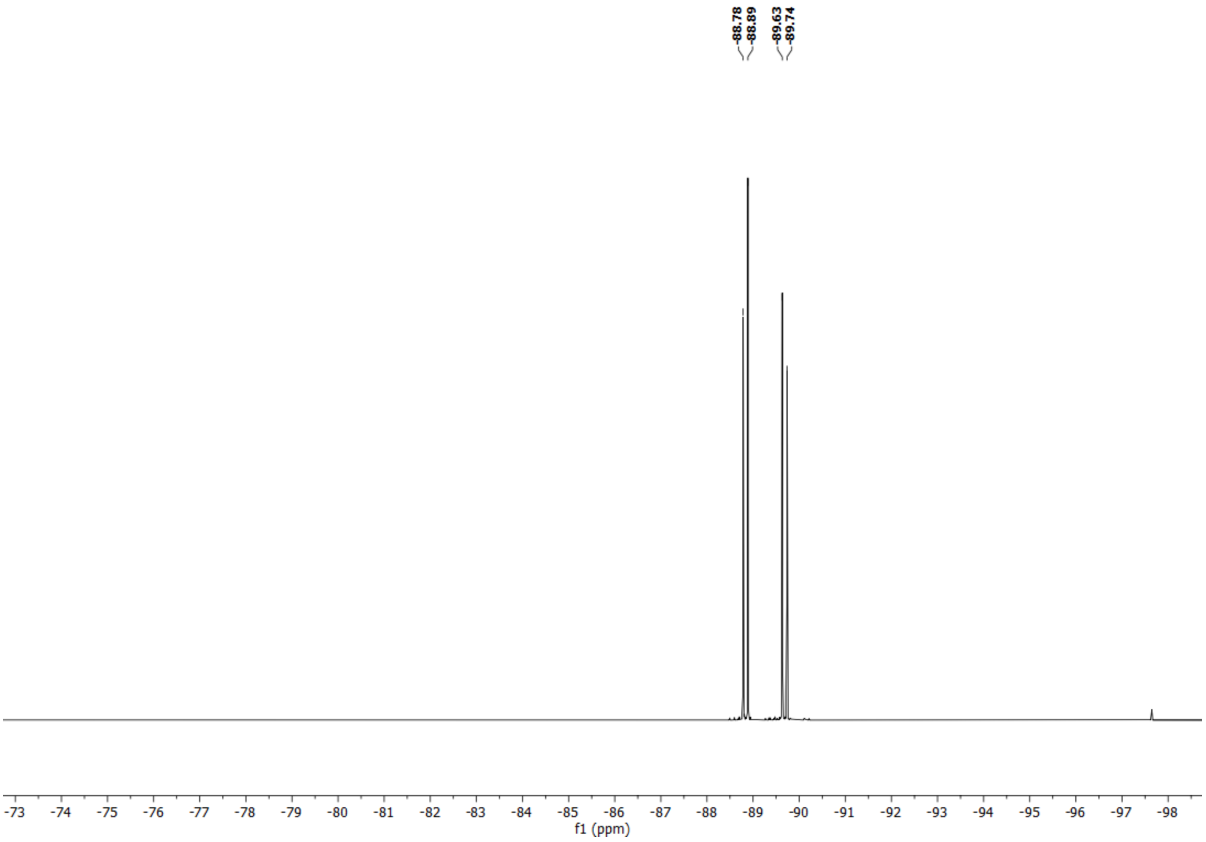

^{1}H NMR (599 MHz, $CDCl_3$)

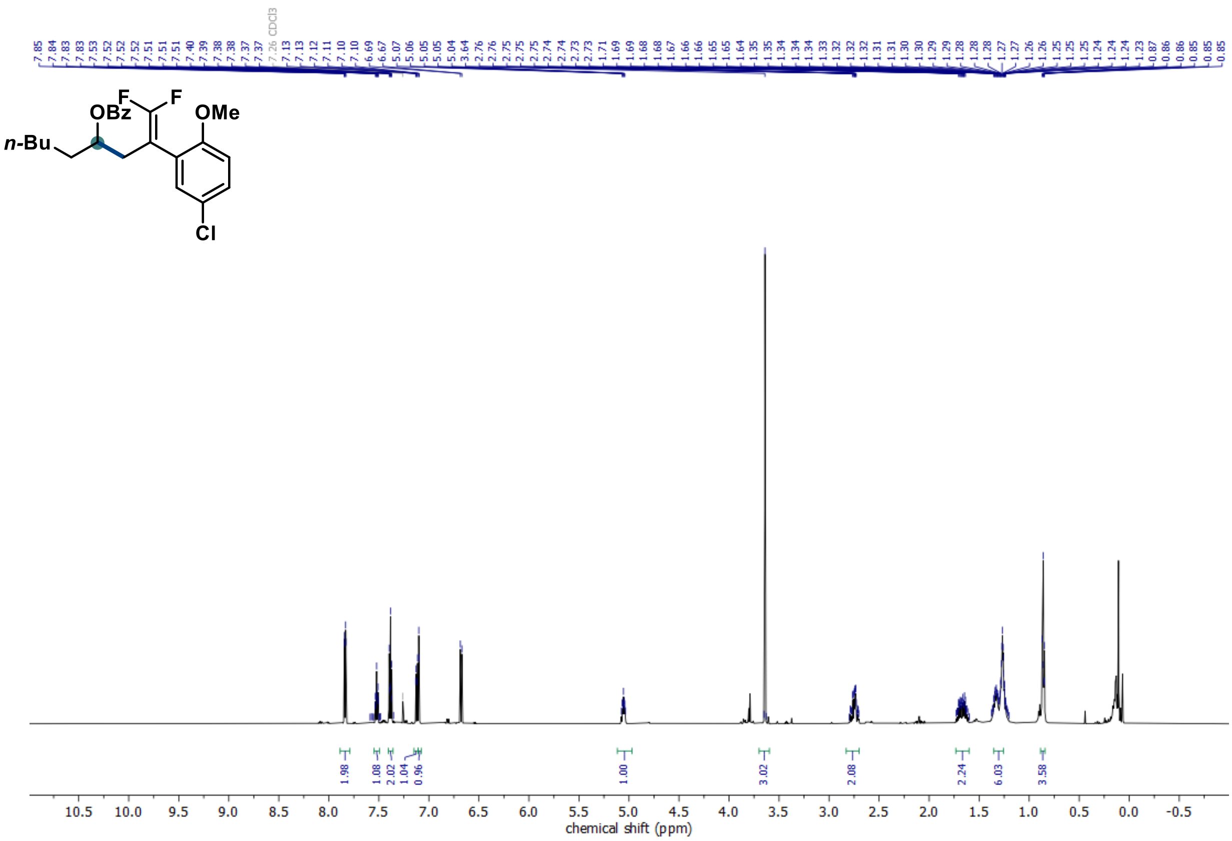

^{13}C{^{1}H} NMR (151 MHz, $CDCl_3$)

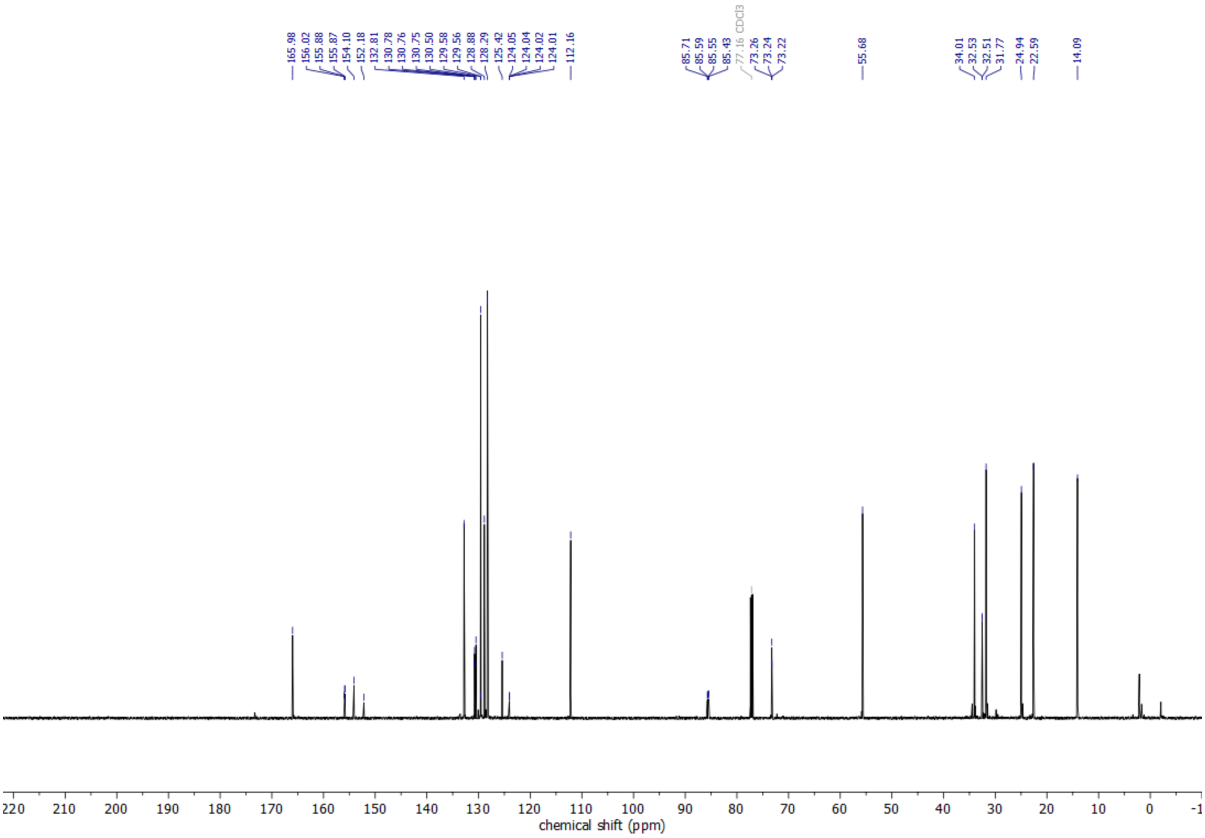

$^{19}F\{^{1}H\}$ NMR (564 MHz, $CDCl_3$)

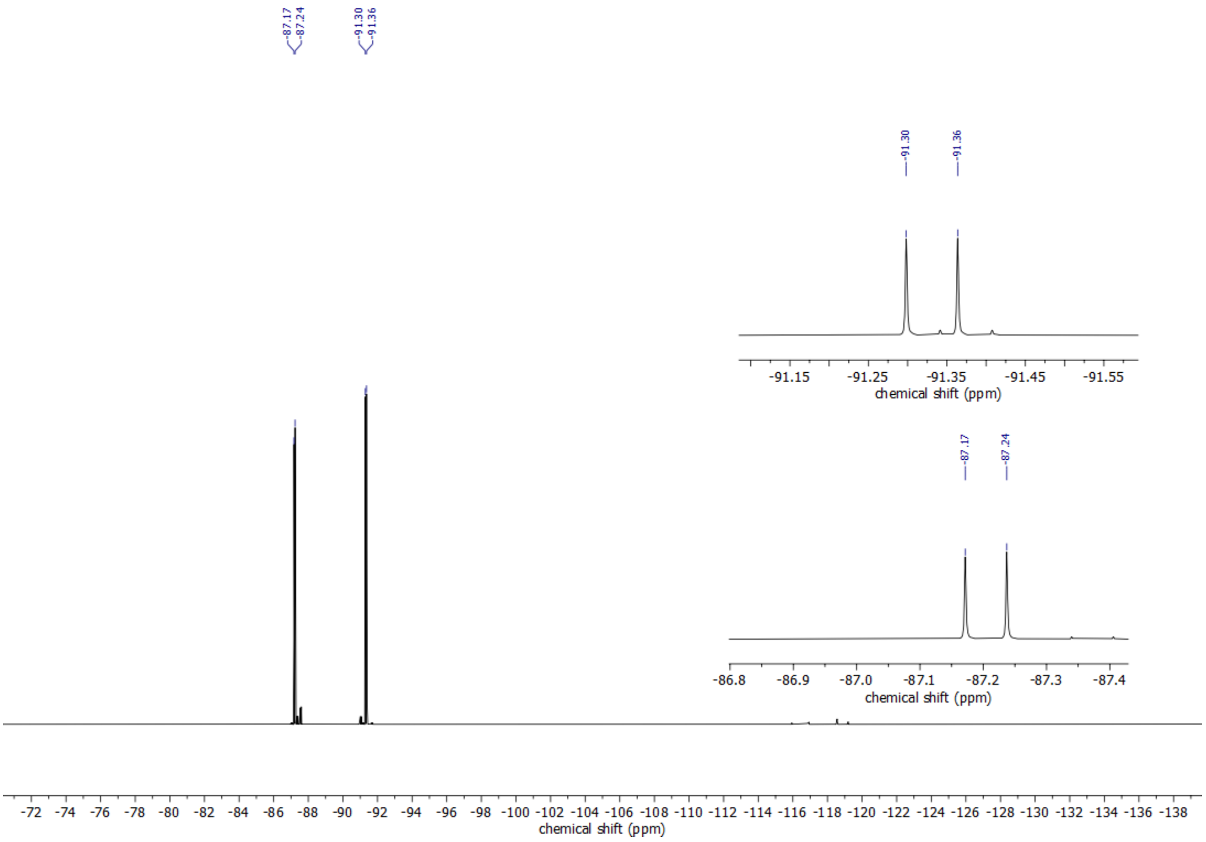

^{1}H-^{1}H COSY NMR (599 MHz, $CDCl_3$)

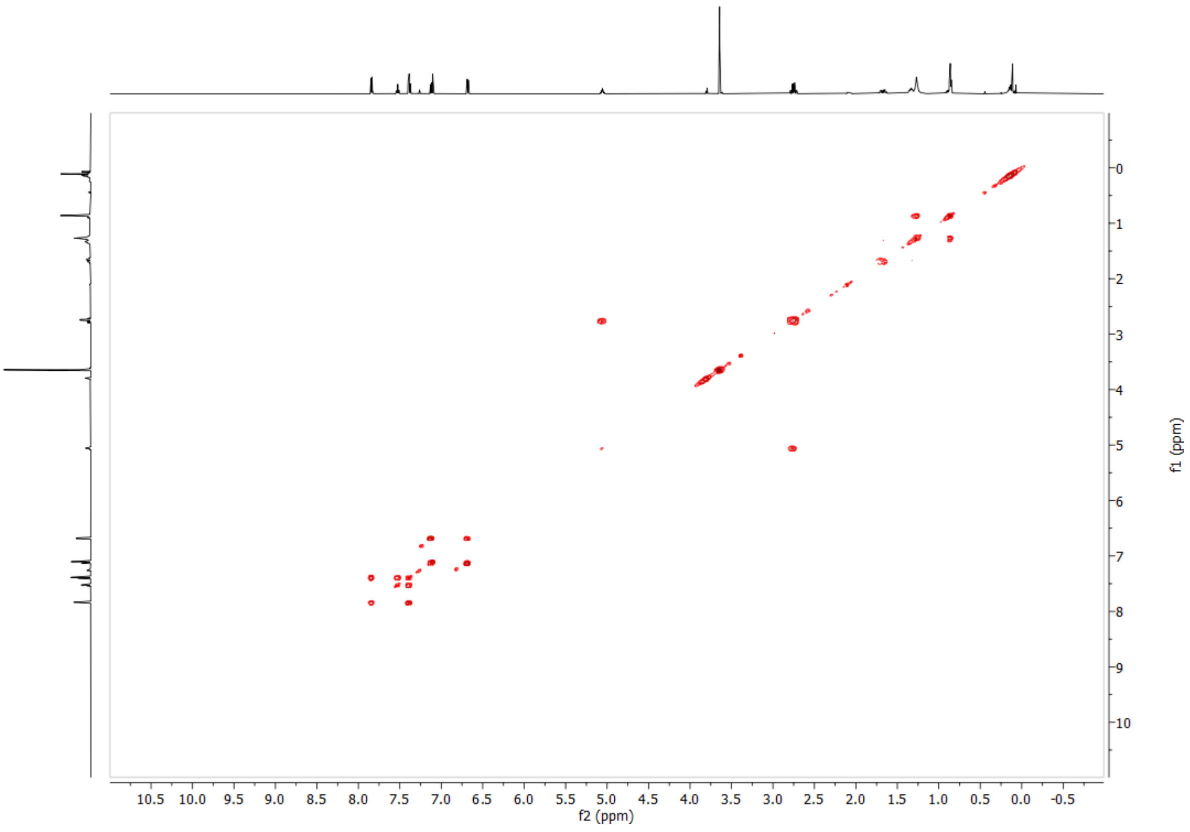

^{1}H-^{13}C HSQC NMR (599 MHz, $CDCl_3$)

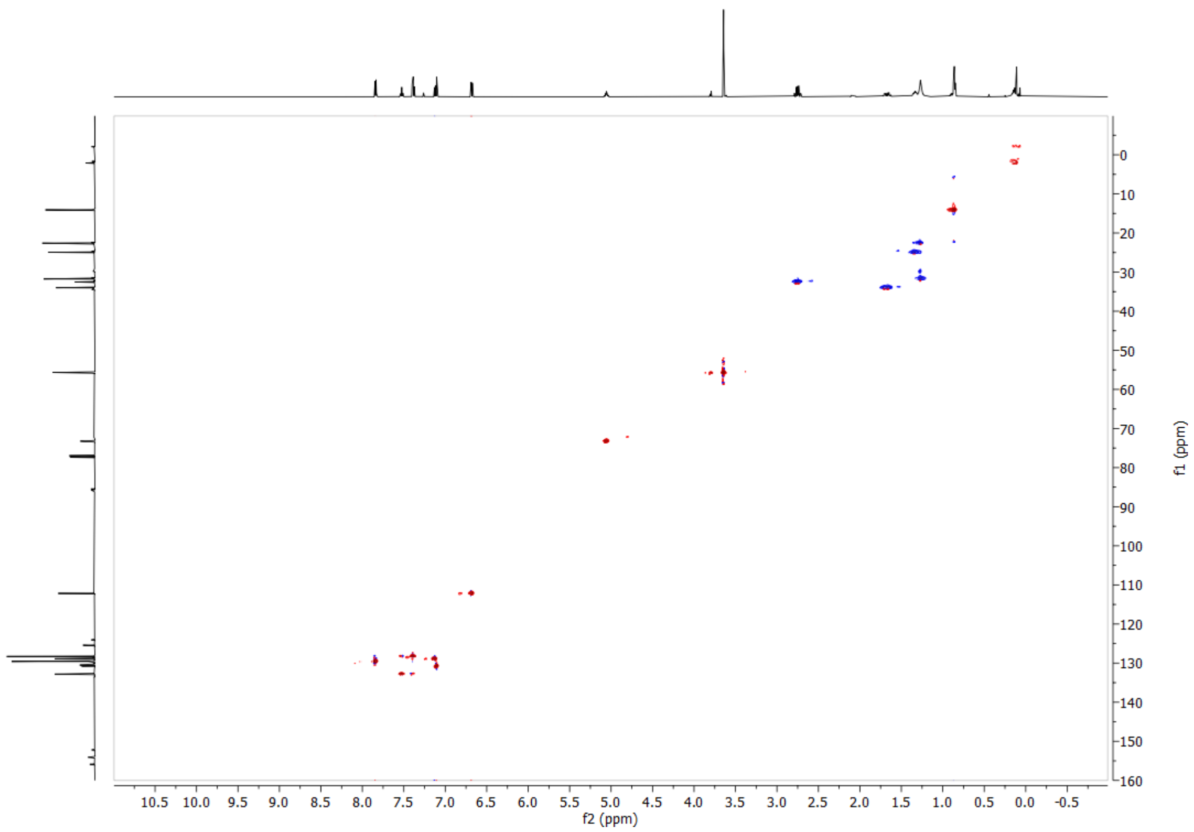

^{1}H-^{13}C HMBC NMR (599 MHz, $CDCl_3$)

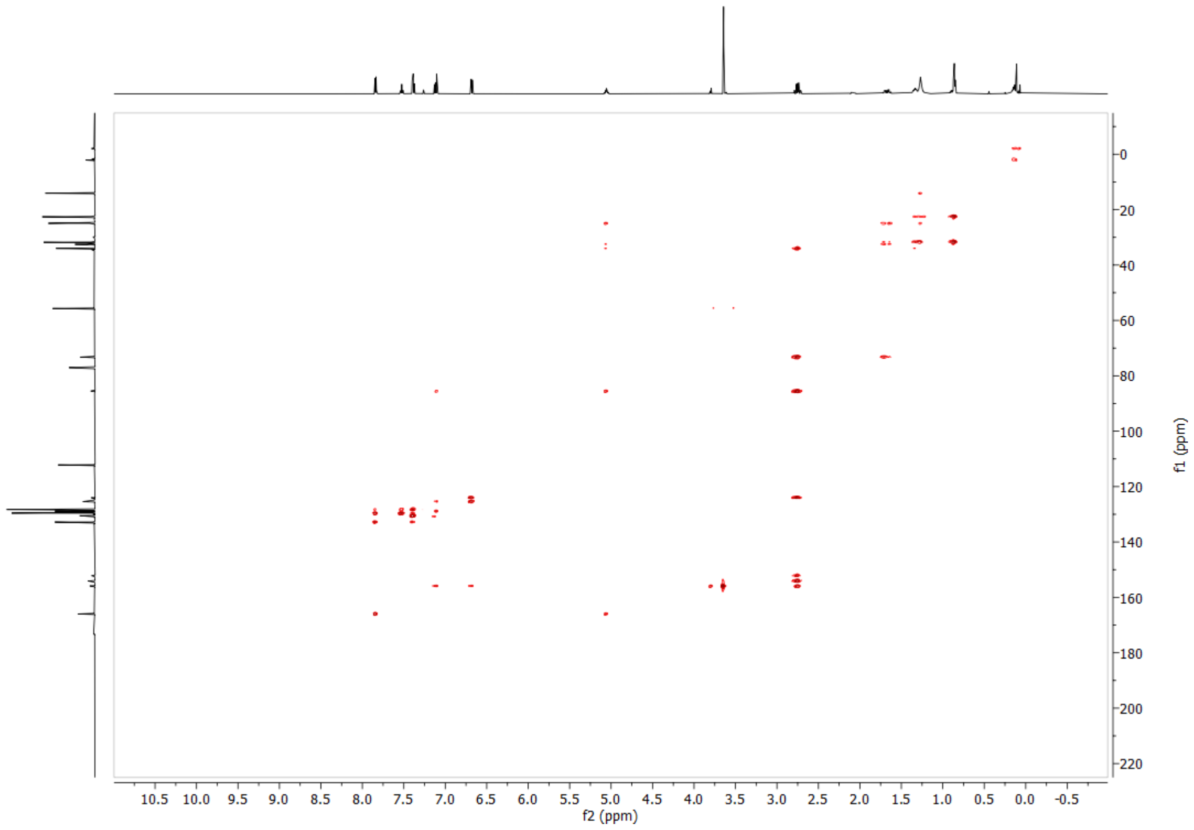

^{1}H NMR (500 MHz, $CDCl_3$)

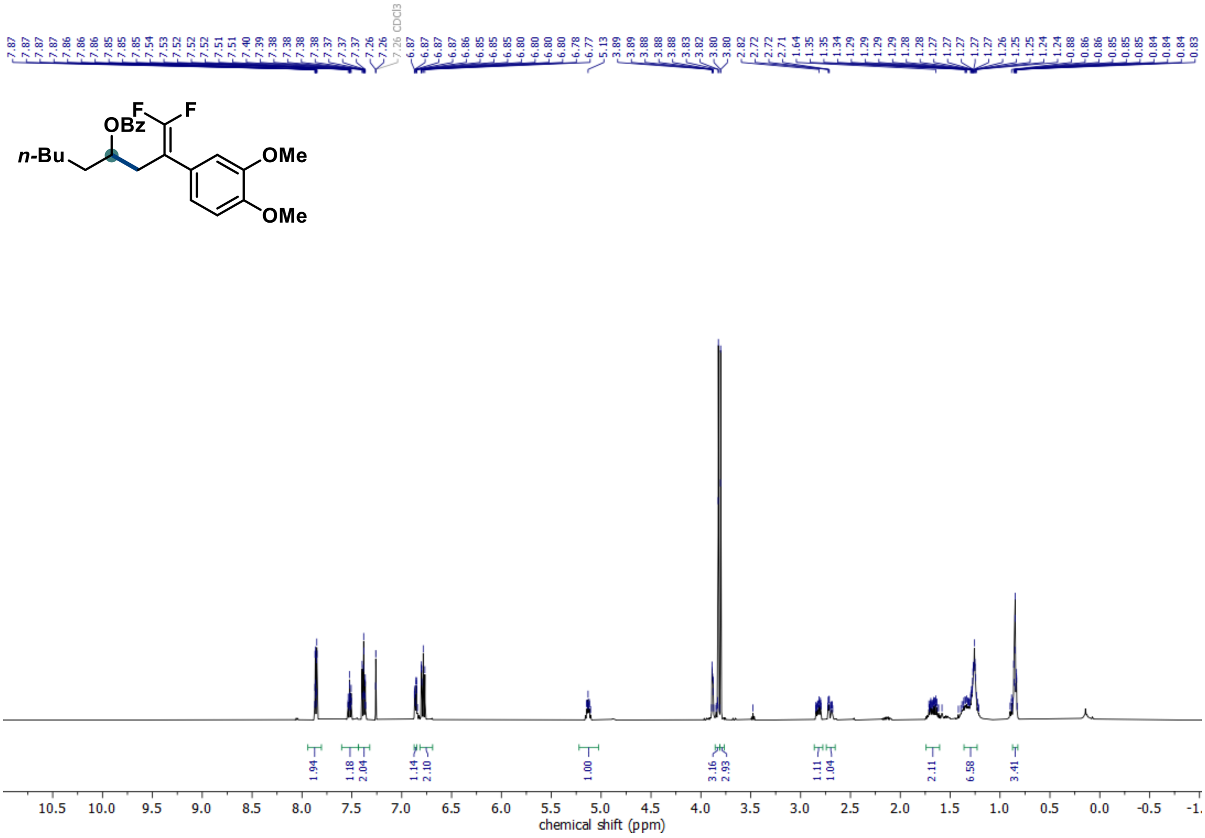

$^{13}C\{^{1}H, ^{19}F\}$ NMR (126 MHz, $CDCl_3$)

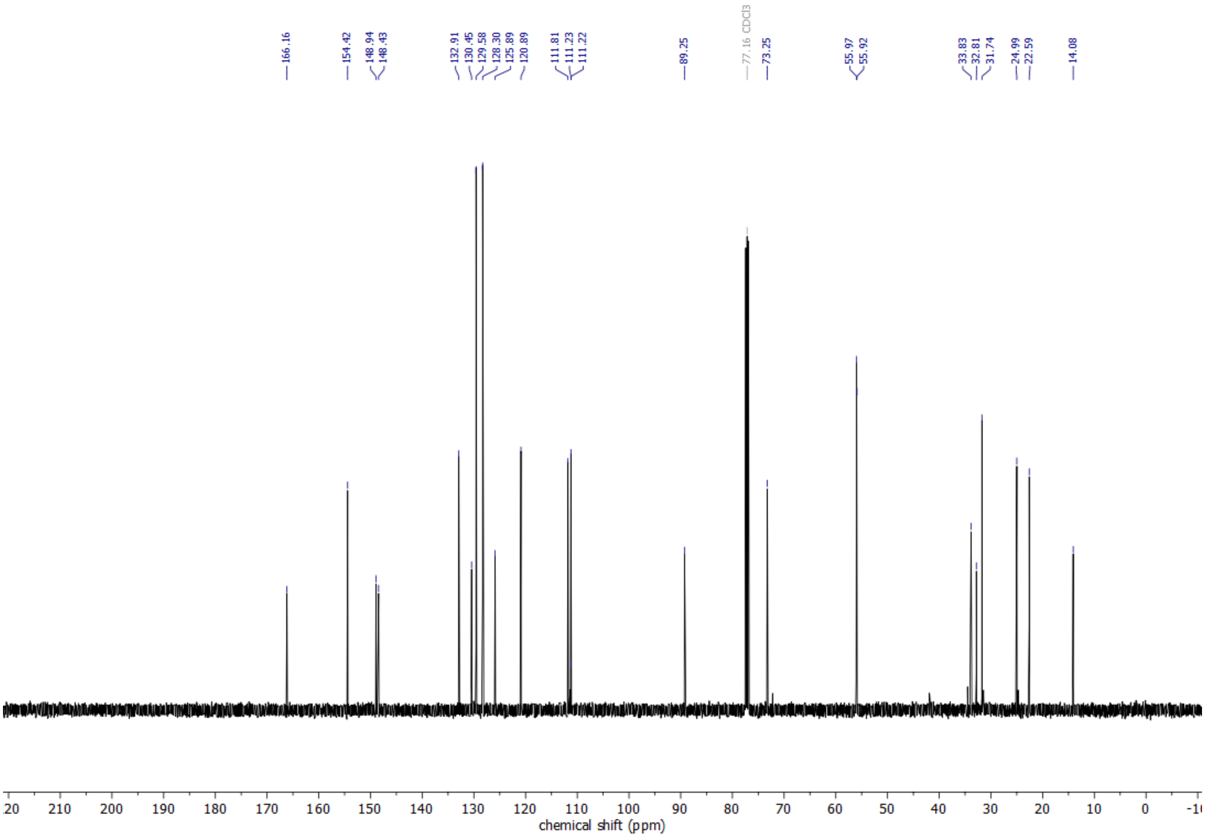

^{19}F{^{1}H} NMR (470 MHz, CDCl$_3$)

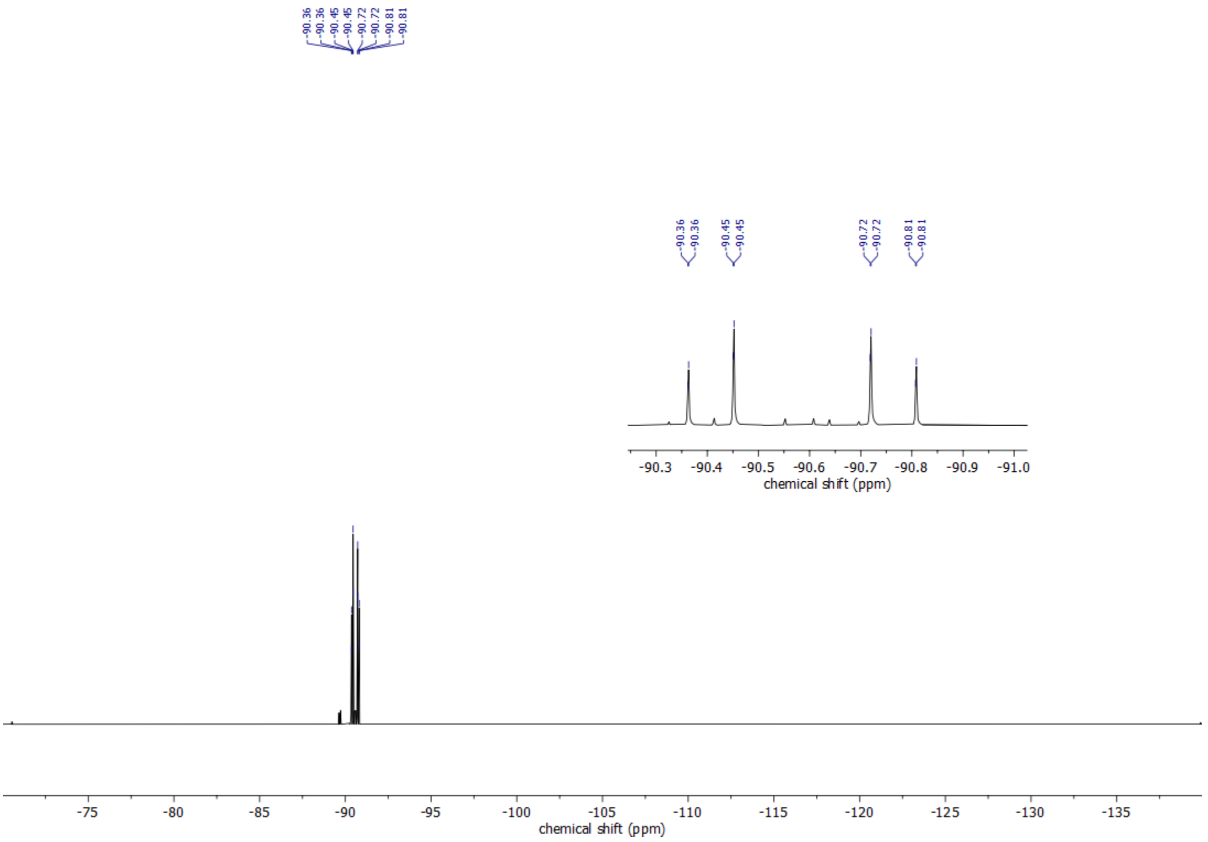

^{1}H NMR (500 MHz, $CDCl_3$)

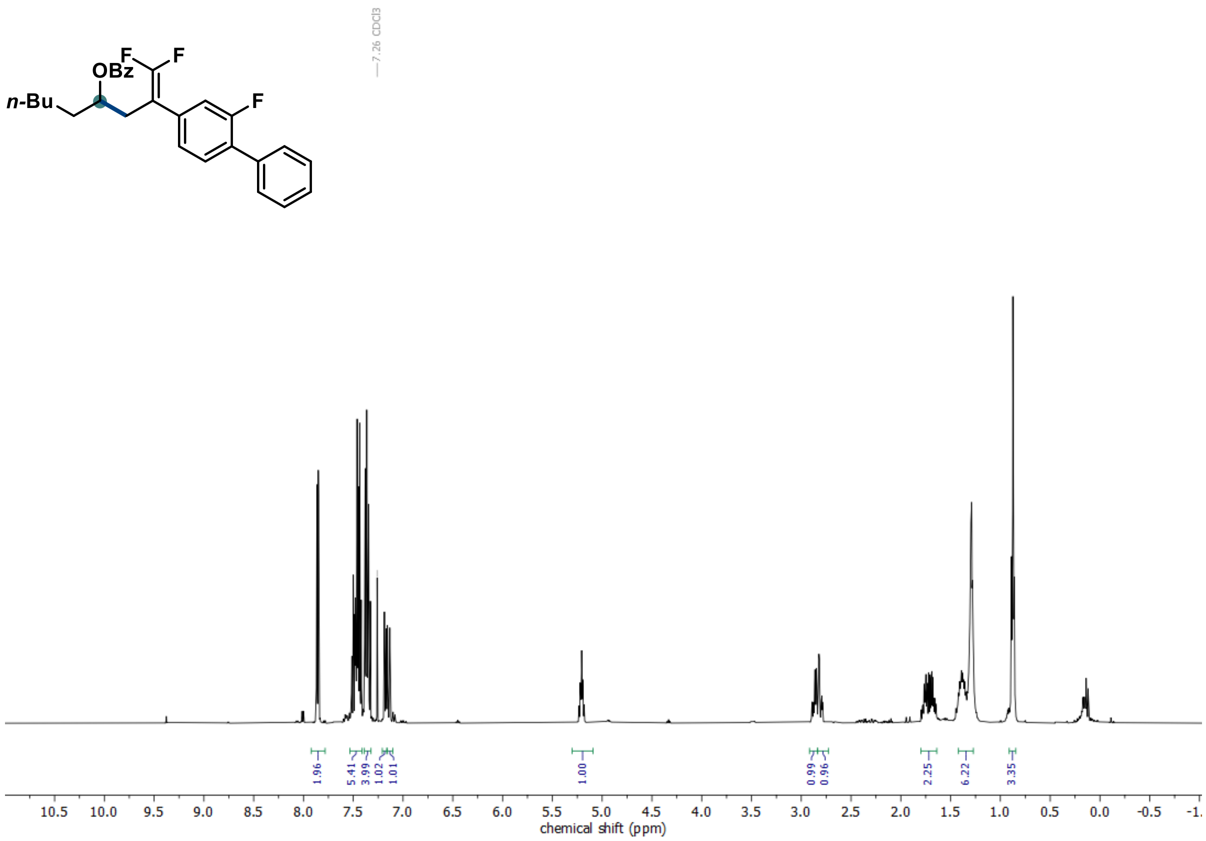

^{13}C{^{1}H} NMR (126 MHz, $CDCl_3$)

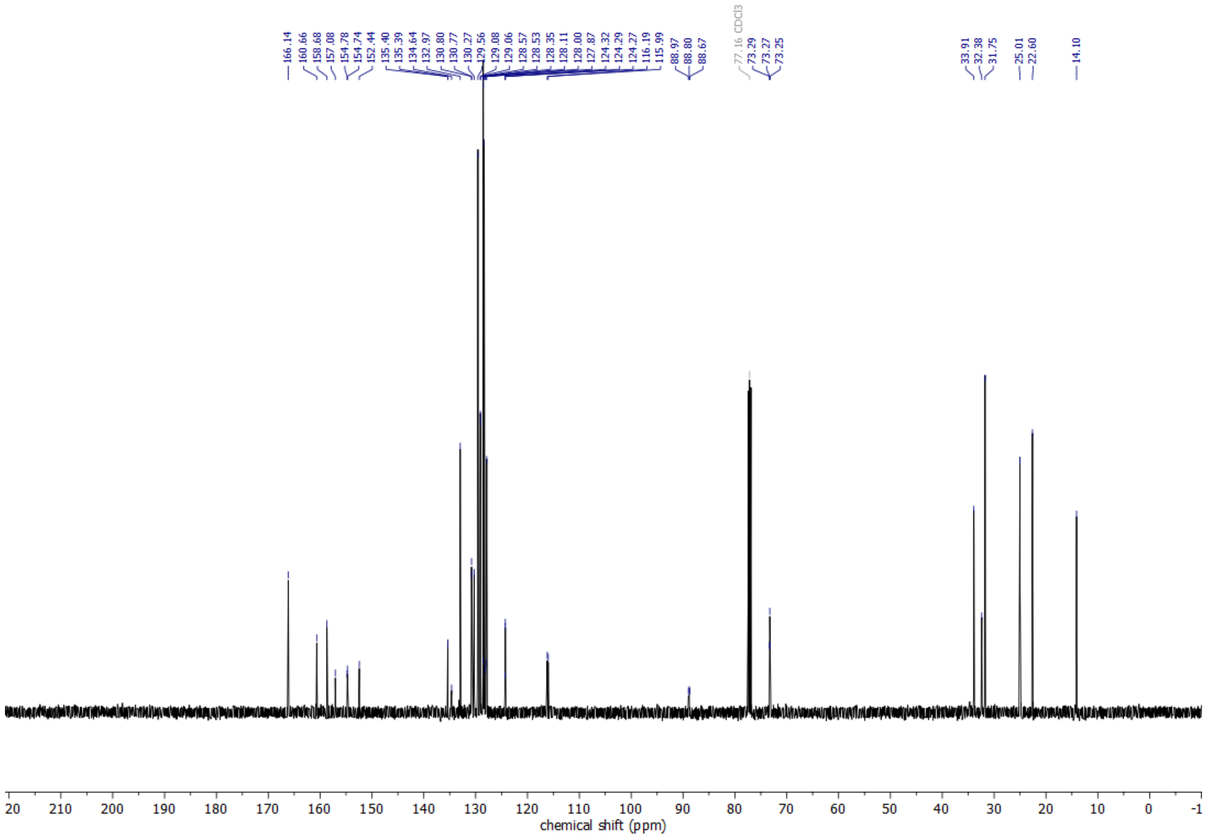

$^{13}C\{^1H, ^{19}F\}$ NMR (126 MHz, CDCl$_3$)

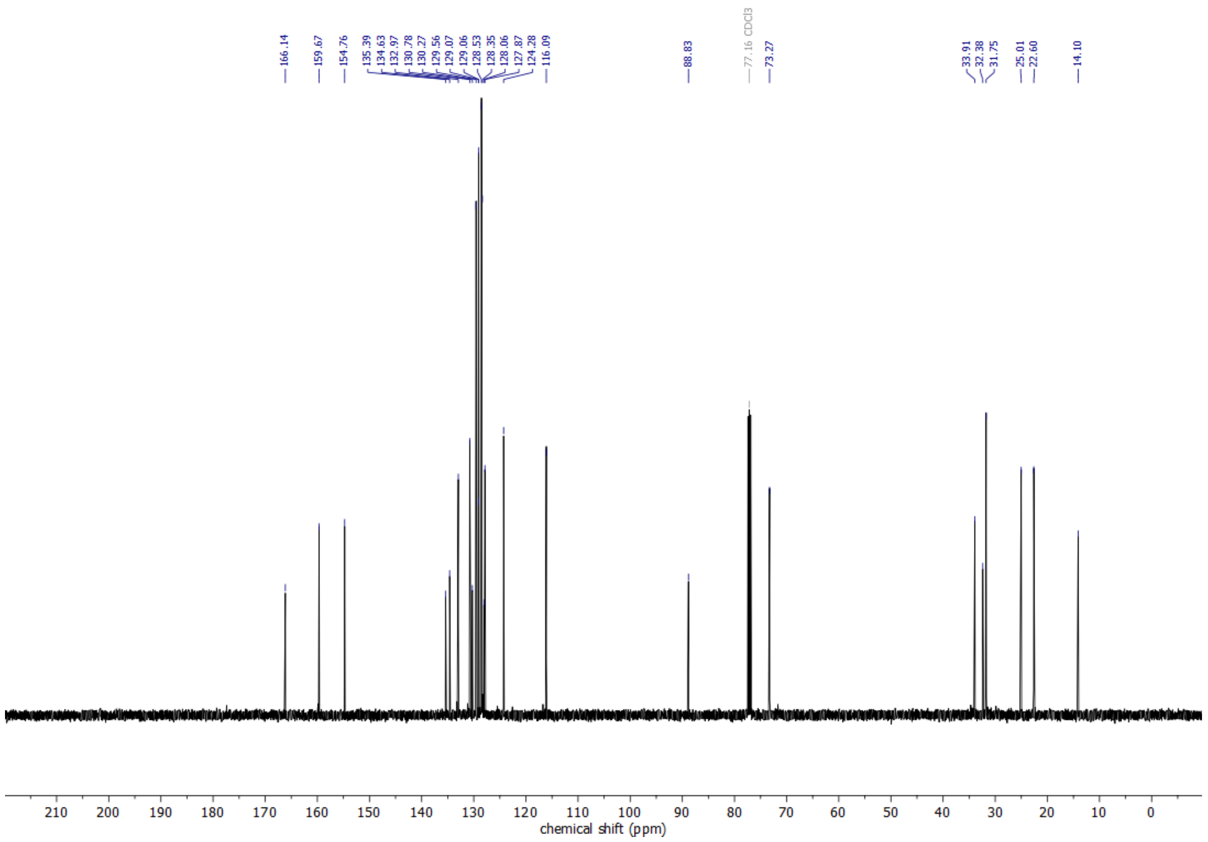

^{19}F NMR (470 MHz, CDCl$_3$)

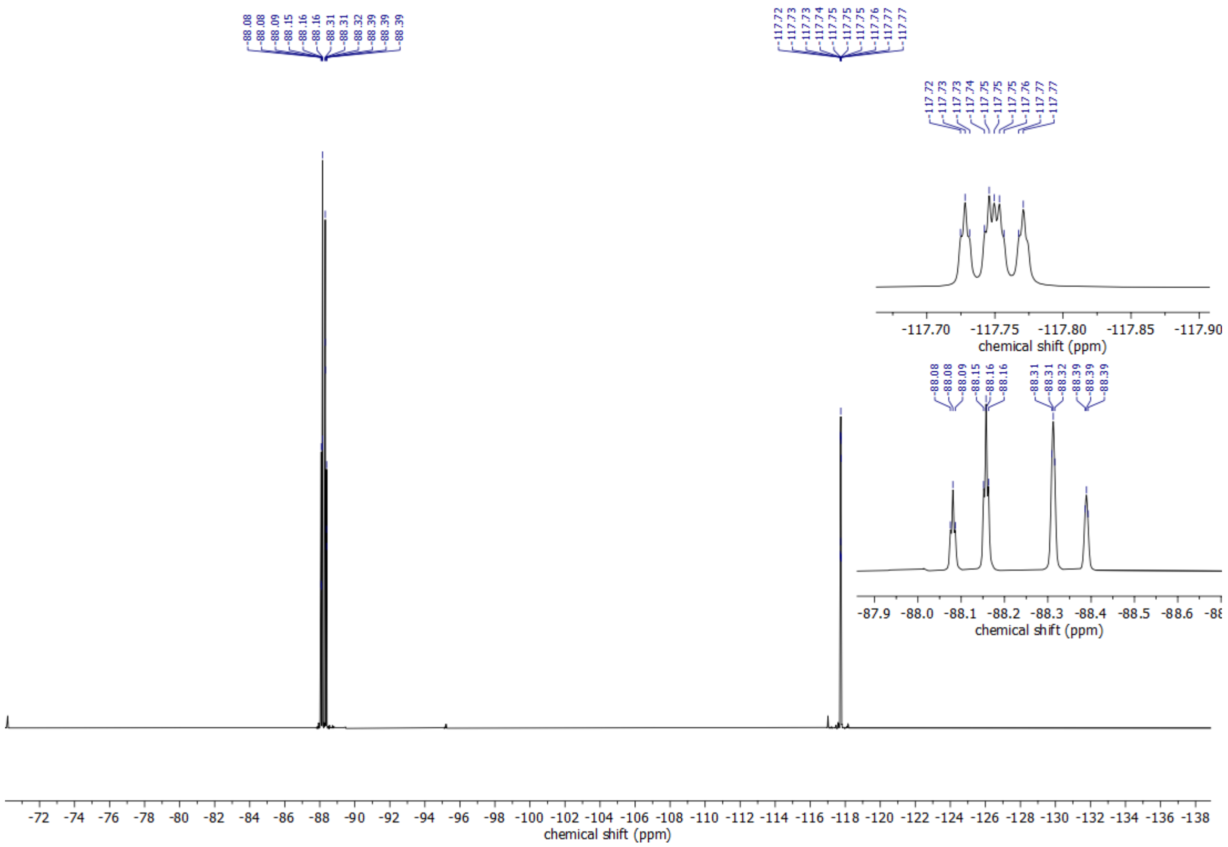

$^{19}F\{^{1}H\}$ NMR (470 MHz, $CDCl_3$)

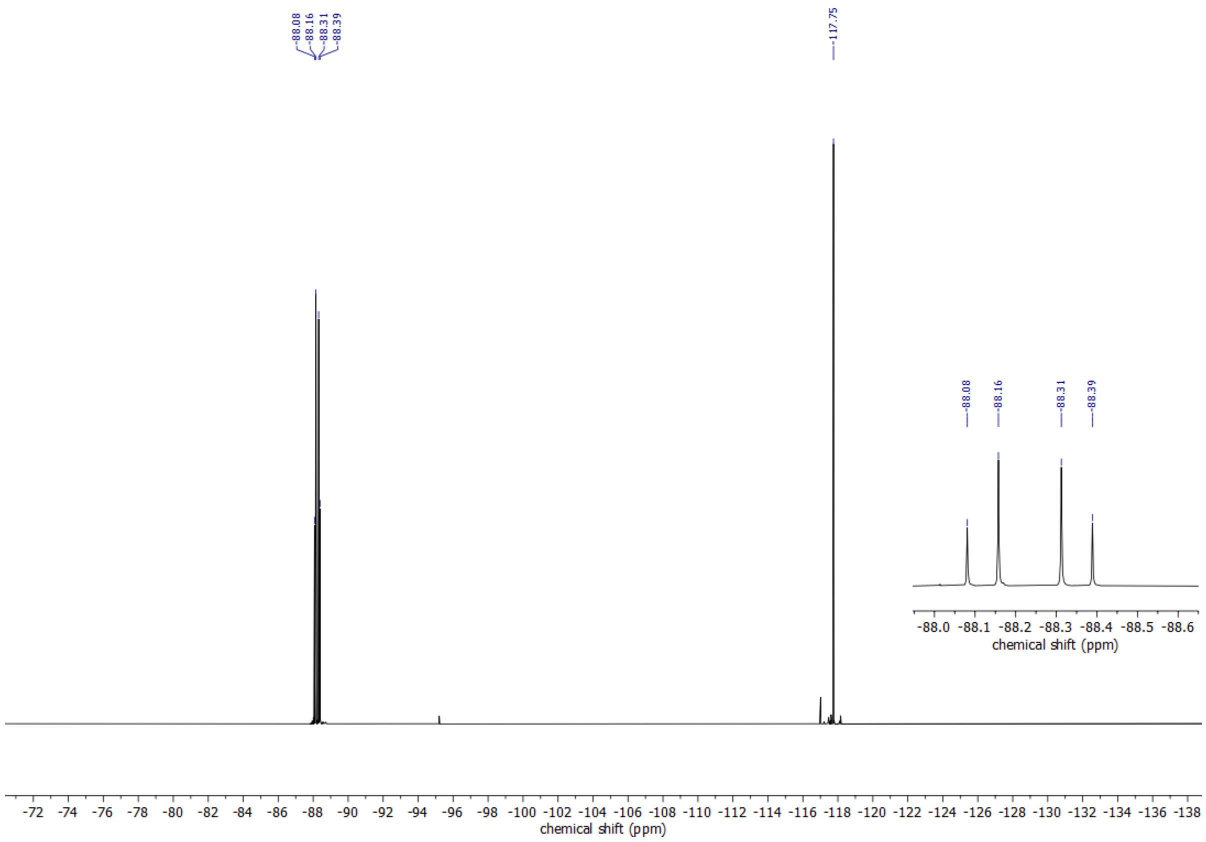

^{1}H NMR (400 MHz, $CDCl_3$)

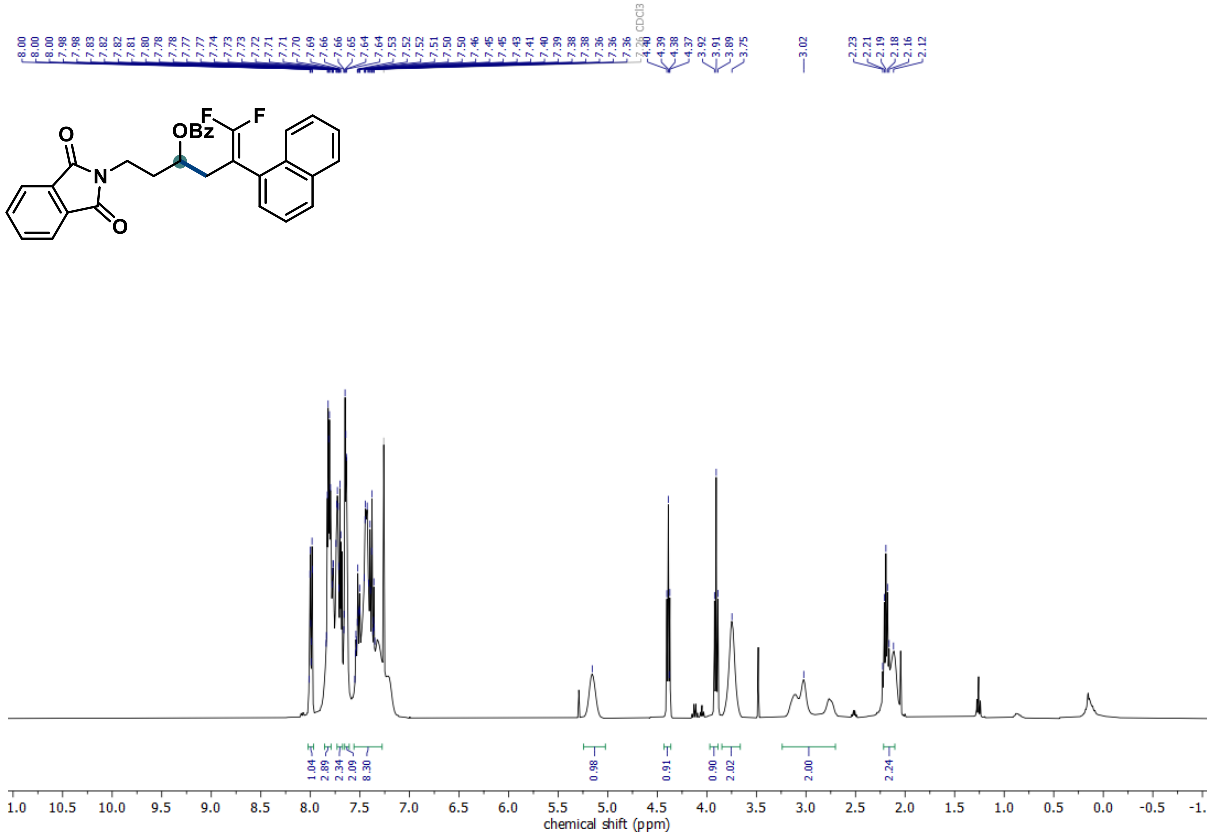

^{13}C{^{1}H} NMR (101 MHz, $CDCl_3$)

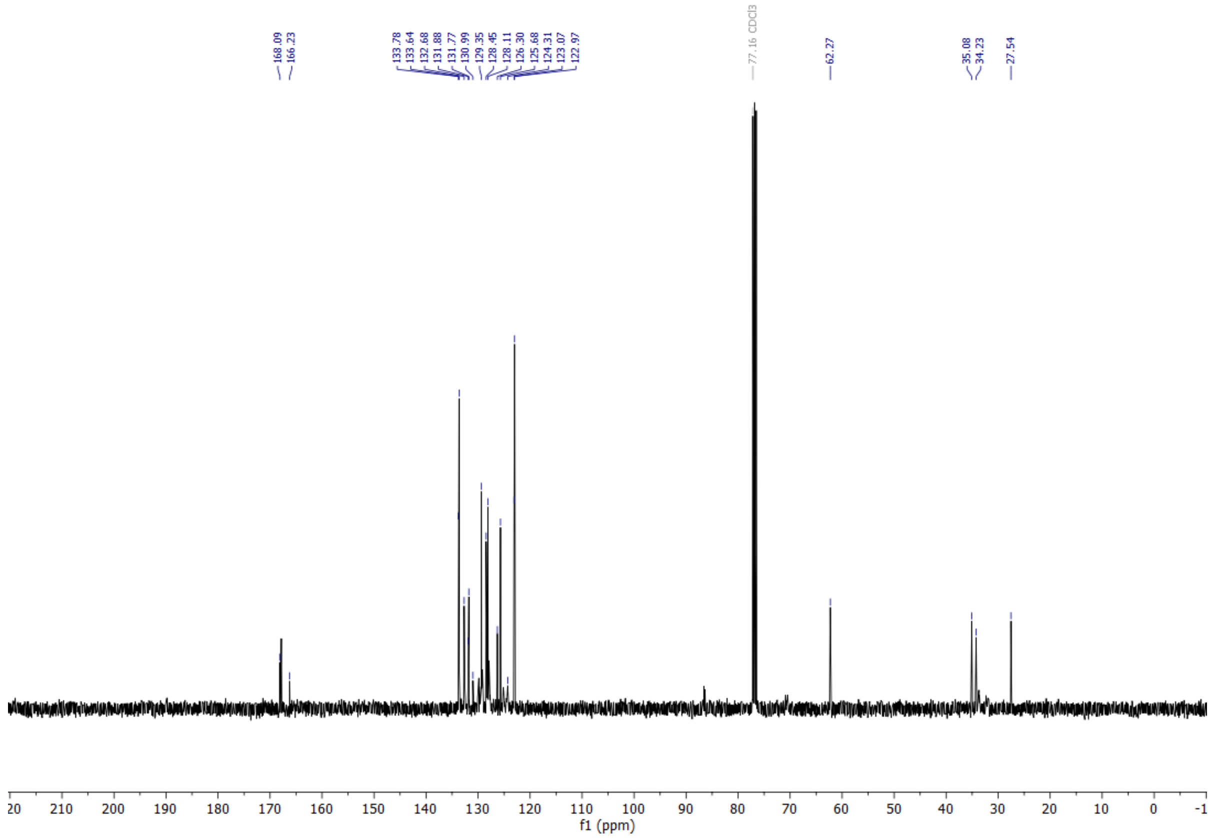

$^{19}F\{^{1}H\}$ NMR (377 MHz, $CDCl_3$)

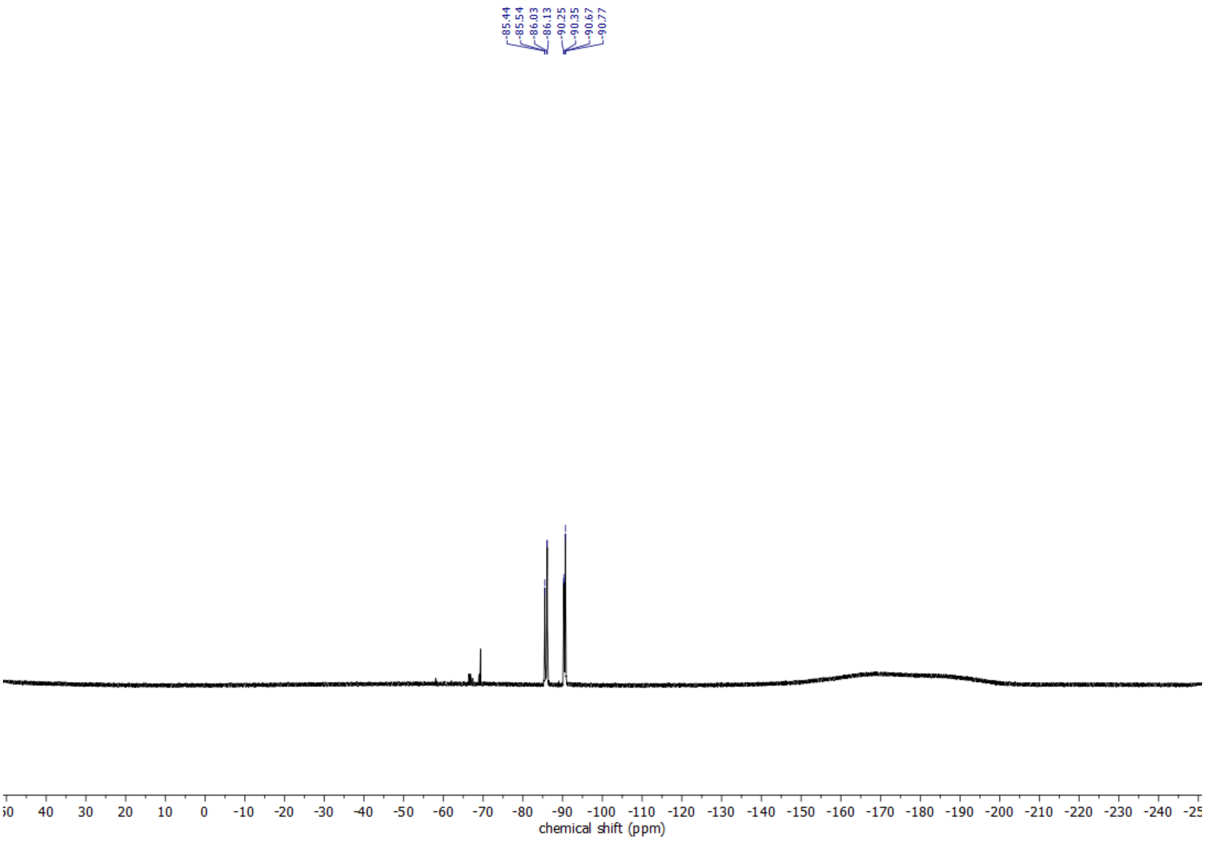

^{1}H NMR (500 MHz, $CDCl_3$)

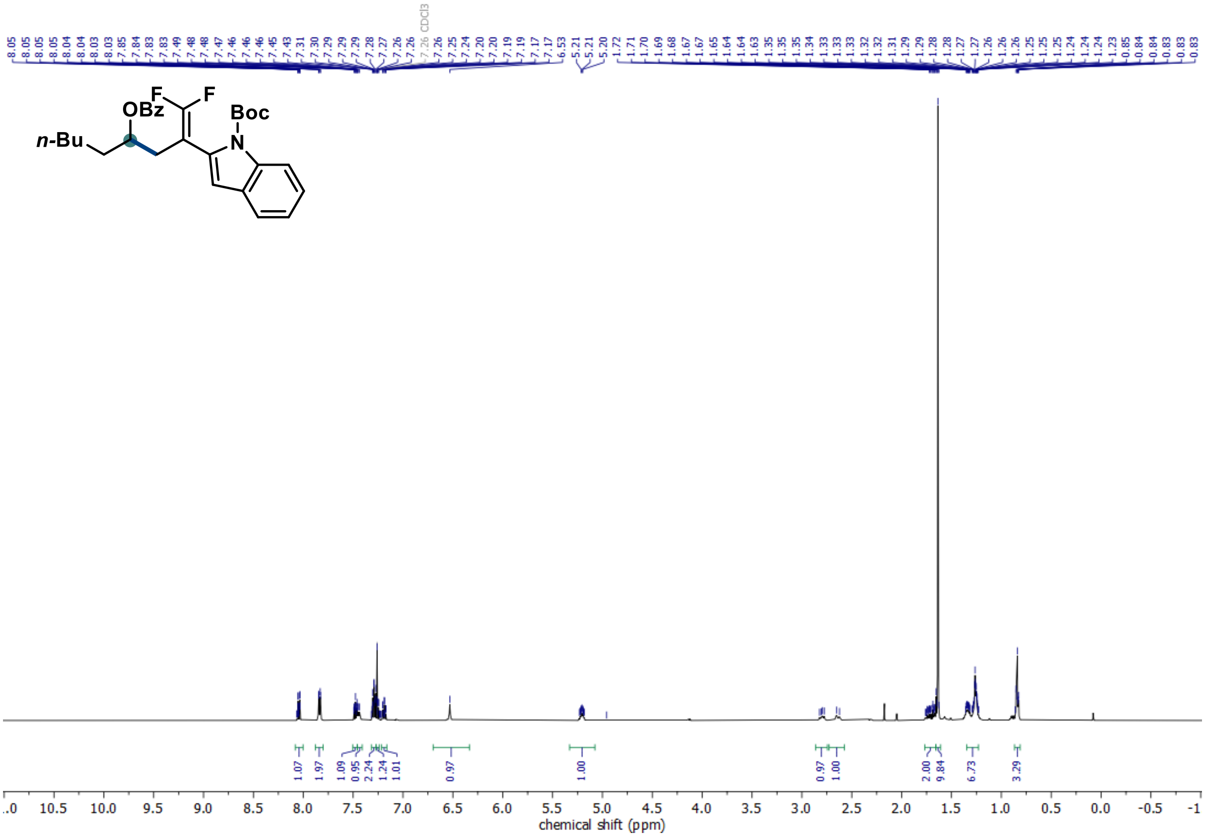

$^{13}C\{^{1}H, ^{19}F\}$ NMR (126 MHz, $CDCl_3$)

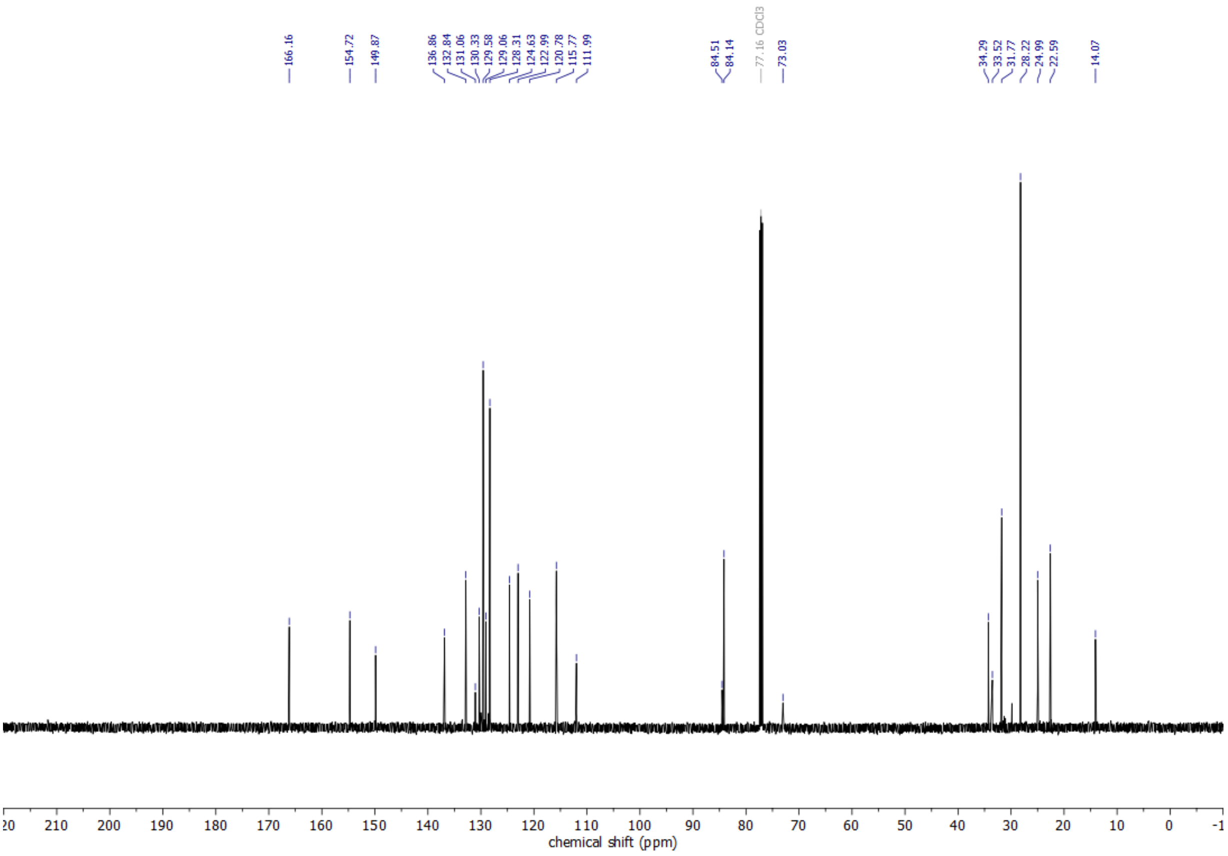

$^{19}F\{^1H\}$ NMR (470 MHz, $CDCl_3$)

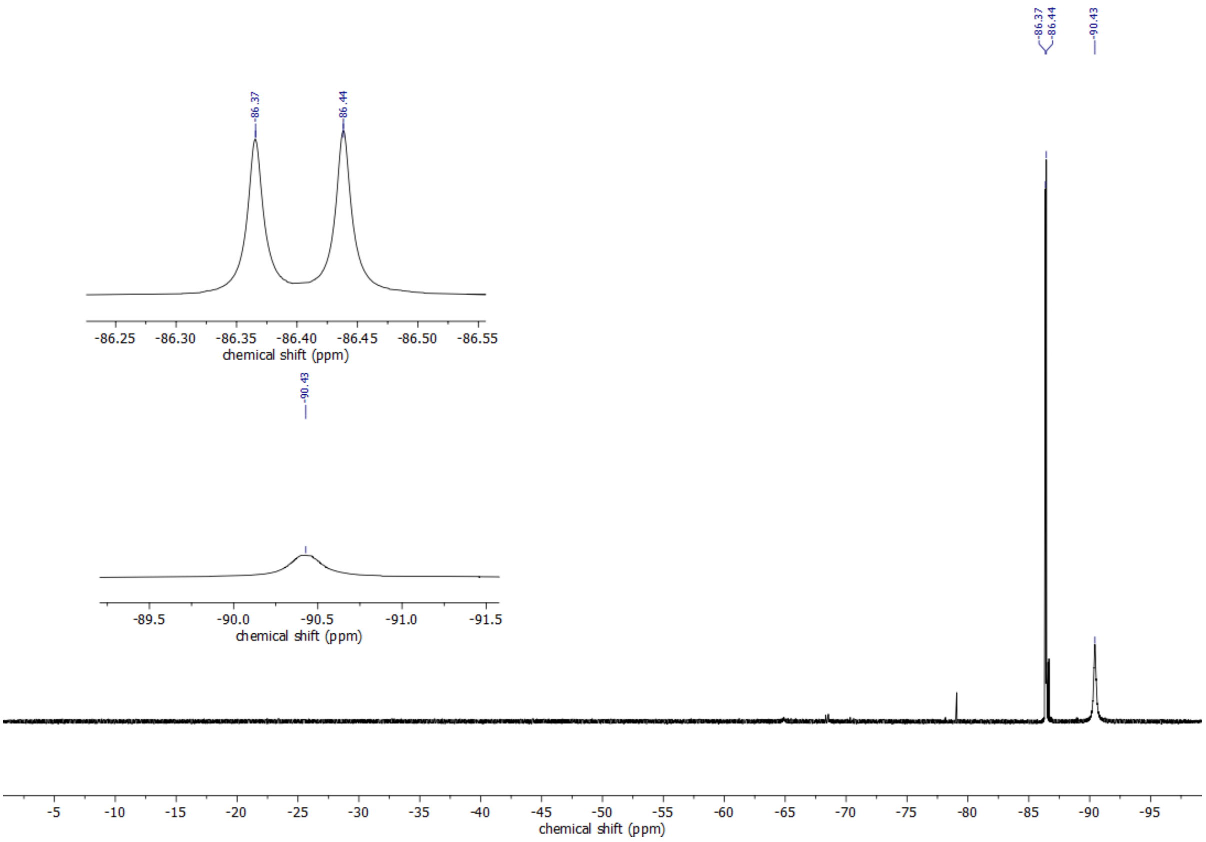

^{1}H NMR (500 MHz, $CDCl_3$)

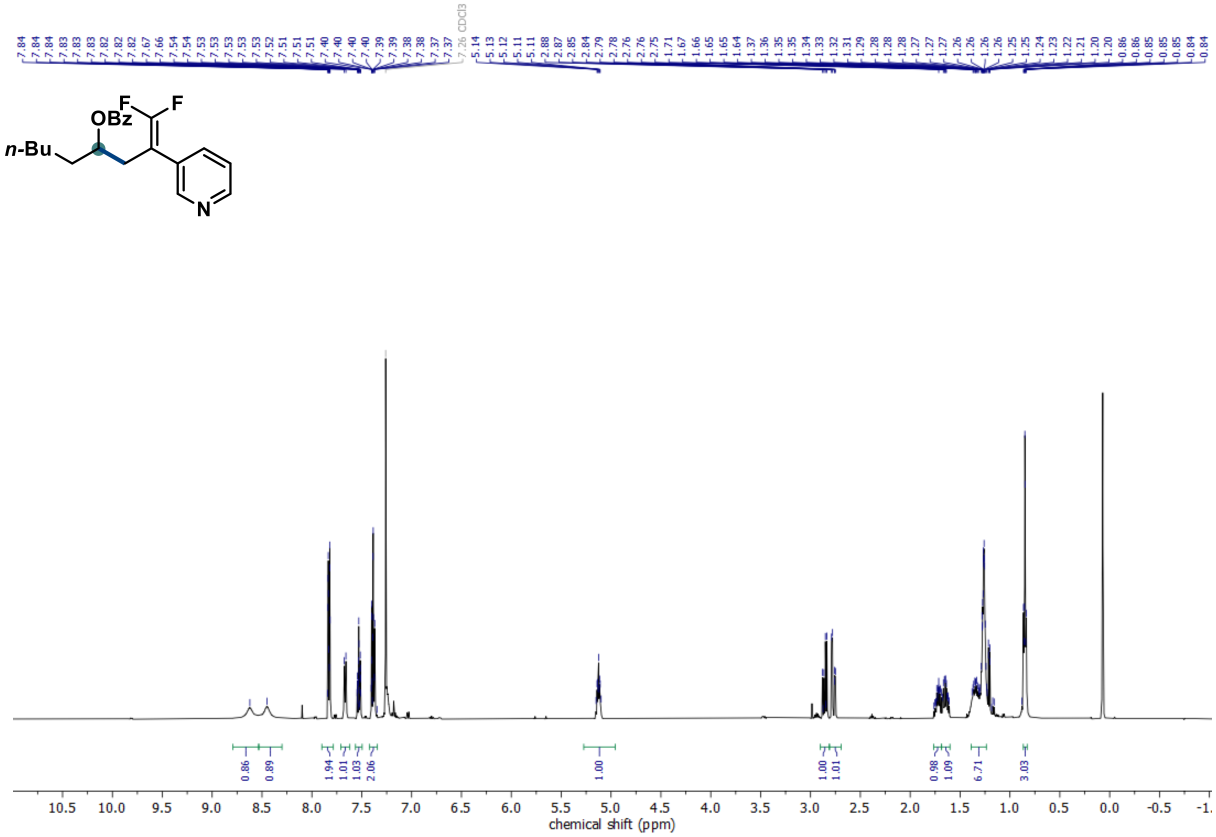

$^{13}C\{^{1}H, ^{19}F\}$ NMR (126 MHz, $CDCl_3$)

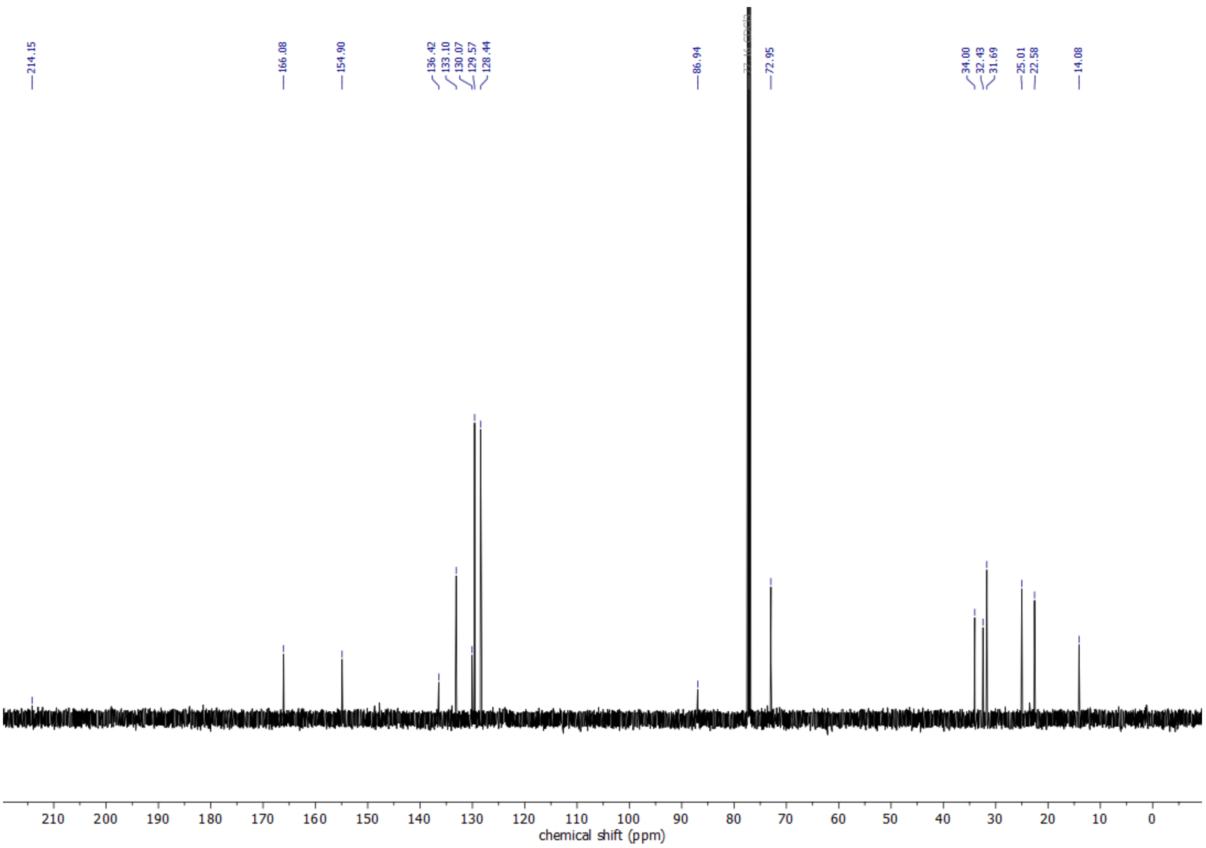

$^{19}F\{^{1}H\}$ NMR (470 MHz, $CDCl_3$)

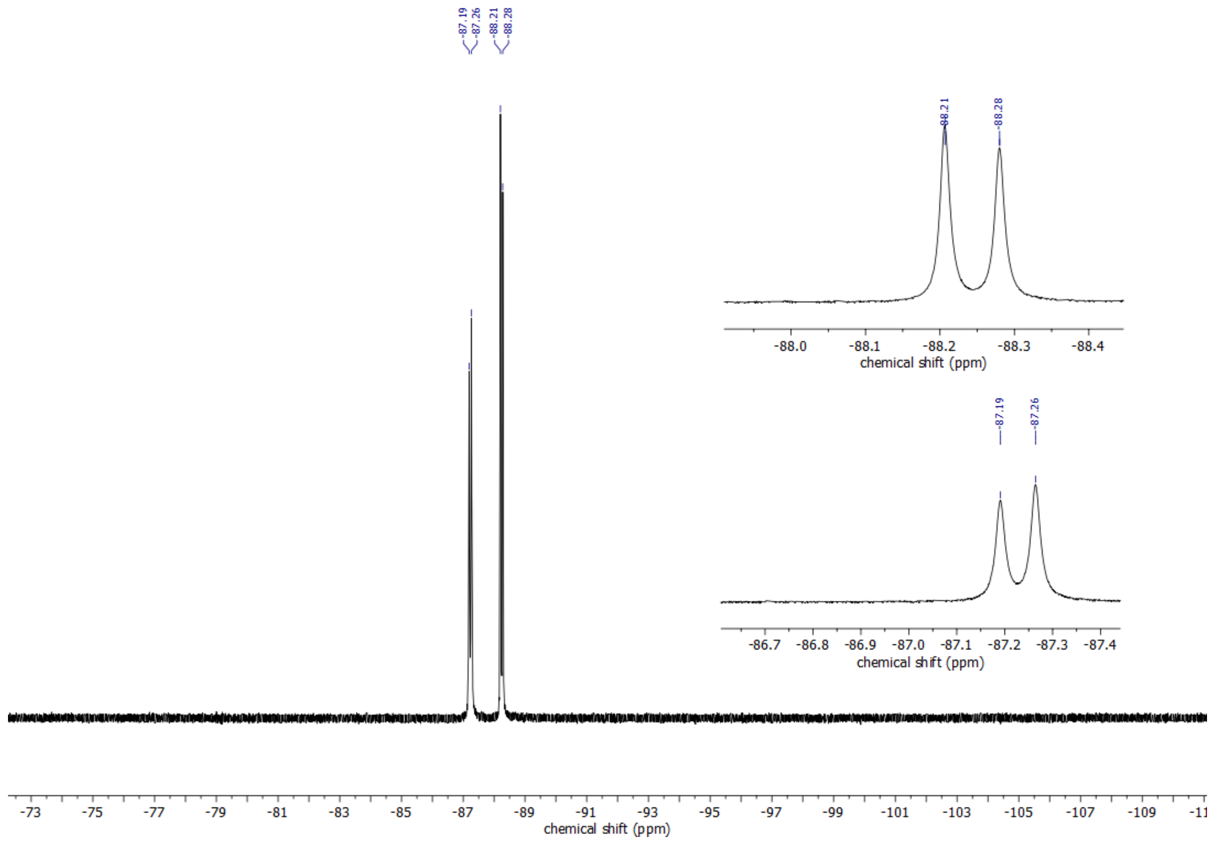

^{1}H NMR (400 MHz, $CDCl_3$)

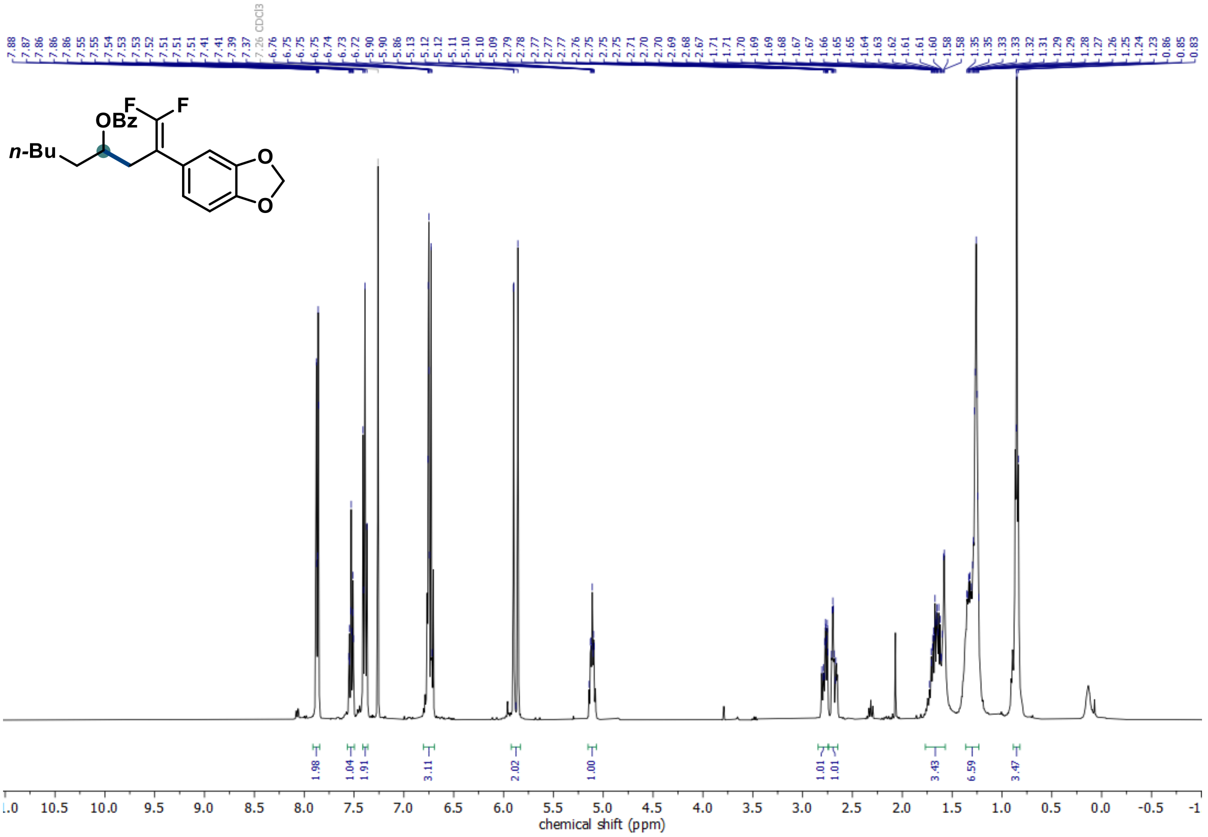

$^{13}C\{^{1}H\}$ NMR (101 MHz, $CDCl_3$)

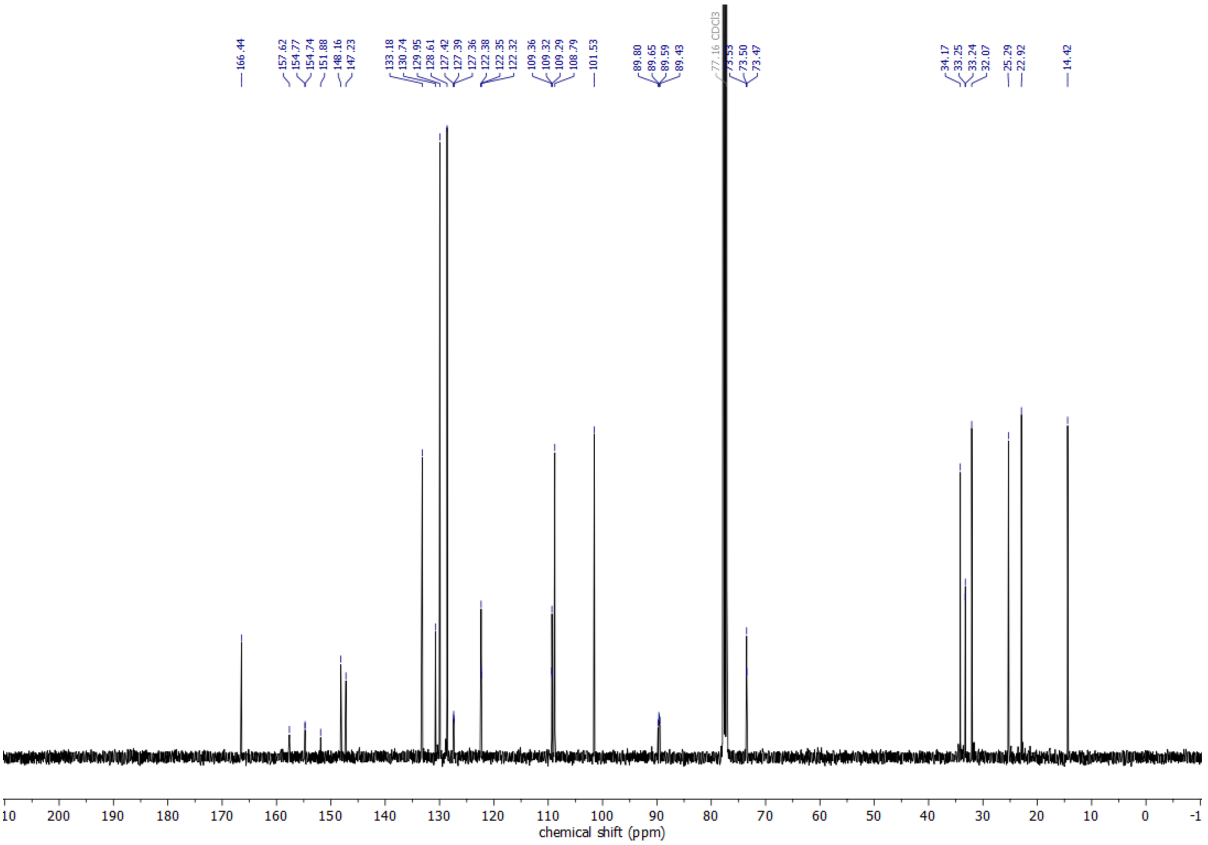

$^{19}F\{^1H\}$ NMR (377 MHz, CDCl$_3$)

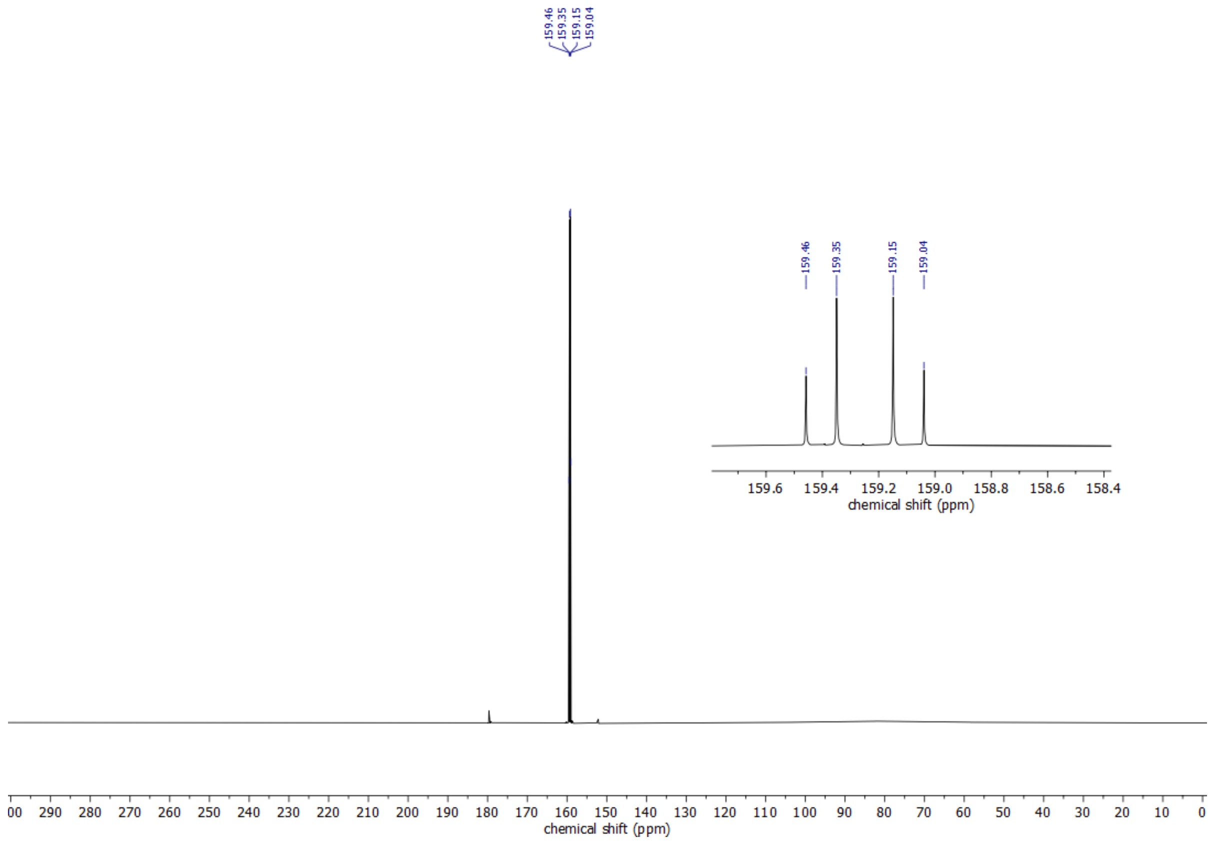

7. References

[1] R. K. Harris, E. D. Becker, S. M. Cabral de Menezes, R. Goodfellow, P. Granger, *Pure Appl. Chem.* **2001**, *73*, 1795–1818.

[2] W. C. Still, M. Kahn, A. Mitra, *J. Org. Chem.* **1978**, *14*, 2923–2925.

[3] A. B. Pangborn, M. A. Giardello, R. H. Grubbs, R. K. Rosen, F. J. Timmers, *Organometallics* **1996**, *15*, 1518–1520.

[4] H.-M. Huang, P. Bellotti, P.-P. Chen, K. N. Houk, F. Glorius, *Nat. Synth.* **2022**, *1*, 59–68.

[5] M. N. Lavagnino, T. Liang, D. W. C. MacMillan, *Proc. Natl. Acad. Sci. U. S. A.* **2020**, *117*, 21058–21064.

[6] V. J. Shiner, M. W. Ensinger, R. D. Rutkowske, *J. Am. Chem. Soc.* **1987**, *109*, 804–809.

[7] H.-M. Huang, P. Bellotti, P. M. Pflüger, J. L. Schwarz, B. Heidrich, F. Glorius, *J. Am. Chem. Soc.* **2020**, *142*, 10173–10183.

[8] L.-F. Wu, J.-W. Yao, X. Zhang, S.-Y. Liu, Z.-N. Zhuang, K. Wei, *Org. Lett.* **2021**, *23*, 6237–6241.

[9] P.-F. Yang, W. Shu, *Org. Lett.* **2020**, *22*, 6203–6208.

[10] H. Li, Y. Hou, C. Liu, Z. Lai, L. Ning, R. Szostak, M. Szostak, J. An, *Org. Lett.* **2020**, *22*, 1249–1253.

[11] J. M. Chong, M. A. Heuft, P. Rabbat, *J. Org. Chem.* **2000**, *65*, 5837–5838.

[12] W. H. Pearson, D. A. Hutta, W. Fang, *J. Org. Chem.* **2000**, *65*, 8326–8332.

[13] M. E. Layton, C. A. Morales, M. D. Shair, *J. Am. Chem. Soc.* **2002**, *124*, 773–775.

[14] N. Kern, T. Dombray, A. Blanc, J.-M. Weibel, P. Pale, *J. Org. Chem.* **2012**, *77*, 9227–9235.

[15] A. V Iosub, Š. Moravčík, C.-J. Wallentin, J. Bergman, *Org. Lett.* **2019**, *21*, 7804–7808.

[16] S. M. Rafferty, J. E. Rutherford, L. Zhang, L. Wang, D. A. Nagib, *J. Am. Chem. Soc.* **2021**, *143*, 5622–5628.

[17] T. A. Bender, R. G. Bergman, K. N. Raymond, F. D. Toste, *J. Am. Chem. Soc.* **2019**, *141*, 11806–11810.

[18] Y. Corre, V. Rysak, F. Capet, J.-P. Djukic, F. Agbossou-Niedercorn, C. Michon, *Chem. – A Eur. J.* **2016**, *22*, 14036–14041.

[19] J. A. Friest, Y. Maezato, S. Broussy, P. Blum, D. B. Berkowitz, *J. Am. Chem. Soc.* **2010**, *132*, 5930–5931.

[20] O. Farooq, M. Marcelli, G. K. S. Prakash, G. A. Olah, *J. Am. Chem. Soc.* **1988**, *110*, 864–867.

[21] D. Christopher Braddock, A. Mahtey, H. S. Rzepa, A. J. P. White, *Chem. Commun.* **2016**, *52*, 11219–11222.

[22] P. C. Too, G. H. Chan, Y. L. Tnay, H. Hirao, S. Chiba, *Angew. Chemie Int. Ed.* **2016**, *55*, 3719–3723.

[23] H.-M. Huang, P. Bellotti, C. G. Daniliuc, F. Glorius, *Angew. Chemie Int. Ed.* **2021**, *60*, 2464–2471.

[24] F. Chen, X. Xu, Y. He, G. Huang, S. Zhu, *Angew. Chemie Int. Ed.* **2020**, *59*, 5398–5402.

[25] M. B. Johansen, O. R. Gedde, T. S. Mayer, T. Skrydstrup, *Org. Lett.* **2020**, *22*, 4068–4072.

[26] Y.-Q. Guo, R. Wang, H. Song, Y. Liu, Q. Wang, *Org. Lett.* **2020**, *22*, 709–713.

[27] C. Yao, S. Wang, J. Norton, M. Hammond, *J. Am. Chem. Soc.* **2020**, *142*, 4793–4799.

[28] T. A. Hamlin, C. B. Kelly, R. M. Cywar, N. E. Leadbeater, *J. Org. Chem.* **2014**, *79*, 1145–1155.

[29] Q.-P. Hu, J. Cheng, Y. Wang, J. Shi, B.-Q. Wang, P. Hu, K.-Q. Zhao, F. Pan, *Org. Lett.* **2021**, *23*, 4457–4462.

[30] S. Meyer, J. Häfliger, M. Schäfer, J. J. Molloy, C. G. Daniliuc, R. Gilmour, *Angew. Chemie - Int. Ed.* **2021**, *60*, 6430–6434.

[31] Q.-P. Hu, J. Cheng, Y. Wang, J. Shi, B.-Q. Wang, P. Hu, K.-Q. Zhao, F. Pan, *Org. Lett.* **2021**, *23*, 4457–4462.

[32] M. Scheepstra, L. Nieto, A. K. H. Hirsch, S. Fuchs, S. Leysen, C. V. Lam, L. In Het Panhuis, C. A. A. Van Boeckel, H. Wienk, R. Boelens, C. Ottmann, L. G. Milroy, L. Brunsveld, *Angew. Chemie - Int. Ed.* **2014**, *53*, 6443–6448.

[33] P. Czerwiński, E. Molga, L. Cavallo, A. Poater, M. Michalak, *Chem. - A Eur. J.* **2016**, *22*, 8089–8094.

[34] Y. Li, B. Zhao, K. Dai, D.-H. Tu, B. Wang, Y.-Y. Wang, Z.-T. Z.-W. Liu, Z.-T. Z.-W. Liu, J. Lu, *Tetrahedron* **2016**, *72*, 5684–5690.

[35] Y. Q. Guo, R. Wang, H. Song, Y. Liu, Q. Wang, *Org. Lett.* **2020**, *22*, 709–713.

[36] P. Fan, C. Zhang, Y. Lan, Z. Lin, L. Zhang, C. Wang, *Chem. Commun.* **2019**, *55*, 12691–12694.

[37] T. Y. Shang, L. H. Lu, Z. Cao, Y. Liu, W. M. He, B. Yu, *Chem. Commun.* **2019**, *55*, 5408–5419.

[38] K. W. Kramarz, J. R. Norton, in *Prog. Inorg. Chem. Vol. 42*, **2007**, pp. 1–65.

[39] X. Wu, J. Riedel, V. M. Dong, *Angew. Chemie - Int. Ed.* **2017**, *56*, 11589–11593.

[40] F. Neese, *WIREs Comput. Mol. Sci.* **2022**, DOI 10.1002/wcms.1606.

[41] Y. Guo, K. Sivalingam, E. F. Valeev, F. Neese, *J. Chem. Phys.* **2016**, *144*, 094111.

[42] A. Schäfer, H. Horn, R. Ahlrichs, *J. Chem. Phys.* **1992**, *97*, 2571–2577.

[43] M. Cossi, N. Rega, G. Scalmani, V. Barone, *J. Comput. Chem.* **2003**, *24*, 669–681.

[44] V. Barone, M. Cossi, *J. Phys. Chem. A* **1998**, *102*, 1995–2001.

[45] S. Grimme, J. Antony, S. Ehrlich, H. Krieg, *J. Chem. Phys.* **2010**, *132*, 154104.

[46] H. G. Roth, N. A. Romero, D. A. Nicewicz, *Synlett* **2016**, *27*, 714–723.

[47] L. Pitzer, F. Schäfers, F. Glorius, *Angew. Chemie Int. Ed.* **2019**, *58*, 8572–8576.
